# Selective Synthesis of Lysine Peptides and the Prebiotically Plausible Synthesis of Catalytically Active Diaminopropionic Acid Peptide Nitriles in Water

**DOI:** 10.1021/jacs.2c12497

**Published:** 2023-01-26

**Authors:** Benjamin Thoma, Matthew W. Powner

**Affiliations:** Department of Chemistry, University College London, 20 Gordon Street, London WC1H 0AJ, U.K.

## Abstract

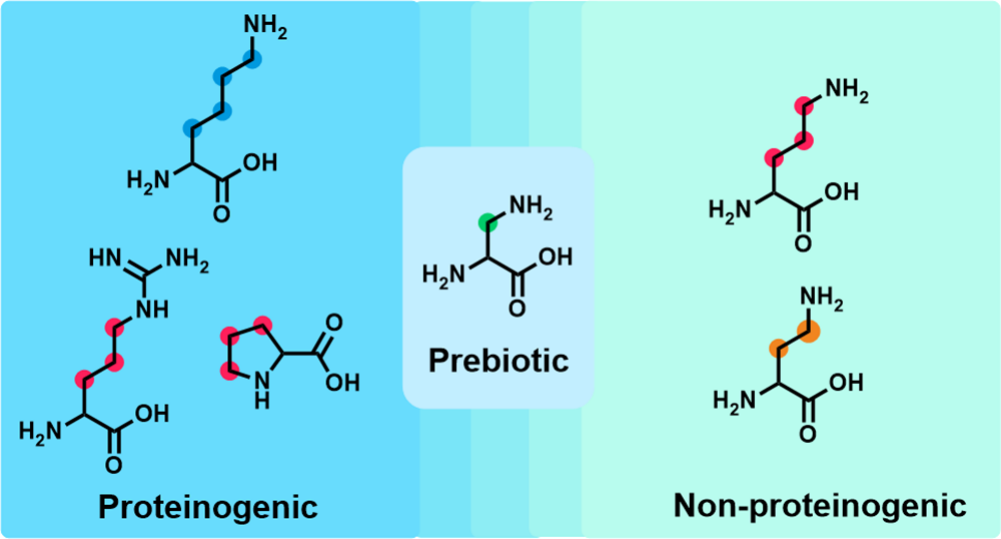

Why life encodes specific proteinogenic amino acids remains an unsolved problem, but a non-enzymatic synthesis that recapitulates biology’s universal strategy of stepwise *N*-to-*C* terminal peptide growth may hold the key to this selection. Lysine is an important proteinogenic amino acid that, despite its essential structural, catalytic, and functional roles in biochemistry, has widely been assumed to be a late addition to the genetic code. Here, we demonstrate that lysine thioacids undergo coupling with aminonitriles in neutral water to afford peptides in near-quantitative yield, whereas non-proteinogenic lysine homologues, ornithine, and diaminobutyric acid cannot form peptides due to rapid and quantitative cyclization that irreversibly blocks peptide synthesis. We demonstrate for the first time that ornithine lactamization provides an absolute differentiation of lysine and ornithine during (non-enzymatic) *N*-to-*C*-terminal peptide ligation. We additionally demonstrate that the shortest lysine homologue, diaminopropionic acid, undergoes effective peptide ligation. This prompted us to discover a high-yielding prebiotically plausible synthesis of the diaminopropionic acid residue, by peptide nitrile modification, through the addition of ammonia to a dehydroalanine nitrile. With this synthesis in hand, we then discovered that the low basicity of diaminopropionyl residues promotes effective, biomimetic, imine catalysis in neutral water. Our results suggest diaminopropionic acid, synthesized by peptide nitrile modification, can replace or augment lysine residues during early evolution but that lysine’s electronically isolated sidechain amine likely provides an evolutionary advantage for coupling and coding as a preformed monomer in monomer-by-monomer peptide translation.

## Introduction

The universal nature of the genetic code, and the specific amino acids encoded, suggests its deep-seated origin.^1−4^ However, how life came to use the particular set of proteinogenic amino acids remains a central challenge within prebiotic chemistry.^5−19^ A rationalization of why the proteinogenic amino acids are as such, and not otherwise, may hold essential clues to elucidating the chemical transition from abiotic chemistry to the first living system(s) and enable a better understanding of life itself.^3,20−24^ Additionally, chemical studies into whether alternative amino acids were available at the origins of life, that were later supplanted or shorn from biology, are necessary to understand both the functional capability of early peptides and which privileged sidechains (if any) in extant life are a remnant of prebiotic chemistry.^3,25,26^

**Figure 1:**
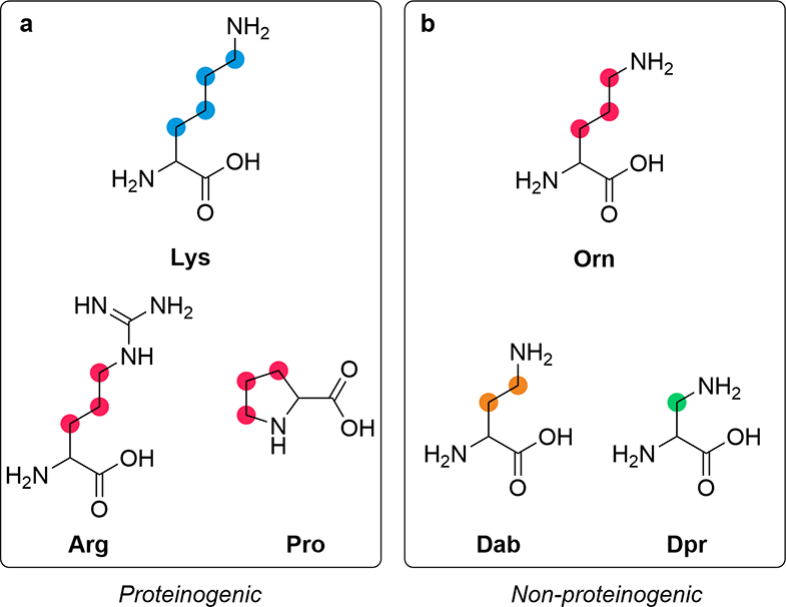
Structural comparison between lysine homologues. (a) The structures of proteinogenic amino acids Lys-OH, Arg-OH, and Pro-OH and (b) the structures non-proteinogenic amino acids Orn-OH, Dab-OH, and Dpr-OH. The non-proteinogenic amino acid Orn-OH contains the same three-carbon methylene chain that is observed in the proteinogenic amino acids Arg-OH and Pro-OH, whereas proteinogenic Lys-OH contains a structurally unique linear four-carbon methylene chain.

Lysine (Lys, Figure 1) is a structurally, catalytically, and functionally important proteinogenic amino acid that possesses a basic sidechain amine, which is protonated and therefore cationic at neutral pH (ε-NH_2_ p*K*_aH_ 10.8).^27^ The charge on Lys’s ε-amine at physiological pH enables many essential interactions, including roles in hydrophilicity, hydrogen bonding, ion transport, cation−π interactions, and the net charge of proteins and protein surfaces, which in turn influence protein function.^28^ Lys is also often involved in post-translational modifications that then further regulate these functions, for example in histone modifications and gene expression.^29^

Lys’s positive charge promotes interactions with negatively charged biopolymers, such as DNA and RNA,^30^ and it has been proposed that electrostatic interactions between cationic peptides and anionic nucleic acids played a key role in abiogenesis.^31−35^ However, Lys has commonly been regarded as a “biological invention”,^36,37^ and so Lys has been widely assumed to be unavailable in the context of prebiotic chemistry. This assumption seems to contradict the obvious value and universal nature of lysine peptides in both extant life and early evolution. Both cysteine (Cys) and arginine (Arg) have similarly been assumed to be late additions to the genetic code.^36,37^ However, prebiotically plausible routes for their synthesis have recently been uncovered, suggesting that the value and availability of Lys must also be re-evaluated.^13,21^ While Lys is unique in the context of proteinogenic amino acids, it can easily be envisioned that other (simpler) diamino acids (Figure 1) can bridge the gap between availability and function during the transition from prebiotic chemistry to extant biology. Therefore, investigations into the intrinsic chemical reactivity of Lys and homologous diamino acids in water are essential to both illuminate their potential origins and to constrain which amino acid sidechains would be compatible with key steps in non-enzymatic prebiotic peptide synthesis. While Lys is incorporated into proteins by the translational machinery of cells, its homologues 2,3-diaminopropionic acid (Dpr), 2,4-diaminobutyric acid (Dab), and ornithine (Orn) are not (Figure 1). Moreover, although Orn is not a proteinogenic amino acid, it still plays an important role in biochemistry, for example it is an intermediate in the biosynthesis of both Arg and proline (Pro)^38,39^ and is a major feedstock for polyamine biosynthesis.^40^

It has been suggested that Orn may have been “proteinogenic” prior to a biological innovation^41^ but was supplanted by Arg due to an (unknown) advantage of Arg over Orn.^42^ Extant Arg aminoacyl tRNA synthetases (ArgRS) do not contain editing domains,^43^ enabling the possibility that pre-translational synthesis on ArgRS afforded Arg from Orn in an early (bio)chemical arena. However, Arg decapeptides inhibit the activity of RNA polymerase ribozymes (at suboptimal Mg^2+^ concentrations), whereas Orn decapeptides boost ribozyme activity,^32^ suggesting that a peptide–ribozyme interaction on its own would not necessarily have led to Arg displacing Orn from a primitive genetic code.

Previous suggestions for the exclusion of Orn and Dab from the genetic code have centered on the proposed lactamization of their respective aminoacylated-tRNAs,^24,44−47^ but it is unlikely that this mechanism would exclude Dpr from the genetic code. The point of prohibition of amino acid sidechains from life’s peptides may have preceded or been orthogonal to aminoacyl-(t)RNAs. The most direct point at which chemical discrimination between amino acid residues can be achieved would seemingly be during either their chemical synthesis or the formation of peptide bonds, and thus direct discrimination during peptide synthesis warrants chemical evaluation. However, none of the suggestions for differentiation of Orn and Lys have been demonstrated to deliver a selective non-enzymatic (protecting-group-free) synthesis of Lys peptides. We specifically envisaged that the reported aqueous lactamization of Orn and Dab^47,48^ could be used to discriminate between proteinogenic and non-proteinogenic sidechains during peptide bond formation in water. The structural relationship between biological amino acids Pro, Arg, and Orn, but dissimilarity of biological amino acid Lys, suggested to us that the selection of Lys must be based upon an underlying chemical differentiation of its homologues during peptide synthesis rather than during monomer synthesis (Figure 1). The unique structural disposition of Lys suggested to us that the length of its sidechain, which impedes lactamization, is chemically privileged to undergo *C*-terminal peptide synthesis at a growing peptide chain. We sought to test this hypothesis through the lens of non-enzymatic peptide synthesis in water.

We have recently shown that α-aminonitriles (AA-CN) can be exploited in a non-enzymatic, biomimetic *N*-to-*C* terminal synthesis of peptide bonds in aqueous solution.^11,13,15^ Our ligation follows the same synthetic strategy as biological peptide growth, which universally proceeds in the *N*-to-*C* terminal direction and through activation of the *C*-terminus of the growing peptide to nucleophilic addition of the incoming monomer. If this (biological) synthetic logic for peptide synthesis has endured from life’s prebiotic beginnings, it may hold the key to understanding the selection of Lys peptides. We suspected that the environmental constraints imposed upon peptide chemistry by near neutral pH aqueous conditions, coupled with (biomimetic) *N*-to-*C* peptide growth, would be a key element in lysyl sidechain selection, so we set out to further investigate prebiotic peptide ligations through *C*-terminal lysyl-peptides in water.

The reactivity of AA-CNs circumvents a myriad of problems for peptide synthesis using amino acids (AA-OH) in water.^11,13,15^ For example, the low basicity of AA-CNs (p*K*_aH_ ∼ 5.6)^49^ makes them ideally suited to be nucleophilic in neutral water, where the nucleophilicity of AA-OHs (p*K*_aH_ ∼ 9.8) is predominantly quenched by protonation.^11^ Importantly, with respect to Lys, the low p*K*_aH_ of an AA-CN provides the chemical differentiation required to directly ligate Lys aminonitrile monomers (Lys-CN) to a growing peptide chain (α/ε > 78:1 at pH 7.0), and therefore protecting group-free Lys peptide ligation in water.^11^

In addition to its effect on α-selectivity, the nitrile moiety delivers the thermodynamic activation required (to the *C*-terminus of the growing peptide chain) to drive further *N*-to-*C* (biomimetic) peptide growth. The iterative ligation of peptidyl thioacids and AA-CNs generates polypeptides and can be achieved over a broad pH range with mild prebiotic activating agents, such as potassium ferricyanide (K_3_Fe(CN)_6_), Cu^2+^, or cyanoacetylene.^11^ Operating within this ligation cycle and in water at near-neutral pH, we sought to test whether *C*-terminal ligation of α-thioacids of Lys (e.g., Ac-Lys-SH), and its homologues, to aminonitriles would provide the selection, via lactamization, required to exclude the non-proteinogenic homologues of Lys from peptide coupling through their carbonyl moiety at neutral pH in water.

## Results and Discussion

### Selective Incorporation of *C*-Terminal Lys over Orn Residues

**Figure 2:**
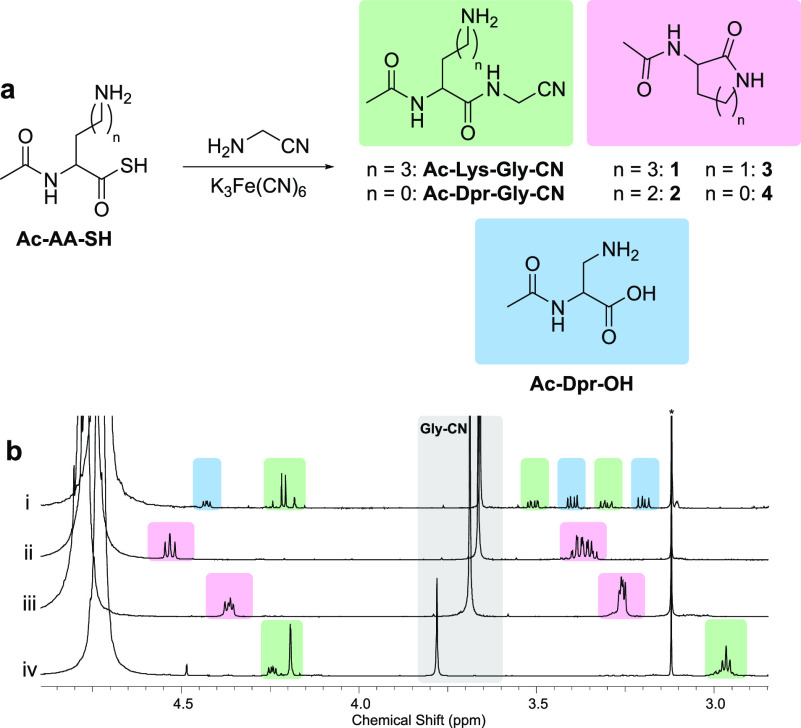
Selective *N*-to-*C* terminal ligation of lysine peptides in water. (a) The reaction of Ac-AA-SH with Gly-CN in neutral water yields ligation (green), hydrolysis (blue), or cyclization (pink). (b) ^1^H nuclear magnetic resonance (NMR; 700 MHz, D_2_O, 25 °C) spectra showing the reaction of Ac-AA-SH (60 mM) with Gly-CN (2 equiv) and K_3_Fe(CN)_6_ (3 equiv) at neutral pH after 30 min: (i) Ac-Dpr-SH; (ii) Ac-Dab-SH; (iii) Ac-Orn-SH; (iv) Ac-Lys-SH. *Methylsulfonylmethane (internal standard).

To demonstrate the efficacy of lysyl-peptide nitrile synthesis in water, Ac-Lys-SH (60 mM) was ligated with Gly-CN (2 equiv) and K_3_Fe(CN)_6_ (3 equiv). At pH 7, near-quantitative formation of dipeptide Ac-Lys-Gly-CN (96%) was observed (Figure 2; Table 1, entry 1). Good yields were also achieved with the more sterically encumbered AA-CNs, Ala-CN (70%), and Val-CN (60%, Supplementary Figures 4–7). Intermolecular AA-CN ligation outcompetes intramolecular cyclization of Ac-Lys-SH at near neutral pH (pH 5.0–7.0); however, at elevated pH (pH 8.0–10), cyclization to lactam **1** begins to dominate (Supplementary Figure 3).^11^ If unbuffered, the reaction of Ac-Lys-SH with AA-CN and K_3_Fe(CN)_6_ is observed to result in a concomitant decrease in the solution pH (by ∼2 pH units at 60 mM initial [thioacid]) as the reaction proceeds to completion. If the reaction is buffered (e.g., phosphate buffer) at pH 6.0–7.0, an equal ligation yield is observed to unbuffered reactions that are initiated between pH 6.0 and 9.0 without undergoing a (significant) change in solution pH. For example, the reaction of Ac-Lys-SH (60 mM) with AA-CN (2 equiv) and K_3_Fe(CN)_6_ (3 equiv) in phosphate buffer (600 mM) is observed to yield 93% Ac-Lys-Gly-CN (Table 1, entry 2). It is of note that in water the low basicity of AA-CN enables these couplings to occur at neutral or even at acidic pH, where lactamization is suppressed by protonation of Lys’s ε-NH_2_.

**Table 1:**
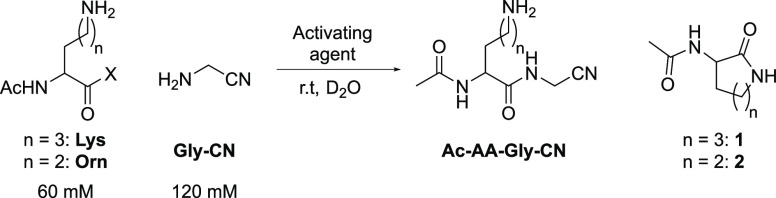
Selective Coupling of Lysine with Glycine Nitrile to Yield Peptide Nitriles^a^

entry	substrate	buffer (600 mM)	time/h	pD_i_	pD_f_	activating agent	Ac-AA-Gly-CN %	lactam %
1	Ac-Lys-SH		0.5	7.5	3.5	K_3_Fe(CN)_6_	96	<1
2	Ac-Lys-SH	PB	0.5	7.0	6.8	K_3_Fe(CN)_6_	93	5
3	Ac-Lys-OH		48	7.0	9.1	EDC	13	56
4	Ac-Lys-OH	PB	1	7.0	7.0	EDC	13	1
5	Ac-Orn-SH		0.5	7.5	5.4	K_3_Fe(CN)_6_	n.d.	95
6	Ac-Orn-SH	PB	0.5	7.0	6.7	K_3_Fe(CN)_6_	n.d.	92
7	Ac-Orn-OH		48	7.0	8.9	EDC	1	73
8	Ac-Orn-OH	PB	1	7.0	7.0	EDC	n.d.	14

a Activating agent = EDC (120 mM) or K_3_Fe(CN)_6_ (180 mM). pD_i_ = initial pD; pD_f_ = final pD. PB = phosphate buffer.

**Figure 3:**
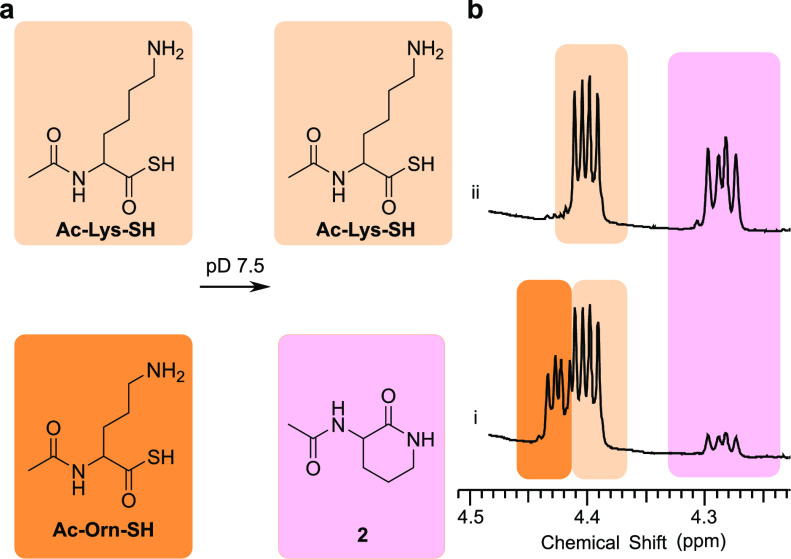
Selective cyclization of Orn thioacid. The *C*-terminal Lys thioacid residue is observed to be highly stable relative to the *C*-terminal Orn thioacid residue. (a) Incubation of a stoichiometric mixture of Ac-Lys-SH (60 mM) and Ac-Orn-SH (60 mM) in D_2_O at pD 7.5 was observed to selectively cyclize Ac-Orn-SH to yield lactam **2** in >90% yield after 3 days, while Ac-Lys-SH was unmodified. (b) ^1^H NMR (700 MHz, D_2_O, 25 °C) spectra of Ac-Lys-SH (60 mM) and Ac-Orn-SH (60 mM) in D_2_O at pD 7.5 after (i) 3 h and (ii) 3 days. For clarity, only Lys and Orn α-CH resonances are shown; full spectra and their assignment are reported in Supplementary Figure 38.

We next tested whether the non-proteinogenic Orn residue, which contains an equally basic δ-NH_2_ (p*K*_aH_ 10.8),^50^ would display the same ligation profile as Lys. We began by incubating Ac-Orn-SH (60 mM) with Gly-CN (2 equiv) in water at pH 7. When K_3_Fe(CN)_6_ (3 equiv) was added to activate the thioacid, near-quantitative cyclization to lactam **2** (95%) was observed (Figure 2; Table 1, entry 5). Peptide ligation through the *C*-terminal Orn residue was not detected. Therefore, at neutral pH, while Lys peptides can grow via *N*-to-*C* terminal ligation, Orn peptides cannot—indicating that the sidechain basicity and length of Lys are both essential for effective *N*-to-*C* terminal ligation. Indeed, upon incubating Ac-Lys-SH (60 mM) and Ac-Orn-SH (1 equiv) in neutral water with Gly-CN (2 equiv) and K_3_Fe(CN)_6_ (6 equiv), Ac-Lys-Gly-CN (>95%) and lactam **2** (93%) were observed as the major products (Supplementary Figure 40). Furthermore, when a mixture of Ac-Lys-SH and Ac-Orn-SH (1:1) were incubated in neutral water at room temperature, selective conversion of Ac-Orn-SH to lactam **2** (>90%) was observed over 3 days, while remarkably 93% Ac-Lys-SH was returned (Figure 3). Given the similar p*K*_aH_ of Lys and Orn sidechain amines, this switch in reactivity must be attributed to the length of the sidechain. Together, these experiments demonstrate for the first time an absolute and direct nonenzymatic discrimination between Lys and Orn residues in water during peptide synthesis.^24,44,46,47^

Lactamization of Ac-Orn-SH (60 mM) cannot be suppressed even by the addition of a large excess of Gly-CN (10 equiv) at neutral pH (Supplementary Figure 11). Seeking conditions under which *C*-terminal Orn-SH residues can be coerced to ligate, we next incubated Ac-Orn-SH (60 mM), Gly-CN (2 equiv), and K_3_Fe(CN)_6_ (3 equiv) under acidic conditions (pH 5.0), where the high p*K*_aH_ Orn δ-NH_2_ would be overwhelmingly protonated and lactamization maximally suppressed. However, the major product of the reaction at pH 5.0 was still observed to be lactam **2** (67%); Ac-Orn-Gly-CN (<10%) only formed in very low yield (Supplementary Figures 14–16). Further acidification did not increase the yield of Ac-Orn-Gly-CN (Supplementary Table 2). In contrast, good to moderate yields of Ac-Lys-Gly-CN were observed even at pH 5.0 (53%) and pH 3.0 (25%) (Supplementary Table 2). These observations are testament to the matched basicity of Gly-CN (low p*K*_aH_) and Lys’s ε-NH_2_ (high p*K*_aH_), allowing peptide ligation with aminonitriles under acidic conditions. However, it is of note that we observed optimal discrimination between Lys and Orn residues at neutral pH, not under acidic conditions. At neutral pH, under our reaction conditions this selection is near-absolute, with near-quantitative Lys peptide ligation and near-quantitative Orn cyclization (Figure 2b). We are not aware of any other equally selective (non-enzymatic) discrimination between Lys and Orn residues during peptide bond formation.

To test whether the differentiation of Lys and Orn was specific to thioacid activation, we next incubated Ac-Lys-OH (60 mM) with the carboxylic acid-activating agent 1-ethyl-3-(3-dimethylaminopropyl)carbodiimide (EDC, 2 equiv) and Gly-CN (2 equiv). Unbuffered EDC ligations were observed to increase in pH (pH 7–9) as the reaction progressed and therefore resulted in extensive lactamization, yielding only 13% Ac-Lys-Gly-CN, alongside 56% lactam **1** (Table 1, entry 3; Supplementary Table 8; Supplementary Figure 51). EDC activation of Ac-Lys-OH in phosphate buffer dramatically improved the ratio of ligation/cyclization (13:1) but yielded a relatively poor (14%) total conversion (Table 1, entry 4; Supplementary Table 8; Supplementary Figure 52). To avoid phosphate-catalyzed EDC hydrolysis during peptide activation, we next investigated imidazole-, MES-, and MOPS-buffered EDC ligations. While these reactions led to improved yields of Ac-Lys-Gly-CN (up to 48%), very poor selectivity was observed; the ligation/cyclization ratio observed in imidazole (1.6:1), MES (1:1.6), and MOPS (1:1.8) was significantly (>8-fold) depressed with respect to phosphate buffer at neutral pH (Supplementary Table 8). Imidazole buffer was also observed to decrease the coupling selectivity of thioacid ligations (Supplementary Table 1), likely due to the partial formation of an acyl imidazole intermediate. On the other hand, the reaction of Ac-Lys-SH with Gly-CN/K_3_Fe(CN)_6_ in phosphate, MES, or MOPS solution furnished much higher yields (>91%) and higher ratios of ligation/cyclization (>18:1; Table 1, entry 2, and Supplementary Table 1). Because these Ac-AA-SH ligations were near-quantitative, and as EDC activation is not prebiotically plausible, we made no further attempt to optimize EDC ligations. However, we found that incubation of Ac-Orn-OH (60 mM) with EDC (2 equiv) and Gly-CN (2 equiv) led to near-absolute selectivity for lactam **2** (Table 1, entries 7–8; Supplementary Table 8; Supplementary Figure 53), as was observed during Ac-Orn-SH activation (Table 1, entries 5–6). These results suggest that the selective *C*-terminal capping of Orn peptides is not wholly dependent on the nature of activation at the *C*-terminus but is an inevitable consequence of the Orn sidechain at neutral pH. These results also underscore the efficacy, selectivity, rate, and high yield of thioacid ligations at neutral pH, even in comparison to EDC activation.

### Selective Incorporation of *C*-Terminal Lys over Dab Residues

Having successfully shown that Lys residues can be selectively incorporated into peptides over Orn residues, we shifted our focus to the other non-proteinogenic homologues of Lys, Dab, and Dpr, within the context of prebiotic AA-CN ligation. Given the rapid δ-lactamization of Orn, we suspected that Dab cyclization, to its γ-lactam **3**, would be even more facile. As anticipated, the reaction of Ac-Dab-SH (60 mM) with Gly-CN (2 equiv) and K_3_Fe(CN)_6_ (3 equiv) in neutral water led to near-quantitative formation of lactam **3** (95%) (Figure 2b; Supplementary Figure 18). This indicates that Dab and Orn can be differentiated from Lys by the same chemical mechanism during *N*-to-*C* terminal peptide ligations.

### Selective Incorporation of *C*-Terminal Lys over Dpr Residues

We next investigated Dpr. Interestingly, Ac-Dpr-SH (60 mM) ligated with Gly-CN (2 equiv) in neutral water to yield Ac-Dpr-Gly-CN (45%) in a moderate yield, alongside significant hydrolysis to Ac-Dpr-OH (37%) (Figure 2b; Supplementary Figures 21–24). Dpr, the shortest homologue of Lys, can therefore also be successfully incorporated into elongating peptides and would not have been excluded from peptide synthesis through lactamization. The ligation yield of Dpr is lower than that of Lys under comparable conditions due to competing hydrolysis. However, in the presence of excess Gly-CN (10 equiv), the yield of ligation rose to 66% (Supplementary Figure 25). Although the formation of β-lactam **4** was not observed in any Dpr-SH ligations (Supplementary Figures 27–28), small amounts of multiple β-branched by-products (<20%) were detected. Equivalent amounts of ε-branched by-products are not observed in the high-yielding Lys ligations.

Given that Dpr (Ac-Dpr-SH p*K*_aH_ 8.7) possesses a significantly less basic sidechain than Lys (Ac-Lys-SH p*K*_aH_ 10.8), we next tested the nucleophilicity of the Dpr β-NH_2_ residue and therefore whether intermolecular reactivity of Dpr would be problematic for the selective formation of α-linked peptides. When Ac-Dpr-OH (2 equiv; p*K*_aH_ 9.2) was incubated with Ac-Gly-SH (30 mM) and K_3_Fe(CN)_6_ (3 equiv) in neutral water, only small amounts (20%) of β-ligated products (Supplementary Figure 64) were observed, even with no other competing amine nucleophile. This is (3×) more reactive than Lys’s ε-NH_2_ which gave only 6% of ε-ligation under the same conditions (Supplementary Figure 66). However, under our reaction conditions (in the presence of AA-CN), β-amidation is not problematic and can be readily outcompeted by AA-CN ligation. Accordingly, Ac-Lys-SH (60 mM) was ligated with Gly-CN (2 equiv) in the presence of Ac-Dpr-OH (1 equiv) to yield 94% Ac-Lys-Gly-CN (Supplementary Figure 44). Furthermore, a competition between Ac-Lys-SH (60 mM) and Ac-Dpr-SH (60 mM) led to similar results, with good yields observed for Lys (84%) and Dpr (52%) ligation (Supplementary Figure 45; Supplementary Table 6). Therefore, while Dpr-SH ligation is lower yielding than the proteinogenic peptide thioacids,^11^ Dpr can still be incorporated in significant yields into growing peptides (∼50% ligation yield is a good yield in the broader context of general prebiotic peptide ligation^9,12,14,16^).

Incorporation of Dpr into a peptide decreases its β-NH_2_ basicity further (i.e., Ac-Dpr-Gly-SH p*K*_aH_ 8.1). Intrigued by the p*K*_aH_ difference between Lys and Dpr peptides, and the preferential incorporation of Lys into dipeptides, we next tested the ligation of Lys and Dpr peptides where the sidechain amine residues are further removed from the *C*-terminus. We also noted that, like the ε-NH_2_ of Ac-Lys-SH, the β-NH_2_ of Ac-Dpr-AA-SH would be 7-atoms from its own activated *C*-terminus. Therefore, we next explored the balance between ligation and cyclization for Ac-Dpr-Gly-SH. The addition of K_3_Fe(CN)_6_ to Ac-Dpr-Gly-SH (20 mM) at pH 9 led to significant amounts of β-amidation (60%; Supplementary Figure 31). However, alkaline conditions are problematic even for Lys. For example, the reaction of Ac-Lys-SH (20 mM) with Gly-CN (2 equiv) buffered at pD 8.5 led to just 10% ligation, with cyclization to lactam **1** occurring in 70% yield (Supplementary Table 1). Even the reaction of Ac-Lys-Gly-SH (20 mM) with Gly-CN (2 equiv) yields only modest amounts of ligation (Ac-Lys-Gly-Gly-CN, 14%), alongside large amounts of ε-amidation (66%) at pH 9 (Supplementary Figure 36; Supplementary Table 5). However, excellent yields of ligation (90%) are recovered at neutral pH (Supplementary Figures 33–35). At neutral pH, Ac-Dpr-Gly-SH (60 mM) also ligates with Gly-CN (2 equiv) to form Ac-Dpr-Gly-Gly-CN in good yield (74%; Supplementary Figure 29; Supplementary Table 4). Moreover, the one-pot reaction of Ac-Lys-SH (60 mM) and Ac-Dpr-Gly-SH (60 mM) with Gly-CN/K_3_Fe(CN)_6_ furnished both Ac-Lys-Gly-CN and Ac-Dpr-Gly-Gly-CN in up to 96 and 69% yields, respectively (Supplementary Figure 50). While Ac-Lys-SH and Ac-Dpr-Gly-SH both react significantly through their sidechains under alkaline conditions, at neutral pH both can be effectively coupled to AA-CN without considerable sidechain amidation. Cyclization of Ac-Dpr-Gly-SH, despite its low p*K*_aH_, is likely suppressed by the kinetic barrier for *cis*–*trans* amide isomerization in the dipeptide backbone, as well as by the 7-atom ring size.

### *C*-Terminal Activation and Dpr Sidechain Synthesis

We have recently shown that Lys aminonitrile (Lys-CN) exhibits unique α-selectivity in oxidative acylations with Ac-AA-SH at neutral pH. For example, poor α-selectivity was observed for the coupling of both Lys-OH (α/ε 1.2:1) and Lys-NH_2_ (α/ε 2.7:1) with Ac-Gly-SH, whereas excellent α-selectivity was observed with Lys-CN (α/ε >78:1, Figure 4a).^11^

**Figure 4:**
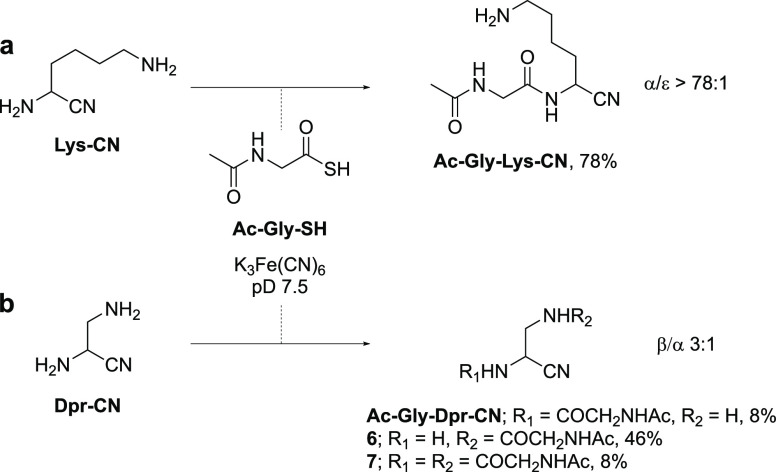
The reaction of Lys-CN is observed to be highly α-selective (>78:1) at neutral pH, due to the large p*K*_aH_ difference (Δp*K*_aH_ = 5.2) between the electronically isolated α- and ε-amines, whereas the ligation of Dpr-CN is β-selective (3:1) at neutral pH. The reaction of Ac-Gly-SH (50 mM) and K_3_Fe(CN)_6_ (3 equiv) at pD 7.5 with (a) Lys-CN (2 equiv) yields exclusively α-acylated product, and that with (b) Dpr-CN (1.5 equiv) yields a mixture of α- and β-acylated products (α/β 1:3).

**Figure 5:**
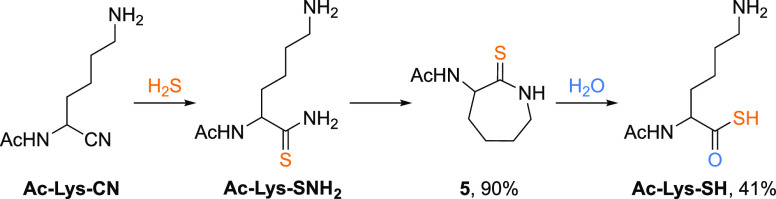
The reaction of Ac-Lys-CN (50 mM) with H_2_S (10 equiv) at pD 9 and room temperature affords the unstable lysine thioamide, Ac-Lys-SNH_2_, which then cyclizes to yield thiolactam **5** in 90% yield after 2 days at room temperature. Hydrolysis of **5**, formed in situ from Ac-Lys-SNH_2_, to Ac-Lys-SH (41%) was observed at 60 °C and pH 9.5 after 10 days.

To test whether Lys-peptide nitriles (e.g., Ac-Lys-CN) formed in α-selective acylations can yield thioacids (e.g., Ac-Lys-SH), we incubated Ac-Lys-CN (50 mM) with H_2_S (10 equiv) at pH 9.0. Ac-Lys-CN was smoothly converted to Ac-Lys-SNH_2_, which concomitantly cyclized to thiolactam **5** (90% after 2 days at room temperature; Figure 5; Supplementary Figure 72). Importantly, sulfur is retained during cyclization; thus, in contrast to lactamization, the observed thiolactamization preserves *C*-terminal activation and the potential for peptide ligation. Heating Ac-Lys-SNH_2_ (50 mM) at 60 °C and pH 9.5 led to rapid formation of **5**, which was then observed to hydrolyze slowly to furnish Ac-Lys-SH (41% from Ac-Lys-SNH_2_ after 10 days; Figure 5; Supplementary Figure 73). In situ activation of Ac-Lys-SH (7 mM), formed from Ac-Lys-SNH_2_, with K_3_Fe(CN)_6_ (3 equiv) was coupled with Gly-CN (2 equiv) at pD 7.5 to afford Ac-Lys-Gly-CN (61% from Ac-Lys-SH, Supplementary Figure 75).

Given Lys-CN’s remarkable α-selectivity, we next tested the reactivity of Dpr-CN as a ligation partner in peptide nitrile synthesis. At neutral pH, where Lys-CN reacted with complete α-selectivity, the reaction of Dpr-CN exhibited inverted (1:3) α/β selectivity (Figure 4b; Supplementary Figure 70), predominately forming β-amide **6** (46%) with small amounts of α-amide (Ac-Gly-Dpr-CN, 8%) and α,β-bis-amide **7** (8%). We attribute the poor α-selectivity of Dpr-CN, and its marked switch in reactivity relative to its homologue Lys-CN, to a combination of Dpr’s low basicity and the unique vicinal position of the two amines (facilitating intramolecular general base catalysis), which together result in β-ligation outcompeting α-ligation for Dpr-CN. Therefore, while Lys-CN monomers can be highly selectively coupled to growing α-peptides, the installation of Dpr’s sidechain must occur after peptide synthesis.

Conceptually, Michael addition of ammonia to a dehydroalanine (Dha) moiety would install the correct β-NH_2_ framework of Dpr. Recently, we have demonstrated that serine nitrile (Ser-CN) can be readily converted to Ac-Dha-CN by thioacid activation,^13^ which necessarily removes sulfide either by oxidation or precipitation. Reintroduction of sulfide to Dha yielded Cys. Thus, we reasoned that addition of ammonia, present in excess (e.g., 5 equiv)^21,22^ from the Strecker synthesis of aminonitriles, would yield Dpr.

**Figure 6:**
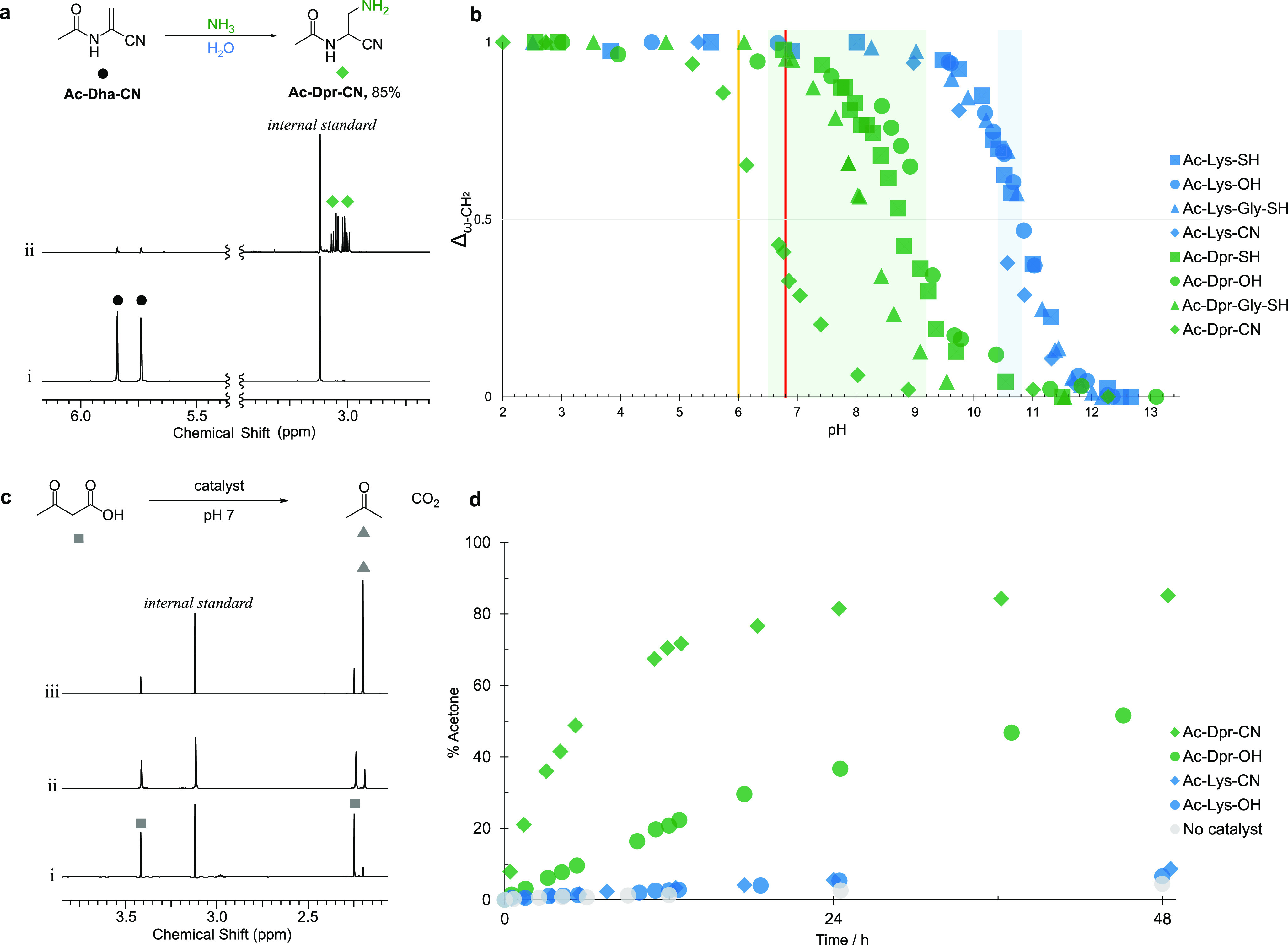
Synthesis of a p*K*_aH_-modulated Dpr-CN facilitates imine catalysis. (a) ^1^H NMR (700 MHz, 99:1 H_2_O/D_2_O, 25 °C) spectra to show (i) Ac-Dha-CN (50 mM); (ii) the reaction of Ac-Dha-CN (50 mM) and NH_3_ (10 equiv) at pH 9 after 3 h at 60 °C. Internal standard = methylsulfonylmethane. (b) Normalized ^1^H NMR ω-methylene resonance chemical shift change (Δ_ω-CH2_) observed upon pH titration of Lys (blue, ε-CH_2_NH_3_^+^ → ε-CH_2_NH_2_) and Dpr (green, β-CH_2_NH_3_^+^ → β-CH_2_NH_2_) residues. The basicity of the Dpr residue (green box, p*K*_aH_ 6.5–9.2) is heavily modulated by the peptide backbone and α-carbon, whereas the basicity of the Lys residue (blue box, p*K*_aH_ 10.4–10.8) is relatively invariant. The colored lines indicate the reported inflection points (p*K*_aH_) of the Lys residues observed in the catalytic site of acetoacetate decarboxylase (yellow line, ref (63)) and d-2-deoxyribose-5-phosphate aldolase (red line, ref (65)). (c) ^1^H NMR (700 MHz, H_2_O, 25 °C) spectra to show the decarboxylation of acetoacetate (50 mM) at pH 7 (150 mM imidazole buffer) in the presence of 10 mol % catalyst after 2 days: (i) Ac-Lys-OH, (ii) Ac-Dpr-OH, (iii) Ac-Dpr-CN. (d) ^1^H NMR yields of acetone produced by the reaction of acetoacetate (50 mM) at pH 7 (150 mM imidazole buffer) in the presence of 50 mol % catalyst.

To test this, we next monitored the reaction of Ac-Dha-CN and ammonia. At pH 9 and room temperature, we observed slow, but clean, conversion of Ac-Dha-CN to Ac-Dpr-CN. This reaction was accelerated at 60 °C, such that Ac-Dha-CN (50 mM) and ammonia (5–10 equiv) furnished Ac-Dpr-CN (81–85%) after only 3 h (Figure 6a, Supplementary Table 10). The reaction of Ac-Dha-CN (50 mM) and Ac-Dha-OH (50 mM) together led to the exclusive formation of Ac-Dpr-CN (77%; Supplementary Figure 79). No Ac-Dpr-OH was detected, highlighting the activation that the nitrile moiety relays to the Dha residue.

The α-nitrile moiety was also observed to have a profound effect on the β-sidechain amine of Dpr (i.e., Ac-Dpr-CN p*K*_aH_ 6.5; Figure 6b), and this suppressed basicity leads to excellent yields of β-amidation (85%, Supplementary Figure 68) if peptide ligation occurs while this Dpr-nitrile is present. Dpr can therefore be an excellent sidechain nucleophile in water if its p*K*_aH_ is unusually depressed by its local environment.

Importantly, the suppressed p*K*_aH_ of *C*-terminal Dpr-CN explains the selectivity of its formation and why multiple alkylations are not observed. Unlike in general alkylations (e.g., NH_3_ p*K*_aH_ 9.2 → NH_2_Et p*K*_aH_ 10.8 → NHEt_2_ p*K*_aH_ 11.1),^51^ where alkylation increases the p*K*_aH_ of ammonia, formation of Ac-Dpr-CN from ammonia substantially decreases the p*K*_aH_ and nucleophilicity of the amine product with respect to the starting amine. Dpr, with respect to other Lys homologues, is uniquely sensitive to modification by the peptide backbone and α-substitution (Figure 6b). This reactivity may be valuable in prebiotic catalysis.^52,53^

To test the application of Dpr-CN to (biomimetic) catalysis, we incubated acetoacetate (50 mM) with Ac-Dpr-CN (10–50 mol %) at pH 7 (Figure 6c; Supplementary Figures 86–88). We observed a pronounced acceleration of acetoacetate decarboxylation. It is particularly of note that, at pH 7, Ac-Dpr-CN appears to be ideally suited to promote imine catalysis. This catalytic activity was likely promoted by the low basicity of Ac-Dpr-CN (p*K*_aH_ 6.5). To test this hypothesis, we additionally investigated the effect of Ac-Lys-CN (p*K*_aH_ 10.4), Ac-Lys-OH (p*K*_aH_ 10.8), Ac-Dpr-OH (p*K*_aH_ 9.2), and Gly-CN (p*K*_aH_ 5.6) on decarboxylation (Figure 6d; Supplementary Figures 86–88). Pleasingly, Ac-Dpr-CN was the most effective catalyst at neutral pH. As a final indication of how pH and catalyst p*K*_aH_ are coupled, we observed that Gly-CN was the superior catalyst at pH 5 (Supplementary Figures 83–85).

The remarkably low p*K*_aH_ of the Dpr-CN moiety requires that onward peptide ligation of Dpr peptides occurs after the conversion of the nitrile moiety to a thioacid. However, this is in line with the outlined strategy for *N*-to-*C* terminal peptide growth by iterative aminonitrile ligation.^11^ Therefore, we next investigated the subsequent step in this process, the transformation of Dpr-CN to Dpr-SH. Ac-Dpr-CN (50 mM) was converted to its thioamide Ac-Dpr-SNH_2_ upon reaction with H_2_S (10 equiv) at pH 9.5, which spontaneously hydrolyzed to give the thioacid Ac-Dpr-SH (45%) as the major product after 21 h (Supplementary Figure 80). Notably, the hydrolysis of Ac-Dpr-SNH_2_ is more facile than proteinogenic peptide nitriles,^11^ occurring rapidly even at room temperature, likely due to the electron withdrawing effect of the β-NH_3_^+^ moiety.^48^

Taken together, our results demonstrate that a prebiotic synthesis of the simplest diamino acid (Dpr) is possible from Dha residues.^47^ Our results show that Dpr peptides can be furnished with comparable (∼0.5×) efficacy of Lys peptides. However, there remains no prebiotically plausible synthesis of Lys, while the reactivity of Dha-CN and ammonia has been demonstrated to yield Dpr. Lys residues, once available on the early Earth, however represent the optimal sidechain for monomer-by-monomer *N*-to-*C* terminal peptide ligation, as selective α-acylation of the monomer is possible, as well as *C*-terminal Lys activation.

## Conclusions

Constraining the makeup of primitive prebiotic peptides will shed light on their structure and reactivity and consequently on the functions and interactions that these peptides would enable during the early evolution of life. Specific interactions between nucleic acids (e.g., DNA and RNA) and peptides may have been essential at the origin of life.^17,18,31−35,54,55^ It is even possible that evidence for this primordial interaction is preserved today in the core of the ribosome,^56^ making the interrogation of the available prebiotic composition of (cationic) peptides an especially important undertaking. By investigating the viability of Lys homologues in aqueous peptide nitrile ligations, we have observed a pronounced differentiation of Lys from both Orn and Dab. While selective and near-quantitative ligation through *C*-terminal Lys residues is observed at near-neutral pH in water, Orn and Dab both rapidly and near-quantitatively cyclize to their respective lactams, completely blocking onward peptide synthesis. It is of note that we observed the maximum discrimination between Lys and Orn residues at neutral pH. The exclusion of Orn and Dab residues from primitive peptides via the same selection process may explain why these amino acids were not coded in extant biological protein synthesis. However, the reactivity of Dpr (Lys’s shortest homologue) is more interesting and nuanced. While we have demonstrated that Dpr can undergo ligation without cyclization, the efficacy of peptide ligation is lower than for Lys. Our results suggest that this decreased coupling efficiency is primarily due to enhanced hydrolysis at the Dpr residue, likely due to the proximal or electron-withdrawing effect of the β-NH_3_^+^ moiety. However, at neutral pH, peptides containing Dpr can still elongate via AA-CN coupling even when Dpr is adjacent to the *C*-terminus, and despite the depressed p*K*_aH_ of Dpr (compared to Lys) residues; β-amidation of Dpr peptides is not problematic at neutral pH for peptide ligation.

Together, these results demonstrate why Lys is the ideal amine residue for monomer-by-monomer *N*-to-*C* terminal peptide ligations. The Lys sidechain length is sufficient that the rapid lactamization observed for both Dab and Orn is completely suppressed at neutral pH. Additionally, the sidechain amine residue is electronically isolated from the peptide backbone (and the α-carbon), such that the Lys residue has the highest possible primary amine p*K*_aH_.^51^ This high p*K*_aH_ is essential to ensure maximal protonation, and isolation of the charged sidechain amine is necessary to prevent hydrolysis at the *C*-terminus. Therefore, for the formation of an α-peptide, Lys is superior to Dpr due to both a higher degree of sidechain protonation at neutral pH and the greater distance of the sidechain amine from the activated *C*-terminus. Moreover, while Orn is equally protonated, with respect to Lys, its shorter sidechain makes it incompatible with *N*-to-*C* terminal ligation at neutral pH. Whether or not Lys was recruited to biology early or late, these factors would seem to chemically predispose the selection of Lys over its shorter homologues in water.

The constitutional simplicity of Dpr’s sidechain compared to that of Lys has prompted speculation that Dpr was used as an early amino acid sidechain (before Lys).^57,58^ We have now demonstrated that Dpr is not only constitutionally simpler than Lys, but it is also generationally simpler when considering Dpr synthesis starting from nitrile chemistry. Michael addition of ammonia to Dha nitriles furnishes catalytically active Dpr-CN in high yields. Facile conversion of a *C*-terminal Dpr nitrile to its respective Dpr thioacid by sulfide in water is then promoted by the electron withdrawing properties of the Dpr sidechain. Dha residues are a key node in extant biology for the synthesis of peptide and amino acid sidechains, including both proteinogenic amino acids (e.g., Cys, tryptophan, selenocysteine) and non-proteinogenic residues (e.g., Dpr, lanthionine) in both non-ribosomal peptide synthesis and ribosomally synthesized and post-translationally modified peptides.^59−62^ Similarly, Dha may be a key prebiotic node for the synthesis of amino acid sidechains,^13^ enabling sidechain installation and diversification following peptide synthesis, rather than at the monomer level prior to peptide synthesis. For the synthesis of α-Dpr peptides, this strategy of sidechain synthesis (following peptide formation) is mandated by the poorly α-selective acylation of its monomers.

These results lead us to tentatively conclude that Dpr would have been a component of prebiotic peptides (formed through secondary modification as a part of the serine family of amino acids) and then Dpr would subsequently have been supplanted by Lys. The proximity of Dpr’s sidechain to the peptide backbone enables its p*K*_aH_, and therefore its charge and reactivity, to be readily modified in short and unstructured peptides (Figure 6b). This, for example, can facilitate imine catalysis (Figure 6c,d). The inherent catalytic activity of Dpr-CN peptides, at neutral pH, may have been a key element in (bio)catalysis prior to the emergence of highly ordered microenvironments in proteins where the p*K*_aH_ of Lys can be heavily suppressed.^63−67^ It is particularly of note, in the prebiotic context, that this catalytic activity can be accessed even in the shortest possible Dpr-CN (i.e., Ac-Dpr-CN). Accordingly, prebiotic Dpr catalysis warrants further investigation. How, why, and when Dpr would be excluded from proteinogenic peptides remains an open question. However, at the emergence of monomeric coding of α-polypeptide biosynthesis, a preformed Dpr monomer would likely be detrimental due to its β-reactivity.^68^ Translational Lys synthesis may have therefore provided a (general) advantage over post-translational Dpr synthesis via secondary modification of translationally coded Ser. Alternatively, the prebiotic synthesis of Dpr may support a later appearance of Lys in biology as a result of other selection filters, such as the benefit of turning on/off Lys catalysis in higher-order catalysts or the greater helix-forming propensity of Lys over Dpr.^69^ The remarkable reactivity of Lys-CN and the chemical efficacy of Lys-SH in peptide ligations at neutral pH, and its inherent differentiation from Orn-SH in peptide synthesis, mandates further investigation of prebiotic diaminonitrile synthesis. However, there is currently no known prebiotically plausible synthesis of Lys,^21,36,37^ whereas Dpr is accessible through sidechain modification of Ser-CN peptides. Therefore, an in-depth evaluation of the structure and function of primitive Dpr peptides is required.

## Data Availability

Experimental procedures and spectroscopic data are available at http://pubs.acs.org.

## Supporting Information Available

General experimental details; prebiotic couplings of Ac-(AA)_n_-SH and AA-CN; competition experiments between Ac-AA^1^-SH, Ac-AA^2^-SH, and Gly-CN; ligations of Ac-AA-OH and Gly-CN using EDC·HCl; pH titrations of amines; prebiotic acylation of amines with Ac-Gly-SH; prebiotic synthesis of Ac-Lys-SH from Ac-Lys-CN; prebiotic synthesis of Ac-Dpr-SH from Ac-Dha-CN; amine-catalyzed decarboxylation of acetoacetate; and preparative syntheses (PDF)

## References

[ref1] Crick F. H. C.; Barnett L.; Brenner S.; Watts-Tobin R. J. General Nature of the Genetic Code for Proteins. Nature 1961, 192, 1227–1232. 10.1038/1921227a0. 13882203

[ref2] Nirenberg M. W.; Matthaei J. H. The dependence of cell-free protein synthesis in *E. coli* upon naturally occurring or synthetic polyribonucleotide s. Proc. Natl. Acad. Sci. U. S. A. 1961, 47, 1588–1602. 10.1073/pnas.47.10.1588. 14479932 PMC223178

[ref3] Crick F. H. C. The Origin of the Genetic Code. J. Mol. Biol. 1968, 38, 367–379. 10.1016/0022-2836(68)90392-6. 4887876

[ref4] Woese C. R.; Dugre D. H.; Saxinger W. C.; Dugre S. A. The Molecular Basis of the Genetic Code. Proc. Natl. Acad. Sci. U. S. A. 1966, 55, 966–974. 10.1073/pnas.55.4.966. 5219702 PMC224258

[ref5] Miller S. L. A Production of Amino Acids Under Possible Primitive Earth Conditions. Science 1953, 117, 528–529. 10.1126/science.117.3046.528. 13056598

[ref6] Harada K.; Fox S. W. Thermal synthesis of natural amino-acids from a postulated primitive terrestrial atmosphere. Nature 1964, 201, 335–336. 10.1038/201335a0. 14109988

[ref7] Wolman Y.; Haverland W. J.; Miller S. L. Nonprotein amino acids from spark discharges and their comparison with the Murchison meteorite amino acids. Proc. Natl. Acad. Sci. U. S. A. 1972, 69, 809–811. 10.1073/pnas.69.4.809. 16591973 PMC426569

[ref8] Ruiz-Mirazo K.; Briones C.; de la Escosura A. Prebiotic Systems Chemistry: New Perspectives for the Origins of Life. Chem. Rev. 2014, 114, 285–366. 10.1021/cr2004844. 24171674

[ref9] Griesser H.; Bechthold M.; Tremmel P.; Kervio E.; Richert C. Amino Acid-Specific, Ribonucleotide-Promoted Peptide Formation in the Absence of Enzymes. Angew. Chem., Int. Ed. 2017, 56, 1124–1128. 10.1002/anie.201610651 28000974

[ref10] Islam S.; Powner M. W. Prebiotic Systems Chemistry: Complexity Overcoming Clutter. Chem 2017, 2, 470–501. 10.1016/j.chempr.2017.03.001.

[ref11] Canavelli P.; Islam S.; Powner M. W. Peptide Ligation by Chemoselective Aminonitrile Coupling in Water. Nature 2019, 571, 546–549. 10.1038/s41586-019-1371-4. 31292542

[ref12] Frenkel-Pinter M.; Samanta M.; Ashkenasy G.; Leman L. J. Prebiotic Peptides: Molecular Hubs in the Origin of Life. Chem. Rev. 2020, 120, 4707–4765. 10.1021/acs.chemrev.9b00664. 32101414

[ref13] Foden C.; Islam S.; Fernández-García C.; Maugeri L.; Sheppard T. D.; Powner M. W. Prebiotic Synthesis of Cysteine Peptides That Catalyze Peptide Ligation in Neutral Water. Science 2020, 370, 865–869. 10.1126/science.abd5680. 33184216

[ref14] Sauer F.; Sydow C.; Siegle A. F.; Lauer C. A.; Trapp O. From amino acid mixtures to peptides in liquid sulphur dioxide on early Earth. Nat. Commun. 2021, 12, 7182 10.1038/s41467-021-27527-7. 34893619 PMC8664857

[ref15] Singh J.; et al. Prebiotic Catalytic Peptide Ligation Yields Proteinogenic Peptides by Intramolecular Amide Catalyzed Hydrolysis Facilitating Regioselective Lysine Ligation in Neutral Water. J. Am. Chem. Soc. 2022, 144, 10151–10155. 10.1021/jacs.2c03486. 35640067 PMC9204760

[ref16] Müller F.; et al. A prebiotically plausible scenario of an RNA–peptide world. Nature 2022, 605, 279–284. 10.1038/s41586-022-04676-3. 35546190 PMC9095488

[ref17] Radakovic A.; DasGupta S.; Wright T. H.; Aitken H. R. M.; Szostak J. W. Nonenzymatic assembly of active chimeric ribozymes from aminoacylated RNA oligonucleotides. Proc. Natl. Acad. Sci. U. S. A. 2022, 119, e2116840119 10.1073/pnas.2116840119. 35140183 PMC8851484

[ref18] Roberts S. J.; Liu Z.; Sutherland J. D. Potentially Prebiotic Synthesis of Aminoacyl-RNA via a Bridging Phosphoramidate-Ester Intermediate. J. Am. Chem. Soc. 2022, 144, 4254–4259. 10.1021/jacs.2c00772. 35230111 PMC9097472

[ref19] Janzen E.; et al. Emergent properties as by-products of prebiotic evolution of aminoacylation ribozymes. Nat. Commun. 2022, 13, 3631 10.1038/s41467-022-31387-0. 35752631 PMC9233669

[ref20] Ksander G.; Bold G.; Lattmann R.; Lehmann C.; Früh T.; Xiang Y. B.; Inomata K.; Buser H. P.; Schreiber J.; Zass E.; Eschenmoser A. Chemie der α-Aminonitrile. Helv. Chim. Acta 1987, 70, 1115–1172. 10.1002/hlca.19870700424.

[ref21] Patel B. H.; Percivalle C.; Ritson D. J.; Duffy C. D.; Sutherland J. D. Common origins of RNA, protein and lipid precursors in a cyanosulfidic protometabolism. Nat. Chem. 2015, 7, 301–307. 10.1038/nchem.2202. 25803468 PMC4568310

[ref22] Islam S.; Bučar D.-K.; Powner M. W. Prebiotic selection and assembly of proteinogenic amino acids and natural nucleotides from complex mixtures. Nat. Chem. 2017, 9, 584–589. 10.1038/nchem.2703.

[ref23] Orgel L. E. Evolution of the genetic apparatus. J. Mol. Biol. 1968, 38, 381–393. 10.1016/0022-2836(68)90393-8. 5718557

[ref24] Weber A. L.; Miller S. L. Reasons for the Occurrence of the Twenty Coded Protein Amino Acids. J. Mol. Evol. 1981, 17, 273–284. 10.1007/BF01795749. 7277510

[ref25] Knight R. D.; Landweber L. F. The Early Evolution of the Genetic Code. Cell 2000, 101, 569–572. 10.1016/S0092-8674(00)80866-1. 10892641

[ref26] Ambrogelly A.; Palioura S.; Söll D. Natural expansion of the genetic code. Nat. Chem. Biol. 2007, 3, 29–35. 10.1038/nchembio847. 17173027

[ref27] Kortüm G.; Vogel W.; Andrussow A. K. Dissociation constants of organic acids in aqueous solution. Pure Appl. Chem. 1960, 1, 187–536. 10.1351/pac196001020187.

[ref28] Choudhary C.; et al. Lysine acetylation targets protein complexes and co-regulates major cellular functions. Science 2009, 325, 834–840. 10.1126/science.1175371. 19608861

[ref29] Freiman R. N.; Tjian R. Regulating the regulators: lysine modifications make their mark. Cell 2003, 112, 11–17. 10.1016/S0092-8674(02)01278-3. 12526789

[ref30] Ukmar-Godec T.; et al. Lysine/RNA-interactions drive and regulate biomolecular condensation. Nat. Commun. 2019, 10, 2909 10.1038/s41467-019-10792-y. 31266957 PMC6606616

[ref31] Kamat N. P.; Tobé S.; Hill I. T.; Szostak J. W. Electrostatic Localization of RNA to Protocell Membranes by Cationic Hydrophobic Peptides. Angew. Chem., Int. Ed. 2015, 54, 11735–11739. 10.1002/anie.201505742. PMC4600236 26223820

[ref32] Tagami S.; Attwater J.; Holliger P. Simple peptides derived from the ribosomal core potentiate RNA polymerase ribozyme function. Nat. Chem. 2017, 9, 325–332. 10.1038/nchem.2739. 28338682 PMC5458135

[ref33] Frenkel-Pinter M.; et al. Mutually stabilizing interactions between proto-peptides and RNA. Nat. Commun. 2020, 11, 3137 10.1038/s41467-020-16891-5. 32561731 PMC7305224

[ref34] Peiying Li P.; Holliger P.; Tagami S. Hydrophobic-cationic peptides modulate RNA polymerase ribozyme activity by accretion. Nat. Commun. 2022, 13, 3050. 35665749 10.1038/s41467-022-30590-3 PMC9166800

[ref35] Iglesias-Artola J. M.; et al. Charge-density reduction promotes ribozyme activity in RNA–peptide coacervates via RNA fluidization and magnesium partitioning. Nat. Chem. 2022, 14, 407–416. 10.1038/s41557-022-00890-8. 35165426 PMC8979813

[ref36] Trifonov E. N. Consensus temporal order of amino acids and evolution of the triplet code. Gene 2000, 261, 139–151. 10.1016/S0378-1119(00)00476-5. 11164045

[ref37] Wong J.; Ng S.-K.; Mat W.-K.; Hu T.; Xue H. Coevolution Theory of the Genetic Code at Age Forty: Pathway to Translation and Synthetic Life. Life 2016, 6, 12 10.3390/life6010012. 26999216 PMC4810243

[ref38] Costilow R.; Laycock L. Orn cyclase (deaminating). Purification of a protein that converts Orn to proline and definition of the optimal assay conditions. J. Biol. Chem 1971, 246, 6655–6660. 10.1016/S0021-9258(19)34165-1. 4399881

[ref39] Charlier D.; Glansdorff N. Biosynthesis of Arginine and Polyamines. EcoSal Plus. 2004, 1, 1. 10.1128/ecosalplus.3.6.1.10 26443366

[ref40] Miller-Fleming L.; Olin-Sandoval V.; Campbell K.; Ralser M. Remaining Mysteries of Molecular Biology: The Role of Polyamines in the Cell. J. Mol. Biol. 2015, 427, 3389–3406. 10.1016/j.jmb.2015.06.020. 26156863

[ref41] Jukes T. H. Arginine as an Evolutionary Intruder into Protein Synthesis. Biochem. Biophys. Res. Commun. 1973, 53, 709–714. 10.1016/0006-291X(73)90151-4. 4731949

[ref42] Longo L. M.; et al. Primordial Emergence of a Nucleic Acid-Binding Protein via Phase Separation and Statistical Ornithine-to-Arginine Conversion. Proc. Natl. Acad. Sci. U. S. A. 2020, 117, 15731–15739. 10.1073/pnas.2001989117. 32561643 PMC7355028

[ref43] Perona J. J.; Gruic-Sovulj I. Synthetic and editing mechanisms of aminoacyl-tRNA synthetases. Top. Curr. Chem. 2014, 344, 1–41. 10.1007/128_2013_456. 23852030

[ref44] Ranganathan S.; Ranganathan D.; Singh W. P. Spontaneous Cyclization of a Chain Shortened Lysine Analog. Tetrahedron Lett. 1988, 29, 3111–3114. 10.1016/0040-4039(88)85099-8.

[ref45] Bayryamov S. G.; Rangelov M. A.; Mladjova A. P.; Yomtova V.; Petkov D. D. Unambiguous Evidence for Efficient Chemical Catalysis of Adenosine Ester Aminolysis by Its 2′/3′-OH. J. Am. Chem. Soc. 2007, 129, 5790–5791. 10.1021/ja068447g. 17429968

[ref46] Hendrickson T. L.; Wood W. N.; Rathnayake U. M. Did Amino Acid Side Chain Reactivity Dictate the Composition and Timing of Aminoacyl-tRNA Synthetase Evolution?. Genes 2021, 12, 409 10.3390/genes12030409. 33809136 PMC8001834

[ref47] Bibhas H.; Prasad M.; Roy R.; Tarafdar P. K. The microenvironment and p*K*_a_ perturbation of aminoacyl-tRNA guided the selection of cationic amino acids. Org. Biomol. Chem. 2021, 19, 8049–8056. 34505850 10.1039/d1ob00798j

[ref48] Hay R.; Morris P. J. Proton ionisation constants and kinetics of base hydrolysis of some α-amino-acid esters in aqueous solution. Part III. Hydrolysis and intramolecular aminolysis of αω-diamino-acid methyl esters. J. Chem. Soc., Perkin Trans 1972, 8, 1021–1029. 10.1039/P29720001021.

[ref49] Stairs S.; et al. Divergent prebiotic synthesis of pyrimidine and 8-oxo-purine ribonucleotides. Nat. Commun. 2017, 8, 15270 10.1038/ncomms15270. 28524845 PMC5454461

[ref50] Kuo L. C.; Herzberg W.; Lipscomb W. N. Substrate specificity and protonation state of ornithine transcarbamoylase as determined by pH studies. Biochemistry 1985, 24, 4754–4761. 10.1021/bi00339a007. 3907689

[ref51] Clark J.; Perrin D. D. Prediction of the strengths of organic bases. Q. Rev., Chem. Soc. 1964, 18, 295–320. 10.1039/qr9641800295.

[ref52] Barbas C. F. III Organocatalysis Lost: Modern Chemistry, Ancient Chemistry, and an Unseen Biosynthetic Apparatus. Angew. Chem., Int. Ed. 2008, 47, 42–47. 10.1002/anie.200702210. 17943929

[ref53] Ottelé J.; Hussain A. S.; Mayer C.; Otto S. Chance emergence of catalytic activity and promiscuity in a self-replicator. Nat. Catal. 2020, 3, 547–553. 10.1038/s41929-020-0463-8.

[ref54] Söding J.; Lupas A. N. More than the sum of their parts: on the evolution of proteins from peptides. BioEssays 2003, 25, 837–846. 10.1002/bies.10321. 12938173

[ref55] Yagi S.; Padhi A. K.; Vucinic J.; Barbe S.; Schiex T.; Nakagawa R.; Simoncini D.; Zhang K. Y. J.; Tagami S. Seven amino acid types suffice to reconstruct the core fold of RNA polymerase. J. Am. Chem. Soc. 2021, 143, 15998–16006. 10.1021/jacs.1c05367. 34559526

[ref56] Hsiao C.; Mohan S.; Kalahar B. K.; Williams L. D. Peeling the Onion: Ribosomes Are Ancient Molecular Fossils. Mol. Biol. Evol. 2009, 26, 2415–2425. 10.1093/molbev/msp163. 19628620

[ref57] Hartman H. Speculations on the evolution of the genetic code IV. The evolution of the aminoacyl-tRNA synthetases. Orig. Life Evol. Biosph. 1995, 25, 265–269. 10.1007/BF01581589. 11536677

[ref58] Hartman H.; Smith T. F. The Evolution of the Ribosome and the Genetic Code. Life 2014, 4, 227–249. 10.3390/life4020227. 25370196 PMC4187167

[ref59] Sauerwald A.; et al. RNA-Dependent Cysteine Biosynthesis in Archaea. Science 2005, 307, 1969–1972. 10.1126/science.1108329. 15790858

[ref60] Chatterjee C.; Paul M.; Xie L.; van der Donk W. A. Biosynthesis and Mode of Action of Lantibiotics. Chem. Rev. 2005, 105, 633–684. 10.1021/cr030105v. 15700960

[ref61] Gaudelli N. M.; Long D. H.; Townsend C. A. β-Lactam formation by a non- ribosomal peptide synthetase during antibiotic biosynthesis. Nature 2015, 520, 383–387. 10.1038/nature14100. 25624104 PMC4401618

[ref62] Buller A. R.; van Roye P.; Murciano-Calles J.; Arnold F. H. Tryptophan Synthase Uses an Atypical Mechanism To Achieve Substrate Specificity. Biochemistry 2016, 55, 7043–7046. 10.1021/acs.biochem.6b01127. 27935677 PMC5207025

[ref63] Highbarger L. A.; Gerlt J. A.; Kenyon G. L. Mechanism of the Reaction Catalyzed by Acetoacetate Decarboxylase. Importance of Lysine 116 in Determining the p*K*_a_ of Active-Site Lysine 115. Biochemistry 1996, 35, 41–46. 10.1021/bi9518306. 8555196

[ref64] Barbas C. F. III; et al. Immune Versus Natural Selection: Antibody Aldolases with Enzymic Rates But Broader Scope. Science 1997, 278, 2085–2092. 10.1126/science.278.5346.2085. 9405338

[ref65] Heine A.; DeSantis G.; Luz J. G.; Mitchell M.; Wong C.-H.; Wilson I. A. Observation of Covalent Intermediates in an Enzyme Mechanism at Atomic Resolution. Science 2001, 294, 369–374. 10.1126/science.1063601. 11598300

[ref66] Lassila J. K.; Baker D.; Herschlag D. Origins of catalysis by computationally designed retroaldolase enzymes. Proc. Natl. Acad. Sci. U. S. A. 2010, 107, 4937–4942. 10.1073/pnas.0913638107. 20194782 PMC2841948

[ref67] Obexer R.; et al. Emergence of a catalytic tetrad during evolution of a highly active artificial aldolase. Nat. Chem. 2017, 9, 50–56. 10.1038/nchem.2596. 27995916

[ref68] Huguenin-Dezot N.; et al. Trapping biosynthetic acyl-enzyme intermediates with encoded 2,3-diaminopropionic acid. Nature 2019, 565, 112–117. 10.1038/s41586-018-0781-z. 30542153 PMC6436733

[ref69] Padmanabhan S.; York E. J.; Stewart J. M.; Baldwin R. L. Helix Propensities of Basic Amino Acids Increase with the Length of the Side-chain. J. Mol. Biol. 1996, 257, 726–734. 10.1006/jmbi.1996.0197. 8648636

