# Supplementary material for: Selective Synthesis of Lysine Peptides and the Prebiotically Plausible Synthesis of Catalytically Active Diaminopropionic Acid Peptide Nitriles in Water
Source: J Am Chem Soc. 2023 Jan 26;145(5):3121–30. doi: 10.1021/jacs.2c12497 (PMC9912261; doi:10.1021/jacs.2c12497)
Supplement: Supplementary file 1 — ja2c12497_si_001.pdf [file ja2c12497_si_001.pdf]

Supplementary Information for

Selective synthesis of lysine peptides and prebiotically plausible synthesis of catalytically active diaminopropionic acid peptide nitriles in water

Benjamin Thoma and Matthew W. Powner*

*Correspondence to: matthew.powner@ucl.ac.uk

Department of Chemistry University College London

20 Gordon Street London WC1H 0AJ, U.K

Table of Contents

General Experimental Details & Safety **5**
Prebiotic couplings of Ac-(AA)$_n$-SH and AA-CN **7**
General procedure A for the prebiotic ligation of Ac-AA-SH with AA-CN 7
General procedure B for the buffered prebiotic ligation of Ac-AA-SH and AA-CN 7
Prebiotic couplings of Ac-Lys-SH and Gly-CN 8
Prebiotic coupling of Ac-Lys-SH and Ala-CN 12
Prebiotic coupling of Ac-Lys-SH and Val-CN 14
Attempted prebiotic couplings of Ac-Orn-SH and Gly-CN 16
Attempted prebiotic coupling of Ac-Dab-SH and Gly-CN **24**
Prebiotic couplings of Ac-Dpr-SH and Gly-CN 27
Investigations into the formation and reactivity of β–lactam **4** 33
Prebiotic couplings of Ac-Dpr-Gly-SH and Gly-CN 35
Prebiotic couplings of Ac-Lys-Gly-SH and Gly-CN 39
Competition experiments between Ac-AA^1-SH, Ac-AA^2-SH and Gly-CN **44**
Stability time course of a mixture of Ac-Lys-SH and Ac-Orn-SH 44
Prebiotic coupling of Ac-Lys-SH, Ac-Orn-SH and Gly-CN 46
Prebiotic couplings of Ac-Lys-SH, Ac-Dpr-SH and Gly-CN. 48
Prebiotic couplings of Ac-Lys-SH, Ac-Dpr-Gly-SH and Gly-CN 53
Ligations of Ac-AA-OH and Gly-CN using EDC·HCl **58**
General Procedure C for EDC-mediated coupling of Ac-AA-OH and Gly-CN 58
Couplings of Ac-Lys-OH and Gly-CN with EDC·HCl 58
Coupling of Ac-Orn-OH and Gly-CN with EDC·HCl 60
Couplings of Ac-Dpr-OH and Gly-CN with EDC·HCl 61
pH titrations of amines **64**
Prebiotic acylation of amines with Ac-Gly-SH **68**
Acylation of Ac-Dpr-OH 68
Acylation of Ac-Lys-OH 70
Acylation of Ac-Dpr-CN 72
Acylation of Dpr-CN 74
Prebiotic Synthesis of Ac-Lys-SH from Ac-Lys-CN **76**
Thiolysis of Ac-Lys-CN 76
Hydrolysis of Ac-Lys-SNH$_2$ 77
Ligation of Ac-Lys-SH formed from the hydrolysis of Ac-Lys-SNH$_2$ with Gly-CN 79
Prebiotic Synthesis of Ac-Dpr-SH from Ac-Dha-CN **80**
Synthesis of Ac-Dpr-CN from Ac-Dha-CN and ammonia 80
Competition between Ac-Dha-CN, Ac-Dha-OH and ammonia 83
Thiolysis and hydrolysis of Ac-Dpr-CN to form Ac-Dpr-SH 84
Amine-catalysed decarboxylation of acetoacetate **87**

Decarboxylation of acetoacetate at pH 5 ... 87
Decarboxylation of acetoacetate at pH 7 ... 89
Decarboxylation of acetoacetate at pH 9 ... 92
Synthesis of Ac-AA(Boc)-OH ... 94
Ac-Orn(Boc)-OH ... 94
Ac-Dab(Boc)-OH ... 96
Fmoc-Dpr(Boc)-OH ... 98
Ac-Dpr(Boc)-OH ... 100
Synthesis of Ac-AA(Boc)-S^-Na^+ from Ac-AA(Boc)-OH ... 102
General procedure E: EDC-mediated 5(4*H*)-oxazolone formation. ... 102
General procedure F: Thiolysis of 5-(4*H*)-oxazolones. ... 102
5-(4*H*)-oxazolone **16** ... 103
Ac-Lys(Boc)-S^-Na^+ ... 104
5-(4*H*)-oxazolone **17** ... 106
Ac-Orn(Boc)-S^-Na^+ ... 107
Ac-Dab(Boc)-S^-Na^+ ... 109
Ac-Dpr(Boc)-S^-Na^+ ... 111
Synthesis of Ac-AA(Boc)-Gly-S^-Na^+ ... 113
Ac-Lys(Boc)-Gly-OMe ... 113
Ac-Lys(Boc)-Gly-OH ... 115
Ac-Lys(Boc)-Gly-S^-Na^+ ... 117
Ac-Dpr(Boc)-Gly-OMe ... 119
Ac-Dpr(Boc)-Gly-OH ... 121
Ac-Dpr(Boc)-Gly-S^-Na^+ ... 123
Synthesis of Ac-AA-$(Gly)_m$-SH from Ac-AA(Boc)-$(Gly)_m$-S^-Na^+ ... 125
General procedure G: Boc-deprotection of Ac-AA(Boc)-$(Gly)_m$-S^-Na^+ with TFA ... 125
Synthesis of Ac-Lys-SH ... 126
Synthesis of Ac-Orn-SH ... 128
Synthesis of Ac-Dab-SH ... 130
Synthesis of Ac-Dpr-SH ... 132
Synthesis of Ac-Lys-Gly-SH ... 134
Synthesis of Ac-Dpr-Gly-SH ... 136
Miscellaneous preparative syntheses ... 138
δ−lactam **2** ... 138
γ-lactam **3** ... 140
β−lactam **4** ... 142
Ac-Dpr-OH·HCl ... 144
N-Fluoren-9-ylmethoxycarbonylaminoacetaldehyde ... 146
Ac-Dpr(Fmoc)-CN ... 148

Ac-Dpr-CN 150
Ac-Lys(Boc)-SNH_2 152
Ac-Lys-SNH_2·HCl 154
Synthesis of Ac-Lys-CN 156
(5-Oxo-pentyl)-carbamic acid benzyl ester **20** 156
Ac-Lys(CBz)-CN 158
Ac-Lys-CN 160
Synthesis of Dpr-CN 162
Dpr-OMe·2HCl 162
Boc-Dpr(Boc)-OMe 164
Boc-Dpr(Boc)-NH_2 166
Boc-Dpr(Boc)-CN 168
Dpr-CN·2HCl 170

General Experimental Details & Safety

Nuclear magnetic resonance spectroscopy:
Proton nuclear magnetic resonance (^{1}H NMR) and carbon nuclear magnetic resonance (^{13}C NMR) were recorded on Bruker NMR spectrometers AVANCE Neo 700, AVANCE III 600, AVANCE Neo 500 and AVANCE III 400, equipped with a Bruker room temperature 5 mm multinuclear gradient probe (700 MHz), 5 mm DCH cryoprobe (600 MHz) and a gradient probe (500 and 400 MHz). ^{1}H NMR spectra were recorded at ambient temperature. Chemical shifts (δ) are reported in parts per million (ppm) and quoted to the nearest 0.01 ppm relative to the residual solvent protons in $CDCl_3$ (7.26 ppm), D_2O (4.79 ppm), DMSO-d_6 (2.50 ppm) or CD_3OD (3.31 ppm). Coupling constants (J) are quoted in Hertz (Hz). Data are presented as follows: chemical shift (multiplicity, coupling constants, integration, assignment). Coupling constants are reported to the nearest 0.1 Hz and multiplicity reported according to the following convention: s = singlet, d = doublet, t = triplet, q = quartet, m = multiplet, br = broad, app = apparent, obs. = obscured/coincidental signals and associated combinations. Diastereotopic geminal (AB) spin systems coupled to one or two additional nuclei are reported as ABX and ABXY, respectively. ^{13}C NMR spectra were recorded at ambient temperature. Chemical shifts (δ) are reported in ppm and quoted to the nearest 0.1 ppm relative to the residual solvent protons in $CDCl_3$ (77.2 ppm), DMSO-d_6 (39.5 ppm) or CD_3OD (49.0 ppm). DEPT 135 and 2-dimensional experiments (COSY, HMBC and HSQC) were used to support assignments where appropriate. Methylsulfonylmethane (MSM) was used as an internal standard for selected ^{1}H NMR experiments (characteristic signal at 3.12 ppm, s, 6H).

Infrared (IR) spectroscopy:
IR spectra were recorded on a Shimadzu IR Tracer 100 FT-IR spectrometer as solids or neat liquid through direct application. Absorbance peaks ($\tilde{\nu}$max) are reported in wavenumbers (cm^{-1}) to the nearest 1 cm^{-1}.

Mass spectrometry:
Mass spectra (MS) and high-resolution mass measurements (HRMS) were recorded on a Waters LCT Premier QTOF connected to a Waters Autosampler Manager 2777C, Thermo Finnigan MAT900, and an Agilent LC connected to an Agilent 6510 QTOF mass spectrometer at the Department of Chemistry, University College London.

Solvents:
Deionized water (H_2O) was obtained from an Elga Option 3 purification system. D_2O was purchased from Sigma Aldrich. H_2O, D_2O or H_2O/D_2O was degassed by standard freeze-pump-thaw degassing techniques. Solution pH values were measured using a Mettler Toledo Seven Compact pH meter with a Mettler Toledo InLab semi-micro pH probe, or a Corning 430 pH meter with a Fisherbrand FB68801 semi-micro pH probe. The readings for D_2O solutions are reported as pD, and corrected according to Covington et al.[1] The readings for H_2O and H_2O/D_2O (9:1 or 98:2) solutions are reported uncorrected. Tetrahydrofuran (THF), diethyl ether (Et_2O), dichloromethane (CH_2Cl_2) and all other anhydrous solvents were dried by standard methods and freshly distilled before use.

Reagents:
Reagents and solvents were obtained and used without further purification, unless specified, from the following commercial sources: Alfa Aesar, Acros Organics, Apollo Scientific, Bachem, BDH, Sigma Aldrich, Fluorochem, MerckMillipore, Fisher Scientific, VWR International, Carbosynth, Manchester Organics, Lancaster, Molekula, Honeywell, TCI and Santa Cruz Biotechnology. $NaSH\cdot xH_2O$ (Sigma-

Aldrich) was used as a source of hydrosulfide. $NaSH \cdot xH_2O$ was 50% w/w NaSH; this stoichiometry was confirmed by stoichiometric conversion and thiosulfate titration.

Chromatography:
Thin layer chromatography (TLC) was performed on Merck Silica gel 60 F_{254} plates to monitor reactions and record retention factors (R_f). Flash column chromatography was performed on a Biotage Isolera One using TELOS® brand columns.

Safety:
HCN and H_2S WARNING: Cyanide and (hydro)sulfide are highly toxic poisons by inhalation, contact, and ingestion. They generate poisonous hydrogen cyanide (pK_a = 9.2) and hydrogen sulfide (pK_a = 7.1) gas at neutral or acidic pH. Solutions containing cyanide or (hydro)sulfide, or compounds which may generate these must be handled in a well-ventilated fumehood equipped with appropriate chemical quenches, such as sodium hypochlorite (bleach) or iron (II) sulfate solution. Read and follow the material safety data sheet (MSDS) instructions for personnel handling, exposure, and disposal information. Also consult local safety personnel for regulations concerning proper disposal.

Prebiotic couplings of Ac-(AA)$_n$-SH and AA-CN

General procedure A for the prebiotic ligation of Ac-AA-SH with AA-CN

To a solution of **Ac-AA-SH** (60 mM, 1 equiv.) and **AA-CN**·HCl (2 equiv.) in degassed D_2O at the desired pD was added $K_3Fe(CN)_6$ (3 equiv.). The reaction mixture was stirred at room temperature for 30 min and then centrifuged. The solution pD was observed decrease to pD 3.5–5.5. The supernatant was analysed by 1D and 2D NMR spectroscopy. The solution pD was increase to pD 7.5 and analysed by 1D and 2D NMR spectroscopy.

General procedure B for the buffered prebiotic ligation of Ac-AA-SH and AA-CN

To a solution of **Ac-AA-SH** (60 mM, 1 equiv.) and **AA-CN**·HCl (2 equiv.) in the relevant buffer (600 mM, D_2O) was added $K_3Fe(CN)_6$ (3 equiv.). The reaction mixture was stirred at room temperature for 30 min and then centrifuged. The supernatant was analysed by 1D and 2D NMR spectroscopy.

Prebiotic couplings of Ac-Lys-SH and Gly-CN

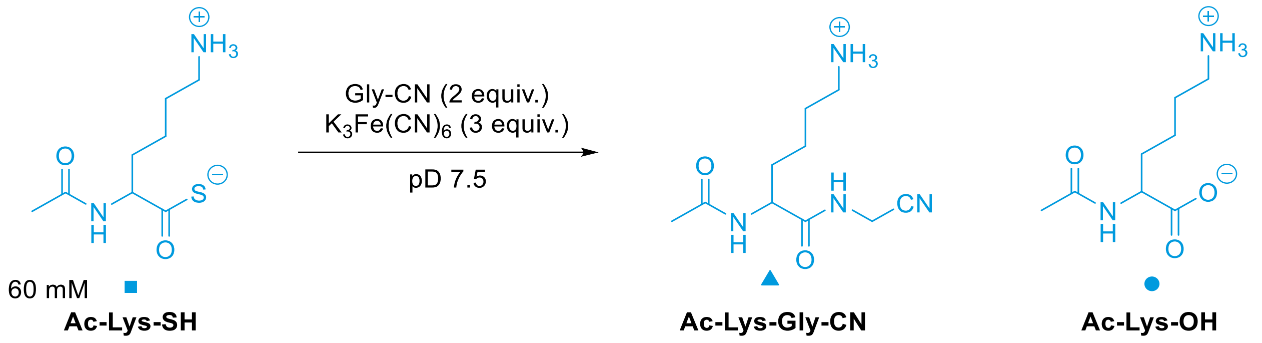

Reaction carried out *via* general procedure **A** using **Ac-Lys-SH** to afford **Ac-Lys-Gly-CN** (96%) and **Ac-Lys-OH** (< 5%).[2]

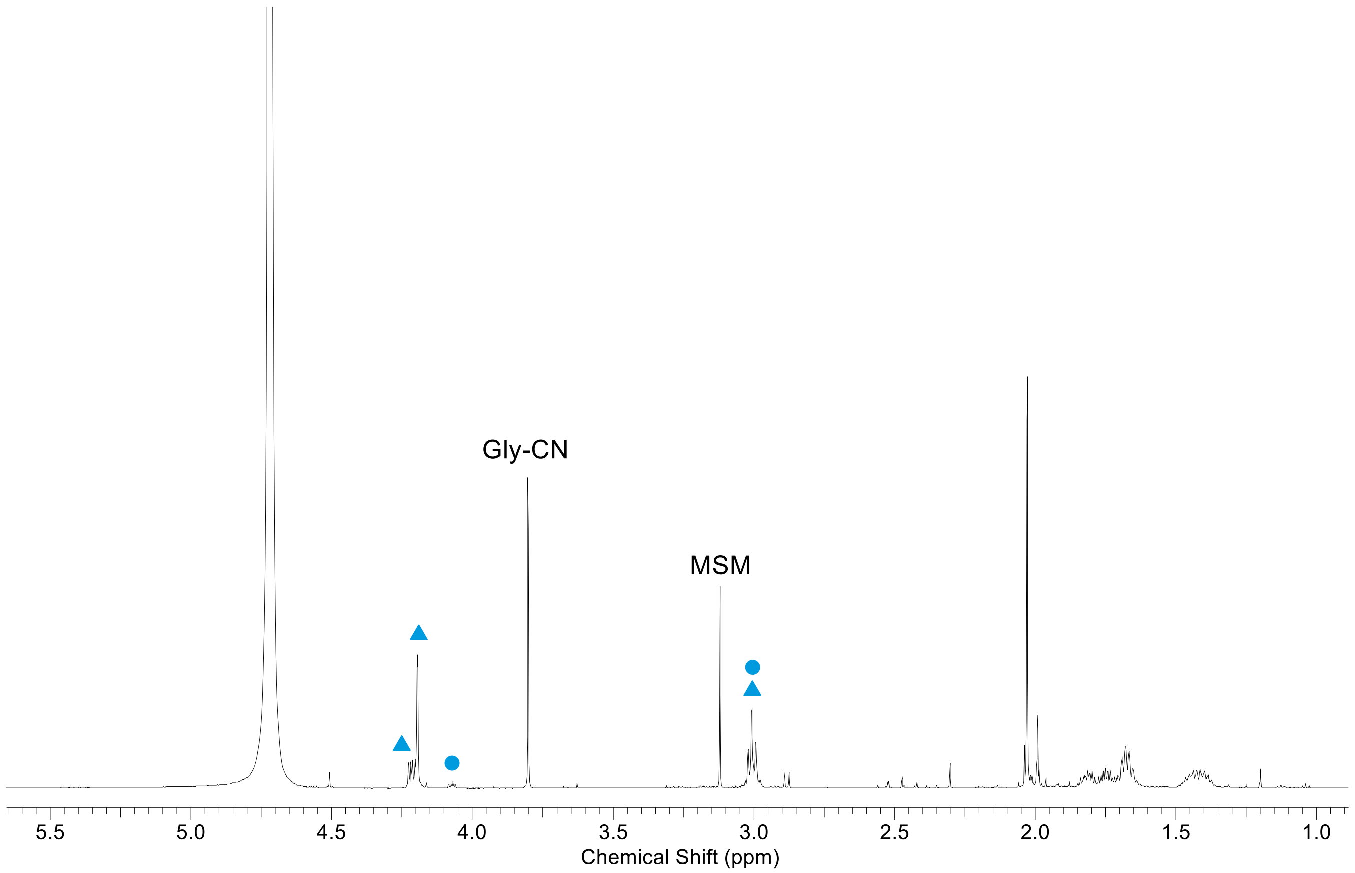

*Supplementary Figure 1. ^{1}H NMR (600 MHz, D_2O, 1.0 – 5.5 ppm) spectrum showing the products of the reaction of **Ac-Lys-SH** (60 mM) with **Gly-CN** (2.0 equiv.) and $K_3Fe(CN)_6$ (3.0 equiv.) at pD 7.5 to form **Ac-Lys-Gly-CN**.*

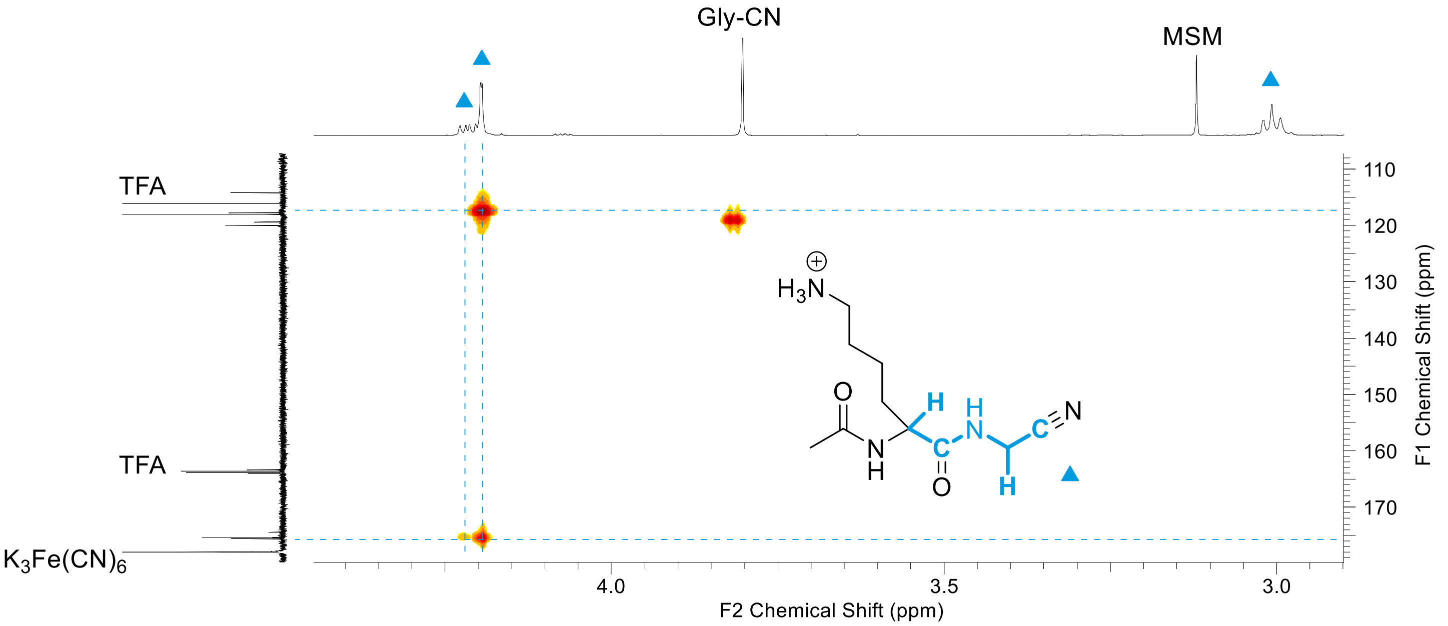

Supplementary Figure 2. ^{1}H-^{13}C HMBC (^{1}H-700 MHz [2.9–4.5 ppm], ^{13}C-176 MHz [107–179 ppm], D_2O) spectrum showing the $^2J_{CH}$ and $^3J_{CH}$ couplings of Gly-(C2)–H_2 at 4.22 ppm of ***Ac-Lys-Gly-CN*** *to C=O resonance at 175.5 ppm and CN resonance at 117.7 ppm, which is diagnostic for peptide bond formation.* ***Gly-CN*** *^{1}H resonance is misaligned with ^{13}C correlation due to fluctuation in pH between ^{1}H and HMBC acquisitions. The Gly-(C2)–H_2 ^{1}H resonance is sensitive to pH changes due to the variable protonation state of* ***Gly-CN*** *amine moiety.*[3]

Ac-Lys-Gly-CN (▲): **^{1}H NMR** (600 MHz, D_2O) δ_H 4.22 (dd, J = 8.7, 5.3 Hz, 1H, Lys-(C2)–H), 4.20 (AB, J = 17.8 Hz 1H, Gly-(C2)–H), 4.18 (AB, J = 17.8 Hz, 1H, Gly-(C2)–H′), 3.01 (t, J = 7.5 Hz, 2H, Lys-(C6)–H_2), 2.03 (s, 3H, $COCH_3$), 1.79-1.86 (m, 1H, (C3)–H), 1.62-1.76 (m, 3H, (C3)–H', (C5)–H_2), 1.34-1.45 (m, 2H, (C4)–H_2). **^{13}C NMR** (151 MHz, D_2O) δ_C 175.5 (Lys-C1), 175.2 (**C**OCH_3), 117.7 (Gly-C1), 54.5 (Lys-C2), 40.2 (Lys-C6), 30.9 (Lys-C3), 28.7 (Gly-C2), 27.0 (Lys-C5), 22.8 (CO**C**H_3/Lys-C4), 22.7 (CO**C**H_3/Lys-C4). **HRMS-ESI** $[M+H]^+$ calc. for $C_{10}H_{19}N_4O_2^+$ 227.1503; obs. 227.1503.

Ac-Lys-OH (●): **^{1}H NMR** (600 MHz, D_2O, partial assignment) δ_H 4.07 (dd, J = 8.7, 4.8 Hz, 1H, (C2)–H).

Gly-CN: **^{1}H NMR** (600 MHz, D_2O) δ_H 3.80 (s, 2H, (C2)–H_2). **^{13}C NMR** (151 MHz, D_2O) δ_C 120.5 (C1), 29.7 (C2).

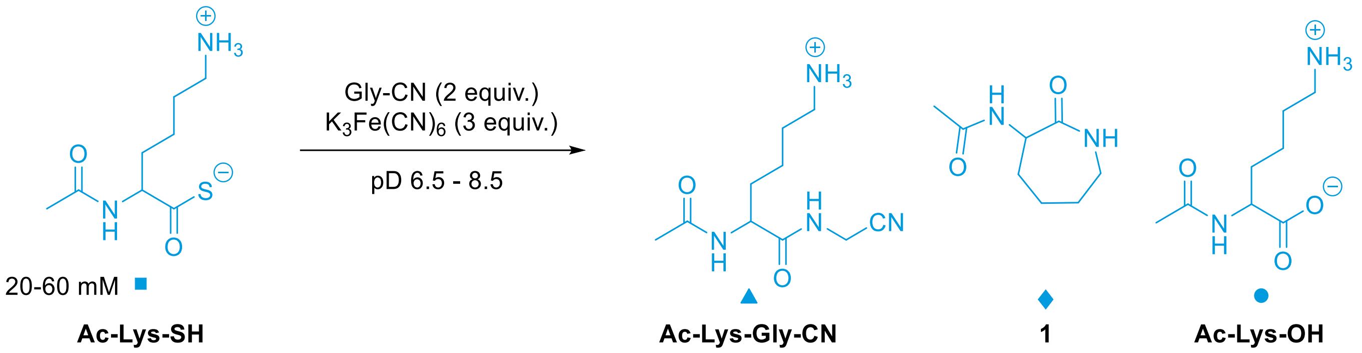

Reaction at pD 7.0 was carried out *via* general procedure **B** using **Ac-Lys-SH**.

Reaction at pD 6.5 was carried out using **Ac-Lys-SH** (60 mM), **Gly-CN** (2 equiv.) and $K_3Fe(CN)_6$ (3 equiv.) in phosphate buffer (600 mM, D_2O, pD 6.5). The reaction mixture was stirred at room temperature for 30 min and then centrifuged. The supernatant was analysed by 1D and 2D NMR spectroscopy.

Reaction at pD 8.5 was carried out using **Ac-Lys-SH** (20 mM), **Gly-CN** (2 equiv.) and $K_3Fe(CN)_6$ (3 equiv.) in borate buffer (600 mM, D_2O, pD 6.5). The reaction mixture was stirred at room temperature for 30 min and then centrifuged. The supernatant was analysed by 1D and 2D NMR spectroscopy

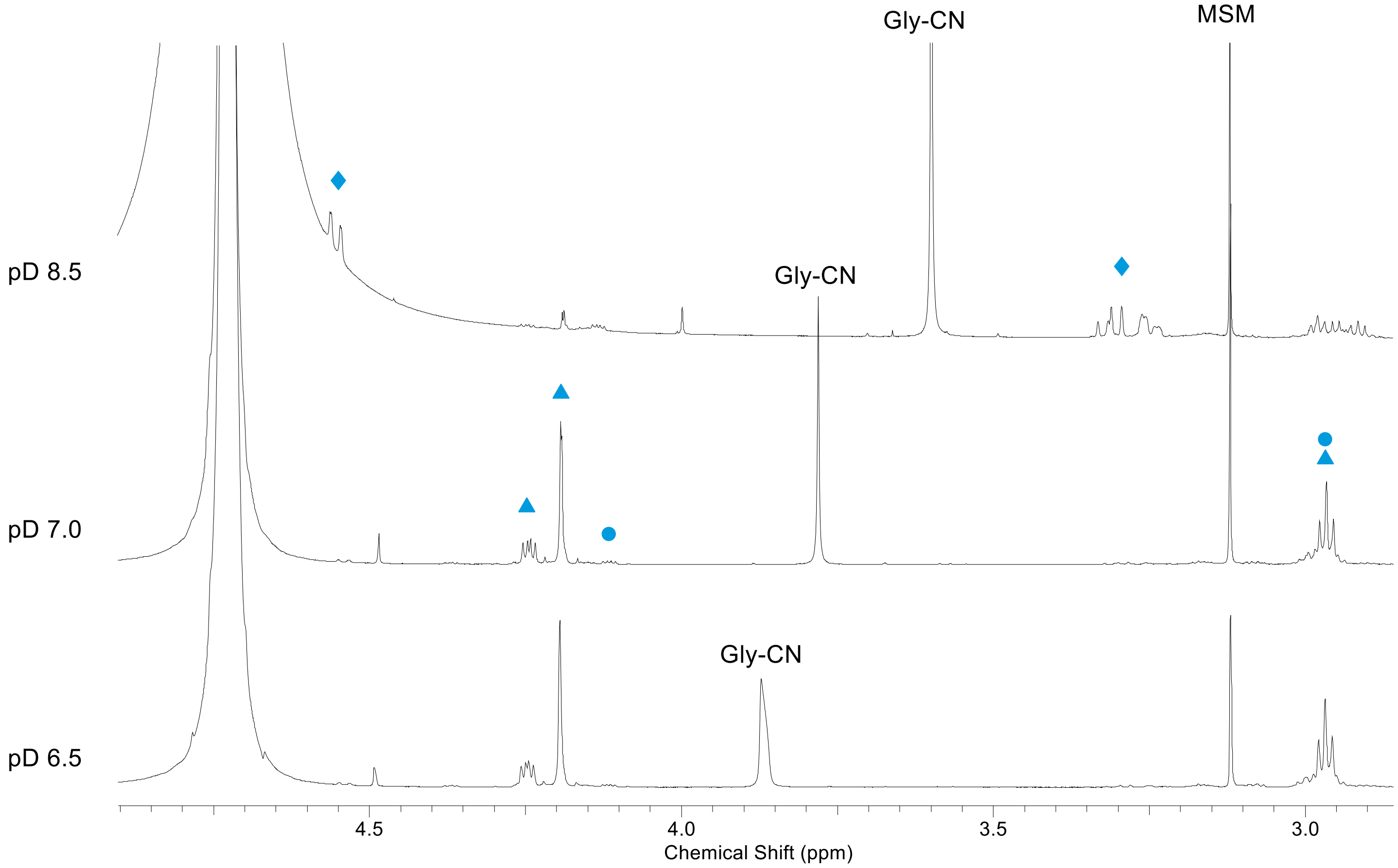

*Supplementary Figure 3. ^{1}H NMR (700 MHz, D_2O, 2.86 – 4.90 ppm) spectra to show the reaction of **Ac-Lys-SH** (60 mM at pD 6.5, 7.0; 20 mM at pD 8.5), **Gly-CN** (2 equiv.) and $K_3Fe(CN)_6$ (3 equiv.) buffered at pD 8.5 (top spectrum, 600 mM borate), pD 7.0 (middle spectrum, 600 mM phosphate) and pD 6.5 (bottom spectrum, 600 mM phosphate).*

Entry	pD	Buffer	% **Ac-Lys-Gly-CN**	% **1**	% **Ac-Lys-OH**
1	6.5	Phosphate	90	< 5	10
2	7.0	Phosphate	93	5	6
3	7.0	Imidazole	55	16	28
4[i]	7.0	MOPS	95	< 5	ND
5	7.0	MES	91	< 5	ND
6	7.5	None	96	< 1	< 5
7[ii]	8.5	Borate	10	70	20

*Supplementary Table 1. ^{1}H NMR yields for the reaction of **Ac-Lys-SH** (60 mM), **Gly-CN** (2 equiv.) and $K_3Fe(CN)_6$ (3 equiv.) at the specified pD and in the specified buffer (600 mM). [i] The pD was observed to fall to 4.8 over 30 mins. [ii] **Ac-Lys-SH** (20 mM), **Gly-CN** (2 equiv.), $K_3Fe(CN)_6$ (3 equiv.) in borate buffer (600 mM). ND = not determined due to signal overlap.*

Prebiotic coupling of Ac-Lys-SH and Ala-CN

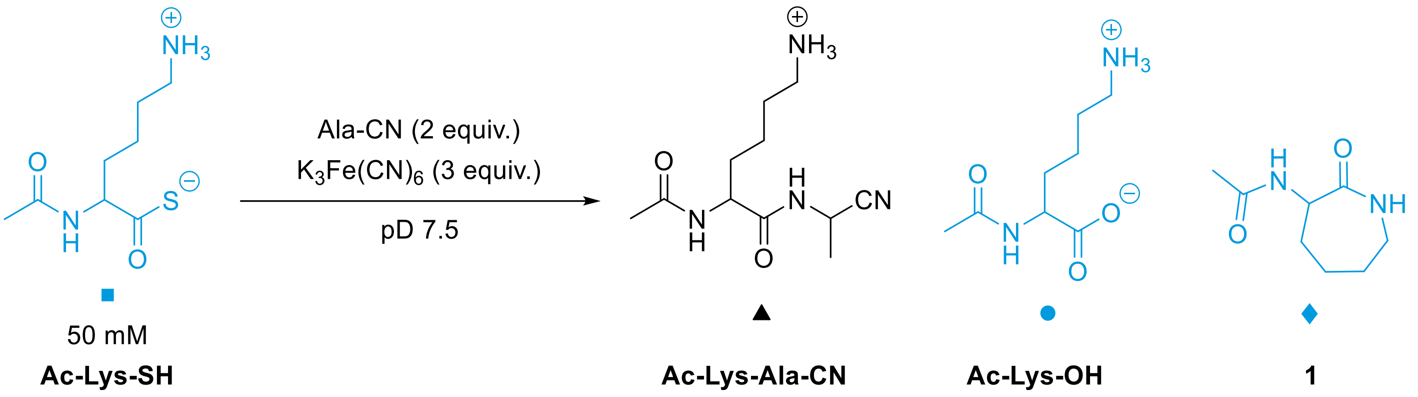

Reaction carried out using **Ac-Lys-SH** (50 mM), **Ala-CN** (2 equiv.) and $K_3Fe(CN)_6$ (3 equiv.) at pD 7.5. The reaction mixture was stirred at room temperature for 30 min and then centrifuged. The supernatant was analysed by 1D and 2D NMR spectroscopy, yielding **Ac-Lys-Ala-CN** (70%) as a mixture of diastereomers, **Ac-Lys-OH** (17%) and **1** (10%).

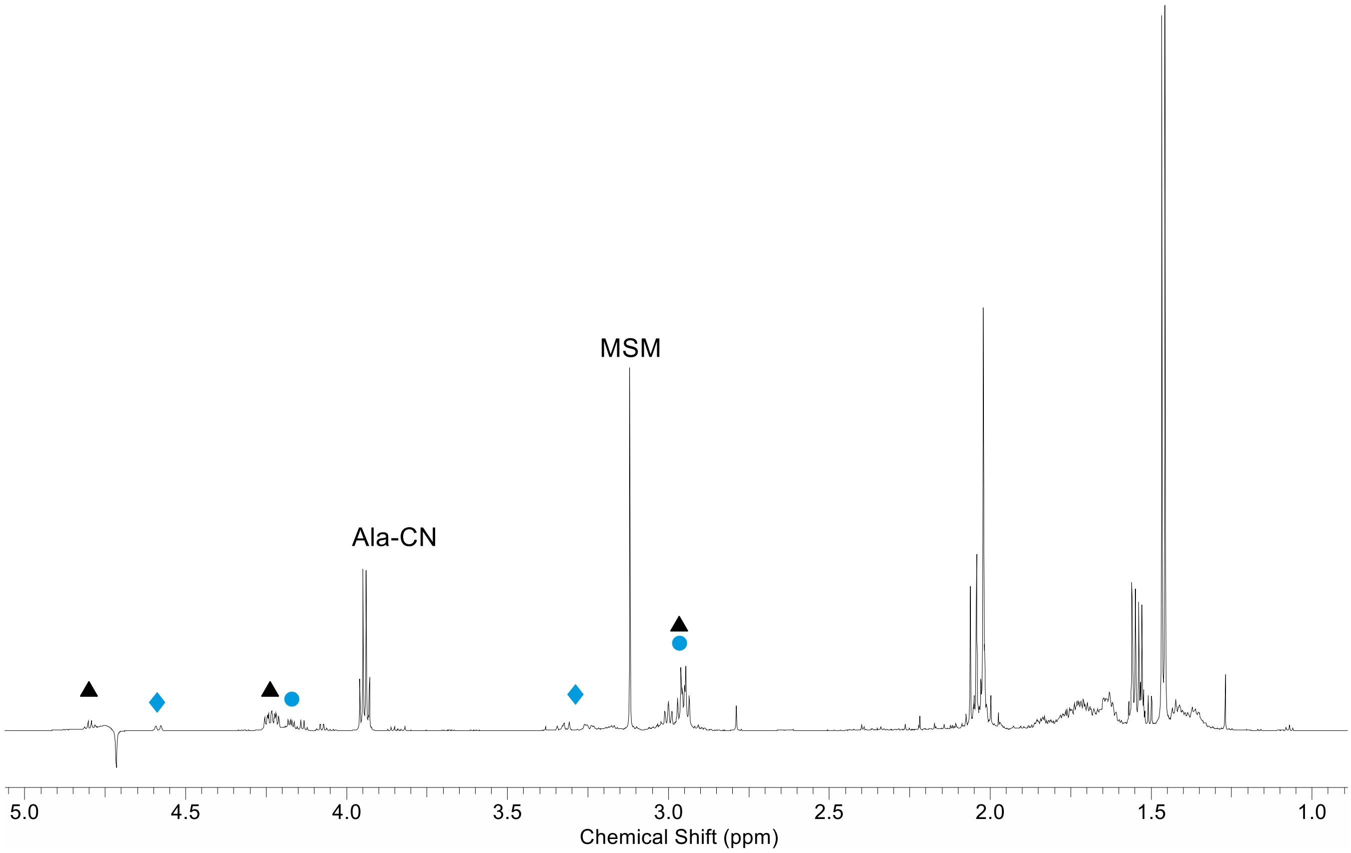

Supplementary Figure 4. ^{1}*H NMR (700 MHz,* D_2O*, noesygppr1d, 1.0 – 5.0 ppm) spectrum showing the products of the reaction of* ***Ac-Lys-SH*** *with* ***Ala-CN*** *(2 equiv.) and* $K_3Fe(CN)_6$ *(3 equiv.) to form* ***Ac-Lys-Ala-CN****.*

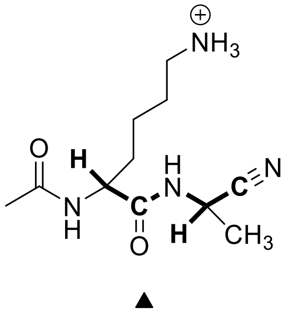

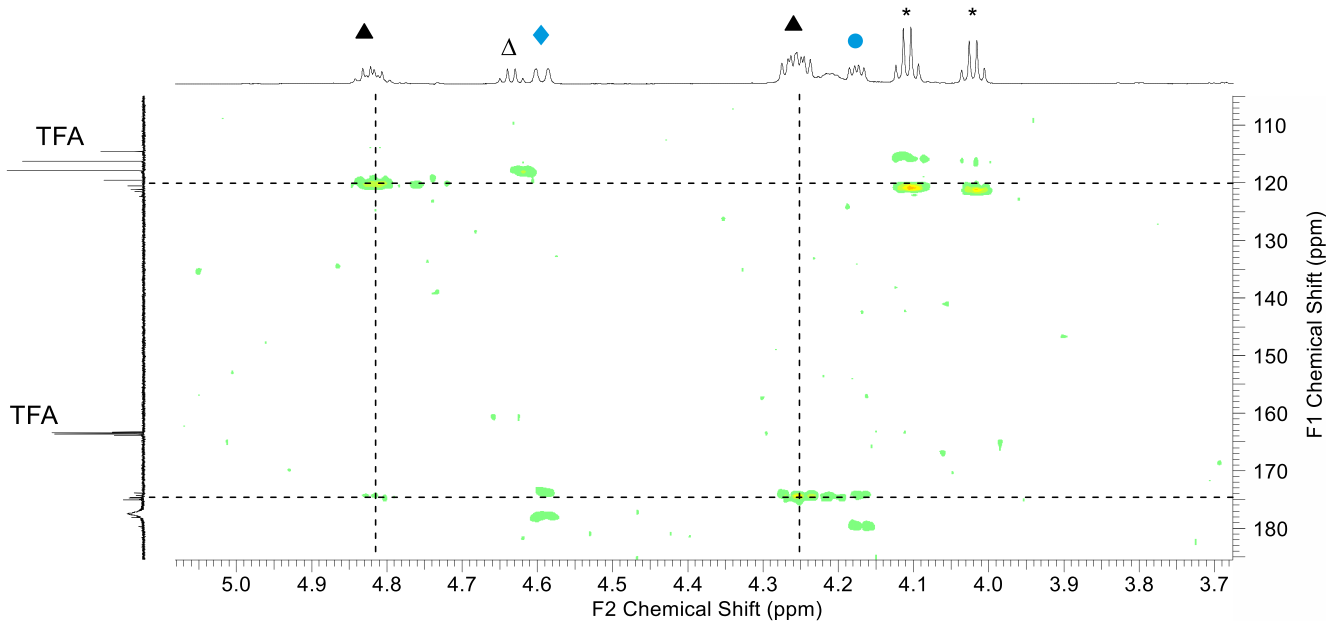

*Supplementary Figure 5. ^{1}H-^{13}C HMBC (^{1}H-700 MHz [3.7–5.1 ppm], ^{13}C-176 MHz [105–185 ppm], D_2O) spectrum showing the $^{2}J_{CH}$ and $^{3}J_{CH}$ couplings of Ala-(C2)–H at 4.83 ppm of **Ac-Lys-Ala-CN** to C=O resonance at 175 ppm and CN resonance at 120 ppm, and the $^{2}J_{CH}$ coupling of Lys-(C2)–H at 4.25 ppm of **Ac-Lys-Ala-CN** to the same C=O resonance at 175 ppm, which is diagnostic for peptide bond formation. Δ denotes lactonitrile; and * denotes 2,2'-iminodipropionitrile (two diastereomers). Both Δ and * are decomposition products of residual **Ala-CN** observed after leaving the reaction mixture for ~ 1 week at room temperature.*

Ac-Lys-Ala-CN (▲, diastereomers a/b, 1:1): **^{1}H NMR** (700 MHz, D_2O, partial assignment) δ_H 4.80 (q, *J* = 7.4 Hz, 1H, Ala-(C2)–H), 4.24 (dd, *J* = 8.5, 5.6, 1H, diastereomer a, Lys-(C2)–H), 4.22 (dd, *J* = 8.5, 5.8 Hz, 1H, diastereomer b, Lys-(C2)–H), 2.96 (t, *J* = 7.9 Hz, 2H, diastereomer a/b, Lys-(C6)–H_2), 2.95 (t, *J* = 7.6 Hz, 2H, diastereomer a/b, Lys-(C6)–H_2). **HRMS-ESI** $[M+H]^+$ calc. for $C_{11}H_{21}N_4O_2^+$ 241.1659; obs. 241.1652.

Ala-CN: **^{1}H NMR** (700 MHz, D_2O, partial assignment) δ_H 3.94 (q, *J* = 7.0 Hz, 1H, (C2)–H), 1.46 (d, *J* = 7.0 Hz, 3H, (C3)–H)

Prebiotic coupling of Ac-Lys-SH and Val-CN

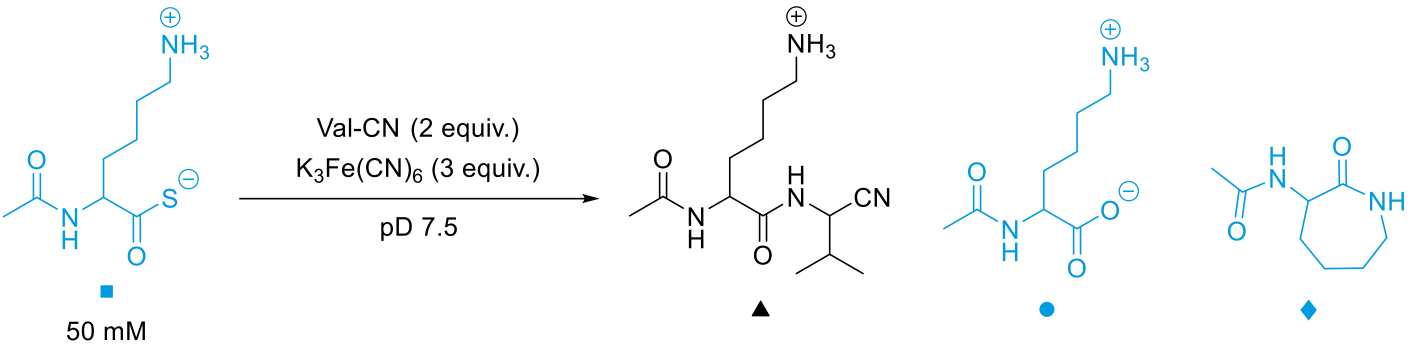

50 mM

Ac-Lys-OH **Ac-Lys-Val-CN** **Ac-Lys-OH** **1**

Reaction carried out using **Ac-Lys-SH** (50 mM), **Val-CN** (2 equiv.) and $K_3Fe(CN)_6$ (3 equiv.) at pD 7.5. The reaction mixture was stirred at room temperature for 30 min and then centrifuged. The supernatant was analysed by 1D and 2D NMR spectroscopy, yielding **Ac-Lys-Val-CN** (60%) as a mixture of diastereomers, **Ac-Lys-OH** (35%) and **1** (6%).

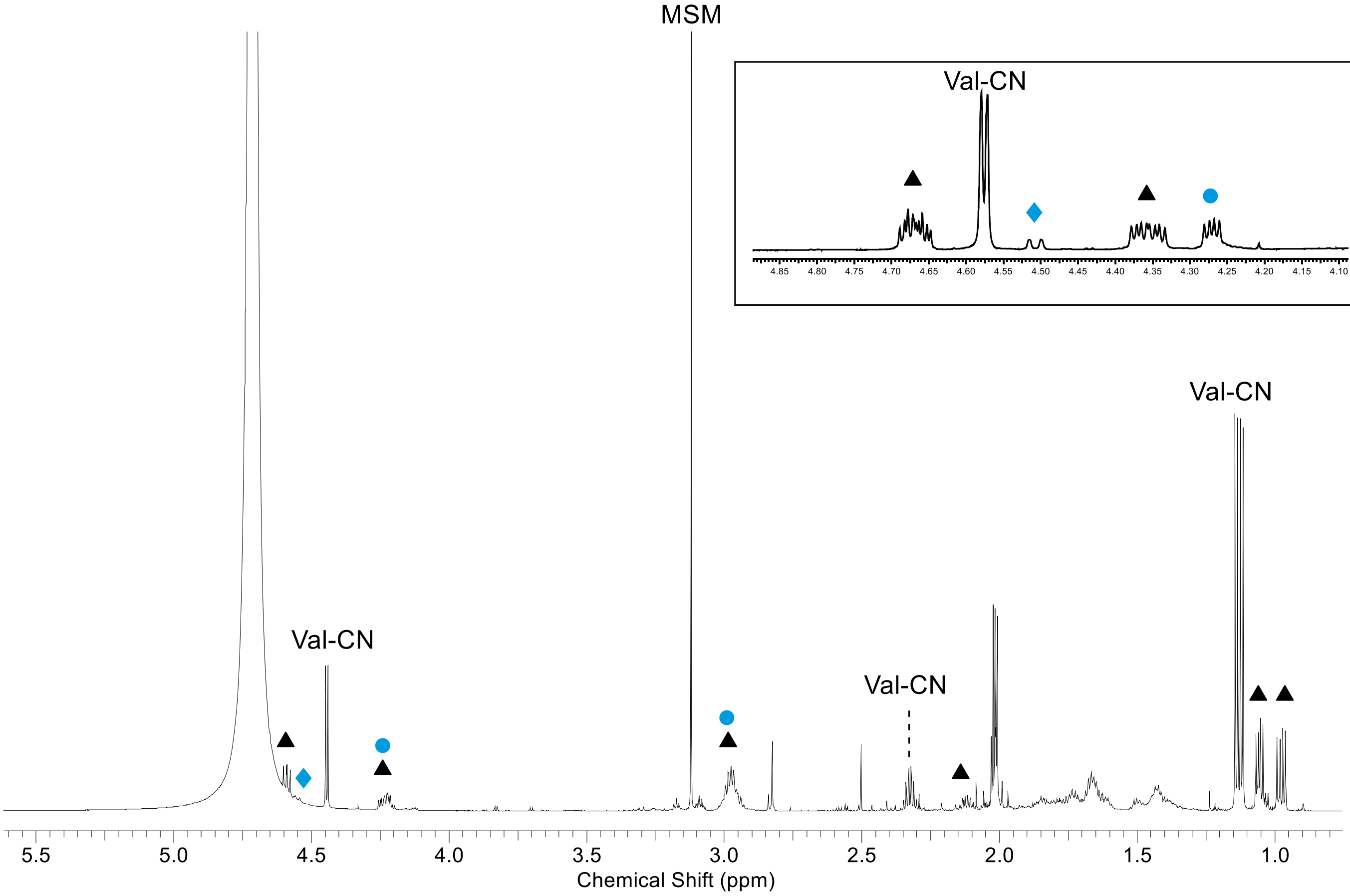

*Supplementary Figure 6. ^{1}H NMR (700 MHz, D_2O, 1.0 – 5.0 ppm) spectrum showing the products of the reaction of **Ac-Lys-SH** with **Val-CN** (2 equiv.) and $K_3Fe(CN)_6$ (3 equiv.) to form **Ac-Lys-Val-CN** as a mixture of diastereomers. Inset: ^{1}H NMR (700 MHz, 9:1 DMSO/D_2O, 4.1 – 4.9 ppm) spectrum of the reaction mixture following lyophilisation and redissolution in 9:1 DMSO/D_2O.*

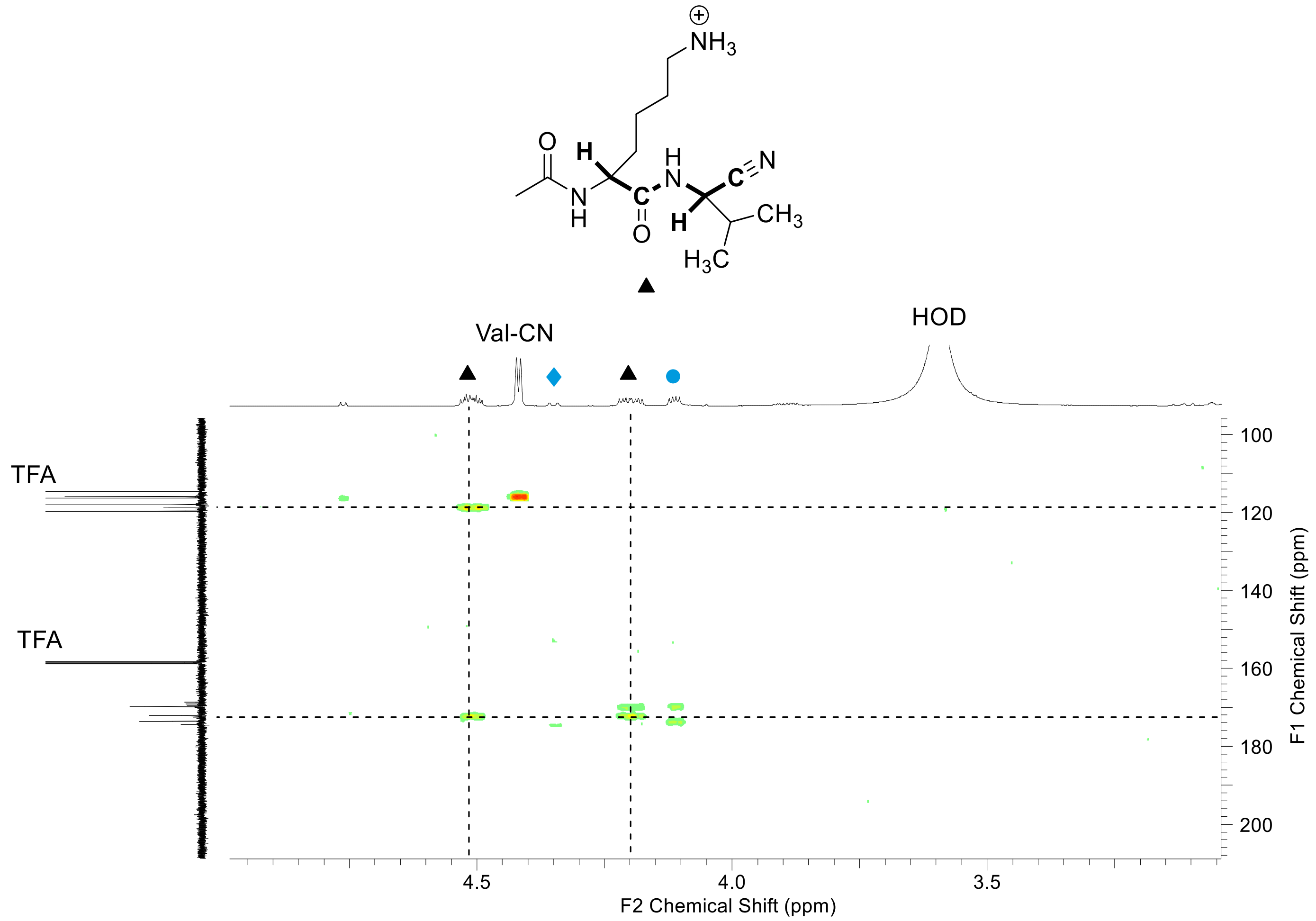

*Supplementary Figure 7. ^{1}H-^{13}C HMBC (^{1}H-700 MHz [3.1–5.0 ppm], ^{13}C-176 MHz [106–208 ppm], 9:1 DMSO/D_2O) spectrum showing the $^{2}J_{CH}$ and $^{3}J_{CH}$ couplings of Val-(C2)–H at 4.51 ppm of **Ac-Lys-Val-CN** to C=O resonance at 172 ppm and CN resonance at 119 ppm, and the $^{2}J_{CH}$ coupling of Lys-(C2)–H at 4.20 ppm of **Ac-Lys-Val-CN** to the same C=O resonance at 172 ppm, which is diagnostic for peptide bond formation.*

Ac-Lys-Val-CN (▲, diastereomers a/b, 1:1): **^{1}H NMR** (700 MHz, D_2O, partial assignment) δ_H 4.60 (d, *J* = 9.22, 1H, diastereomer a, Val-(C2)–H), 4.58 (d, *J* = 7.6 Hz, 1H, diastereomer b, Val-(C2)–H)) 1.06 (d, *J* = 6.8, 3H, diastereomer a/b, Val-(C4)–H_3), 1.05 (d, *J* = 6.8, 3H, diastereomer a/b, Val-(C4′)–H_3), 0.99 (d, *J* = 6.8 Hz, 3H, diastereomer b/a, Val-(C4)–H_3), 0.97 (d, *J* = 6.8 Hz, 3H, diastereomer b/a, Val-(C4′)–H_3). **HRMS-ESI** $[M+H]^+$ calc. for $C_{13}H_{25}N_4O_2^+$ 269.1972; obs. 269.1972.

Val-CN: ^{1}H NMR (700 MHz, D_2O, partial assignment) δ_H 4.44 (d, *J* = 5.8 Hz, 1H, (C2)–H), 1.14 (d, *J* = 6.7, 3H, (C4)–H_3), 1.12 (d, *J* = 6.8, 3H, (C4′)–H_3).

Attempted prebiotic couplings of Ac-Orn-SH and Gly-CN

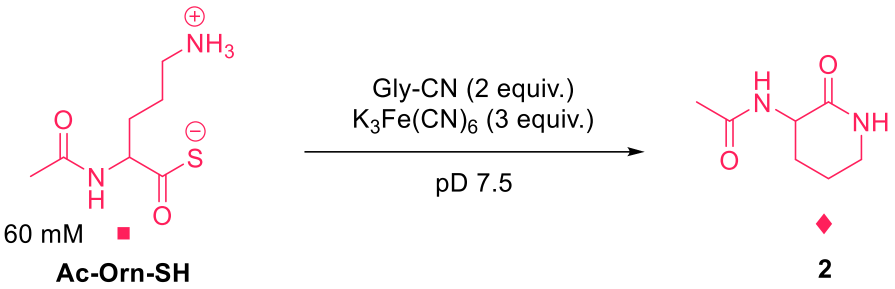

Reaction carried out *via* general procedure **A** using **Ac-Orn-SH** to afford **2** (95%).

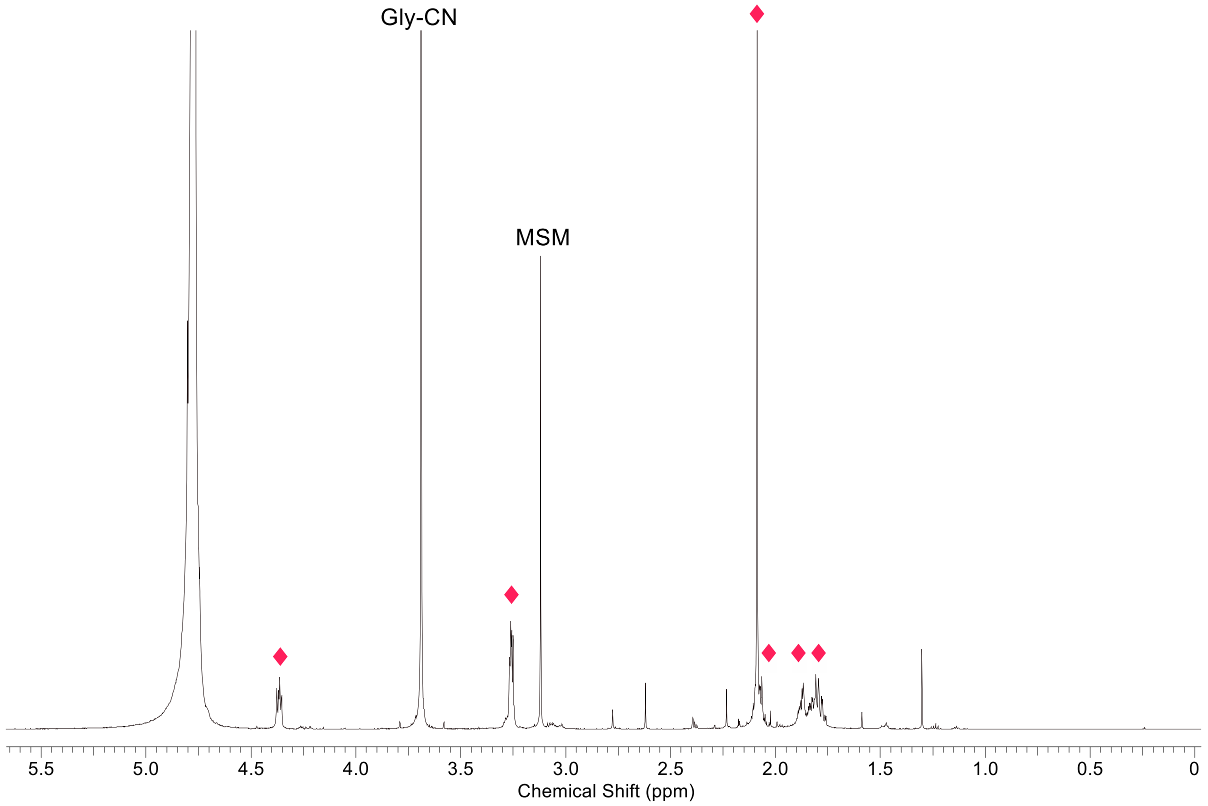

*Supplementary Figure 8. ^{1}H NMR (700 MHz, D_2O, 0.0 – 5.5 ppm) spectrum showing the products of the reaction of **Ac-Orn-SH** (60 mM) with **Gly-CN** (2 equiv.) and $K_3Fe(CN)_6$ (3 equiv.) at pD 7.5 to form **2**.*

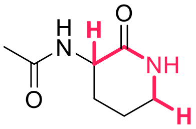

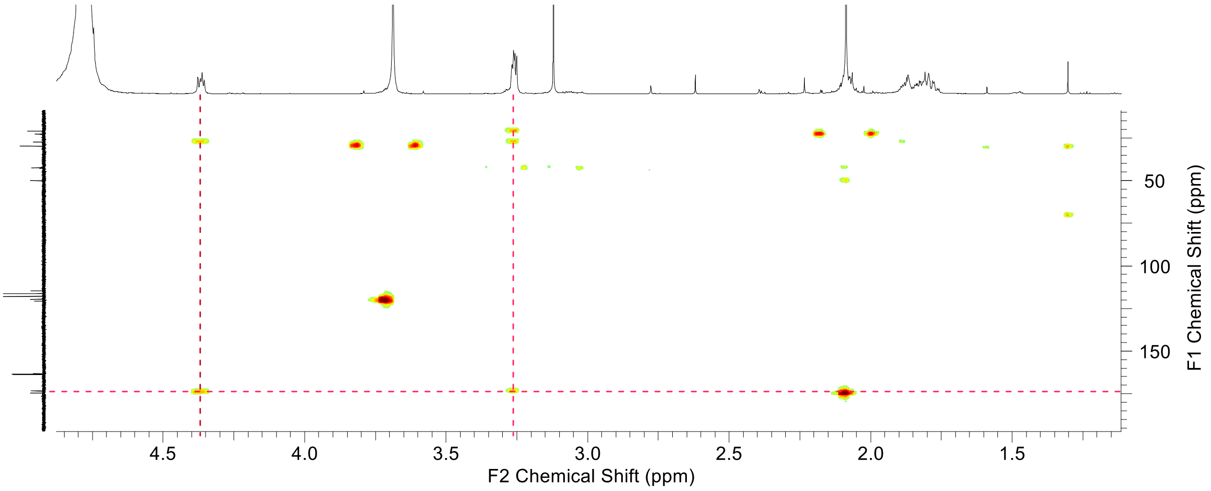

*Supplementary Figure 9. ^{1}H-^{13}C HMBC (^{1}H-700 MHz [1.5-4.5 ppm], ^{13}C-176 MHz [0-230 ppm], D_2O) spectrum showing the $^{2}J_{CH}$ and $^{3}J_{CH}$ couplings of Orn-(C2)–H at 4.37 ppm and Orn-(C5)–H_2 at 3.26 ppm of **2** to C=O resonance at 173.4 ppm, which is diagnostic for lactam formation.*

2 (♦): **^{1}H NMR** (700 MHz, D_2O) δ_H 4.37 (dd, J = 10.4, 6.4 Hz, 1H, (C2)–H), 3.26 (m, 2H, (C5)–H_2), 2.05-2.11 (m, 1H, (C3)–H), 2.08 (s, 3H, $COCH_3$), 1.76-1.90 (obs. m, 3H, (C3)–H', (C4)–H_2). **^{13}C NMR** (176 MHz, D_2O) δ_C 174.6 (**C**OCH_3), 173.4 (C1), 50.2 (C2), 42.4 (C5), 27.5 (C3), 22.9 (CO**C**H_3), 21.1 (C4). **HRMS-ESI** $[M+H]^+$ calc. for $C_7H_{13}N_2O_2^+$ 157.0972; obs 157.0972.

Gly-CN: **^{1}H NMR** (700 MHz, D_2O) δ_H 3.69 (s, 2H, (C2)–H_2). **^{13}C NMR** (176 MHz, D_2O) δ_C 120.7 (C1), 29.9 (C2).

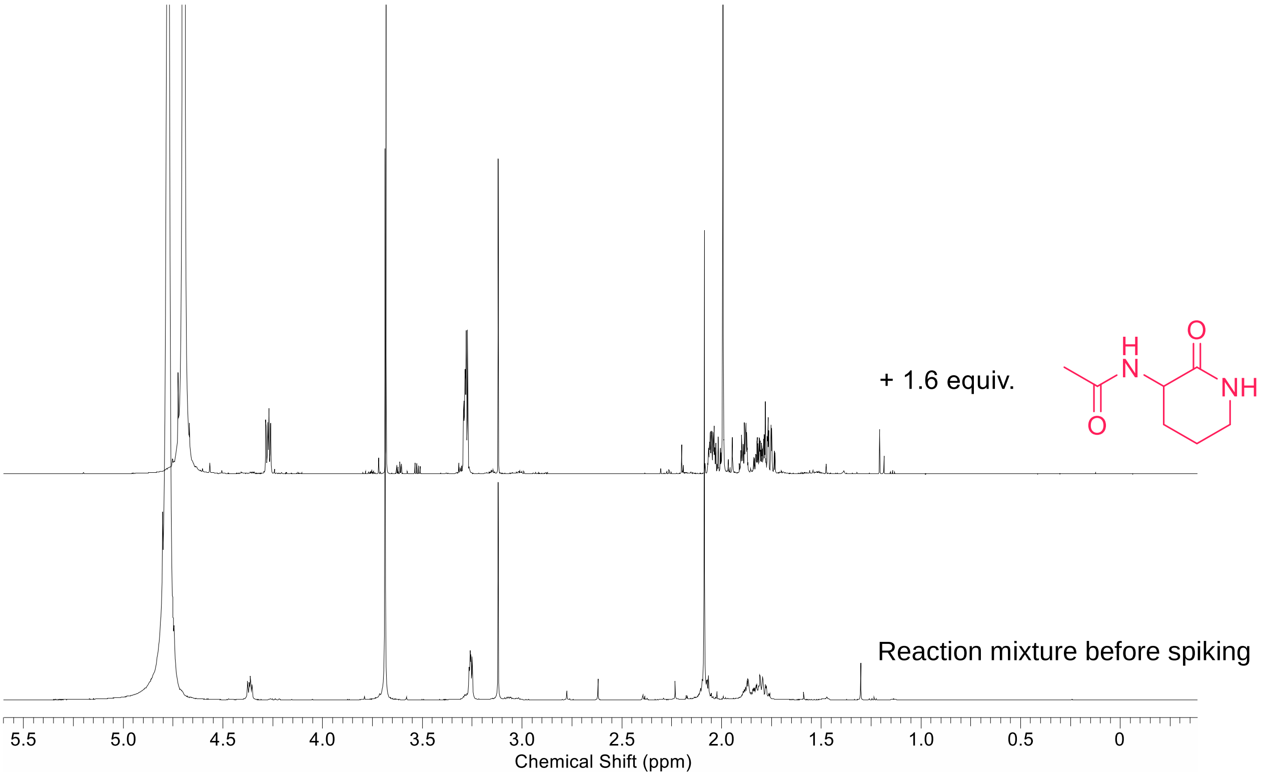

*Supplementary Figure 10. ^{1}H NMR (700 MHz, D_2O, 0.0 – 5.5 ppm) spectra of **2** and **Gly-CN** formed from the reaction of **Ac-Orn-SH** (60 mM), **Gly-CN** (2 equiv.) and $K_3Fe(CN)_6$ (3 equiv.) at pD 7.5 before (bottom) and after (top) the addition of 1.6 equiv. of authentically synthesised **2**. ABX system visible at ~3.6 ppm (top spectrum) is an impurity leached from Mettler Toledo pH probes.*

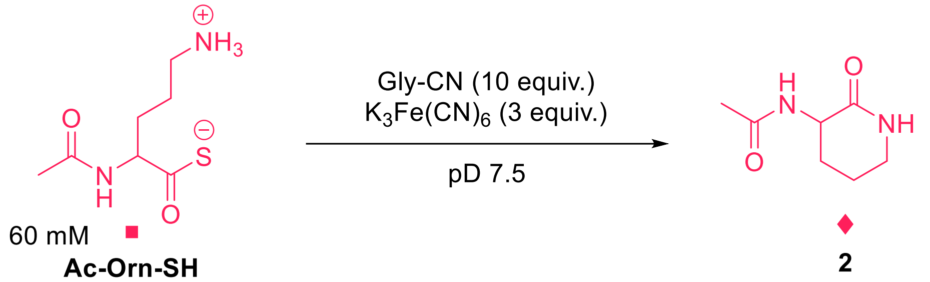

Reaction carried out using **Ac-Orn-SH** (60 mM), **Gly-CN** (10 equiv.), $K_3Fe(CN)_6$ (3 equiv.) at pD 7.5. The reaction mixture was stirred at room temperature for 30 min and then centrifuged. The supernatant was analysed by 1D and 2D NMR spectroscopy, yielding **2** (94%).

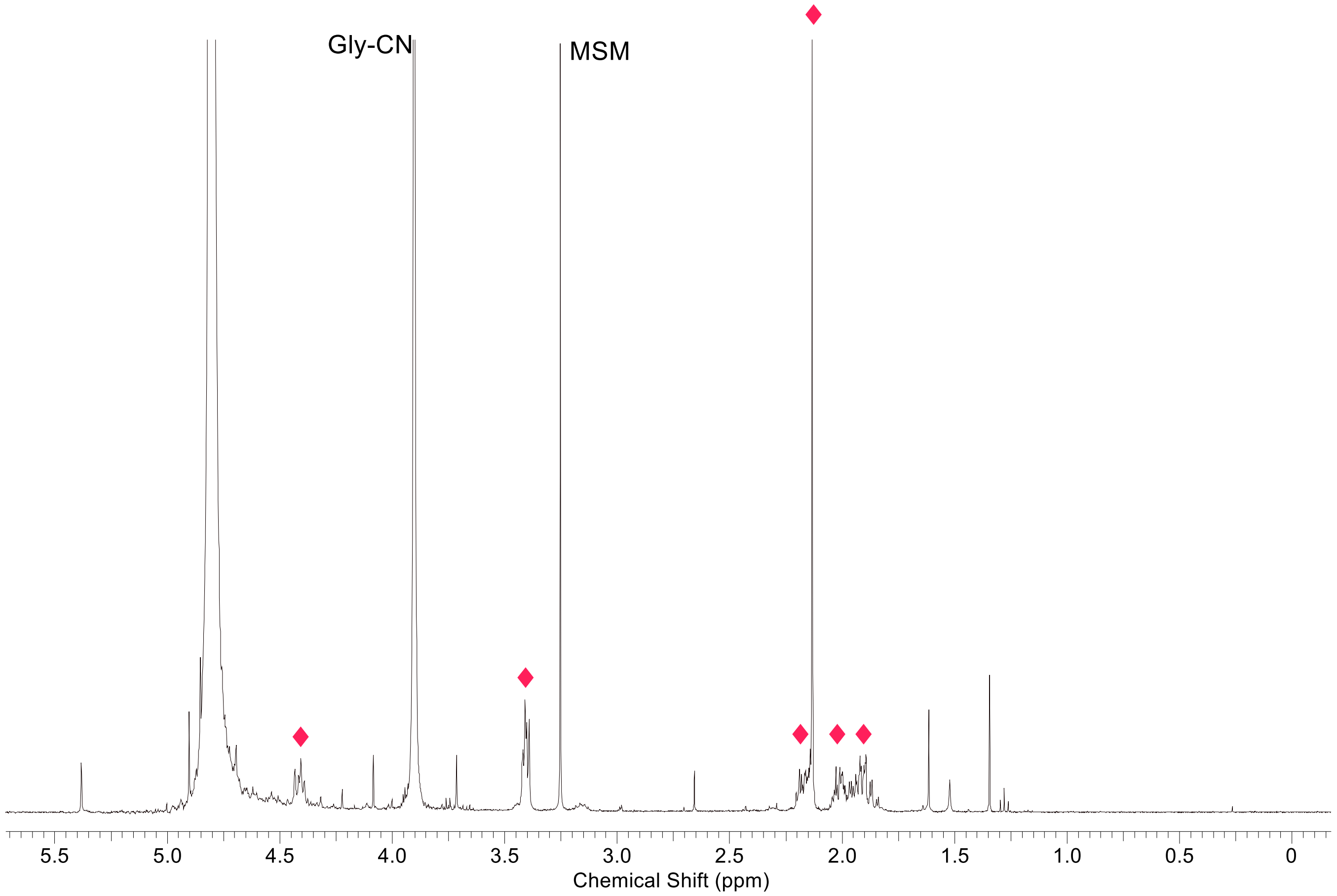

*Supplementary Figure 11. ^{1}H NMR (400 MHz, D_2O, 0.0 – 5.5 ppm) spectrum of **2** and **Gly-CN** formed from the reaction of **Ac-Orn-SH** (60 mM), **Gly-CN** (10 equiv.) and $K_3Fe(CN)_6$ (3 equiv.) at pD 7.5.*

Ac-Orn-SH cyclises to **2** under an ambient atmosphere, so **Ac-Orn-SH** was monitored to ensure that cyclisation to **2** was not occurring before the addition of $K_3Fe(CN)_6$. NMR spectra could be acquired of the reaction mixture (**Ac-Orn-SH** and **Gly-CN**) before the addition of $K_3Fe(CN)_6$. Cyclisation to **2** was then observed following the addition of K_3FeCN_6.

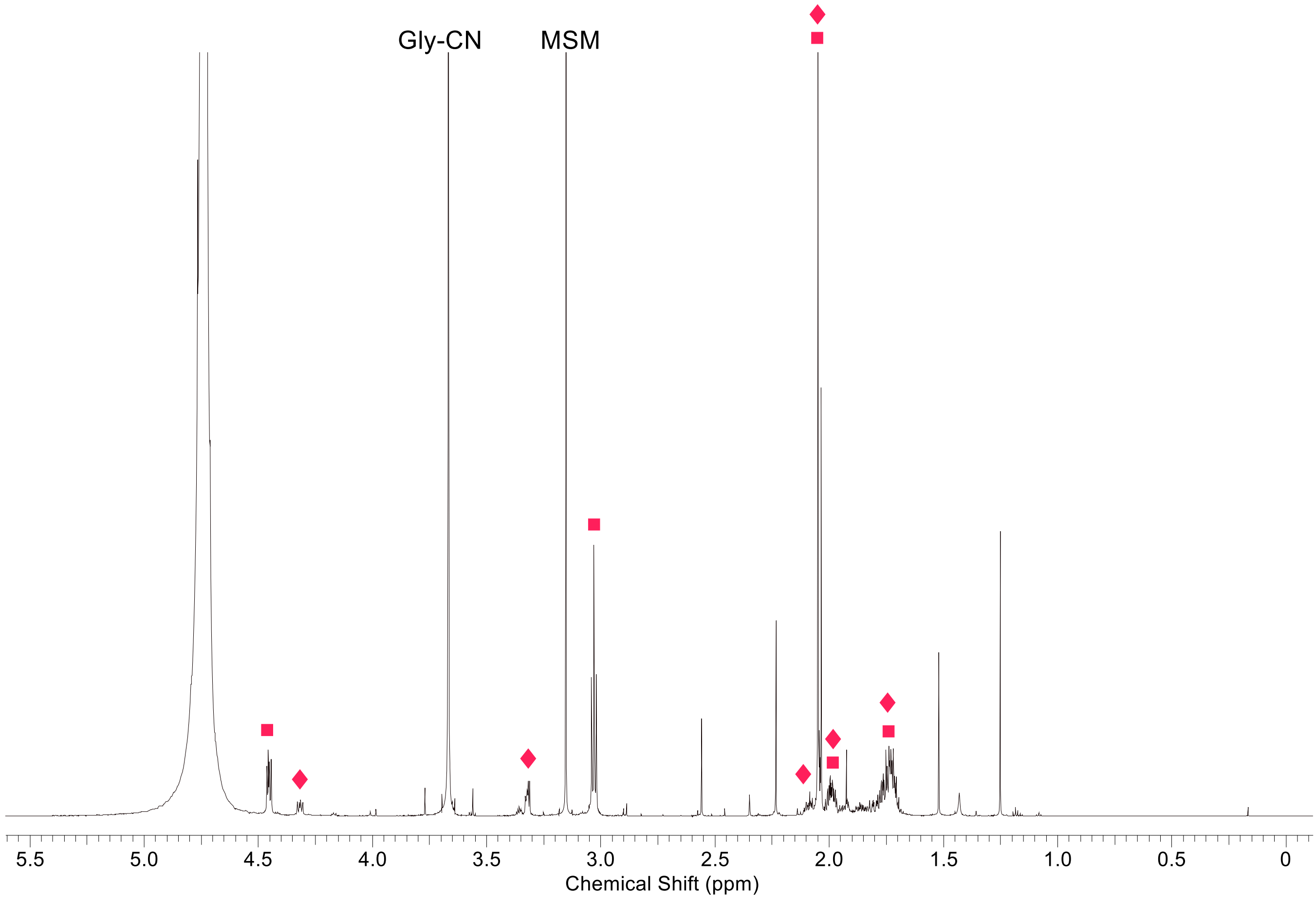

*Supplementary Figure 12. ^1H NMR (400 MHz, D_2O, 0.0 – 5.5 ppm) spectrum showing the resulting mixture formed upon mixing **Ac-Orn-SH** (60 mM) and **Gly-CN** (2 equiv.) at pD 7.5. Cyclisation to **2** is then observed upon addition of $K_3Fe(CN)_6$ to the reaction mixture.*

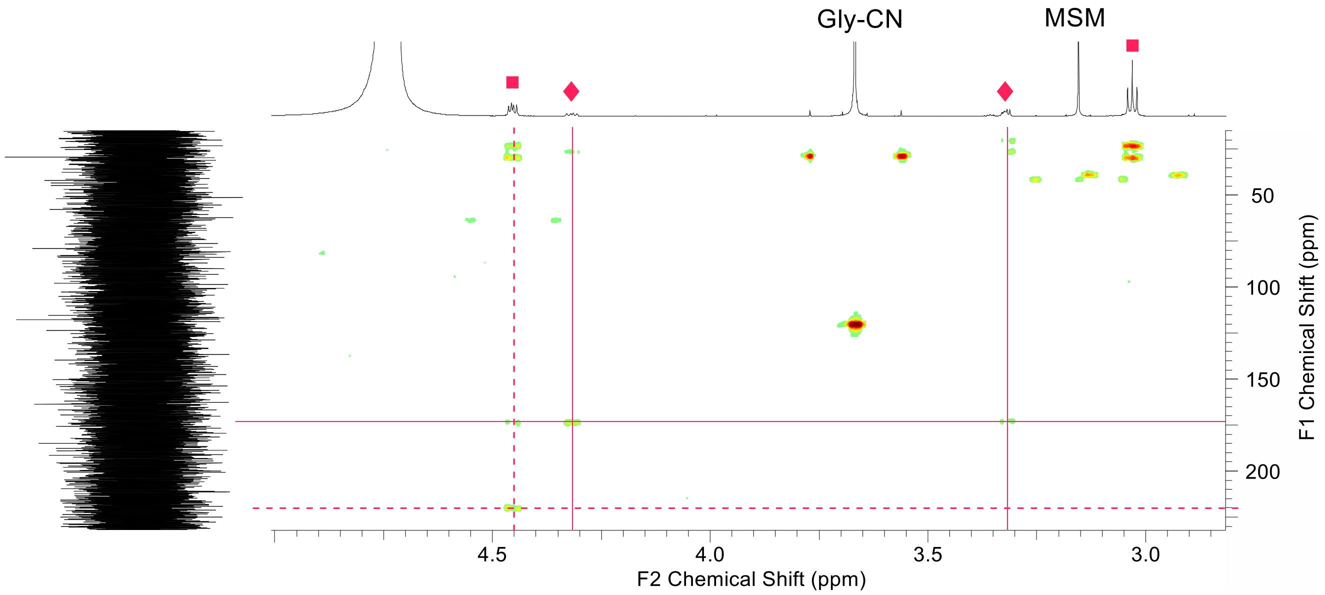

*Supplementary Figure 13. ^1H-^{13}C HMBC (^1H-700 MHz [2.8 – 5.0 ppm], ^{13}C-176 MHz [25-225 ppm], D_2O) spectrum showing **Gly-CN** and the $^2J_{CH}$ coupling of Orn-(C2)–H at 4.45 ppm of **Ac-Orn-SH** to C=O resonance*

*at 220.6 ppm, and the $^{2}J_{CH}$ and $^{3}J_{CH}$ couplings of Orn-(C2)–H at 4.33 ppm and Orn-(C5)–H_2 at 3.32 ppm of **2** to C=O resonance at 173.4 ppm, which are diagnostic for thioacid and lactam formation, respectively.*

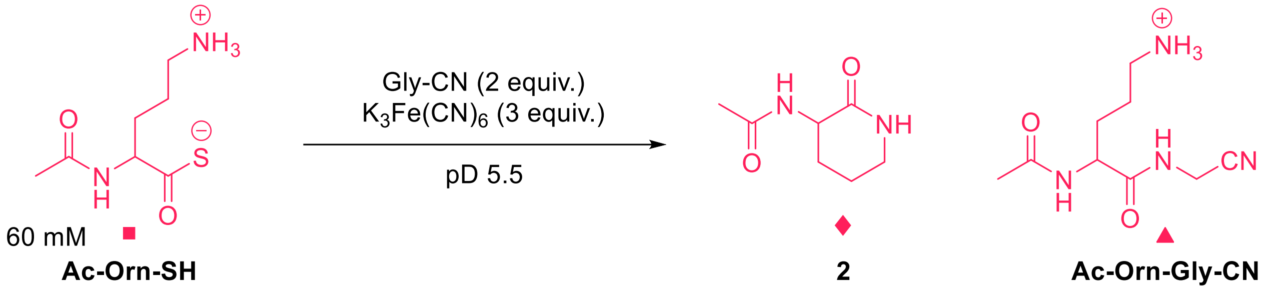

Reaction carried out using **Ac-Orn-SH** (60 mM), **Gly-CN** (2 equiv.), $K_3Fe(CN)_6$ (3 equiv.) at pD 5.5. The reaction mixture was stirred at room temperature for 30 min and then centrifuged. The supernatant was analysed by 1D and 2D NMR spectroscopy, yielding **2** (67%) and **Ac-Orn-Gly-CN** (<10%).

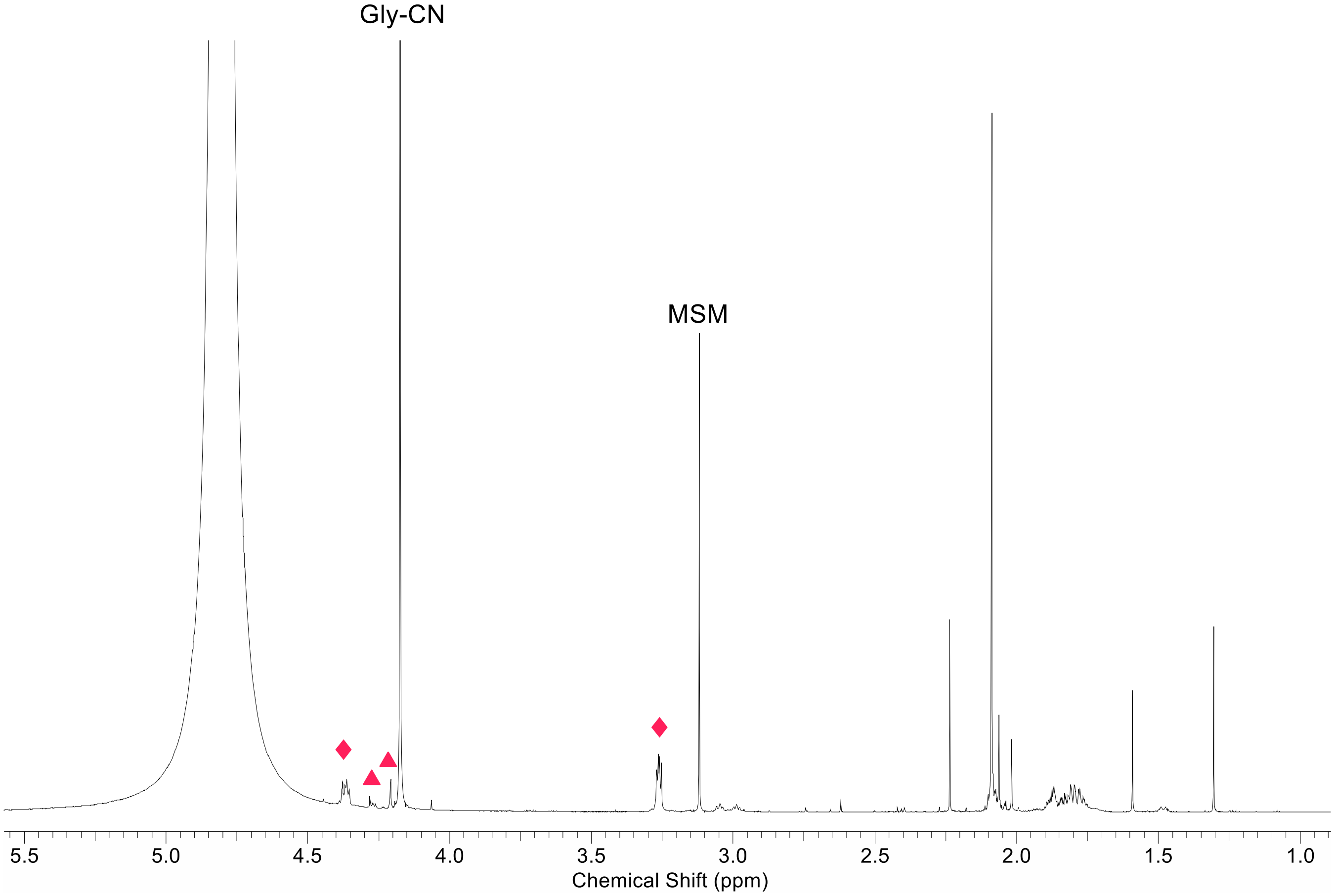

*Supplementary Figure 14. ^{1}H NMR (700 MHz, D_2O, 1.0 – 5.5 ppm) spectrum of **2** and **Ac-Orn-Gly-CN** formed from the reaction of **Ac-Orn-SH** (60 mM), **Gly-CN** (2 equiv.) and $K_3Fe(CN)_6$ (3 equiv.) at pD 5.5.*

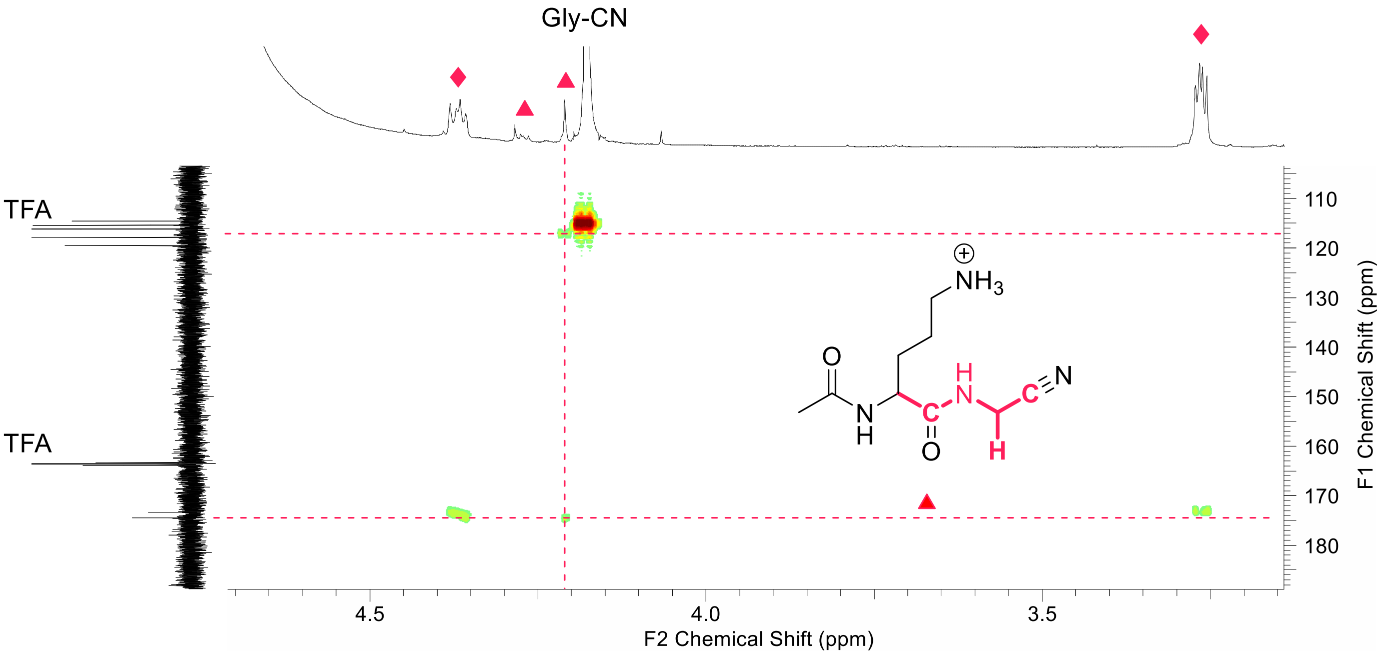

Supplementary Figure 15. *^{1}H-^{13}C HMBC (^{1}H-700 MHz [3.1 – 4.7 ppm], ^{13}C-176 MHz [103 – 188 ppm], D_2O) spectrum showing the $^{2}J_{CH}$ coupling of Gly-(C2)–H_2 at 4.21 ppm of* ***Ac-Orn-Gly-CN*** *to C=O resonance at 174.7 ppm and CN resonance at 117.3 ppm, indicative of amide bond formation.*

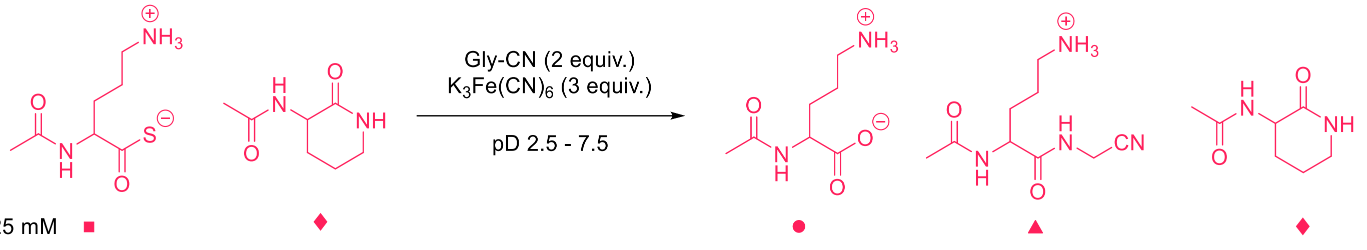

Reaction carried out using **Ac-Orn-SH** (25 mM), **Gly-CN** (2 equiv.) and $K_3Fe(CN)_6$ (3 equiv.) at either pD 2.5, 3.5, 5.5 or 7.5. The reaction mixture was stirred at room temperature for 30 min and then centrifuged. The supernatant was analysed by 1D and 2D NMR spectroscopy.

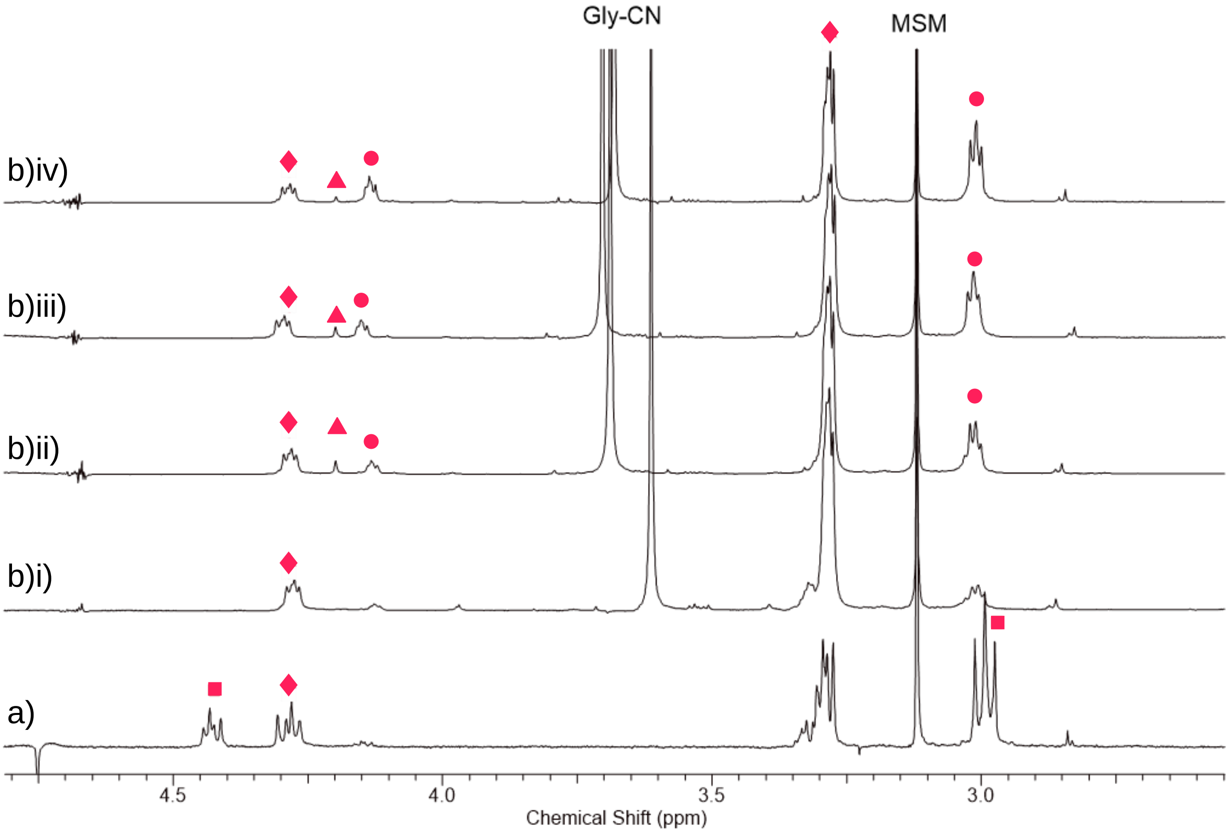

*Supplementary Figure 16. ^{1}H NMR (700 MHz, D_2O, 2.60 – 4.80, noesygppr1d) spectra showing a) lactam **2** (25 mM) and **Ac-Orn-SH** (25 mM) at pD 7.5; b) addition of **Gly-CN** (2.0 equiv.) and $K_3Fe(CN)_6$ (3.0 equiv.) at (i): pD 7.5; (ii) pD 5.5 (iii) pD 3.5; and (iv) pD 2.5.*

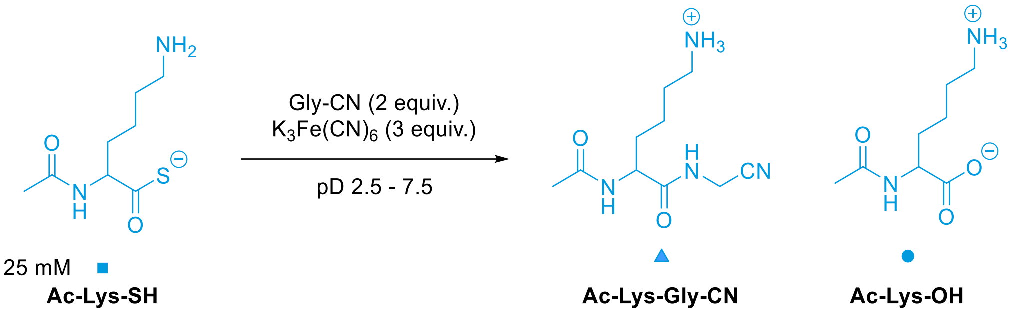

Reaction carried out using **Ac-Lys-SH** (25 mM), **Gly-CN** (2 equiv.) and $K_3Fe(CN)_6$ (3 equiv.) at either pD 2.5, 3.5, 5.5 or 7.5. The reaction mixture was stirred at room temperature for 30 min and then centrifuged. The supernatant was analysed by 1D and 2D NMR spectroscopy.

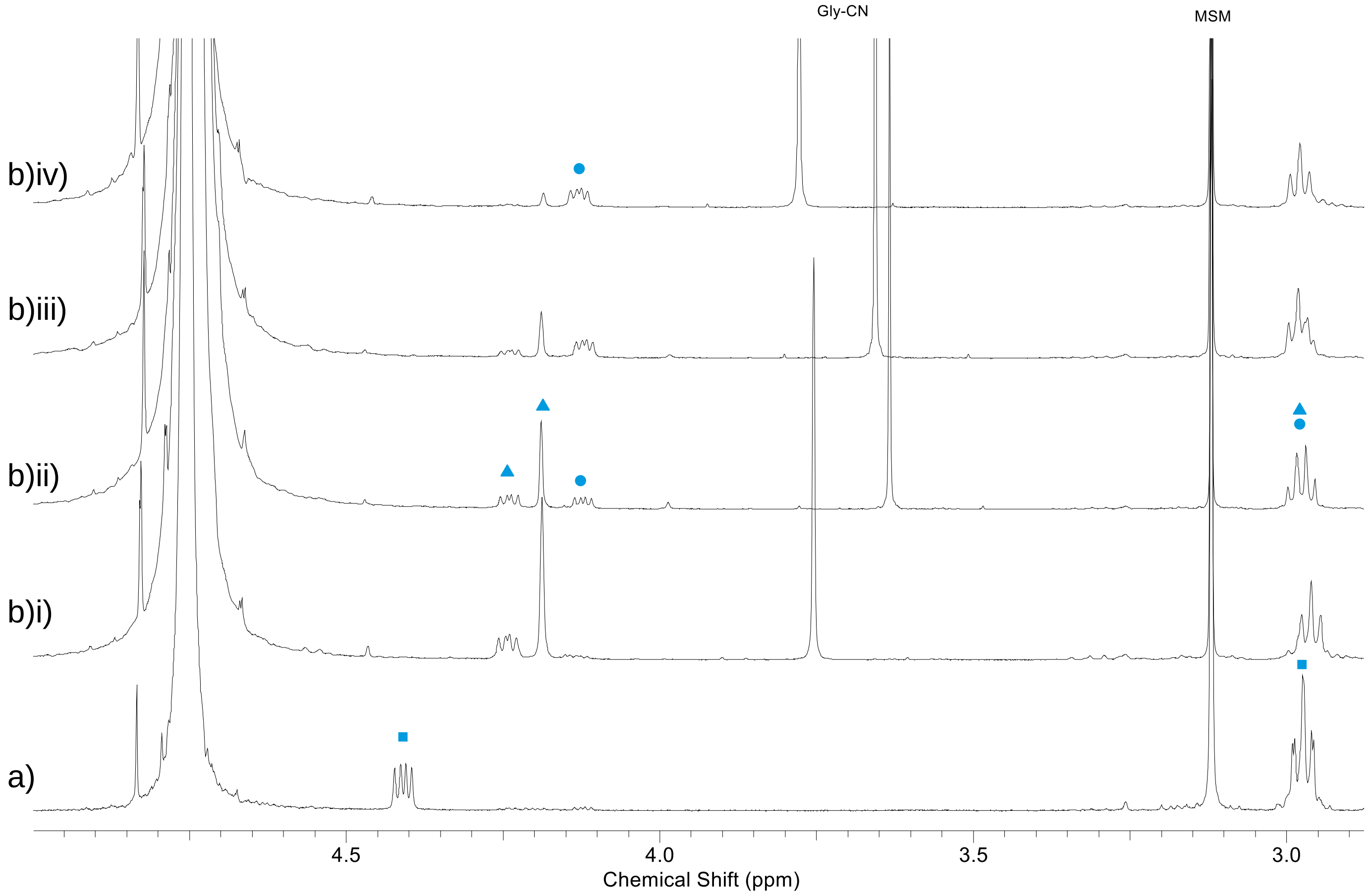

*Supplementary Figure 17. ^{1}H NMR (500 MHz, D_2O, 2.90 – 4.90) spectra showing a) **Ac-Lys-SH** at pD 7.5; b) addition of **Gly-CN** (2.0 equiv.) and $K_3Fe(CN)_6$ (3.0 equiv.) at (i): pD 7.5; (iii) pD 5.5 (iv) pD 3.5; and (v) pD 2.5.*

Entry	AA	pD	Ac-AA-Gly-CN %	Cyclisation %	Ac-AA-OH %
1	Lys	7.5	90	< 10	8
2	Orn	7.5	< 1	90	10
3	Lys	5.5	53	<10	49
4	Orn	5.5	5	70	20
5	Lys	3.5	25	< 5	60
6	Orn	3.5	5	60	25
7	Lys	2.5	10	< 5	75
8	Orn	2.5	< 5	50	55

*Supplementary Table 2. ^{1}H NMR yields for the reaction of **Ac-AA-SH** (25 mM), **Gly-CN** (2 equiv.) and $K_3Fe(CN)_6$ (3 equiv.) at room temperature and initiated at the specified pD.*

Attempted prebiotic coupling of Ac-Dab-SH and Gly-CN

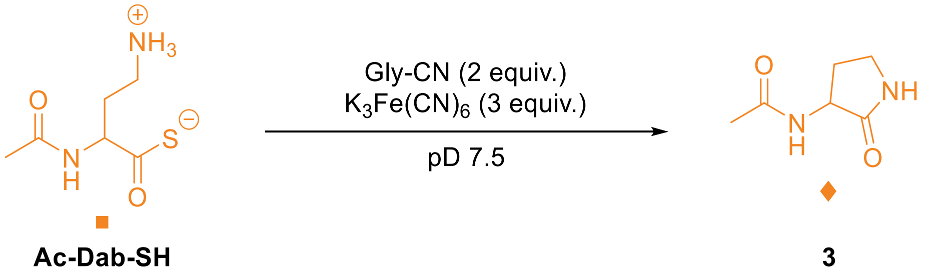

Reactions were carried out *via* general procedure **A** using **Ac-Dab-SH** to afford **3** (95%).

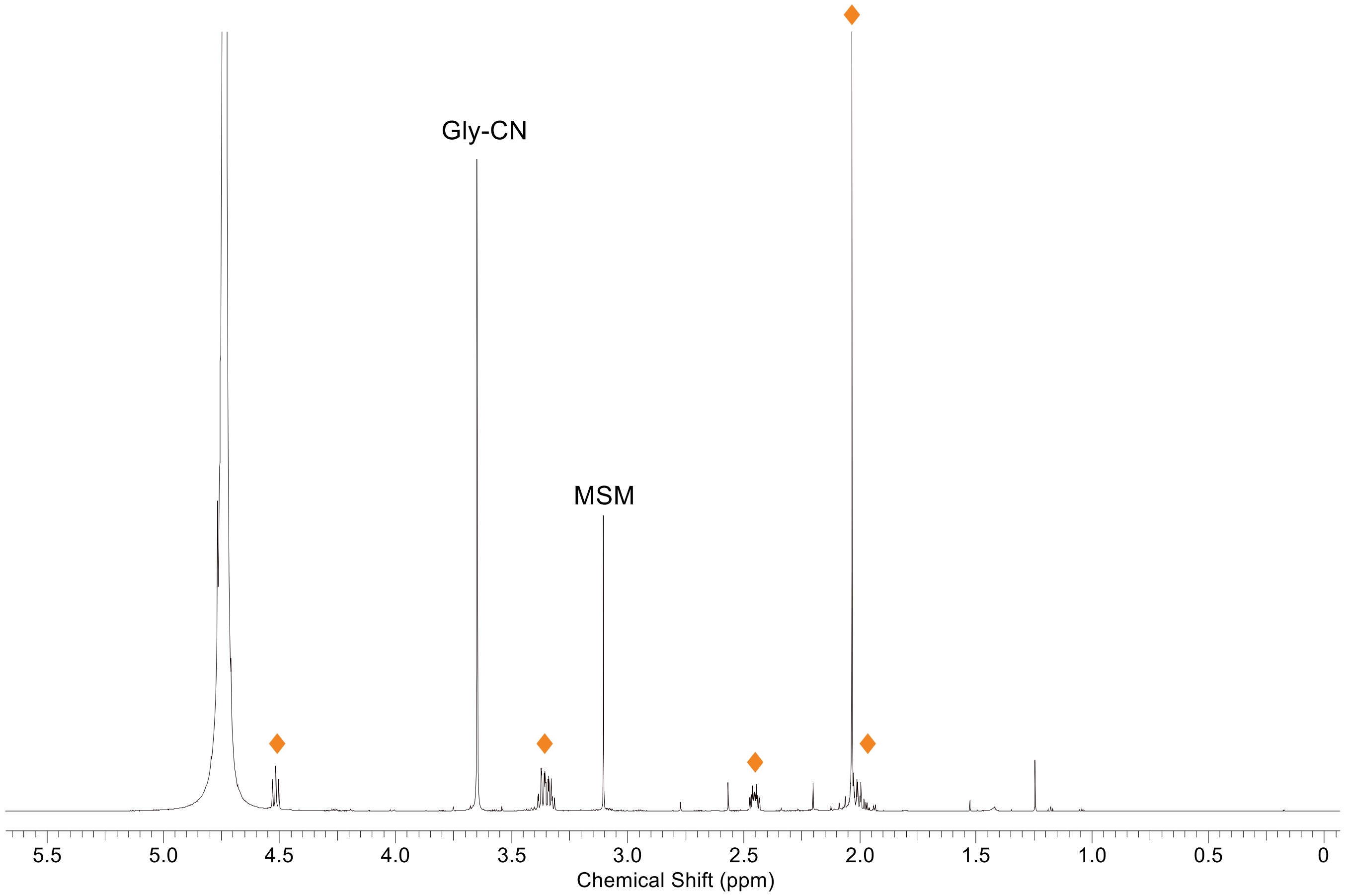

*Supplementary Figure 18. ^{1}H NMR (700 MHz, D_2O, 0.0 – 5.5 ppm) spectrum of **3** and **Gly-CN** formed from the reaction of **Ac-Dab-SH**, **Gly-CN** and $K_3Fe(CN)_6$ at pD 7.5.*

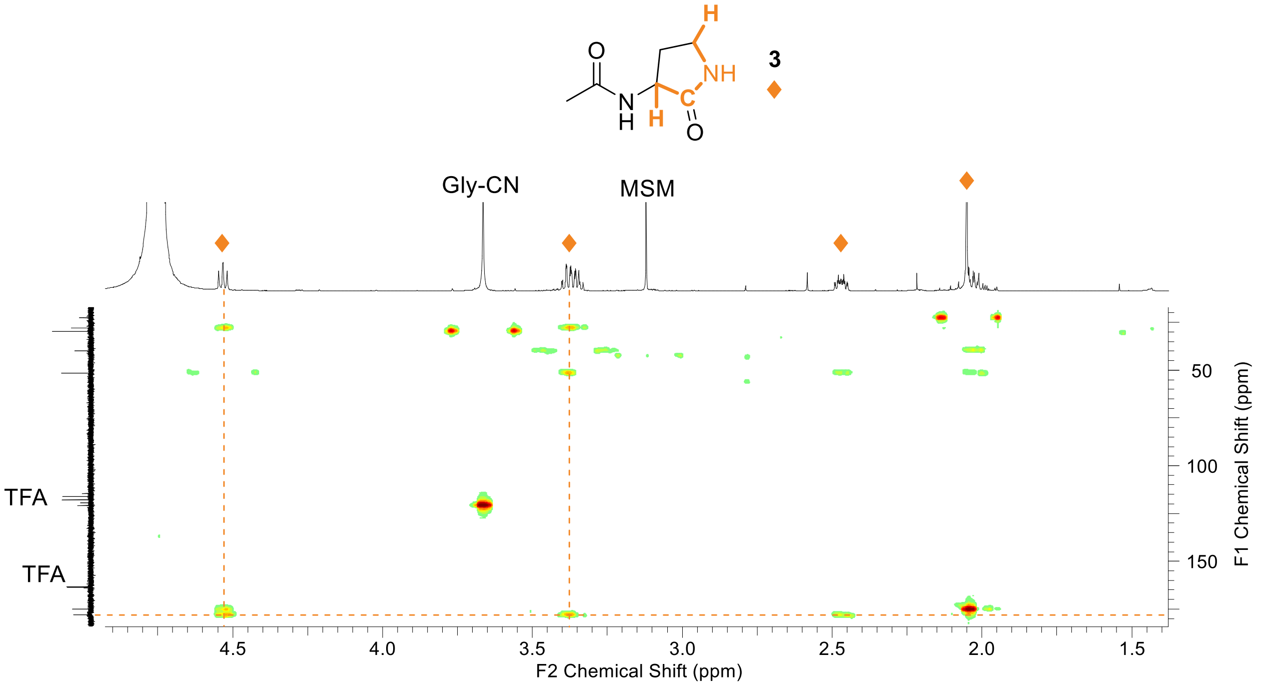

*Supplementary Figure 19. ^1H-^{13}C HMBC (^1H-700 MHz [1.4-4.9 ppm], ^{13}C-176 MHz [0-185 ppm], D_2O) spectrum showing the $^2J_{CH}$ and $^3J_{CH}$ couplings of Dab-(C2)–H at 4.55 ppm and Dab-(C4)–H_2 at 3.38 ppm of **3** to C=O resonance at 178.0 ppm, which are diagnostic for lactam formation.*

3 (♦): **^{1}H NMR** (700 MHz, D_2O) δ_H 4.53 (t, *J* = 9.8 Hz, 1H, (C2)–H), 3.33-3.40 (m, 2H, (C4)–H_2), 2.45-2.49 (m, 1H, (C3)–H), 2.05 (s, 3H, $COCH_3$), 1.98-2.08 (m, 1H, (C3)–H'). **^{13}C NMR** (176 MHz, D_2O) δ_C 178.0 (C1), 174.9 (**C**OCH_3), 51.5 (C2), 40.0 (C4), 27.9 (C3), 22.7 (CO**C**H_3). **HRMS-ESI** $[M+H]^+$ calc. for $C_6H_{11}N_2O_2^+$ 143.0815; obs 143.0816.

Gly-CN: **^{1}H NMR** (700 MHz, D_2O) δ_H 3.66 ((C2)–H_2). **^{13}C NMR** (176 MHz, D_2O) δ_C 120.8 (C1), 29.7 (C2).

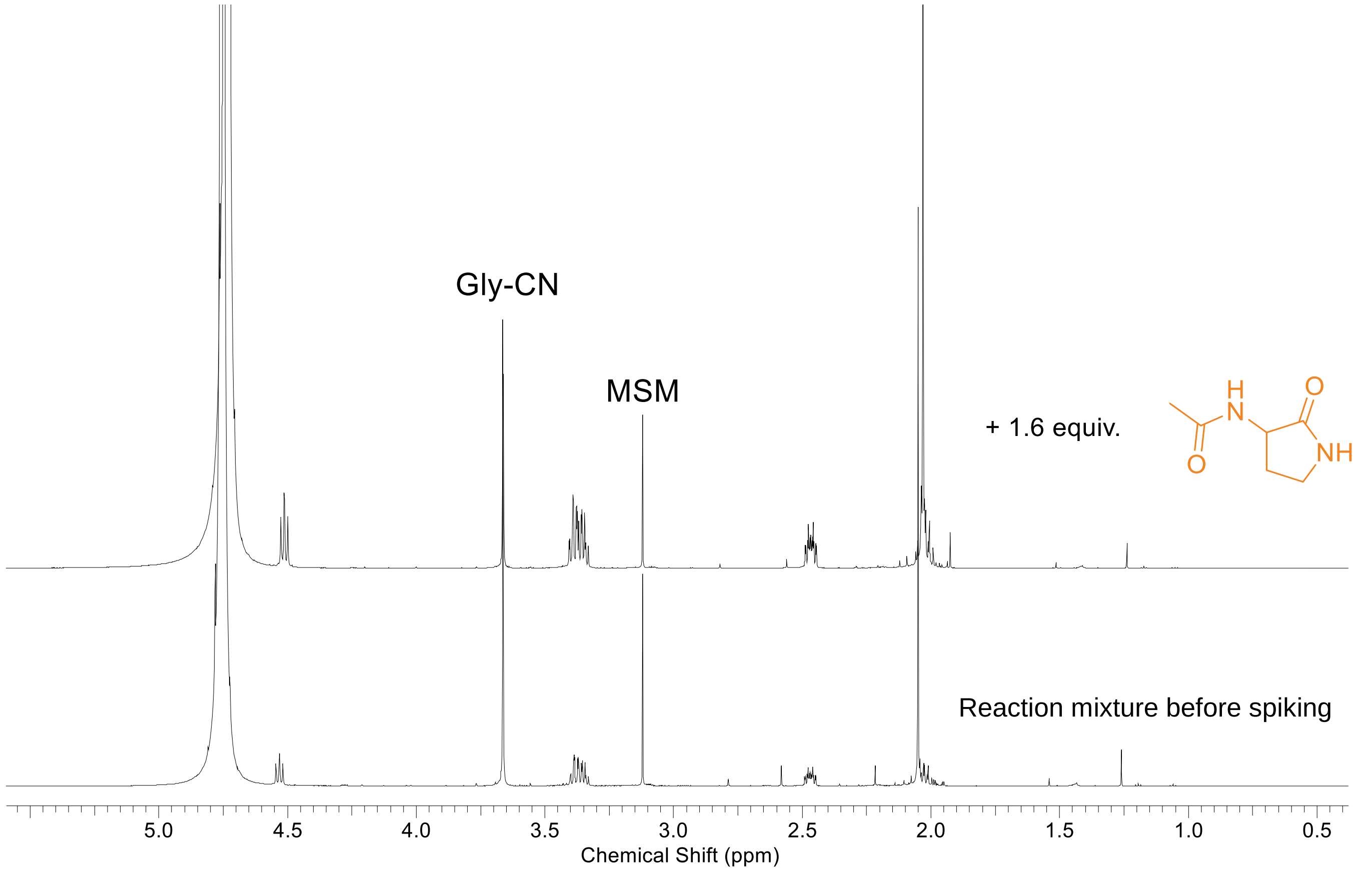

*Supplementary Figure 20. ^{1}H NMR (700 MHz, D_2O, 0.0 – 5.5 ppm) spectra of **3** and **Gly-CN** formed from the reaction of **Ac-Dab-SH**, **Gly-CN** and $K_3Fe(CN)_6$ at pD 7.5 before (bottom) and after (top) the addition of 1.6 equiv. of authentic **3**.*

Prebiotic couplings of Ac-Dpr-SH and Gly-CN

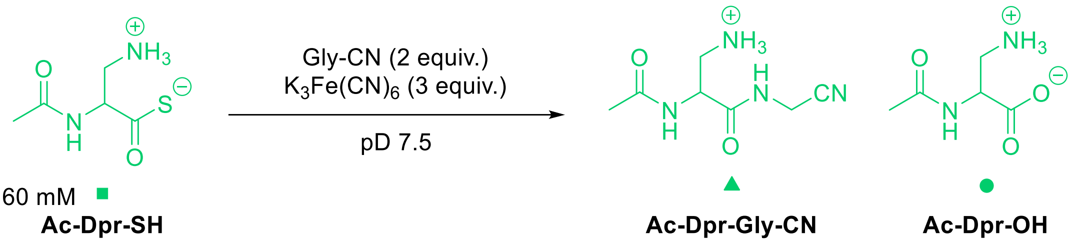

Reaction carried out *via* general procedure **A** using **Ac-Dpr-SH** to afford **Ac-Dpr-Gly-CN** (45%) and **Ac-Dpr-OH** (20%).

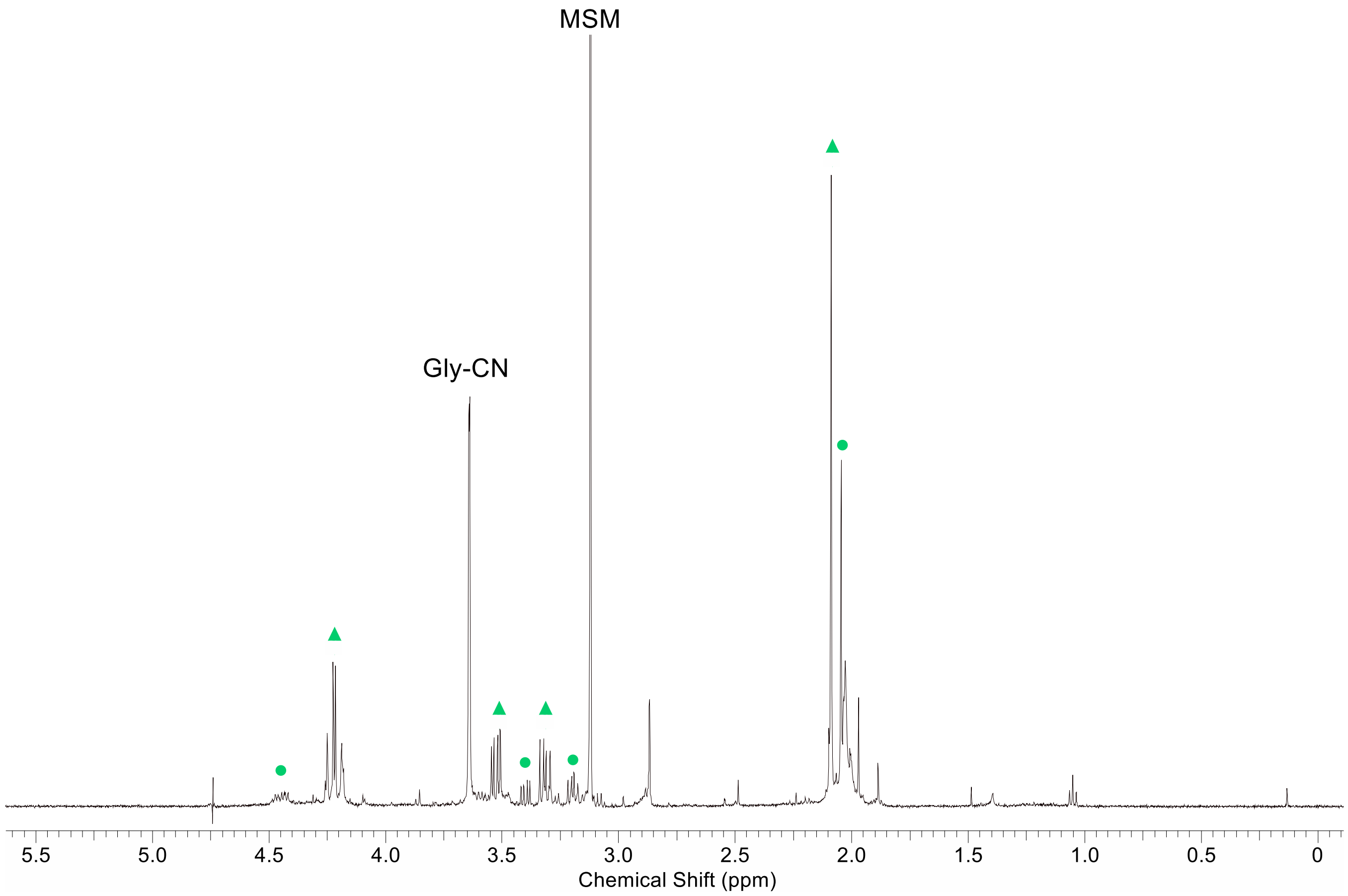

*Supplementary Figure 21. ^{1}H NMR (700 MHz, D_2O, 0.0 – 5.5 ppm) spectrum showing the reaction of **Ac-Dpr-SH** (60 mM), **Gly-CN** (2 equiv.) and $K_3Fe(CN)_6$ (3 equiv.) at pD 7.5.*

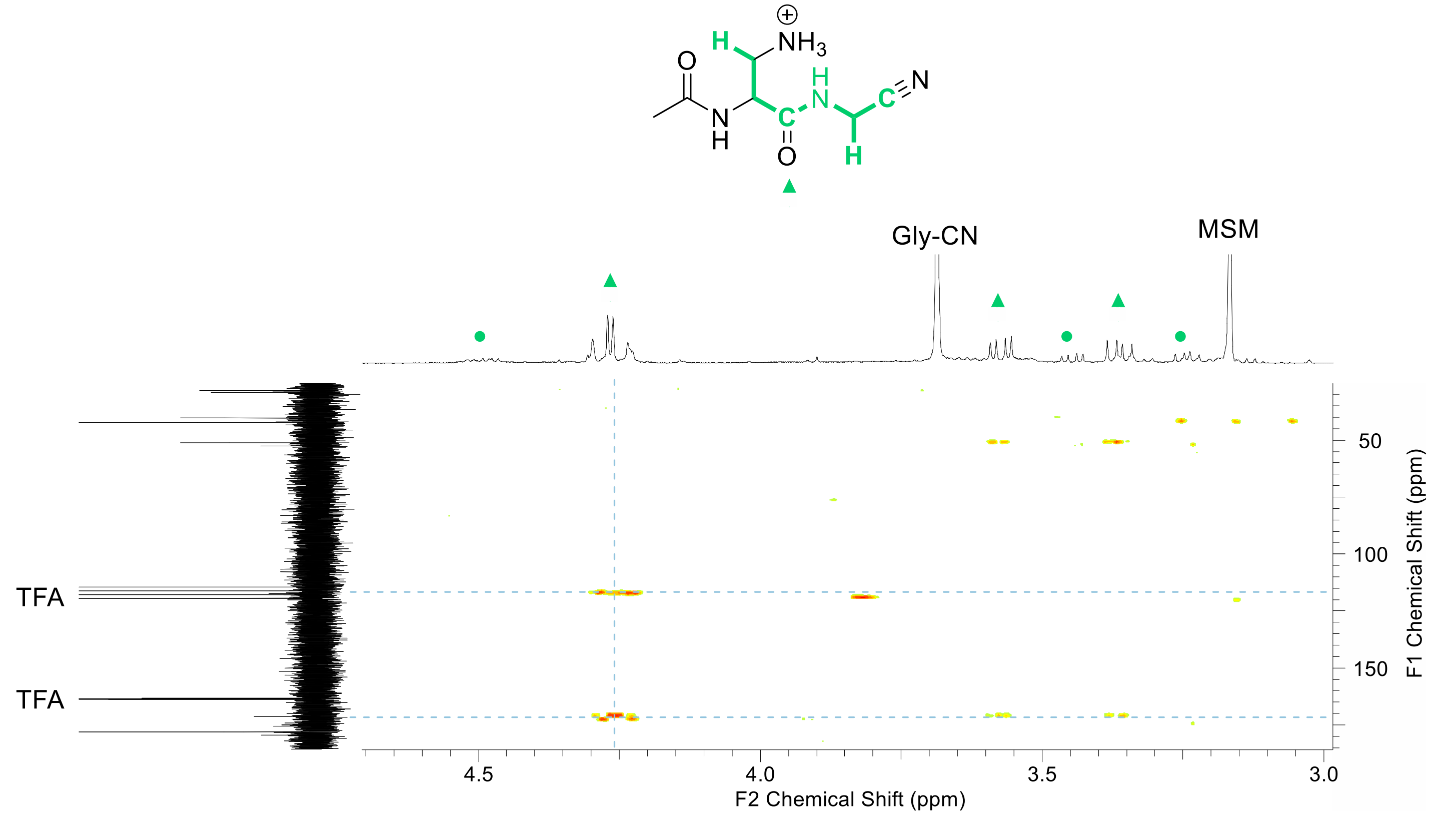

Supplementary Figure 22. ^{1}*H-*^{13}*C HMBC (*^{1}*H-700 MHz [1.8–5.2 ppm],* ^{13}*C-176 MHz [15–185 ppm],* D_2O*) spectrum showing the* $^{2}J_{CH}$ *and* $^{3}J_{CH}$ *couplings of Gly-(C2)–*H_2 *at 4.20 and 4.25 ppm of* ***Ac-Dpr-Gly-CN*** *to C=O resonance at 171.4 ppm and CN resonance at 117.5 ppm, which is diagnostic for peptide bond formation.* ***Gly-CN*** ^{1}*H resonance is misaligned with* ^{13}*C correlation due to fluctuation in pH between* ^{1}*H and HMBC acquisitions. The Gly-(C2)–*H_2 ^{1}*H resonance is sensitive to pH changes due to the variable protonation state of* ***Gly-CN*** *amine moiety.*

Ac-Dpr-Gly-CN (▲): ^{1}**H NMR** (700 MHz, D_2O) δ_H 4.45-4.49 (obs. ABX, 1H, Dpr-(C2)–H), 4.25 (AB, J = 17.6 Hz, 1H, Gly-(C2)–H), 4.20 (AB, J = 17.6 Hz, 1H, Gly-(C2)–H'), 3.53 (ABX, J = 13.4, 5.3 Hz, 1H, Dpr-(C3)–H), 3.31 (ABX, J = 13.4, 8.4 Hz, 1H, Dpr-(C3)–H') 2.09 (s, 3H, $COCH_3$). ^{13}**C NMR** (176 MHz, D_2O, partial assignment) δ_C 171.7 (Dpr-C1), 117.8 (Gly-C1), 51.6 (Dpr-C2), 23.1 (CO**C**H_3). **HRMS-ESI** $[M+H]^+$ calc. for $C_7H_{13}N_4O_2^+$ 185.1033; obs. 185.1033.

Ac-Dpr-OH (●): ^{1}**H NMR** (700 MHz, D_2O, partial assignment) δ_H 4.43 (ABX, J = 8.3, 5.6 Hz, 1H, (C2)–H), 3.40 (ABX, J = 13.2, 5.6 Hz, 1H, (C3)–H), 3.20 (ABX, J = 13.2, 8.3 Hz, 1H, (C3)–H'), 2.05 (s, 3H, $COCH_3$). ^{13}**C NMR** (176 MHz, D_2O, partial assignment) δ_C 175.4 (Dpr-C1).

Gly-CN: ^{1}**H NMR** (700 MHz, D_2O, partial assignment) δ_H 3.65 (s, 2H, (C2)–H_2) ^{13}**C NMR** (176 MHz, D_2O, partial assignment) δ_C 119.7 (C1).

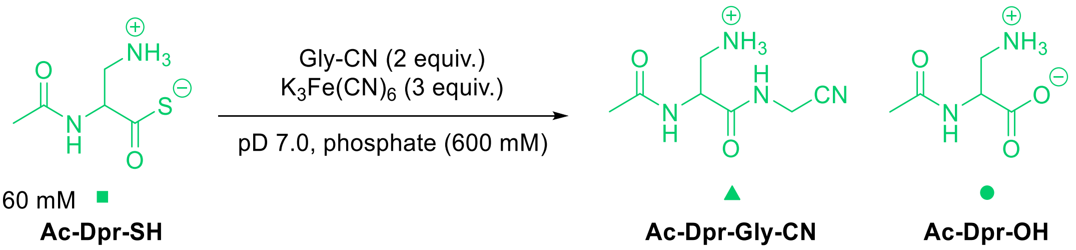

Reaction carried out *via* general procedure **B** using **Ac-Dpr-SH** to afford **Ac-Dpr-Gly-CN** (35%) and **Ac-Dpr-OH** (37%).

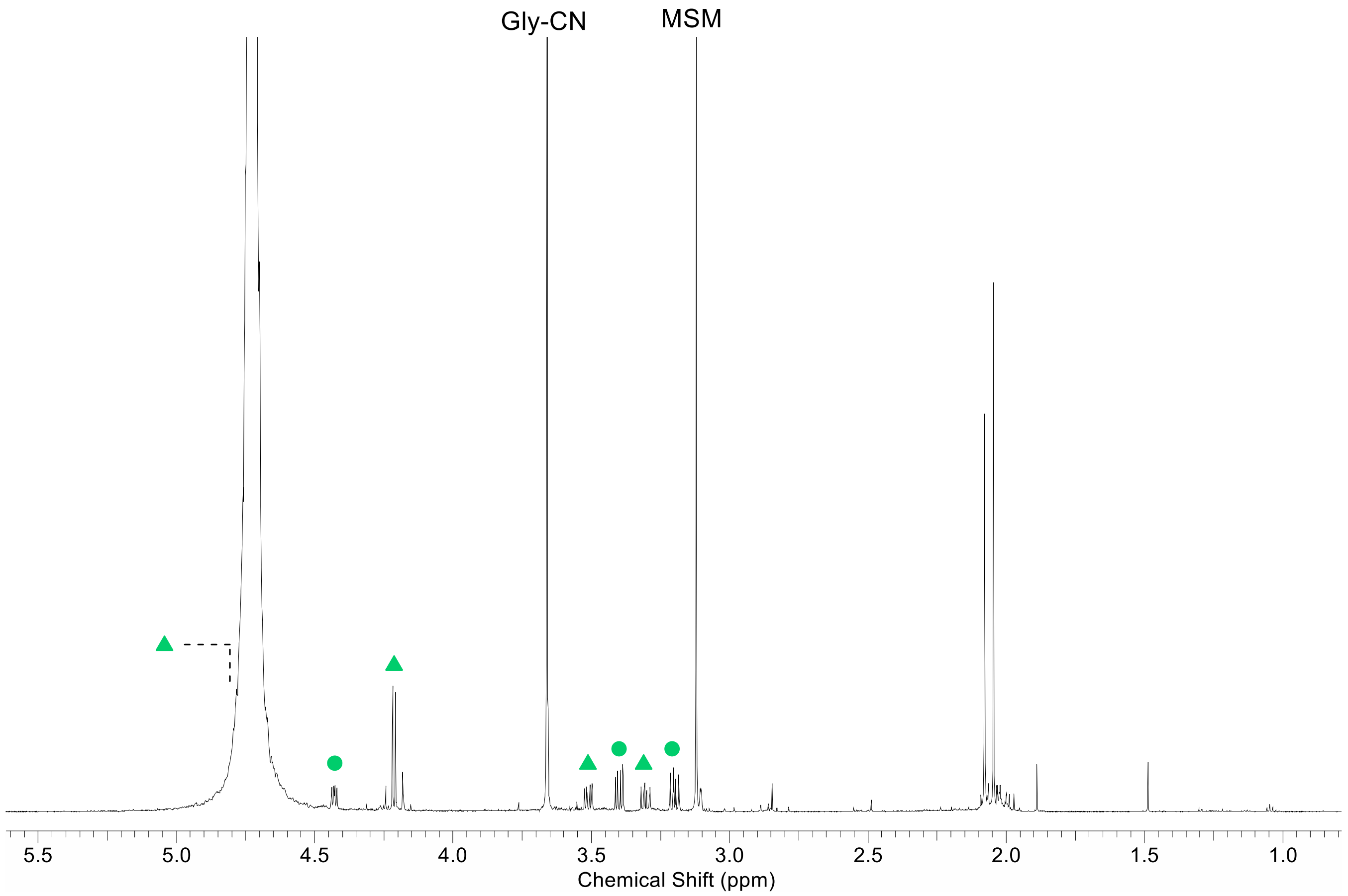

Supplementary Figure 23. ^{1}*H NMR (700 MHz,* D_2O*, 0.0 – 5.5 ppm) spectrum showing the reaction of* ***Ac-Dpr-SH*** *(60 mM),* ***Gly-CN*** *(2 equiv.) and* $K_3Fe(CN)_6$ *(3 equiv.) buffered at pD 7.0 with phosphate (600 mM).*

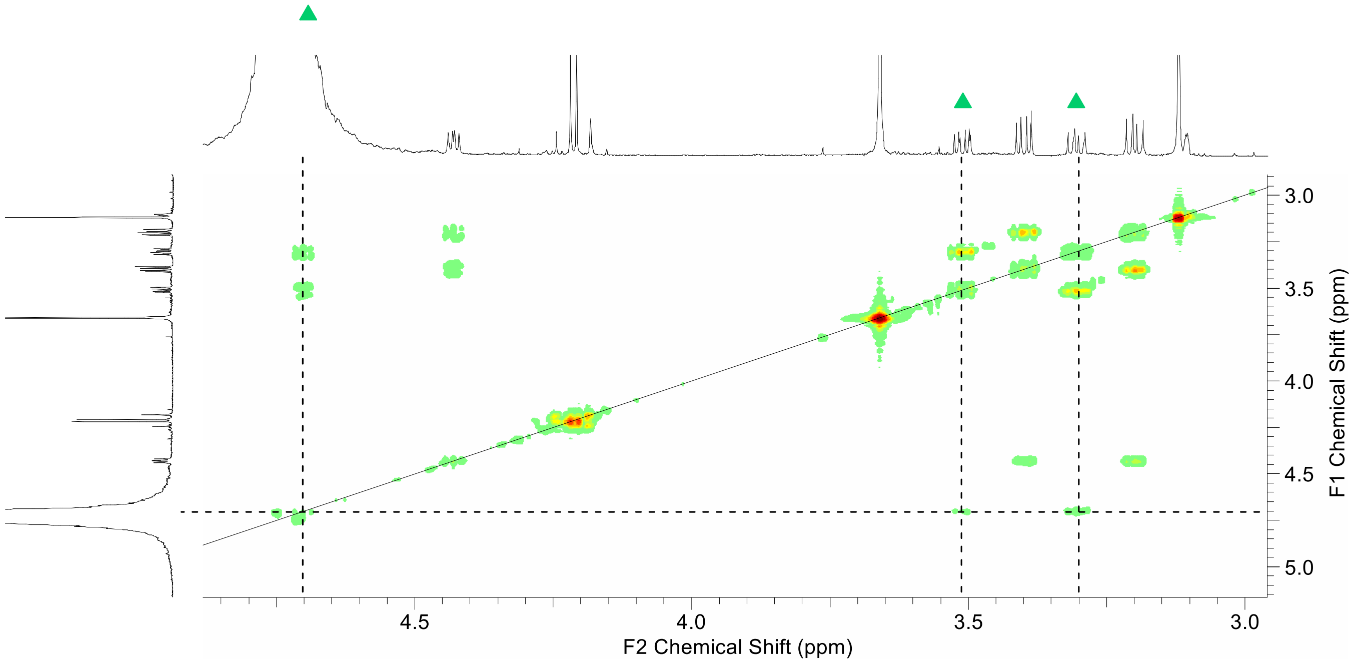

*Supplementary Figure 24. ^1H-^1H COSY (^1H-700 MHz [2.9–5.0 ppm], D_2O) spectrum showing the $^3J_{HH}$ couplings of Dpr-(C2)–H of **Ac-Dpr-Gly-CN**, which resonates at 4.70 ppm, under the HOD signal.*

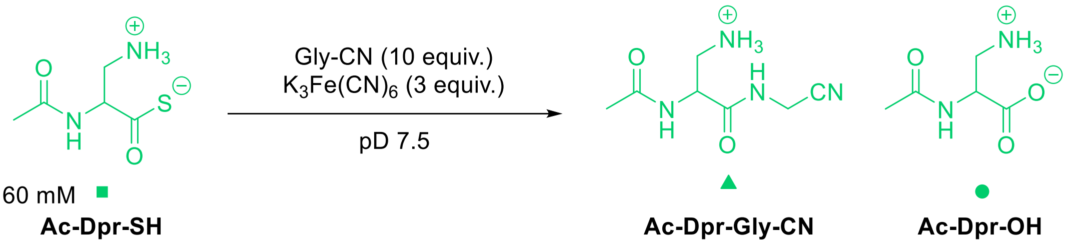

Reaction carried out using **Ac-Dpr-SH** (60 mM), **Gly-CN** (10 equiv.) and $K_3Fe(CN)_6$ at pD 7.5. The reaction mixture was stirred at room temperature for 30 min and then centrifuged. The supernatant was analysed by 1D and 2D NMR spectroscopy, yielding **Ac-Dpr-Gly-CN** (66%) and **Ac-Dpr-OH** (<10%).

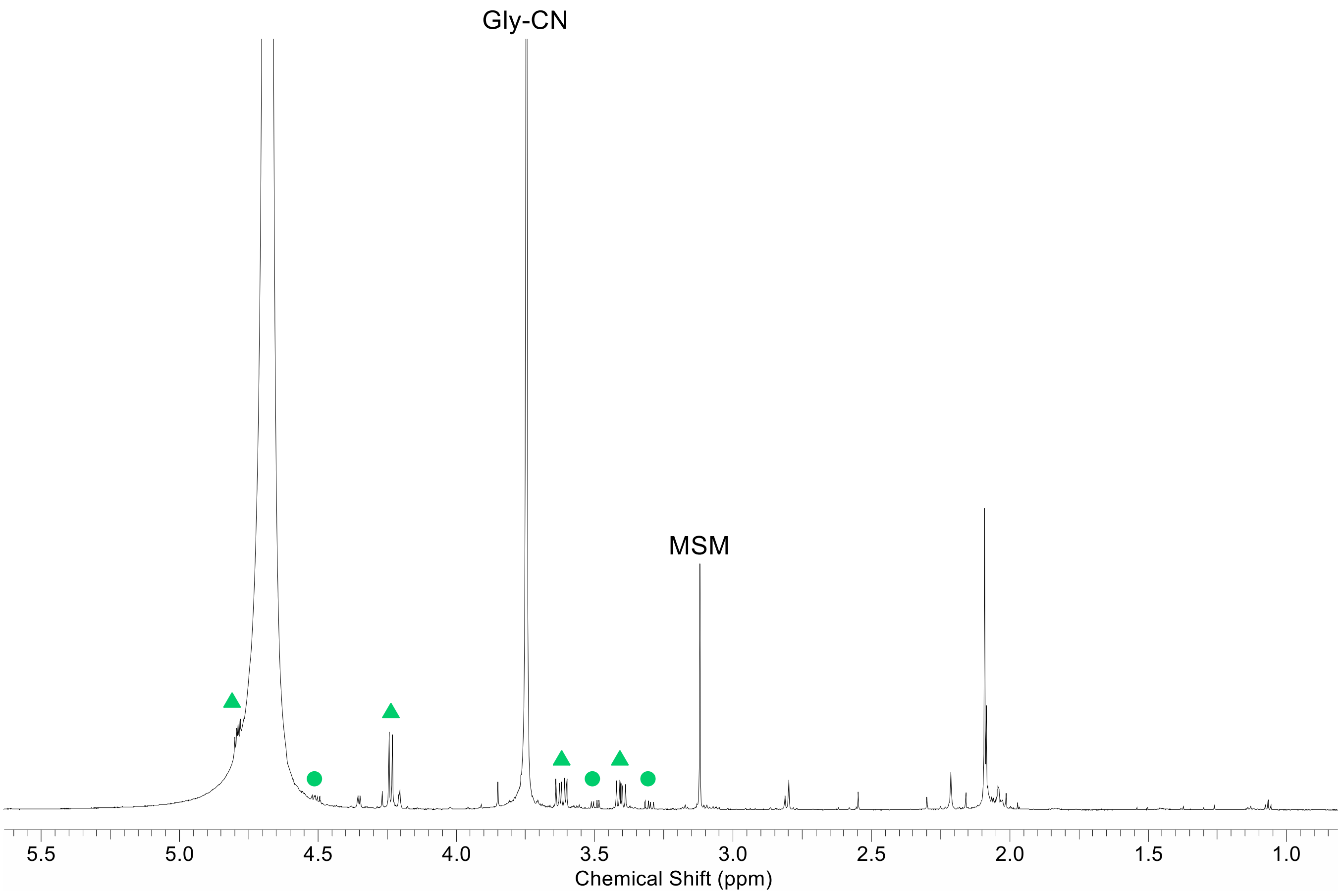

*Supplementary Figure 25. ^{1}H NMR (700 MHz, D_2O, 0.0 – 5.5 ppm) spectrum showing the reaction of **Ac-Dpr-SH** (60 mM), **Gly-CN** (10 equiv.) and $K_3Fe(CN)_6$ (3 equiv.) at pD 7.5.*

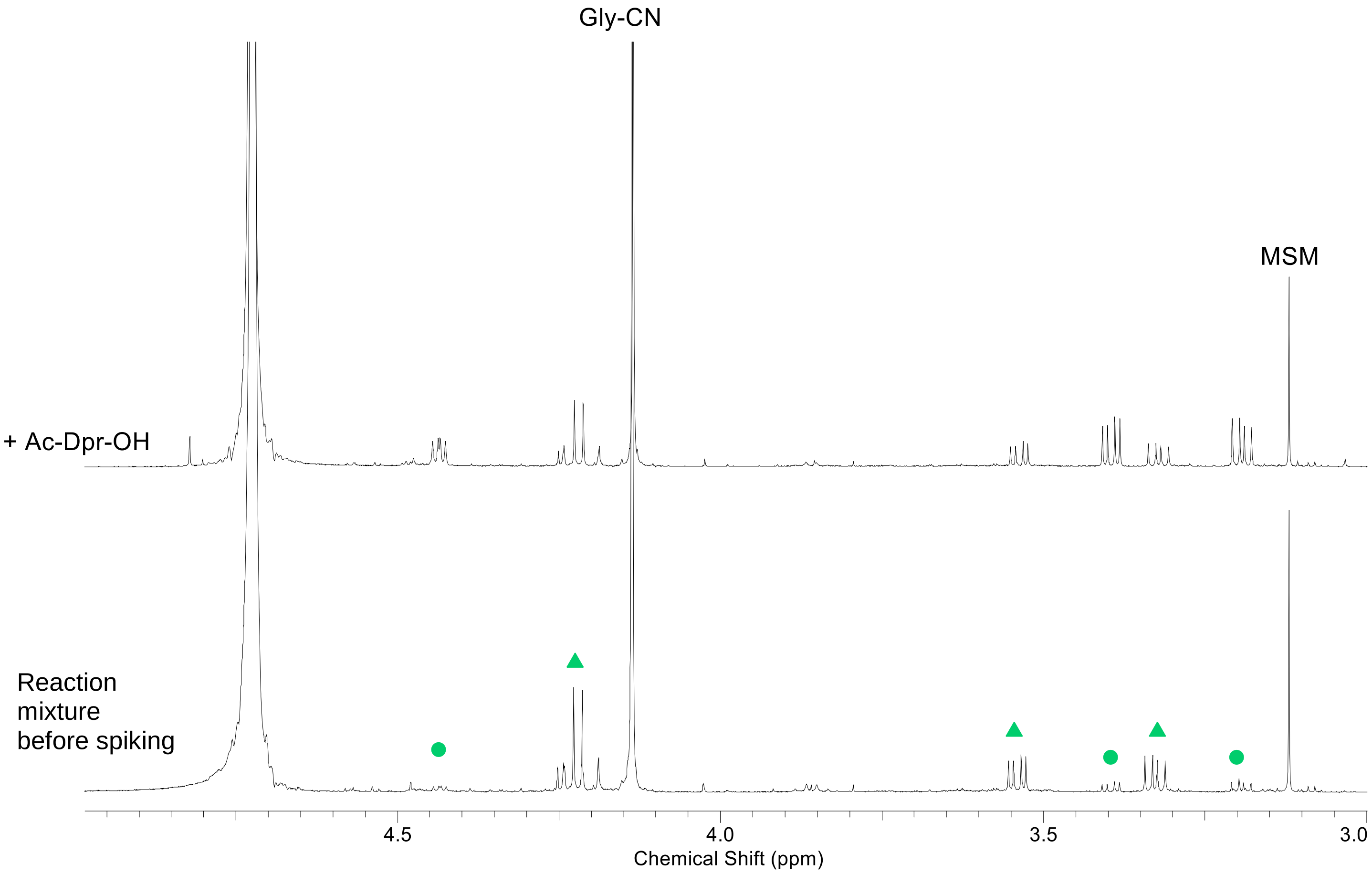

*Supplementary Figure 26. ^{1}H NMR (700 MHz, D_2O, 3.0 – 5.0 ppm) spectra showing the products of the reaction of **Ac-Dpr-SH** (60 mM), **Gly-CN** (10 equiv.) and $K_3Fe(CN)_6$ (3 equiv.) after lyophilisation in D_2O before (bottom ^{1}H NMR spectrum) and after (top ^{1}H NMR spectrum) spiking with authentic **Ac-Dpr-OH**, confirming its presence in the reaction mixture.*

Entry	Gly-CN / equiv.	pD	Buffer	% **Ac-Dpr-Gly-CN**	% **Ac-Dpr-OH**
1	2	7.0	Phosphate	31	37
2	2	7.5	None	45	20
3	10	7.5	None	66	< 10

*Supplementary Table 3. ^{1}H NMR yields for the reaction of **Ac-Dpr-SH** (60 mM), **Gly-CN** and $K_3Fe(CN)_6$ (3 equiv.) at the specified pD and in the presence of the relevant buffer (600 mM, D_2O).*

Investigations into the formation and reactivity of β–lactam 4

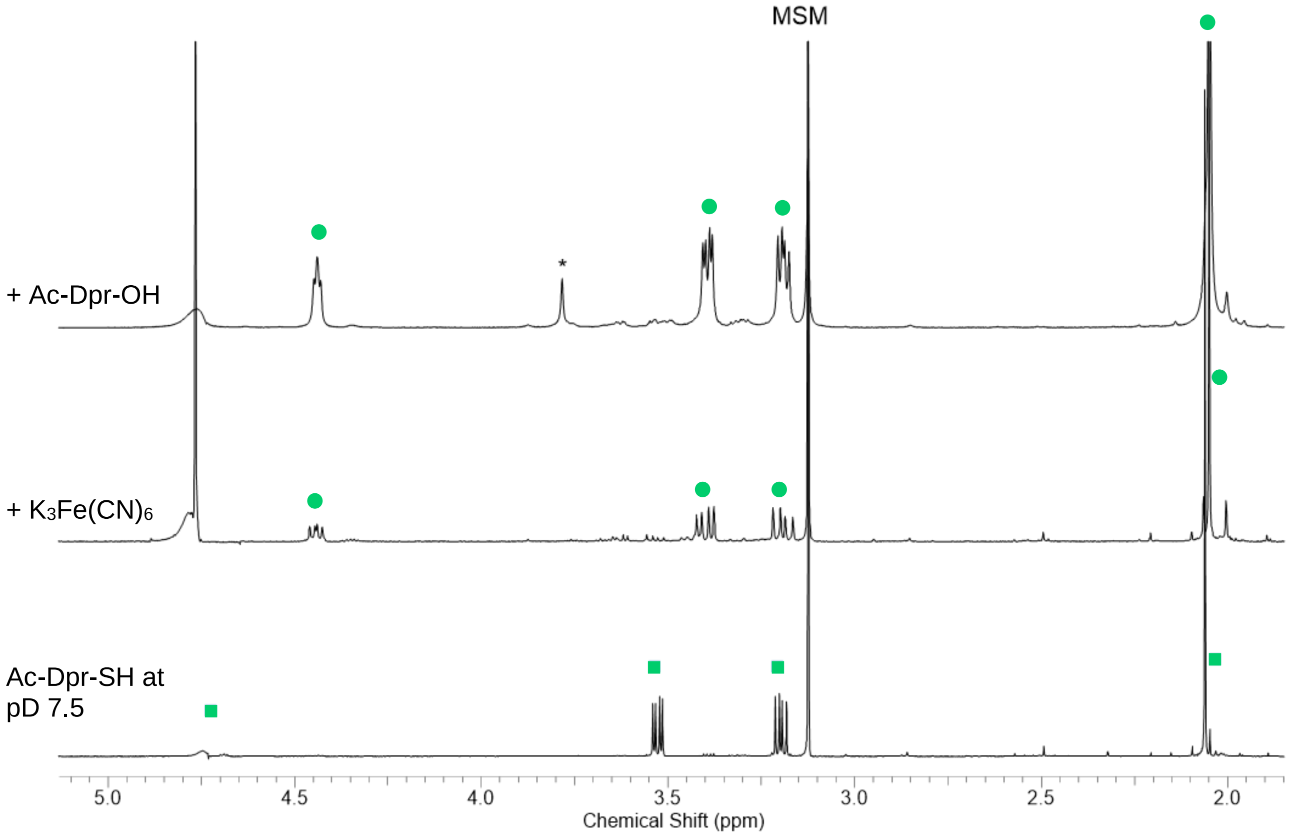

Supplementary Figure 27. ^{1}*H NMR (700 MHz,* D_2O*, noesygppr1d) spectra to show* ***Ac-Dpr-SH*** *(25 mM) at pD 7.5 before (bottom spectrum), after the addition of* $K_3Fe(CN)_6$ *(3 equiv., middle spectrum) and after spiking the reaction mixture with* ***Ac-Dpr-OH*** *(2 equiv.). *Residual dioxane from the preparative synthesis of* ***Ac-Dpr-OH****.*

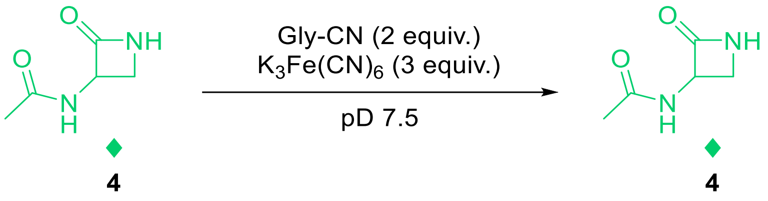

4 (1.10 mg, 8.60 µmol), **Gly-CN**·HCl (1.59 mg, 17 µmol) and MSM (1.40 µL of 1 M D_2O solution) in D_2O (110 µL) was adjusted to pD 7.5 with 2 M NaOD (4.00 µL). $K_3Fe(CN)_6$ (8.50 mg, 26.0 µmol) was added, and the reaction mixture stirred vigorously at room temperature for 30 min. The reaction mixture was diluted with D_2O (400 µL), centrifuged, the supernatant readjusted to pD 7.5 and analysed by 1D and 2D NMR spectroscopy.

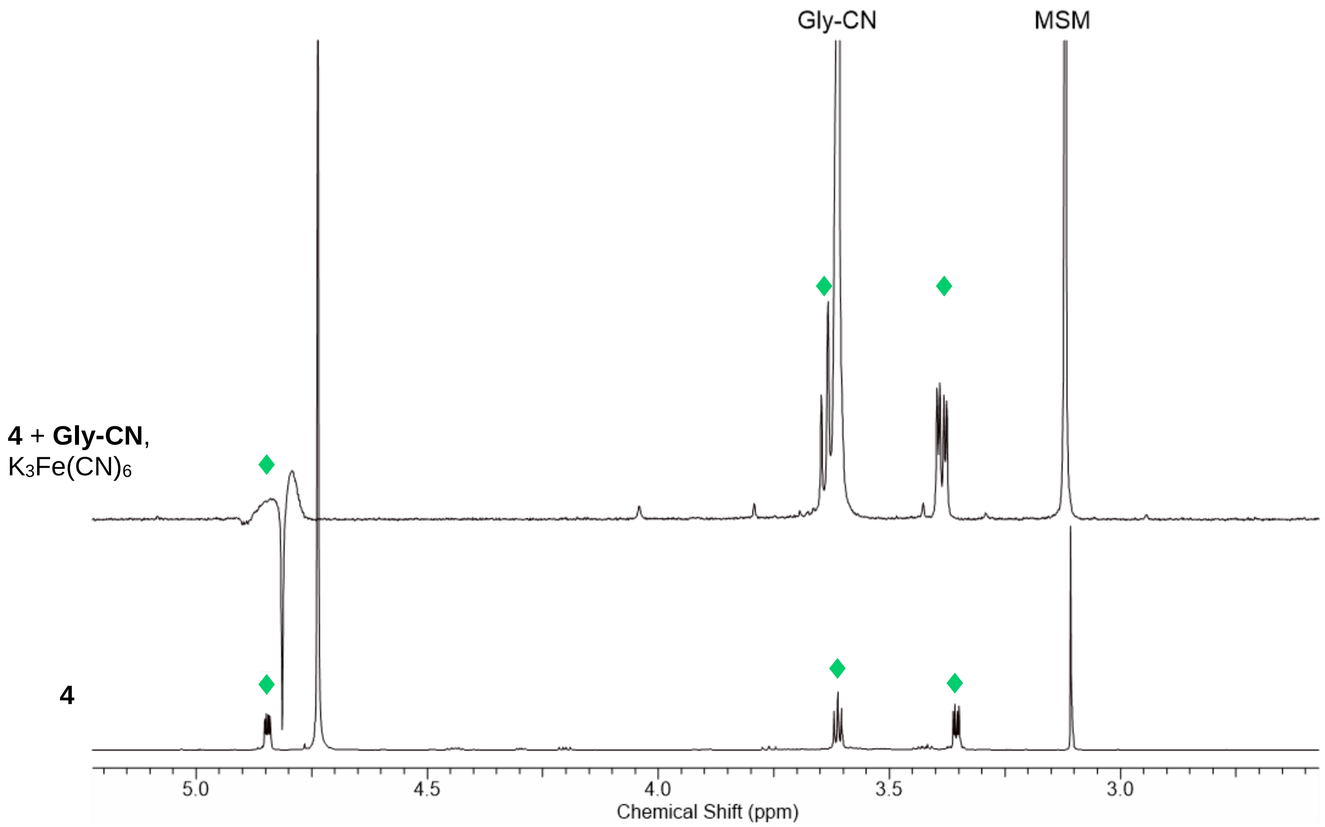

*Supplementary Figure 28. ^{1}H NMR (700 MHz, D_2O) spectra to show **4** in D_2O (bottom) and ^{1}H NMR (700 MHz, D_2O, noesygppr1d) to show the reaction of **4** (75 mM) with **Gly-CN** (2 equiv.) and $K_3Fe(CN)_6$ (3 equiv.) at pD 7.5 (top spectrum).*

Prebiotic couplings of Ac-Dpr-Gly-SH and Gly-CN

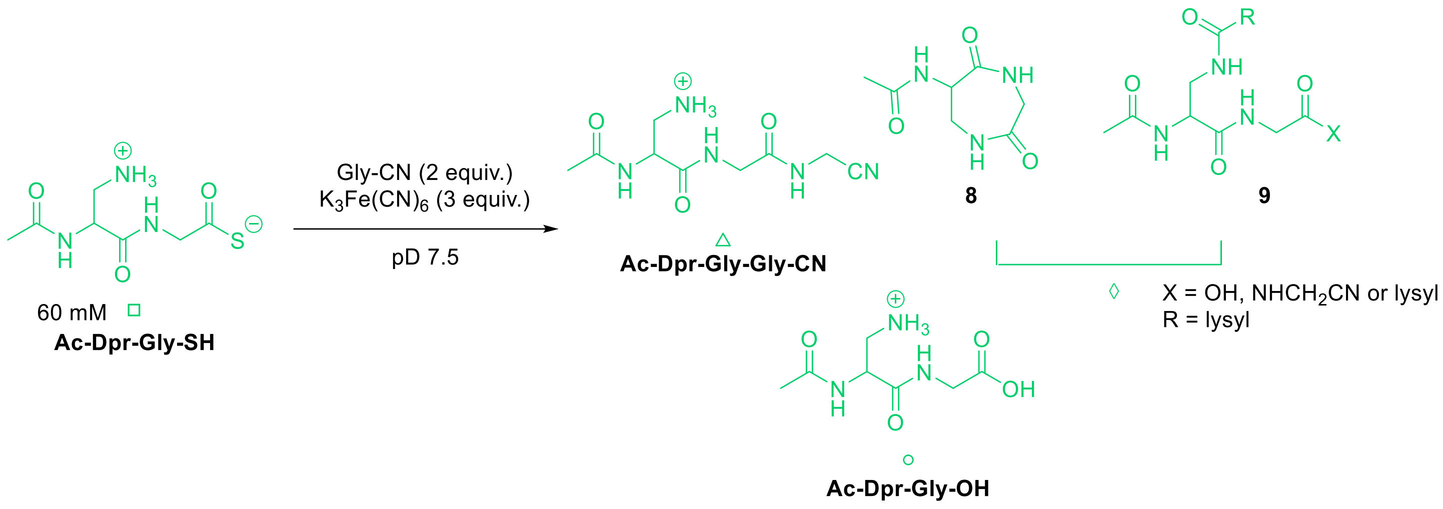

Reaction carried out *via* general procedure **A** using **Ac-Dpr-Gly-SH** to afford **Ac-Dpr-Gly-Gly-CN** (74%) and **Ac-Dpr-Gly-OH** (15%) and side-chain amidation products **8**, **9** (< 10%).

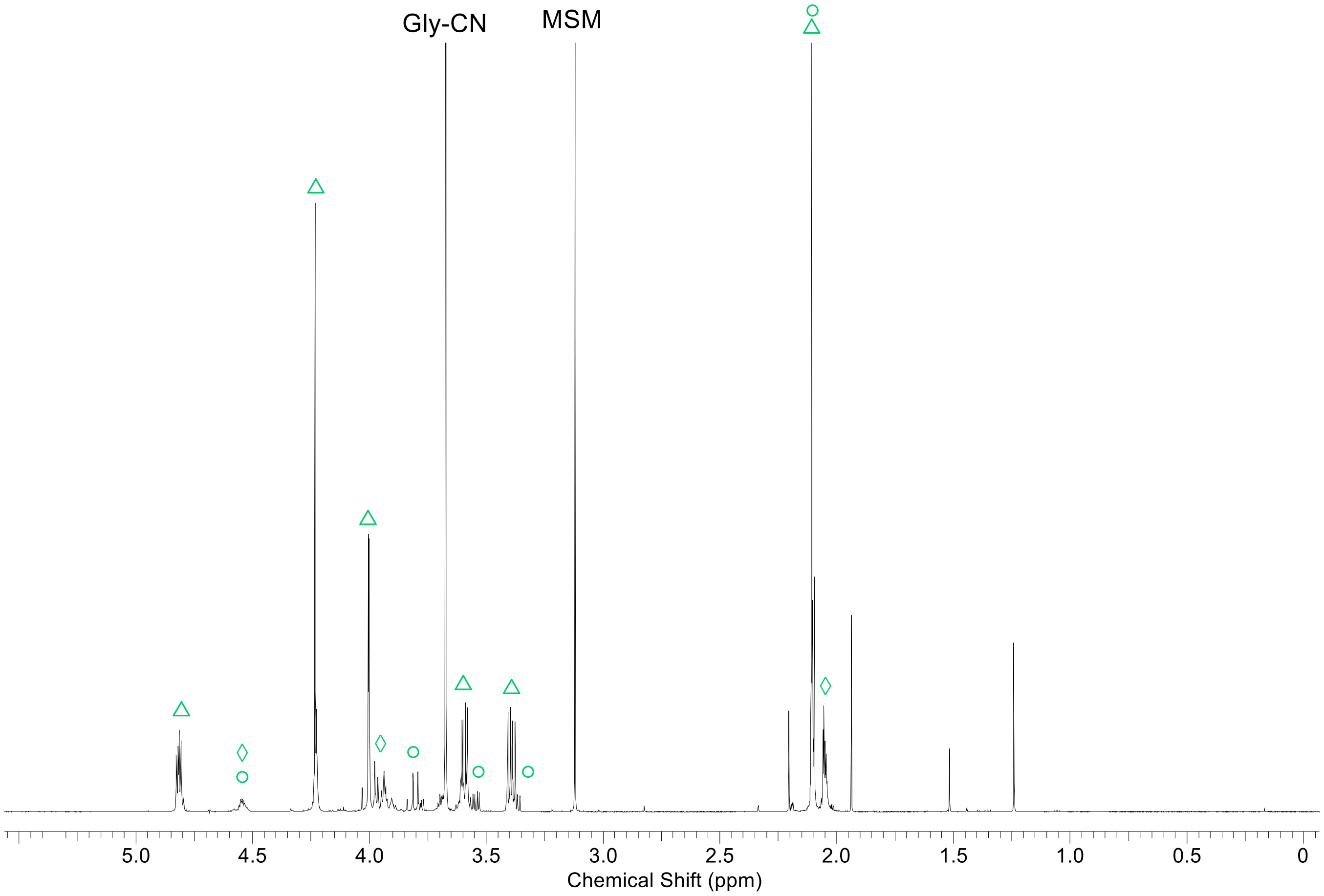

Supplementary Figure 29. [1]*H NMR (700 MHz, D_2O, 0.0 – 5.5 ppm) spectrum showing the reaction of* ***Ac-Dpr-Gly-SH*** *(60 mM),* ***Gly-CN*** *(2 equiv.) and $K_3Fe(CN)_6$ (6 equiv.) at pD 7.5.*

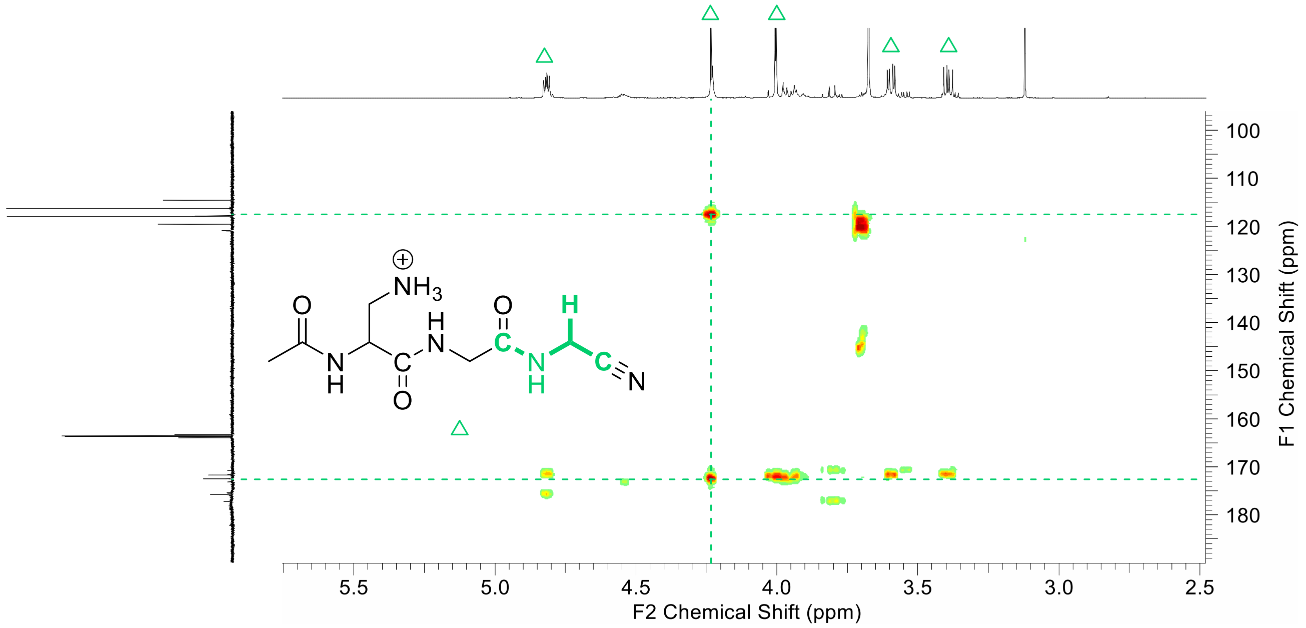

*Supplementary Figure 30. ^1H-^{13}C HMBC (^1H-700 MHz [2.5–5.7 ppm], ^{13}C-176 MHz [15–185 ppm], D_2O) spectrum showing the $^2J_{CH}$ and $^3J_{CH}$ couplings of Dpr-Gly-Gly-(C2)–H_2 at 4.23 ppm of **Ac-Dpr-Gly-Gly-CN** to C=O resonance at 172.5 ppm and CN resonance at 117.8 ppm, which is diagnostic for peptide formation.*

Ac-Dpr-Gly-Gly-CN (△): **^{1}H NMR** (700 MHz, D_2O) δ_H 4.81 (ABX, *J* = 8.4, 5.2 Hz, 1H, Dpr-(C2)–H), 4.23 (s, 2H, Dpr-Gly-Gly-(C2)–H_2), 4.01 (AB, *J* = 17.1 Hz, 1H, Dpr-Gly-(C2)–H), 4.00 (AB, *J* = 17.1 Hz, 1H, Dpr-Gly-(C2)–H′), 3.58 (ABX, *J* = 13.5, 5.2 Hz, 1H, Dpr-(C3)–H), 3.38 (ABX, *J* = 13.5, 8.4 Hz, 1H, Dpr-(C3)–H′), 2.11 (s, 3H, $COCH_3$).**^{13}C NMR** (176 MHz, D_2O, partial assignment) δ_C 175.8 (**C**OCH_3), 172.4 (Gly_1-C1), 171.7 (Dpr-C1), 117.8 (Gly_2-C1), 51.6 (Dpr-C2), 43.3 (Gly_2-C2), 40.8 (Dpr-C3). **HRMS-ESI** $[M+H]^+$ calc. for $C_9H_{16}N_5O_3^+$: 242.1248; obs. 242.1244.

Ac-Dpr-Gly-OH (○): **^{1}H NMR** (700 MHz, D_2O, partial assignment) δ_H 3.79 (AB, *J* = 17.2 Hz, 1H, Gly-(C2)–H), 3.76 (AB, *J* = 17.2 Hz, 1H, Gly-(C2)–H′), 3.50 (ABX, *J* = 13.4, 5.6 Hz, 1H, Dpr-(C3)–H), 3.32 (ABX, *J* = 13.4, 7.6 Hz, 1H, Dpr-(C3)–H′). **HRMS-ESI** $[M+H]^+$ calc. for $C_7H_{14}N_3O_4^+$: 204.0979; obs. 204.0979.

8 is tentatively assigned as the major product of side-chain amidation. Signals corresponding to **8** and **9** increased in intensity under alkaline conditions where no **Gly-CN** was present (See Supplementary Figure 31)

8: **HRMS-ESI** $[M+H]^+$ calc. for $C_7H_{12}N_3O_3^+$: 186.0873; obs. 186.0873.

9: **HRMS-ESI** $[M+H]^+$ calc. for $C_{14}H_{25}N_6O_7^+$: 389.1779; obs. 389.1769.

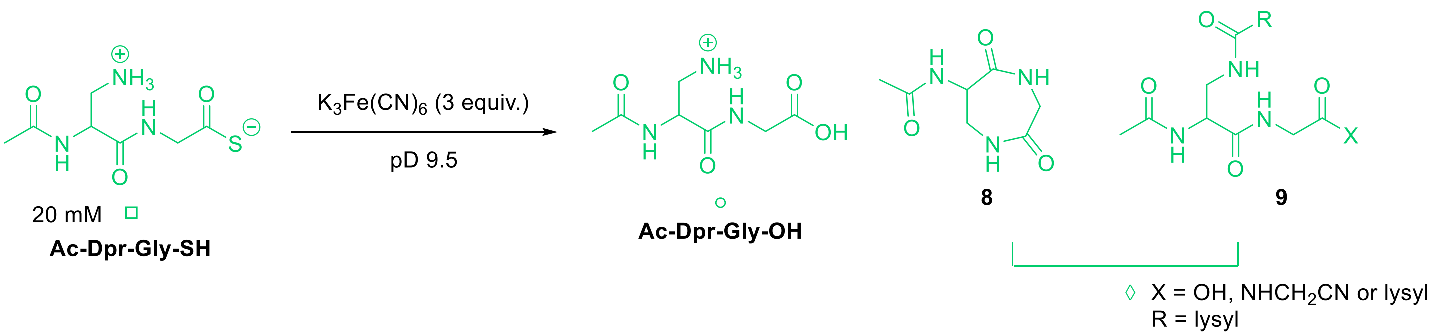

Reaction carried out using **Ac-Dpr-Gly-SH** (20 mM) and $K_3Fe(CN)_6$ (3 equiv.) at pD 9.5. The reaction mixture was stirred at room temperature for 30 min and then centrifuged. The supernatant was analysed by 1D and 2D NMR spectroscopy, yielding **Ac-Dpr-Gly-OH** (37%) and side-chain amidation products **8**, **9** (60%).

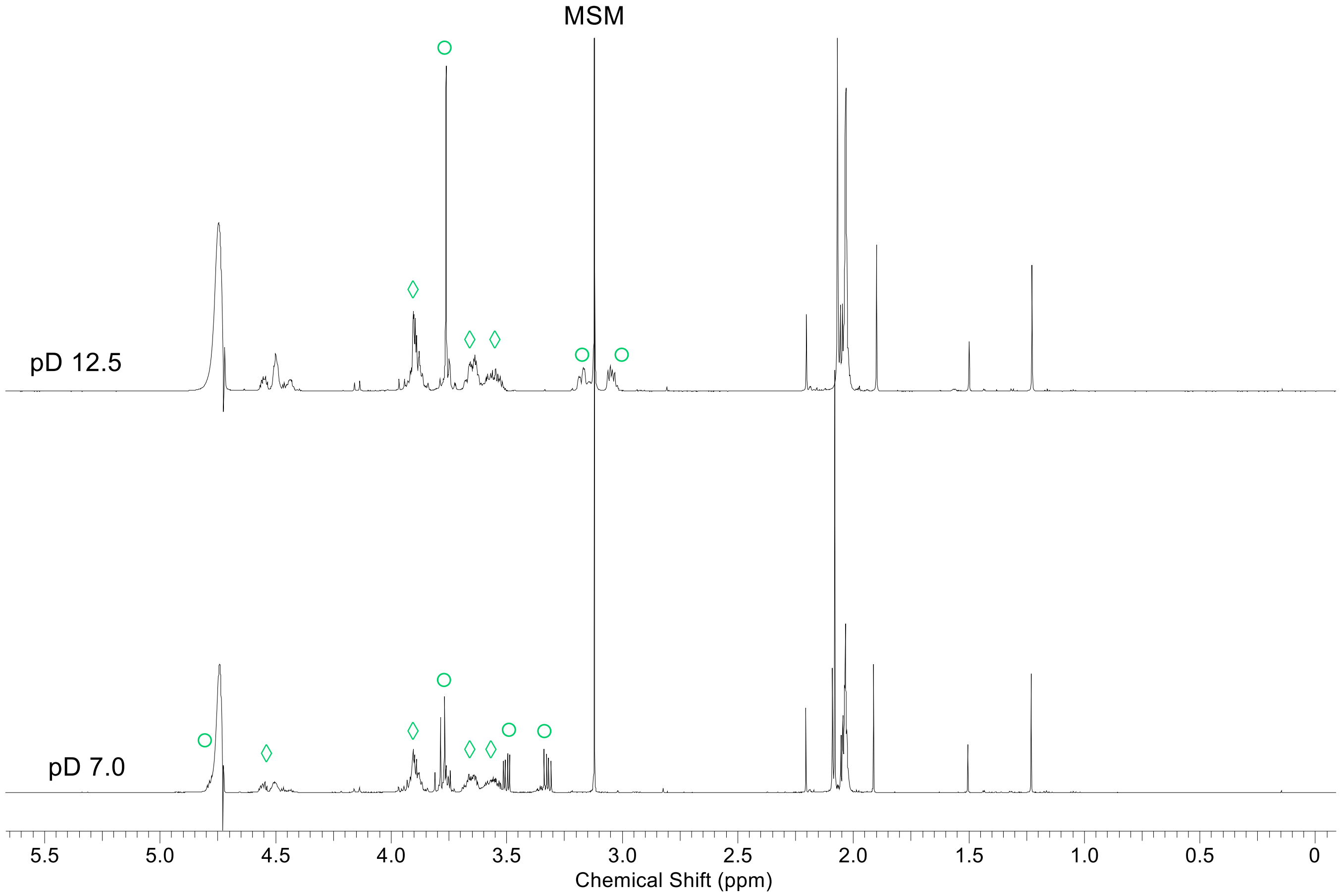

*Supplementary Figure 31. ^1H NMR (700 MHz, D_2O, 0.0 – 5.5 ppm, noesygppr1d) spectra showing the reaction products of **Ac-Dpr-Gly-SH** (20 mM) and $K_3Fe(CN)_6$ (3 equiv.) initiated at pD 9.5, displayed at pD 7 (bottom spectrum) and pD 12.5 (top spectrum). The upfield shift in **Ac-Dpr-Gly-OH**'s β-CH_2NH_2 indicates they are unfunctionalized, as they have passed through their pK_{aH}, whereas **8**'s β-CH_2NH_2 does not move, indicating that it is functionalised.*

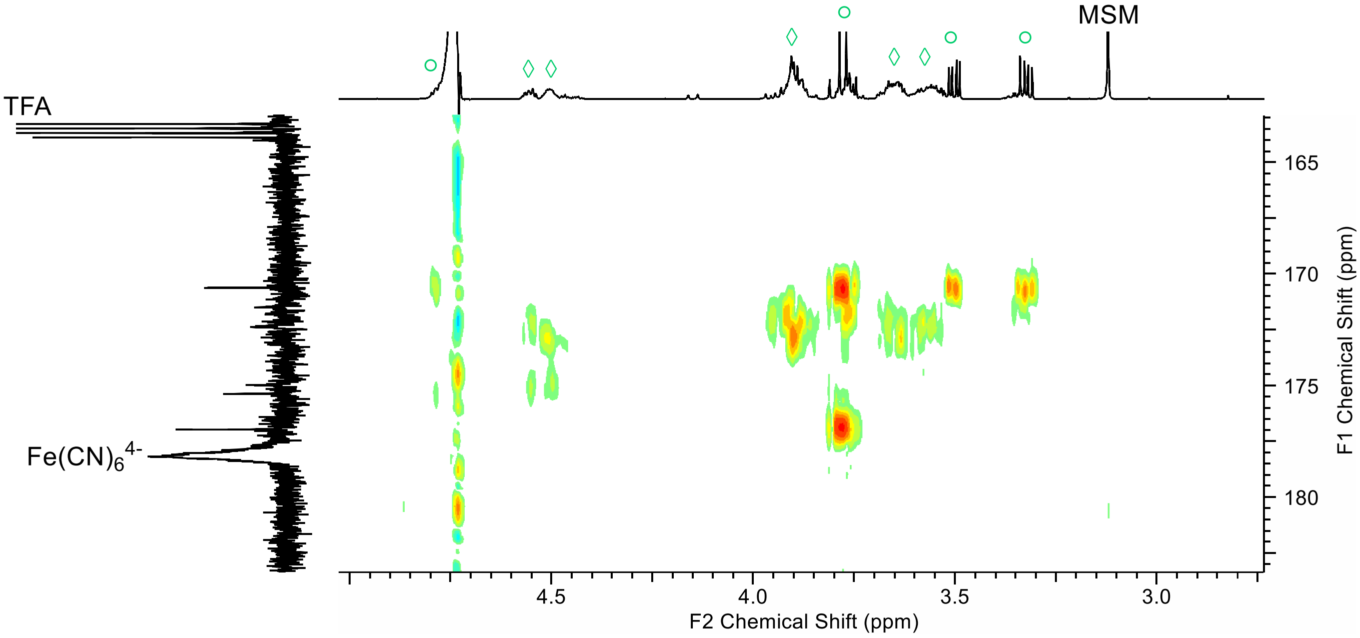

*Supplementary Figure 32. ^{1}H-^{13}C HMBC (^{1}H-700 MHz [3.3–5.0 ppm], ^{13}C-176 MHz [165–183 ppm], D_2O) spectrum showing the reaction products of **Ac-Dpr-Gly-SH** (20 mM) and $K_3Fe(CN)_6$ (3 equiv.) initiated at pD 9.5.*

Entry	pD	Buffer	% **Ac-Dpr-Gly-Gly-CN**	% side chain amidation (**8**+**9**)	% **Ac-Dpr-Gly-OH**
1	7.0	Phosphate	58	25	10
2	7.5	None	74	15	<10
3	9.5[i]	Borate	37	60	<5

*Supplementary Table 4. ^{1}H NMR yields for the reaction of **Ac-Dpr-Gly-SH** (60 mM), **Gly-CN** (2 equiv.) and $K_3Fe(CN)_6$ (3 equiv.) at the specified pD and in the specified buffer (600 mM, D_2O).[i] **Ac-Dpr-Gly-SH** (20 mM).*

Prebiotic couplings of Ac-Lys-Gly-SH and Gly-CN

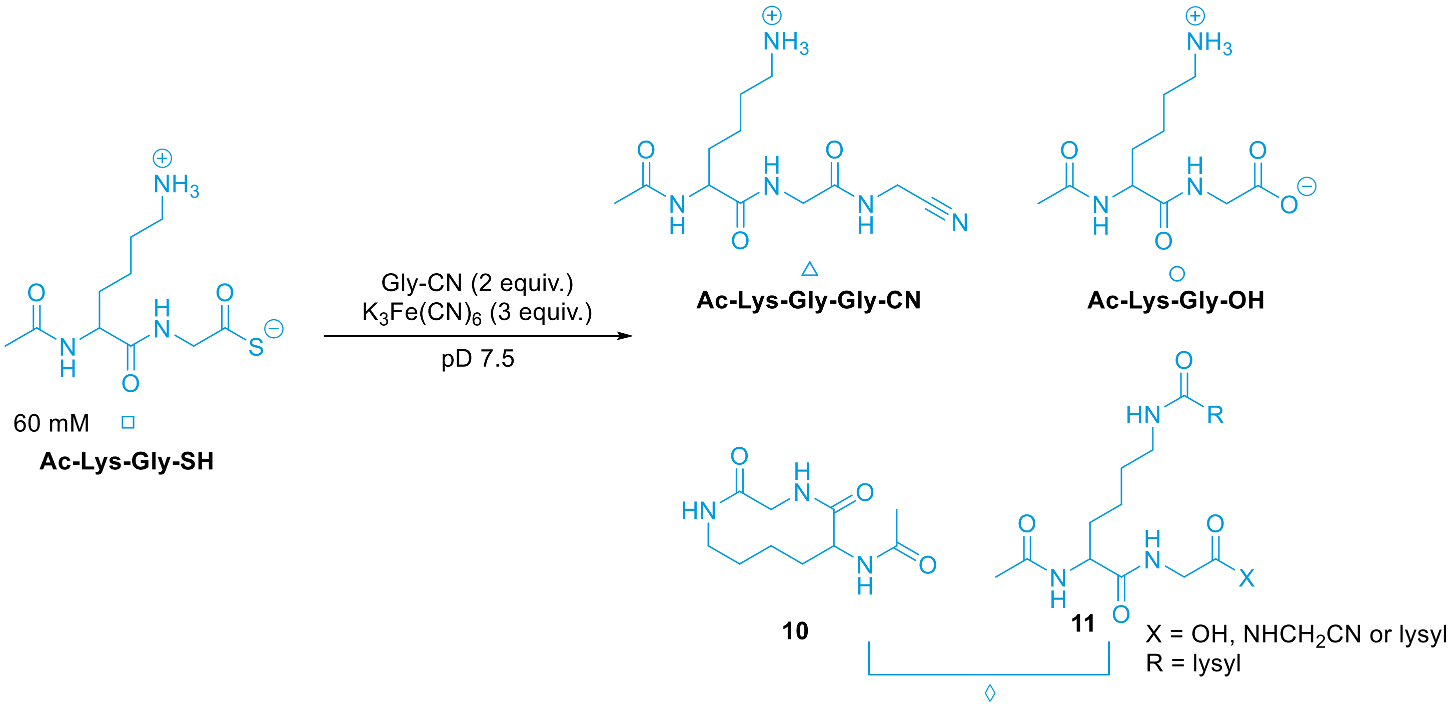

Reaction carried out *via* general procedure **A** using **Ac-Lys-Gly-SH** to afford **Ac-Lys-Gly-Gly-CN** (90%), **Ac-Lys-Gly-Gly-OH** (<5%) and side-chain amidation products **10**, **11** (8%).

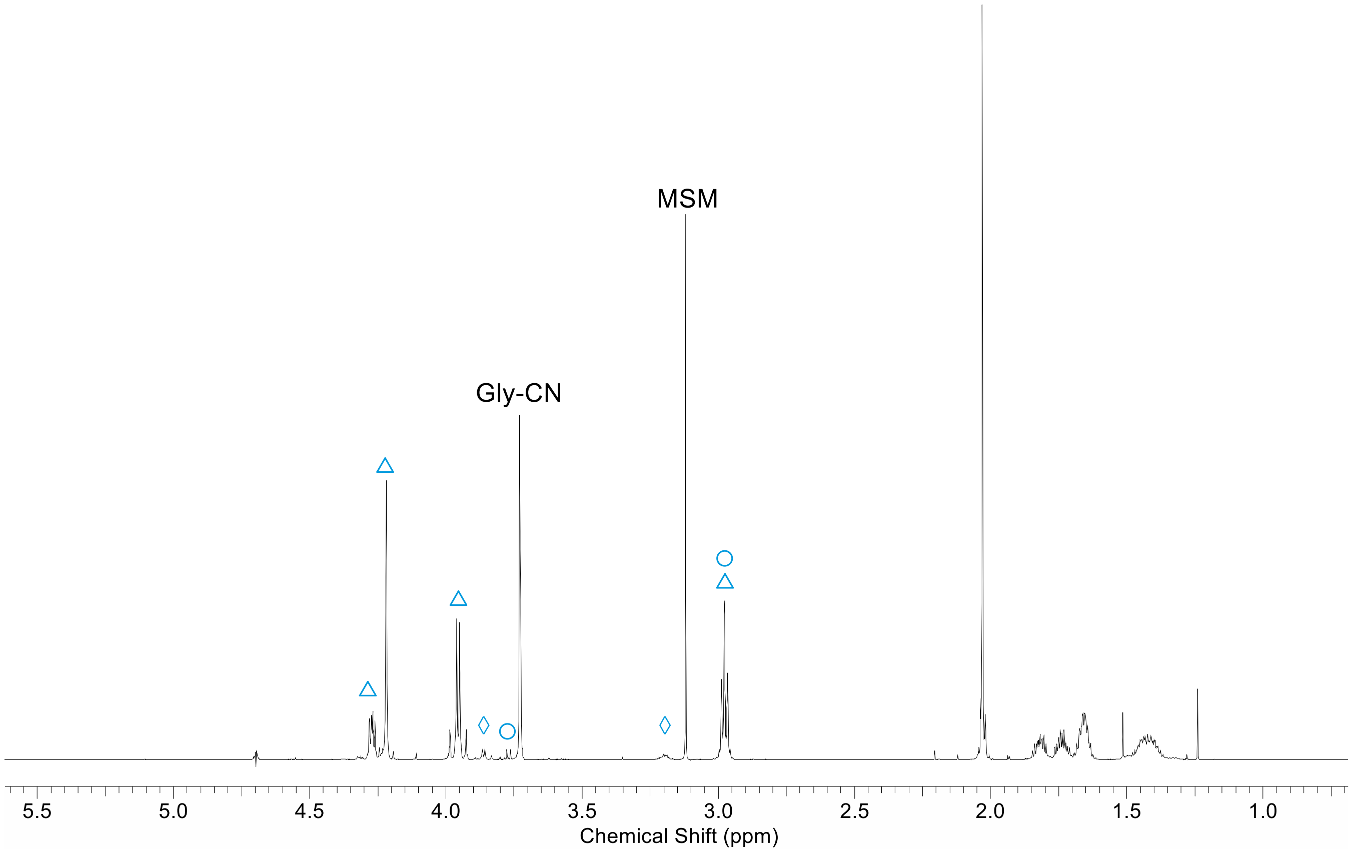

*Supplementary Figure 33. ^{1}H NMR (700 MHz, D_2O, 0.0 – 5.5 ppm, noesygppr1d) spectrum showing the reaction of **Ac-Lys-Gly-SH** (60 mM), **Gly-CN** (2 equiv.) and $K_3Fe(CN)_6$ (3 equiv.) at pD 7.5.*

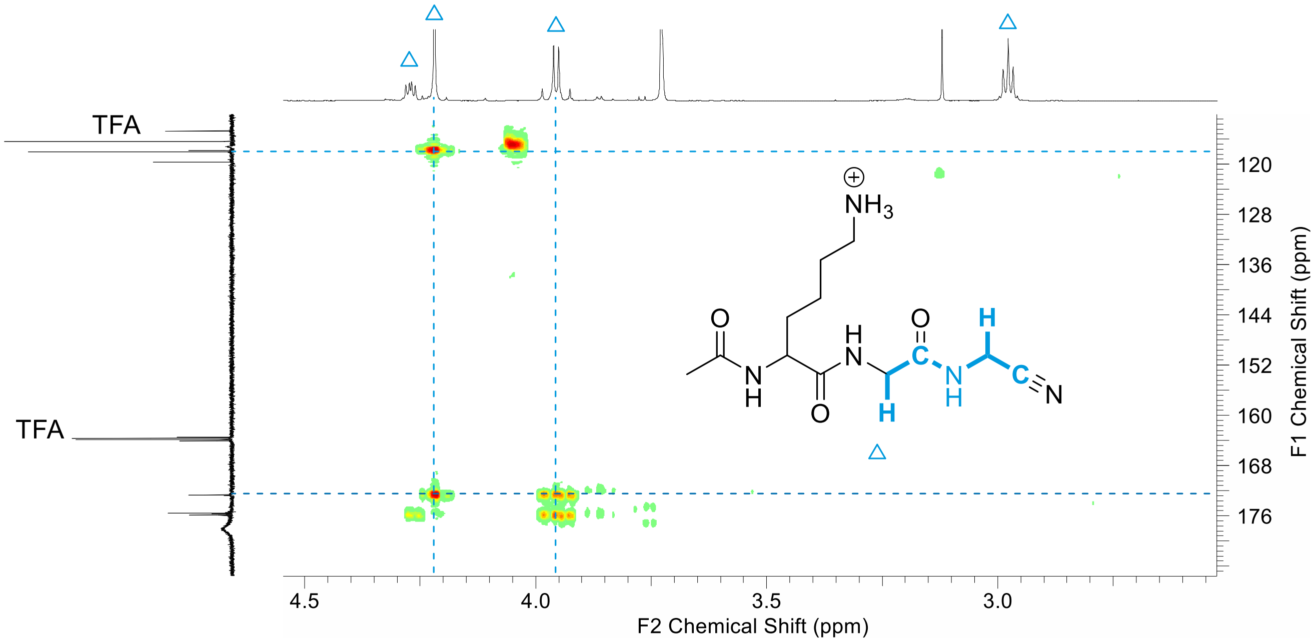

Supplementary Figure 34. ^1H-^{13}C HMBC (^1H-700 MHz [2.5–4.5 ppm], ^{13}C-176 MHz [112–185 ppm], D_2O) spectrum showing the $^2J_{CH}$ coupling of Lys-Gly-(C2)–H_2 at 3.95 of ***Ac-Lys-Gly-Gly-CN*** *to C=O resonance at 172.5 ppm and the $^2J_{CH}$ and $^3J_{CH}$ couplings of Lys-Gly-Gly-(C2)–H_2 at 4.22 to the same C=O resonance at 172.5 ppm and a CN resonance at 117.9 ppm.* ***Gly-CN*** *^1H resonance is misaligned with ^{13}C correlation due to fluctuation in pH between ^1H and HMBC acquisitions. The Gly-(C2)–H_2 ^1H resonance is sensitive to pH changes due to the variable protonation state of Gly-CN amine moiety.*

Ac-Lys-Gly-Gly-CN (△): **^{1}H NMR** (700 MHz, D_2O) δ_H 4.27 (dd, J = 8.7, 5.4 Hz, 1H, Lys-(C2)–H), 4.22 (s, 2H, Gly$_1$-(C2)–H_2), 3.97 (AB, J = 17.1 Hz, 1H, Gly$_2$-(C2)–H), 3.94 (AB, J = 17.1 Hz, 1H, Gly$_2$-(C2)–H'), 2.98 (app t, J = 7.6 Hz, 2H, Lys-(C6)–H_2), 2.03 (s, 3H, $COCH_3$), 1.80-1.85 (m, 1H, Lys-(C3)–H), 1.71-1.77 (m, 1H, Lys-(C3)–H'), 1.62-1.69 (m, 2H, Lys-(C5)–H_2), 1.37-1.50 (m, 2H, Lys-(C4)–H_2). **^{13}C NMR** (176 MHz, D_2O) δ_C 175.8 (Lys-C1), 175.5 (**C**OCH_3) 172.7 (Gly$_1$-C1), 117.8 (Gly$_2$-C1), 54.8 (Lys-C2), 43.3 (Gly$_1$-C2), 40.2 (Lys-C6), 31.0 (Lys-C3), 28.4 (Gly$_2$-C2), 27.0 (Lys-C5), 22.8 (Lys-C4/CO**C**H_3), 22.6 (Lys-C4/CO**C**H_3). **HRMS-ESI** $[M+H]^+$ calc. for $C_{12}H_{22}N_5O_3^+$ 284.1717; obs 284.1713.

Ac-Lys-Gly-Gly-OH (○): **^{1}H NMR** (700 MHz, D_2O, partial assignment) δ_H 4.31 (obs. dd, 1H, Lys-(C2)–H), 3.79 (AB, J = 17.2 Hz, 1H, Gly-(C2)–H), 3.75 (obs. AB, 1H, Gly-(C2)–H').

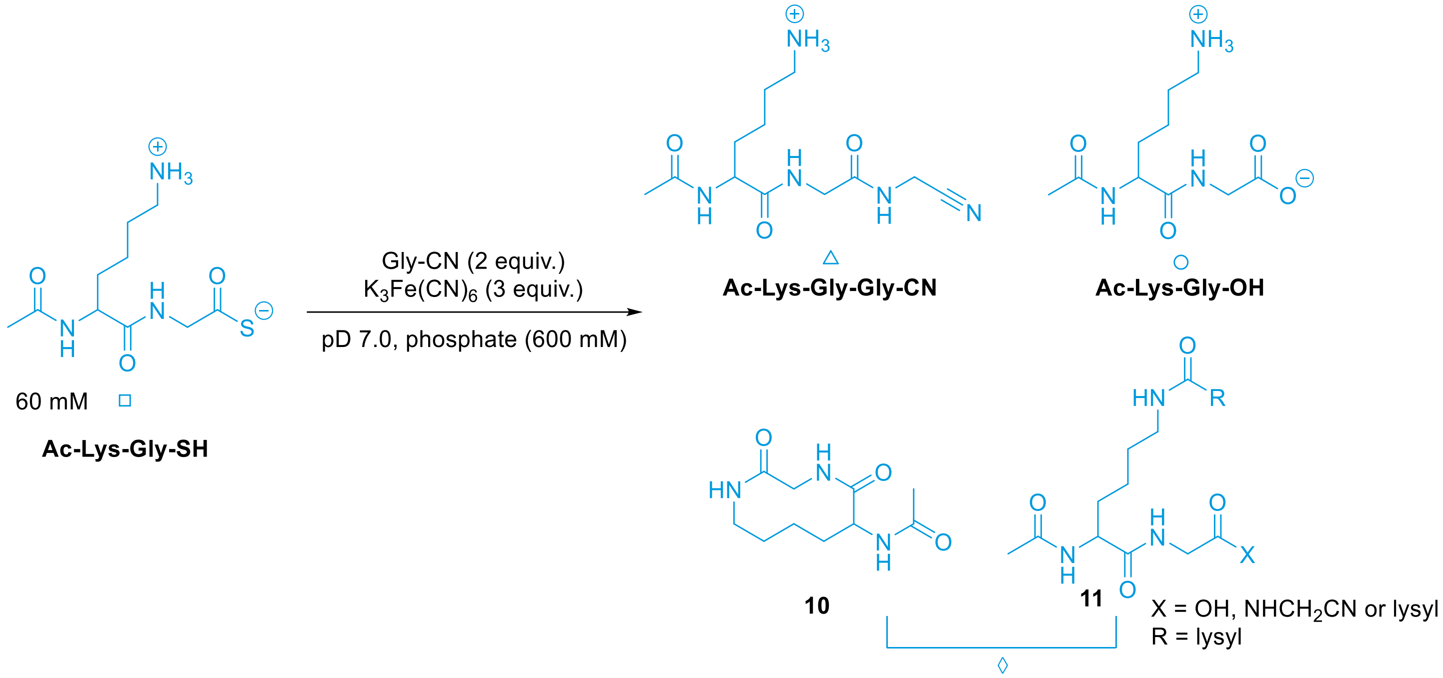

Reaction carried out *via* general procedure **B** using **Ac-Lys-Gly-SH** to afford **Ac-Lys-Gly-Gly-CN** (85%), **Ac-Lys-Gly-Gly-OH** (10%) and side-chain amidation products **10**, **11** (<10%).

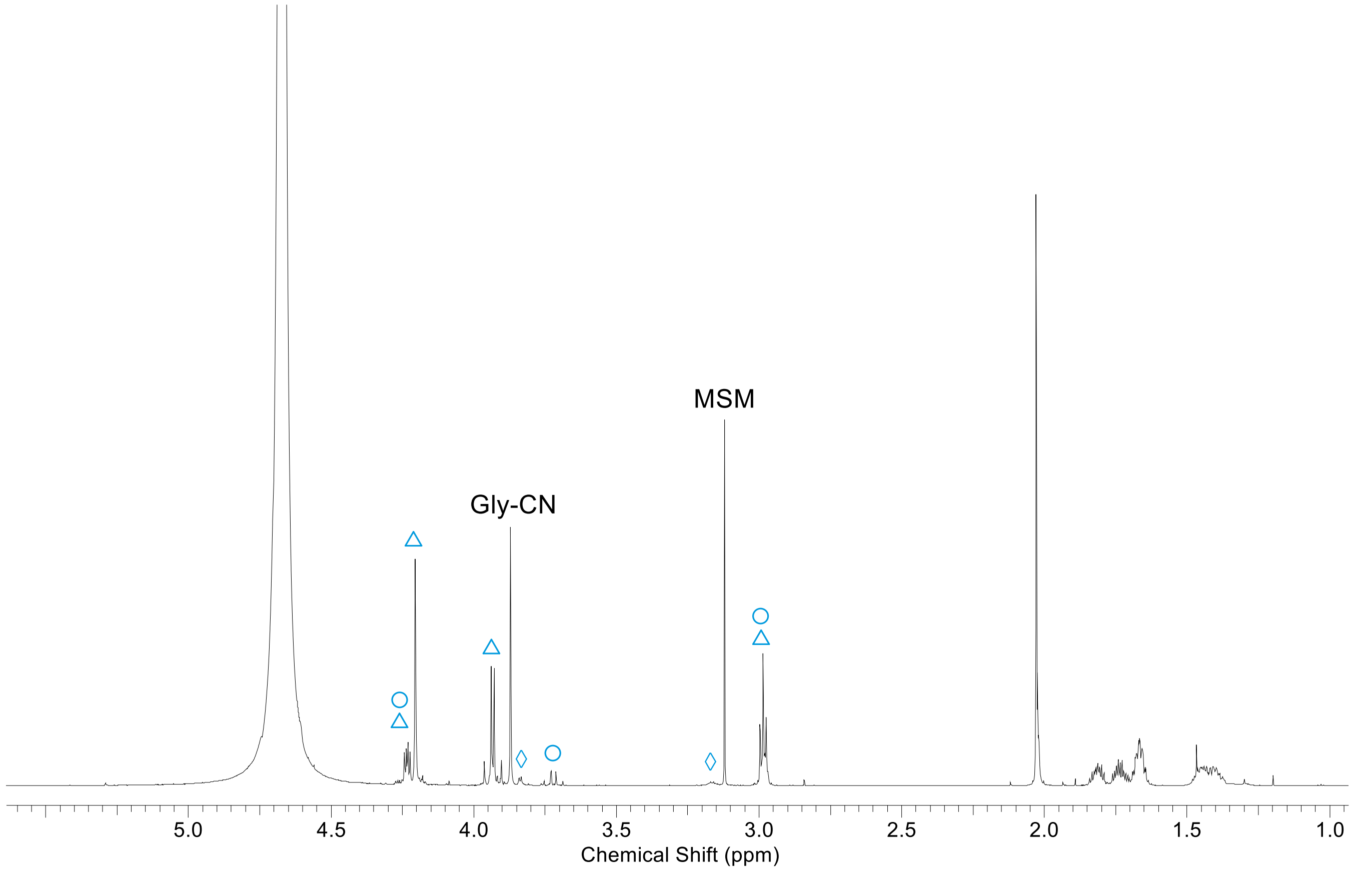

*Supplementary Figure 35. ^{1}H NMR (700 MHz, D_2O, 0.0 – 5.5 ppm, noesygppr1d) spectrum showing the reaction of **Ac-Lys-Gly-SH** (60 mM), **Gly-CN** (2 equiv.) and $K_3Fe(CN)_6$ (3 equiv.) buffered at pD 7.0 with phosphate (600 mM).*

10 is tentatively assigned as the major product of side-chain amidation. Signals corresponding to **10** and **11** were observed to increase in intensity under alkaline (pD 9.5) conditions of ligation (See Supplementary Figure 36). **10**: **^{1}H NMR** (700 MHz, D_2O, partial assignment) δ_H 3.88 (AB, J = 17.3 Hz, 1H, Gly-(C2)–H), 3.85 (AB, J = 17.3 Hz, 1H, Gly-(C2)–H'), 3.16-3.23 (m, 2H, Lys-(C6)–H_2). **HRMS-ESI** $[M+H]^+$ calc. for $C_{10}H_{18}N_3O_3^+$ 228.1343; obs 228.1337.

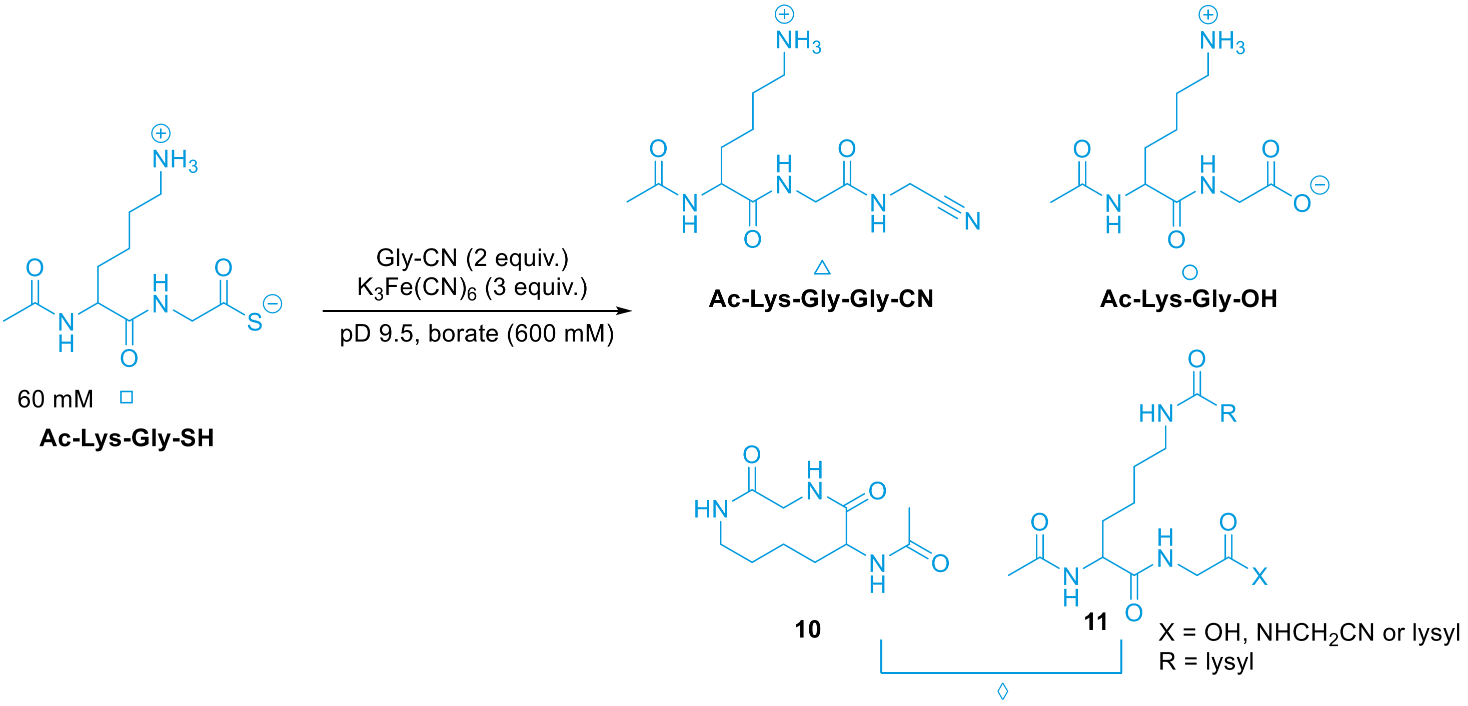

Reaction carried out using **Ac-Lys-Gly-SH** (20 mM), **Gly-CN** (2 equiv.) and $K_3Fe(CN)_6$ in borate buffer (600 mM, D_2O, pD 9.5). The reaction mixture was stirred at room temperature for 30 min and then centrifuged. The supernatant was analysed by 1D and 2D NMR spectroscopy, yielding **Ac-Lys-Gly-Gly-CN** (14%), **Ac-Lys-Gly-Gly-OH** (7%) and side-chain amidation products **10**, **11** (66%).

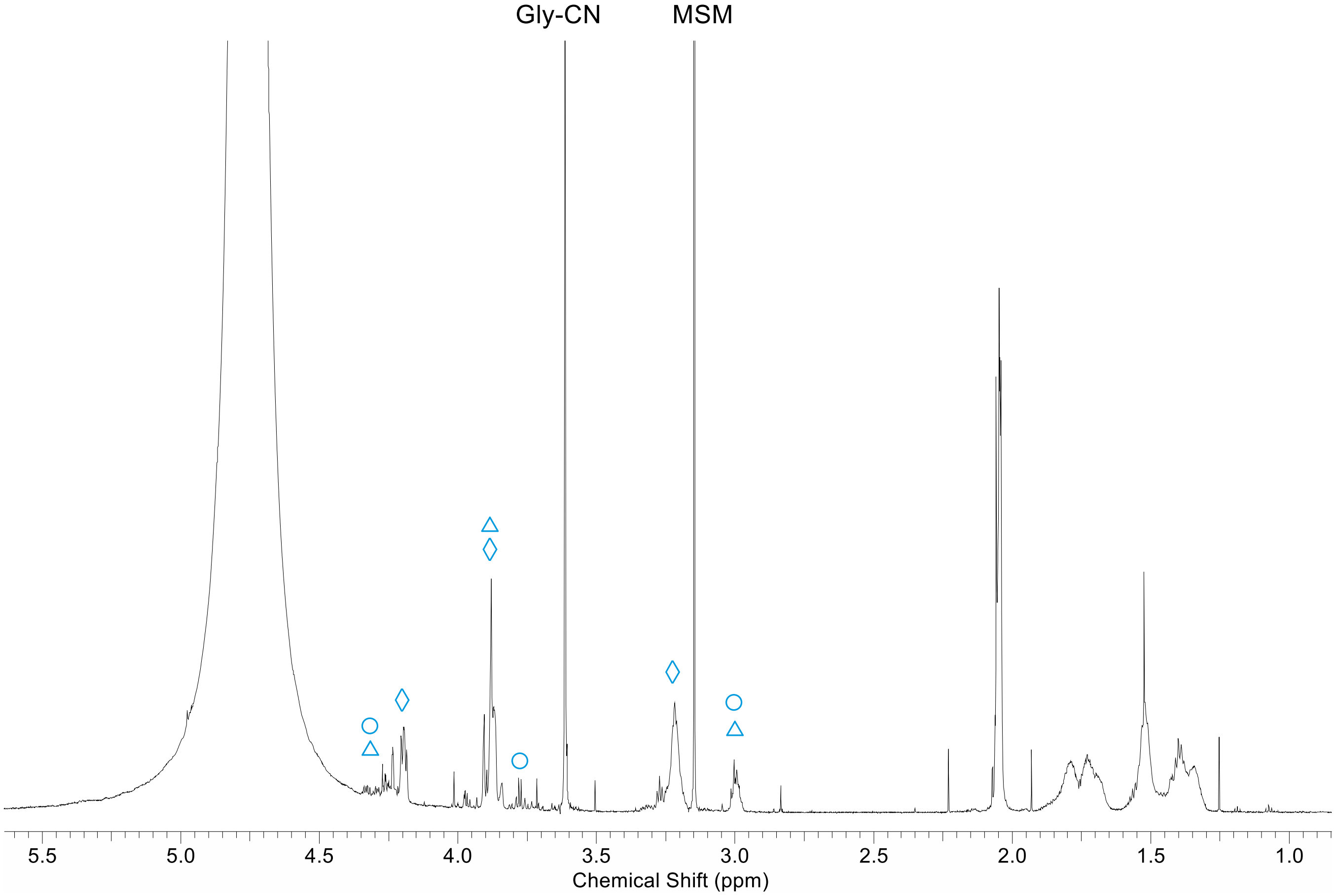

*Supplementary Figure 36. ^{1}H NMR (700 MHz, D_2O, 1.0 – 5.5 ppm) spectrum showing the reaction of **Ac-Lys-Gly-SH** (60 mM), **Gly-CN** (2 equiv.) and $K_3Fe(CN)_6$ (3 equiv.) buffered at pD 9.5 with borate (600 mM).*

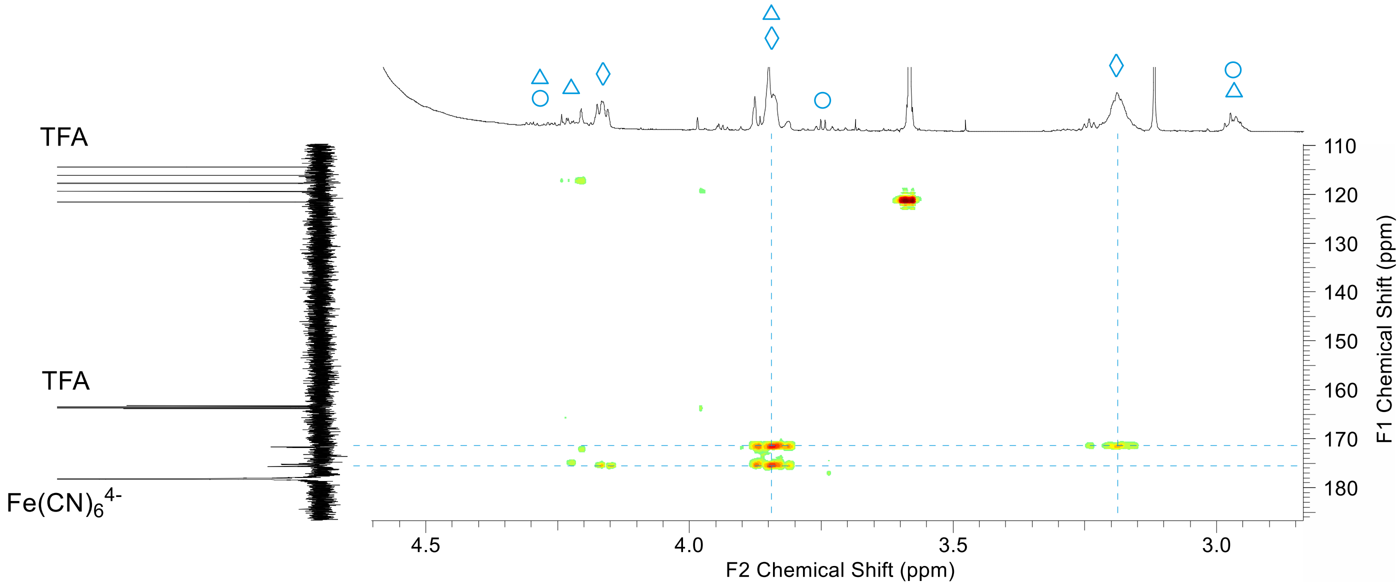

Supplementary Figure 37. ^{1}H-^{13}C HMBC (^{1}H-700 MHz [2.9–4.6 ppm], ^{13}C-176 MHz [110–185 ppm], D_2O) spectrum showing the $^{2}J_{CH}$ and $^{3}J_{CH}$ couplings of Lys-Gly-(C2)–H_2 at 3.85 of side-chain amidation products to C=O resonances at 171.6 ppm and 175.5 and the $^{3}J_{CH}$ coupling of Lys-(C6)–H_2 at 3.19 to a C=O resonance at 171.6 ppm.

Entry	pD	Buffer	% **Ac-Lys-Gly-Gly-CN**	% side chain amidation (**10**+**11**)	% **Ac-Lys-Gly-OH**
1	7.0	Phosphate	85	<10	10
2	7.5	None	90	8	< 5
3	9.5	Borate	26	64	< 5
4[i]	9.5	Borate	17	66	7

*Supplementary Table 5. ^{1}H NMR yields for the reaction of **Ac-Lys-Gly-SH** (60 mM), **Gly-CN** (2 equiv.) and $K_3Fe(CN)_6$ (3 equiv.) at the specified pD and in the specified buffer (600 mM, D_2O). [i] **Ac-Lys-Gly-SH** (20 mM), **Gly-CN** (2 equiv.) and $K_3Fe(CN)_6$ (3 equiv.) in borate buffer (600 mM, D_2O, pD 9.5).*

Competition experiments between Ac-AA[1]-SH, Ac-AA[2]-SH and Gly-CN

Stability time course of a mixture of Ac-Lys-SH and Ac-Orn-SH

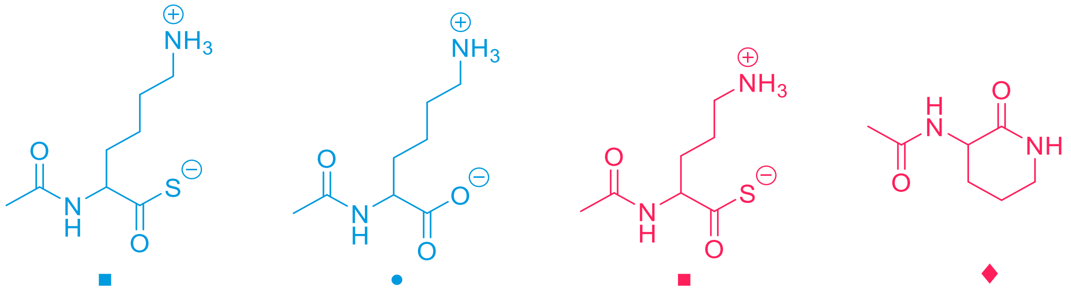

Ac-Lys-SH ■ **Ac-Lys-OH** ● **Ac-Orn-SH** ■ **2** ◆

Ac-Lys-SH (60 mM), **Ac-Orn-SH** (60 mM), **Ac-Lys-OH** (13 mM), **2** (13 mM) were observed at pD 7.5 in D_2O over 3 days. The reaction mixture was monitored by NMR spectroscopy.

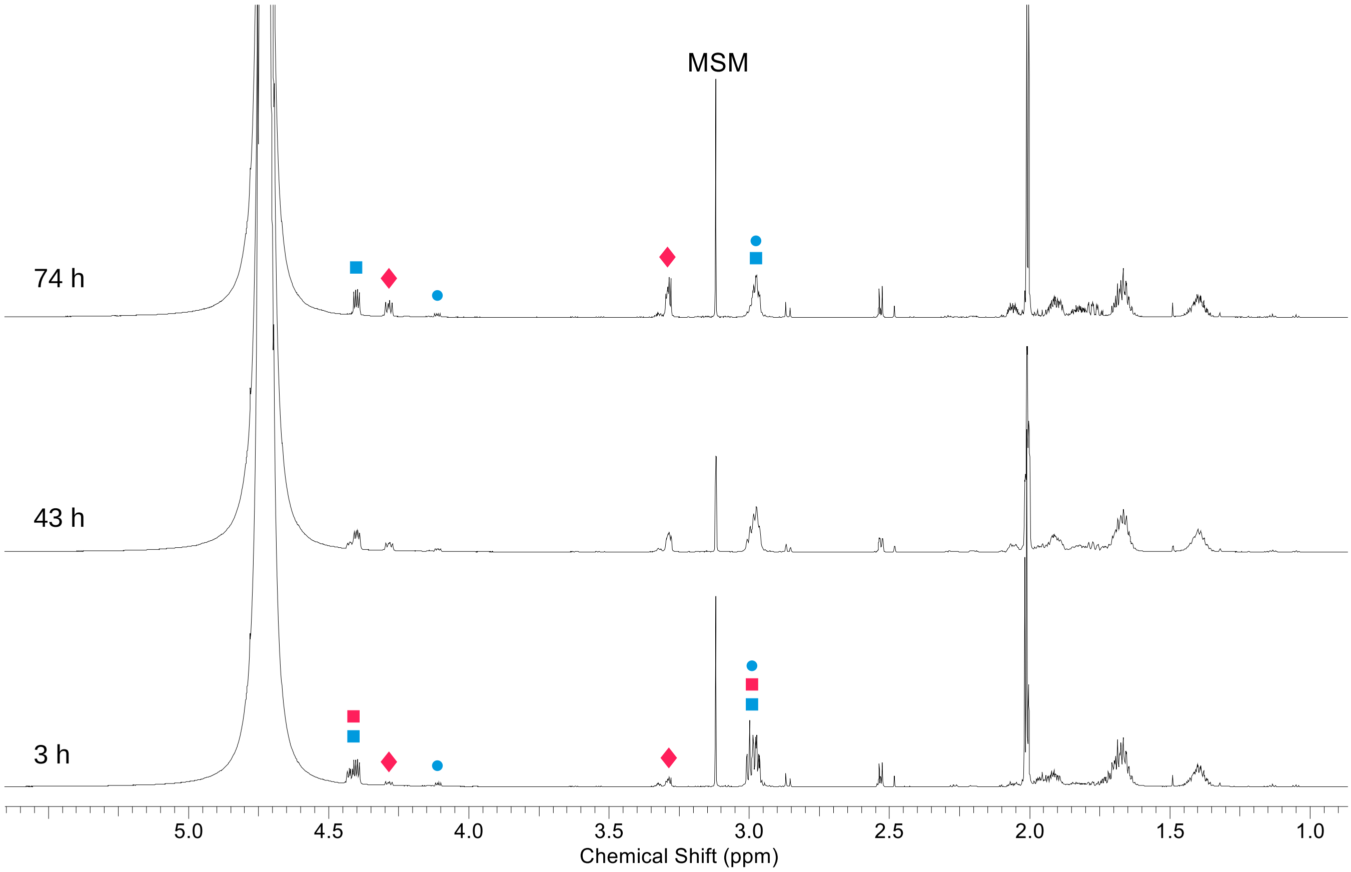

*Supplementary Figure 38. ^{1}H NMR (700 MHz, D_2O, 1.0 – 4.9 ppm) spectra showing the reaction of **Ac-Lys-SH**, **Ac-Orn-SH**, **Ac-Lys-OH** and **2** in D_2O at pD 7.5 after: 3 h (bottom spectrum), 43 h (middle spectrum) and 74 h (top spectrum).*

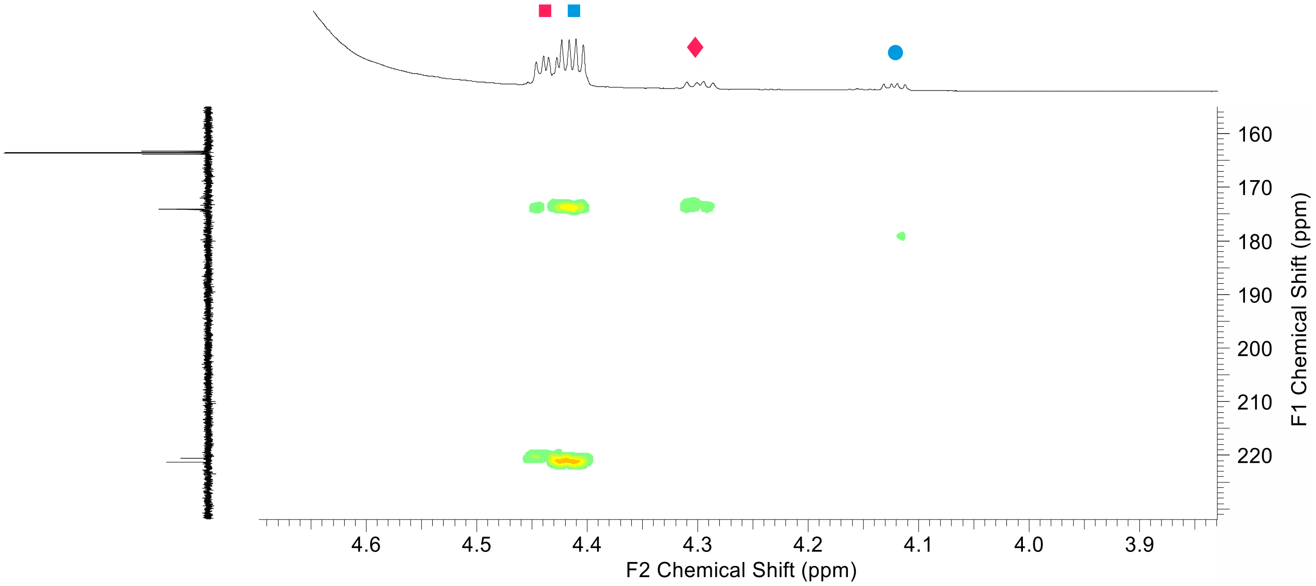

Supplementary Figure 39. *^{1}H-^{13}C HMBC (^{1}H-700 MHz [3.83–4.69 ppm], ^{13}C-176 MHz [55–230 ppm], D_2O) spectrum showing the $^{2}J_{CH}$ couplings of (C2)–H at 4.43 ppm of **Ac-Lys-SH** and **Ac-Orn-SH** to C=O resonances at 221.3 ppm and 220.6 ppm, diagnostic of thioacid formation and the $^{2}J_{CH}$ couplings of (C2)–H of **Ac-Lys-OH** and **2** to more upfield C=O resonances.*

Prebiotic coupling of Ac-Lys-SH, Ac-Orn-SH and Gly-CN

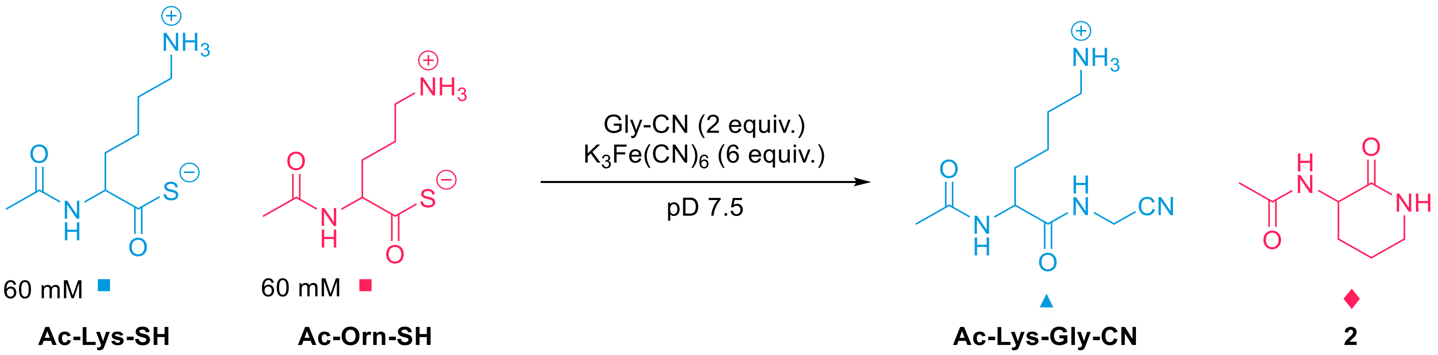

Reaction carried out using **Ac-Lys-SH** (60 mM), **Ac-Orn-SH** (60 mM), **Gly-CN** (2 equiv.) and $K_3Fe(CN)_6$ (6 equiv.) at pD 7.5. The reaction mixture was stirred at room temperature for 30 min and then centrifuged. The supernatant was analysed by 1D and 2D NMR spectroscopy, yielding **Ac-Lys-Gly-CN** (>95%) and **2** (93%).

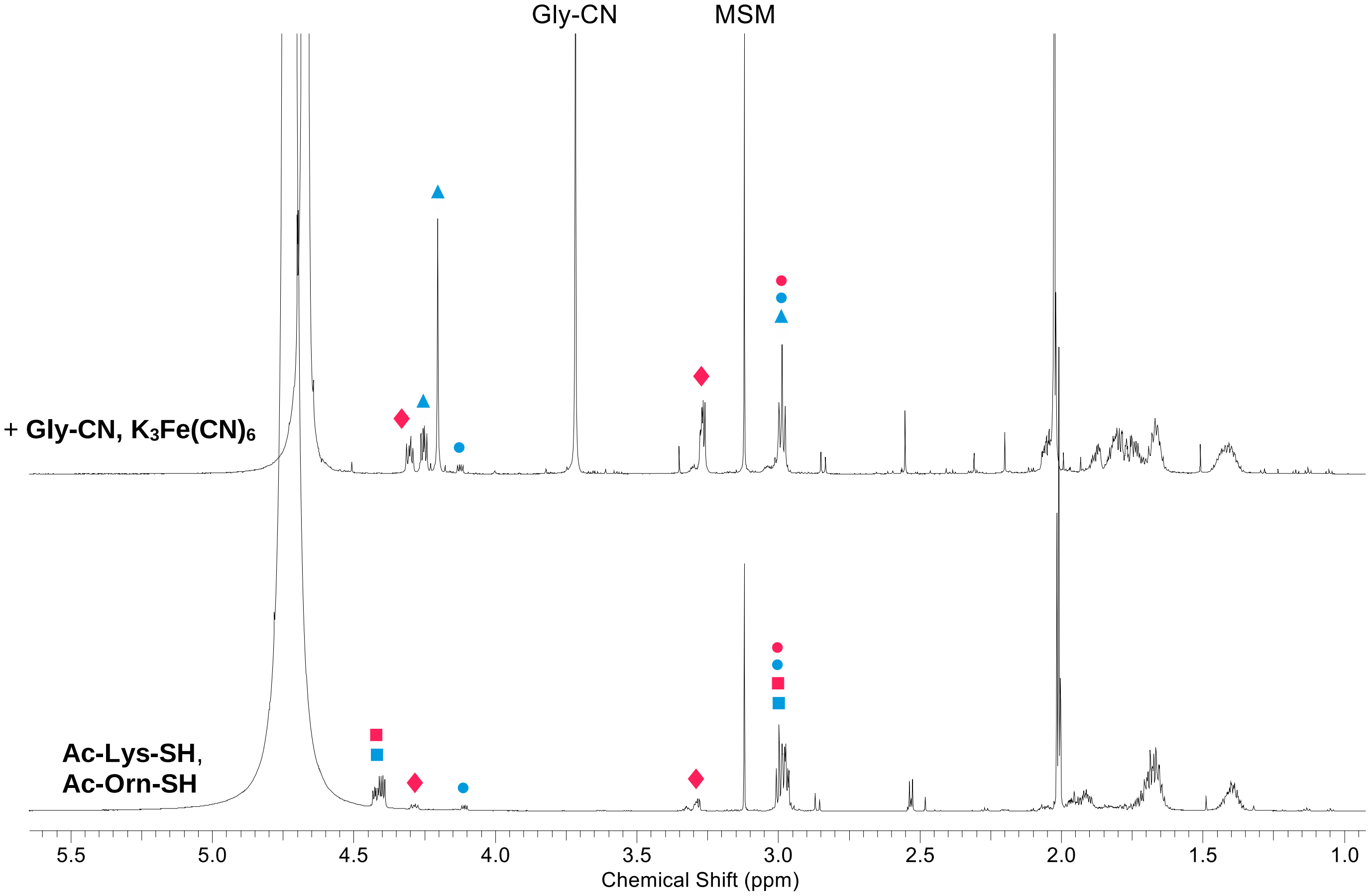

*Supplementary Figure 40. ^{1}H NMR (700 MHz, D_2O, 1.0 – 5.5 ppm) spectra **Ac-Lys-SH** (60 mM), **Ac-Orn-SH** (1 equiv.), **Ac-Lys-OH** (0.1 equiv.), **2** (0.1 equiv.) before (bottom spectrum) and after (top spectrum) the addition of **Gly-CN** (2 equiv.) and $K_3Fe(CN)_6$ (6 equiv.) at pD 7.5.*

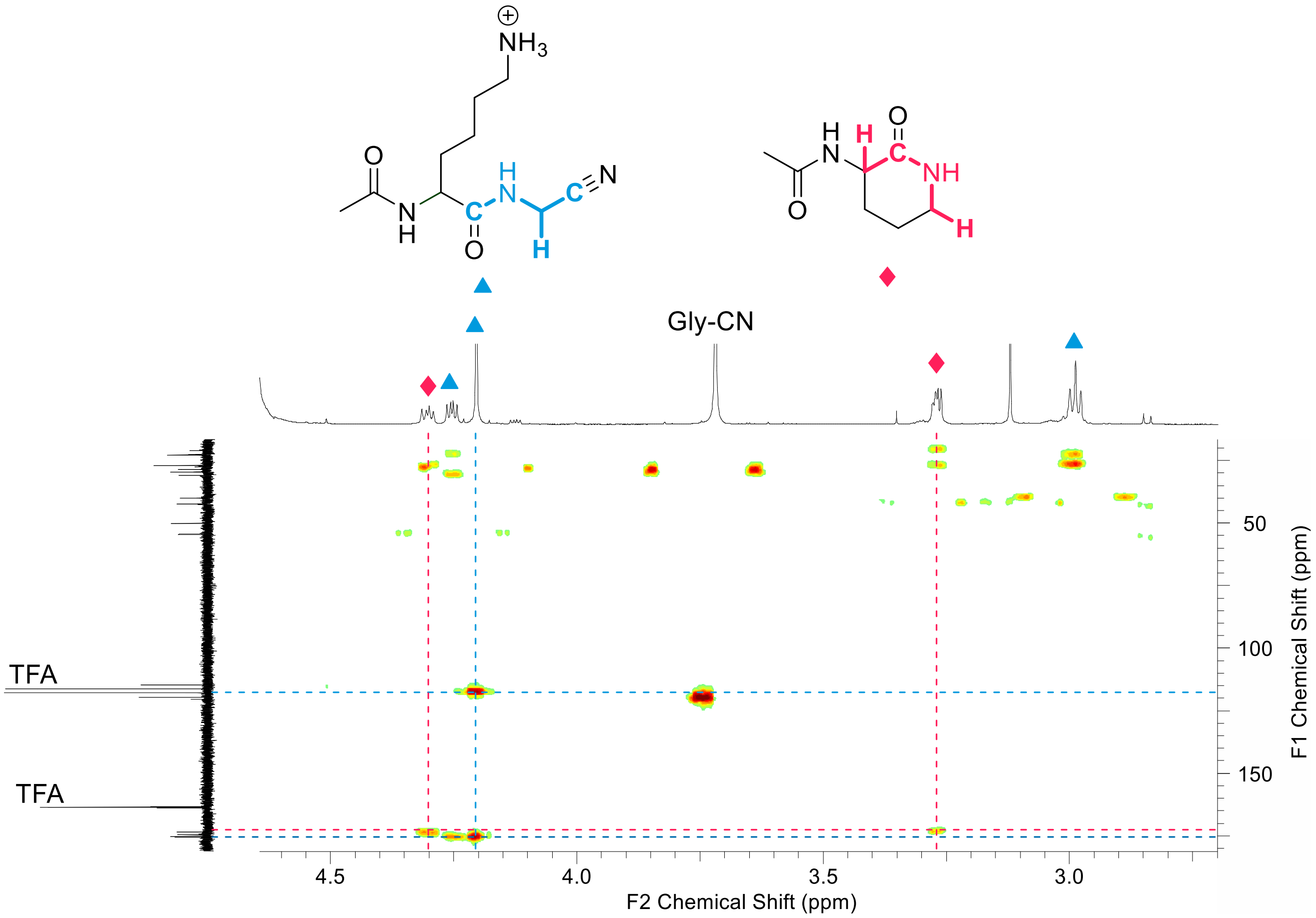

Supplementary Figure 41. *^{1}H-^{13}C HMBC (^{1}H-700 MHz [1.8–5.2 ppm], ^{13}C-176 MHz [15–185 ppm], D_2O) spectrum showing the $^{2}J_{CH}$ and $^{3}J_{CH}$ couplings of Gly-(C2)–H_2 at 4.25 ppm of* ***Ac-Lys-Gly-CN*** *to C=O resonance at 175.5 ppm and CN resonance at 117.7 ppm, and the $^{2}J_{CH}$ and $^{3}J_{CH}$ couplings of Orn-(C2)–H at 4.30 ppm and Orn-(C5)–H_2 at 3.27 ppm of* ***2*** *to C=O resonance at 173.4 ppm, which are diagnostic for peptide and lactam formation, respectively.* ***Gly-CN*** *^{1}H resonance is misaligned with ^{13}C correlation due to fluctuation in pH between ^{1}H and HMBC acquisitions. The Gly-(C2)–H_2 ^{1}H resonance is sensitive to pH changes due to the variable protonation state of* ***Gly-CN*** *amine moiety.*

Prebiotic couplings of Ac-Lys-SH, Ac-Dpr-SH and Gly-CN.

Ac-Lys-SH (60 mM) and **Ac-Dpr-SH** (60 mM) were observed by NMR spectroscopy in D_2O at pD 7.5. Both compounds were stable over a period of 3 days.

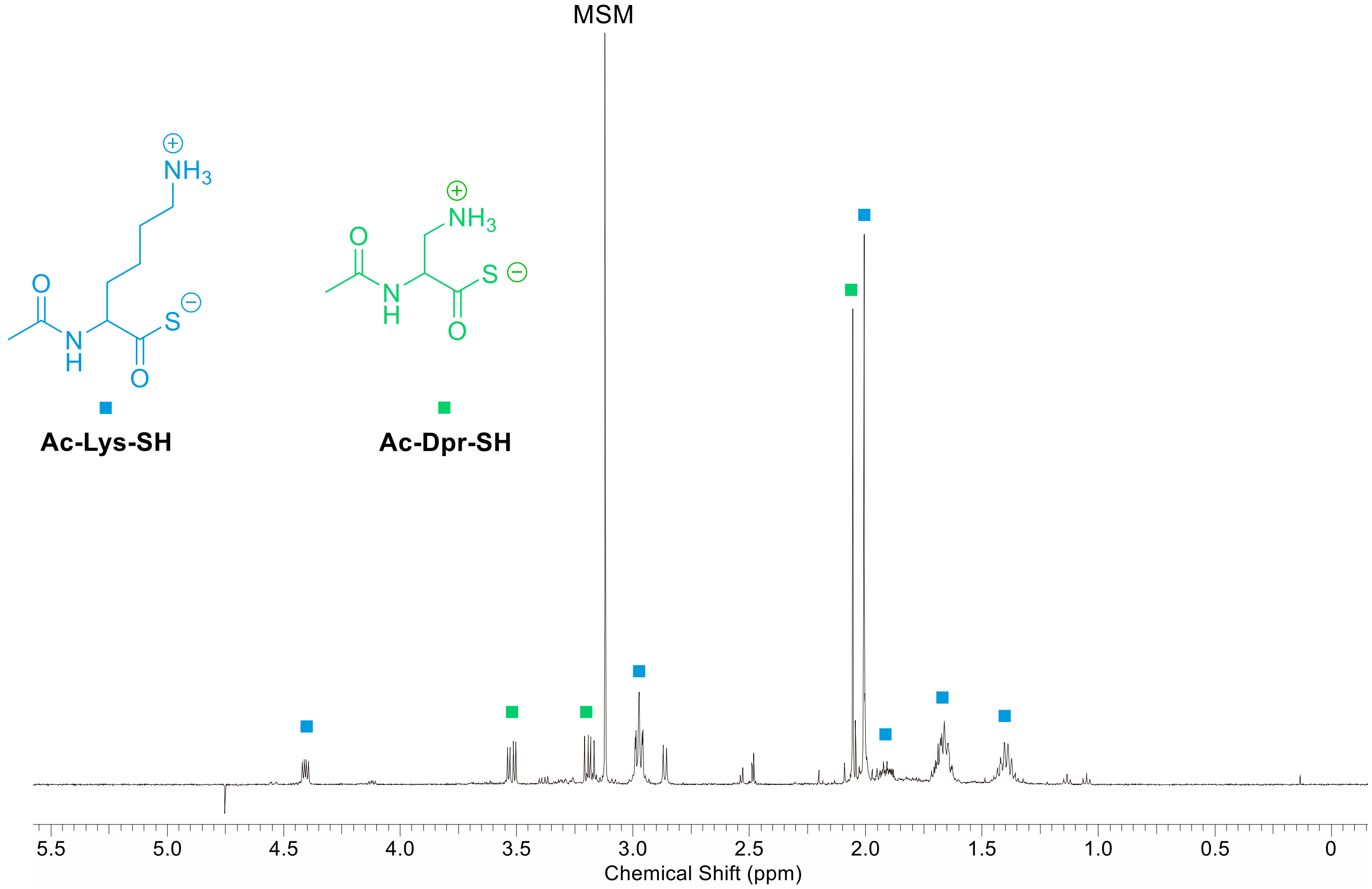

Supplementary Figure 42. [1]*H NMR (700 MHz, D_2O, 0.0 – 5.5 ppm) spectrum showing a mixture of **Ac-Lys-SH** (60 mM) and **Ac-Dpr-SH** (60 mM) at pD 7.5. The (C2)–H of **Ac-Dpr-SH** is under water.*

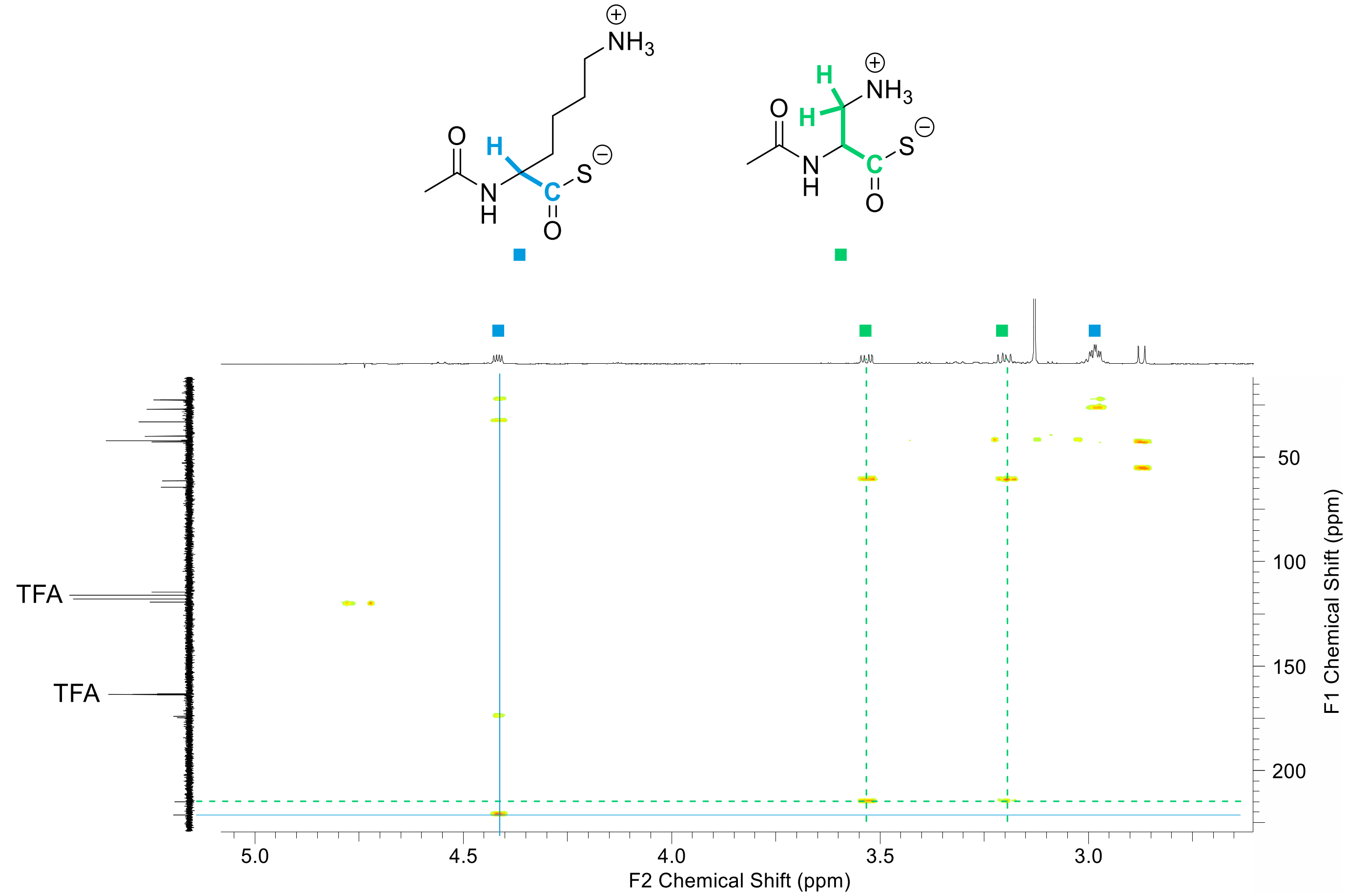

*Supplementary Figure 43. ^{1}H-^{13}C HMBC (^{1}H-700 MHz [2.3–5.1 ppm], ^{13}C-176 MHz [15–225 ppm], D_2O) spectrum showing the $^{2}J_{CH}$ coupling of Lys-(C2)–H at 4.41 ppm of **Ac-Lys-SH** to C=O resonance at 221 ppm, and the $^{3}J_{CH}$ couplings of Dpr-(C3)–H and Dpr-(C3)–H' at 3.52 ppm and 3.19 ppm of **Ac-Dpr-SH** to C=O resonance at 215 ppm, which are diagnostic thioacid formation.*

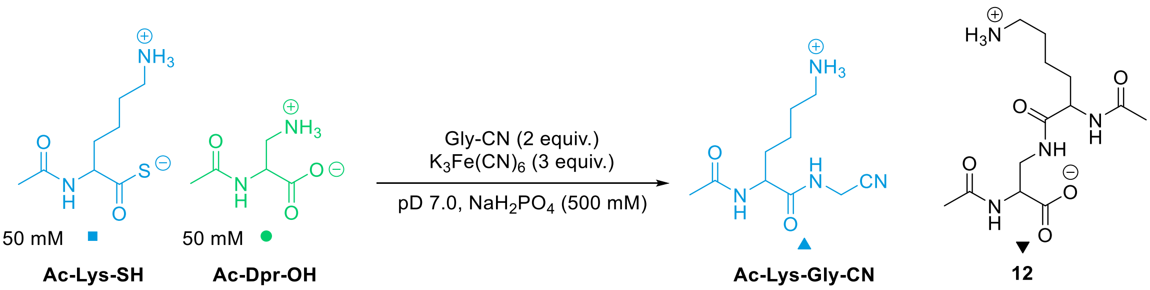

Reaction carried out using **Ac-Lys-SH** (50 mM), **Ac-Dpr-OH** (1 equiv.), **Gly-CN** (2 equiv.) and $K_3Fe(CN)_6$ (3 equiv.) in phosphate buffer (500 mM, pD 7.0). The reaction mixture was stirred at room temperature for 30 min and then centrifuged. The supernatant was analysed by 1D and 2D NMR spectroscopy, yielding **Ac-Lys-Gly-CN** (94%), returning **Ac-Dpr-OH** (82%) and yielding what we tentatively assign as **12** (<8%).

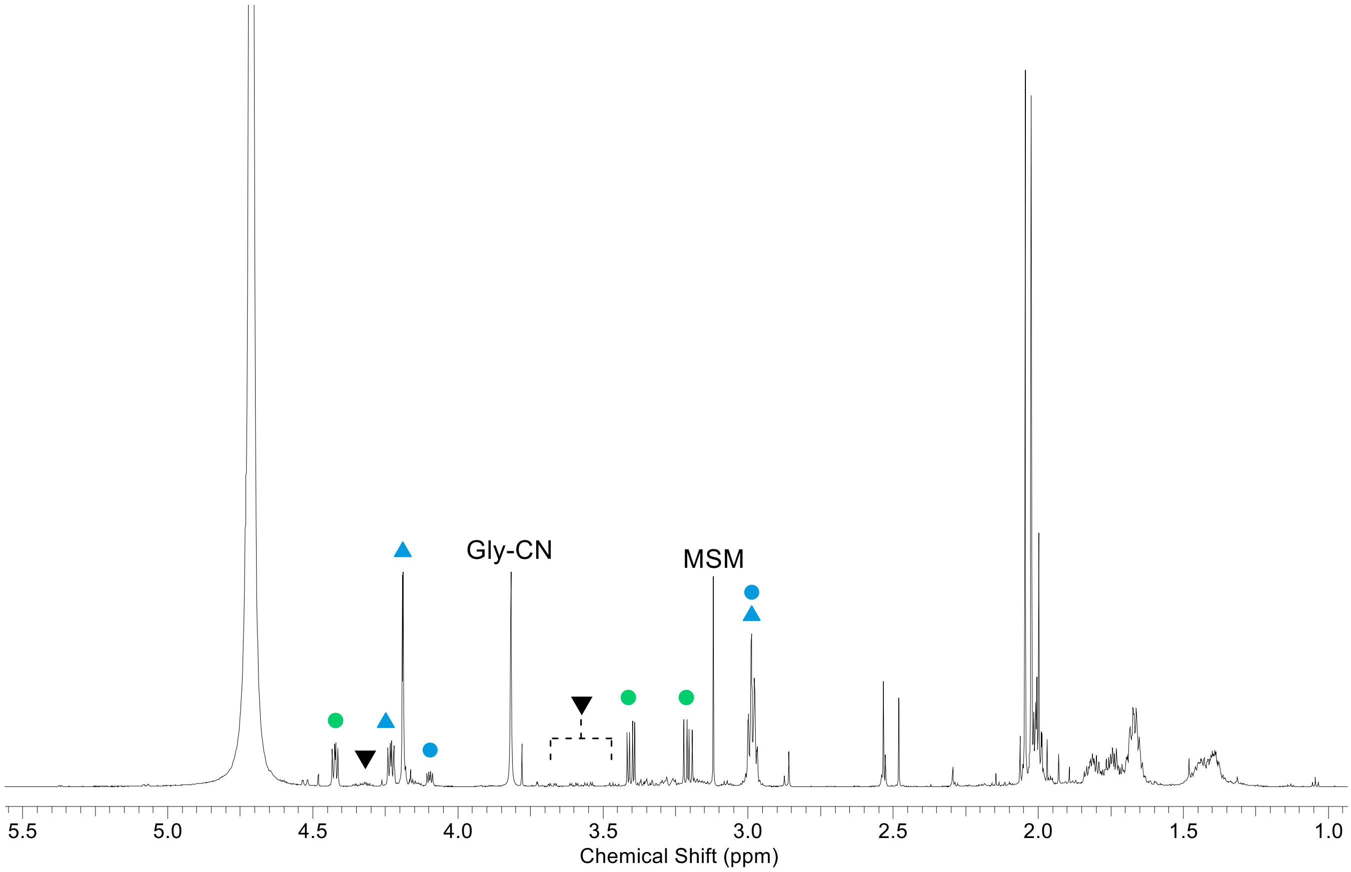

Supplementary Figure 44. ^{1}*H NMR (700 MHz,* D_2O*, 1.0 – 5.5 ppm) spectrum showing the reaction of* ***Ac-Lys-SH*** *(50 mM),* ***Ac-Dpr-OH*** *(50 mM),* ***Gly-CN*** *(2 equiv.) and* $K_3Fe(CN)_6$ *(3 equiv.) buffered in phosphate (500 mM,* D_2O*, pD 7.0).* ***Ac-Lys-OH*** *(●, 11 mM) was present in starting material.*

12 (▲): **HRMS-ESI** $[M+H]^+$ calc. for $C_{13}H_{25}N_4O_5^+$ 317.18195; obs 317.1817.

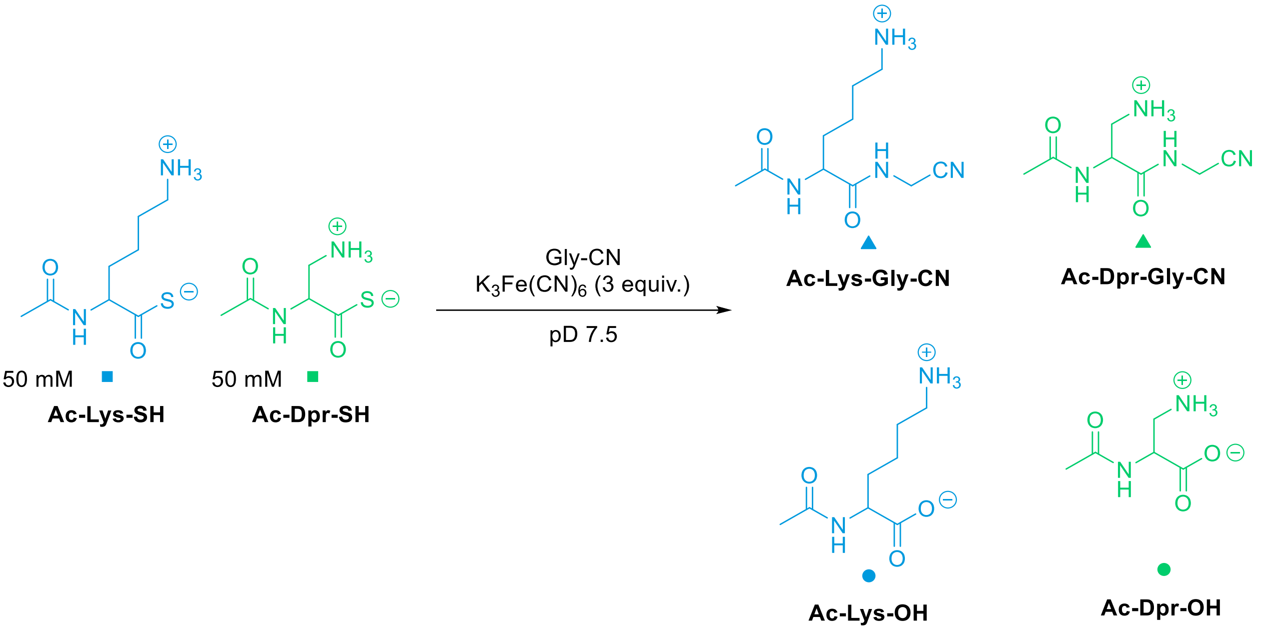

Reactions were carried out using **Ac-Lys-SH** (50 mM, 1 equiv.), **Ac-Dpr-SH** (50 mM), **Gly-CN** (1, 2 or 4 equiv.) and $K_3Fe(CN)_6$ (6 equiv.) at pD 7.5. The reaction mixture was stirred at room temperature for 30 min and then centrifuged. The supernatant was analysed by 1D and 2D NMR spectroscopy.

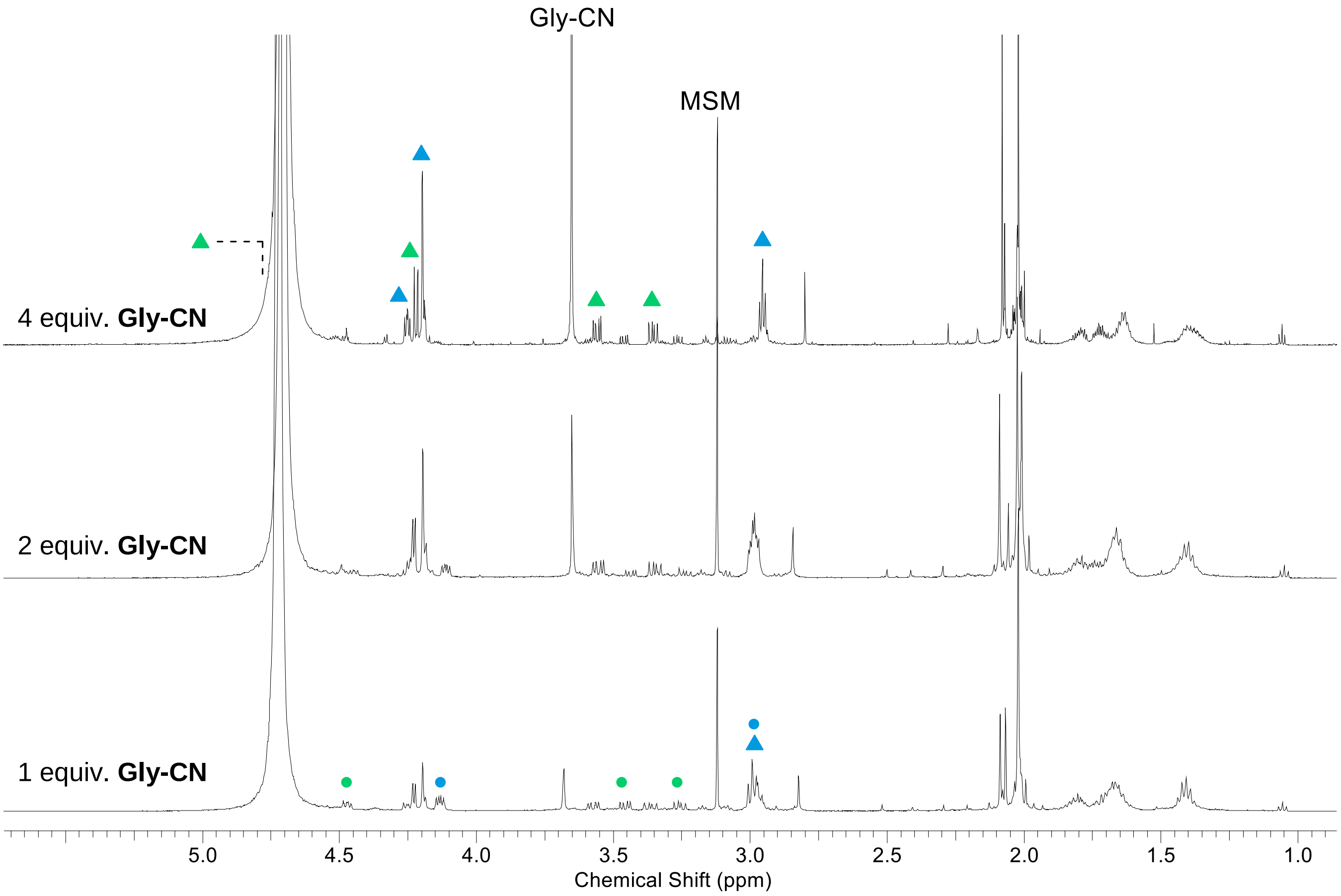

*Supplementary Figure 45. ^{1}H NMR (700 MHz, D_2O, 0.0 – 5.5 ppm) spectra showing the reaction of **Ac-Lys-SH** (60 mM), **Ac-Dpr-SH** (1.00 equiv.) and $K_3Fe(CN)_6$ (6 equiv.) with **Gly-CN** (1 equiv., bottom spectrum) **Gly-CN** (2 equiv., middle spectrum) or **Gly-CN** (4 equiv., top spectrum) at pD 7.5.*

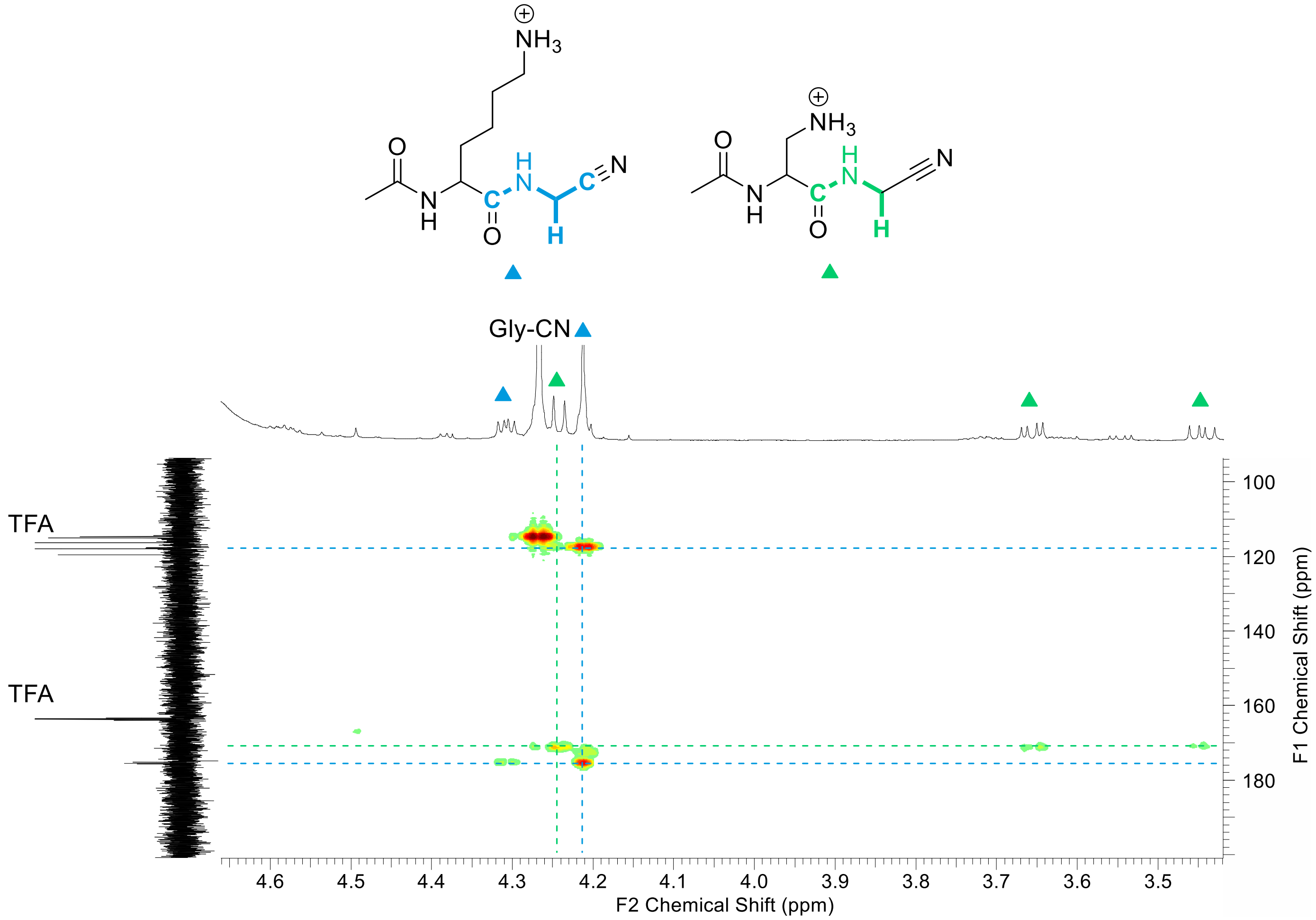

*Supplementary Figure 46. ^{1}H-^{13}C HMBC (^{1}H-700 MHz [1.8–5.2 ppm], ^{13}C-176 MHz [15–185 ppm], D_2O) spectrum showing the $^{2}J_{CH}$ and $^{3}J_{CH}$ couplings of Lys-Gly-(C2)–H_2 at 4.60 ppm of **Ac-Lys-Gly-CN** to C=O resonance at 175.5 ppm and CN resonance at 117.7 ppm, and the $^{3}J_{CH}$ coupling of Dpr-Gly-(C2)–H_2 at 4.51 ppm of **Ac-Dpr-SH** to C=O resonance at 173.4 ppm, which are diagnostic for peptide formation.*

Entry	**Ac-Lys-SH** / mM	**Ac-Dpr-SH** / equiv.	**Gly-CN** / equiv.	**Ac-Lys-Gly-CN** / %	**Ac-Dpr-Gly-CN** / %	Lys:Dpr
1	60	0	1	57	-	-
2	60	1	1	35	25	1.4
3	60	1	2	55	37	1.5
4	60	1	4	84	52	1.6

*Supplementary Table 6. ^{1}H NMR yields for the reaction of **Ac-Lys-SH** (60 mM), **Ac-Dpr-SH** (0 – 60 mM), **Gly-CN** (1 – 4 equiv.) and $K_3Fe(CN)_6$ (3 equiv. per thioacid) at pD 7.5.*

Prebiotic couplings of Ac-Lys-SH, Ac-Dpr-Gly-SH and Gly-CN

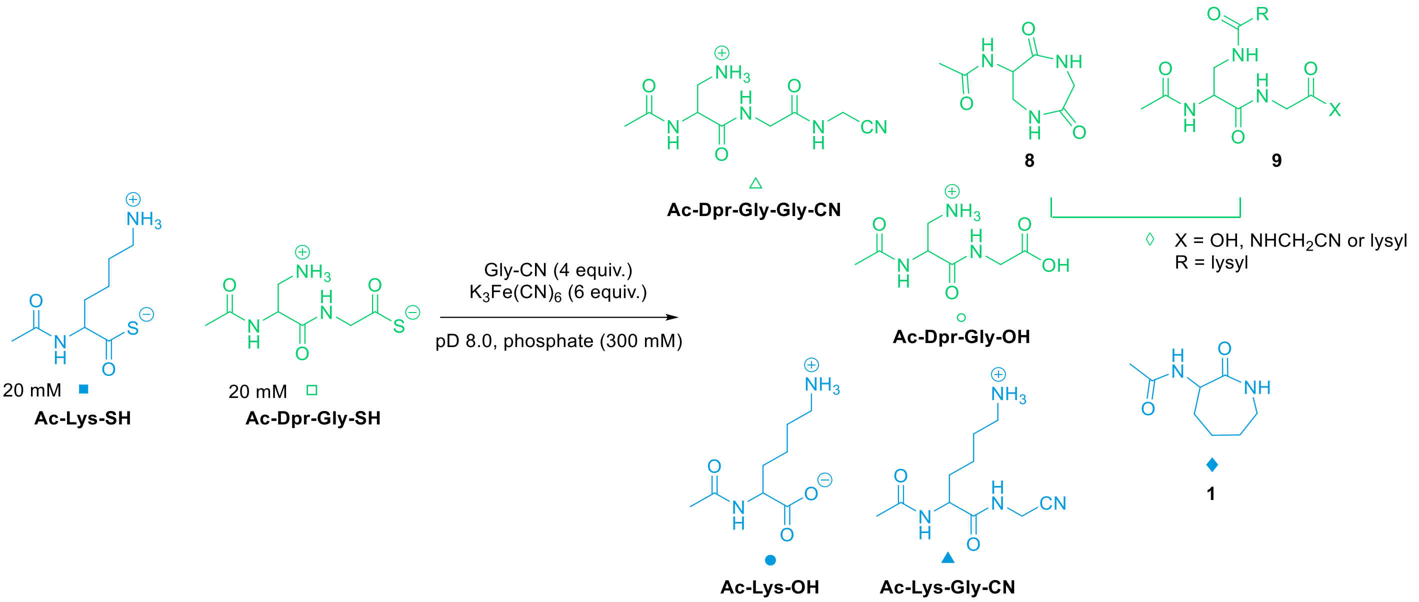

Reaction carried out using **Ac-Lys-SH** (20 mM), **Ac-Dpr-Gly-SH** (20 mM), **Gly-CN** (4 equiv.), $K_3Fe(CN)_6$ (6 equiv.) in phosphate buffer (300 mM, pD 8.0). The reaction mixture was stirred at room temperature for 30 min and then centrifuged. The supernatant was analysed by 1D and 2D NMR spectroscopy, yielding **Ac-Lys-Gly-CN** (58%), **1** (40%), **Ac-Dpr-Gly-Gly-CN** (40%) and β-amidation products **8**, **9** (28–38%).

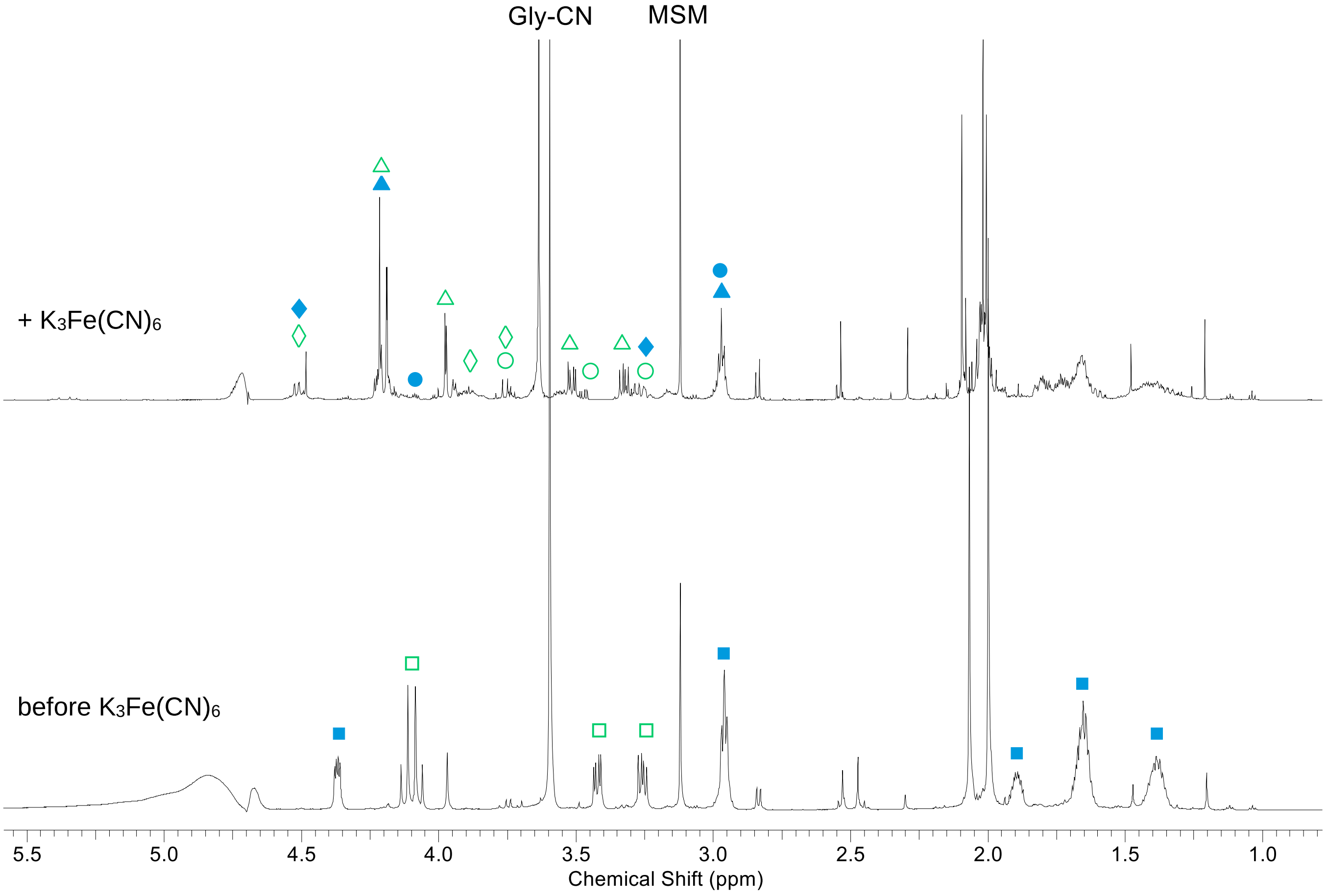

*Supplementary Figure 47. ^{1}H NMR (700 MHz, D_2O, 1.0 – 5.5 ppm, noesygppr1d) spectra showing the reaction products of **Ac-Dpr-Gly-SH** (20 mM), **Ac-Lys-SH** (1 equiv.) and **Gly-CN** (4 equiv.) in phosphate buffer (300 mM, D_2O, pD 8.0) before (bottom spectrum) and after the addition of $K_3Fe(CN)_6$ (6 equiv., top spectrum).*

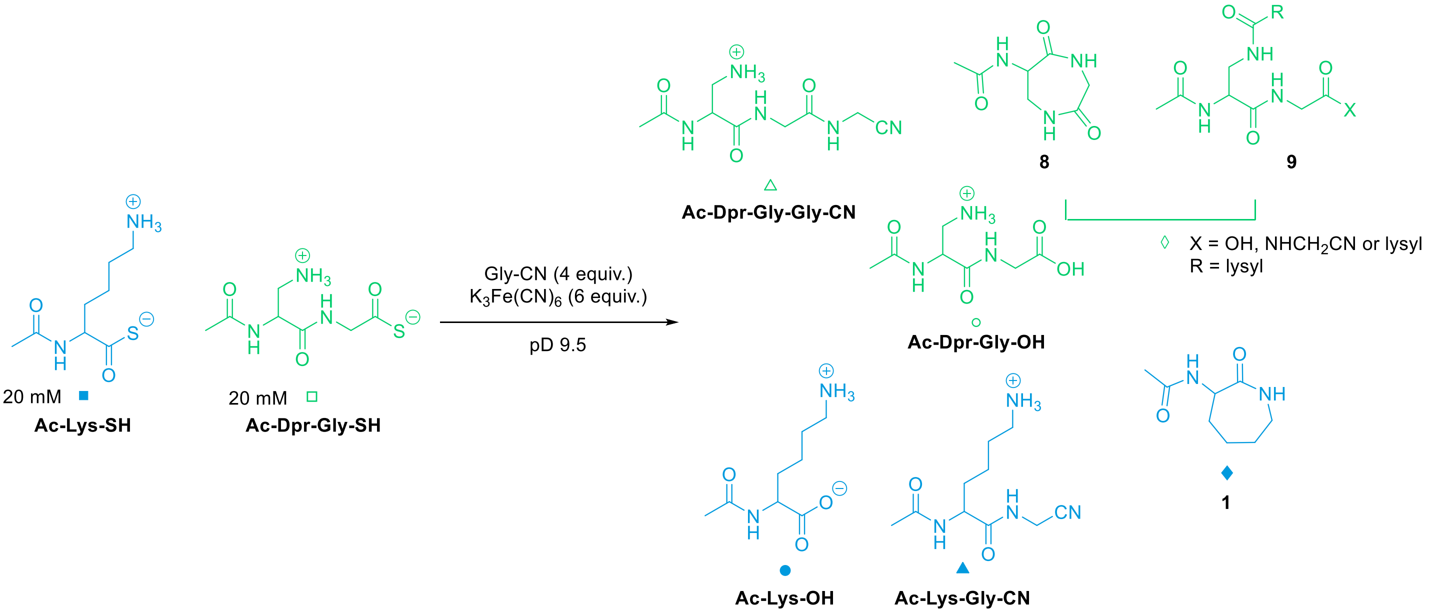

Reaction carried out using **Ac-Lys-SH** (20 mM, 1 equiv.), **Ac-Dpr-Gly-SH** (1 equiv.), **Gly-CN** (4 equiv.), $K_3Fe(CN)_6$ (6 equiv.) at pD 9.5. The reaction mixture was stirred at room temperature for 30 min and then centrifuged. The supernatant was analysed by 1D and 2D NMR spectroscopy, yielding **Ac-Lys-Gly-CN** (86%), **1** (14%), **Ac-Dpr-Gly-Gly-CN** (53%) and β-amidation products **8**, **9** (31%).

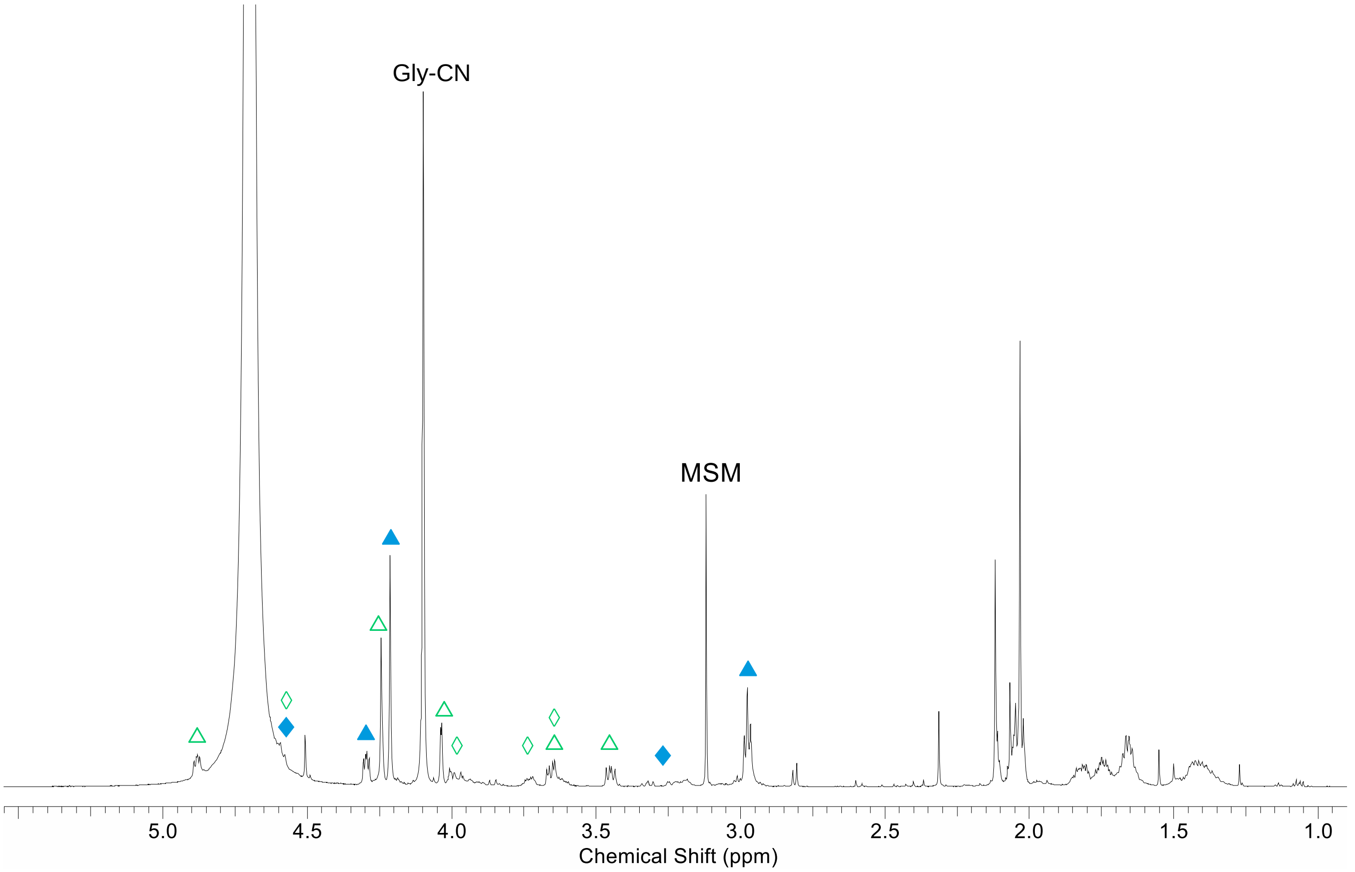

*Supplementary Figure 48. ^{1}H NMR (700 MHz, D_2O, 1.0 – 5.5 ppm) spectrum showing the reaction products of **Ac-Dpr-Gly-SH** (20 mM), **Ac-Lys-SH** (1 equiv.), **Gly-CN** (4 equiv.) and $K_3Fe(CN)_6$ (6 equiv.) at pD 9.5.*

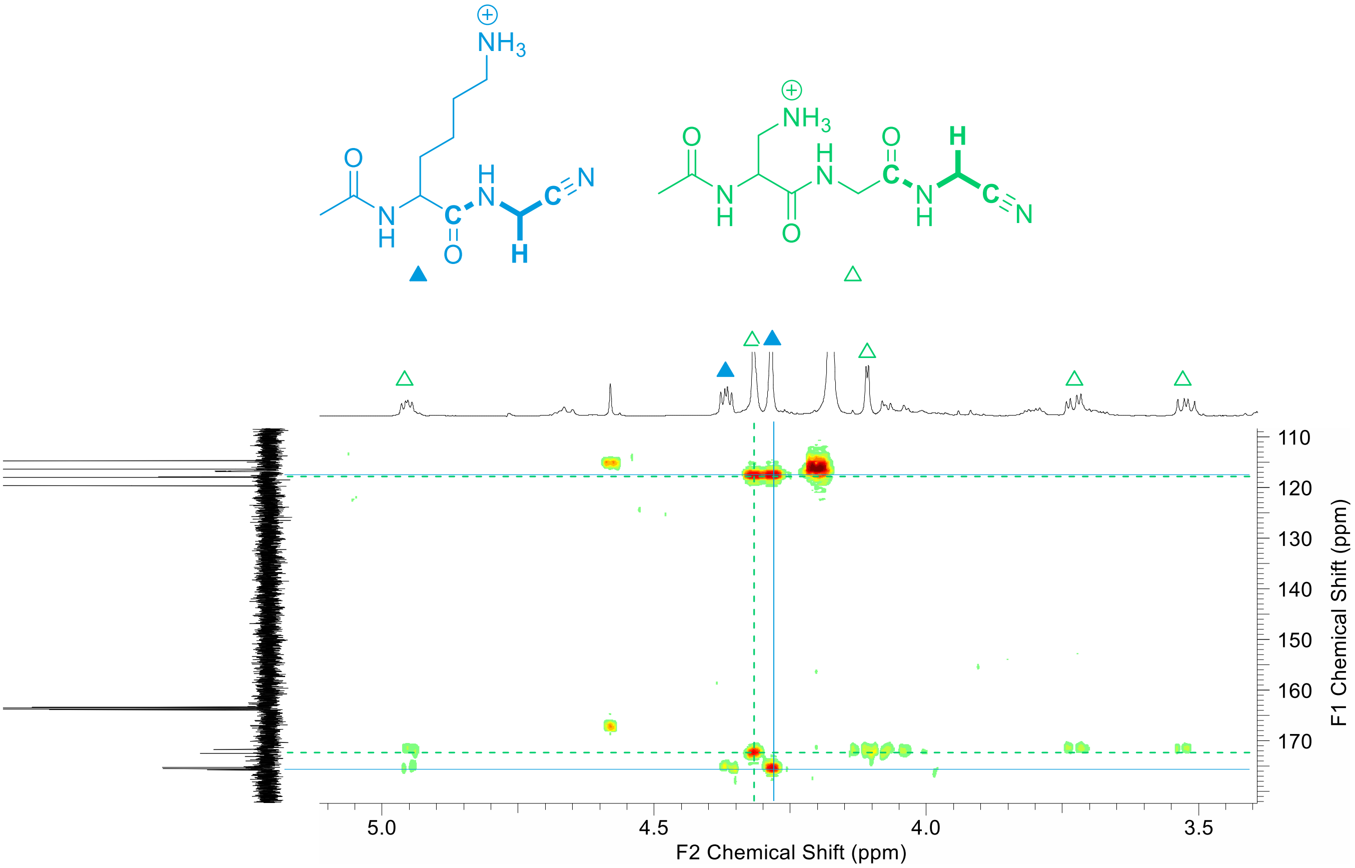

Supplementary Figure 49. ^{1}H-^{13}C *HMBC* (^{1}H*-700 MHz [3.4–5.1 ppm],* ^{13}C*-176 MHz [110–180 ppm],* D_2O*) spectrum showing the* $^{2}J_{CH}$ *and* $^{3}J_{CH}$ *couplings of Dpr-Gly-Gly-(C2)–*H_2 *at 4.32 ppm of* ***Ac-Dpr-Gly-Gly-CN*** *to C=O resonance at 172.1 ppm and CN resonance at 117.6 ppm, and the* $^{2}J_{CH}$ *and* $^{3}J_{CH}$ *couplings of Lys-Gly-(C2)–*H_2 *at 4.29 ppm of* ***Ac-Lys-Gly-CN*** *to C=O resonance at 175.5 ppm and CN resonance at 117.6 ppm which is diagnostic for peptide formation.*

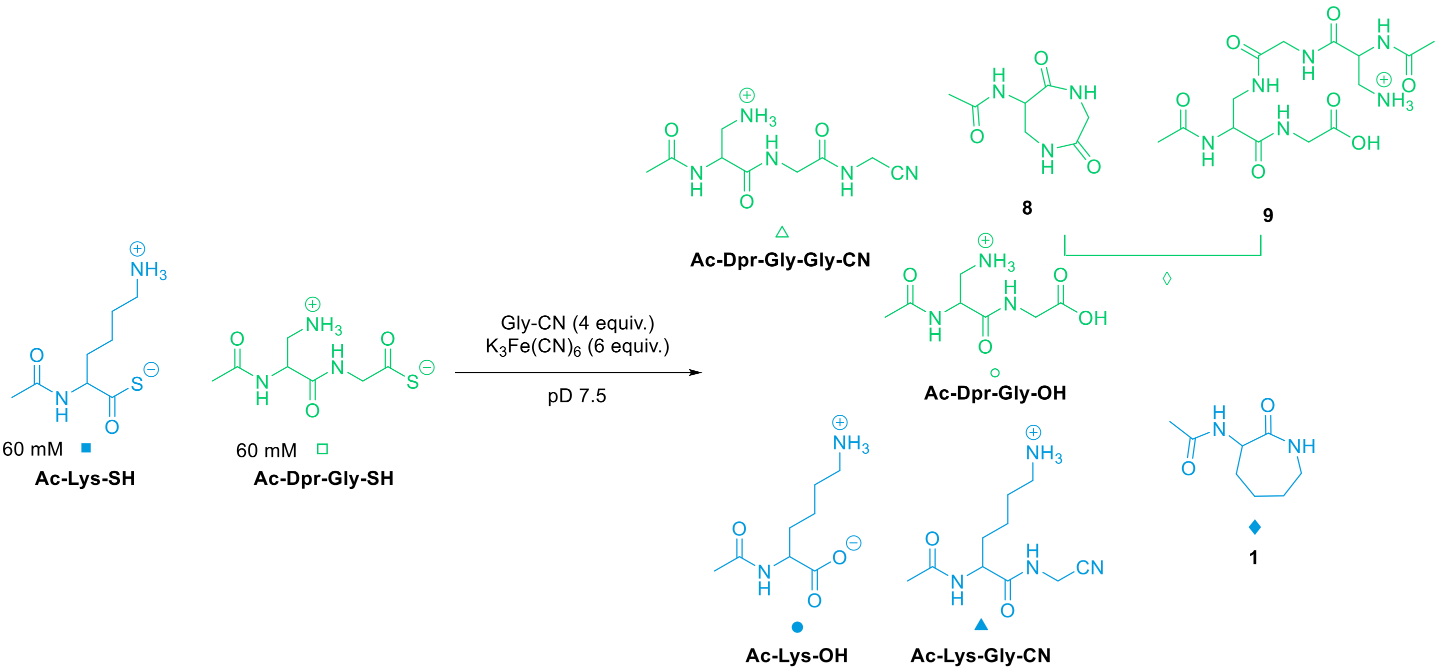

Reaction carried out using **Ac-Lys-SH** (60 mM, 1 equiv.), **Ac-Dpr-Gly-SH** (1 equiv.), **Gly-CN** (4 equiv.), $K_3Fe(CN)_6$ (6 equiv.) at pD 7.5. The reaction mixture was stirred at room temperature for 30 min and then centrifuged. The supernatant was analysed by 1D and 2D NMR spectroscopy, yielding **Ac-Lys-Gly-CN** (96%), **1** (<5%), **Ac-Dpr-Gly-Gly-CN** (69%).

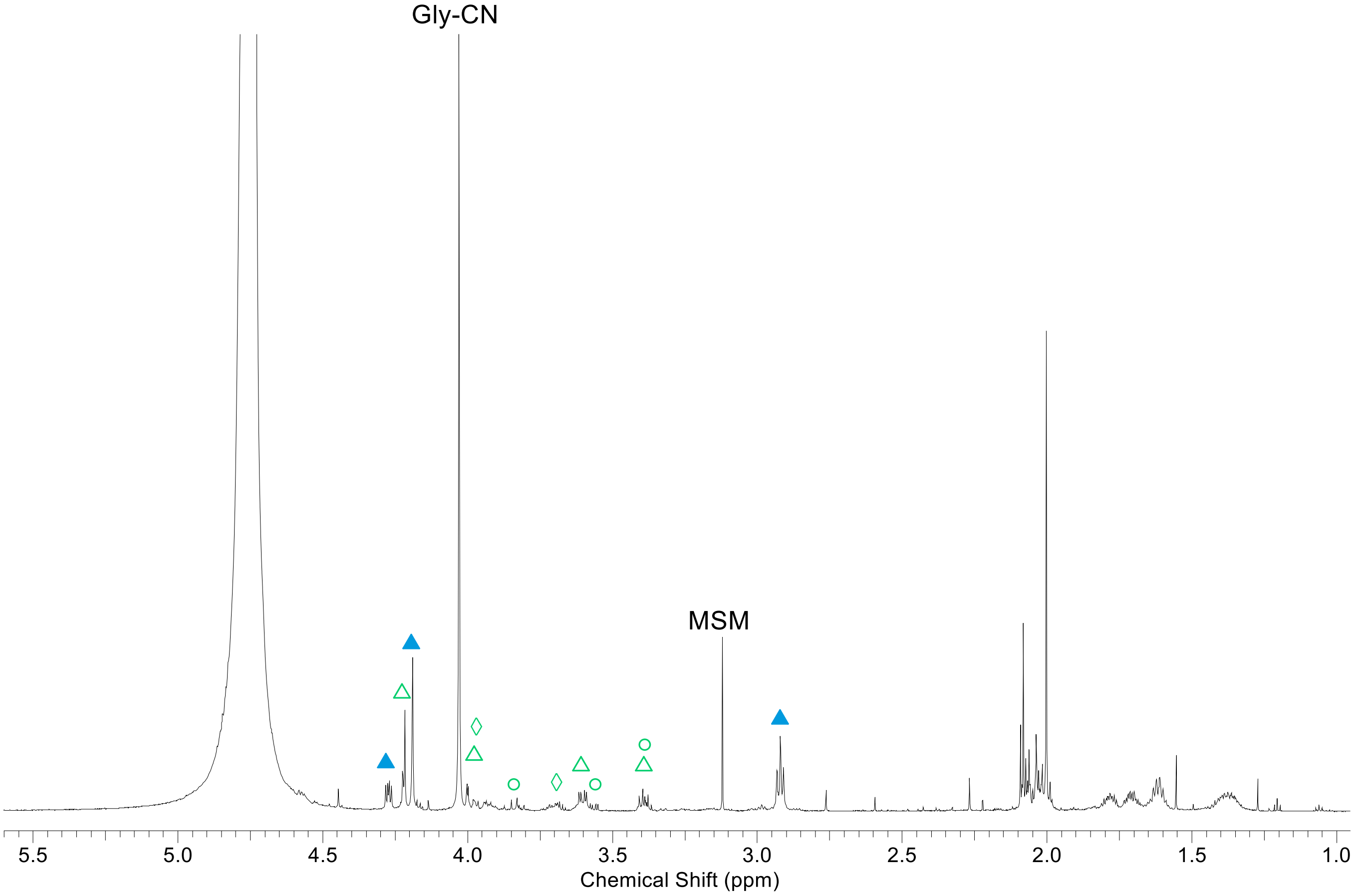

*Supplementary Figure 50. ^{1}H NMR (700 MHz, D_2O, 1.0 – 5.5 ppm) spectrum showing the reaction products of **Ac-Lys-SH** (60 mM), **Ac-Dpr-Gly-SH** (1 equiv.), **Gly-CN** (4 equiv.) and $K_3Fe(CN)_6$ (6 equiv.) at pD 7.5.*

Entry	pD	Buffer	% **Ac-Lys-Gly-CN**	% **1**	% **Ac-Dpr-Gly-Gly-CN**	% **8**+**9**
1	7.5	None	96	<5	69	*
2	9.5	None	86	14	53	31
3	8.0	Phosphate	58	40	40	28–38

Supplementary Table 7. ^{1}H NMR yields for the reaction of ***Ac-Lys-SH*** *(20 mM),* ***Ac-Dpr-Gly-SH*** *(20 mM) with* ***Gly-CN*** *(4 equiv.) and $K_3Fe(CN)_6$ at pD 7.5 (Entry 1), pD 9.5 (Entry 2) or in phosphate buffer (Entry 3, 400mM, D_2O, pD 8.0). %* ***8***+***9*** *for Entry 3 is given as a range due to signal overlap. *Signal overlap prevented accurate quantification of %* ***8***+***9***.

Ligations of Ac-AA-OH and Gly-CN using EDC·HCl

General Procedure C for EDC-mediated coupling of Ac-AA-OH and Gly-CN

To **Ac-AA-OH** (60 mM, 1 equiv.) and **Gly-CN** (2 equiv.) at pD 7.0 or in either phosphate/imidazole/MOPS buffer (600 mM, D_2O, pD 7.0) was added EDC·HCl (2 equiv.). The reaction was monitored by NMR spectroscopy.

Couplings of Ac-Lys-OH and Gly-CN with EDC·HCl

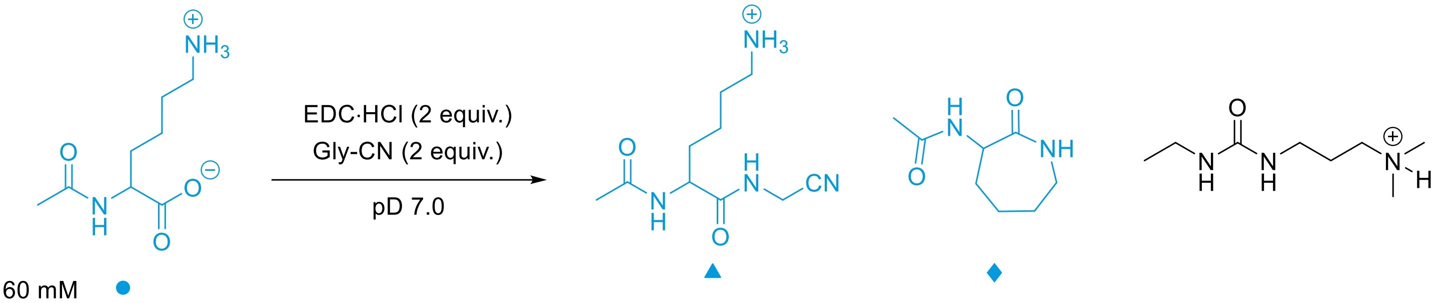

Ac-Lys-OH **Ac-Lys-Gly-CN** **1** **EDU**

Reaction carried out *via* general procedure **C** using **Ac-Lys-OH** at pD 7.0 to afford **Ac-Lys-Gly-CN** (13%) and **1** (56%) after 48 h. The solution pD was measured as 9.1 after 48 h.

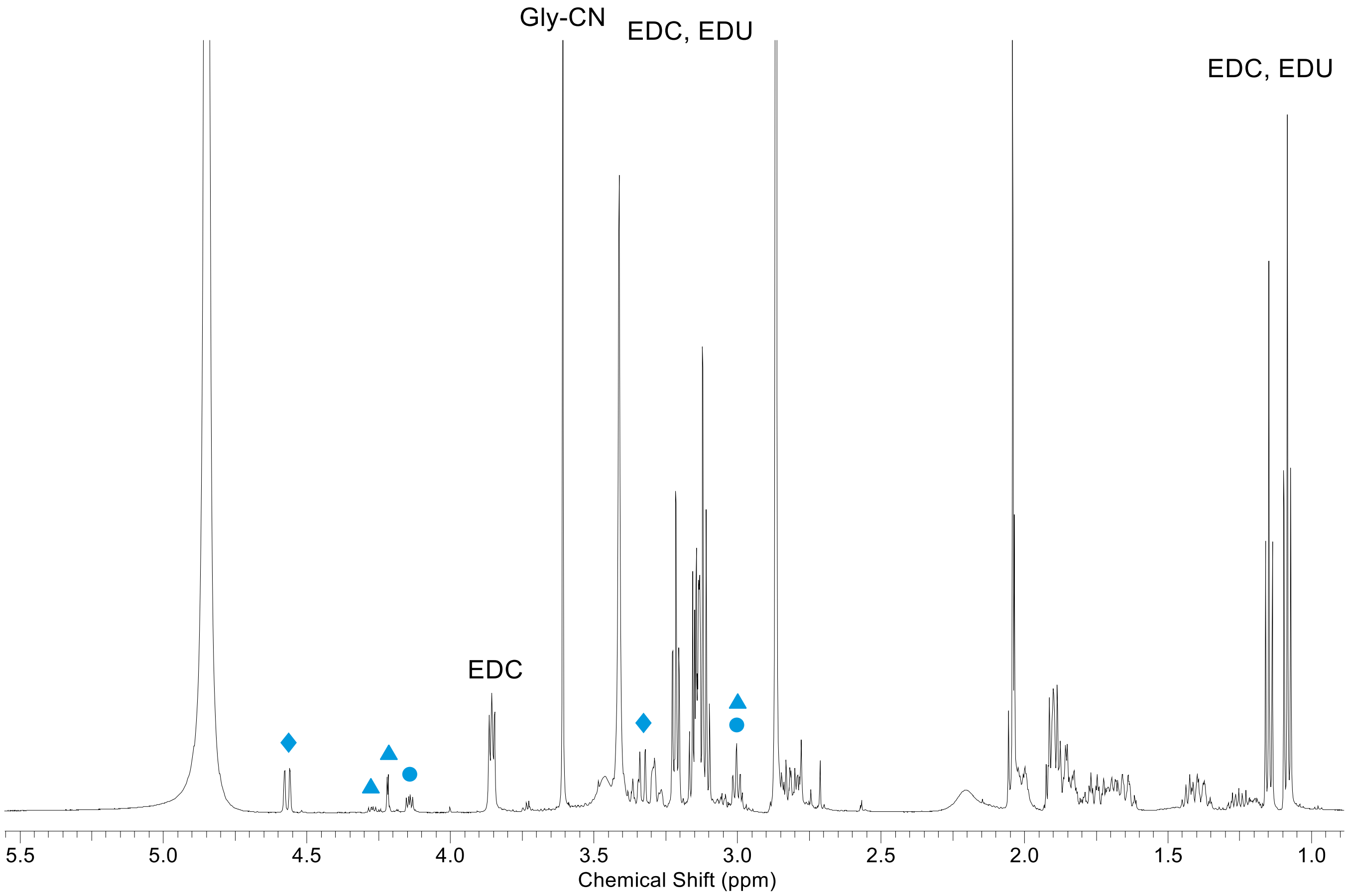

Supplementary Figure 51. ^{1}H NMR (700 MHz, D_2O, 1.0 – 5.5 ppm) spectrum showing the reaction of ***Ac-Lys-OH*** *(60 mM), EDC·HCl (2 equiv.),* ***Gly-CN*** *(2 equiv.) initiated at pD 7.0 after 48 h. The solution pD was measured as 9.1 after 48 h.*

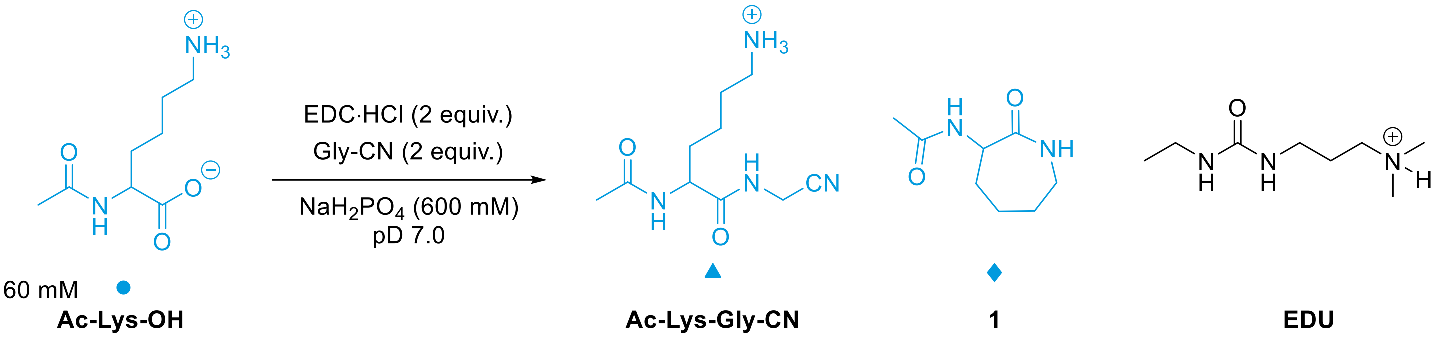

Reaction carried out *via* general procedure **C** using **Ac-Lys-OH** in phosphate buffer to afford **Ac-Lys-Gly-CN** (13%) and **1** (1%).

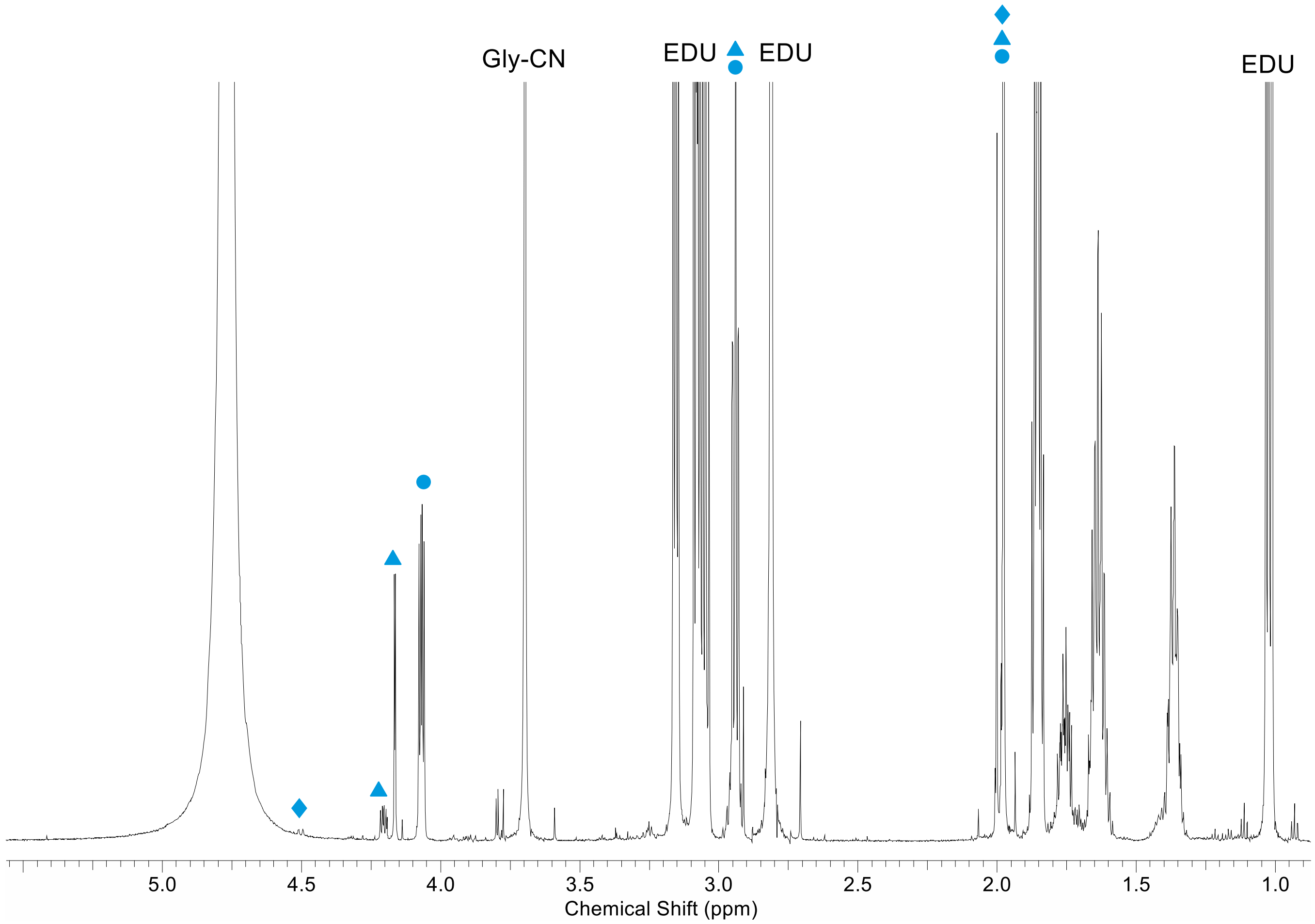

*Supplementary Figure 52. ^{1}H NMR (700 MHz, D_2O, 1.0 – 5.5 ppm) spectrum showing the reaction of **Ac-Lys-OH** (60 mM), EDC·HCl (2 equiv.), **Gly-CN** (2 equiv.) in phosphate buffer (600 mM, D_2O, pD 7.0) after 1 h.*

Coupling of Ac-Orn-OH and Gly-CN with EDC·HCl

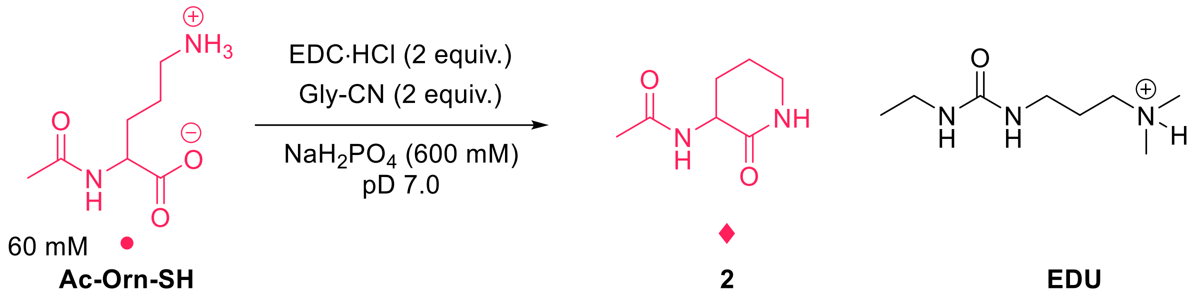

Reaction carried out *via* general procedure **C** using **Ac-Orn-OH** in phosphate buffer to afford **2** (14%).

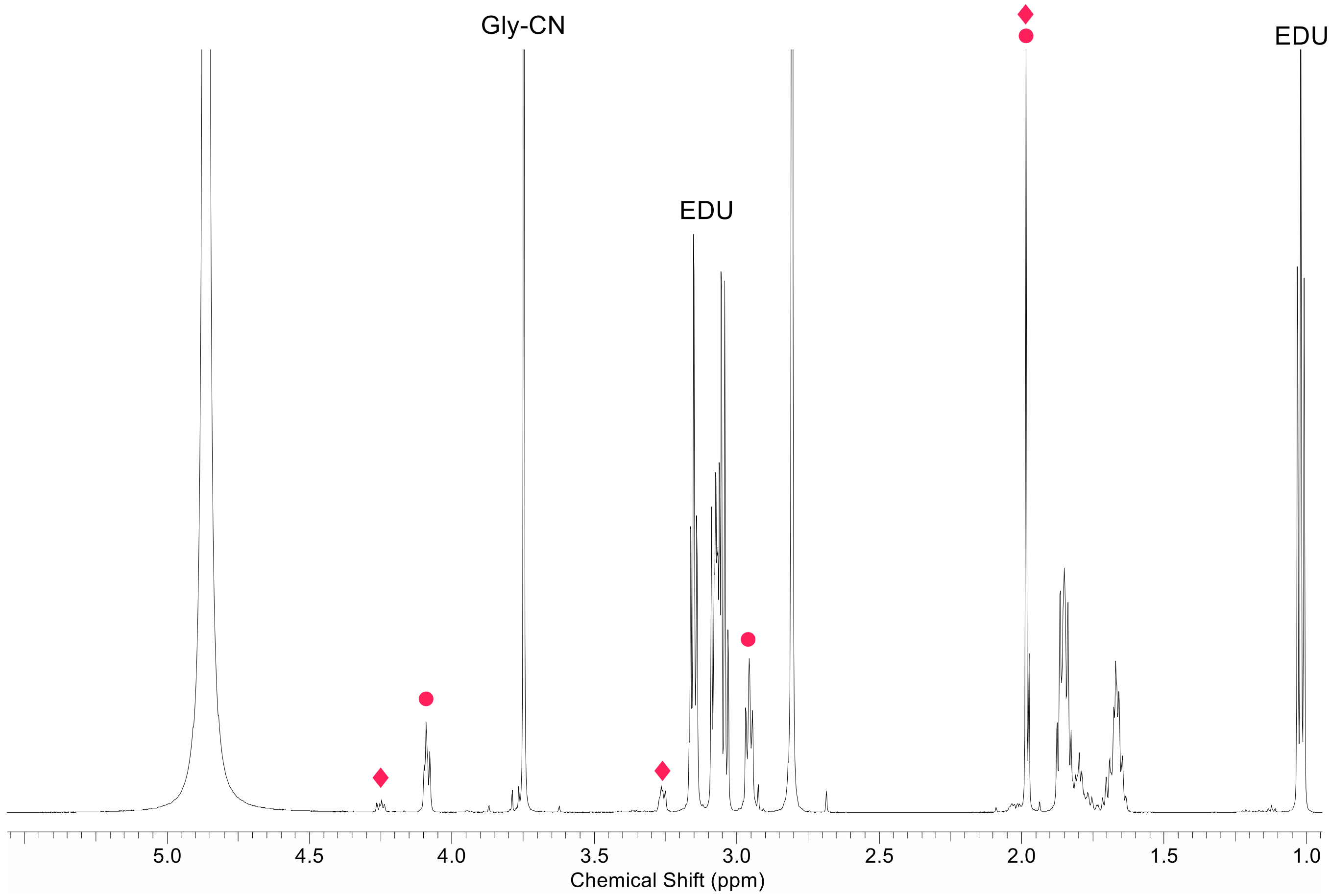

*Supplementary Figure 53. ^{1}H NMR (700 MHz, D_2O, 1.0 – 5.5 ppm) spectrum showing the reaction of a mixture of **Ac-Orn-OH** (60 mM), EDC·HCl (2 equiv.), **Gly-CN** (2 equiv.) in phosphate buffer (600 mM, D_2O, pD 7.0) after 1 h.*

Couplings of Ac-Dpr-OH and Gly-CN with EDC·HCl

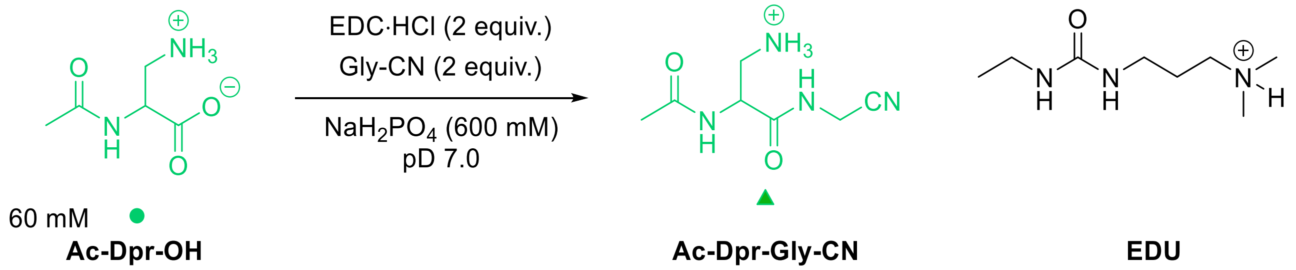

Reaction carried out *via* general procedure **C** using **Ac-Dpr-OH** in phosphate buffer to afford **Ac-Dpr-Gly-CN** (1%).

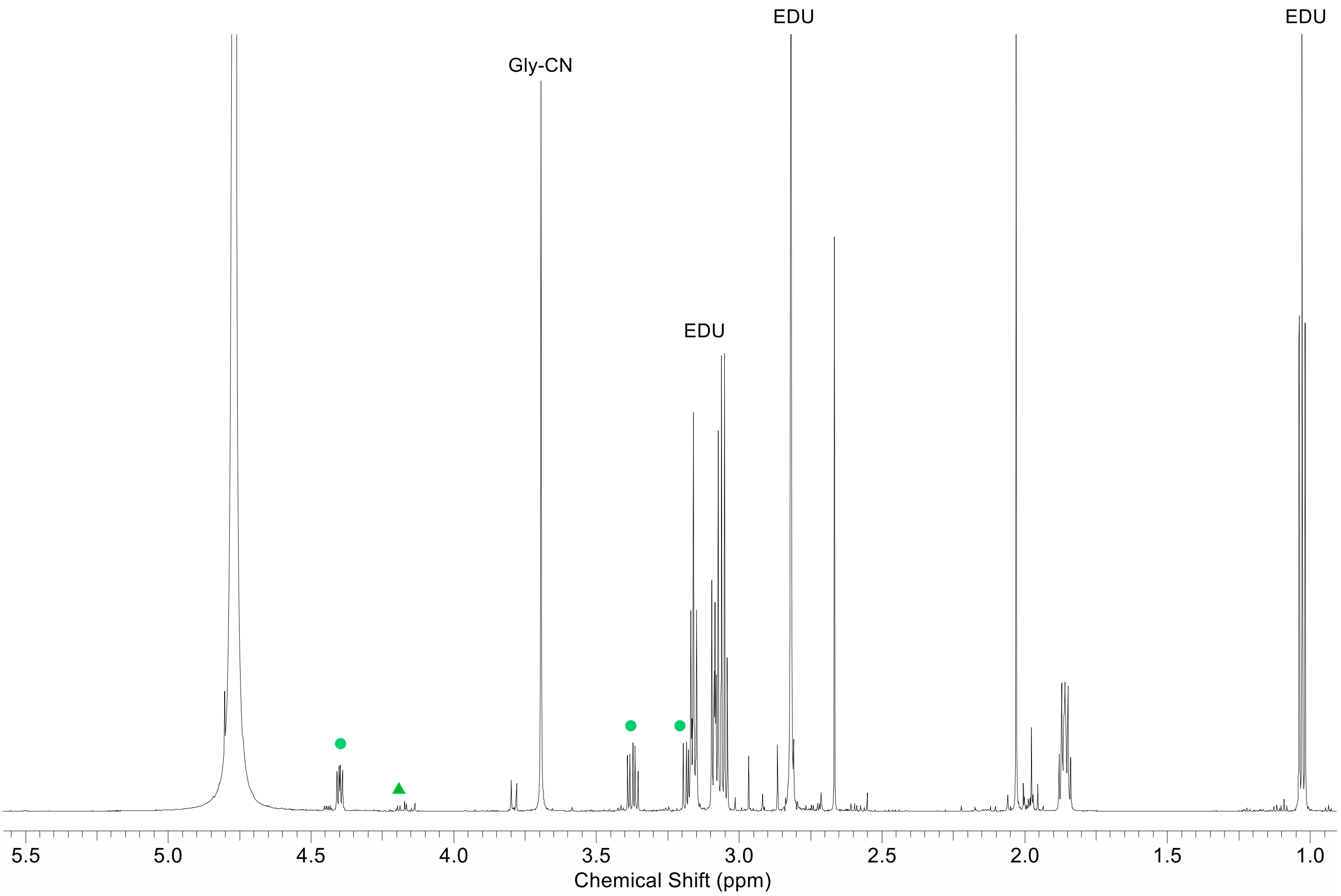

*Supplementary Figure 54. ^{1}H NMR (700 MHz, D_2O, 1.0 – 5.5 ppm) spectrum showing the reaction of **Ac-Dpr-OH** (60 mM), EDC·HCl (2 equiv.), **Gly-CN** (2 equiv.) in phosphate buffer (600 mM, D_2O, pD 7.0) after 1 h.*

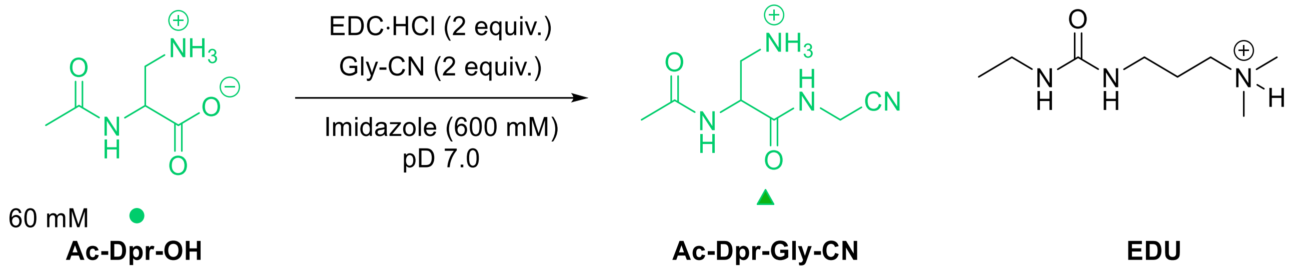

Reaction carried out *via* general procedure **C** using **Ac-Dpr-OH** in imidazole buffer to afford **Ac-Dpr-Gly-CN** (21%).

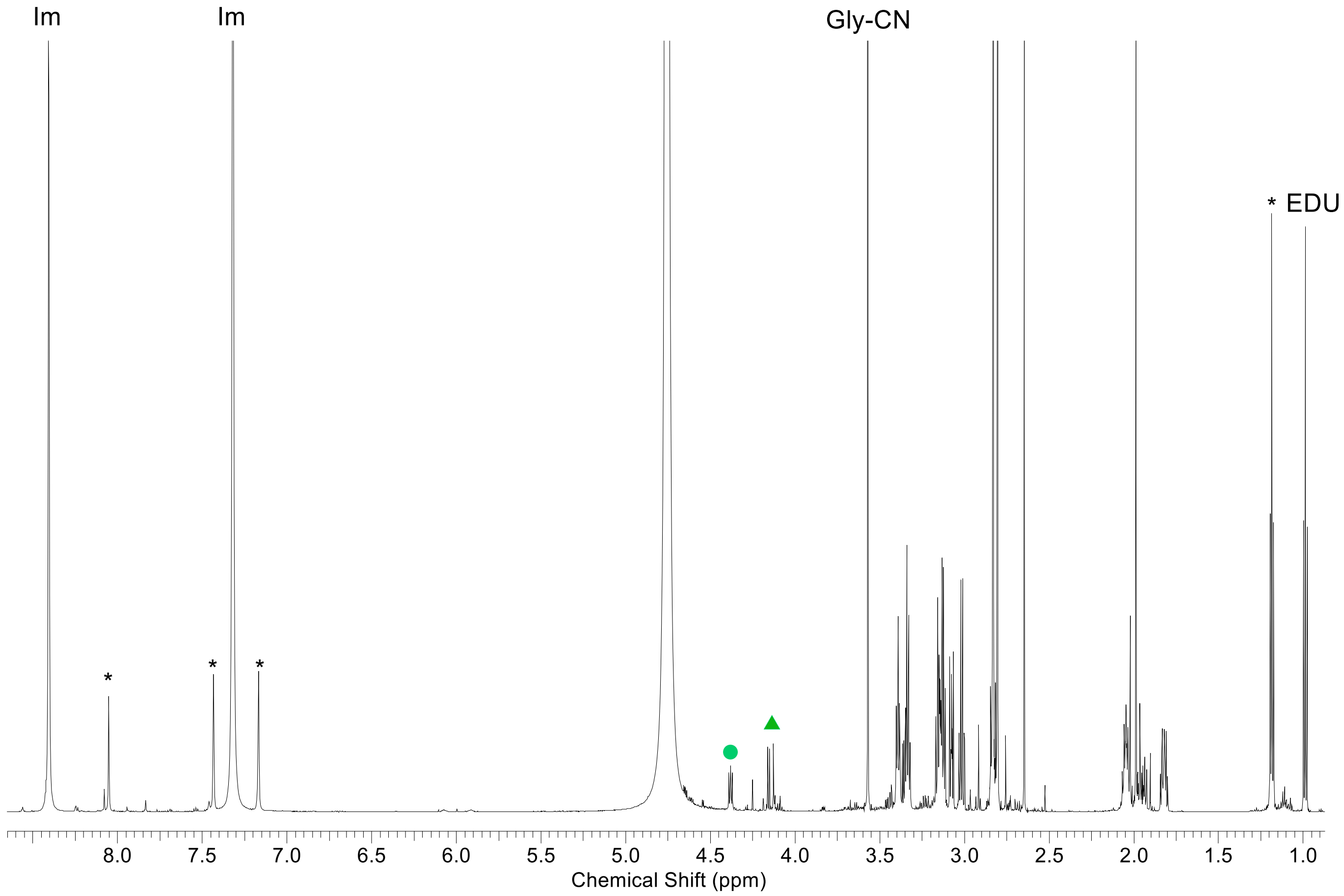

*Supplementary Figure 55. ^{1}H NMR (700 MHz, D_2O, 1.0 – 5.5 ppm) spectrum showing the reaction of **Ac-Dpr-OH** (60 mM), EDC·HCl (2 equiv.), **Gly-CN** (2 equiv.) in imidazole buffer (600 mM, D_2O, pD 7.0) after 24 h. We tentatively assign * as an EDC-Im adduct. EDC-Im was observed to accumulate during the initial phase of the reaction between **Ac-AA-OH**, **Gly-CN** and EDC at pD 7.0, as the concentration of EDC was decreasing. EDC-Im (53% from EDC) was observed to remain after all EDC was consumed at 24 h. After 24 h no further **Ac-Dpr-Gly-CN** was observed to form but EDC-Im was observed to slowly hydrolyse to yield EDU over 12 d.*

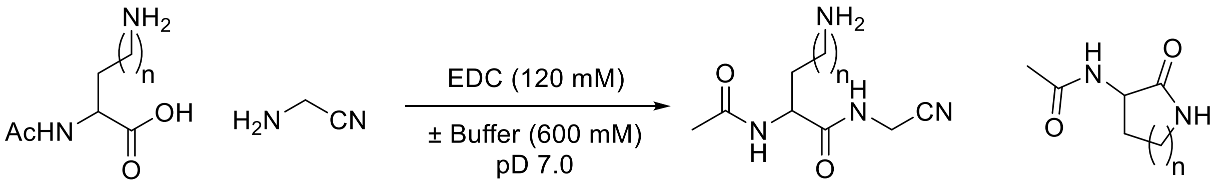

n = 3: **Lys**
n = 2: **Orn**
n = 0: **Dpr**

Gly-CN

Ac-AA-Gly-CN

n = 3: **1**
n = 2: **2**

60 mM 120 mM

Entry	AA	Buffer	Time / h	% ligation	% cyclisation	% Ac-AA-OH
1[i]	Lys	None	48	13	56	29
2[ii]	Orn	None	48	1	73	21
3[*]	Lys	Imidazole	24	48	30	24
4[*]	Orn	Imidazole	24	1	80	23
5[*]	Dpr	Imidazole	24	21	n.d.	60
6[†,‡]	Lys	MOPS	12	34	60	< 5
7	Orn	MOPS	12	n.d.	> 95	n.d.
8[†,‡]	Lys	MES	12	34	56	< 5
9	Orn	MES	12	n.d.	> 95	n.d.
10[§]	Lys	Phosphate	1	13	1	84
11[§]	Orn	Phosphate	1	n.d.	14	83
12[§]	Dpr	Phosphate	1	5	n.d.	85

*Supplementary Table 8. ^{1}H NMR yields for the reaction of **Ac-AA-OH** (60 mM), **Gly-CN** (2 equiv.) and EDC·HCl (2 equiv.) at pD 7.0 or in the relevant buffer (600 mM, D_2O, pD 7.0). [i] The solution was observed to rise to pD 9.1 over 48 h. [ii] The solution was observed to rise to pD 8.9 over 48 h. n.d. = not detected.*

[*] We also observed the formation of an EDC-Im adduct (see Supplementary Figure 55) which was not observed to activate **Ac-AA-OH** towards ligation.
[†] H_2O/D_2O (99:1) at pH 6.5.
[‡] Deuteration of the (C2)–H of **Ac-Lys-Gly-CN** and **1** was observed in MOPS and MES buffered EDC-ligation in D_2O (c.f. Beaufils, D. et al. Diastereoselectivity in prebiotically relevant 5(4H)-oxazolone-mediated peptide couplings. *Chem. Commun.*, **2014,** 50, 3100.). Deuteration of **2** was not observed in MOPS or MES buffered EDC-activation in D_2O. Deuteration of **Ac-Lys-Gly-CN** and **1** was also not observed during the reaction of **Ac-Lys-SH** with $K_3Fe(CN)_6$/**Gly-CN** in any buffer, including MOPS/MES buffers, in D_2O (see Supplementary Table 1).
[§] The yields of phosphate buffered EDC-ligations (Supplementary Table 8, Entries 10–12) are likely low due to the reaction of the buffer with EDC (pyrophosphate was observed as a by-product).

pH titrations of amines

Aqueous solutions containing the relevant molecule and MSM were adjusted to the desired pH using HCl/NaOH and submitted to analysis by ^{1}H NMR. The chemical shift of the relevant proton(s) (**Ac-Dpr-SH**, **Ac-Dpr-OH**, **Ac-Dpr-CN**, **Ac-Dpr-Gly-SH**: most downfield β-CH signal; **Ac-Lys-SH**, **Ac-Lys-OH**, **Ac-Lys-CN**, **Ac-Lys-Gly-SH**: ε-CH_2) were recorded following referencing all ^{1}H NMR spectra to the methyl protons of MSM. Chemical shift was plotted against pH across a range of pHs to give the relevant pH titration curve, where pH = pK_{aH} at the equivalence point.

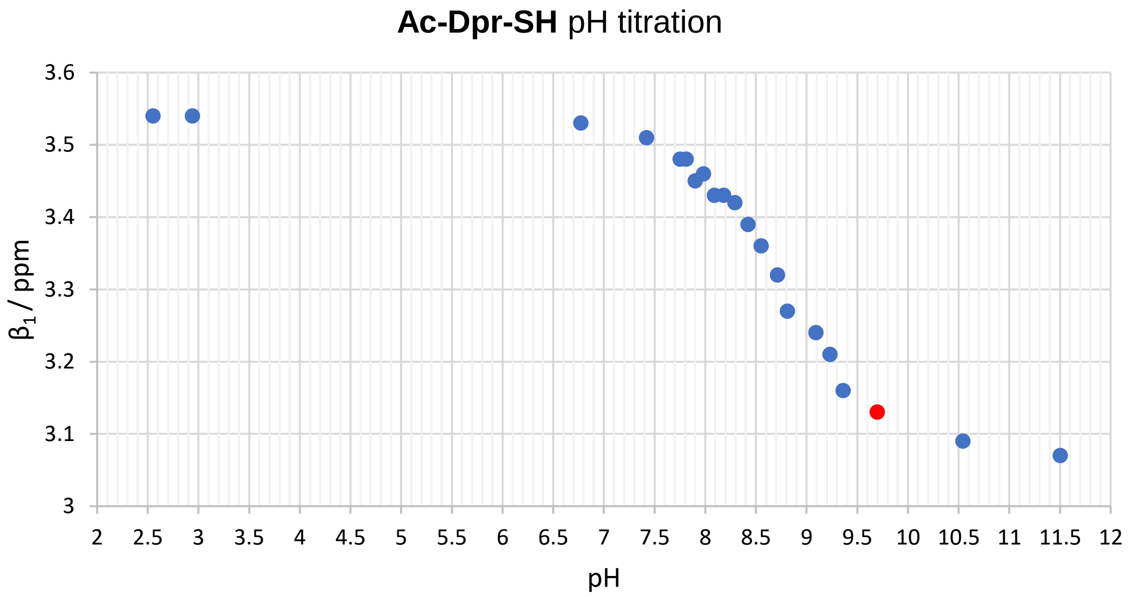

*Supplementary Figure 56. pH titration curve of **Ac-Dpr-SH**. pK_{aH} (β-$CH_2NH_3^+$ → β-CH_2NH_2) was determined to be approximately 8.7. Data points in red are those for which the chemical shift of MSM overlaps with that of the β-proton.*

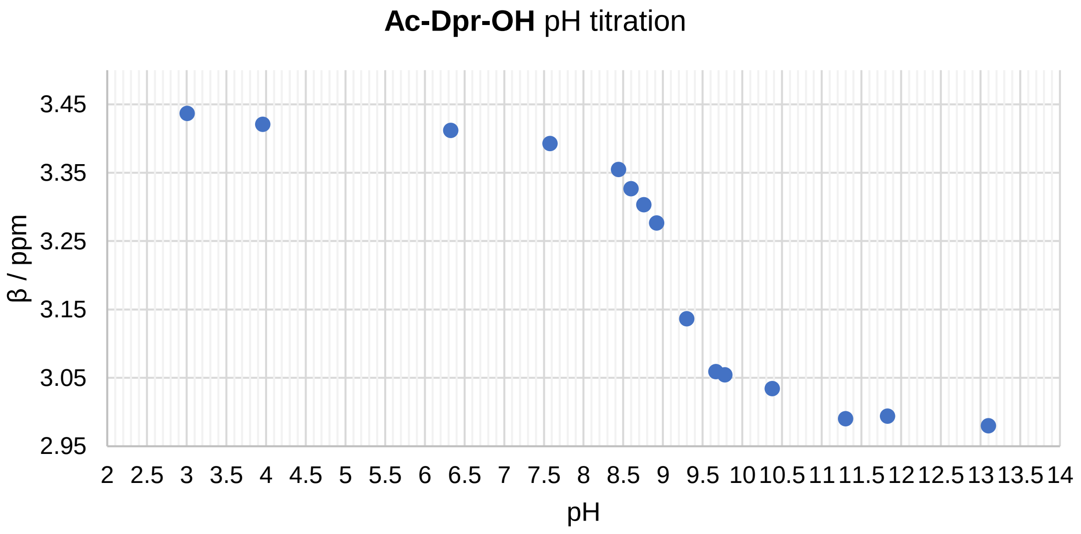

*Supplementary Figure 57. pH titration curve of **Ac-Dpr-OH**. pK_{aH} (β-$CH_2NH_3^+$ → β-CH_2NH_2) was determined to be approximately 9.2.*

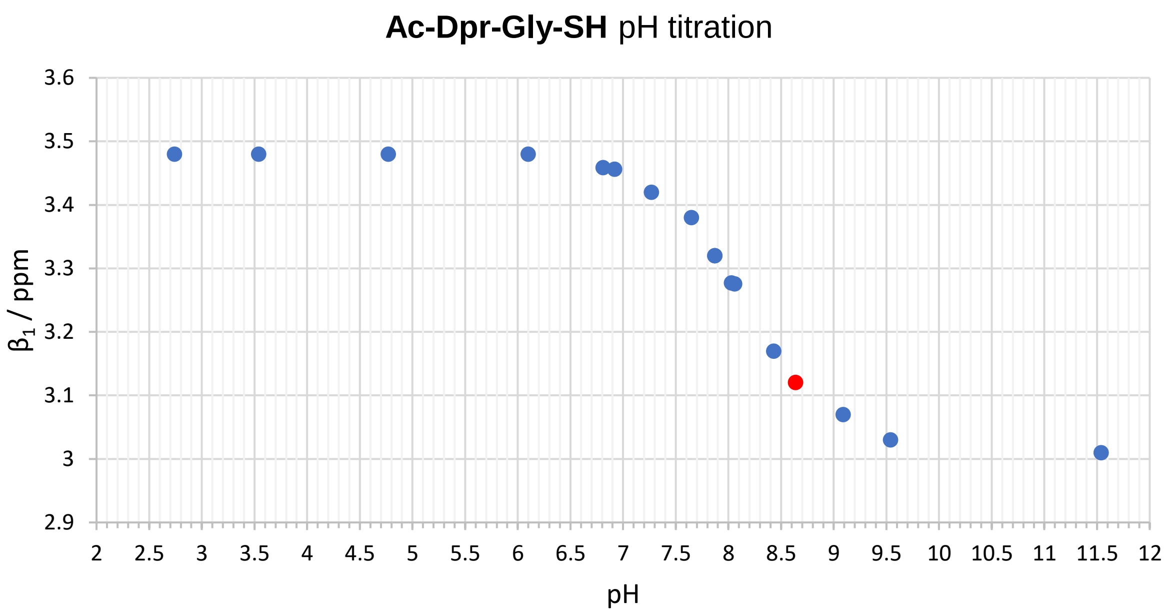

*Supplementary Figure 58. pH titration curve of **Ac-Dpr-Gly-SH**. pK_{aH} (β-$CH_2NH_3^+$ → β-CH_2NH_2) was determined to be approximately 8.1. Data points in red are those for which the chemical shift of MSM overlaps with that of the β-proton.*

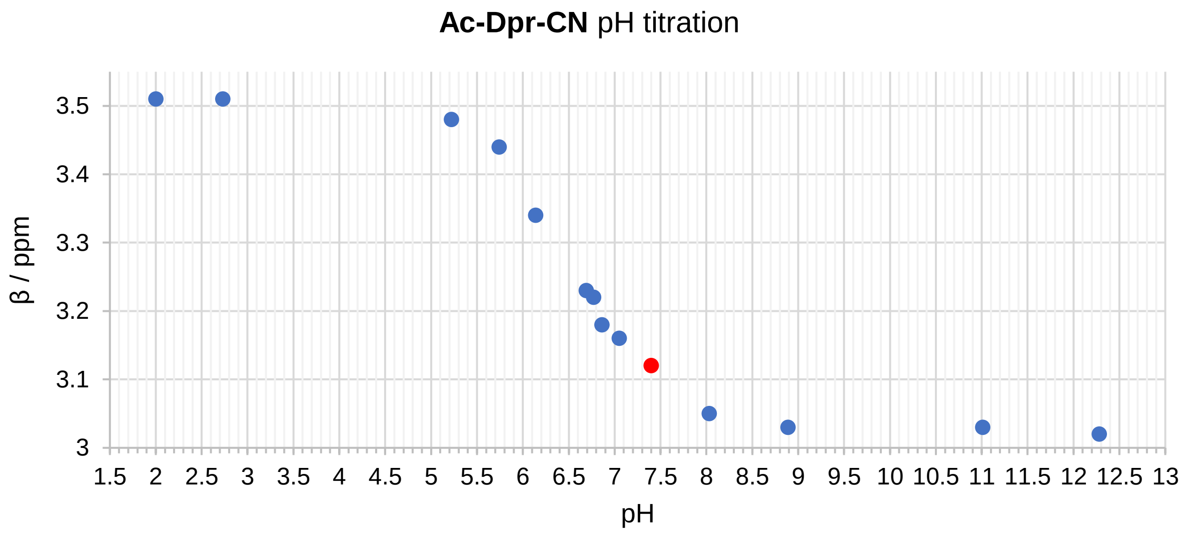

*Supplementary Figure 59. pH titration curve of **Ac-Dpr-CN**. pK_{aH} (β-$CH_2NH_3^+$ → β-CH_2NH_2) was determined to be approximately 6.5. Data points in red are those for which the chemical shift of MSM overlaps with that of the β-proton.*

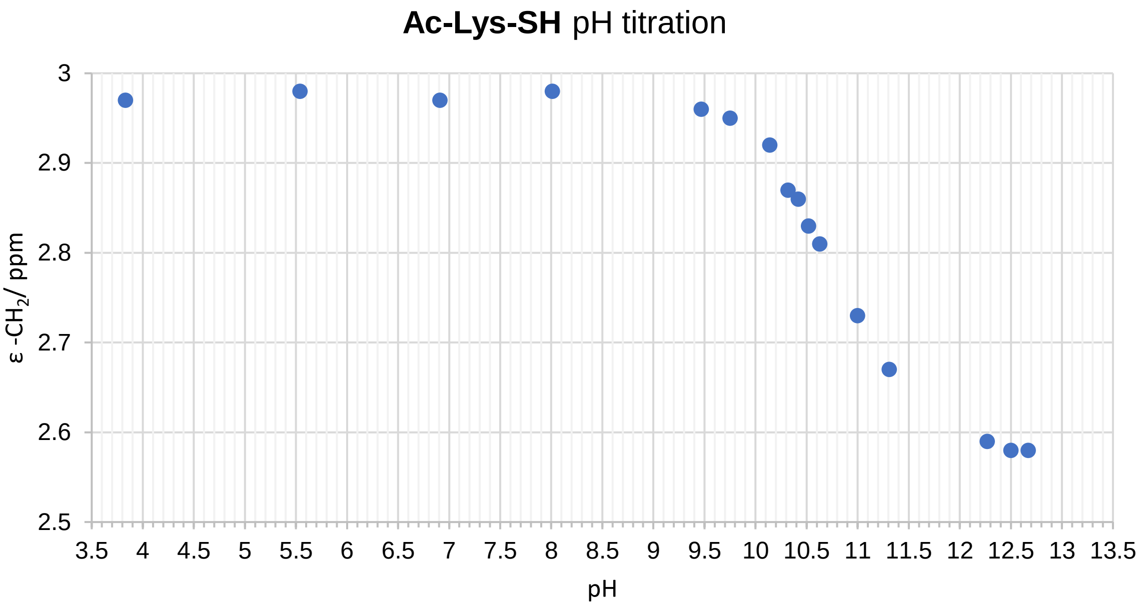

*Supplementary Figure 60. pH titration curve of **Ac-Lys-SH**. pK_{aH} (ε-$CH_2NH_3^+$ → ε-CH_2NH_2) was determined to be approximately 10.8.*

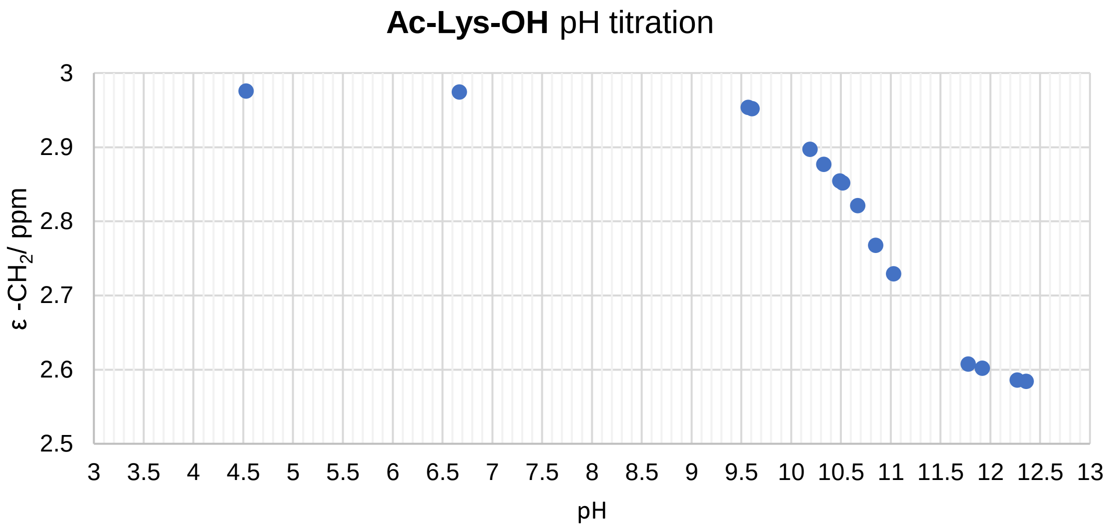

*Supplementary Figure 61. pH titration curve of **Ac-Lys-OH**. pK_{aH} (ε-$CH_2NH_3^+$ → ε-CH_2NH_2) was determined to be approximately 10.8.*

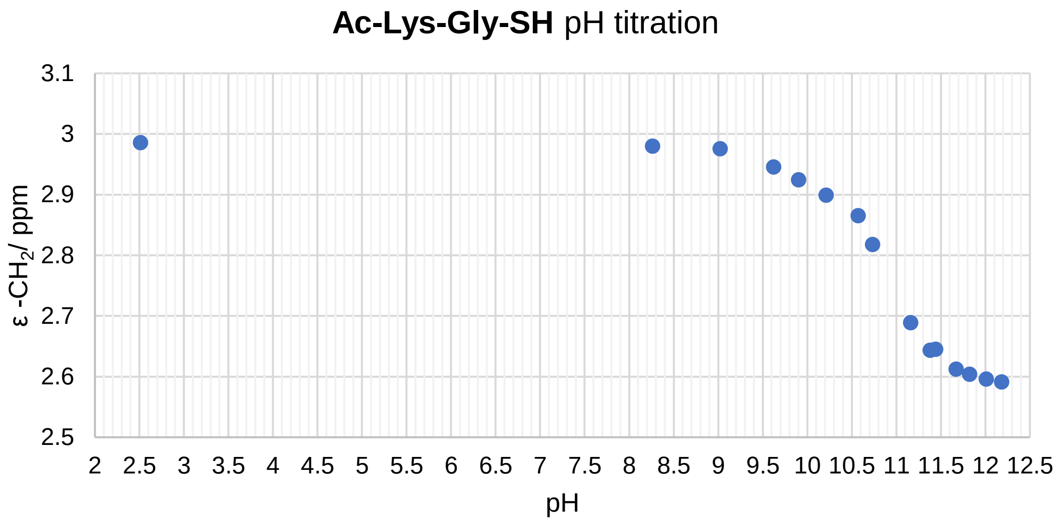

*Supplementary Figure 62. pH titration curve of **Ac-Lys-Gly-SH**. pK_{aH} (ε-$CH_2NH_3^+$ → ε-CH_2NH_2) was determined to be approximately 10.8.*

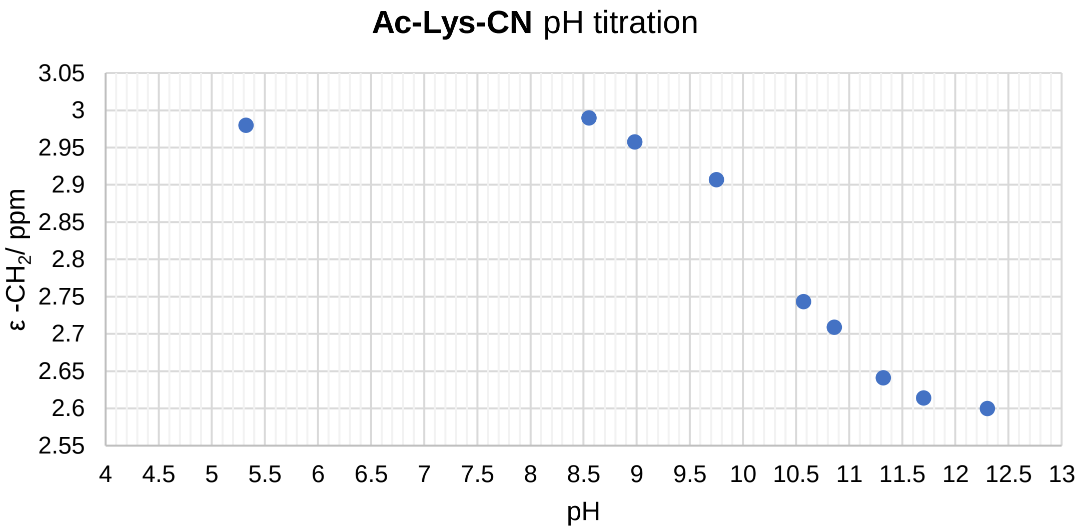

*Supplementary Figure 63. pH titration curve of **Ac-Lys-CN**. pK_{aH} (ε-$CH_2NH_3^+$ → ε-CH_2NH_2) was determined to be approximately 10.4.*

The normalised ^{1}H NMR ω-methylene resonance chemical shift change, $\Delta_{\omega CH_2}$, used in order to compare the pK_{aH} values of Dpr and Lys compounds on the same y-axis, was calculated as follows: $\Delta_{\omega CH_2} = (\delta_{\omega CH_2} - \delta_{min\omega CH_2}) / (\delta_{max\omega CH_2} - \delta_{min\omega CH_2})$ where $\delta_{min\omega CH_2}$ = the chemical shift of the most upfield shifted ω-CH_2NH_2 (found at the highest pH measured), $\delta_{max\omega CH_2}$ = the chemical shift of the most downfield shifted ω-CH_2NH_2 (found at the lowest pH measured) and $\delta_{\omega CH_2}$ is the chemical shift of the ω-methylene resonance at the measured pH.

Prebiotic acylation of amines with Ac-Gly-SH

General procedure D: To **Ac-Gly-SH** (30 mM, 1 equiv.) and the desired amine coupling partner (2 equiv.) at pD 7.5 was added $K_3Fe(CN)_6$ (3 equiv.). The reaction mixture was stirred at room temperature for 30 min, centrifuged and the supernatant readjusted to pD 7.5 for analysis by 1D and 2D NMR spectroscopy.

Acylation of Ac-Dpr-OH

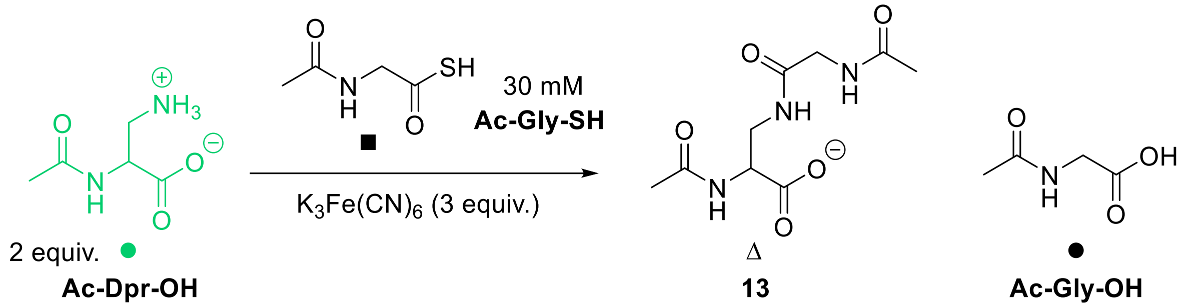

Reaction carried out *via* general procedure **D** using **Ac-Dpr-OH** to afford **13** (20% with respect to **Ac-Gly-SH**).

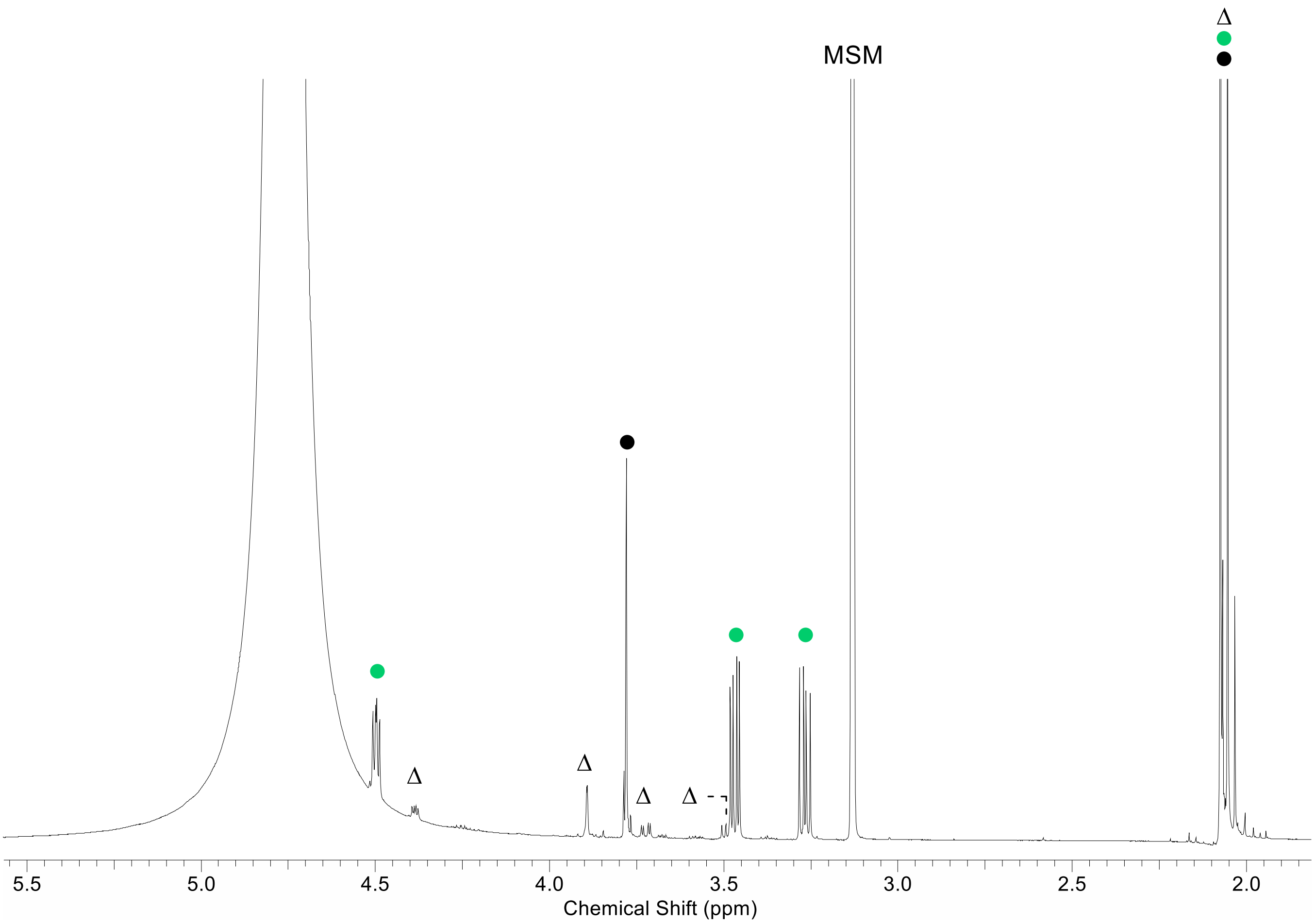

*Supplementary Figure 64. ^1H NMR (700 MHz, D_2O, 1.8 – 5.5 ppm) spectrum showing the reaction of **Ac-Gly-SH**, **Ac-Dpr-OH** (2 equiv.) and $K_3Fe(CN)_6$ (3 equiv.) at pD 7.5.*

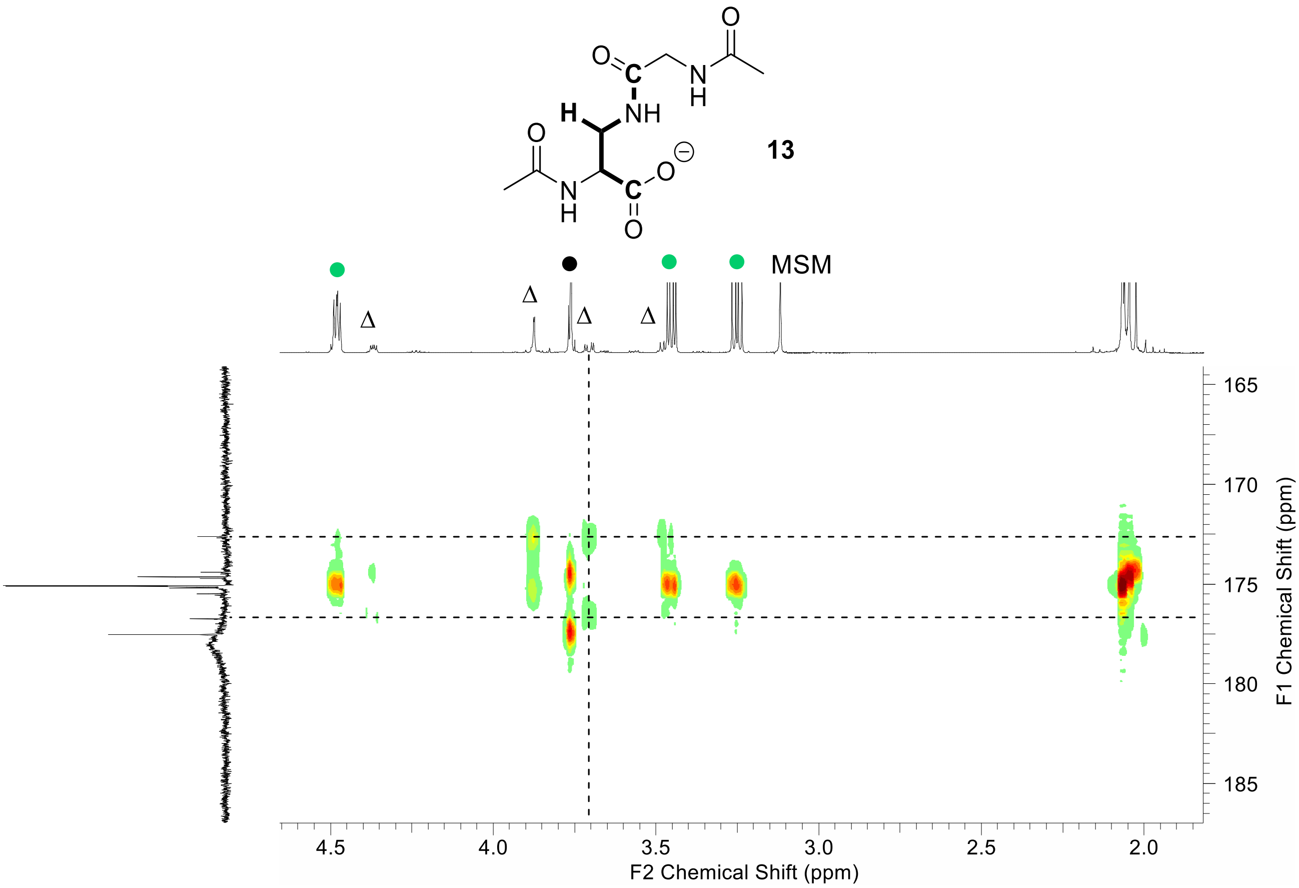

*Supplementary Figure 65. ^{1}H-^{13}C HMBC (^{1}H-700 MHz [1.8–4.6 ppm], ^{13}C-176 MHz [164–186 ppm], D_2O) spectrum showing the two $^{3}J_{CH}$ couplings of Dpr-(C3)–H at 3.70 ppm of **13** to C=O resonances at 172.6 ppm and 176.5 ppm, diagnostic for side-chain amidation.*

13 (Δ): **^{1}H NMR** (700 MHz, D_2O, partial assignment) δ_H 4.37 (ABX, *J* = 7.8, 4.5 Hz, 1H, Dpr-(C2)–H), 3.89 (AB, *J* = 17.2, 1H, Gly-(C2)–H), 3.87 (AB, *J* = 17.2, 1H, Gly-(C2)–H′), 3.71 (ABX, *J* = 13.9, 4.3 Hz, 1H, Dpr-(C3)–H), 3.48 (ABX, *J* = 14.0, 8.0 Hz, 1H, Dpr-(C3)–H′). **^{13}C NMR** (176 MHz, D_2O, partial assignment) δ_C 176.8 (Dpr-C1), 175.5 (Gly-C1), 172.6 (Gly-**C**OCH_3), 55.3 (Dpr-C2), 43.3 (Gly-C2). **HRMS-ESI** $[M+H]^+$ calc. for $C_9H_{16}N_3O_5^+$: 246.1084; obs. 246.1082.

Acylation of Ac-Lys-OH

Reaction carried out *via* general procedure **D** using **Ac-Lys-OH** to afford **14** (6% with respect to **Ac-Gly-SH**).

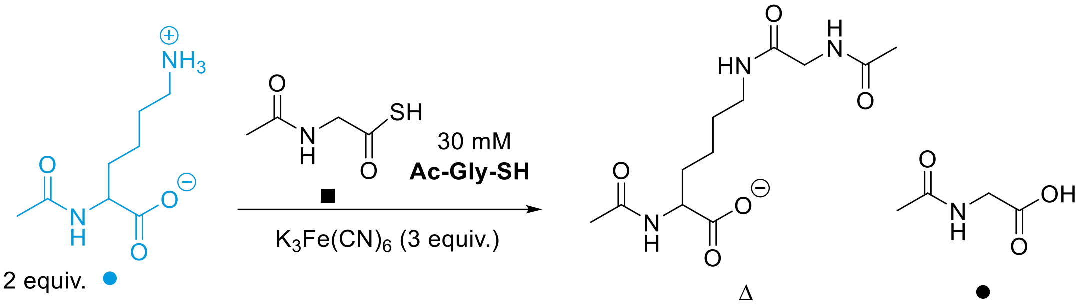

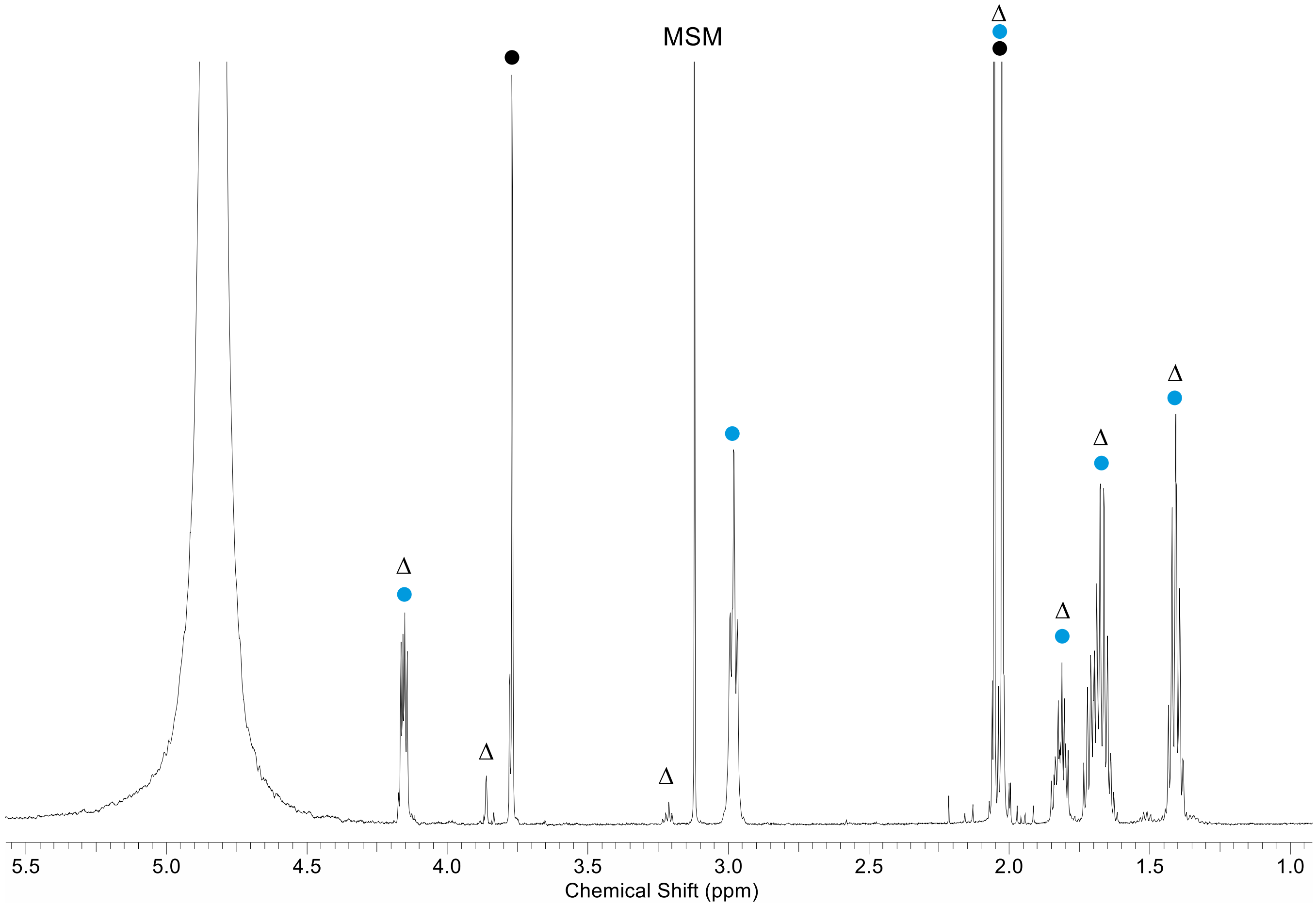

*Supplementary Figure 66. ^{1}H NMR (600 MHz, D_2O, 1.8 – 5.5 ppm) spectrum showing the reaction of **Ac-Gly-SH**, **Ac-Lys-OH** (2 equiv.) and $K_3Fe(CN)_6$ (3 equiv.) at pD 7.5.*

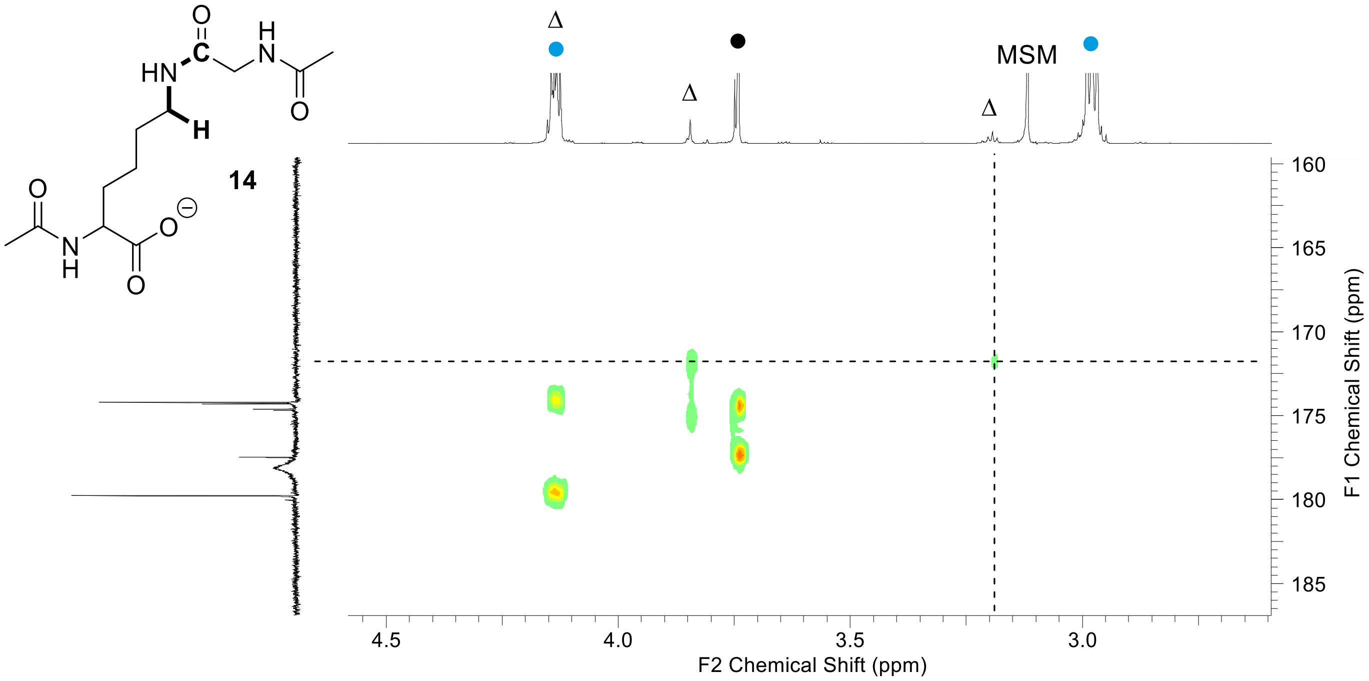

*Supplementary Figure 67. ^{1}H-^{13}C HMBC (^{1}H-700 MHz [2.6–4.7 ppm], ^{13}C-176 MHz [160–186 ppm], D_2O) spectrum showing the $^{3}J_{CH}$ couplings of Lys-(C6)–H at 3.19 ppm of **14** to a C=O resonance at 171.9 ppm, diagnostic for side-chain amidation.*

14 (Δ): **^{1}H NMR** (700 MHz, D_2O, partial assignment) δ_H 3.86 (s, 2H, Gly-(C2)–H_2), 3.21 (t, *J* = 6.9 Hz, 2H, Lys-(C6)–H_2). **HRMS-ESI** $[M+H]^+$ calc. for $C_{12}H_{22}N_3O_5^+$: 288.1554; obs. 288.1556.

Acylation of Ac-Dpr-CN

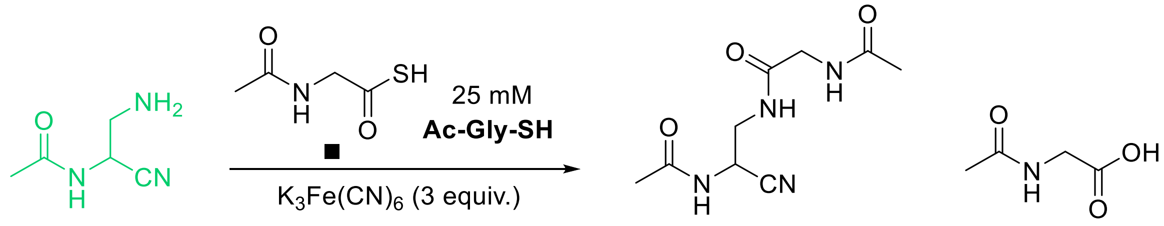

2 equiv. ⊗ **Ac-Dpr-CN** | Δ **15** | ● **Ac-Gly-OH**

Reaction carried out *via* general procedure **D** using **Ac-Dpr-CN** to afford **15** (85% with respect to **Ac-Gly-SH**).

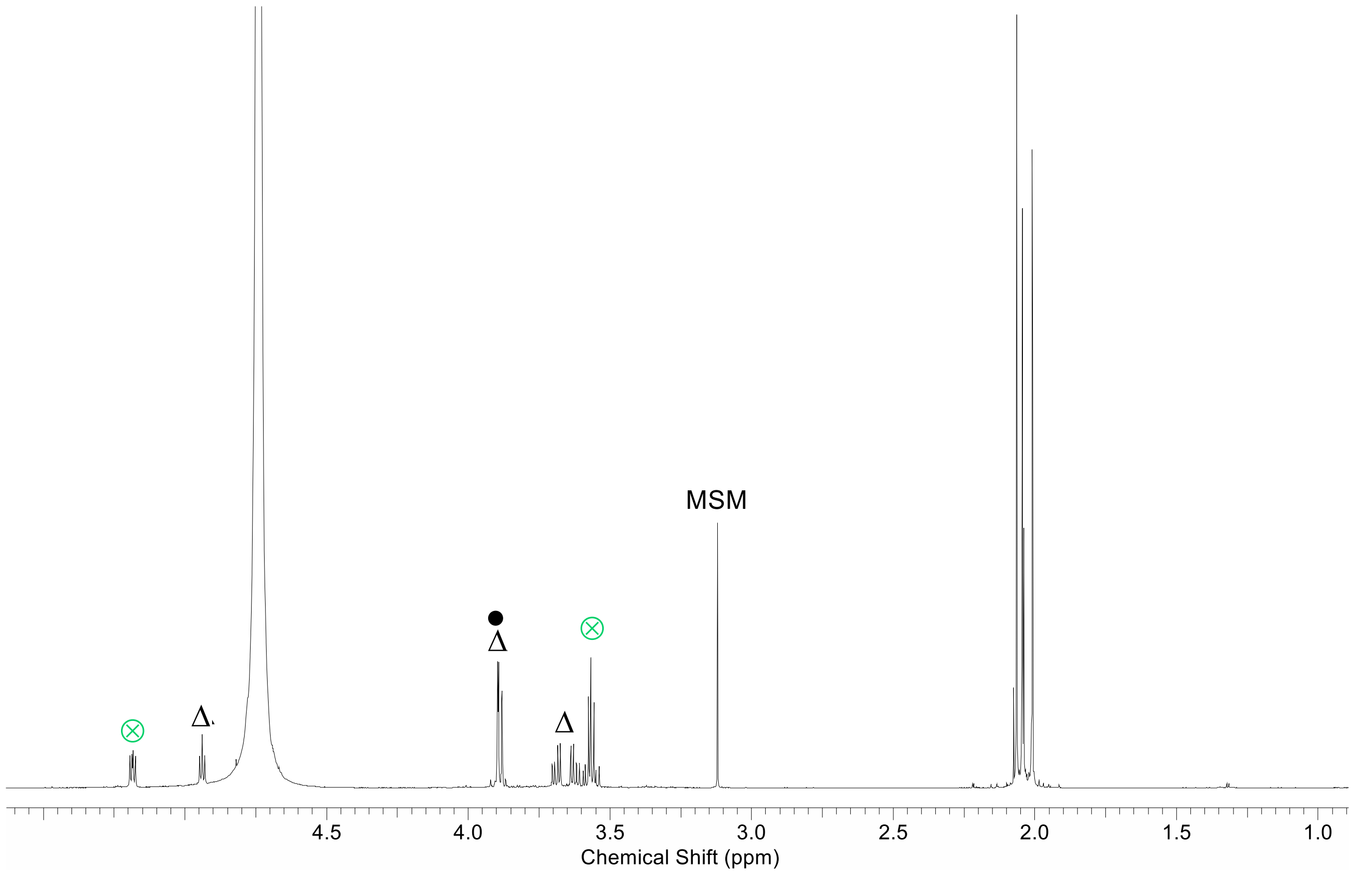

*Supplementary Figure 68. ^{1}H NMR (700 MHz, D_2O, 1.0 – 5.5 ppm) spectrum showing the reaction of **Ac-Gly-SH** (25 mM), **Ac-Dpr-CN** (2 equiv.) and $K_3Fe(CN)_6$ (3 equiv.) at pD 7.5.*

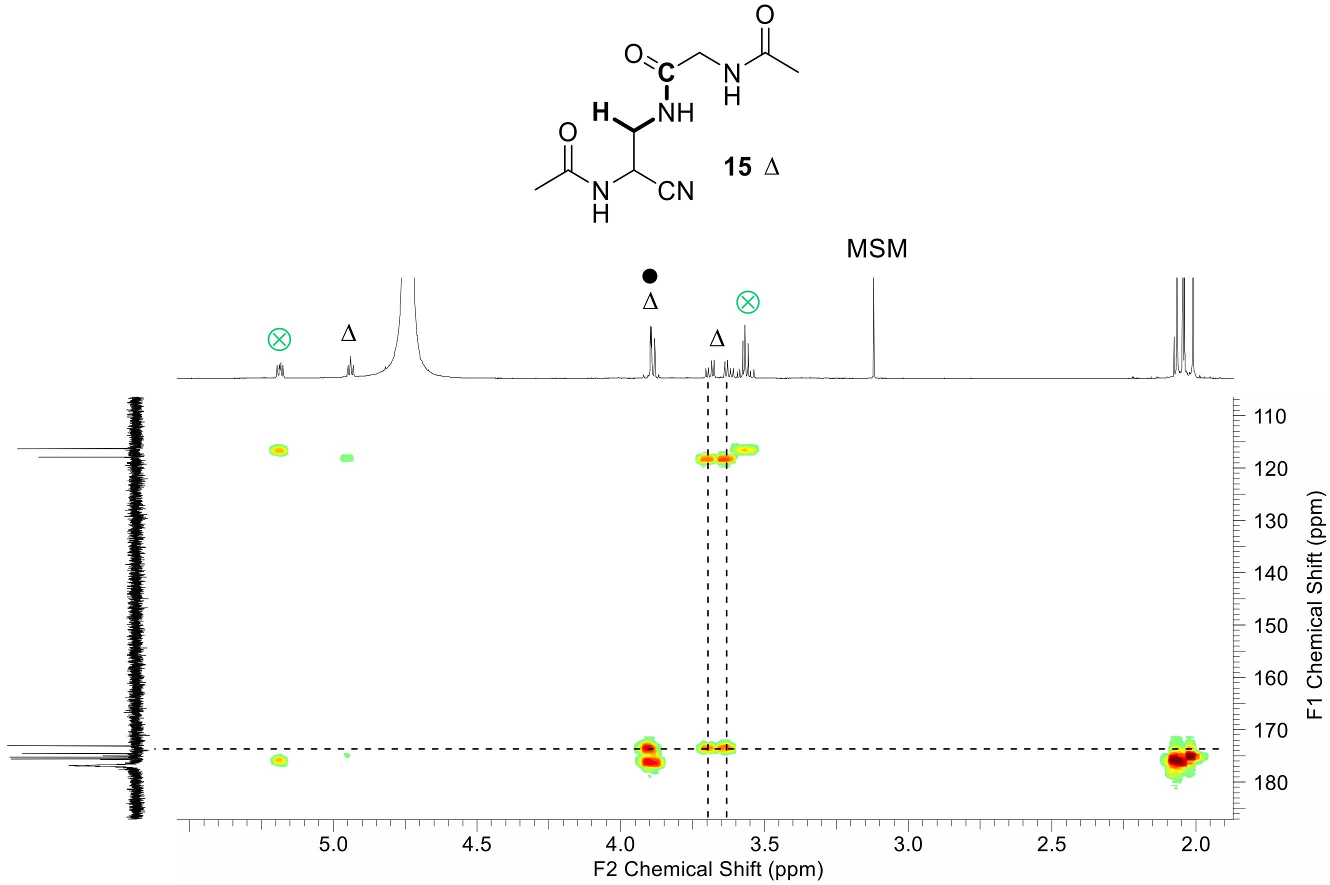

Supplementary Figure 69. ^{1}H-^{13}C HMBC (^{1}H-700 MHz [2.0 – 5.5 ppm], ^{13}C-176 MHz [160–186 ppm], D_2O) spectrum showing the $^{3}J_{CH}$ couplings of Dpr-(C3)–H at 3.69 ppm and 3.62 ppm of ***15*** *to a C=O resonance at 173.0 ppm, diagnostic for side-chain amidation.*

15 (Δ): **^{1}H NMR** (700 MHz, D_2O, partial assignment) δ_H 4.94 (app t, J = 6.6 Hz, 1H, Dpr-(C2)–H), 3.90 (AB, J = 17.2 Hz, 1H, Gly-(C2)–H), 3.89 (AB, J = 17.2 Hz, 1H, Gly-(C2)–H′), 3.69 (ABX, J = 14.0, 6.2 Hz, 1H, Dpr-(C3)–H), 3.62 (ABX, J = 14.0, 6.9 Hz, 1H, Dpr-(C3)–H′). **^{13}C NMR** (176 MHz, D_2O, partial assignment) δ_C 174.6 (Dpr-**C**OCH$_3$), 173.1 (Gly-C1), 118.0 (Dpr-C1), 43.2 (Gly-C2). **HRMS-ESI** $[M+H]^+$ calc. for $C_9H_{15}N_4O_3^+$: 227.1139; obs. 227.1137.

Acylation of Dpr-CN

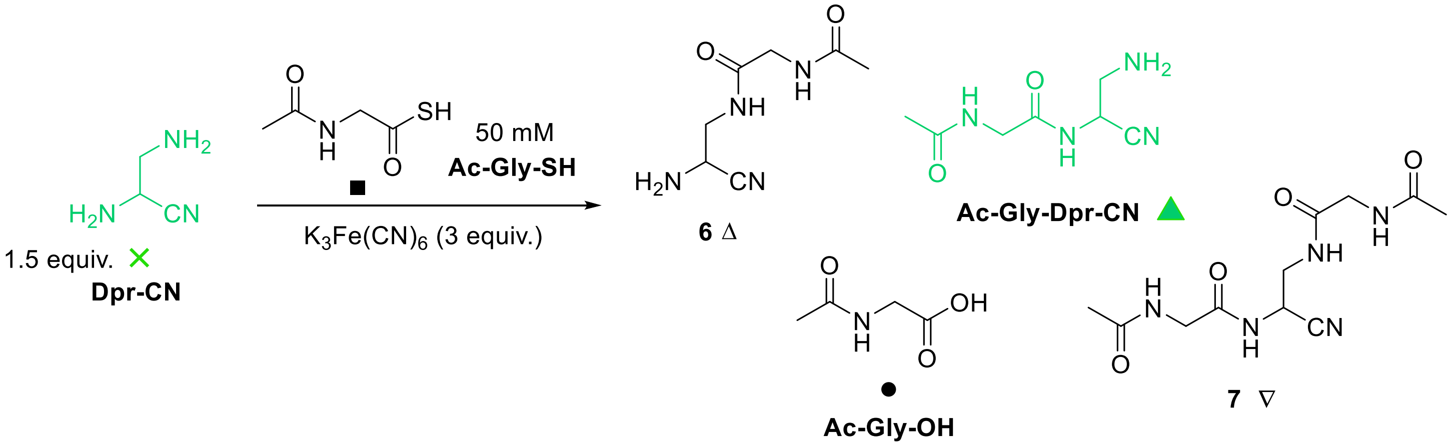

Reaction carried out using **Ac-Gly-SH** (50 mM, 1 equiv.), **Dpr-CN** (1.5 equiv.) and $K_3Fe(CN)_6$ at pD 7.5. The reaction mixture was stirred at room temperature for 30 min, centrifuged and the supernatant readjusted to pD 8.5, 6.8 and 5.5 for analysis by 1D and 2D NMR spectroscopy, yielding **6** (46% with respect to **Ac-Gly-SH**), **Ac-Gly-Dpr-CN** (8% with respect to **Ac-Gly-SH**) and **7** (8% with respect to **Ac-Gly-SH**).

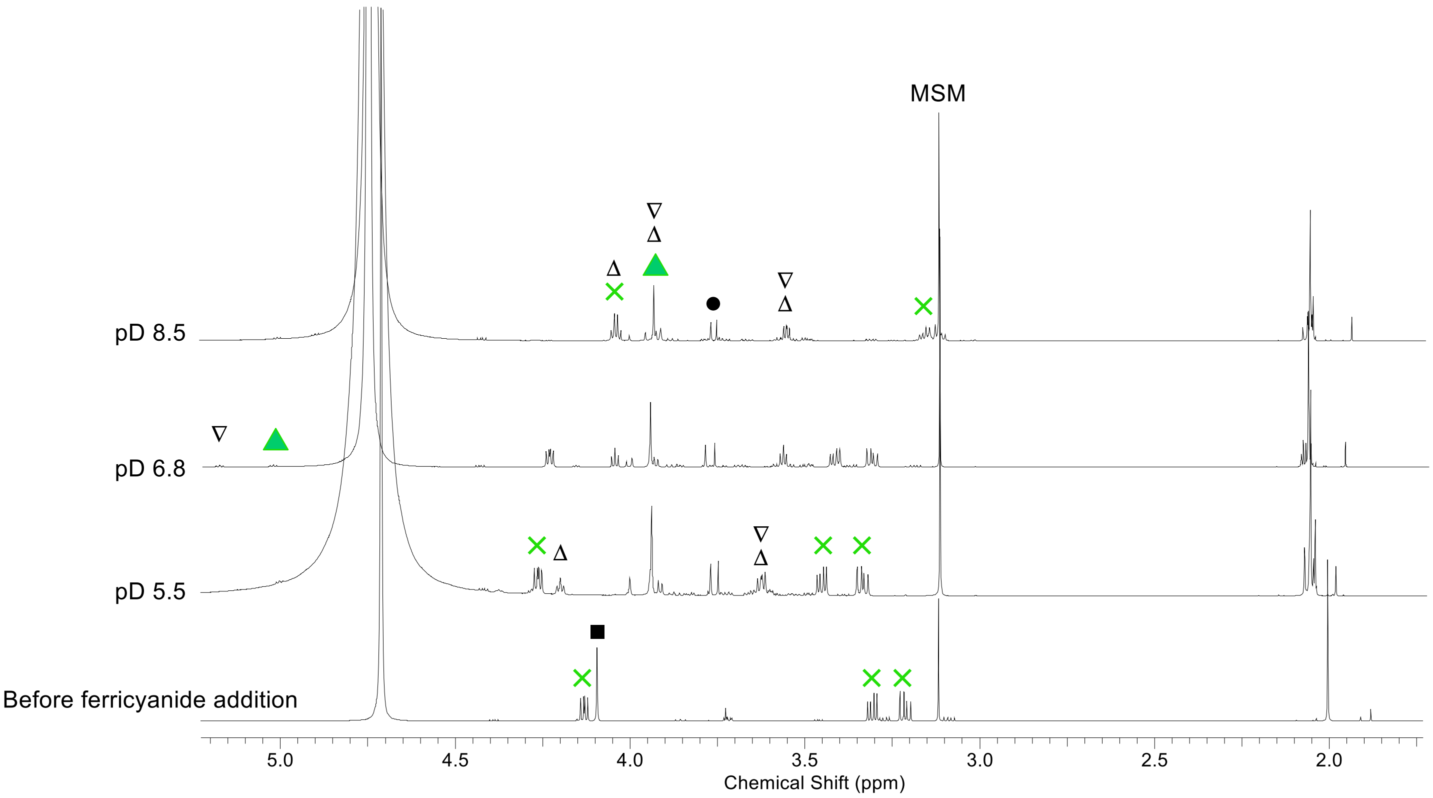

*Supplementary Figure 70. ^{1}H NMR (700 MHz, D_2O, 1.8 – 5.2 ppm) spectrum showing the reaction of **Ac-Gly-SH** (50 mM) and **Dpr-CN** (1.5 equiv.) at pD 7.5 before (bottom spectrum) and after the addition of **$K_3Fe(CN)_6$** (3 equiv.), observed at pD 5.5, 6.8 and 8.5.*

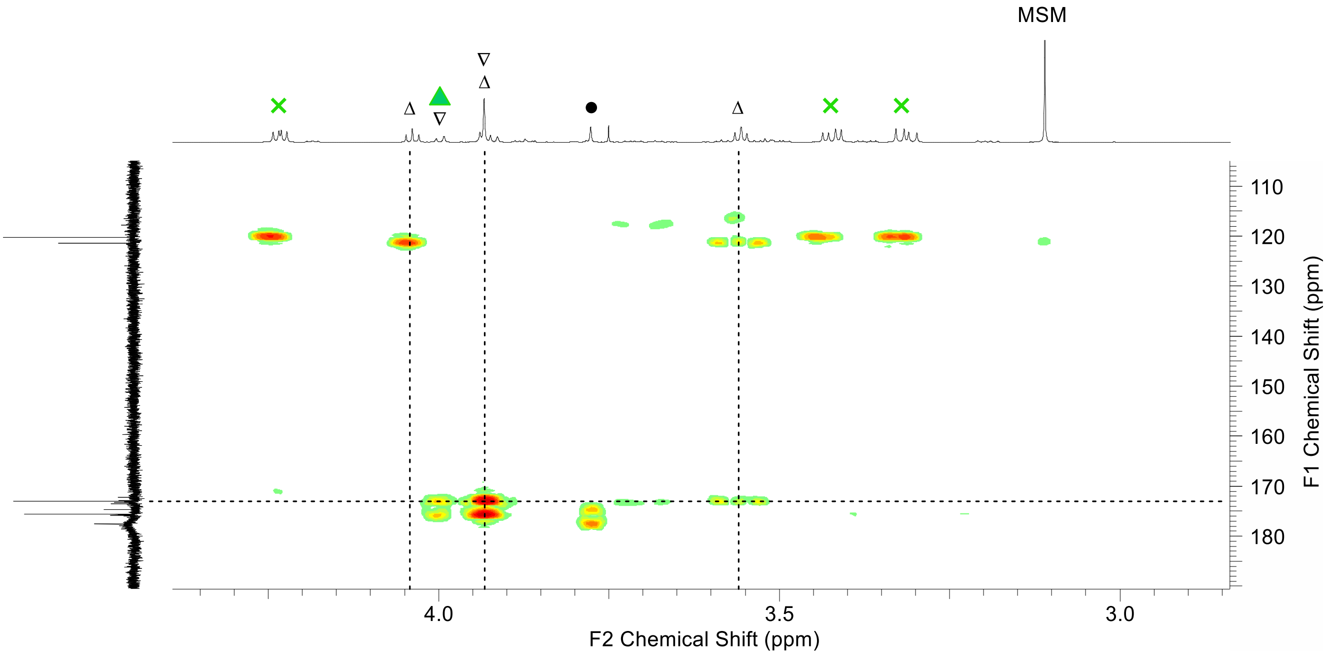

*Supplementary Figure 71. ^{1}H-^{13}C HMBC (^{1}H-700 MHz [2.9–4.3 ppm], ^{13}C-176 MHz [160–190 ppm], D_2O) spectrum showing the $^2J_{CH}$ couplings of Dpr-(C2)–H at 4.04 ppm of **6** to a CN resonance at 121.4 ppm and Gly-(C2)–H_2 at 3.93 ppm to a C=O resonance at 173.0 ppm, and the $^3J_{CH}$ couplings of Dpr-(C3)–H_2 to the same CN resonance and C=O resonance, diagnostic for side-chain amidation.*

6: **^{1}H NMR** (700 MHz, D_2O, partial assignment, pD 5.5) δ_H 4.20 (app. t, J = 6.3 Hz, 1H, Dpr-(C2)–H), 3.94 (app. s, 2H, Gly-(C2)–H_2), 3.60-3.66 (m, 2H, Dpr-(C2)–H_2). **^{13}C NMR** (176 MHz, D2O, partial assignment) δ_C 175.6 (**C**OCH_3), 173.0 (Gly-C1), 121.4 (Dpr-C1). **HRMS-ESI** [M+H]$^+$ calc. for $C_7H_{13}N_4O_2^+$: 185.1033; obs. 185.1030.

Ac-Gly-Dpr-CN: **^{1}H NMR** (700 MHz, D_2O, partial assignment, pD 5.5) δ_H 5.28 (app. t, J = 6.9 Hz, 1H, Dpr-(C2)–H. **HRMS-ESI** [M+H]$^+$ calc. for $C_7H_{13}N_4O_2^+$: 185.1033; obs. 185.1030.

7: **^{1}H NMR** (700 MHz, D_2O, partial assignment, pD 5.5) δ_H 5.39 (app. t, J = 5.4 Hz, 1H, Dpr-(C2)–H). **HRMS-ESI** [M+H]$^+$ calc. for $C_{11}H_{18}N_5O_4^+$: 284.1353; obs. 284.1373.

Prebiotic Synthesis of Ac-Lys-SH from Ac-Lys-CN

Thiolysis of Ac-Lys-CN

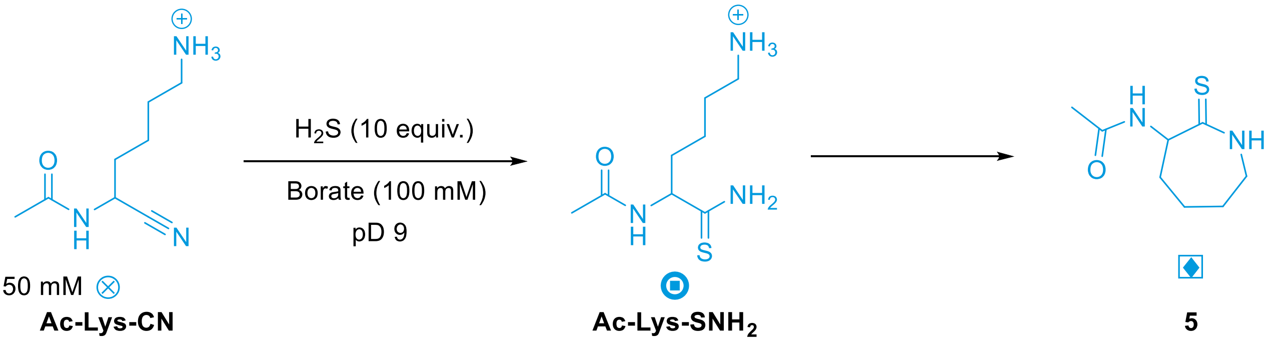

Ac-Lys-CN (50 mM) and NaSH·xH_2O (10 equiv.) were monitored in borate buffer (100 mM, D_2O, pD 9). The solution monitored by 1D and 2D NMR spectroscopy, affording thiolactam **5** (90%) after 2 days.

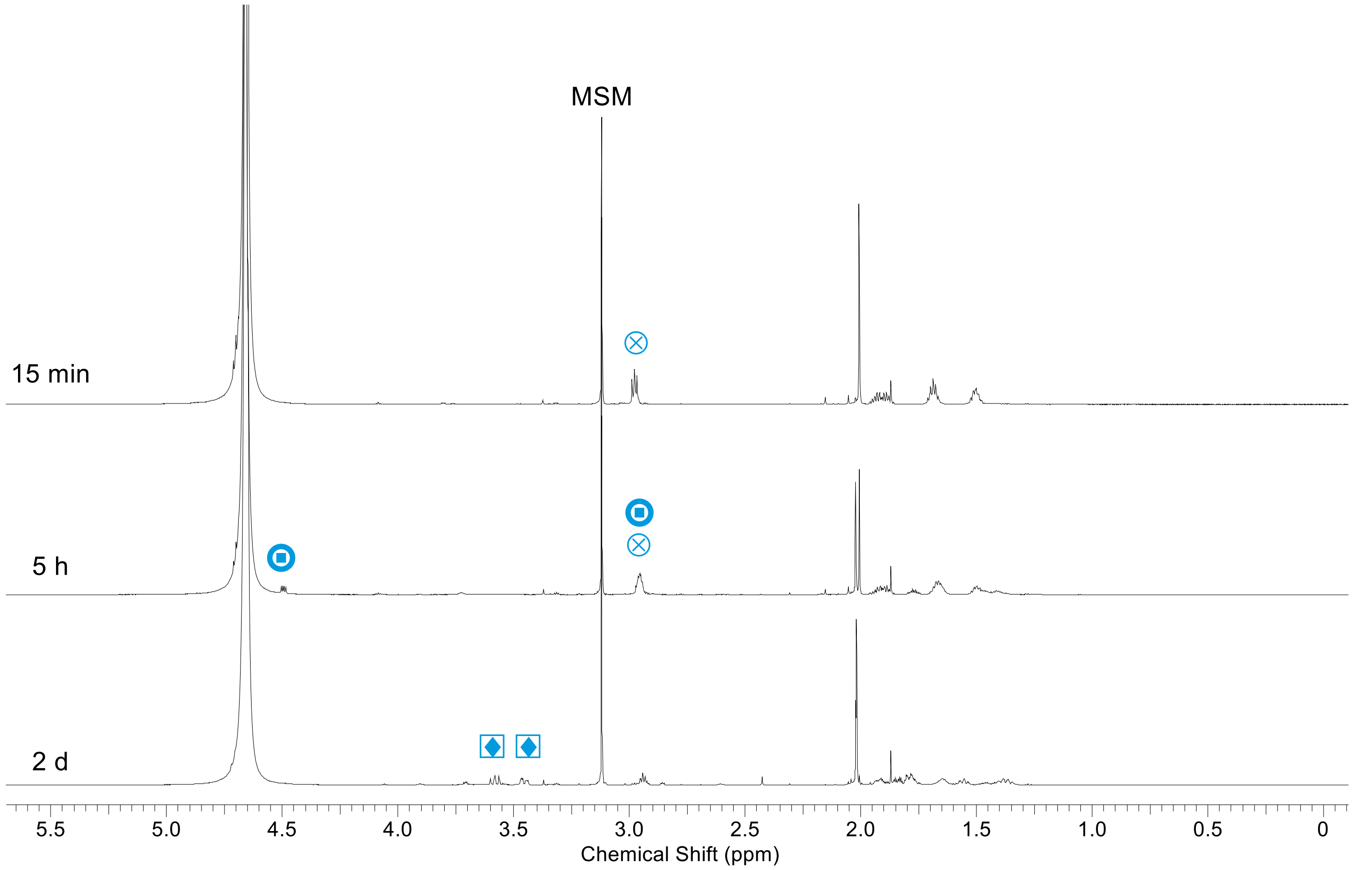

*Supplementary Figure 72. ^{1}H NMR (700 MHz, D_2O, 0.0 – 5.0 ppm) spectra showing the reaction of **Ac-Lys-CN** (50 mM) with NaSH·xH_2O (10 equiv.) in borate buffer (100 mM, D_2O, pD 9) after 15 min (top spectrum), 5 h (middle spectrum) and 2 d (bottom spectrum).*

5 (◆)[4]: **^{1}H NMR** (700 MHz, D_2O, partial assignment) δ_H 3.58-3.62 (m, 1H, (C6)–H), 3.44-3.47 (m, 1H, (C6)–H′). **^{13}C NMR** (176 MHz, D_2O, partial assignment) δ_C 208.0 (C1).

Hydrolysis of Ac-Lys-SNH2

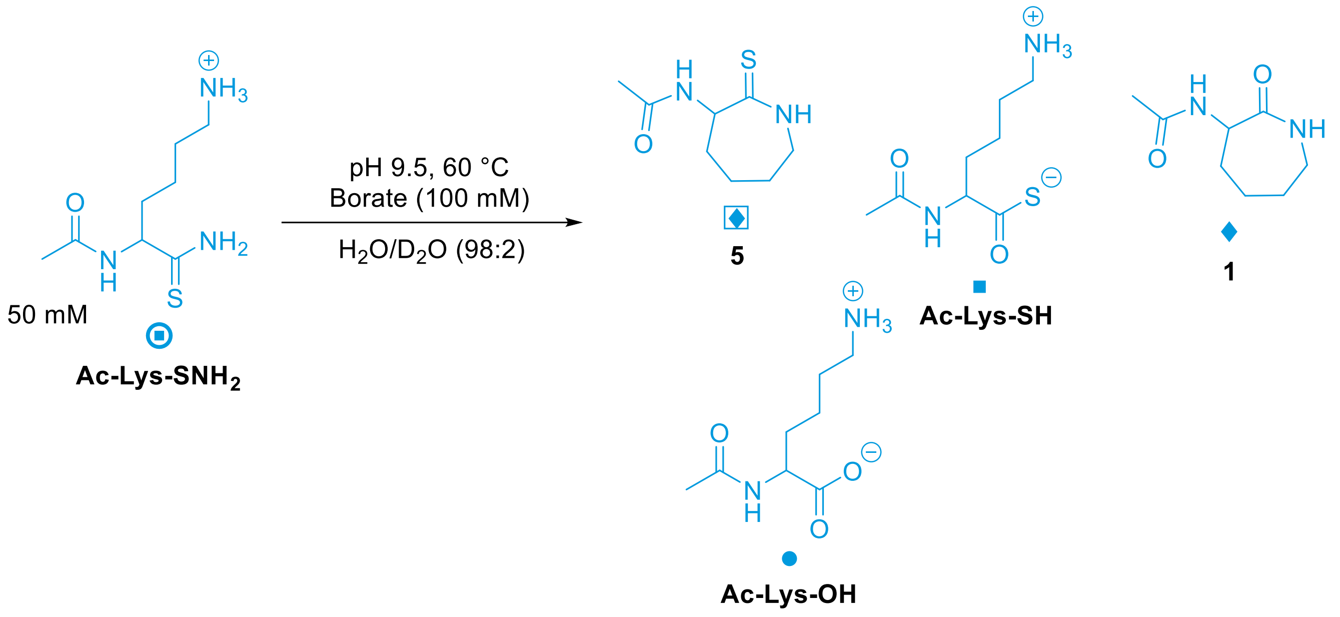

Ac-Lys-SNH2 (50 mM, 1 equiv.) in degassed borate buffer (100 mM, 98:2 H_2O/D_2O, pH 9.5). The reaction mixture was heated at 60 °C in a sealed NMR tube. NMR spectra were periodically acquired. The reaction mixture had a pH of 9.1 after 10 days.

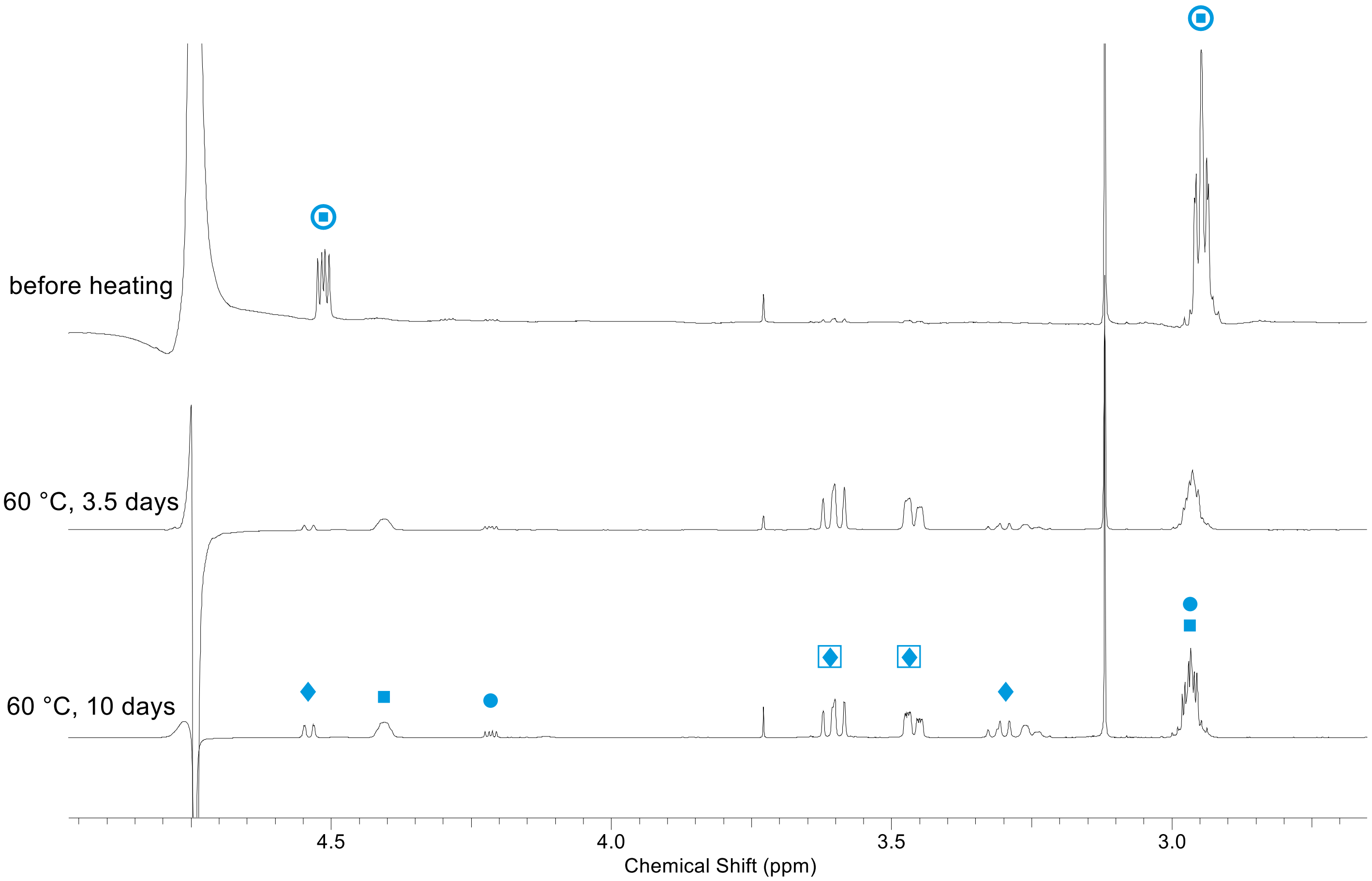

*Supplementary Figure 73. ^{1}H NMR (700 MHz, H_2O/D_2O (98:2), 2.7 – 4.9 ppm) spectra showing the hydrolysis of **Ac-Lys-SNH2** (50 mM) at pH 9.5 buffered with borate (2 equiv.) before heating (top spectrum), after heating at 60 °C for 3.5 d (middle spectrum) and for 10 days (bottom spectrum).*

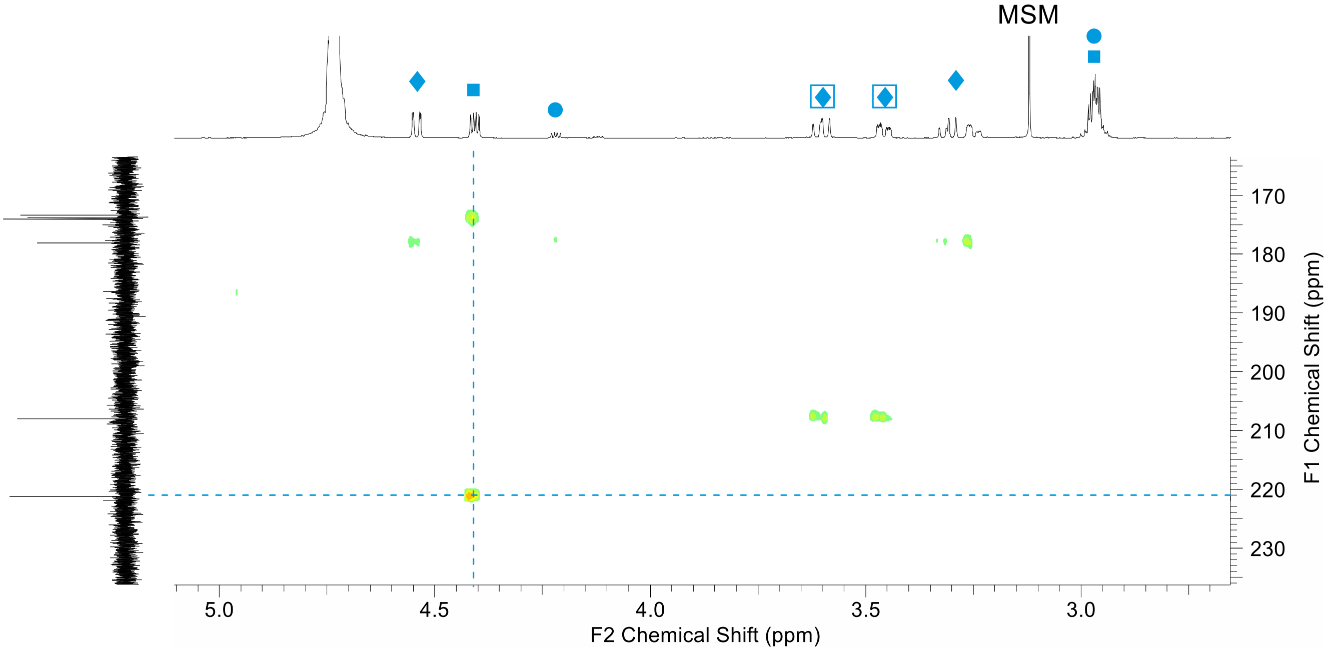

*Supplementary Figure 74. ^{1}H-^{13}C HMBC (^{1}H-700 MHz [2.7–5.1 ppm], ^{13}C-176 MHz [163–236 ppm], D_2O) spectrum showing the reaction mixture following lyophilisation and redissolution in D_2O. The $^{3}J_{CH}$ coupling of Lys-(C2)–H at 4.41 ppm of **Ac-Lys-SH** to a C=O resonance at 221.2 ppm is diagnostic for thioacid formation.*

Entry	pH	Time / d	% **Ac-Lys-SNH$_2$**	% **Ac-Lys-SH**	% **5**	% **1**
1	6.1	4	37	n.d.	39	< 2
2	9.5	10	n.d.	41	30	24
3	10.5	7	n.d.	32	12	25

*Supplementary Table 9. ^{1}H NMR yields for the hydrolysis of **Ac-Lys-SNH$_2$** (50 mM) at 60 °C and at the specified pH.*

Ligation of Ac-Lys-SH formed from the hydrolysis of Ac-Lys-SNH2 with Gly-CN

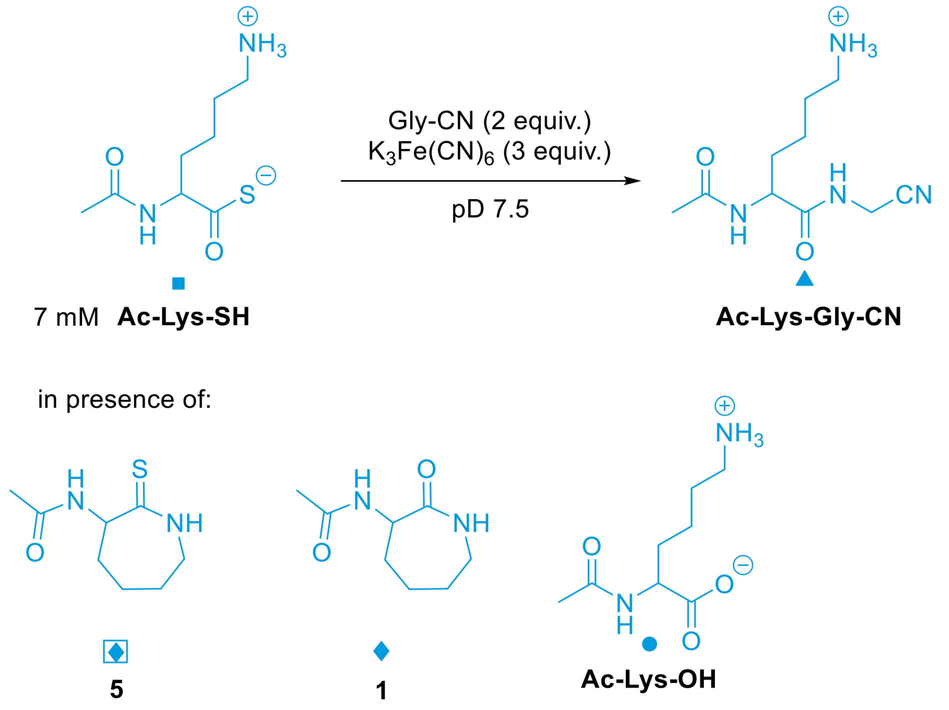

Reaction carried out using **Ac-Lys-SH** (7 mM) from the hydrolysis of **Ac-Lys-SNH2**, **Gly-CN** (2 equiv.) and $K_3Fe(CN)_6$ (3 equiv.) at pD 7.5. The reaction mixture was stirred at room temperature for 30 min and then centrifuged. The supernatant was analysed by 1D and 2D NMR spectroscopy.

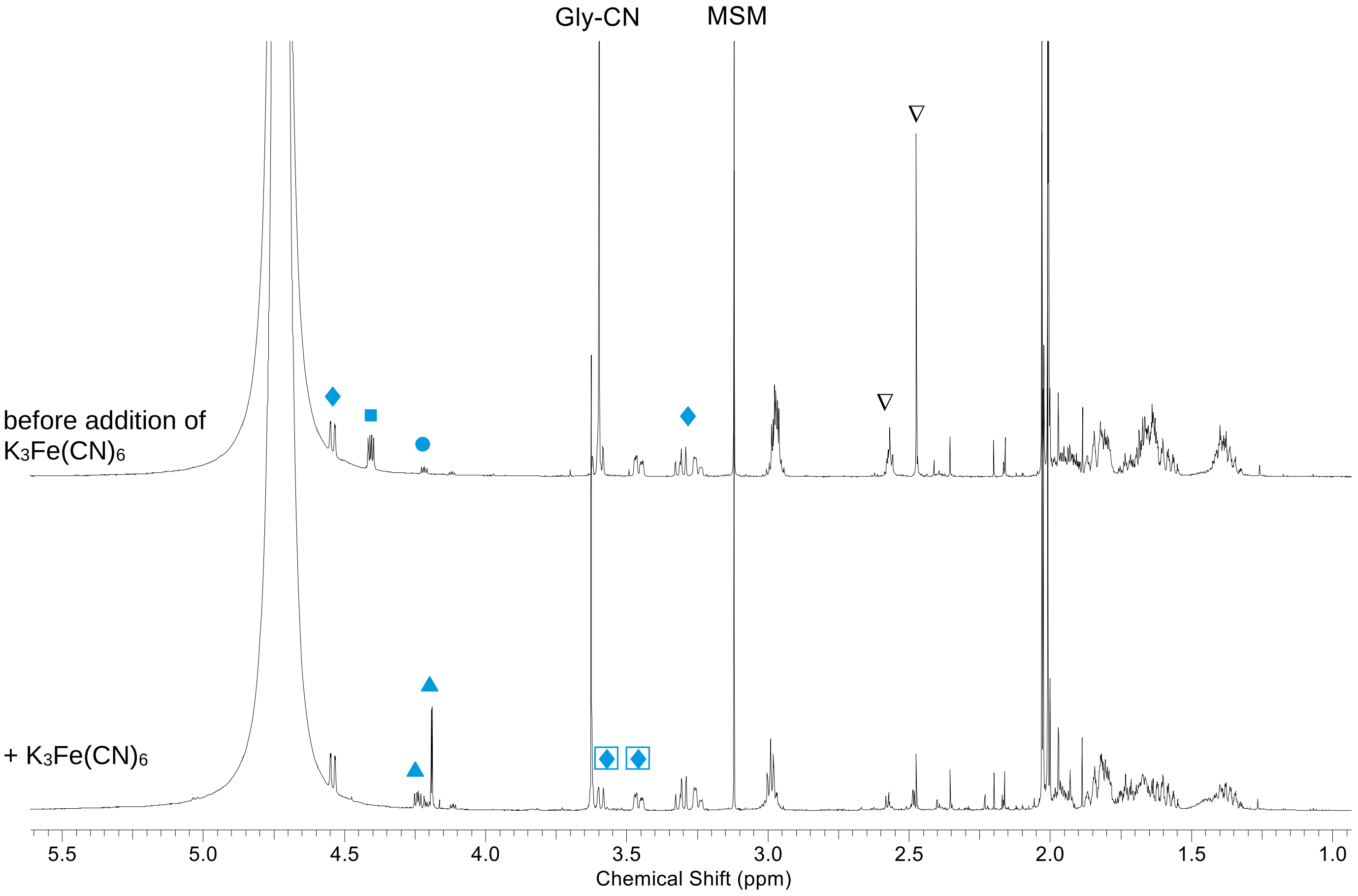

*Supplementary Figure 75. ^{1}H NMR (700 MHz, D_2O, 1.0 – 5.5 ppm) spectra showing the reaction of **Ac-Lys-SH** (7 mM) formed from hydrolysis of **Ac-Lys-SNH2** with Gly-CN (2 equiv.) at pD 7.5 before (top spectrum) and*

after (bottom spectrum, pD readjusted to 7.5) the addition of $K_3Fe(CN)_6$ (3 equiv.). ▽ ***19** from the synthesis of **Ac-Lys-SNH₂**, see Supplementary Figure 156.*

Prebiotic Synthesis of Ac-Dpr-SH from Ac-Dha-CN

Synthesis of Ac-Dpr-CN from Ac-Dha-CN and ammonia

Ac-Dha-CN was prepared according to Foden *et al*.[5] **Ac-Dha-CN** (50 mM) and ammonia were incubated in aqueous solution (see Supplementary Table 10) and monitored by 1D and 2D NMR spectroscopy. If required, residual **Ac-Dha-CN** could be removed from the reaction mixture by washing with $CHCl_3$/^{i}PrOH (7:3, 5 × 0.5 mL).

Entry	Equiv. NH_4Cl	Buffer	Time / h	% **Ac-Dpr-CN**
1	10	None	24	48
2	10	None^{i}	3	76
3	10	Borate (10 equiv.)	3	85
4	10	Borate (5 equiv.)	120^{ii}	67
5	5	Borate (5 equiv.)	3	81
6	3	Borate (3 equiv.)	3	57
7	10	Carbonate (10 equiv.)	3	82^{iii}

*Supplementary Table 10.The reaction of **Ac-Dha-CN** (50 mM) with NH_4Cl at pH 9.0 and 60 °C. ^{i}pH readjusted from 8.9 to 9 at 1.5 h; ^{ii} Reaction performed at room temperature. ^{iii} Combined yield of **Ac-Dpr(CO_2)-CN** and **Ac-Dpr-CN**. **Ac-Dpr(CO_2)-CN** forms by reversible β–amine carboxylation of **Ac-Dpr-CN** in carbonate buffer. A 10.7:1 ratio of **Ac-Dpr(CO_2)-CN/Ac-Dpr-CN** is observed at pH 9.*

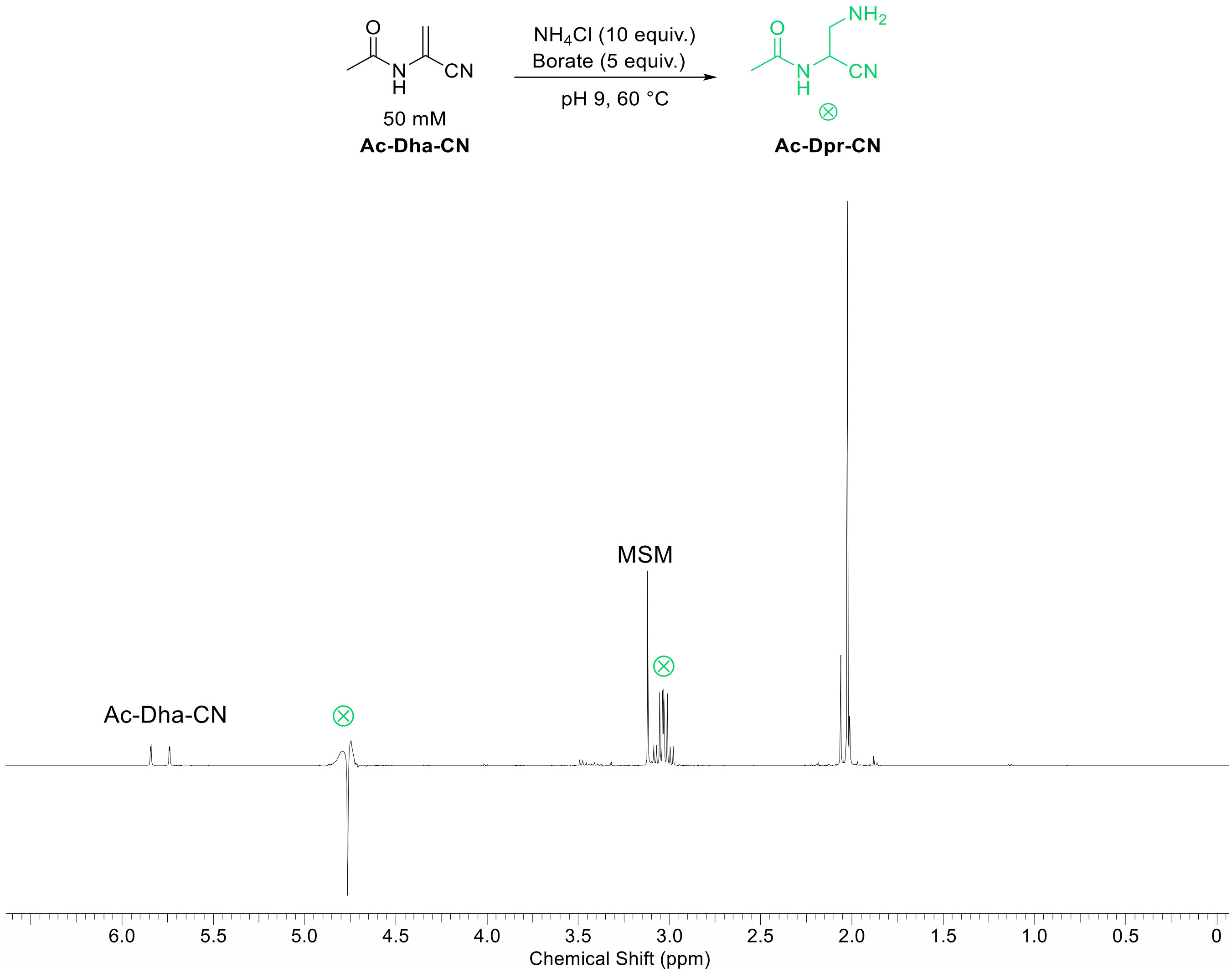

*Supplementary Figure 76. ^{1}H NMR (400 MHz, 99:1 H_2O/D_2O, 0.0 – 6.5 ppm, noesygppr1d) spectrum of **Ac-Dpr-CN** formed from the reaction of **Ac-Dha-CN** (50 mM), ammonia (10 equiv.) in borate buffer (250 mM, 99:1 H_2O/D_2O, pH 9).*

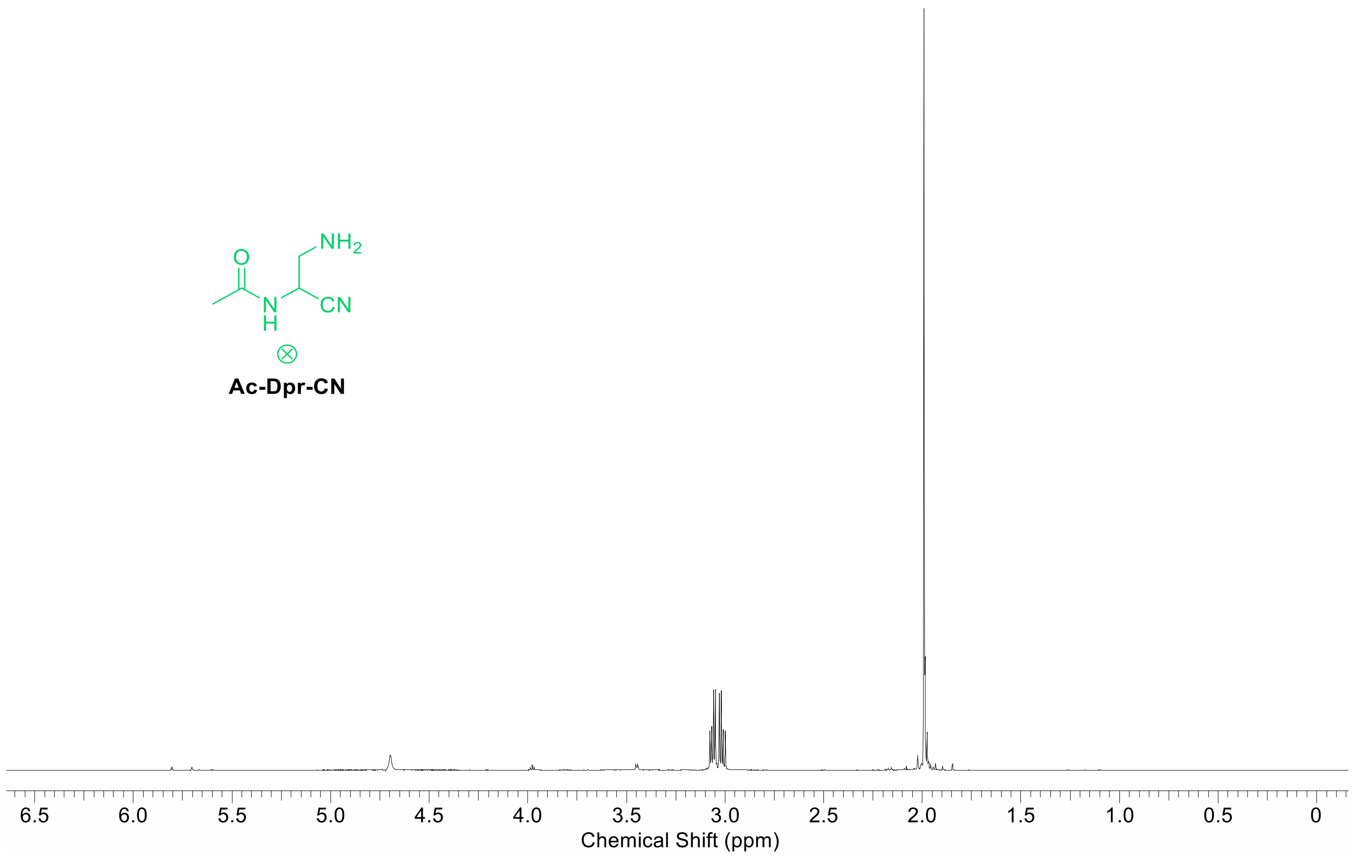

*Supplementary Figure 77. ^{1}H NMR (700 MHz, 98:2 H_2O/D_2O, 0.0 – 6.5 ppm, noesygppr1d) spectrum of **Ac-Dpr-CN** formed from the reaction of **Ac-Dha-CN** (50 mM), ammonia (10 equiv.) and borate (10 equiv.) at pH 9 following washing with $CHCl_3/^{i}PrOH$ to remove residual **Ac-Dha-CN**.*

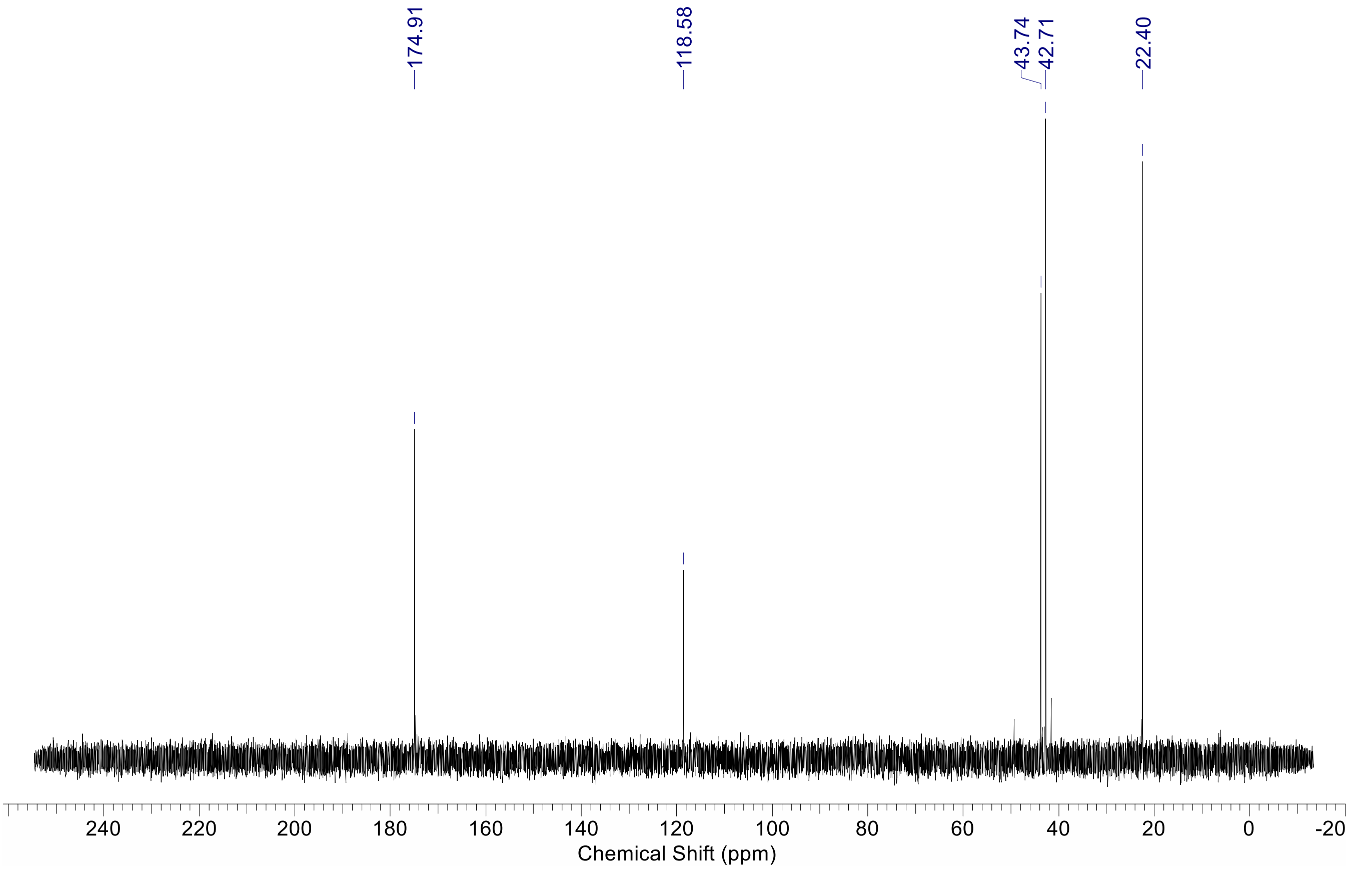

*Supplementary Figure 78. ^{13}C NMR (176 MHz, 98:2 H_2O/D_2O, -20 – 240 ppm) spectrum of **Ac-Dpr-CN** formed from the reaction of **Ac-Dha-CN** (50 mM), ammonia (10 equiv.) and borate (10 equiv.) at pH 9 following washing with $CHCl_3/^{i}PrOH$ to remove residual **Ac-Dha-CN**.*

Ac-Dpr-CN: **^{1}H NMR** (700 MHz, 98:2 H_2O/D_2O, noesygppr1d, partial assignment) δ_H 3.06 (ABX, *J* = 13.4, 6.0 Hz, 1H, Dpr-(C3)–H), 3.01 (ABX, *J* = 13.4, 6.9 Hz, 1H, Dpr-(C3)–H'), 1.99 (s, 3H, $COCH_3$). **^{13}C NMR** (176 MHz, D_2O) δ_C 174.9 (**C**OCH_3), 118.6 (Dpr-C1), 43.7 (Dpr-C2), 42.7 (Dpr-C3), 22.4 (CO**C**H_3). **HRMS-ESI** $[M+H]^+$ calc. for $C_5H_{10}N_3O^+$: 128.0818; obs. 128.0820.

Competition between Ac-Dha-CN, Ac-Dha-OH and ammonia

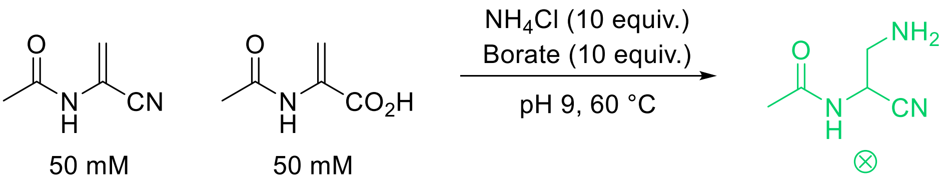

Ac-Dha-CN (50 mM, 1 equiv.), **Ac-Dha-OH** (50 mM) and ammonia (10 equiv.) in borate buffer (500 mM, 9:1 H_2O/D_2O, pH 9.0). The reaction mixture heated at 60 °C and periodically monitored by 1D and 2D NMR spectroscopy, yielding **Ac-Dpr-CN** (77%). No **Ac-Dpr-OH** was detected by NMR spectroscopy.

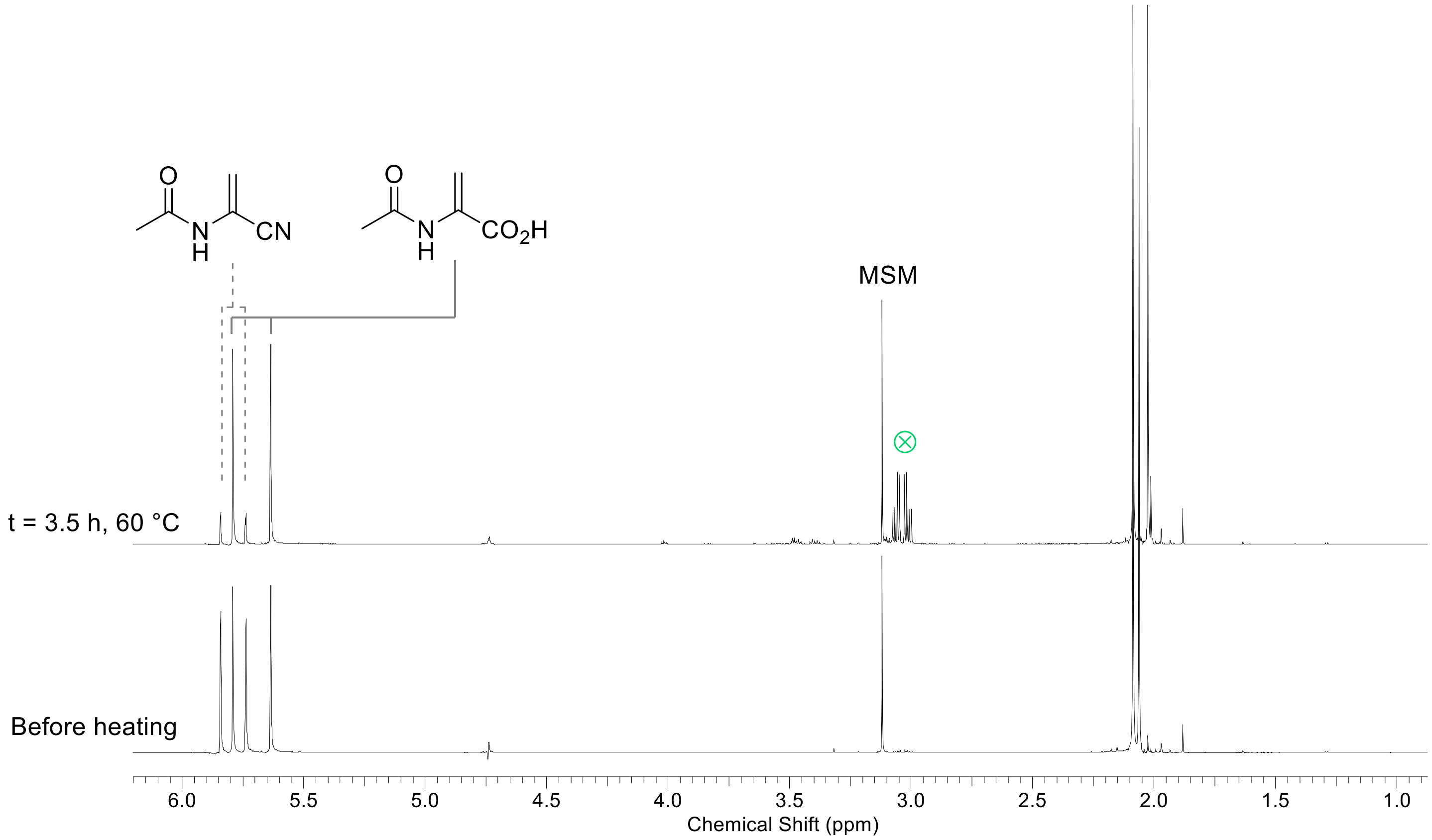

*Supplementary Figure 79. ^{1}H NMR (700 MHz, 9:1 H_2O/D_2O, 1.0 – 6.0 ppm, noesygppr1d) spectra to show the reaction of **Ac-Dha-CN** (50 mM), **Ac-Dha-OH** (1 equiv.) and **NH_4Cl** (10 equiv.) buffered at pH 9 with borate (10 equiv.) before (bottom spectrum) and after (top spectrum) heating at 60 °C for 3.5 h.*

Thiolysis and hydrolysis of Ac-Dpr-CN to form Ac-Dpr-SH

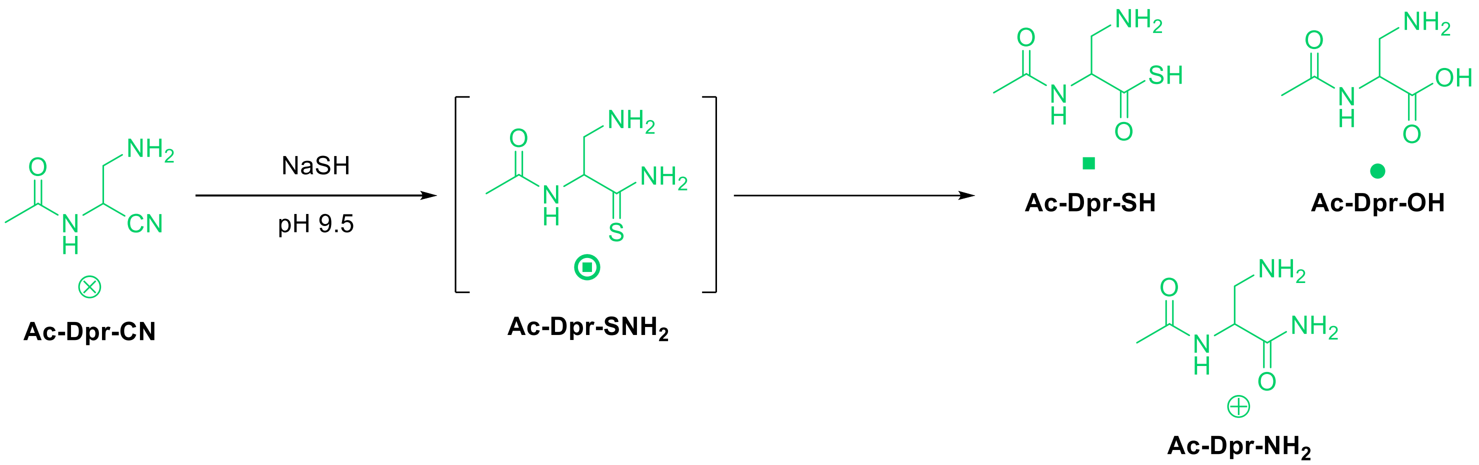

Ac-Dpr-CN (50 mM, 1 equiv.) and NaSH·×H_2O (10 equiv.) in degassed H_2O/D_2O (9:1) at pH 9.5 was monitored by 1D and 2D NMR spectroscopy, yielding **Ac-Dpr-SH** (45%). The solution was measured as pH 11.1 after 24 h.

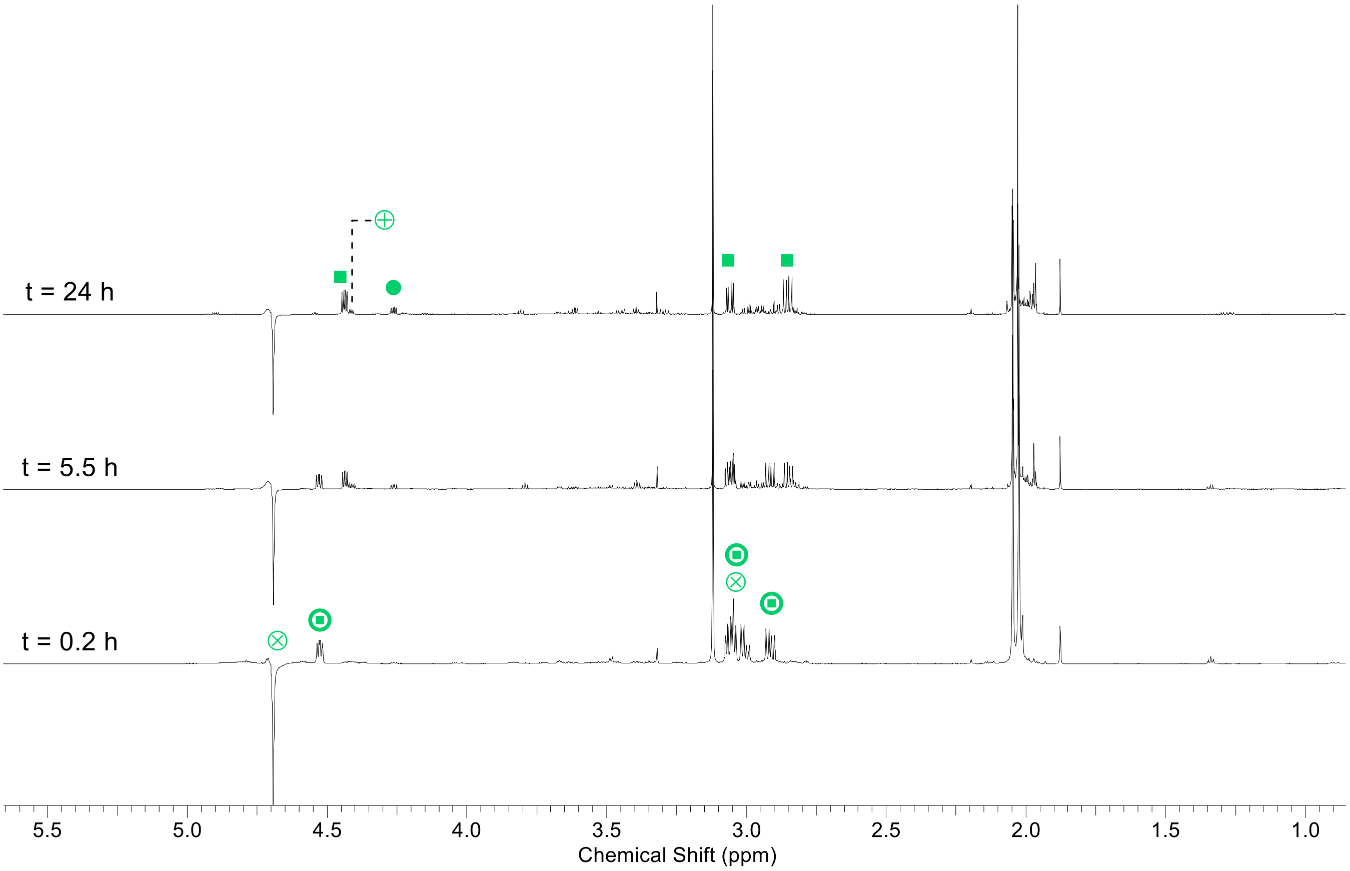

*Supplementary Figure 80. ^{1}H NMR (700 MHz, 9:1 H_2O/D_2O, 1.0 – 5.5 ppm, noesygppr1d) spectra to show the reaction of **Ac-Dpr-CN** (50 mM) and H_2S (10 equiv.) at pH 9.5 after 0.2 h (bottom spectrum), 5.5 h (middle spectrum) and 24 h (top spectrum).*

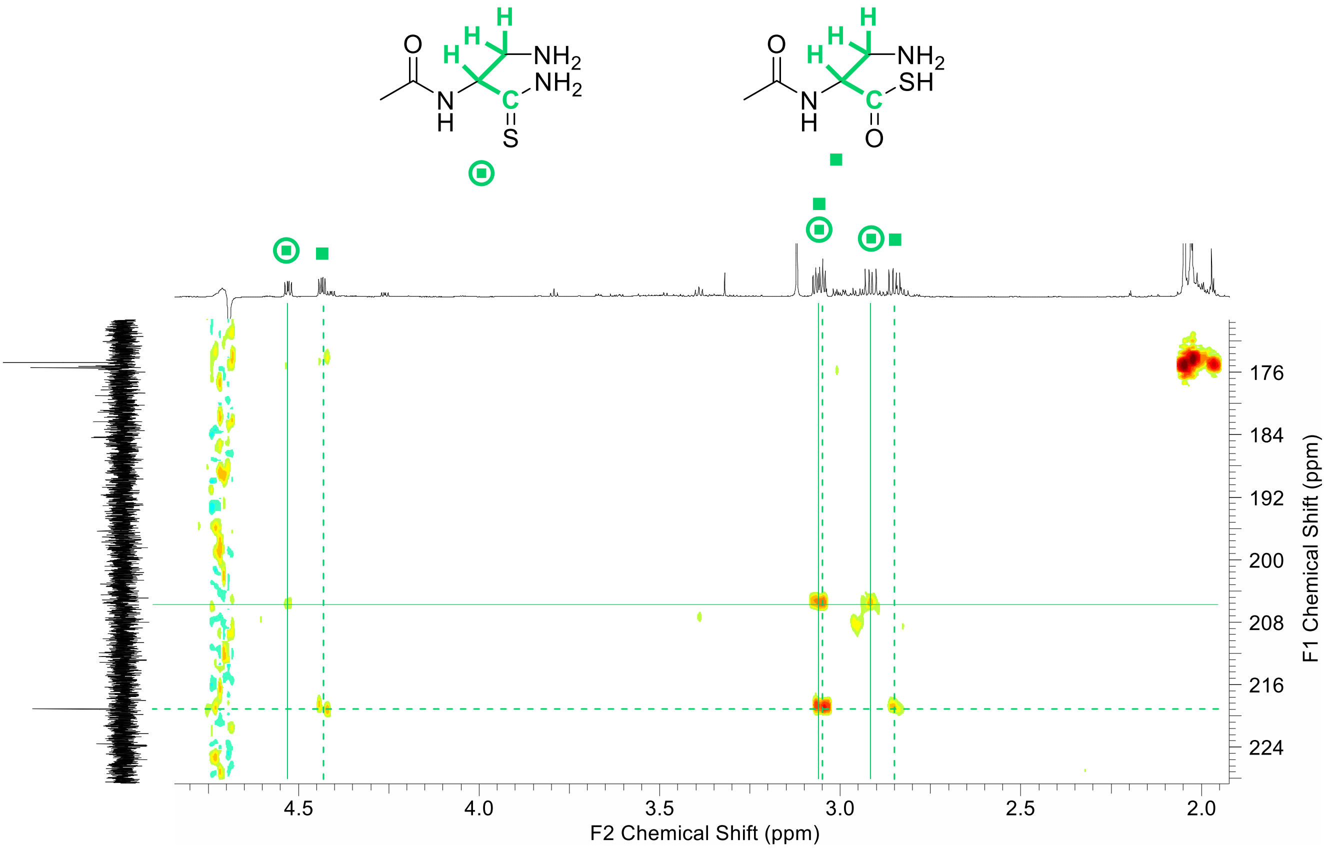

*Supplementary Figure 81. 5.5 h ^{1}H-^{13}C HMBC (^{1}H-700 MHz [2.0 – 4.4 ppm], ^{13}C-176 MHz [170 – 228 ppm], 9:1 H_2O/D_2O) spectrum showing the reaction of **Ac-Dpr-CN** (50 mM) and H_2S (10 equiv.) at pH 9.5 after 0.5 h. The $^{2}J_{CH}$ and $^{3}J_{CH}$ couplings of Dpr-(C2)–H at 4.54 ppm and Dpr-(C3)–H at 2.90 ppm of **Ac-Dpr-SNH₂** to the same C=O resonance at 206 ppm is diagnostic for thioamide formation. The $^{2}J_{CH}$ and $^{3}J_{CH}$ couplings of Dpr-(C2)–H at 4.45 ppm and Dpr-(C3)–H at 2.85 ppm of **Ac-Dpr-SH** to the same C=O resonance at 219 ppm is diagnostic for thioacid formation.*

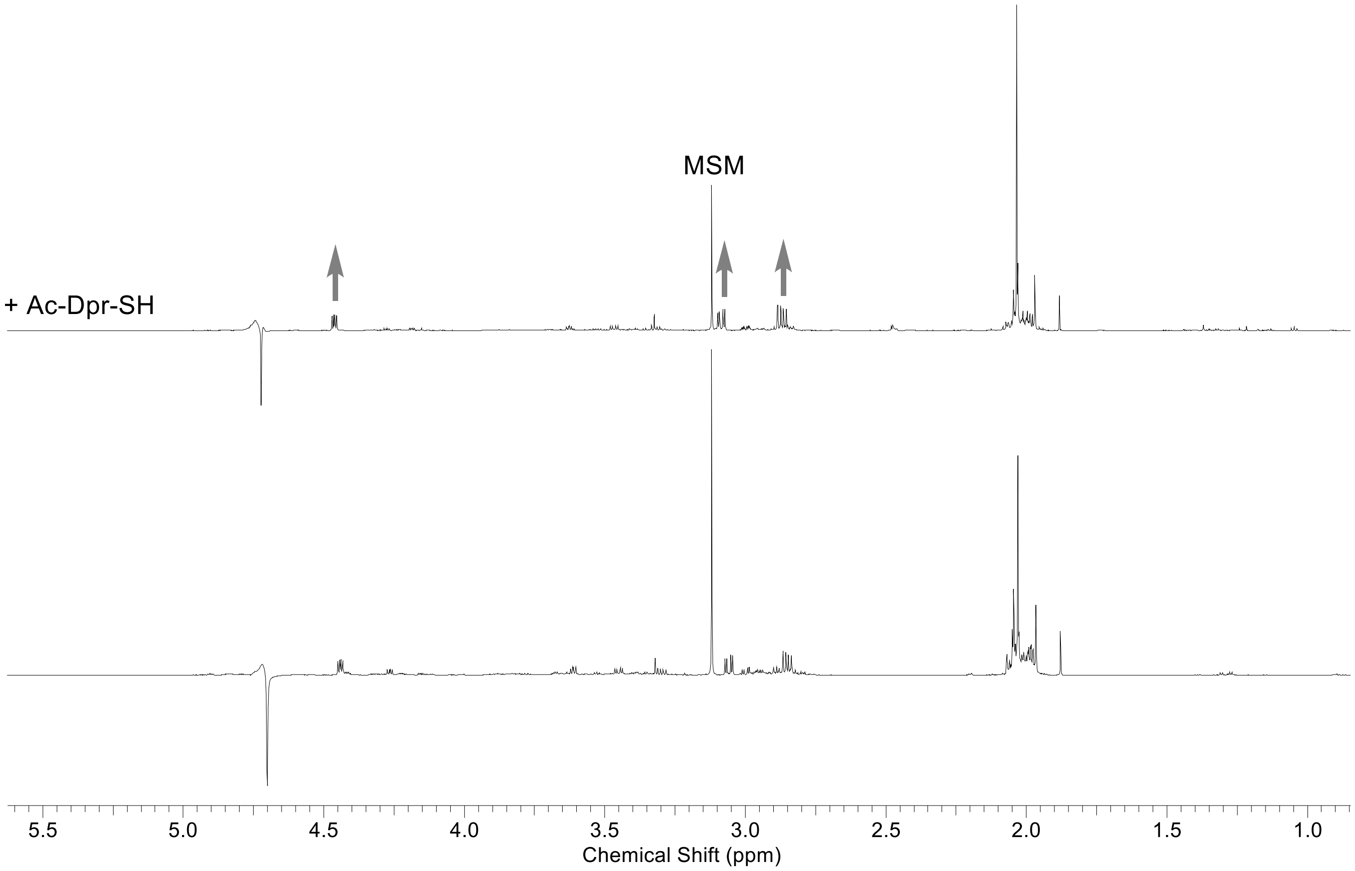

*Supplementary Figure 82. ^{1}H NMR (700 MHz, 9:1 H_2O/D_2O, 1.0 – 5.5 ppm, noesygppr1d) spectra to show the reaction of **Ac-Dpr-CN** (50 mM) and H_2S (10 equiv.) at pH 9.5 after 24 h before (bottom spectrum) and after (top spectrum) spiking with authentic **Ac-Dpr-SH**.*

Amine-catalysed decarboxylation of acetoacetate

Lithium acetoacetate (50 mM, 1 equiv.) was added to the relevant amine catalyst (0.1 equiv. or 0.5 equiv.) and a known amount of MSM in either sodium acetate, imidazole or borate buffer (150 mM, H_2O, pH 5, 7 or 9). The reaction mixture was monitored by 1D and 2D NMR spectroscopy.

Decarboxylation of acetoacetate at pH 5

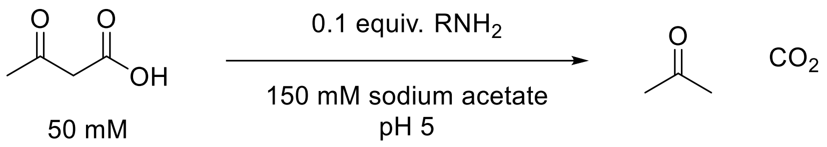

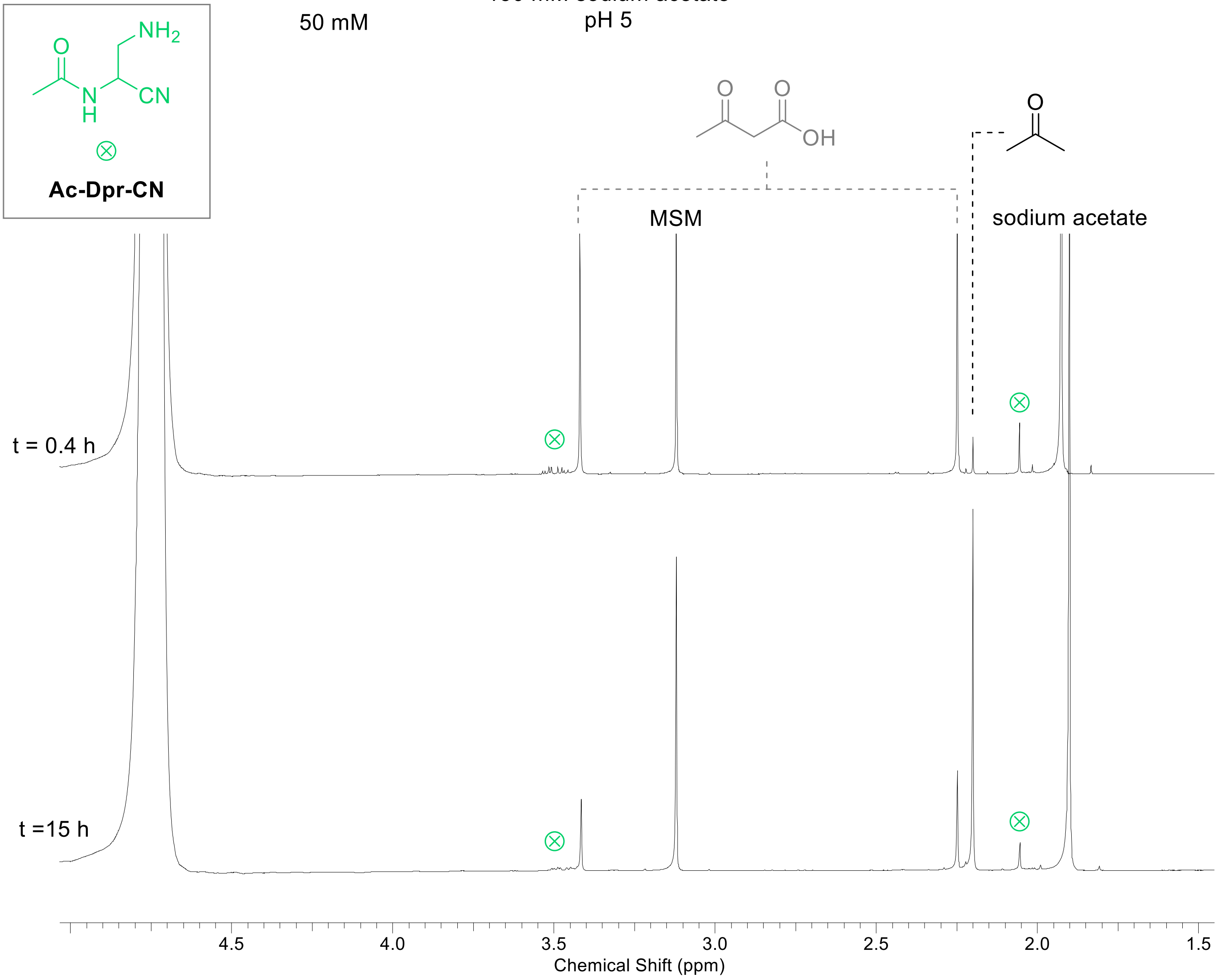

Supplementary Figure 83. *^{1}H NMR (700 MHz, H_2O, 1.5 – 5.0 ppm, noesygppr1d, field frequency lock = off) spectra to show the decarboxylation of acetoacetate (50 mM) catalysed by **Ac-Dpr-CN** (10 mol%) in sodium acetate buffer (150 mM, H_2O, pH 5) after 0.4 h (top spectrum) and 15 h (bottom spectrum).*

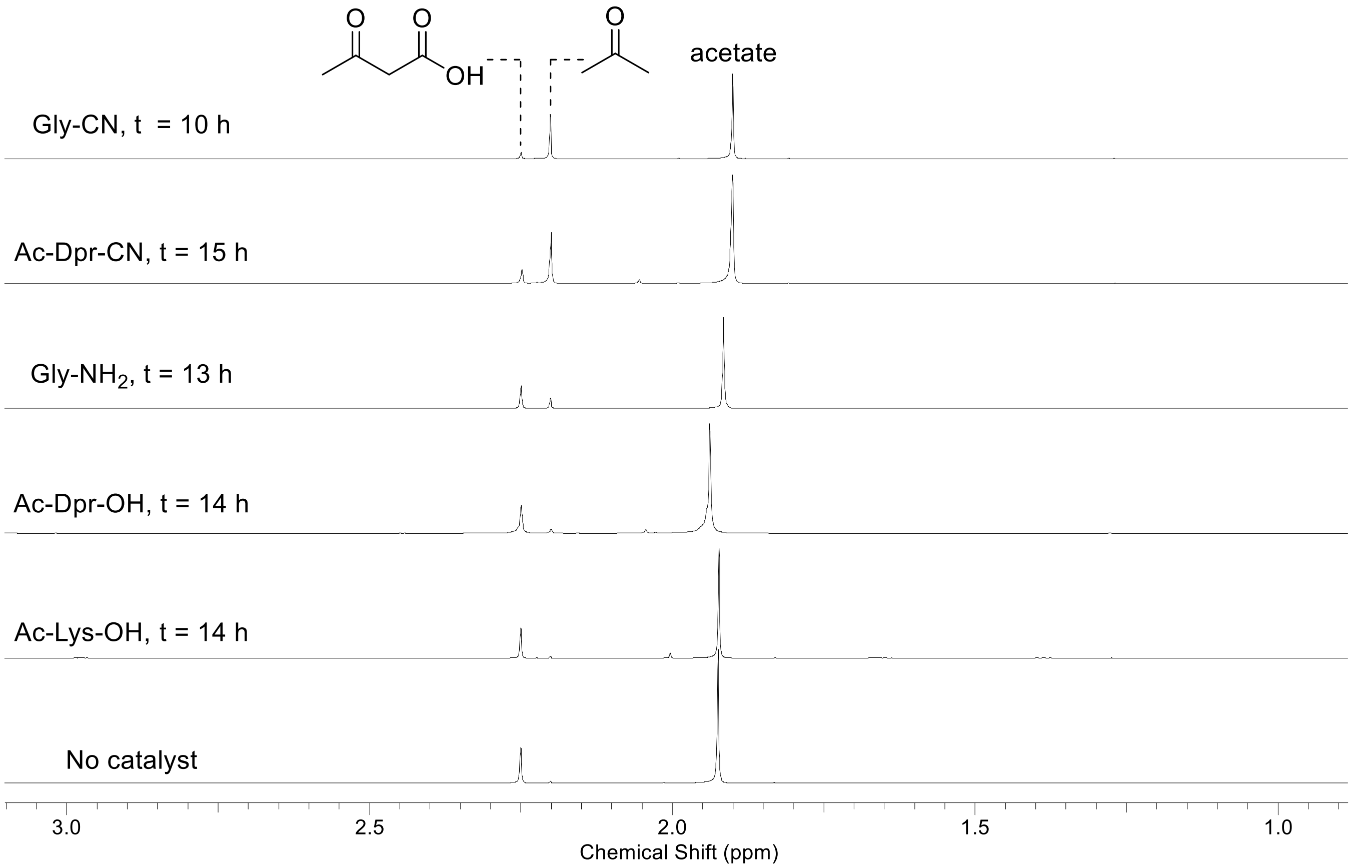

Supplementary Figure 84. ^{1}H NMR (700 MHz, H_2O, 1.0 – 3.0 ppm, noesygppr1d, field frequency lock = off) spectra to show the decarboxylation of acetoacetate (50 mM) catalysed by the specified amine catalyst (10 mol%) in sodium acetate buffer (150 mM, H_2O, pH 5).

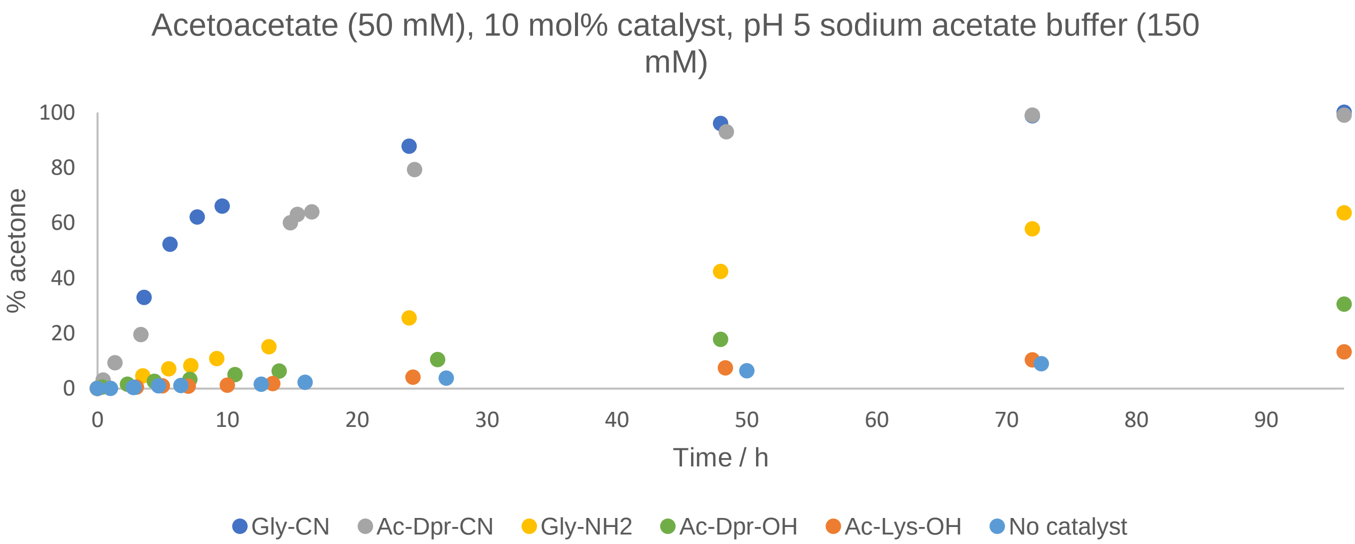

Supplementary Figure 85. ^{1}H NMR yields (%) of acetone plotted against time (h) in the decarboxylation of acetoacetate (50 mM) in sodium acetate buffer (150 mM, H_2O, pH 5) with the specified amine catalyst (10 mol%).

Decarboxylation of acetoacetate at pH 7

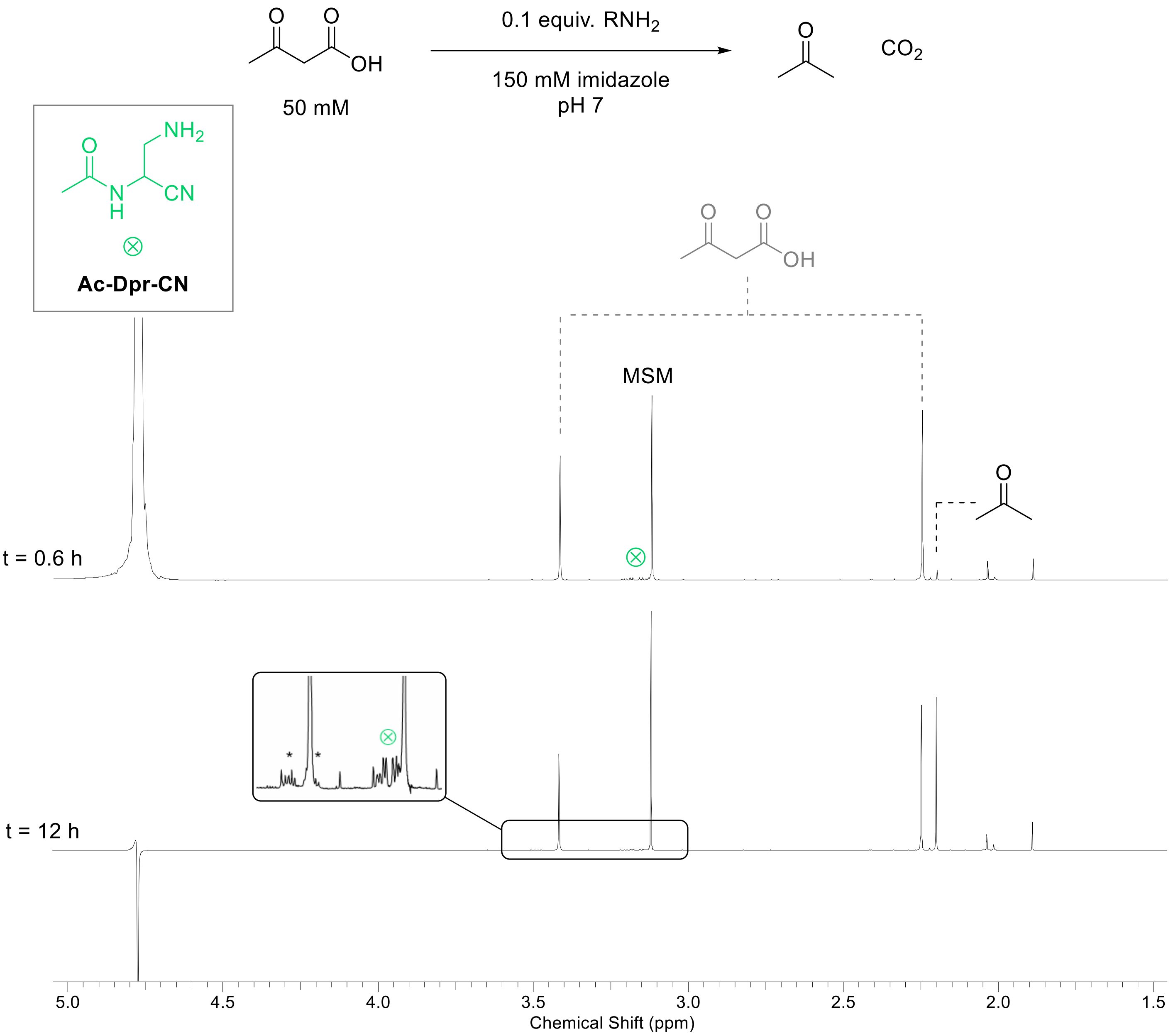

*Supplementary Figure 86. ^{1}H NMR (700 MHz, H_2O, 1.5 – 5.0 ppm, noesygppr1d, field frequency lock = off) spectra to show the decarboxylation of acetoacetate (50 mM) catalysed by **Ac-Dpr-CN** (10 mol%) in imidazole buffer (150 mM, H_2O, pH 7) after 0.6 h (top spectrum) and 12 h (bottom spectrum). Inset: ***Ac-Dpr(CO_2)-CN** forms by reversible β–amine carboxylation of **Ac-Dpr-CN**.*

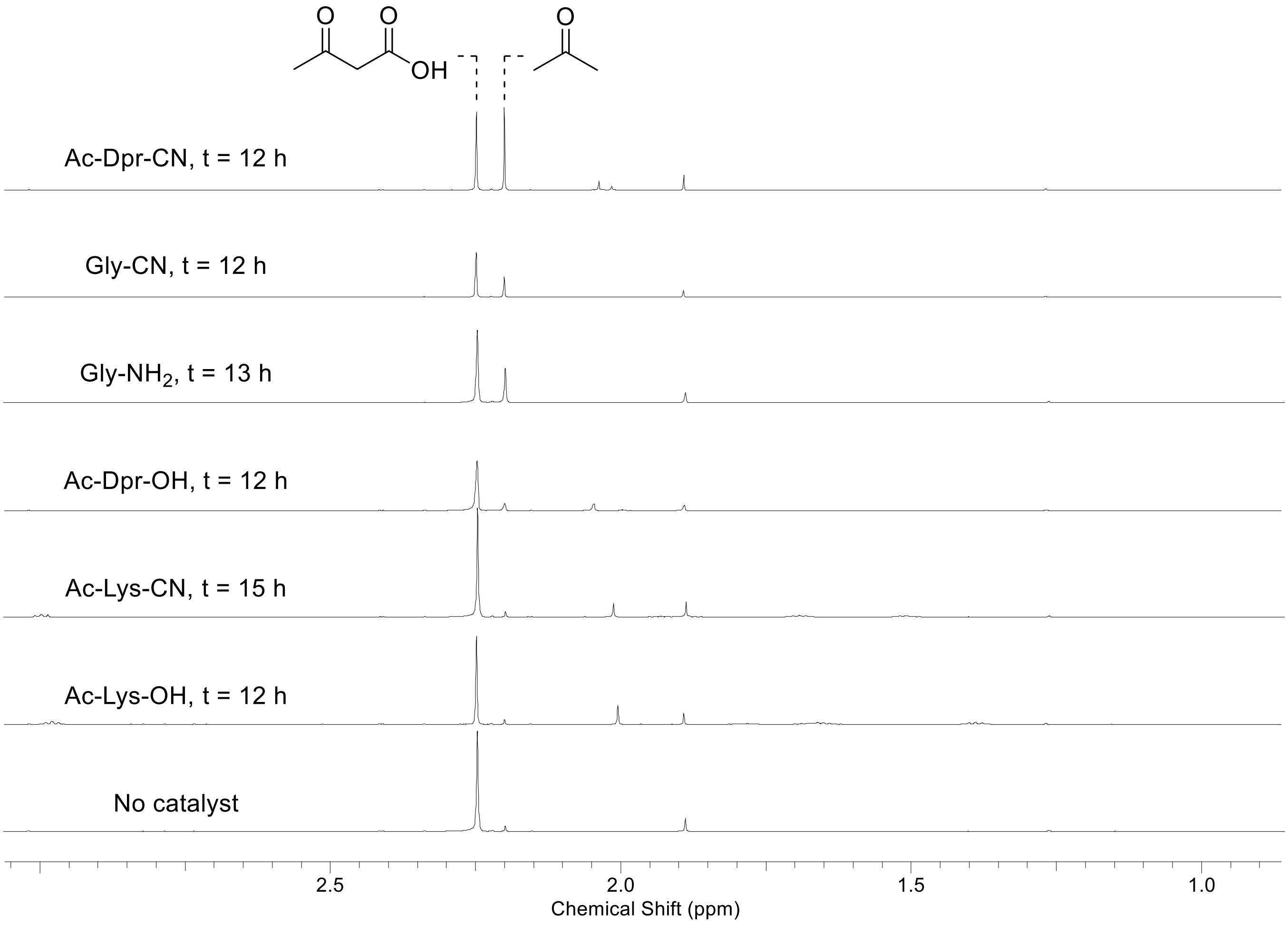

Supplementary Figure 87. [1]*H NMR (700 MHz, H_2O, 1.0 – 3.0 ppm, noesygppr1d, field frequency lock = off) spectra to show the decarboxylation of acetoacetate (50 mM) catalysed by the specified amine catalyst (10 mol%) in imidazole buffer (150 mM, H_2O, pH 7).*

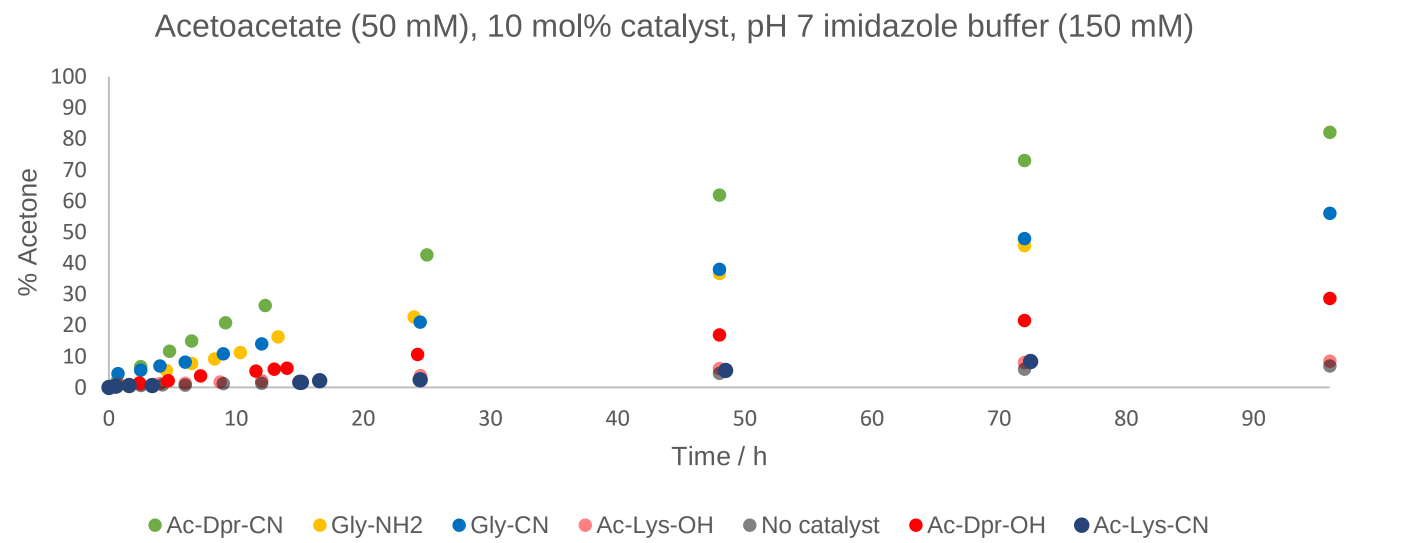

Supplementary Figure 88. [1]*H NMR yields (%) of acetone plotted against time (h) in the decarboxylation of acetoacetate (50 mM) in imidazole buffer (150 mM, H_2O, pH 7) with the specified amine catalyst (10 mol%).*

Ac-Dpr-CN		Ac-Dpr-OH		Ac-Lys-CN		Ac-Lys-OH		None	
Time / h	% Acetone	Time / h	% Acetone	Time / h	% Acetone	Time / h	% Acetone	Time / h	% Acetone
0	0	0	0	0	0	0	0	0	0
0.4	8	1	1	0.5	0	1	1	1	0
1	21	2	3	1	1	2	1	3	1
3	36	3	6	3	1	3	1	4	1
4	42	4	8	5	2	4	1	6	1
5	49	5	10	7	2	5	1	9	1
11	68	10	16	12	3	10	2	12	1
12	71	11	20	18	4	11	3	25	3
13	72	12	21	24	6	12	3	48	4
18	77	13	22	49	9	13	3		
24	82	18	30			19	4		
36	84	25	37			24	5		
48	85	37	47			48	7		
		45	52						

Supplementary Table 11. ^{1}H NMR yields of acetone over time in the decarboxylation of acetoacetate (50 mM) in imidazole buffer (150 mM, H_2O, pH 7) with the specified amine catalyst (50 mol%).

Decarboxylation of acetoacetate at pH 9

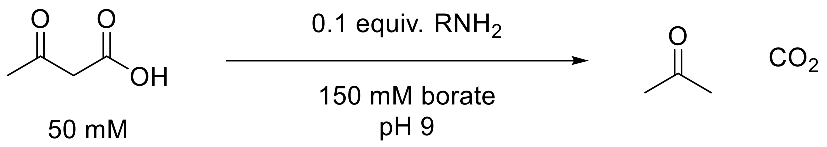

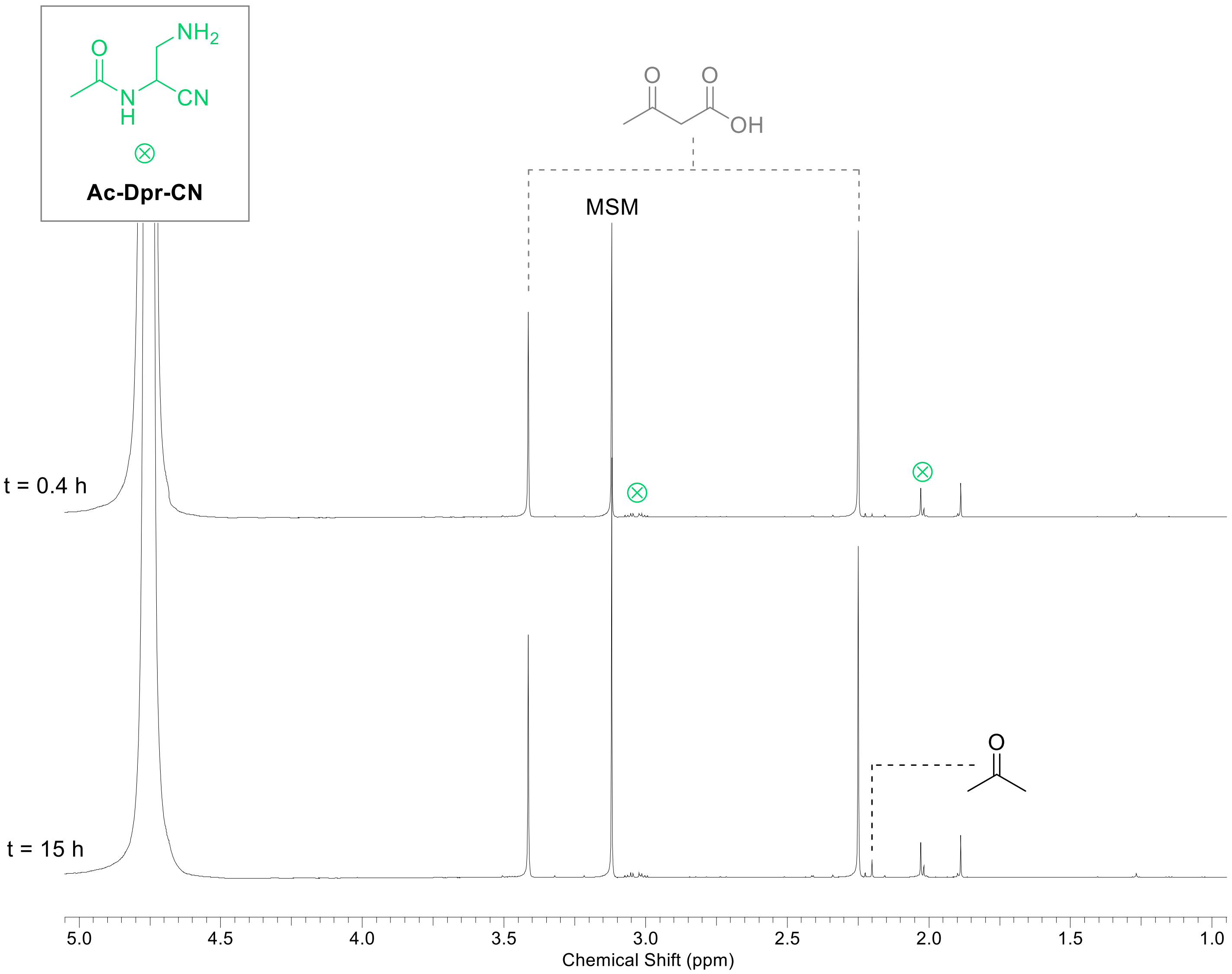

*Supplementary Figure 89. ^{1}H NMR (700 MHz, H_2O, 1.0 – 5.0 ppm, noesygppr1d, field frequency lock = off) spectra to show the decarboxylation of acetoacetate (50 mM) catalysed by **Ac-Dpr-CN** (10 mol%) in borate buffer (150 mM, H_2O, pH 9) after 0.4 h (top spectrum) and 15 h (bottom spectrum).*

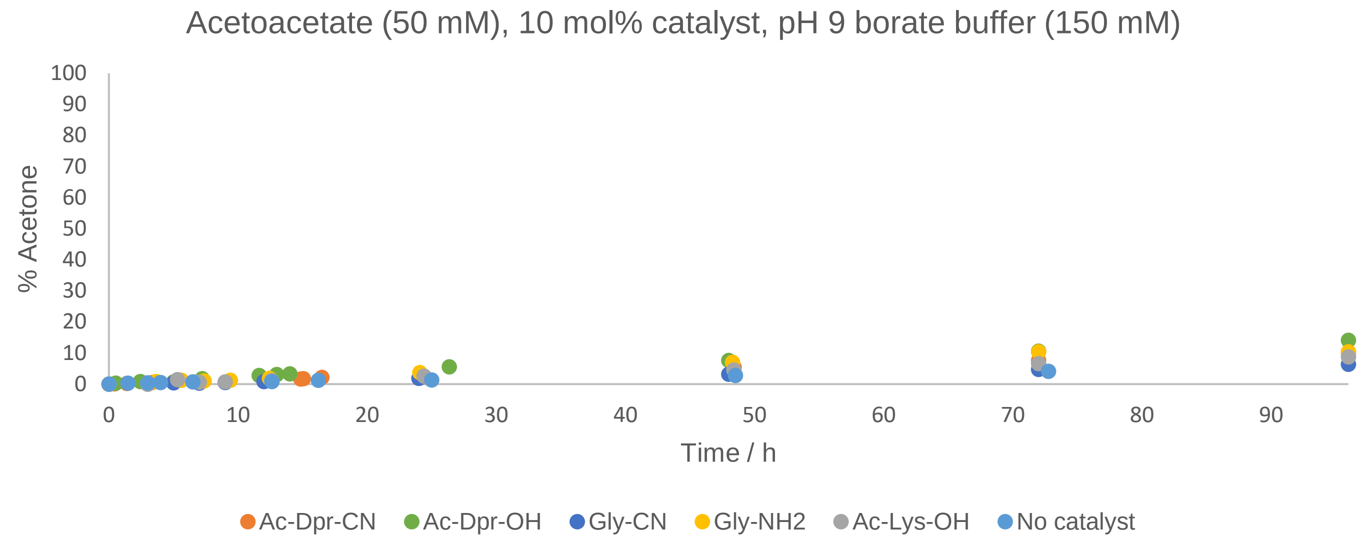

Supplementary Figure 90. ^{1}H NMR yields (%) of acetone plotted against time (h) in the decarboxylation of acetoacetate (50 mM) in borate buffer (150 mM, H_2O, pH 9) with the specified amine catalyst (10 mol%).

Synthesis of Ac-AA(Boc)-OH

Ac-Orn(Boc)-OH[6]

To **Ac-Orn-OH** (200 mg, 1.15 mmol) and $NaHCO_3$ (579 mg, 6.89 mmol) in MeOH (6.0 mL) was added Boc_2O (606 mg, 2.78 mmol). The reaction mixture was sonicated for 90 min then stirred at room temperature for 16 h. The reaction mixture was filtered and the solvent removed *in vacuo*. Purification by column chromatography (EtOAc/AcOH; 99:1 to EtOAc/MeOH; 69:30:1) afforded the title compound as a white powder (250 mg, 0.91 mmol, 79%). **^{1}H NMR** (700 MHz, CD_3OD) δ_H 4.31 (dd, *J* = 8.3, 4.8 Hz, 1H, (C2)–H), 3.05 (t, *J* = 6.7 Hz, 2H, (C5)–H_2), 1.99 (s, 3H, $COCH_3$), 1.83-1.88 (m, 1H, (C3)–H), 1.64-1.69 (m, 1H, (C3)–H'), 1.51-1.56 (m, 2H, (C4)–H_2), 1.43 (s, 9H, (CO)OC$(CH_3)_3$). **^{13}C NMR** (176 MHz, CD_3OD) δ_C 177.2 (C1), 173.2 (**C**OCH_3), 158.7 ((**C**O)OC$(CH_3)_3$), 80.0 ((CO)O**C**$(CH_3)_3$), 54.7 (C2), 41.1 (C5), 30.6 (C3), 28.9 (3C, (CO)OC(**C**$H_3)_3$), 27.6 (C4), 22.7 (CO**C**H_3). **HRMS-ESI** $[M+Na]^+$ calc. for $C_{12}H_{22}N_2O_5Na^+$ 297.1421; obs. 297.1421. **IR** (solid, cm^{-1}): 3328, 2964, 1698, 1680, 1623, 1560, 1519. **R_f** = 0.16 in 89:10:1 $CHCl_2$/MeOH/AcOH.

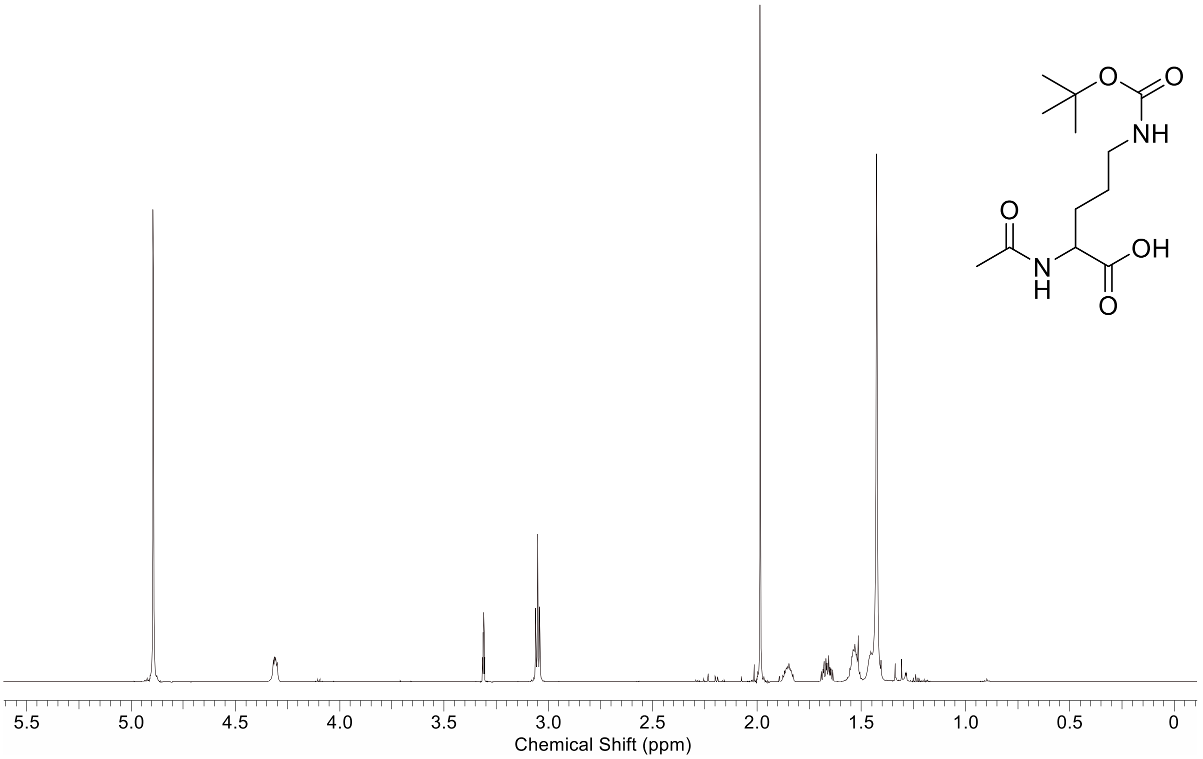

Supplementary Figure 91. ^{1}H NMR (700 MHz, CD_3OD, 0.0 – 5.5 ppm) spectrum of ***Ac-Orn(Boc)-OH****.*

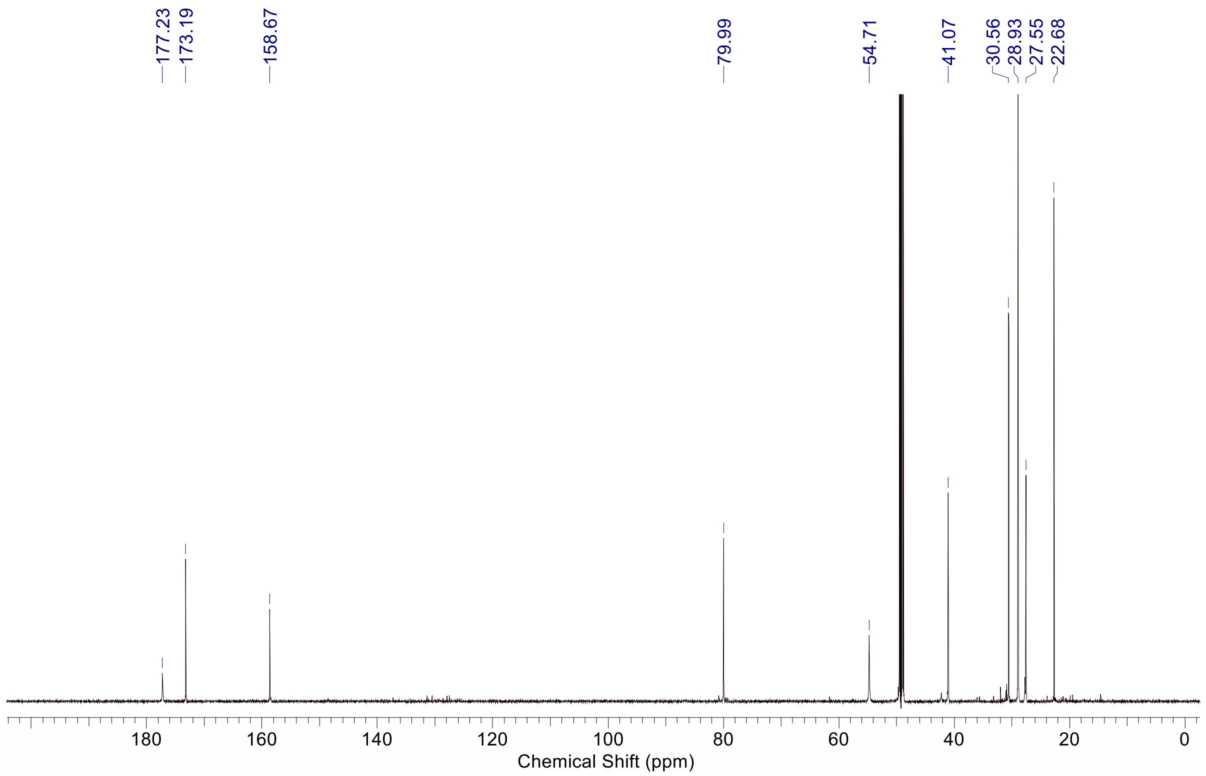

*Supplementary Figure 92. ^{13}C NMR (176 MHz, CD_3OD, 0 – 200 ppm) spectrum of **Ac-Orn(Boc)-OH**.*

Ac-Dab(Boc)-OH

To **H-Dab(Boc)-OH** (390 mg, 1.79 mmol) in H_2O (10.0 mL) at pH 10 was added Ac_2O (1.50 mL). The reaction mixture was sonicated for 30 min and left to stand at room temperature overnight. Purification by column chromatography (CH_2Cl_2/AcOH; 99.9:0.1 to CH_2Cl_2/MeOH/AcOH; 89:10:1) afforded the title compound as a white powder (156 mg, 0.60 mmol, 33%). **^{1}H NMR** (700 MHz, D_2O) δ_H 4.38 (dd, J = 8.6, 3.9 Hz, 1H, (C2)–H), 3.19 (t, J = 6.3 Hz, 2H, (C4)–H_2), 2.06-2.10 (m, 1H, (C3)–H), 2.05 (s, 3H, $COCH_3$), 1.86-1.91 (m, 1H, (C3)–H'), 1.43 (br. s, 9H, (CO)OC(CH_3)$_3$). **^{13}C NMR** (176 MHz, D_2O) δ_H 176.4 (C1), 174.7 (**C**OCH$_3$), 158.6 ((**C**O)OC(CH$_3$)$_3$), 81.5 ((CO)O**C**(CH$_3$)$_3$), 51.1 (C2), 37.0 (C4), 31.0 (C3), 28.2 (3C, (CO)OC(**C**H$_3$)$_3$), 22.2 (CO**C**H$_3$). **HRMS-ESI** [M+Na]$^+$ calc. for $C_{11}H_{20}N_2O_5Na^+$ 283.1264; obs. 283.1263. **IR** (solid, cm^{-1}): 3331, 1703, 1680, 1619, 1560, 1519. **R$_f$** = 0.17 in 69.9:30:0.1 EtOAc/MeOH/HCO_2H.

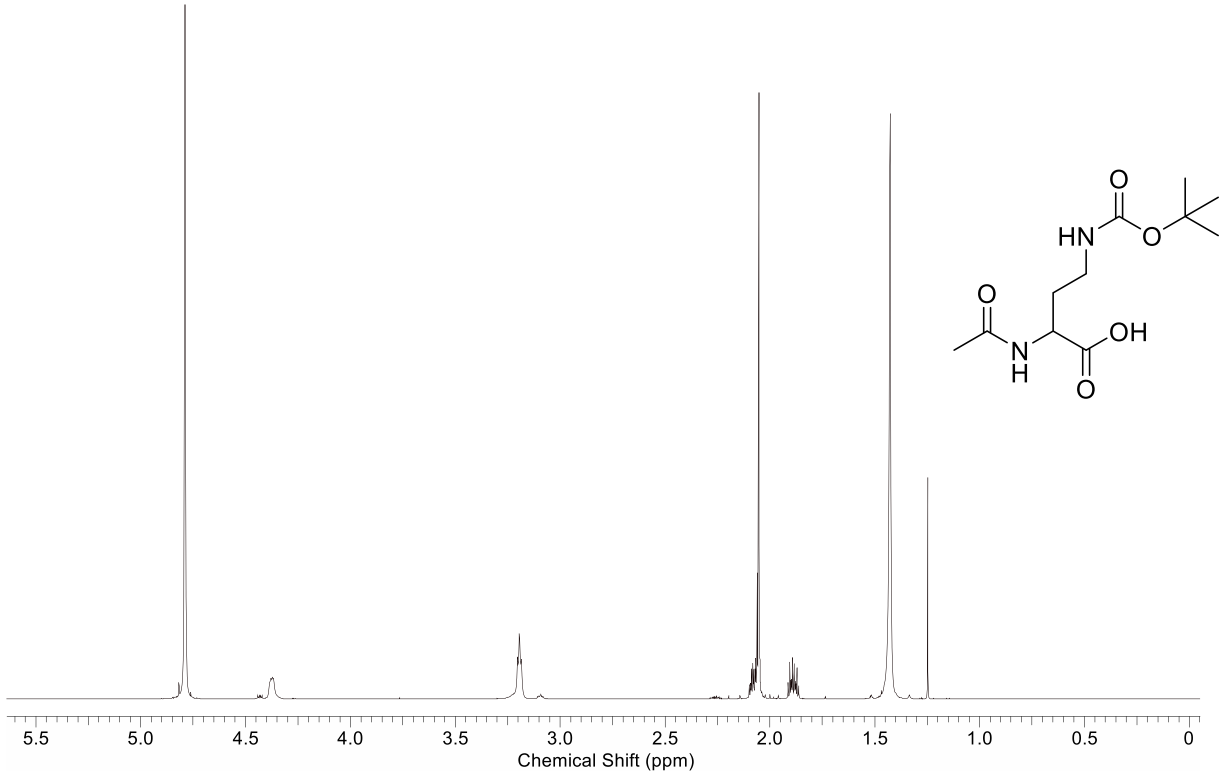

*Supplementary Figure 93. ^{1}H NMR (700 MHz, D_2O, 0.0 – 5.5 ppm) spectrum of **Ac-Dab(Boc)-OH**.*

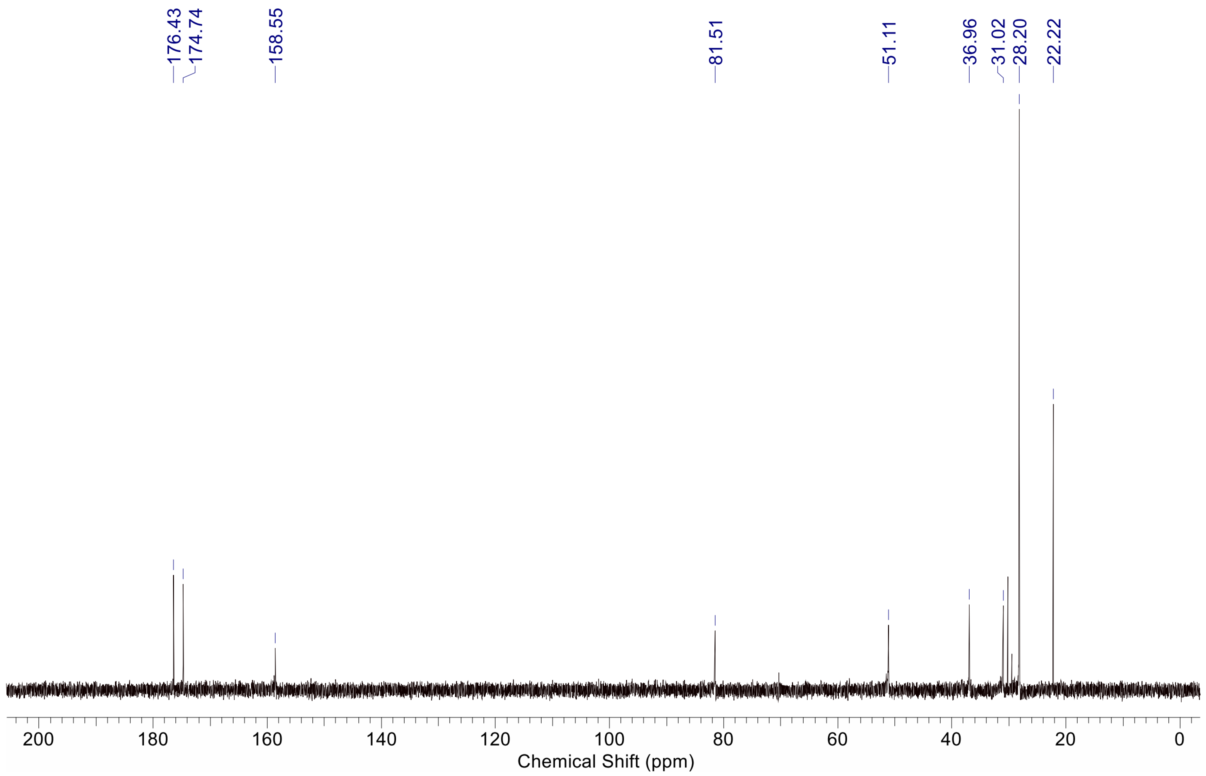

Supplementary Figure 94. ^{13}C NMR (176 MHz, D_2O, 0 – 200 ppm) spectrum of ***Ac-Dab(Boc)-OH.***

Fmoc-Dpr(Boc)-OH[7]

To **Fmoc-Dpr-OH** (815 mg, 2.50 mmol) in THF/H_2O (20.0 mL, 1:1) was added $NaHCO_3$ (419 mg, 4.99 mmol) at 0 °C. Boc_2O (713 mg, 3.27 mmol) was added portionwise and stirred at room temperature for 16 h. The reaction mixture was extracted with Et_2O (2 × 20.0 mL) and the aqueous layer adjusted to pH 1.0 with 5% $KHSO_4$. The aqueous layer was then extracted with EtOAc (3 × 20.0 mL), CH_2Cl_2 (20.0 mL) and the combined organic extracts dried over anhydrous Na_2SO_4 and the solvents removed *in vacuo* to afford the title compound as a white powder (836 mg, 1.96 mmol, 78%). **^{1}H NMR** (400 MHz, CD_3OD) δ_H 7.79 (d, *J* = 7.5 Hz, 2H, $(C_6H_4)_2$), 7.67 (dd, *J* = 7.5, 2.3 Hz, 2H, $(C_6H_4)_2$), 7.39 (t, *J* = 7.4Hz, 2H, $(C_6H_4)_2$), 7.31 (t, *J* = 7.4 Hz, 2H, $(C_6H_4)_2$), 4.28-4.37 (overlapping m, 3H, (C2)–H, OC**H**$_2$CH$(C_6H_4)_2$), 4.23 (t, *J* = 7.0 Hz, 1H, OCH$_2$C**H**$(C_6H_4)_2$), 3.53 (dd, *J* = 13.8, 4.5 Hz, 1H, (C3)–H), 3.38 (dd, *J* = 13.8, 7.5 Hz, 1H, (C3)–H'), 1.43 (s, 9H, (CO)O$(CH_3)_3$). **^{13}C NMR** (101 MHz, CD_3OD) δ_C 174.0 (C1), 158.7 (**C**OOCH$_2$CH$(C_6H_4)_2$/(**C**O)OC$(CH_3)_3$), 158.6 (**C**OOCH$_2$CH$(C_6H_4)_2$/(**C**O)OC$(CH_3)_3$) 145.4 (2C, $(C_6H_4)_2$), 142.6 (2C, $(C_6H_4)_2$), 128.9 (2C, $(C_6H_4)_2$), 128.3 (2C, $(C_6H_4)_2$), 126.4 (2C, $(C_6H_4)_2$), 121.0 (2C, $(C_6H_4)_2$), 80.6 ((CO)O**C**(CH_3)), 68.3 (OCH$_2$Fm), 56.1 (C2), 48.4 (OCH$_2$**C**H$(C_6H_4)_2$), 42.8 (C3), 28.9 (C3, (CO)OC(**C**H$_3$)). **HRMS-ESI** $[M+H]^+$ calc. for $C_{23}H_{27}N_2O_6^+$ 427.1864 ; obs. 427.1864. **IR** (solid, cm^{-1}): 3381, 2962, 1677, 1611, 1587, 1521. Characterisation data were in accordance with literature values.[7]

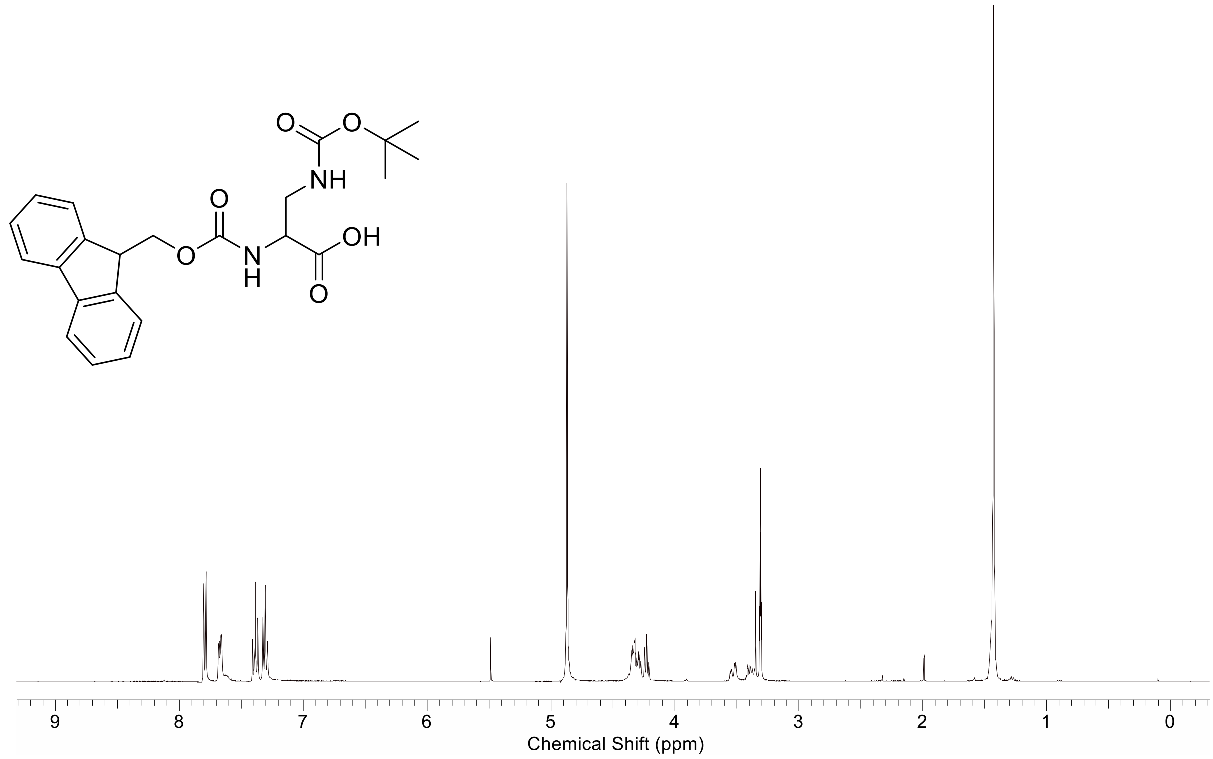

*Supplementary Figure 95. ^{1}H NMR (400 MHz, CD_3OD, 0.0 – 9.0 ppm) spectrum of **Fmoc-Dpr(Boc)-OH**.*

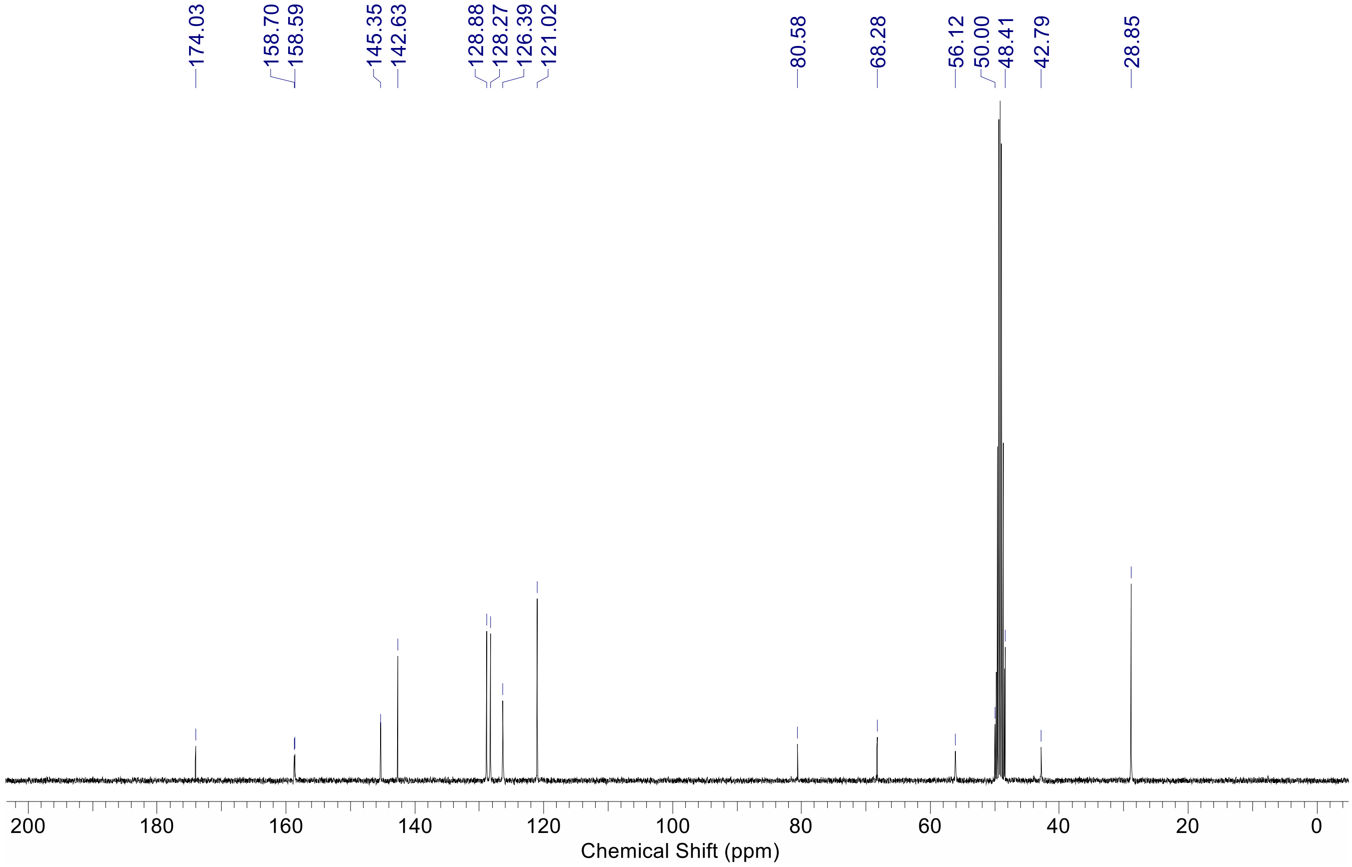

Supplementary Figure 96. ^{13}C NMR (101 MHz, CD_3OD, 0 – 200 ppm) spectrum of ***Fmoc-Dpr(Boc)-OH.***

Ac-Dpr(Boc)-OH[8]

Fmoc-Dpr(Boc)-OH (3.74 g, 8.77 mmol) was dissolved in 20% piperidine in DMF (19.0 mL) stirred at room temperature for 10 min. Volatiles were removed *in vacuo* and coevaporated with heptane (3 × 3.0 mL, t_{bath} = 55 °C). To the crude **H-Dpr(Boc)-OH** was added H_2O (60.0 mL) and the reaction mixture adjusted to pH 9.0 with 15 M KOH. Ac_2O (2.58 mL, 27.3 mmol) was added to the stirring solution whilst continually readjusted to pH 9.0 with 15 M KOH. The reaction mixture was then stirred at room temperaturet for 1 h. The reaction mixture was filtered and the filter paper washed with H_2O (3 × 10.0 mL). The solvent was reduced *in vacuo* to 20 mL and filtered again. The filtrate was then evaporated to dryness and coevaporated with toluene (3 × 10.0 mL, t_{bath} = 38 °C). The solids were dissolved in H_2O (100 mL), the solution was adjusted to pH 5.5 using 4 M HCl and washed with CH_2Cl_2 (4 × 25.0 mL). The aqueous layer was evaporated to dryness under reduced pressure, coevaporating with toluene (3 × 10.0 mL, t_{bath} = 38 °C). MeOH (100 mL) was added and the mixture filtered and concentrated to dryness. The crude residue was purified by column chromatography (CH_2Cl_2/MeOH/AcOH; 89:10:1) to afford the title compound as an off-white powder (1.07 g, 4.34 mmol, 50% over 2 steps). **^{1}H NMR** (400 MHz, CD_3OD) δ_H 4.48 (dd, *J* = 4.8, 7.3 Hz, 1H, (C2)–H), 3.51 (dd, *J* = 4.8, 13.9 Hz, 1H, (C3)–H), 3.38 (dd, *J* = 7.3, 13.9 Hz, 1H, (C3)–H'), 1.99 (s, 3H, $COCH_3$), 1.45 (s, 9H, (CO)OC(CH_3)$_3$). **^{13}C NMR** (101 MHz, CD_3OD) δ_C 173.6 (C1/**C**OCH_3), 173.5 (C1/**C**OCH_3), 158.7 ((**C**O)OC(CH_3)$_3$), 80.5 ((CO)O**C**(CH_3)$_3$), 54.6 (C2), 42.7 (C3), 28.8 (3C, (CO)OC(**C**H_3)$_3$), 22.6 (CO**C**H_3). **HRMS-ESI** $[M+H]^+$ calc. for $C_{10}H_{19}N_2O_5^+$ 247.1288 ; obs. 247.1288. **IR** (solid, cm^{-1}): 3293, 2977, 1719, 1687, 1638, 1524.

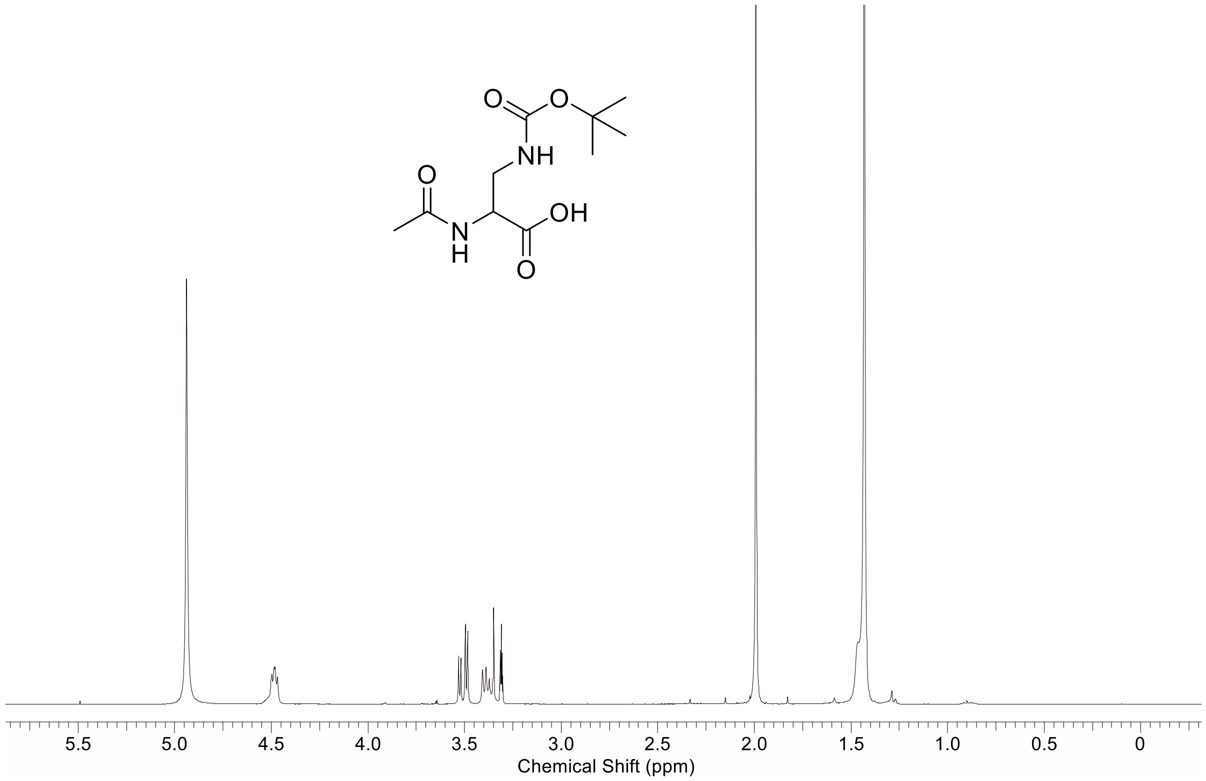

*Supplementary Figure 97. ^{1}H NMR (400 MHz, CD_3OD, 0.0 – 9.0 ppm) spectrum of **Ac-Dpr(Boc)-OH**.*

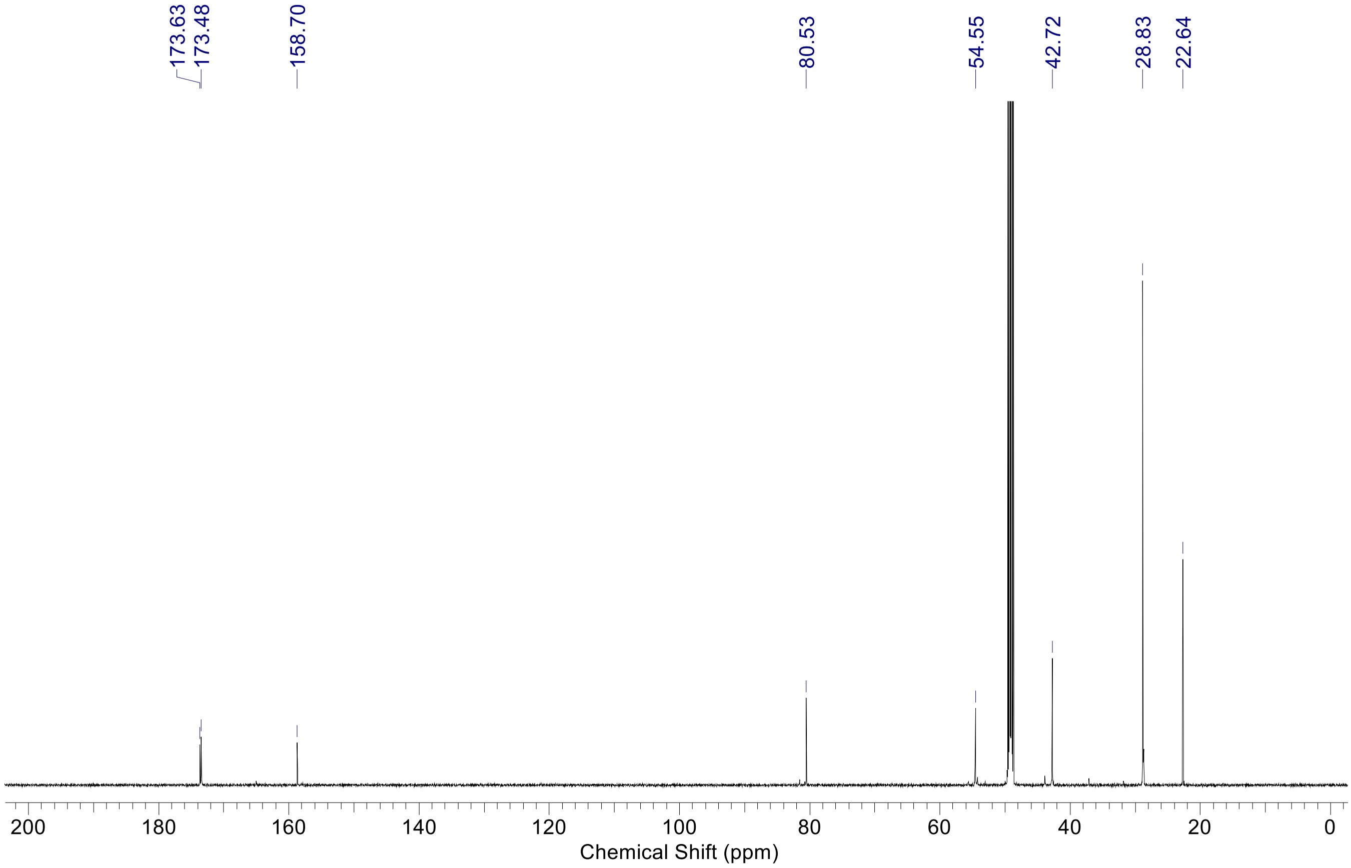

*Supplementary Figure 98. ^{13}C NMR (101 MHz, CD_3OD, 0 – 200 ppm) spectrum of **Ac-Dpr(Boc)-OH.***

Synthesis of Ac-AA(Boc)-S⁻Na⁺ from Ac-AA(Boc)-OH

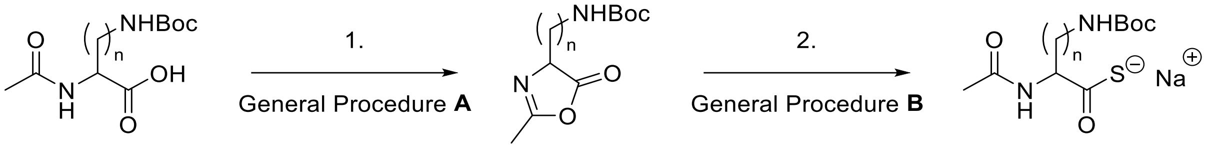

Supplementary Figure 99. Synthesis of ***Ac-AA(Boc)-S⁻Na⁺****. 1. EDC·HCl, CH_2Cl_2, 0 °C → rt, 1 h; 2. NaSH, MeCN, rt, 16 h.*

General procedure E: EDC-mediated 5(4*H*)-oxazolone formation.

To a suspension of **Ac-AA(Boc)-OH** (1.00 equiv.) in CH_2Cl_2 (9.5 mL/mmol) at 0 °C was added EDC·HCl (1.36 equiv.) under an argon atmosphere. The reaction mixture was warmed to room temperature and stirred for 1 h. The homogenous solution was then diluted with CH_2Cl_2 (9.5 mL/mmol), washed with water (3 × 9.5 mL/mmol), sat. aq. $NaHCO_3$ (3 × 9.5 mL/mmol) and brine (9.5 mL/mmol). The organic layer was dried over anhydrous Na_2SO_4, filtered and concentrated *in vacuo* to afford the corresponding 5(4*H*)-oxazolone as a colourless oil.

General procedure F: Thiolysis of 5-(4*H*)-oxazolones.

To the corresponding 5-(4*H*)-oxazolone (1.00 equiv.) in anhydrous MeCN (4.0 mL/mmol) was added freshly ground NaSH (1.05 equiv.) under an argon atmosphere. The reaction mixture was stirred at room temperature for 16 h, centrifuged and the supernatant dried *in vacuo* to afford the desired compound.

5-(4*H*)-oxazolone 16[2]

Prepared according to general procedure **E** using **Ac-Lys(Boc)-OH** (608 mg, 2.11 mmol) to afford **16** (570 mg, 2.11 mmol, 99%) as a colourless oil. **^{1}H NMR** (400 MHz, $CDCl_3$, mixture of two rotamers a/b, 85:15) δ_H 4.55 (br. s, 0.85H, NH, rotamer a), 4.30 (br. s, 0.15H, NH, rotamer b) 4.12 (ddd, J = 7.9, 5.5, 2.0 Hz, 1H, (C4)–H), 3.11 (br. dd, J = 7.9, 6.3 Hz, 2H, (C4')–H_2), 2.20 (d, J = 2.0 Hz, 3H, (C2)–CH_3), 1.95-1.87 (m, 1H, (C1')–H), 1.77-1.67 (m, 1H, (C1')–H'), 1.55-1.43 (m, 4H, (C2')–H_2, (C3')–H_2), 1.42 (s, 9H, $C(CH_3)_3$). **^{13}C NMR** (100 MHz, $CDCl_3$) δ_C 178.5 (C5), 162.9 (C2), 155.9 ((**C**O)OC($CH_3)_3$), 79.1 ((CO)O**C**($CH_3)_3$), 64.7 (C4), 40.2 (C4'), 30.8 (C1'), 29.6 (C3'), 28.4 (3C, (CO)OC(**C**$H_3)_3$), 22.6 (C2'), 15.2 ((C2)–**C**H_3). **HRMS-ESI** $[M+H]^+$ calc. for $C_{13}H_{23}N_2O_4^+$ 271.1652 ; obs. 271.1652.

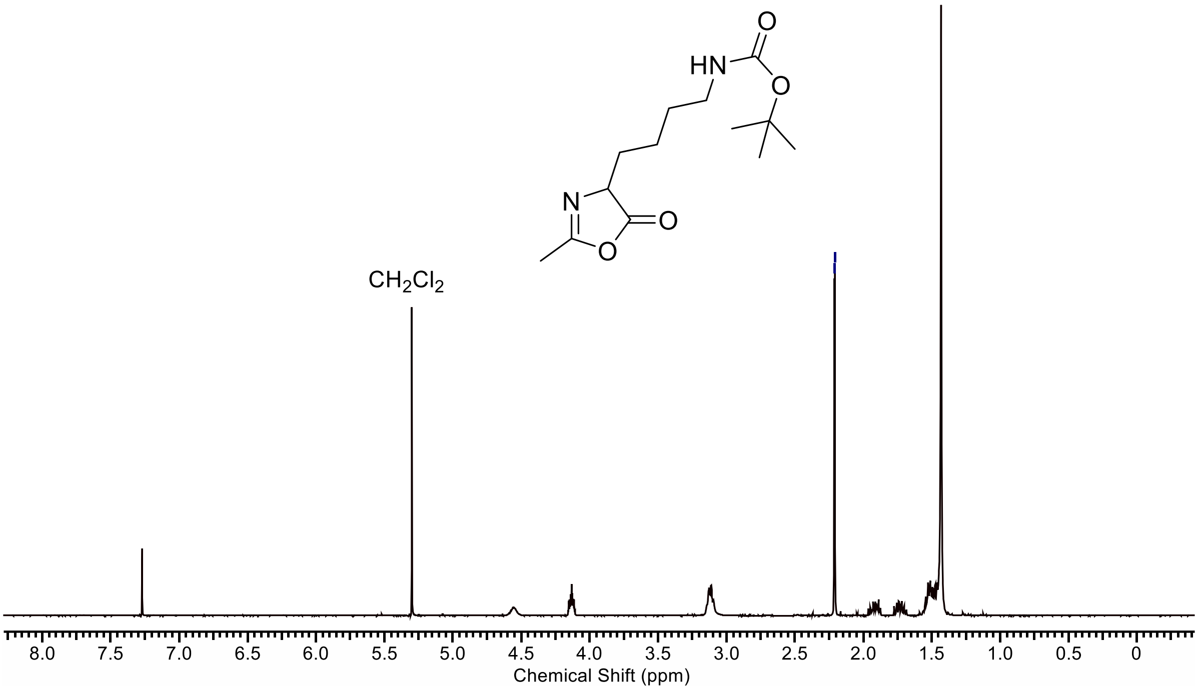

*Supplementary Figure 100. ^{1}H NMR (400 MHz, $CDCl_3$, 0.0 – 8.0 ppm) spectrum of **16.***

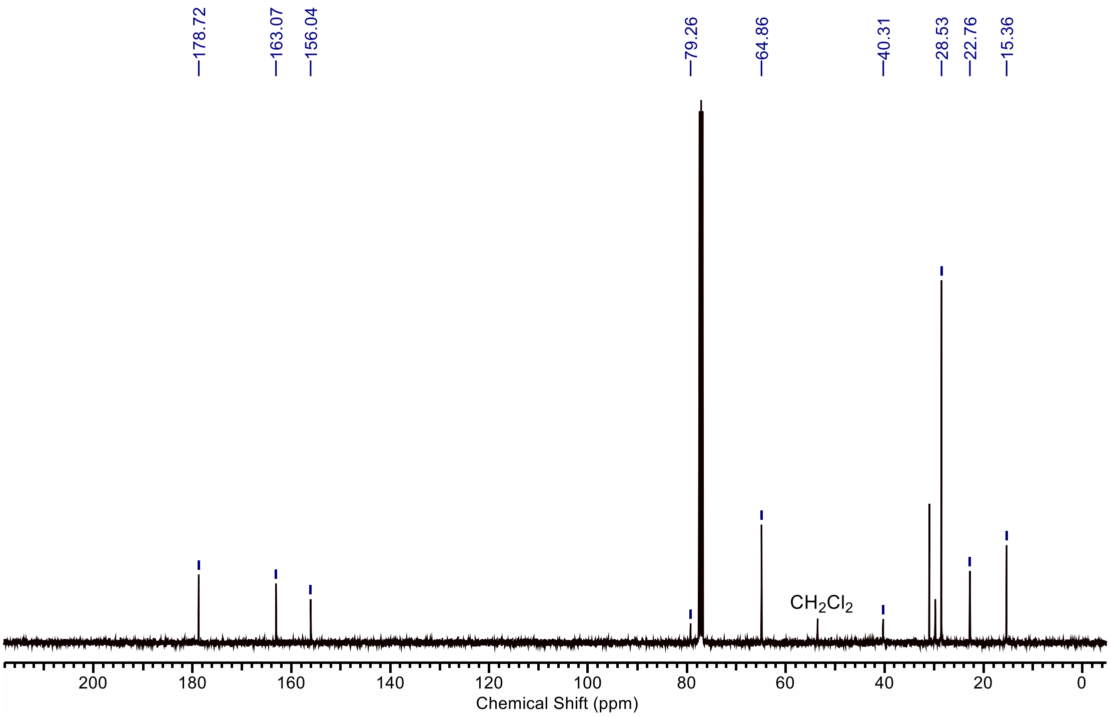

*Supplementary Figure 101. ^{13}C NMR (100 MHz, $CDCl_3$, 0 – 200 ppm) spectrum of **16**.*

Ac-Lys(Boc)-S⁻Na⁺ [2]

Prepared according to general procedure **F** using **16** (584 mg, 1.98 mmol) to afford **Ac-Lys(Boc)-S⁻Na⁺** (646 mg, 1.98 mmol, 90% pure by ^{1}H NMR spectroscopy) as a hydroscopic white powder. **^{1}H NMR** (400 MHz, D_2O) δ_H 4.39 (dd, J = 9.3, 4.4 Hz, 1H, (C2)–H), 3.05 (br. t, J = 5.9 Hz, 2H, (C6)–H_2), 2.03 (s, 3H, $COCH_3$), 1.96-1.87 (m, 1H, (C3)–H), 1.66 (m, 1H, (C3')–H), 1.53-1.45 (m, 2H, (C5)–H_2), 1.43 (s, 9H, (CO)OC(CH_3)$_3$), 1.40-1.30 (m, 2H, (C4)–H_2). **^{13}C NMR** (100 MHz, D_2O) δ_C 221.6 (C1), 173.9 (**C**OCH_3), 158.9 ((**C**O)OC(CH_3)$_3$), 81.3 ((CO)O**C**(CH_3)$_3$), 64.7 (C2), 40.4 (C6), 33.3 (C3), 29.2 (3C, (CO)OC(**C**H_3)$_3$), 28.3 (C5), 23.1 (C4), 22.6 (CO**C**H_3). **HRMS-ESI** [M+H]$^+$ calc. for $C_{13}H_{24}N_2O_4SNa^+$ 327.1349 ; obs. 327.1349. **IR** (solid, cm^{-1}): 3289, 2931, 1687, 1656, 1510.

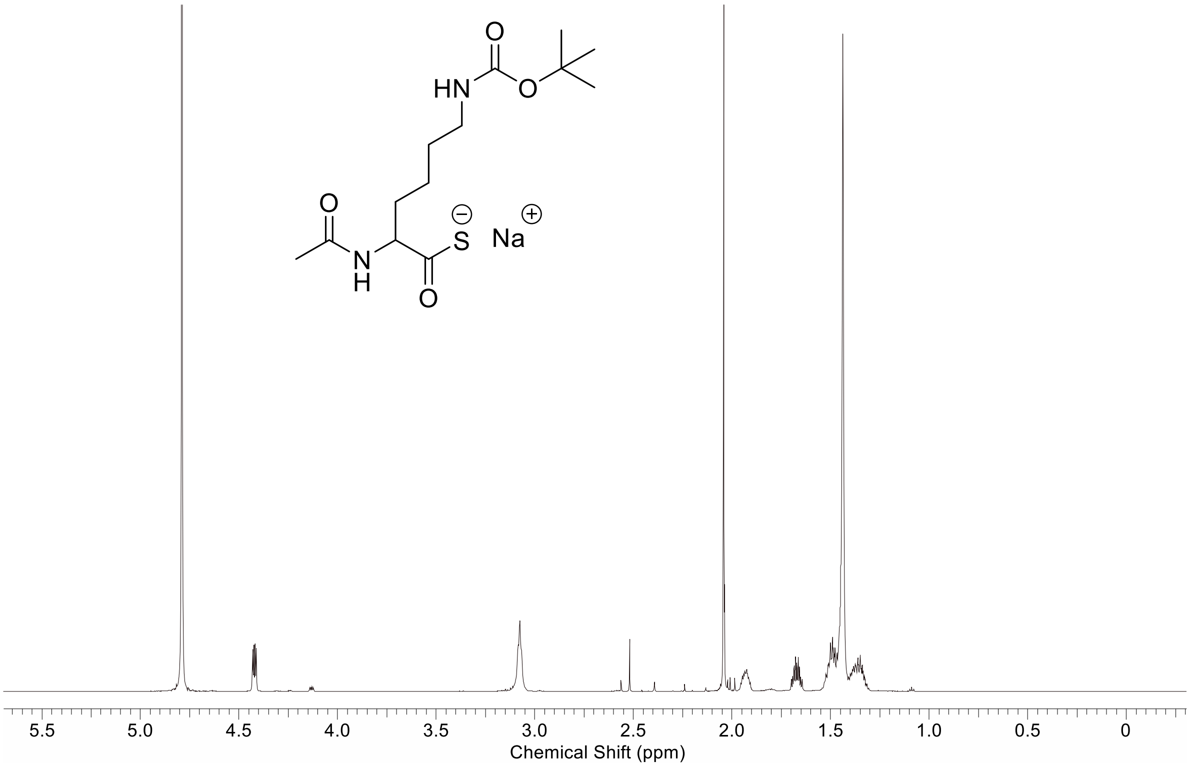

*Supplementary Figure 102. ^{1}H NMR (400 MHz, D_2O, 0.0 – 5.5 ppm) spectrum of **Ac-Lys(Boc)-S⁻Na⁺**.*

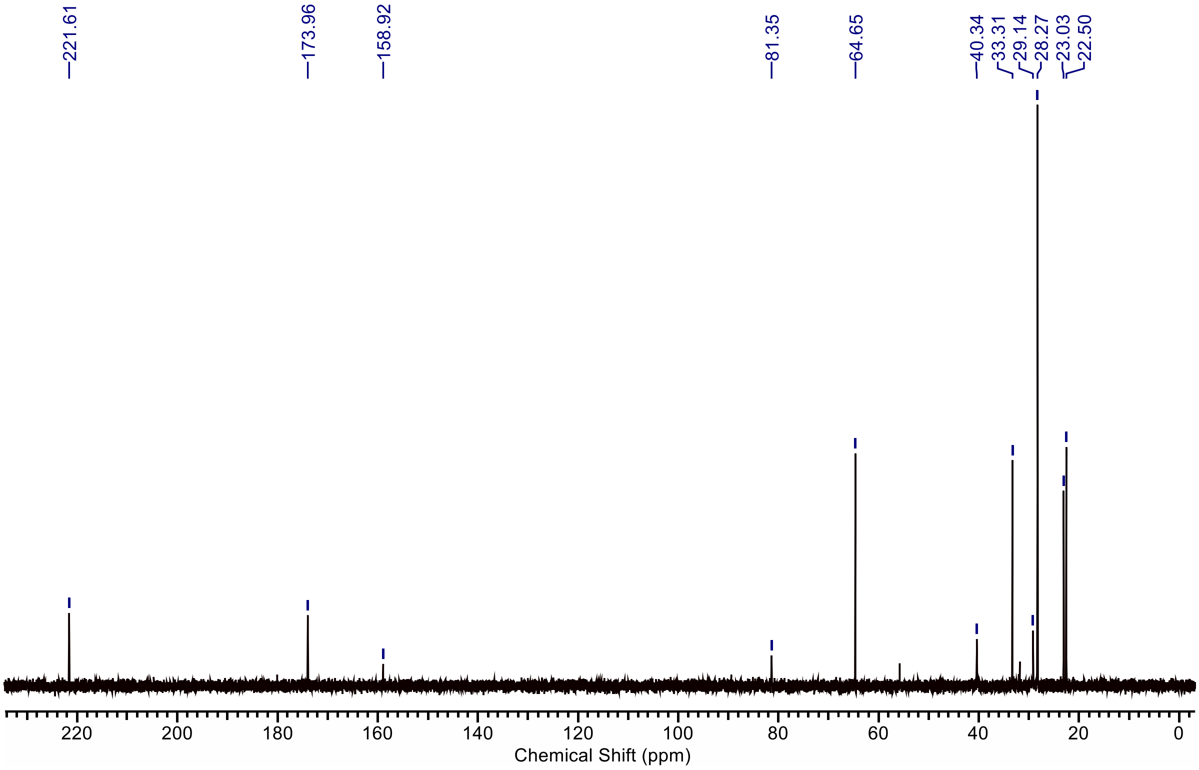

Supplementary Figure 103. ^{13}C NMR (100 MHz, D_2O, 0 – 225 ppm) spectrum of ***Ac-Lys(Boc)-S$^-$Na$^+$****.*

5-(4*H*)-oxazolone 17

Prepared according to general procedure **E** using **Ac-Orn(Boc)-OH** (560 mg, 2.04 mmol) to afford **17** (480 mg, 1.87 mmol, 92%) as an unstable yellow oil. **^{1}H NMR** (400 MHz, $CDCl_3$) δ_H 4.70 (br. s, 1H, NH), 4.13 (br. m, 1H, (C4)–H), 3.13 (br. m, 2H, (C3')–H_2), 2.17 (d, *J* = 2.0 Hz, 3H, CH_3), 1.95 – 1.86 (m, 1H, (C1')–H), 1.72 – 1.53 (m, 3H, (C1')–H', (C2')–H_2), 1.39 (s, 9H, (CO)OC(CH_3)$_3$). **^{13}C NMR** (100 MHz, $CDCl_3$) δ_C 178.5 (C5), 163.2 (C2), 156.1 ((**C**O)OC(CH_3)$_3$), 79.3 ((CO)O**C**(CH_3)$_3$), 64.5 (C4), 39.8 (C3'), 28.5 (C1'), 28.5 (3C, (CO)OC(**C**H_3)$_3$), 26.1 (C2'), 15.3 ((C2)–**C**H_3).

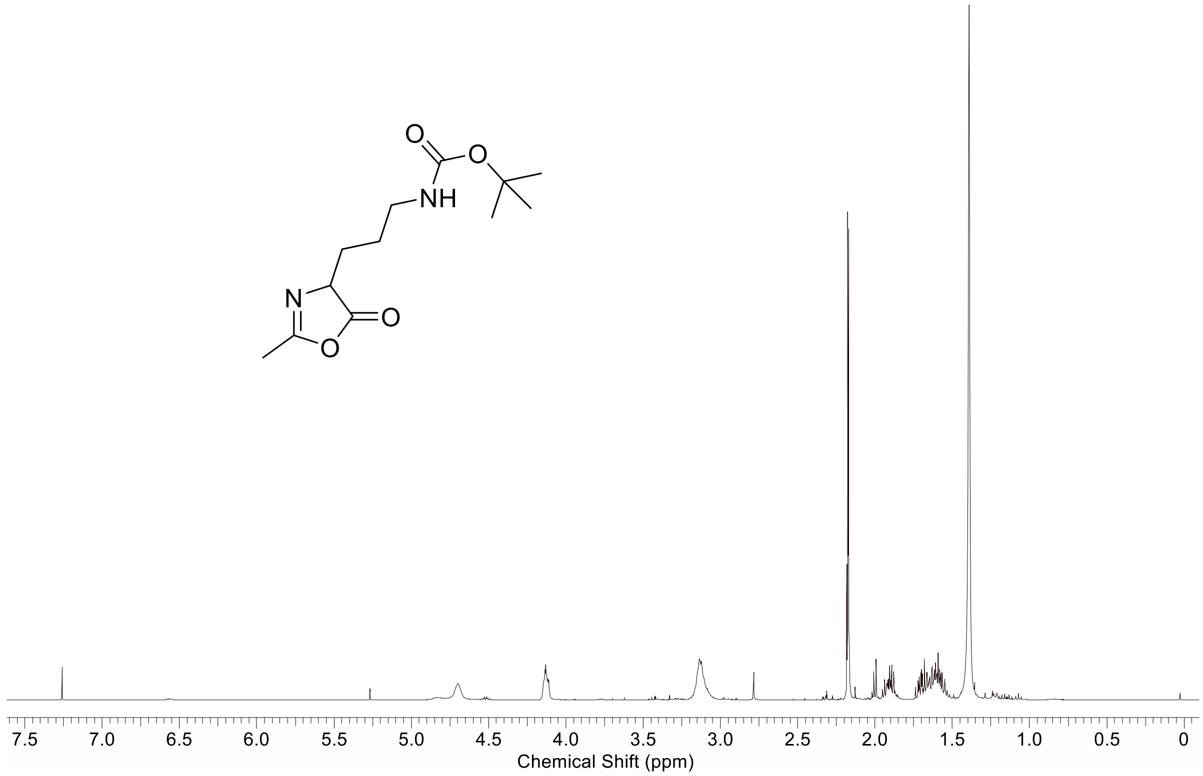

*Supplementary Figure 104. ^{1}H NMR (400 MHz, $CDCl_3$, 0.0 – 7.5 ppm) spectrum of **17**.*

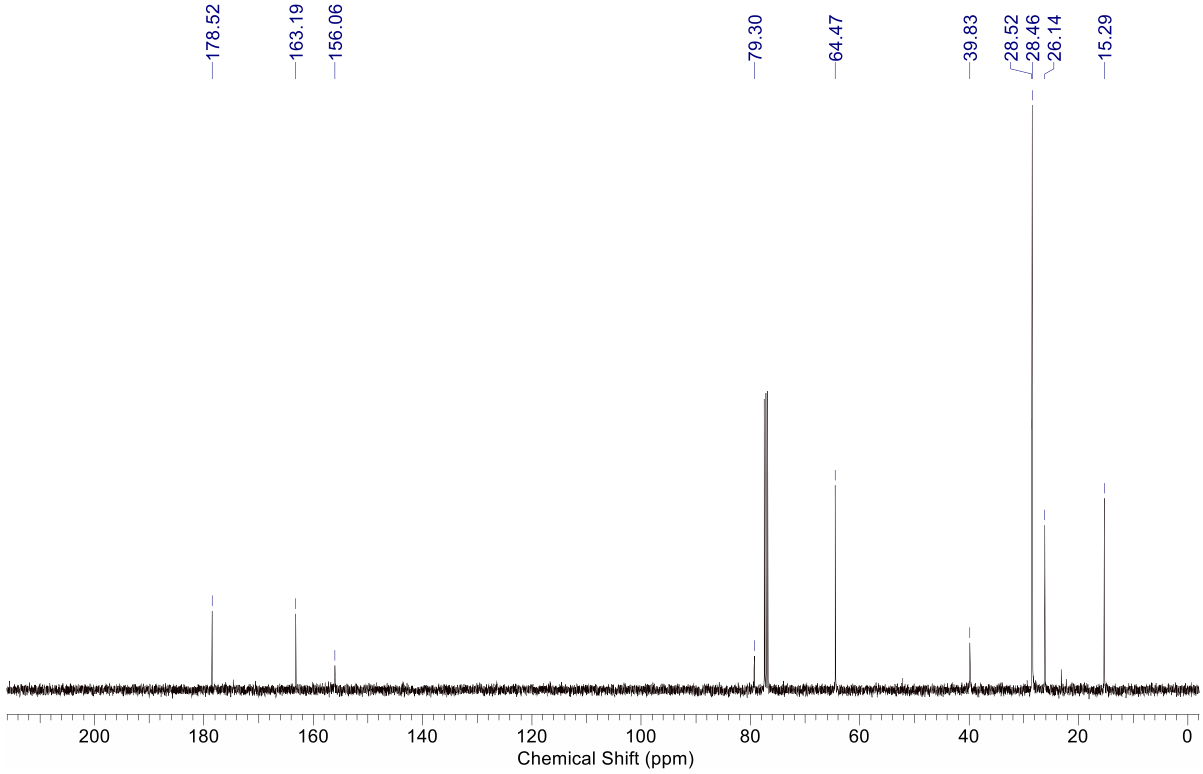

*Supplementary Figure 105.^{13}C NMR (100 MHz, $CDCl_3$, 0 – 200 ppm) spectrum of **17**.*

Ac-Orn(Boc)-S$^-$Na$^+$

Prepared according to general procedure **F** using **17** (367 mg, 1.43 mmol) to afford **Ac-Orn(Boc)-S$^-$Na$^+$** (463 mg, 1.48 mmol, 97% pure by ^{1}H NMR) as a white powder. **^{1}H NMR** (400 MHz, D_2O) δ_H 4.42 (dd, J = 8.8, 4.8 Hz, 1H, (C2)–H), 3.08 (t, J = 6.8 Hz, 2H, (C5)–H_2), 2.04 (s, 3H, $COCH_3$), 1.99-1.88 (m, 1H, (C3)–H), 1.72-1.63 (m, 1H, (C3)–H'), 1.59-1.47 (m, 2H, (C4)–H_2), 1.44 (s, 9H, (CO)OC$(CH_3)_3$). **^{13}C NMR** (176 MHz, D_2O) δ_C 221.2 (C1), 174.0 (**C**OCH_3), 158.9 ((**C**O)OC$(CH_3)_3$), 81.4 ((CO)O**C**$(CH_3)_3$), 64.4 (C2), 40.2 (C5), 31.0 (C3), 28.3 (3C, (CO)OC(**C**$H_3)_3$), 26.0 (C4), 22.5 (CO**C**H_3). **HRMS-ESI** [M+Na]$^+$ calc. for $C_{12}H_{22}N_2O_4SNa^+$ 313.1190 ; obs. 313.1190. **IR** (solid, cm^{-1}): 3345, 2978, 1699, 1626, 1510.

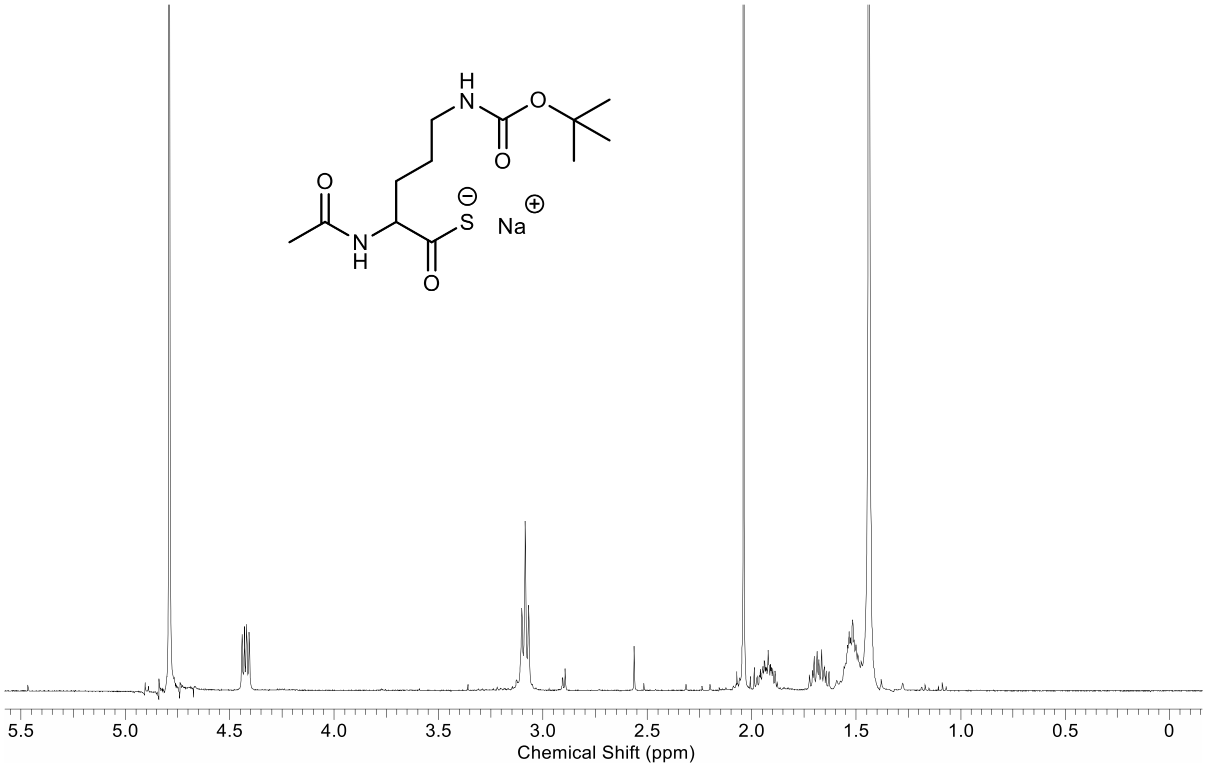

*Supplementary Figure 106. ^{1}H NMR (400 MHz, D_2O, 0.0 – 5.5 ppm) spectrum of **Ac-Orn(Boc)-S$^-$Na$^+$**.*

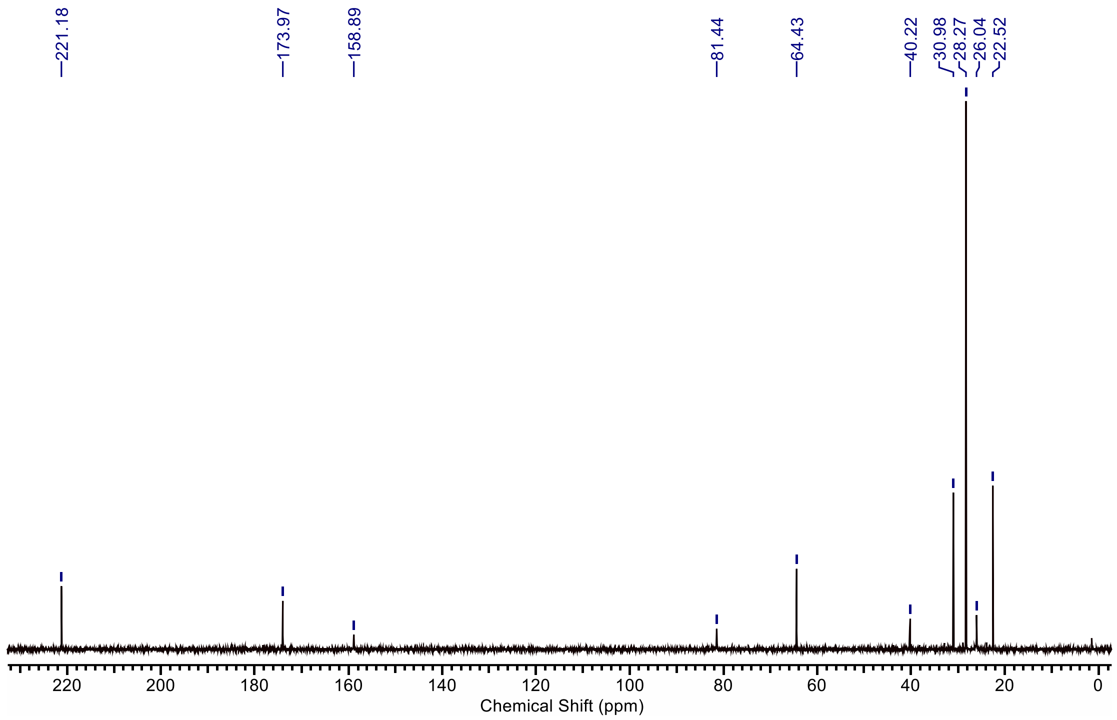

Supplementary Figure 107. ^{13}*C NMR (176 MHz,* D_2O*, 0 – 225 ppm) spectrum of* ***Ac-Orn(Boc)-S⁻Na⁺****.*

Ac-Dab(Boc)-S$^-$Na$^+$

Prepared according to general procedure **E** and **F** using **Ac-Dab(Boc)-OH** (66.0 mg, 0.27 mmol) to afford **Ac-Dab(Boc)-S$^-$Na$^+$** (65% by ^{1}H NMR)/lactam **18** (8% by ^{1}H NMR) over two steps as a mixture that was used without further purification.

Ac-Dab(Boc)-S$^-$Na$^+$ (■) **^{1}H NMR** (700 MHz, D_2O) δ_H 4.46 (dd, J = 3.8, 9.9 Hz, 1H, (C2)–H), 3.16 (t, J = 6.7 Hz, 2H, (C4)–H_2), 2.14 (m, 1H, (C3)–H), 2.05 (s, 3H, $COCH_3$), 1.78 (m, 1H, (C3)–H'), 1.44 (br s, 9H, (CO)OC$(CH_3)_3$). **^{13}C NMR** (176 MHz, D_2O) δ_C 221.0 (C1), 174.0 (**C**OCH_3), 158.6 ((**C**O)OC$(CH_3)_3$), 81.5 ((CO)O**C**$(CH_3)_3$), 62.3 (C2), 37.4 (C4), 33.5 (C3), 28.2 (3C, (CO)OC(**C**H_3)$_3$), 22.5 (CO**C**H_3). **HRMS-ESI** [M+Na]$^+$ calc. for $C_{11}H_{21}N_2O_4S^+$ 277.1217; obs. 277.1215.

18 (♦) **^{1}H NMR**: 4.62 (dd, J = 12.0, 9.0 Hz, 1H, (C2)–H), 3.90 (app tt, J = 9.5, 1.4 Hz, 1H, (C4)–H), 3.67 (m, 1H, (C4)–H'), 2.42 (m, 1H, (C3)–H)), 2.06 (s, 3H, $COCH_3$), 2.00 (m, 1H, (C3–H')), 1.53 (s, 9H, (CO)OC$(CH_3)_3$). **^{13}C NMR** (176 MHz, D_2O) δ_C 175.8 (C1/**C**OCH_3), 174.9 (C1/**C**OCH_3), 151.7 ((**C**O)OC$(CH_3)_3$), 85.8 ((CO)O**C**$(CH_3)_3$), 53.1 (C2), 44.0 (C4), 27.7 (3C, (CO)OC(**C**H_3)$_3$), 24.4 (C3), 22.4 (CO**C**H_3). **HRMS-ESI** [M+H]$^+$ calc. for $C_{11}H_{19}N_2O_4^+$ 243.1339; obs. 243.1336.

IR (solid, cm^{-1}): 3312, 2976, 1777, 1686, 1638, 1511.

18 was also observed as a by-product during the synthesis of **Ac-Dab(Boc)-OH** (Supplementary Figure 93) and could be isolated by column chromatography.

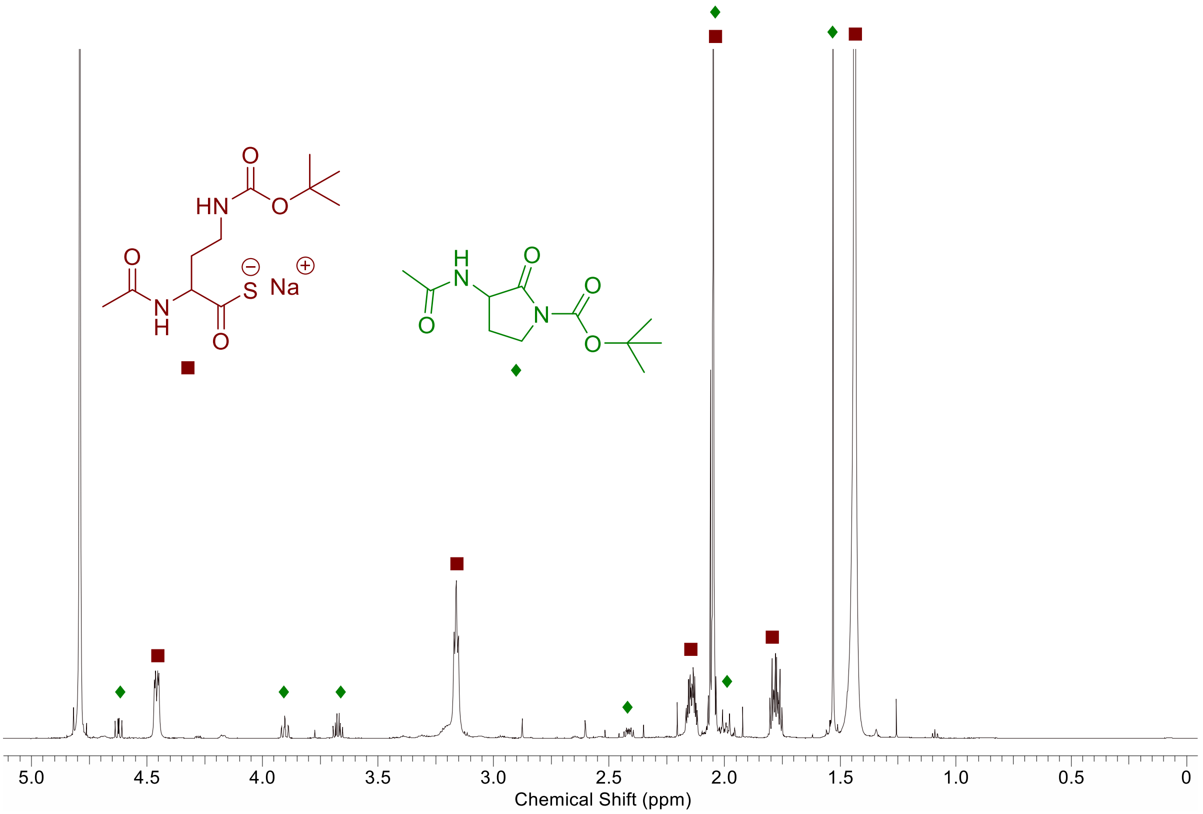

*Supplementary Figure 108. ^{1}H NMR (700 MHz, D_2O, 0.0 – 5.0 ppm) spectrum of **Ac-Dab(Boc)-S$^-$Na$^+$** and lactam **18**.*

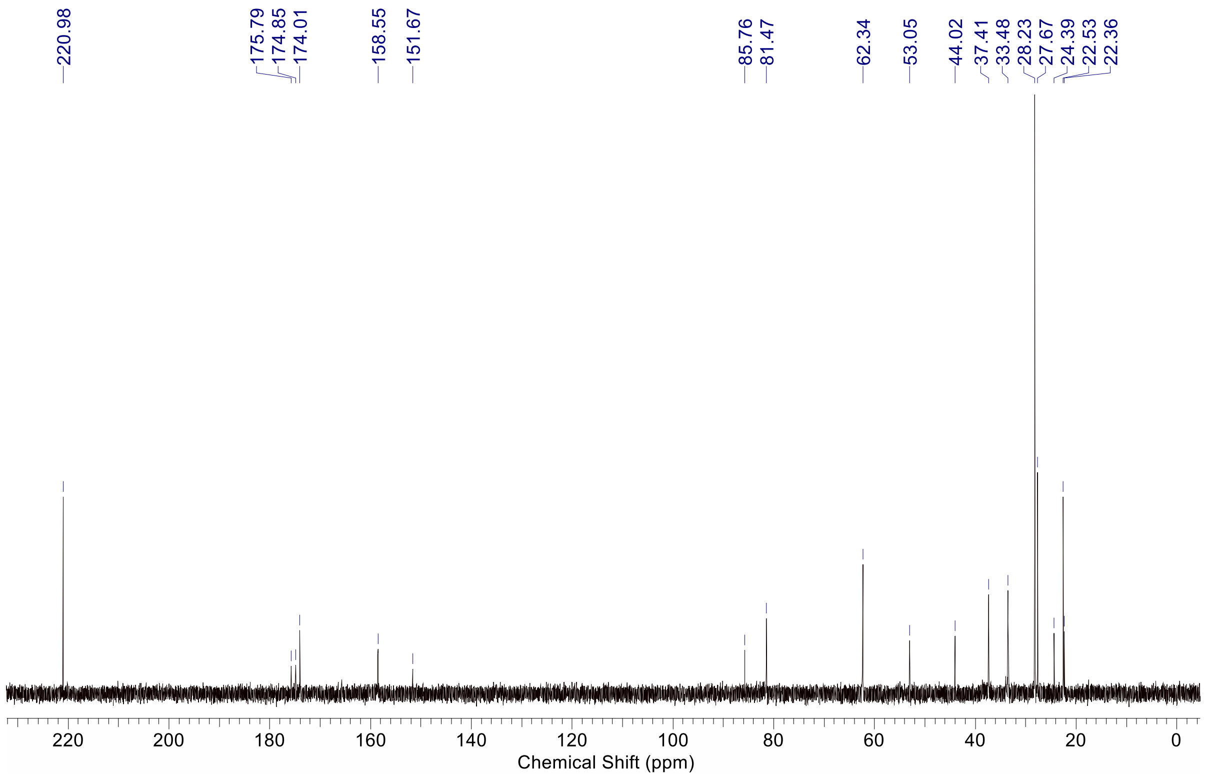

Supplementary Figure 109. ^{13}*C NMR (176 MHz,* D_2O*, 0 – 230 ppm) spectrum of* ***Ac-Dab(Boc)-S⁻Na⁺*** *and* ***18****.*

Ac-Dpr(Boc)-S^-Na^+

Prepared according to general procedure **E** and **F** using **Ac-Dpr(Boc)-OH** (52.4 mg, 0.21 mmol) to afford **Ac-Dpr(Boc)-S^-Na^+** (37.0 mg, 80% pure by ^{1}H NMR, 0.11 mmol, 54% over 2 steps). **^{1}H NMR** (700 MHz, D_2O) δ_H 4.56-4.62 (br ABX, 1H, (C2)–H), 3.61-3.67 (br ABX, 1H, (C3)–H), 3.30 (ABX, J = 14.2, 7.8 Hz, 1H, (C3)–H′), 2.01 (s, 3H, $COCH_3$), 1.40 (br s, 9H, $(CO)OC(CH_3)_3$). **^{13}C NMR** (176 MHz, D_2O) δ_C 217.7 (C1), 174.0 (**C**OCH_3), 158.7 ((**C**O)$OC(CH_3)_3$), 81.7 ((CO)O**C**$(CH_3)_3$), 64.4 (C2), 43.7 (C3), 28.2 (3C, (CO)OC(**C**$H_3)_3$), 22.7 (CO**C**H_3). **HRMS-ESI** $[M+H]^+$ calc. for $C_{10}H_{19}N_2O_4S^+$ 263.1060; obs. 263.1060. **IR** (solid, cm^{-1}): 3306 (br), 2977, 1691, 1648, 1507 (br).

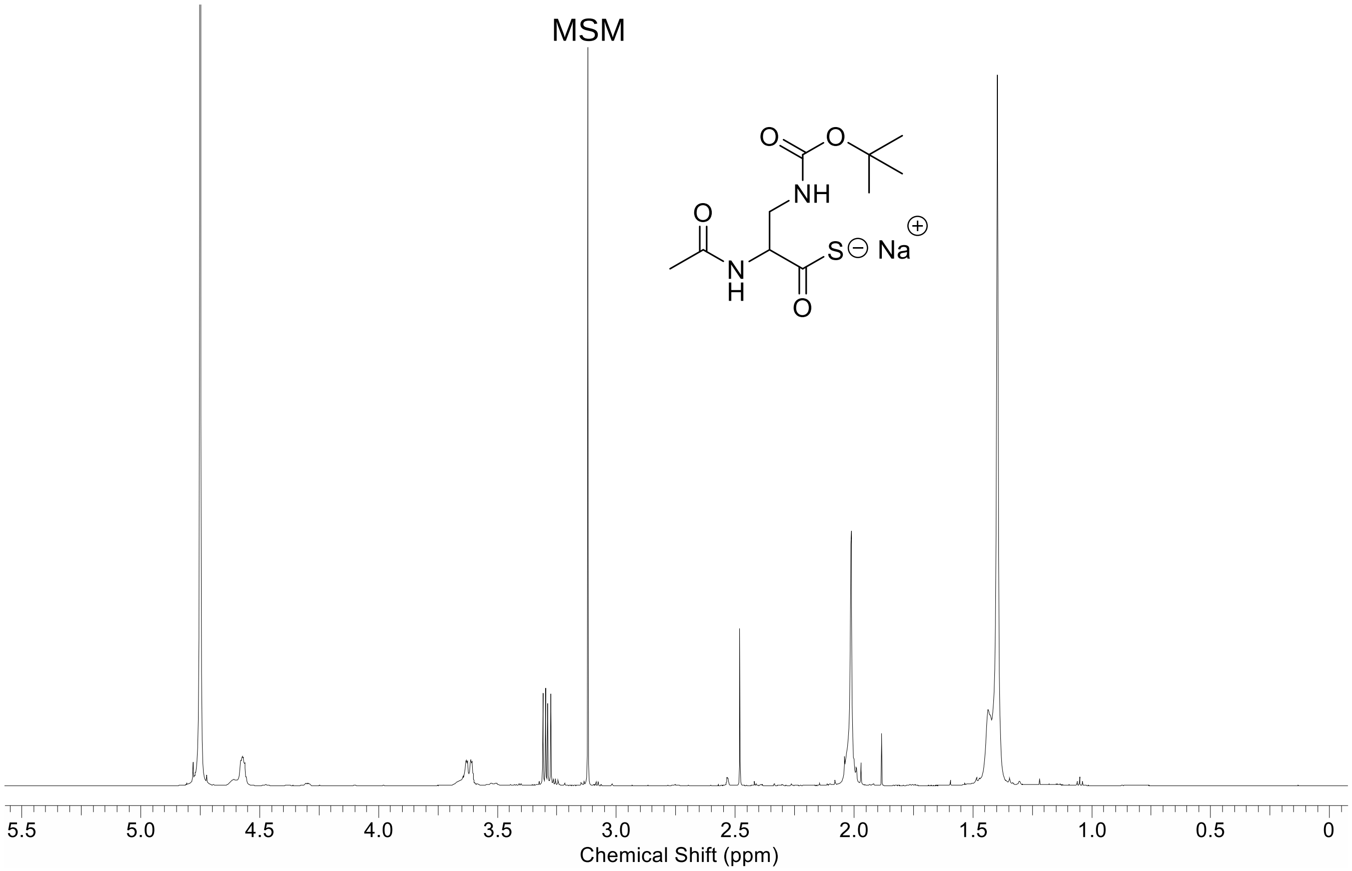

*Supplementary Figure 110. ^{1}H NMR (700 MHz, D_2O, 0.0 – 5.0 ppm) spectrum of **Ac-Dpr(Boc)-S^-Na^+**.*

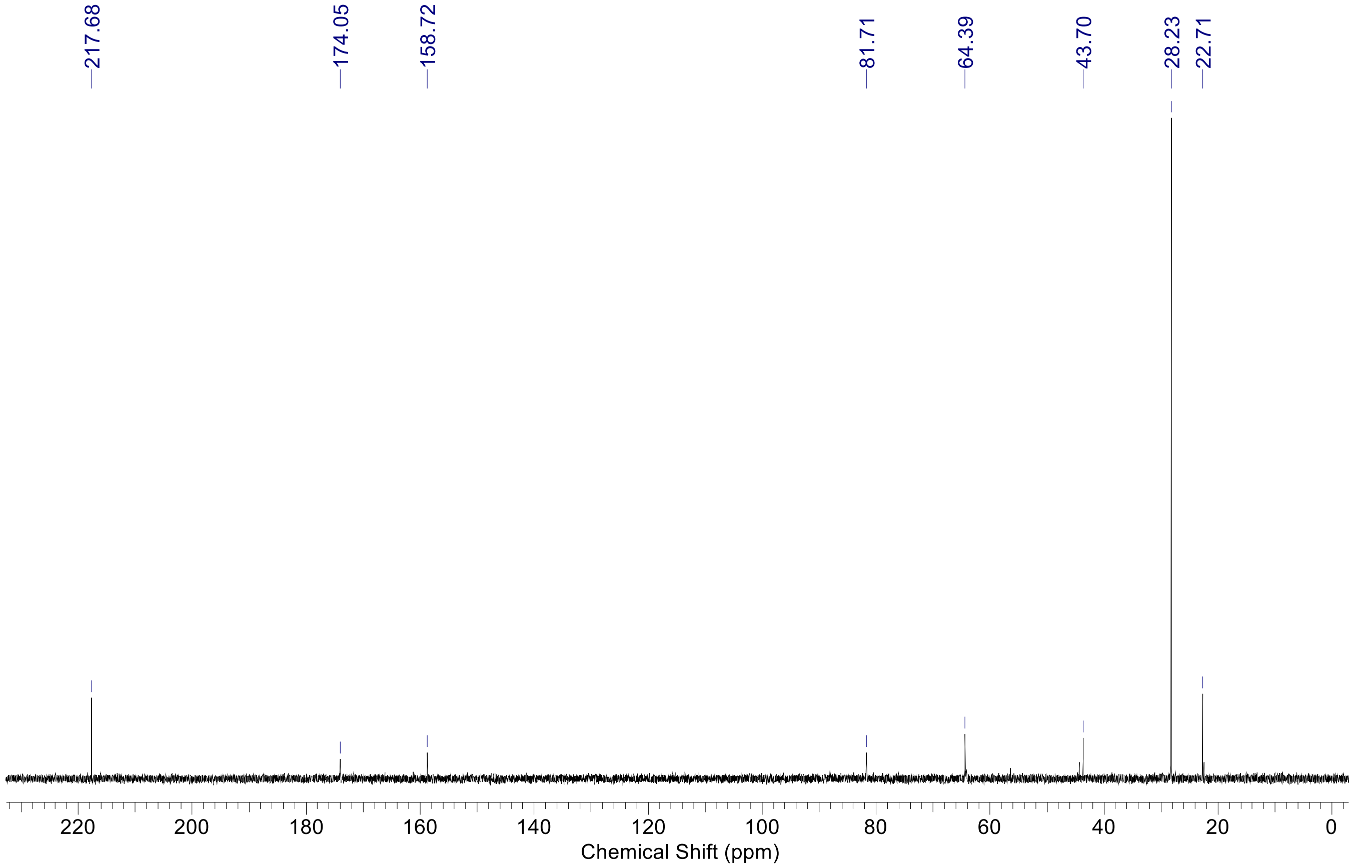

Supplementary Figure 111. ^{13}C NMR (176 MHz, D_2O, 0 – 230 ppm) spectrum of ***Ac-Dpr(Boc)-S$^-$Na$^+$****.*

Synthesis of Ac-AA(Boc)-Gly-S⁻Na⁺

Ac-Lys(Boc)-Gly-OMe[9]

To **Ac-Lys(Boc)-OH** (790 mg, 2.74 mmol) and EDC·HCl (1.05 g, 5.5 mL) in CH_2Cl_2 (50.0 mL) was added **Gly-OMe**·HCl (690 mg, 5.5 mmol) followed by NEt_3 (840 µL, 6.00 mmol). The reaction mixture was stirred at room temperature for 16 h and washed with 100 mM aq. citrate (3 × 25 mL) followed by brine (1 × 25 mL). The organic layer was dried over anhydrous Na_2SO_4, filtered and the solution concentrated *in vacuo* to afford the title compound as a white powder that was used without purification (800 mg, 2.23 mmol, 81%). **^{1}H NMR** (400 MHz, $CDCl_3$) δ_H 6.99 (br. t, 1H, NH), 6.46 (d, *J* = 7.2 Hz, 1H, NH), 4.74 (br. s, 1H, NH), 4.48 (app q, *J* = 7.5 Hz, 1H, Lys-(C2)–H), 4.05 (ABX, *J* = 18.1, 5.5 Hz, 1H, Gly-(C2)–H), 3.98 (ABX, *J* = 18.1, 5.3 Hz, Gly-(C2)–H′) 3.75 (s, 3H, $COOCH_3$), 3.11 (br. m, 2H, Lys-(C6)–H_2), 2.03 (s, 3H, $COCH_3$), 1.82-1.91 (m, 1H, Lys-(C3)–H), 1.64-1.73 (m, 1H, Lys-(C3)–H′), 1.35-1.54 (overlapping m, 4H, Lys-(C4)–H_2, Lys-(C5)–H_2), 1.44 (s, 9H, (CO)OC$(CH_3)_3$). **^{13}C NMR** (100 MHz, $CDCl_3$) δ_C 172.1 (Lys-C1), 170.6 (**C**OCH_3), 170.0 (Gly-C1), 156.3 ((**C**O)OC$(CH_3)_3$), 79.2 ((CO)O**C**$(CH_3)_3$), 52.9 (Lys-C2). 52.4 (COO**C**H_3), 41.1 (Gly-C2), 40.0 (Lys-C6), 31.7 (Lys-C3), 29.6 (Lys-C5), 28.4 (3C, (CO)OC(**C**H_3)$_3$), 23.1 (CO**C**H_3), 22.3 (Lys-C4). **HRMS-ESI** $[M+H]^+$ calc. for $C_{16}H_{30}N_3O_6^+$ 360.2129; obs. 360.2125. **IR** (solid, cm^{-1}): 3282, 2935, 1738, 1728, 1681, 1631, 1516. Characterisation data were in accordance with literature values.[9]

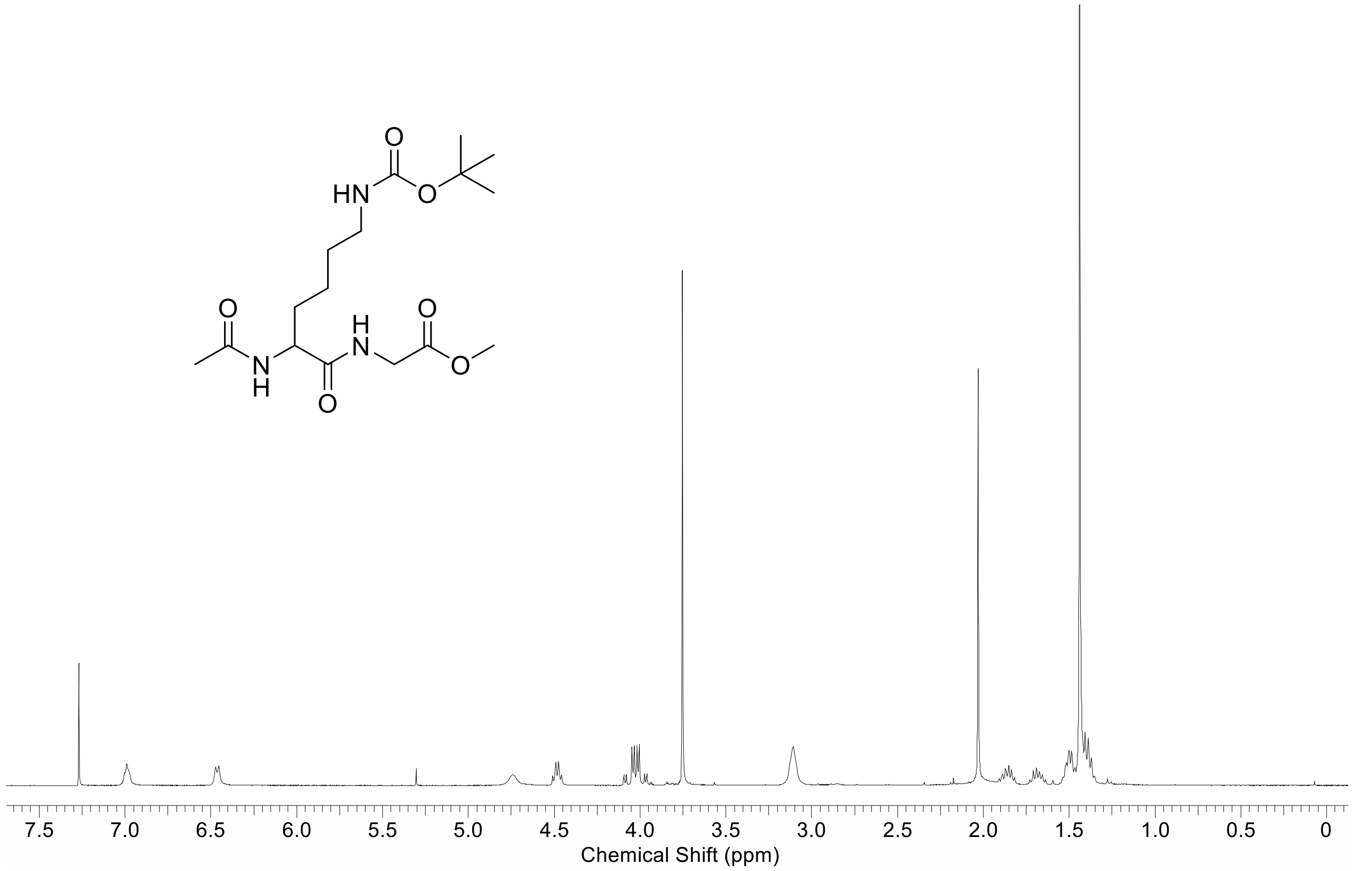

*Supplementary Figure 112. ^{1}H NMR (400 MHz, $CDCl_3$, 0.0 – 7.5 ppm) spectrum of **Ac-Lys(Boc)-Gly-OMe**.*

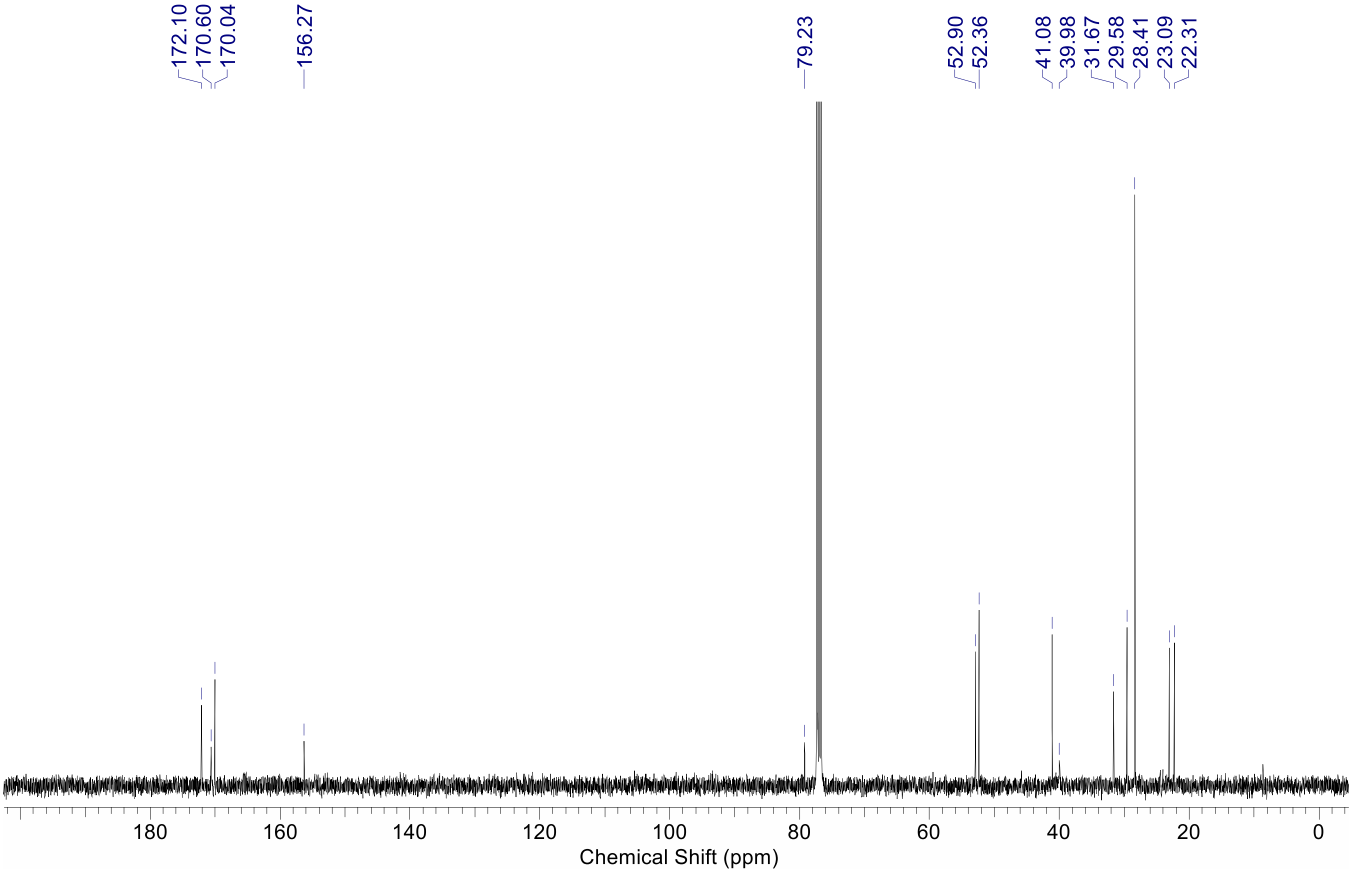

*Supplementary Figure 113. ^{13}C NMR (101 MHz, $CDCl_3$, 0 – 200 ppm) of **Ac-Lys(Boc)-Gly-OMe**.*

Ac-Lys(Boc)-Gly-OH

To **Ac-Lys(Boc)-Gly-OMe** (600 mg, 1.67 mmol) in MeOH (15.0 mL) was added 2 M KOH (15 mL). The reaction mixture was stirred for 1 h at room temperature. The solution was adjusted to pH 2.0-2.5 with 0.5 M/12 M HCl and the solvent reduced *in vacuo* to ~ 30 mL. The solution was extracted with CH_2Cl_2/^{i}PrOH (9:1, 6 × 15 mL), the combined organic extracts dried over anhydrous Na_2SO_4, filtered and the solvent removed *in vacuo* to afford the title compound as a white powder (464 mg, 1.34 mmol, 80%). **^{1}H NMR** (600 MHz, CD_3OD) δ_H 4.34 (dd, *J* = 8.7, 5.3 Hz, 1H, Lys-(C2)–H), 3.95 (AB, *J* = 17.7 Hz, 1H, Gly-(C2)–H), 3.86 (AB, *J* = 17.7 Hz, 1H, Gly-(C2)–H′), 3.03 (app t, *J* = 6.8 Hz, 2H, Lys-(C6)–H_2), 1.99 (s, 3H, $COCH_3$), 1.80-1.86 (m, 1H, Lys-(C3)–H), 1.62-1.69 (m, 1H, Lys-(C3)–H′), 1.43 (s, 9H, (CO)OC$(CH_3)_3$), 1.35-1.53 (overlapping m, 4H, Lys-(C4)–H_2, Lys-(C5)–H_2). **^{13}C NMR** (151 MHz, CD_3OD) δ_C 175.0 (Lys-C1), 173.5 (**C**OCH_3), 172.9 (Gly-C1), 158.7 ((**C**O)OC$(CH_3)_3$), 80.0 ((CO)O**C**$(CH_3)_3$), 54.8 (Lys-C2), 41.9 (Gly-C2), 41.2 (Lys-C6), 32.9 (Lys-C3), 30.7 (Lys-C5), 28.9 (3C, (CO)OC(**C**$H_3)_3$), 24.2 (Lys-C4), 22.6 (CO**C**H_3). **HRMS-ESI** $[M+H]^+$ calc. for $C_{15}H_{28}N_3O_6^+$ 346.1973; obs. 346.1969. **IR** (solid, cm^{-1}): 3297, 2934, 1645 (br), 1521 (br). **R_f** = 0.33 in 1:1 EtOAc/MeOH.

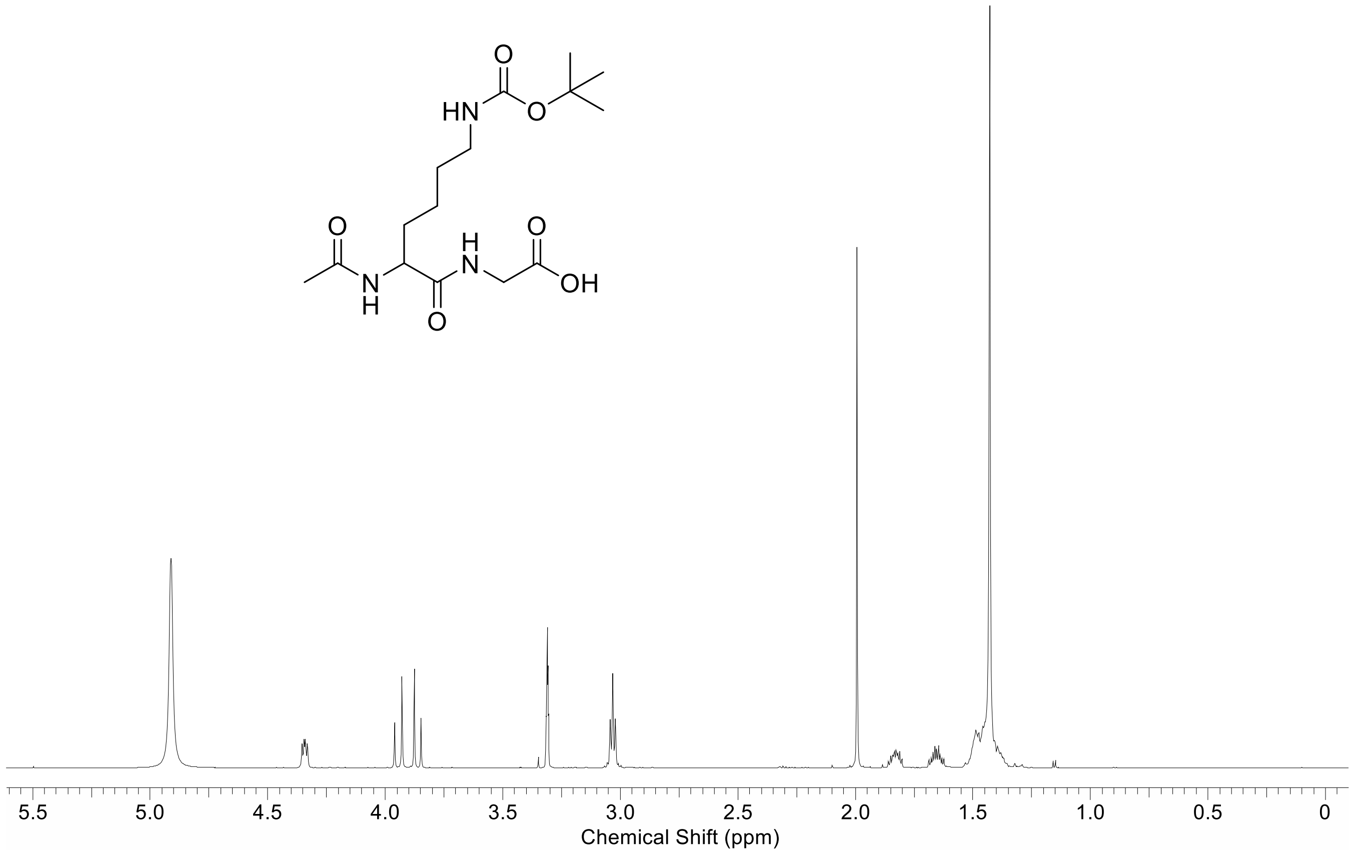

Supplementary Figure 114. ^{1}H NMR (600 MHz, CD_3OD, 0.0 – 5.5 ppm) spectrum of ***Ac-Lys(Boc)-Gly-OH****.*

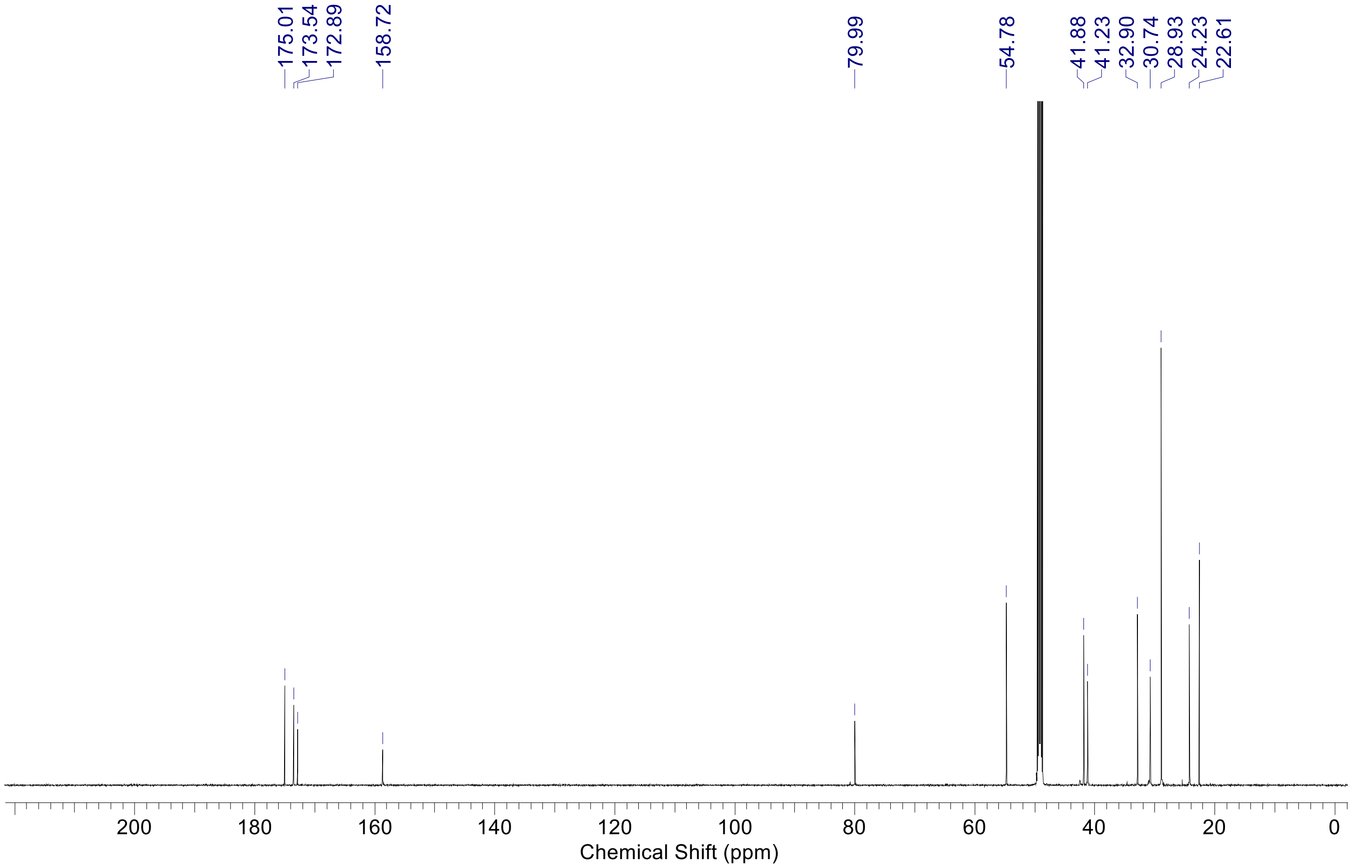

*Supplementary Figure 115. ^{13}C NMR (151 MHz, CD_3OD, 0 – 220 ppm) spectrum of **Ac-Lys(Boc)-Gly-OH**.*

Ac-Lys(Boc)-Gly-S⁻Na⁺

Ac-Lys(Boc)-Gly-OH (100 mg, 0.29 mmol), EDC·HCl (167 mg, 0.87 mmol) and pentafluorophenol (58.9 mg, 0.32 mmol) were stirred in CH_2Cl_2 (7.00 mL) at room temperature for 4 h under an N_2 atmosphere. The solvent was removed *in vacuo*, and the crude residue purified by column chromatography (gradient, CH_2Cl_2 to MeCN) to afford **Ac-Lys(Boc)-Gly-OPFP** (51.8 mg, 0.10 mmol, 35%) which was used immediately in the following step. To **Ac-Lys(Boc)-Gly-OPFP** (45.7 mg, 0.09 mmol) in MeCN (2.50 mL) was added NaSH·xH_2O (10.0 mg, 0.09 mmol). The reaction mixture was stirred for 4 h at room temperature under an N_2 atmosphere, centrifuged and the pellet resuspended in Et_2O (2.0 mL) and the suspension centrifuged again. The resulting pellet was dried *in vacuo* to afford **Ac-Lys(Boc)-Gly-S^-Na^+** (80% pure by ^{1}H NMR spectroscopy, 25.1 mg, 0.05 mmol, 56%) as a white powder. **^{1}H NMR** (700 MHz, D_2O) δ_H 4.33 (dd, *J* = 9.1, 5.0 Hz, 1H, Lys-(C2)–H), 4.16 (AB, *J* = 18.3 Hz, 1H, Gly-(C2)–H), 4.15 (AB, *J* = 18.3 Hz, 1H, Gly-(C2)–H′), 3.10 (br. t, *J* = 5.7 Hz, 2H, Lys-(C6)–H_2), 2.08 (s, 3H, $COCH_3$), 1.86-1.91 (m, 1H, Lys-(C3)–H), 1.72-1.77 (m, 1H, Lys-(C3)–H′), 1.46 (s, 9H, (CO)OC$(CH_3)_3$), 1.37-1.57 (overlapping m, 4H, Lys-(C4)–H_2, Lys-(C5)–H_2). **^{13}C NMR** (176 MHz, D_2O) δ_C 216.1 (Gly-C1), 174.9 (**C**OCH_3), 174.4 (Lys-C1), 158.9 ((**C**O)OC$(CH_3)_3$), 81.4 ((CO)O**C**$(CH_3)_3$), 54.4 (Lys-C2), 53.8 (Gly-C2), 40.3 (Lys-C6), 31.4 (Lys-C3), 29.0 (Lys-C5), 28.3 (3C, (CO)OC(**C**H_3)$_3$), 22.9 (Lys-C4), 22.3 (CO**C**H_3). **HRMS-ESI** $[M+H]^+$ for $C_{15}H_{26}N_3O_5SNa^+$ 384.1564; obs. 384.1564. **IR** (solid, cm^{-1}): 3336, 2939, 1686, 1623, 1541 (br).

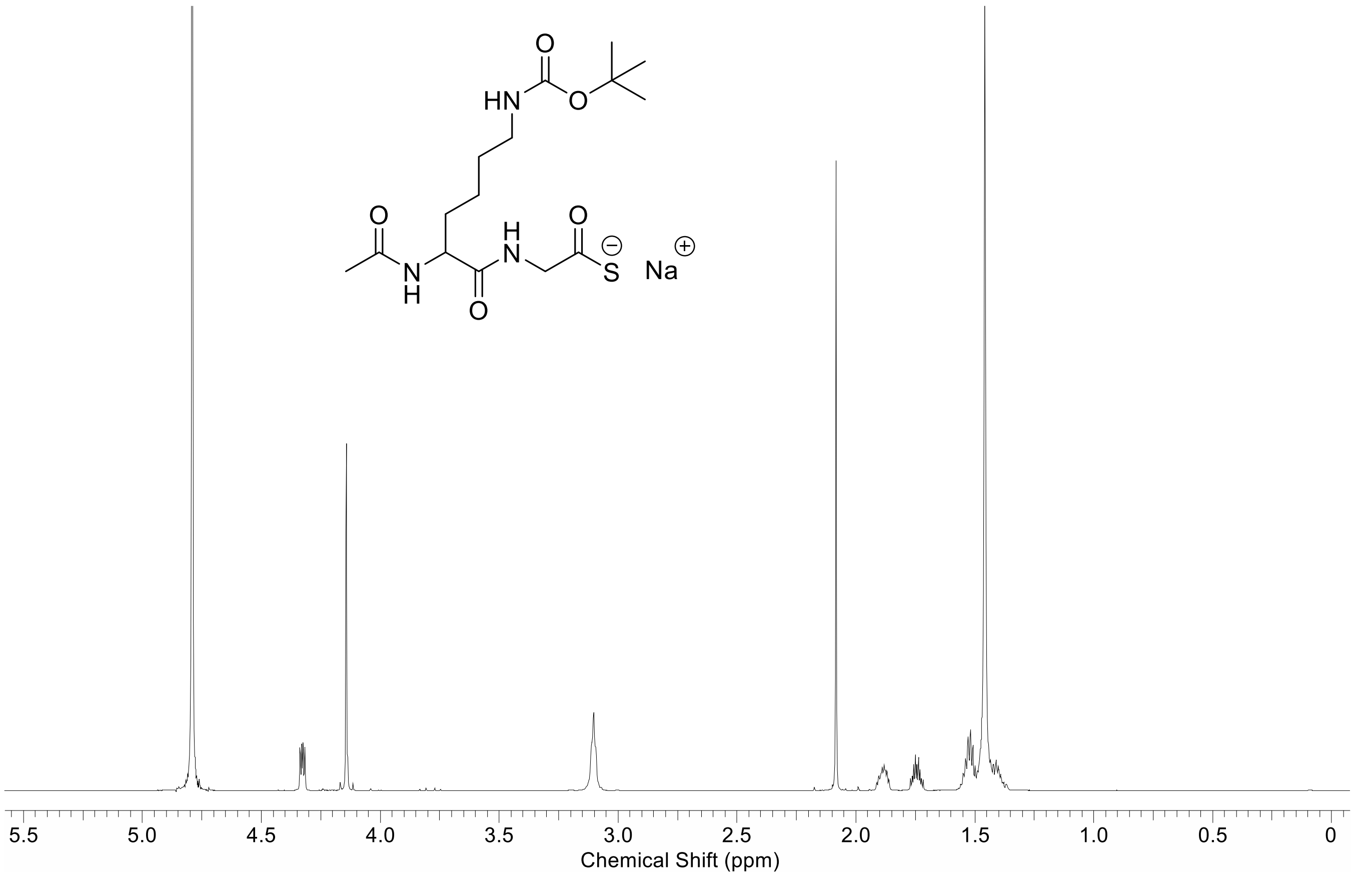

*Supplementary Figure 116. ^{1}H NMR (700 MHz, D_2O, 0.0 – 5.5 ppm) spectrum of **Ac-Lys(Boc)-Gly-S^-Na^+**.*

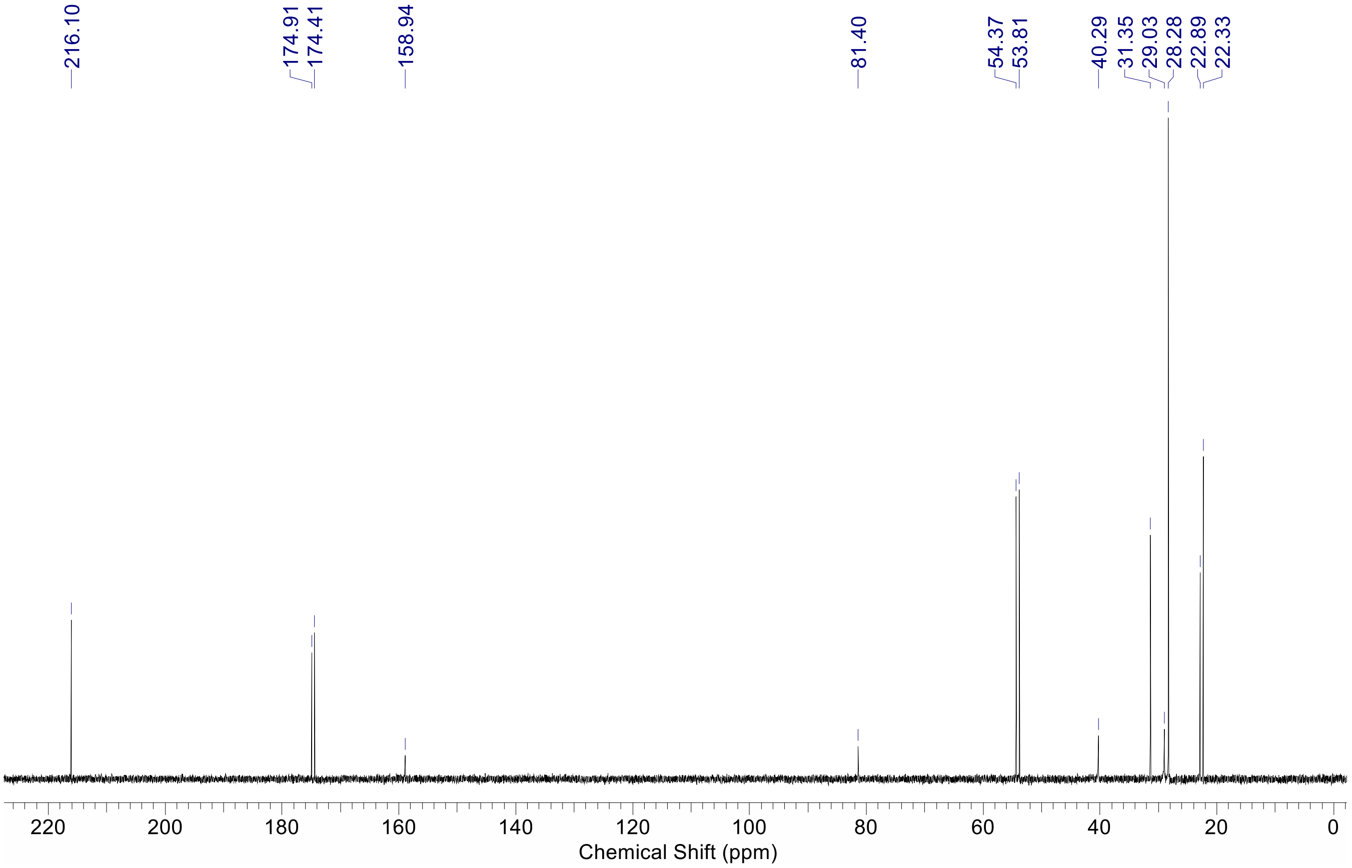

Supplementary Figure 117. ^{13}*C NMR (176 MHz,* D_2O*, 0 – 225 ppm) spectrum of* ***Ac-Lys(Boc)-Gly-S⁻Na⁺***.

Ac-Dpr(Boc)-Gly-OMe

To **Ac-Dpr(Boc)-OH** (101 mg, 0.41 mmol) and EDC·HCl (157 mg, 0.82 mmol) in CH_2Cl_2 (8.00 mL) was added **Gly-OMe**·HCl (103 mg, 0.82 mmol) followed by NEt_3 (126 µL, 0.90 mmol). The reaction mixture was stirred under an N_2 atmosphere for 17 h at room temperature. The reaction mixture was then washed with 100 mM aq. citrate (3 × 4.0 mL) followed by brine (1 × 4.0 mL). The combined aqueous layers were extracted with CH_2Cl_2/^{i}PrOH (9:1, 6 × 4.0 mL). All organic layers were combined and dried over anhydrous Na_2SO_4, filtered and the solvent removed *in vacuo* to afford the crude product. Purification by column chromatography (EtOAc) afforded **Ac-Dpr(Boc)-Gly-OMe** (86.6 mg, 0.27 mmol, 66%) as a white powder. **^{1}H NMR** (400 MHz, $CDCl_3$) δ_H 7.45 (br s, 1H, NH), 7.18 (br s, 1H, NH), 5.38 (br s, 1H, NH), 4.51 (br dd, *J* = 10.7, 5.5 Hz, 1H, Dpr-(C2)–H), 4.05 (ABX, *J* = 18.0, 5.8 Hz, Gly-(C2)–H), 3.97 (ABX, *J* = 18.0, 5.3 Hz, Gly-(C2)–H′), 3.74 (s, 3H, $COOCH_3$), 3.51 (br m, 2H, Dpr-(C3)–H_2), 2.04 (s, 3H, $COCH_3$), 1.44 (s, 9H, (CO)OC(CH_3)$_3$). **^{13}C NMR** (151 MHz, $CDCl_3$) δ_C 171.6 (**C**OCH_3), 170.6 (Dpr-C1), 169.9 (Gly-C1), 157.7 ((**C**O)OC(CH_3)$_3$), 80.3 ((CO)O**C**(CH_3)$_3$), 55.2 (Dpr-C2), 52.4 (COO**C**H_3), 42.0 (Dpr-C3), 41.2 (Gly-C2), 28.2 (3C, (CO)OC(**C**H_3)$_3$), 23.2 (CO**C**H_3). **HRMS-ESI** $[M+H]^+$ calc. for $C_{13}H_{24}N_3O_6^+$ 318.1660; obs. 318.1661. **IR** (solid, cm^{-1}): 3322, 2948, 1751, 1696, 1646, 1529, 1366. **R$_f$** = 0.23 in EtOAc.

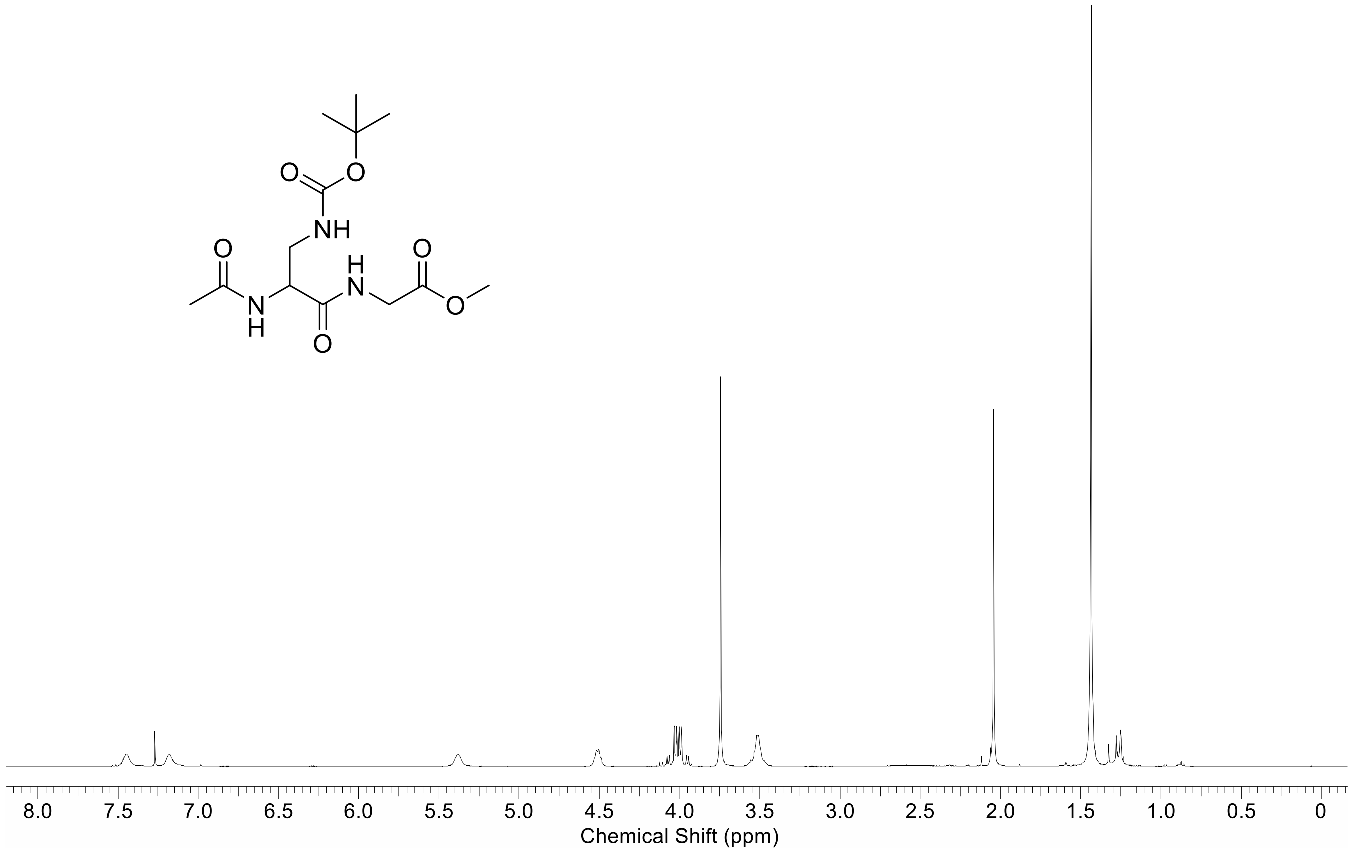

*Supplementary Figure 118. ^{1}H NMR (400 MHz, $CDCl_3$, 0.0 – 8.0 ppm) spectrum of **Ac-Dpr(Boc)-Gly-OMe**.*

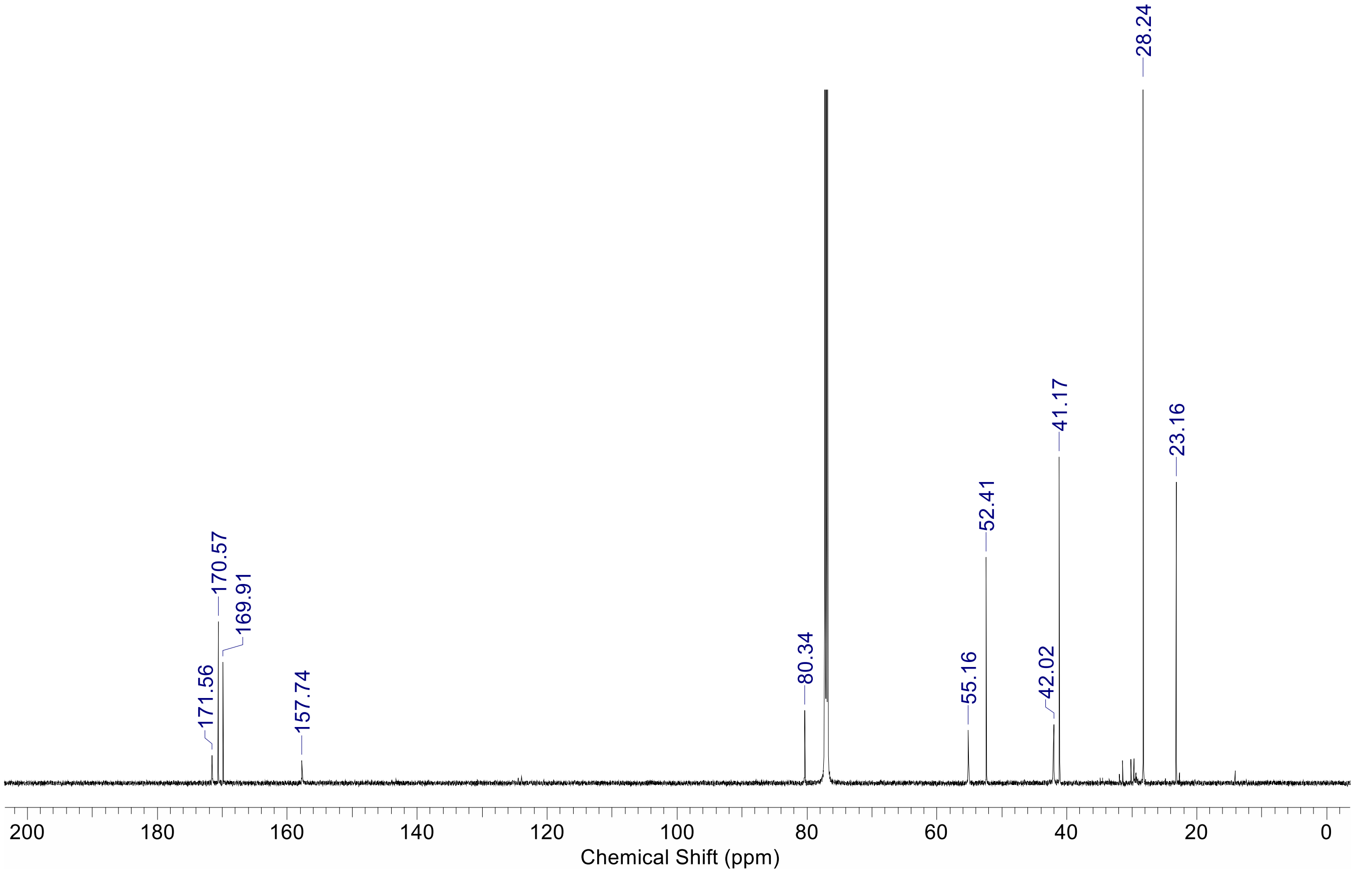

Supplementary Figure 119. 13C NMR (151 MHz, $CDCl_3$, 0 – 200 ppm) spectrum of ***Ac-Dpr(Boc)-Gly-OMe****.*

Ac-Dpr(Boc)-Gly-OH

To **Ac-Dpr(Boc)-Gly-OMe** (86.0 mg, 0.27 mmol) in MeOH (2.50 mL) was added 2 M NaOH (2.50 mL) dropwise at 0 °C. The reaction mixture was warmed to room temperature over 15 min, adjusted to pH 3 with 1 M HCl and then reduced *in vacuo* to dryness, co-evaporating with toluene (2 × 2.0 mL). The crude residue was washed with acetone (3 × 5.0 mL), filtered and the solvent removed *in vacuo* to afford **Ac-Dpr(Boc)-Gly-OH** (79.3 mg, 0.26 mmol, 97%) as a white solid. **^{1}H NMR** (400 MHz, D_2O) δ_H 4.53 (app. t, *J* = 5.9 Hz, 1H, Dpr-(C2)–H), 3.97 (AB, *J* = 17.8 Hz, 1H, Gly-(C2)–H), 3.91 (AB, *J* = 17.8 Hz, 1H, Gly-(C2)–H'), 3.54 (br. ABX, *J* = 14.4, 5.0 Hz, 1H, Dpr-(C3)–H), 3.37 (ABX, *J* = 14.4 Hz, 7.4 Hz, 1H, Dpr-(C3)–H'), 2.06 (s, 3H, $COCH_3$), 1.43 (s, 9H, (CO)OC(CH_3)$_3$). **^{13}C NMR** (176 MHz, CD_3OD) δ_C 173.6 (**C**OCH_3), 172.9 (Gly-C1/Dpr-C1), 172.9 (Gly-C1/Dpr-C1), 158.8 ((**C**O)OC(CH_3)$_3$), 80.6 ((CO)O**C**(CH_3)$_3$), 55.2 (Dpr-C2), 43.1 (Dpr-C3), 42.0 (Gly-C2), 28.8 (3C, (CO)OC(**C**H$_3$)$_3$), 22.8 (CO**C**H$_3$). **HRMS-ESI** [M+H]$^+$ calc. for $C_{12}H_{22}N_3O_6^+$ 304.1503; obs. 304.1501. **IR** (solid, cm^{-1}): 3324, 1735, 1678, 1642, 1523.

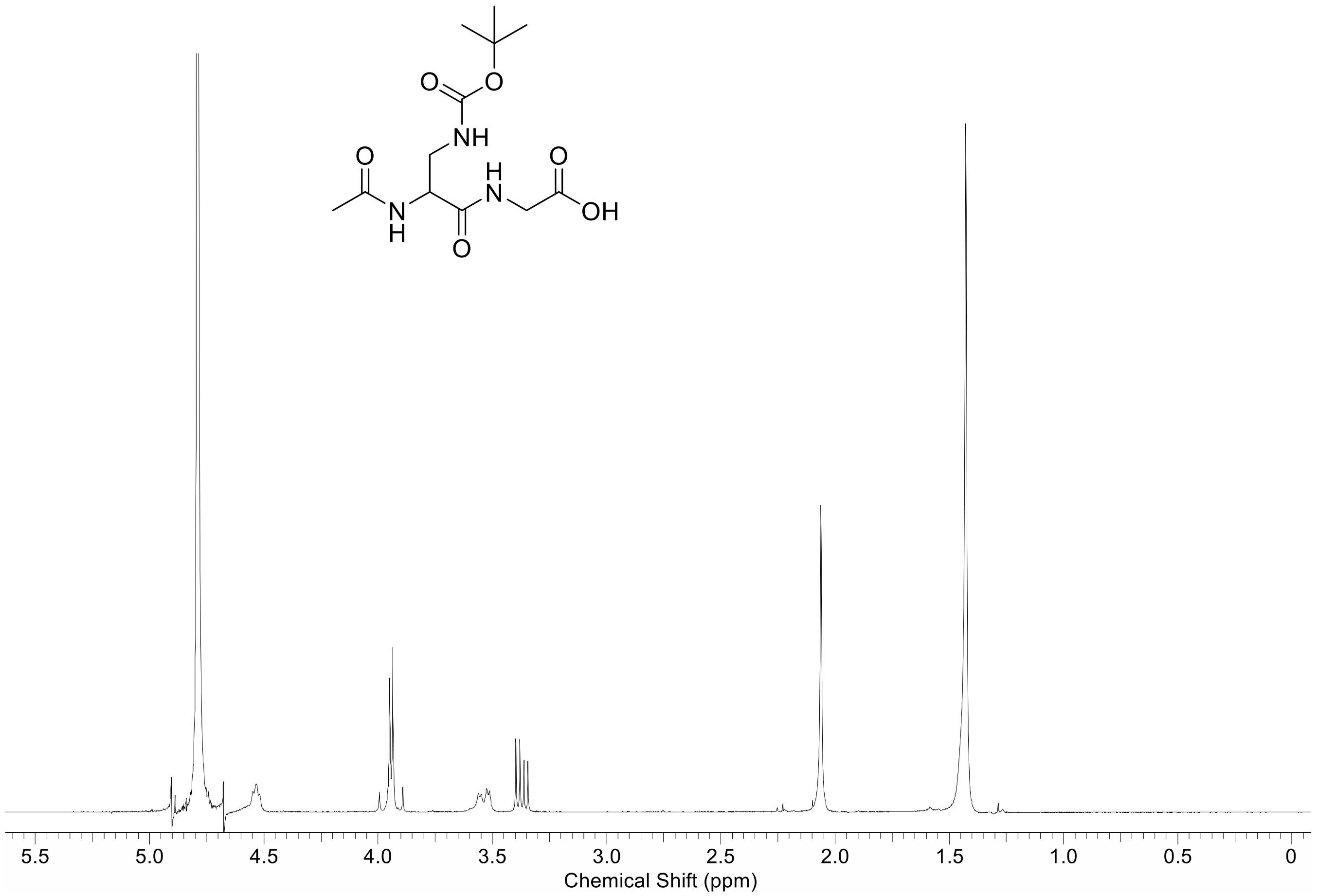

*Supplementary Figure 120. ^{1}H NMR (400 MHz, D_2O, 0.0 – 5.5 ppm) spectrum of **Ac-Dpr(Boc)-Gly-OH**.*

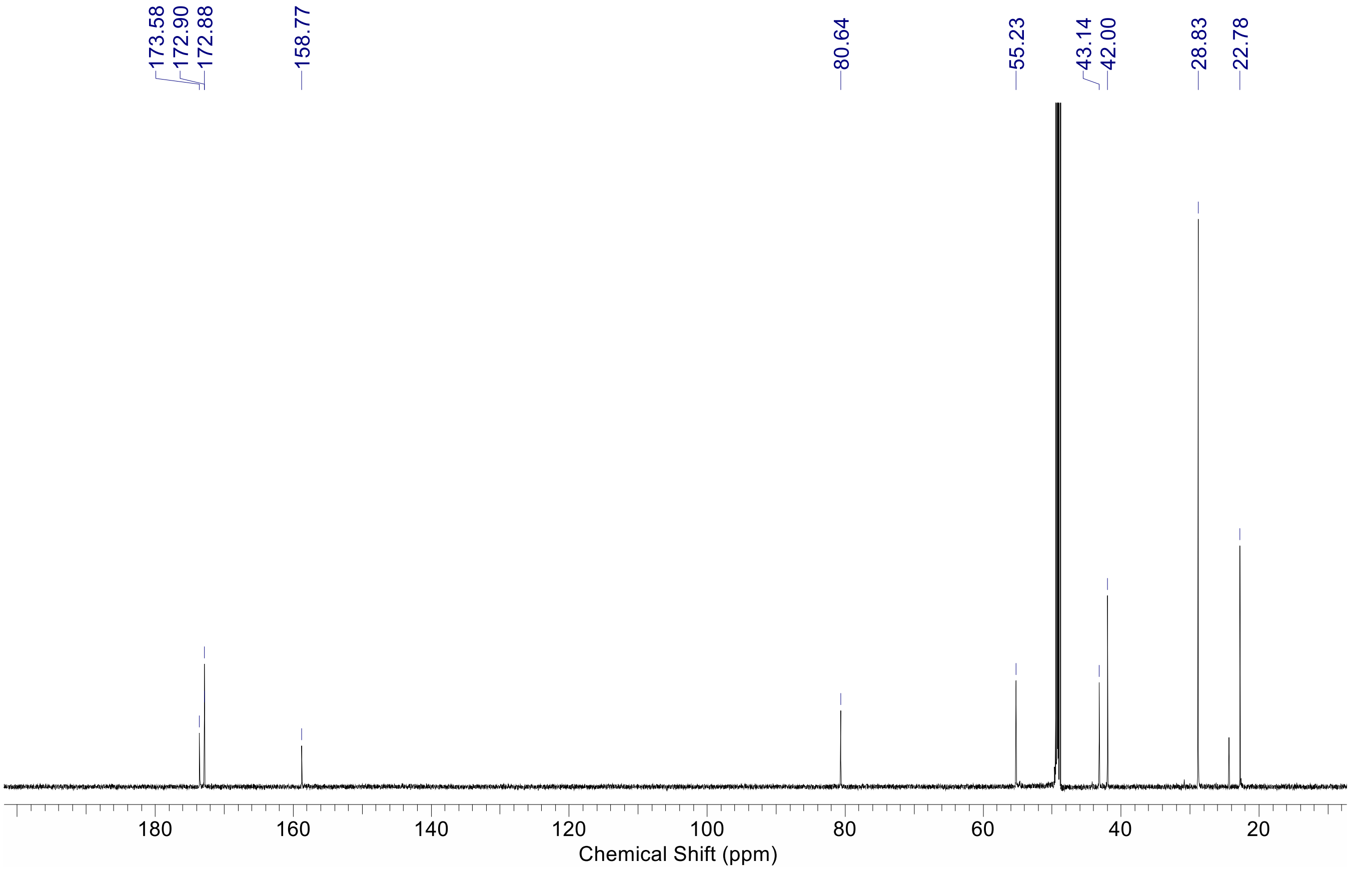

Supplementary Figure 121. ^{13}C NMR (176 MHz, CD_3OD, 10 – 200 ppm) spectrum of ***Ac-Dpr(Boc)-Gly-OH****.*

Ac-Dpr(Boc)-Gly-S$^-$Na$^+$

To **Ac-Dpr(Boc)-Gly-OH** (44.0 mg, 0.15 mmol) in CH_2Cl_2 (4.00 mL) was added pentafluorophenol (29.4 mg, 0.16 mmol) and EDC·HCl (36.2 mg, 0.19 mmol) at 0 °C. The reaction mixture was warmed to room temperature and stirred under an N_2 atmosphere for 20 h. The solvent was removed *in vacuo* and the crude residue was purified by column chromatography (gradient, CH_2Cl_2 to 8:2 CH_2Cl_2/MeCN) to afford **Ac-Dpr(Boc)-Gly-OPFP** (15.8 mg, 0.03 mmol, 20%) which was immediately dissolved in MeCN (1.00 mL) and to this stirring solution was added NaSH·xH_2O (3.90 mg, 0.03 mmol) under an Ar atmosphere. The reaction mixture was stirred for 16 h, centrifuged and the pellet washed with MeCN (1 mL), Et_2O (1 mL) and dried *in vacuo* to afford **Ac-Dpr(Boc)-Gly-S$^-$Na$^+$** (11.6 mg, 0.03 mmol, 95% pure by ^{1}H NMR, 96%) as a white powder. **^{1}H NMR** (700 MHz, D_2O) δ_H 4.51 (br app t, 1H, Dpr-(C2)–H), 4.13 (AB, J = 17.8 Hz, 1H, Gly-(C2)–H), 4.08 (AB, J = 17.8 Hz, 1H, Gly-(C2)–H'), 3.52-3.60 (br ABX, 1H, Dpr-(C3)–H), 3.38 (ABX, J = 14.4, 7.3 Hz, Dpr-(C3)–H'), 2.05 (br s, 3H, $COCH_3$), 1.42 (br s, 9H, (CO)OC(CH_3)$_3$). **^{13}C NMR** (176 MHz, D_2O) δ_C 216.3 (Gly-C1), 175.0 (**C**OCH_3), 171.9 (Dpr-C1), 158.7 ((**C**O)OC(CH_3)$_3$), 82.0 ((CO)O**C**(CH_3)$_3$), 54.5 (Dpr-C2), 54.0 (Gly-C2), 41.9 (Dpr-C3), 28.3 (3C, (CO)OC(**C**H_3)$_3$), 22.6 (CO**C**H_3). **HRMS-ESI** [M+H]$^+$ calc. for $C_{12}H_{20}N_3O_5SNa^+$ 342.1094; obs. 342.1085. **IR** (solid, cm^{-1}): 3345, 3314, 1686, 1631, 1562, 1540.

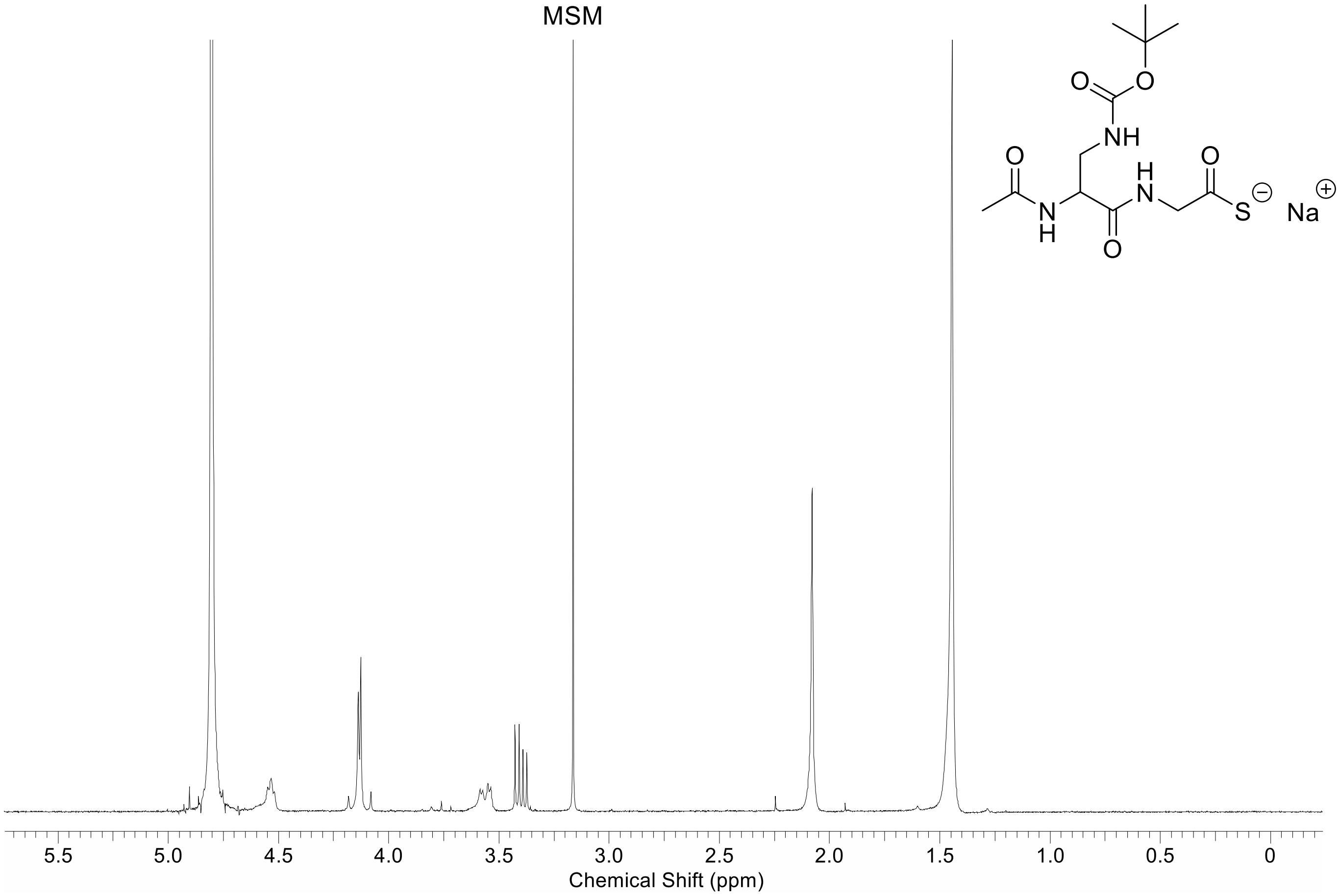

Supplementary Figure 122. ^{1}H NMR (700 MHz, D_2O, 0 – 5.5 ppm) spectrum of ***Ac-Dpr(Boc)-Gly-S$^-$Na$^+$****.*

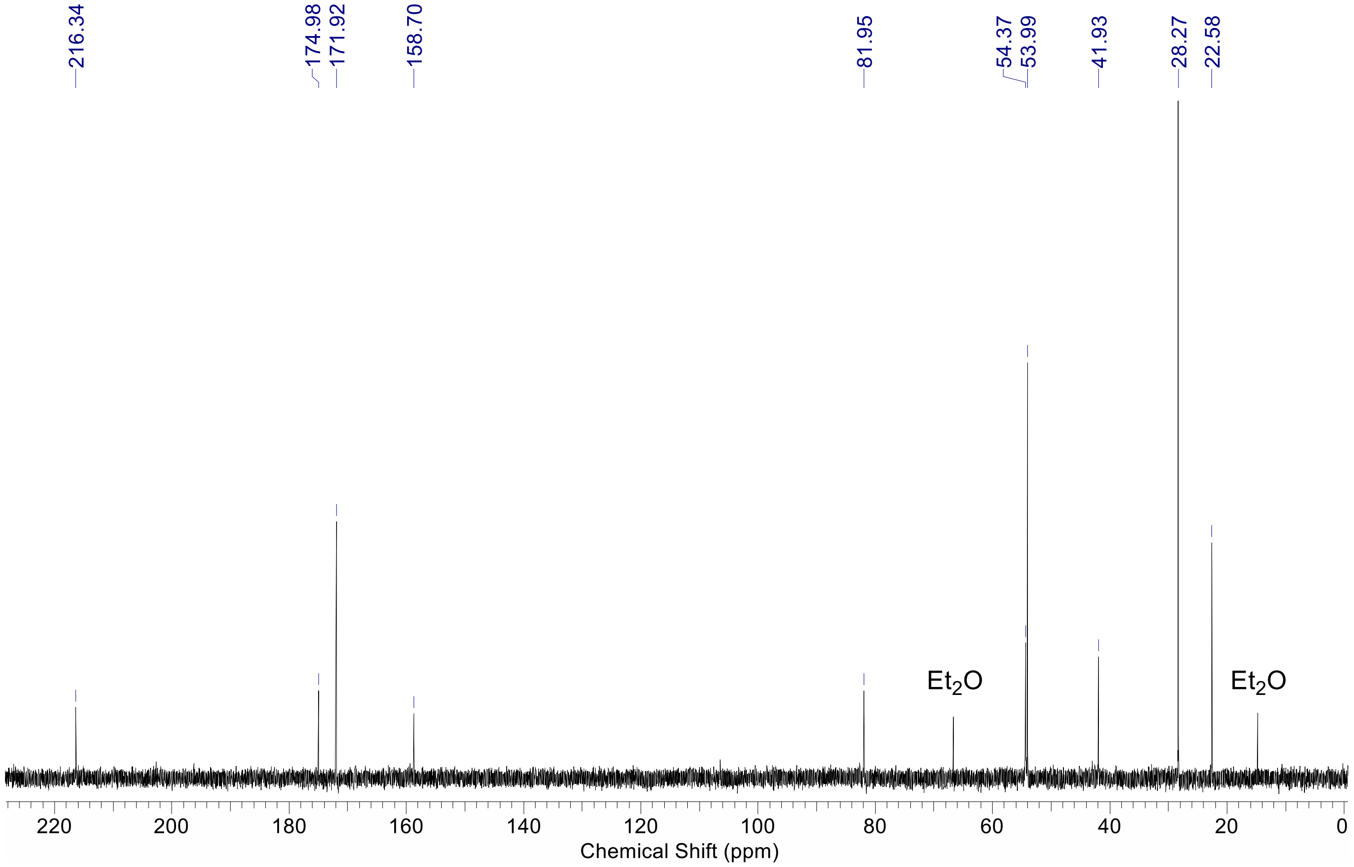

*Supplementary Figure 123. ^{13}C NMR (176 MHz, D_2O, 0 – 225 ppm) spectrum of **Ac-Dpr(Boc)-Gly-S^-Na^+**.*

Synthesis of Ac-AA-$(Gly)_m$-SH from Ac-AA(Boc)-$(Gly)_m$-S^-Na^+

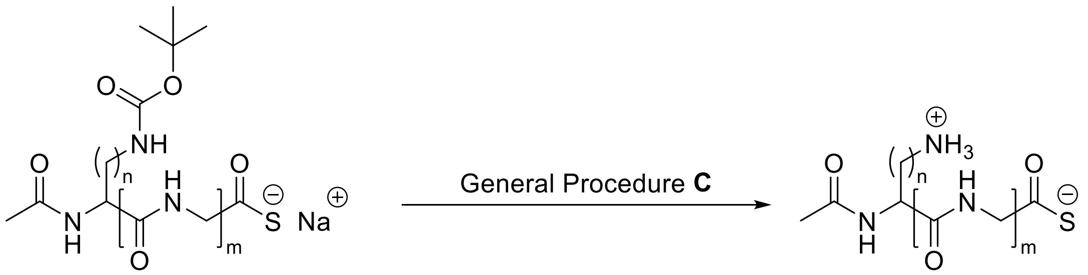

Supplementary Figure 124. Deprotection of ***Ac-AA(Boc)-$(Gly)_m$-S^-Na^+*** *(TFA, rt, 1 min). n = 1–4, m = 0, 1.*

General procedure G: Boc-deprotection of Ac-AA(Boc)-$(Gly)_m$-S^-Na^+ with TFA

Ac-AA(Boc)-$(Gly)_m$-S^-Na^+ (1.00 equiv.) was dissolved in **TFA** (20.0 equiv.) under an N_2 atmosphere and left to stand for 1 min. The volatiles were removed *in vacuo* (T_{bath} = 23-28 °C), coevaporating with toluene (3 × ~ 2 mL). Degassed D_2O (9.4 mL/mmol) and 4 M NaOD (0.78 mL/mmol) were added concurrently and the solution was immediately adjusted to pD 7.5 with 4 M NaOD/HCl and transferred to a separate flask. An aliquot (5-50 µL) was taken and diluted with degassed D_2O (500 µL) and 1 M MSM to determine the molarity of the stock solution.

Synthesis of Ac-Lys-SH [2]

Prepared according to general procedure **G** using **Ac-Lys(Boc)-S⁻Na⁺** (24.2 mg, 0.065 mmol in thioacid) to afford **Ac-Lys-SH** (90% by ^{1}H NMR spectroscopy). **^{1}H NMR** (700 MHz, D_2O) δ_H 4.40 (dd, *J* = 8.9, 4.7 Hz, 1H, (C2)–H), 2.98 (app td, *J* = 8.1, 3.5 Hz, 2H, (C6)–H_2), 2.01 (s, 3H, $COCH_3$), 1.89-1.94 (m, 1H, (C3)–H), 1.63-1.71 (m, 3H, (C3)–H', (C5)–H_2), 1.36-1.45 (m, 2H, (C4)–H_2). **^{13}C NMR** (176 MHz, D_2O) δ_C 221.3 (C1), 174.1 (**C**OCH_3), 64.4 (C2), 39.9 (C6), 33.0 (C3), 27.0 (C5), 22.7 (C4), 22.5 (**C**OCH_3). **HRMS-ESI** $[M+H]^+$ calc. for $C_8H_{17}N_2O_2S^+$ 205.1005; obs. 205.1005.

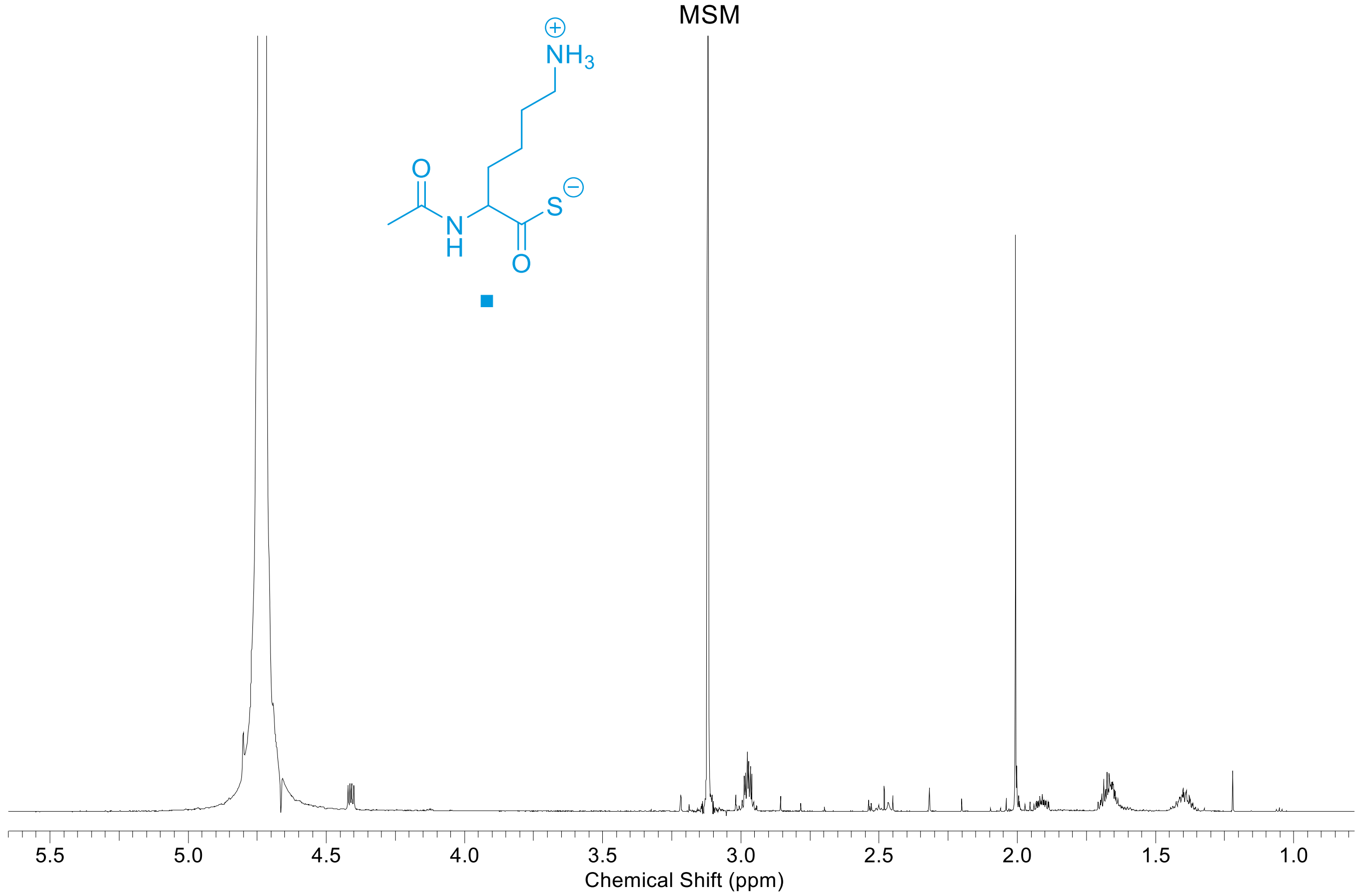

*Supplementary Figure 125. ^{1}H NMR (700 MHz, D_2O, 0.0 – 5.5 ppm) spectrum of **Ac-Lys-SH**.*

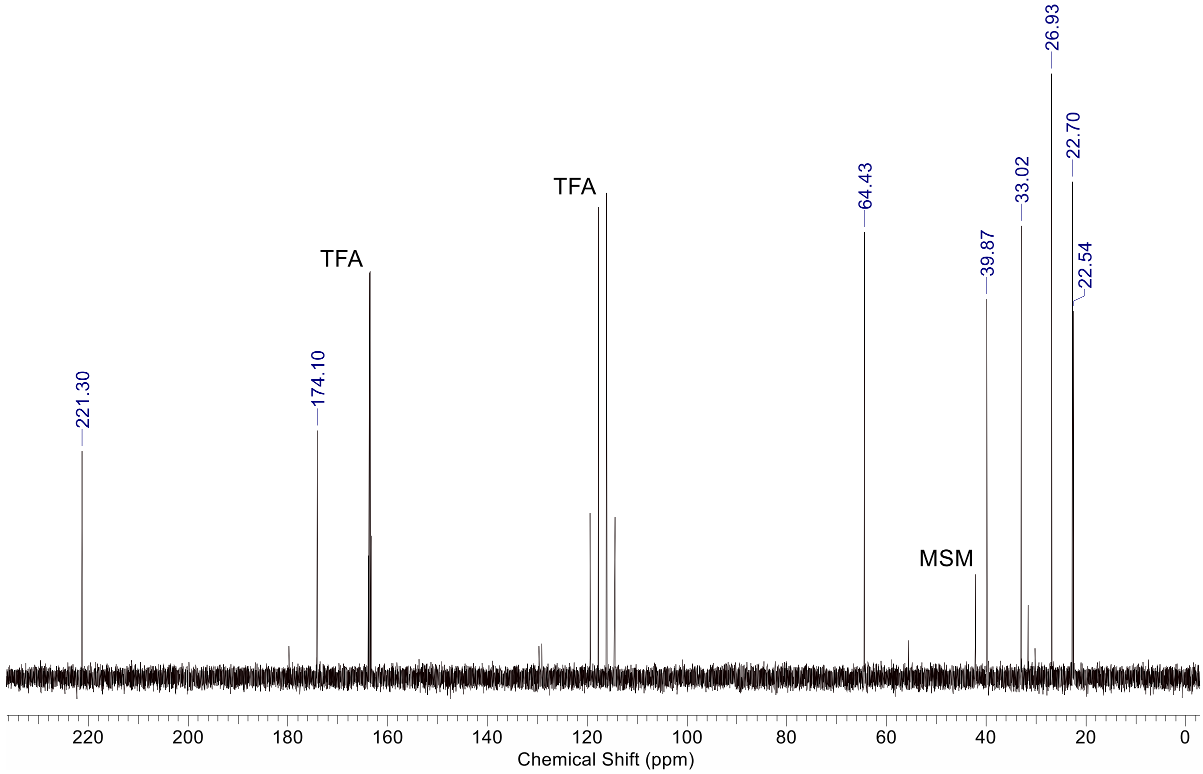

Supplementary Figure 126. ^{13}C *NMR (176 MHz,* D_2O*, 0 – 225 ppm) spectrum of* ***Ac-Lys-SH****.*

Synthesis of Ac-Orn-SH

Prepared according to general procedure **G** using a 10:1 mixture of **Ac-Orn(Boc)-S$^-$Na$^+$/Ac-Orn(Boc)-OH** (22.0 mg, 0.064 mmol in thioacid) to afford **Ac-Orn-SH** (85% by ^{1}H NMR spectroscopy) and **2** (17% by ^{1}H NMR spectroscopy).

Ac-Orn-SH (■) **^{1}H NMR** (700 MHz, D_2O) δ_H 4.43 (dd, *J* = 8.1, 4.7 Hz, 1H, (C2)–H), 3.00 (t, *J* = 7.4 Hz, 2H, (C5)–H_2), 2.02 (s, 3H, $COCH_3$), 1.94-1.98 (m, 1H, (C3)–H), 1.65-1.76 (obs. m, 3H, (C3)–H', (C4)–H_2) . **^{13}C NMR** (176 MHz, D_2O) δ_C 220.6 (C1), 174.1 (**C**OCH_3), 63.9 (C2), 39.7 (C5), 30.5 (C3), 23.9 (C4), 22.6 (CO**C**H_3). **HRMS-ESI** $[M+H]^+$ calc. for $C_7H_{15}N_2O_2S^+$ 191.0849; obs. 191.0849.

2 (♦) **^{1}H NMR** (700 MHz, D_2O, partial assignment) δ_H 4.28 (dd, *J* = 10.6, 6.3 Hz, 1H, (C2)–H), 3.29 (m, 2H, (C5)–H_2), 2.00 (s, 3H, $COCH_3$). **^{13}C NMR** (176 MHz, D_2O, partial assignment) δ_C 174.5 (**C**OCH_3), 173.4 (C1), 50.1 (C2), 27.3 (C3), 20.9 (C4). **HRMS-ESI** $[M+H]^+$ calc. for $C_7H_{13}N_2O_2^+$ 157.0972; obs. 157.0973.

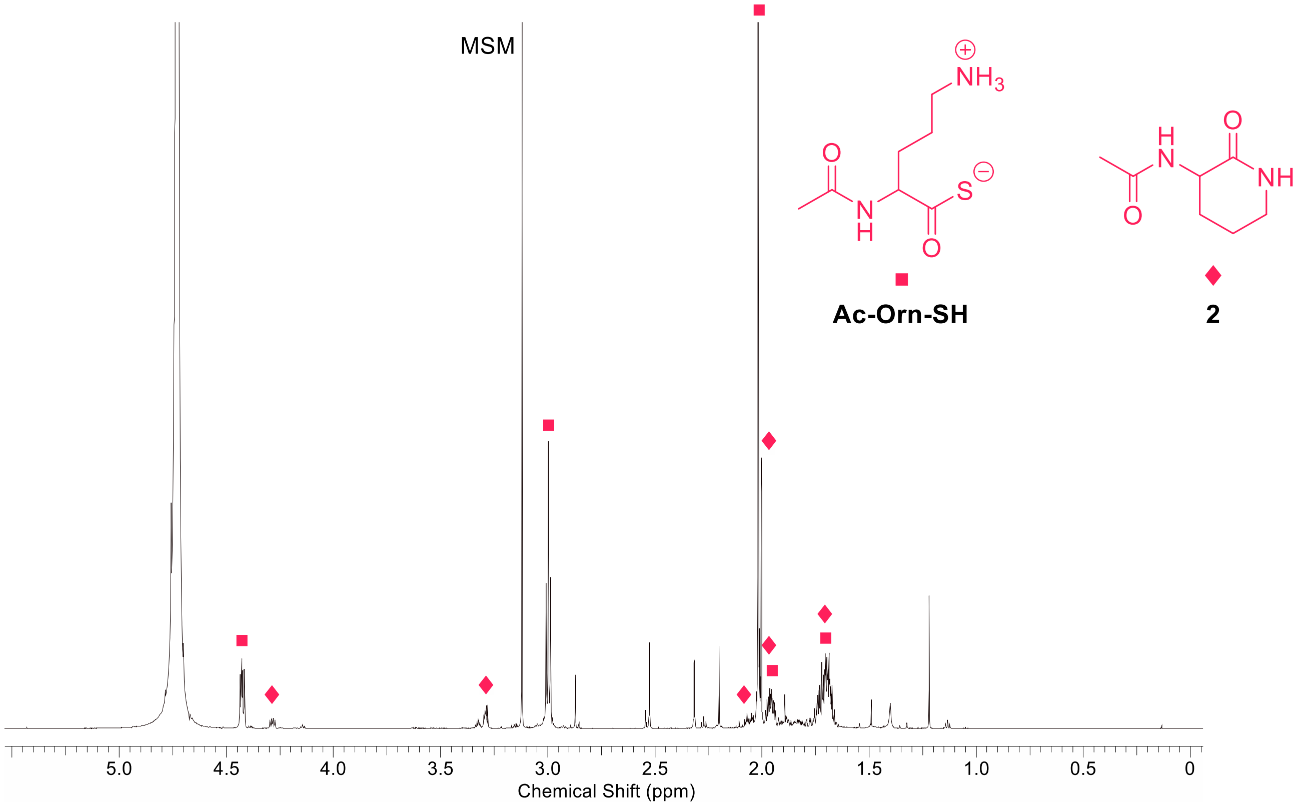

*Supplementary Figure 127. ^{1}H NMR (700 MHz, D_2O, 0.0 – 5.5 ppm) spectrum of **Ac-Orn-SH** and **2**.*

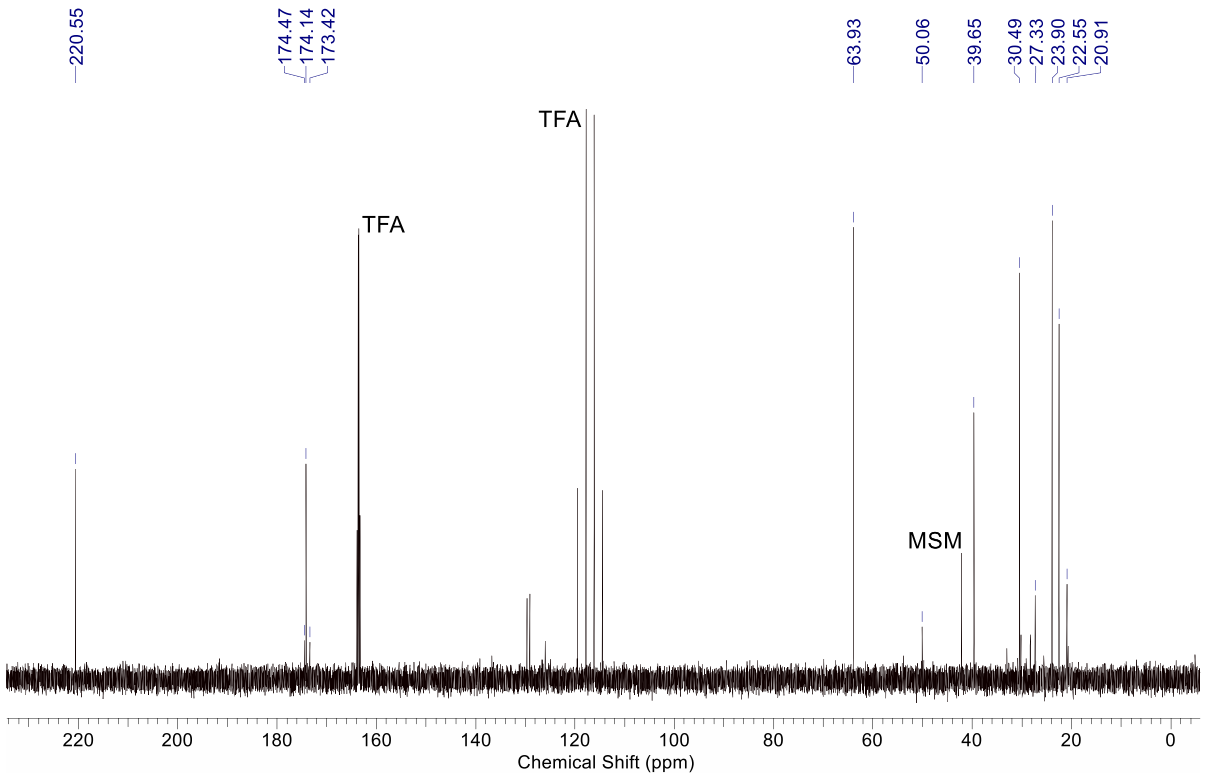

*Supplementary Figure 128. ^{13}C NMR (176 MHz, D_2O, 0 – 230 ppm) spectrum of **Ac-Orn-SH** and **2**.*

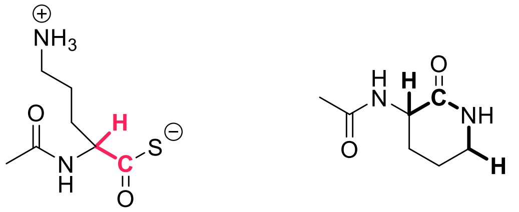

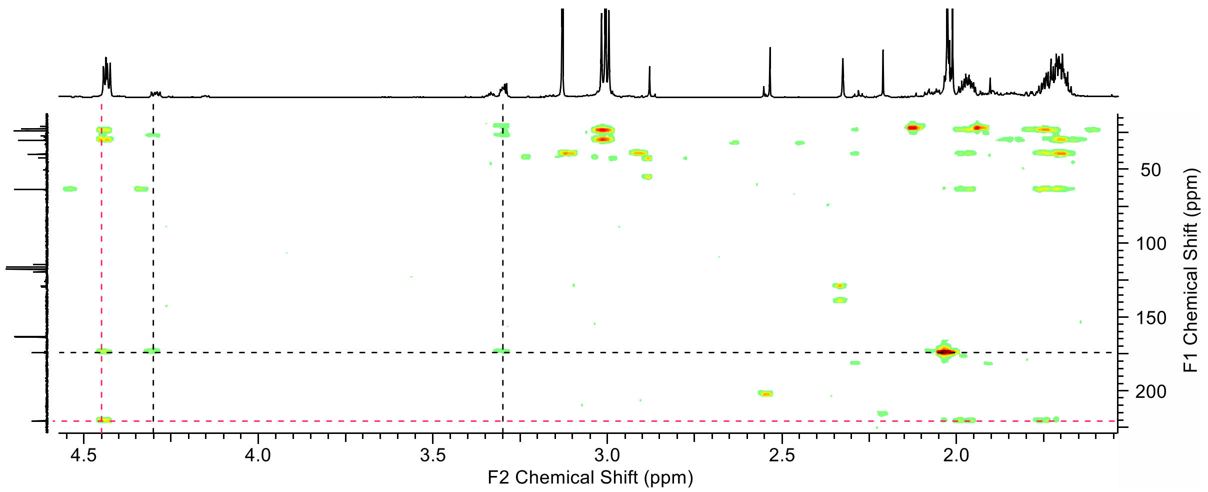

Supplementary Figure 129. ^{1}H-^{13}C HMBC (^{1}H-700 MHz [1.5-4.5 ppm], ^{13}C-176 MHz [0-230 ppm], D_2O) spectrum showing the $^{2}J_{CH}$ coupling of Orn-(C2)–H at 4.43 ppm of **Ac-Orn-SH** to C=O resonance at 220.6 ppm, and the $^{2}J_{CH}$ and $^{3}J_{CH}$ couplings of Orn-(C2)–H at 4.28 ppm and Orn-(C5)–H_2 at 3.29 ppm of **2** to C=O resonance at 173.4 ppm, which are diagnostic for thioacid and lactam formation, respectively.

Synthesis of Ac-Dab-SH

Prepared according to general procedure **G** using a 8:1 mixture of **Ac-Dab(Boc)-S⁻Na⁺**/lactam **18** (22.0 mg, 0.06 mmol in thioacid) to form **Ac-Dab-SH** (75% by ^{1}H NMR), **3** (20% by ^{1}H NMR), **Ac-Dab-OH** (< 5% by ^{1}H NMR).

Ac-Dab-SH (■): **^{1}H NMR** (700 MHz, D_2O) δ_H 4.50 (dd, *J* = 8.9, 4.9 Hz 1H, (C2)–H), 3.00-3.07 (m, 2H, (C4)–H_2), 2.25-2.30 (m, 1H, (C3)–H), 2.03 (s, 3H, $COCH_3$), 1.95-2.00 (m, 1H, (C3)–H'). **^{13}C NMR** (176 MHz, D_2O) δ_C 218.9 (C1), 174.3 (**C**OCH_3), 61.8 (C2), 37.2 (C4), 31.5 (C3), 22.5 (CO**C**H_3). **HRMS-ESI** $[M+H]^+$ calc. for $C_6H_{13}N_2O_2S^+$ 177.0692; obs. 177.0692.

3 (♦): **^{1}H NMR** (700 MHz, D_2O, partial assignment) δ_H 3.42-3.46 (m, 2H, (C4)–H_2), 2.45-2.50 (m, 1H, (C3)–H), 2.01 (s, 3H, $COCH_3$). **^{13}C NMR** (176 MHz, D_2O, partial assignment) δ_C 174.9 (C1/**C**OCH_3), 51.4 (C2), 39.8 (C4), 27.8 (C3).

Ac-Dab-OH (●): **^{1}H NMR** (700 MHz, D_2O, partial assignment) δ_H 4.22 (dd, *J* = 8.8, 5.1 Hz, 1H, (C2)–H).

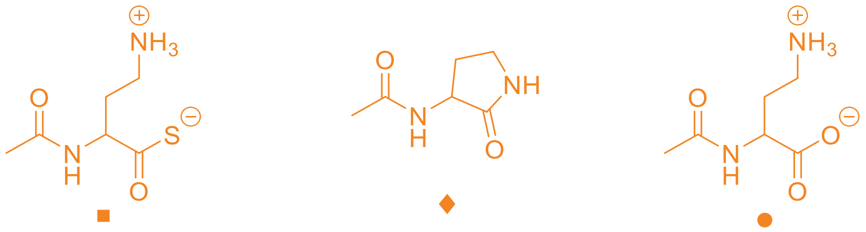

Ac-Dab-SH **3** **Ac-Dab-OH**

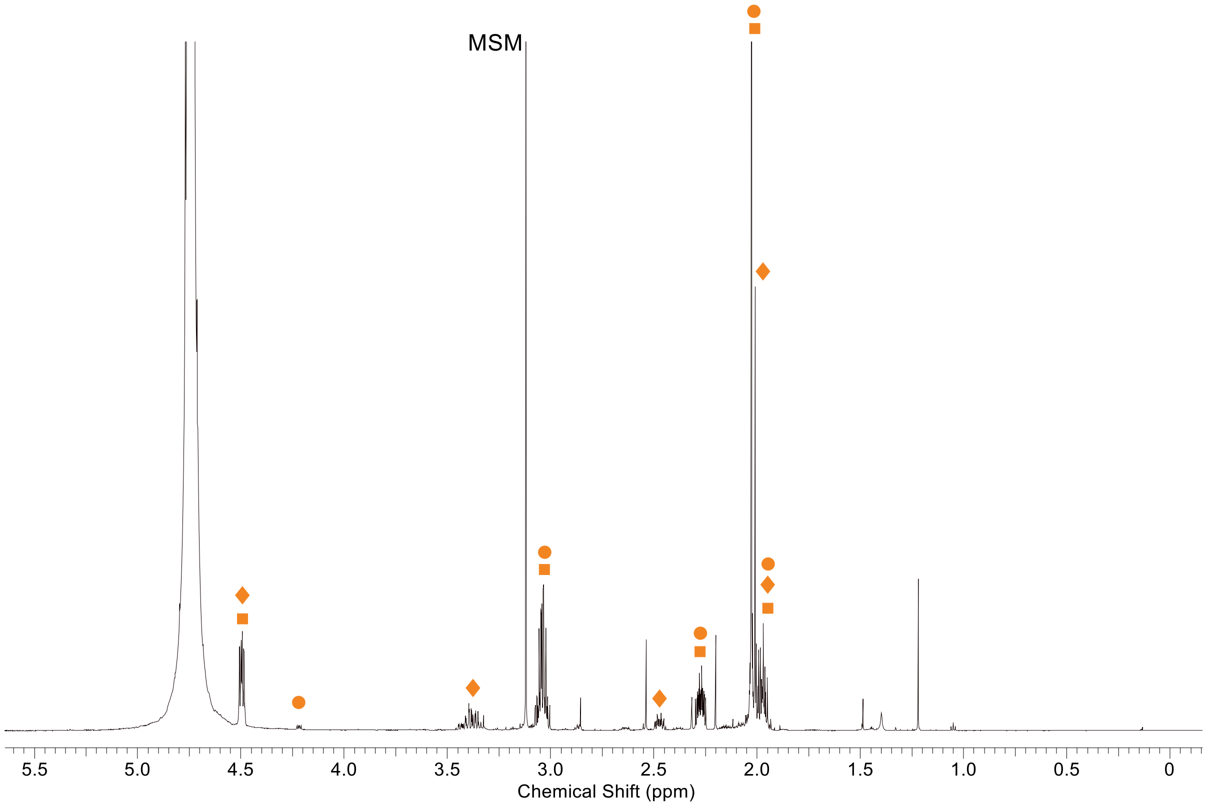

*Supplementary Figure 130. ^{1}H NMR (700 MHz, D_2O, 0.0 – 5.5 ppm) spectrum showing the formation of **Ac-Dab-SH**, **3** and **Ac-Dab-OH** from a mixture of **Ac-Dab(Boc)-S⁻Na⁺** and **18**.*

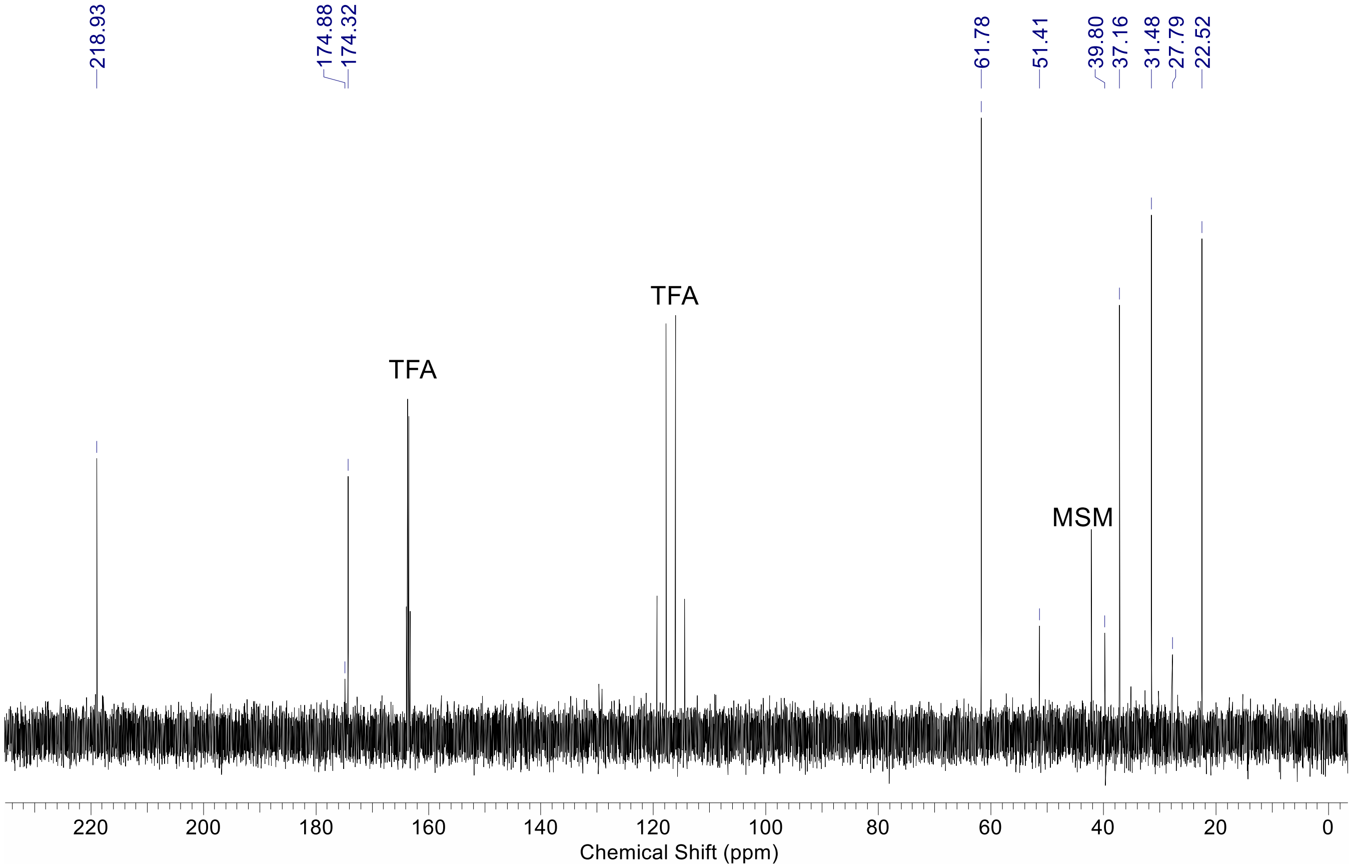

*Supplementary Figure 131. ^{13}C NMR (176 MHz, D_2O, 0 – 230 ppm) spectrum of **Ac-Dab-SH** and lactam **3**.*

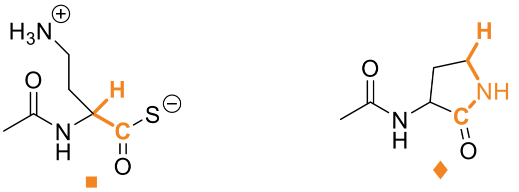

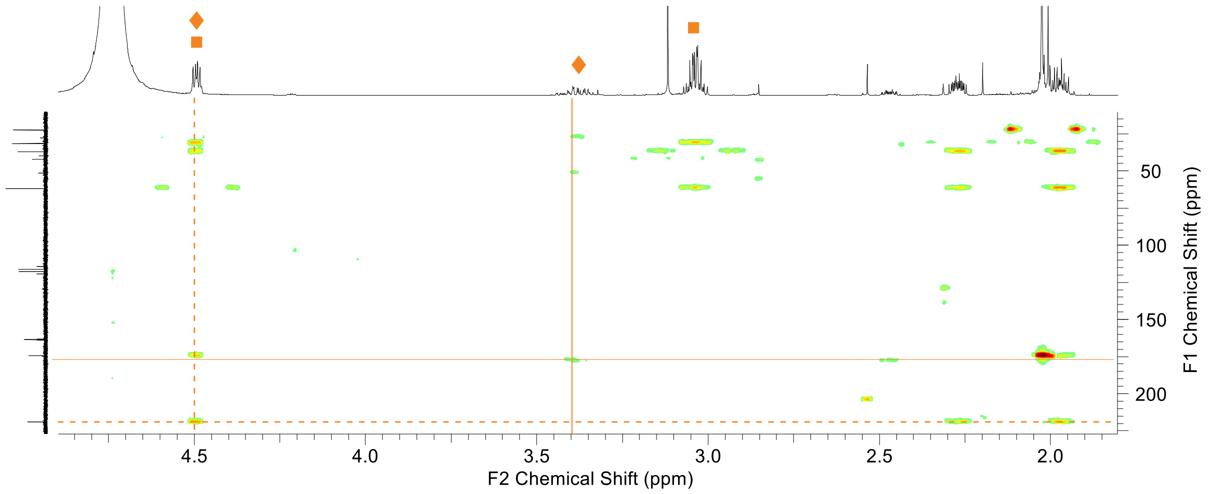

*Supplementary Figure 132. ^{1}H-^{13}C HMBC (^{1}H-700 MHz [1.8 – 4.9 ppm], ^{13}C-176 MHz [0 – 230 ppm], D_2O) spectrum showing the $^{2}J_{CH}$ coupling of Dab-(C2)–H at 4.50 ppm of **Ac-Dab-SH** to C=O resonance at 218.9 ppm, and the $^{3}J_{CH}$ coupling of Dab-(C4)–H at 3.40 ppm of **3** to C=O resonance at 174.9 ppm, which are diagnostic for thioacid and lactam formation, respectively.*

Synthesis of Ac-Dpr-SH

Prepared according to general procedure **G** using 70% **Ac-Dpr(Boc)-S$^-$Na$^+$** (12.1 mg, 0.03 mmol in thioacid) to form **Ac-Dpr-SH** (> 95% yield from **Ac-Dpr(Boc)-S$^-$Na** by ^{1}H NMR).

Ac-Dpr-SH (■) **^{1}H NMR** (700 MHz, D_2O) δ_H 4.69 (obs. m, 1H, (C2)–H), 3.53 (dd, *J* = 13.1, 5.2, Hz, 1H, (C3)–H), 3.20 (dd, *J* = 13.1, 8.0 Hz, 1H, (C3)–H'), 2.06 (s, 3H, $COCH_3$). **^{13}C NMR** (176 MHz, D_2O) δ_C 215.0 (C1), 174.8 (**C**OCH_3), 61.2 (C2), 42.5 (C3), 22.7 (CO**C**H_3). **HRMS-ESI** [M+H]$^+$ calc. for $C_5H_{11}N_2O_2S^+$ 163.0536; obs. 163.0536.

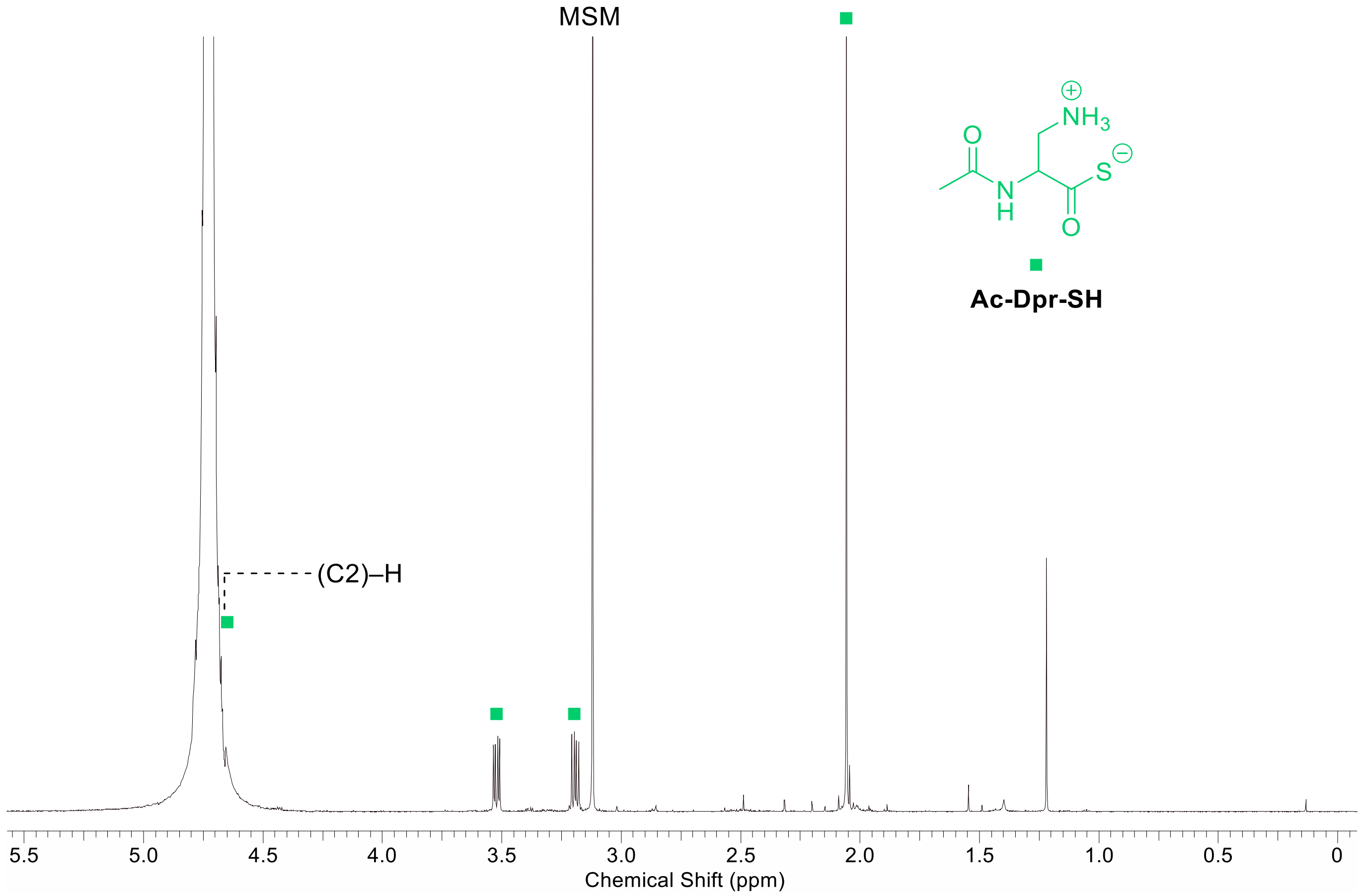

*Supplementary Figure 133. ^{1}H NMR (700 MHz, D_2O, 0.0 – 5.5 ppm) of **Ac-Dpr-SH**. The (C2)–H of **Ac-Dpr-SH** is partially obscured by HOD.*

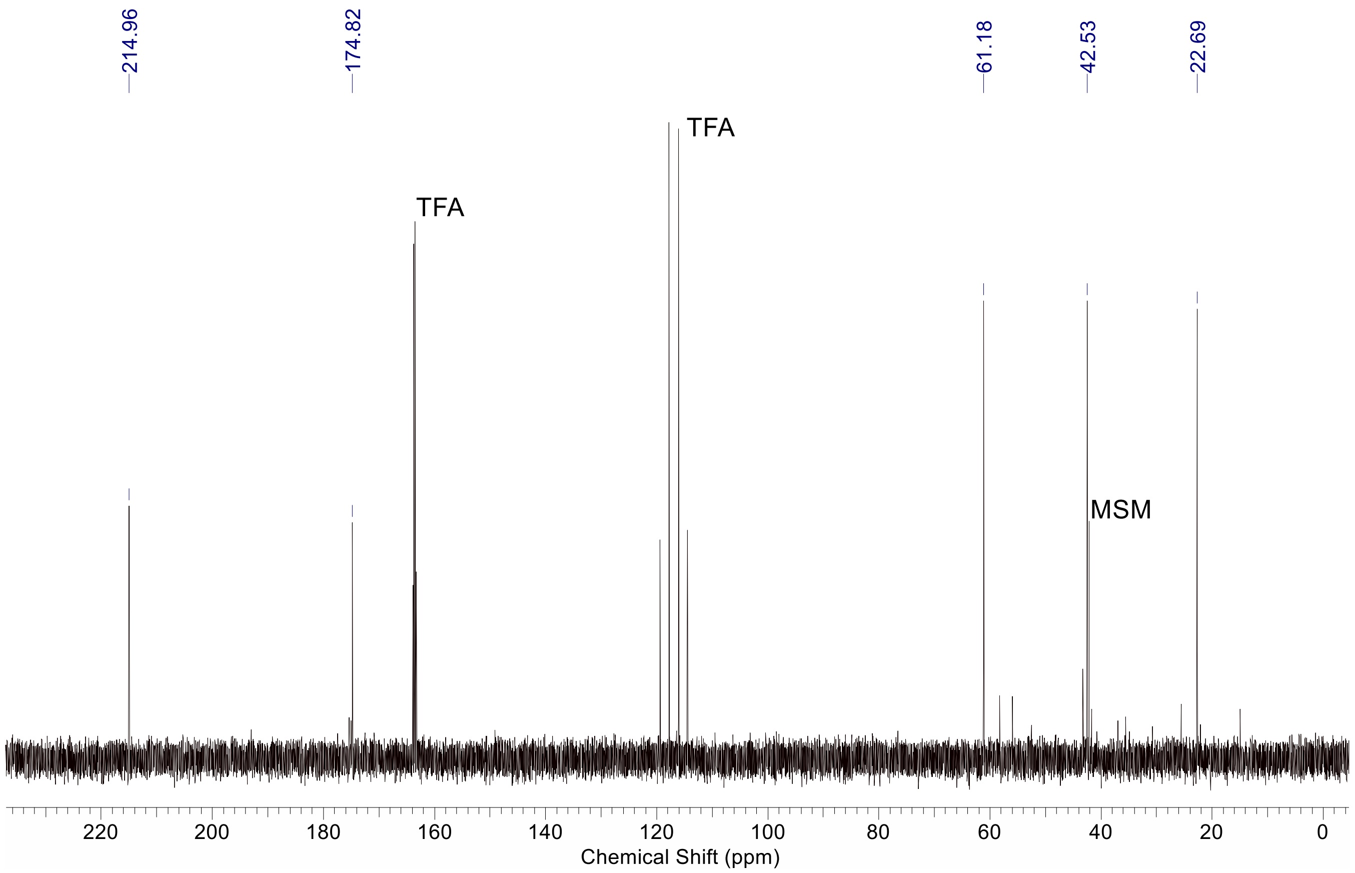

Supplementary Figure 134. *^{13}C NMR (176 MHz, D_2O, 0 – 230 ppm) spectrum of **Ac-Dpr-SH.***

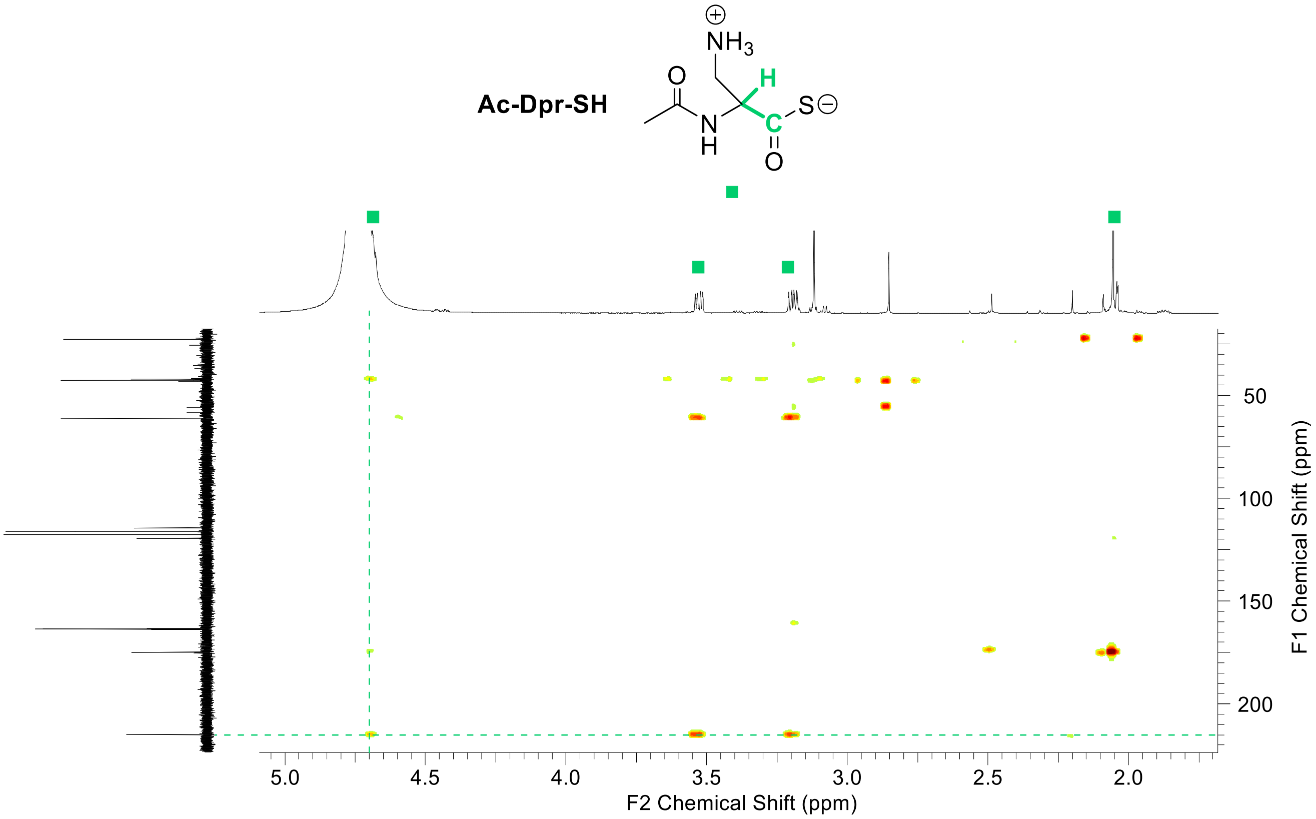

*Supplementary Figure 135. ^{1}H-^{13}C HMBC (^{1}H-700 MHz [1.6-5.1 ppm], ^{13}C-176 MHz [25-220 ppm], D_2O) spectrum showing the submerged $^{2}J_{CH}$ coupling of Dpr-(C2)–H at 4.69 ppm of **Ac-Dpr-SH** to C=O resonance at 215.0 ppm, diagnostic for thioacid formation.*

Synthesis of Ac-Lys-Gly-SH

Prepared according to general procedure **G** using 80% **Ac-Lys(Boc)-Gly-S^-Na^+** (11.1 mg, 0.02 mmol in thioacid) to form **Ac-Lys-Gly-SH** (90% yield from **Ac-Lys(Boc)-Gly-S^-Na^+** by ^{1}H NMR). **^{1}H NMR** (700 MHz, D_2O) δ_H 4.29 (dd, *J* = 8.5, 5.6 Hz, 1H, Lys-(C2)–H), 4.11 (AB, *J* = 17.7 Hz, 1H, Gly-(C2)–H), 4.07 (AB, *J* = 17.7 Hz, 1H, Gly-(C2)–H'), 2.98 (app td, *J* = 7.6, 1.3 Hz, 2H, Lys-(C6)–H_2), 2.02 (s, 3H, $COCH_3$), 1.83-1.88 (m, 1H, Lys-(C3)–H), 1.71-1.76 (m, 1H, Lys-(C3)–H'), 1.64-1.70 (m, 2H, Lys-(C5)–H_2), 1.40-1.52 (m, 2H, Lys-(C4)–H_2). **^{13}C NMR** (176 MHz, D_2O) δ_C 216.6 (Lys-C1), 175.4 (Gly-C1/**C**OCH_3), 174.6 (Gly-C1/**C**OCH_3), 54.6 (Lys-C2/Gly-C2), 54.3 (Lys-C2/Gly-C2), 40.3 (Lys-C6), 31.5 (Lys-C3), 27.3 (Lys-C5), 23.0 (Lys-C4), 22.8 (CO**C**H_3). **HRMS-ESI** $[M+H]^+$ calc. for $C_{10}H_{19}N_3O_3S^+$ 262.1220; obs. 262.1220.

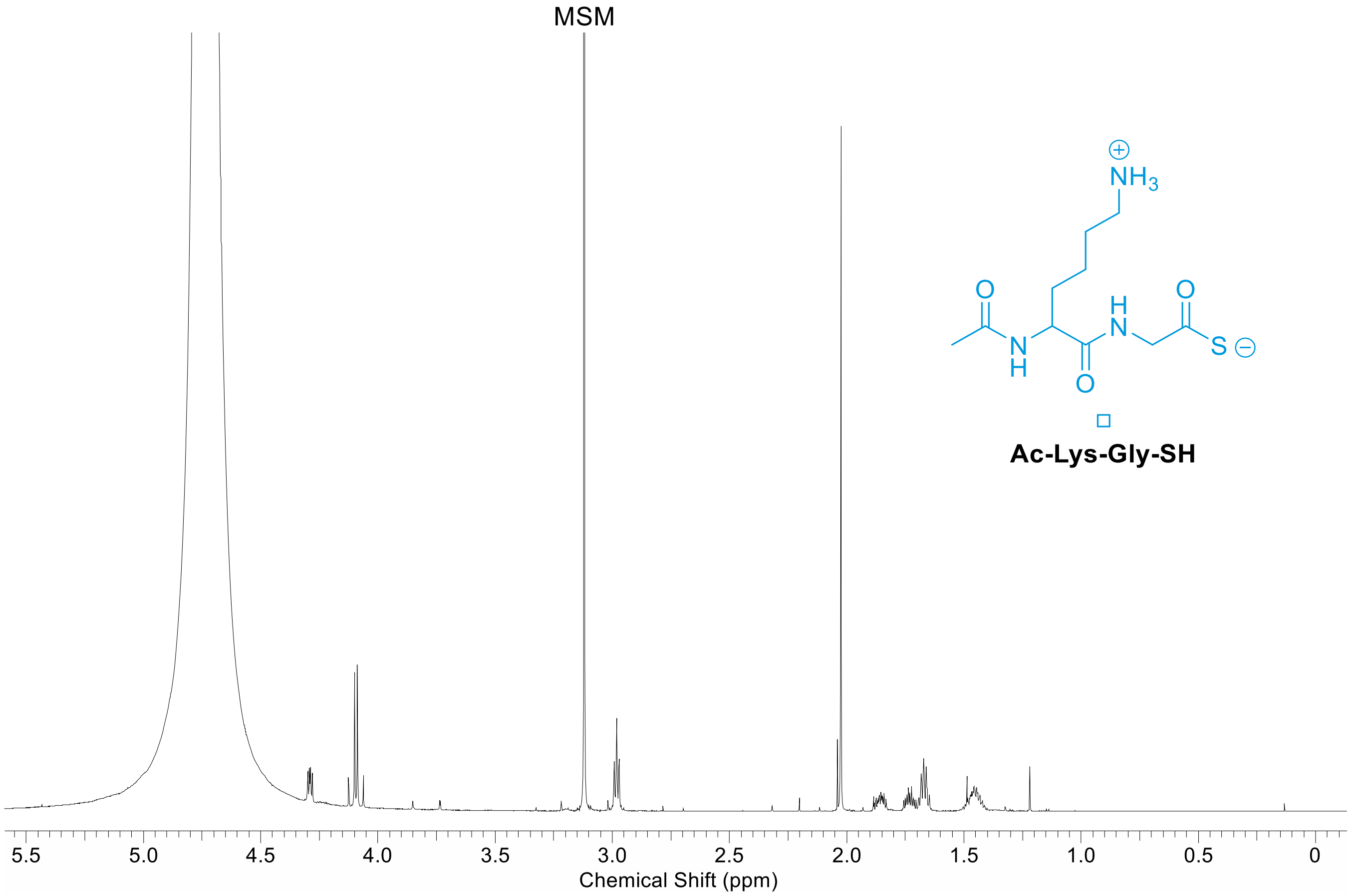

*Supplementary Figure 136. ^{1}H NMR (700 MHz, D_2O, 0.0 – 5.5 ppm) spectrum of **Ac-Lys-Gly-SH**.*

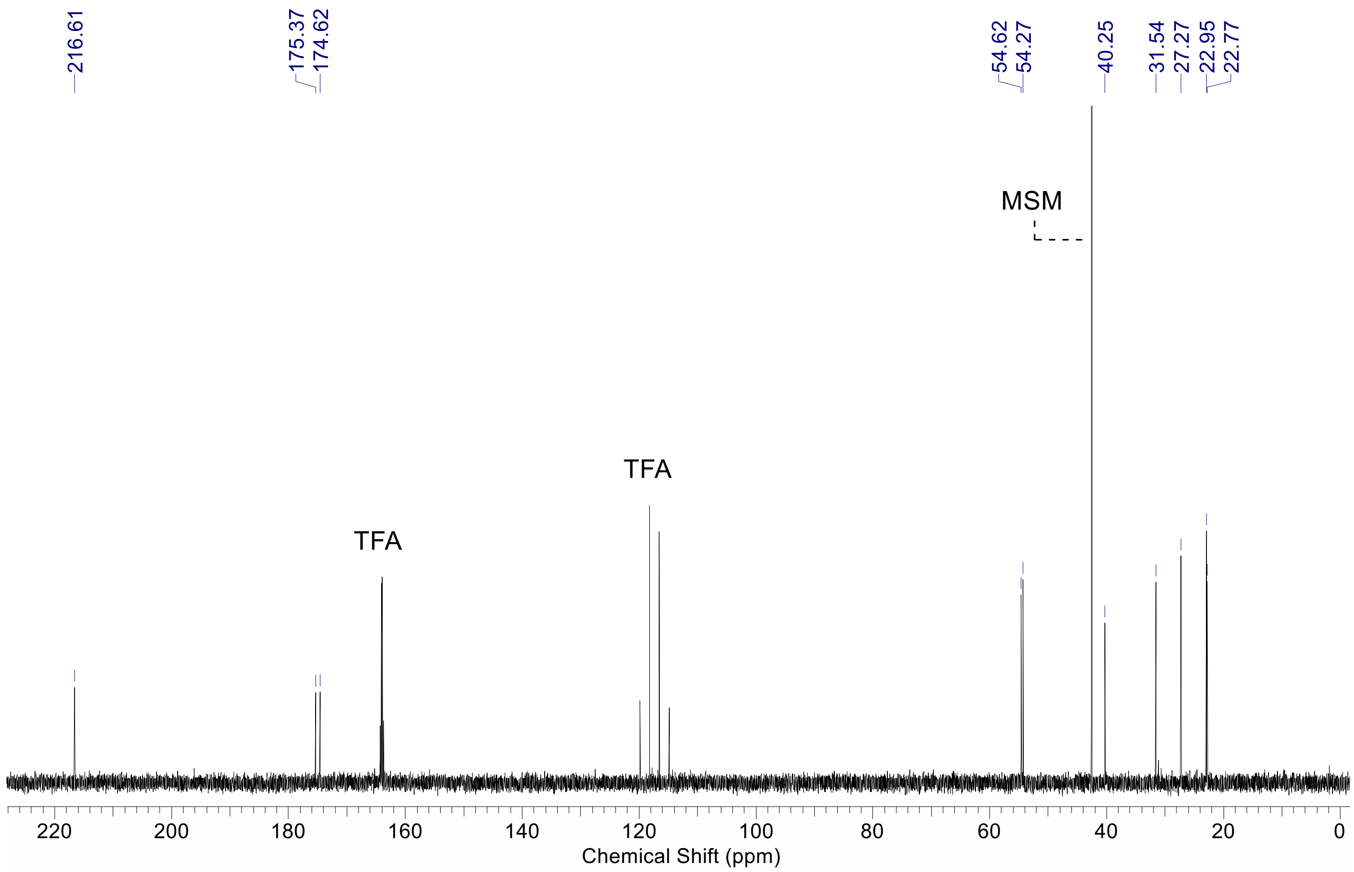

Supplementary Figure 137. ^{13}C NMR (176 MHz, D_2O, 0 – 225 ppm) spectrum of ***Ac-Lys-Gly-SH****.*

Synthesis of Ac-Dpr-Gly-SH

Prepared according to general procedure **G** using 90% **Ac-Dpr(Boc)-Gly-S$^-$Na$^+$** (14.4 mg, 0.04 mmol in thioacid) to form **Ac-Dpr-Gly-SH** (>95% yield from **Ac-Dpr(Boc)-Gly-S$^-$Na$^+$** by ^{1}H NMR). **^{1}H NMR** (700 MHz, D_2O, partial assignment) δ_H 4.14 (AB, *J* = 17.7 Hz, 1H, Gly-(C2)–H), 4.08 (AB, *J* = 17.7 Hz, 1H, Gly-(C2)–H'), 3.46 (ABX, *J* = 13.3, 5.5 Hz, 1H, Dpr-(C3)–H), 3.27 (ABX, *J* = 13.3, 7.7 Hz, 1H, Dpr-(C3)–H'), 2.07 (s, 3H, $COCH_3$). **^{13}C NMR** (176 MHz, D_2O) δ_C 216.9 (Gly-C1), 175.7 (**C**OCH_3), 170.9 (Dpr-C1), 54.3 (Gly-C2), 51.9 (Dpr-C2), 41.4 (Dpr-C3), 23.0 (CO**C**H_3). **HRMS-ESI** [M+H]$^+$ calc. for $C_7H_{14}N_3O_3S^+$: 220.0750; obs. 220.0749.

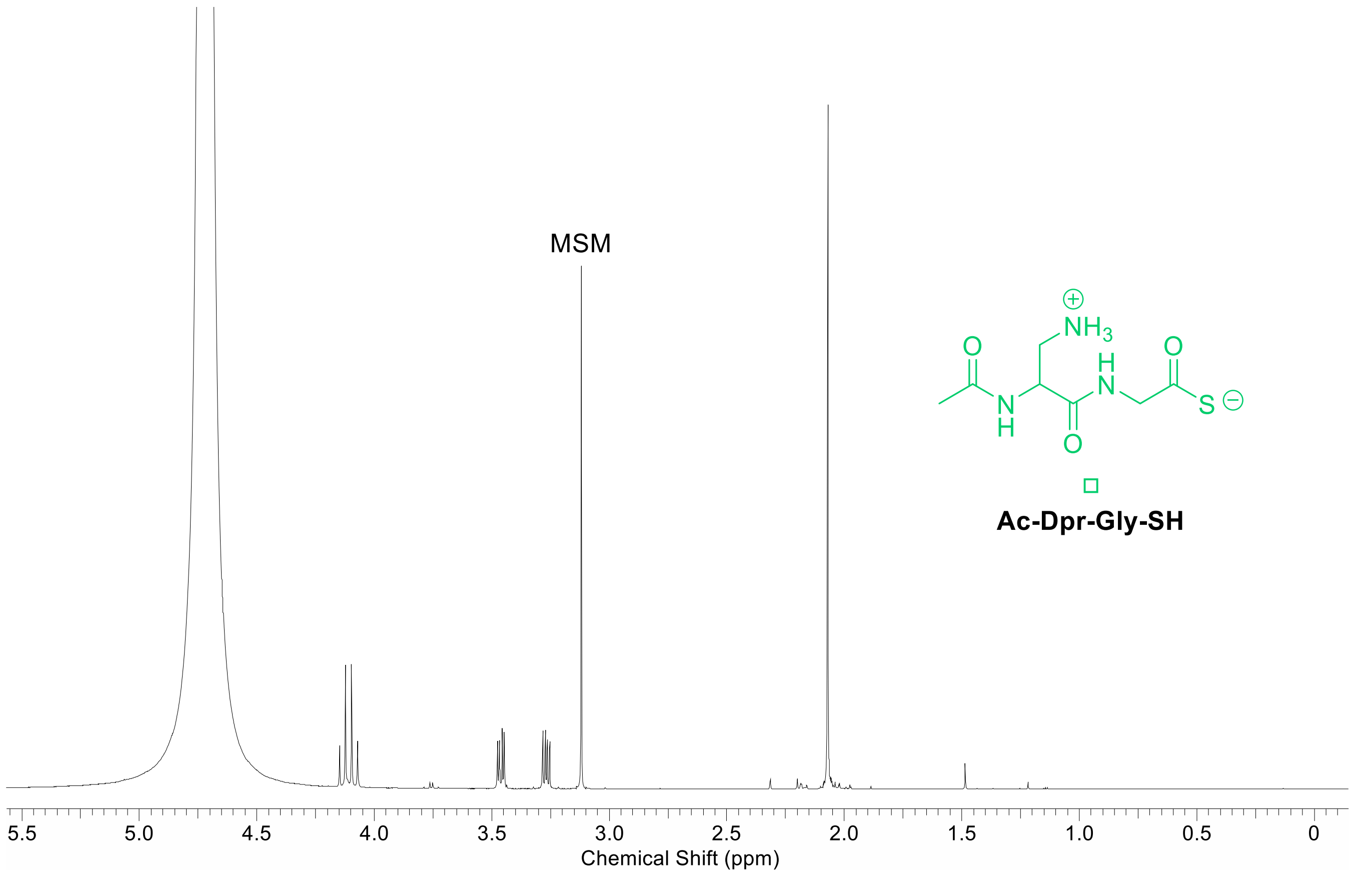

*Supplementary Figure 138. ^{1}H NMR (700 MHz, D_2O, 0.0 – 5.5 ppm) spectrum of **Ac-Dpr-Gly-SH**. The (C2)–H of **Ac-Dpr-Gly-SH** is obscured by HOD.*

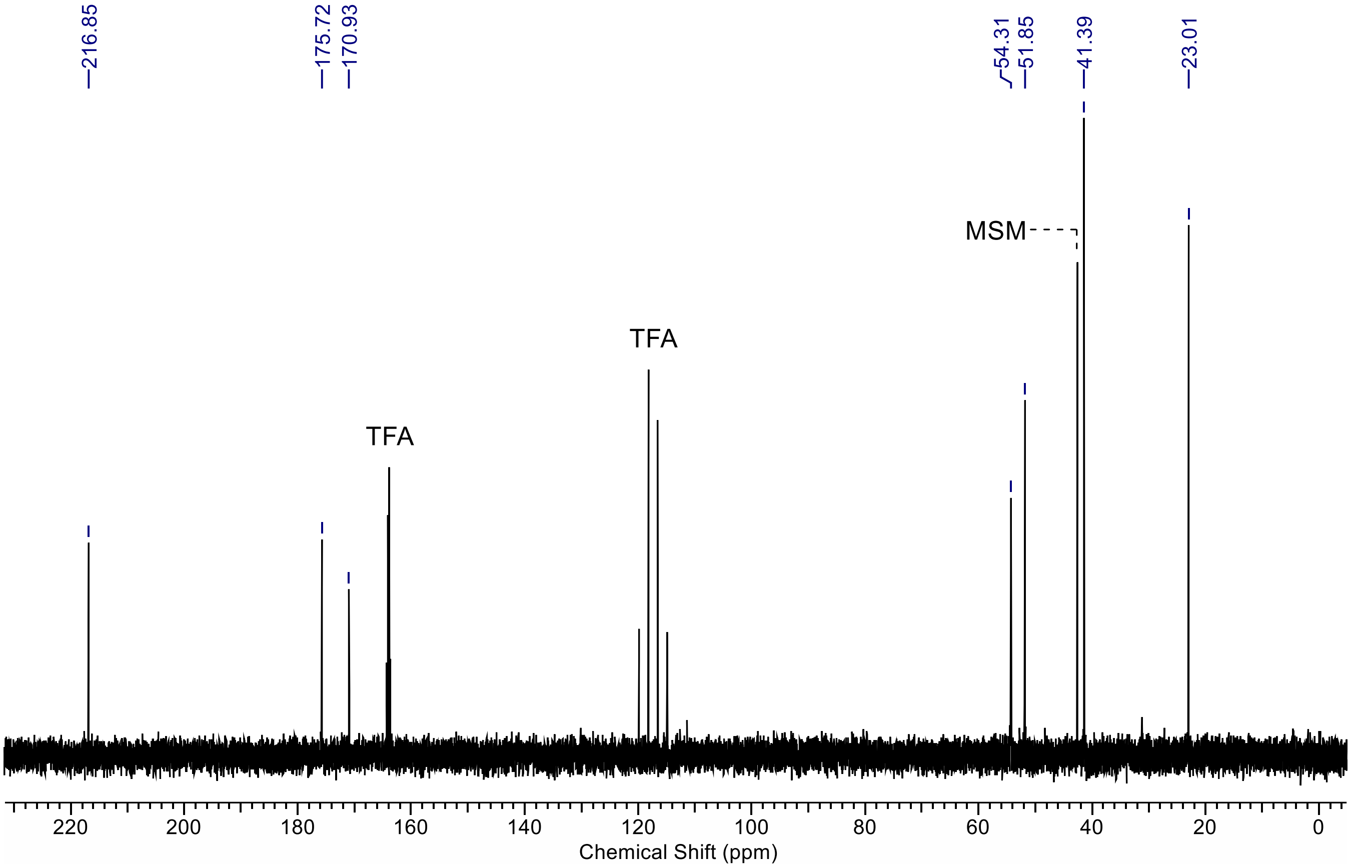

*Supplementary Figure 139. ^{13}C NMR (176 MHz, D_2O, 0 – 225 ppm) spectrum of **Ac-Dpr-Gly-SH**.*

Miscellaneous preparative syntheses

δ–lactam 2[10]

Prepared according the adapted procedure of Henkel *et al.*[10] To DL-ornithine monohydrochloride (200 mg, 1.19 mmol) was added Ac_2O/pyridine (20.0 mL, 10:1). The reaction mixture was stirred at room temperature for 16 h and the volatiles then removed *in vacuo*. Purification by column chromatography (98:2 CH_2Cl_2/MeOH) afforded **2** (100.0 mg, 0.64 mmol, 54%) as a white powder. **^{1}H NMR** (600 MHz, D_2O) δ_H 4.25 (dd, J = 10.6, 5.6 Hz, 1H, (C2)–H), 3.27-3.25 (m, 2H, (C5)–H_2), 2.06-2.01 (m, 1H, (C3)–H), 1.97 (s, 3H, $COCH_3$), 1.90-1.85 (m, 1H, (C3)–H'), 1.83-1.71 (m, 2H, (C4)–H_2). **^{13}C NMR** (176 MHz, D_2O) δ_C 174.4 (**C**OCH_3), 173.4 (C1), 50.0 (C2), 42.1 (C5), 27.3 (C3), 22.4 (CO**C**H_3), 20.9 (C4). **HRMS-ESI** $[M+Na]^+$ calc. for $C_7H_{12}N_2O_2Na^+$ 179.0791; obs. 179.0791. **IR** (solid, cm^{-1}): 3299, 3193, 3062, 2947, 1654, 1627, 1534. Characterisation data were in accordance with literature values.[10]

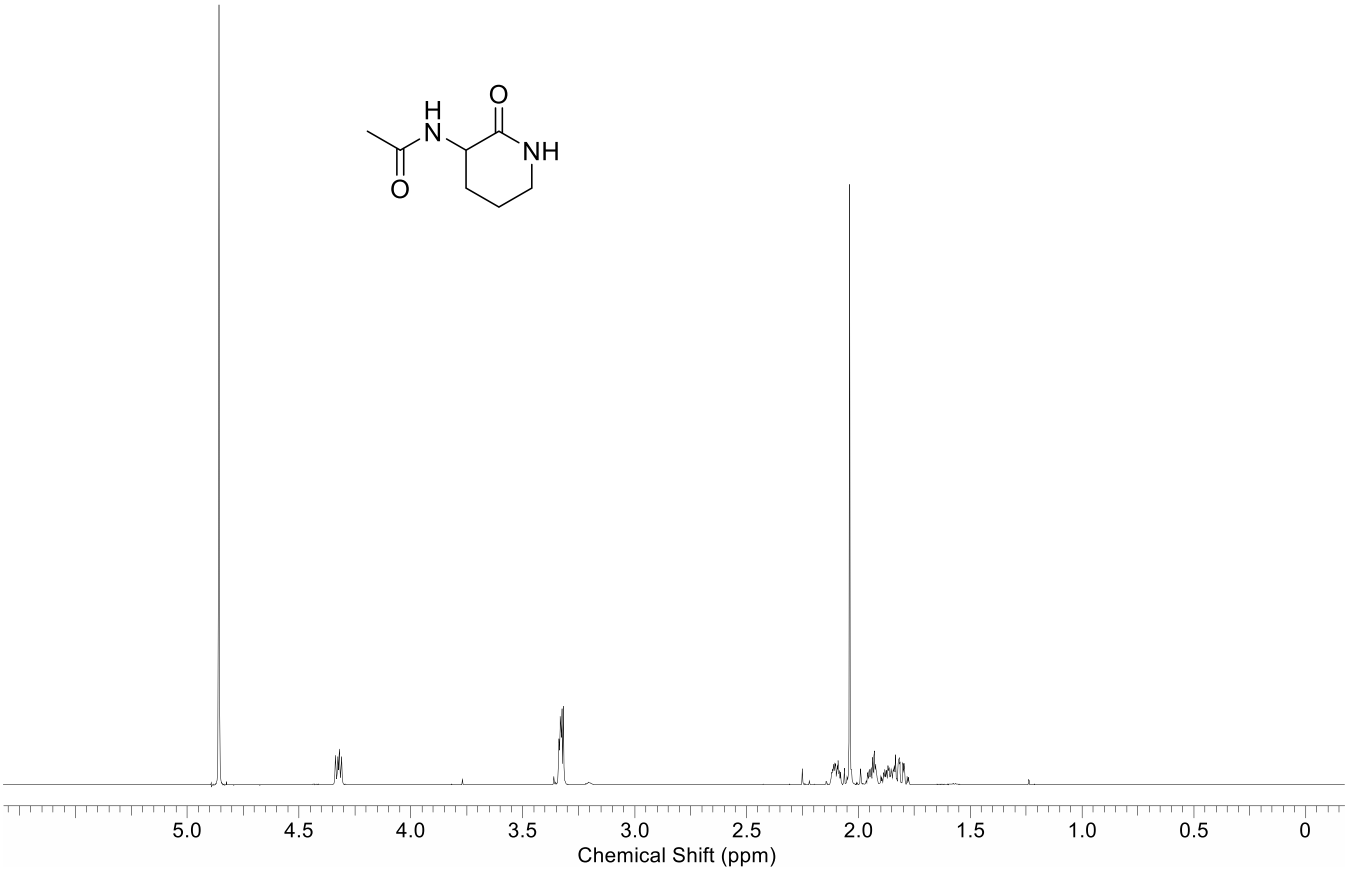

*Supplementary Figure 140. ^{1}H NMR (600 MHz, D_2O, 0.0 – 8.0 ppm) spectrum of **2**.*

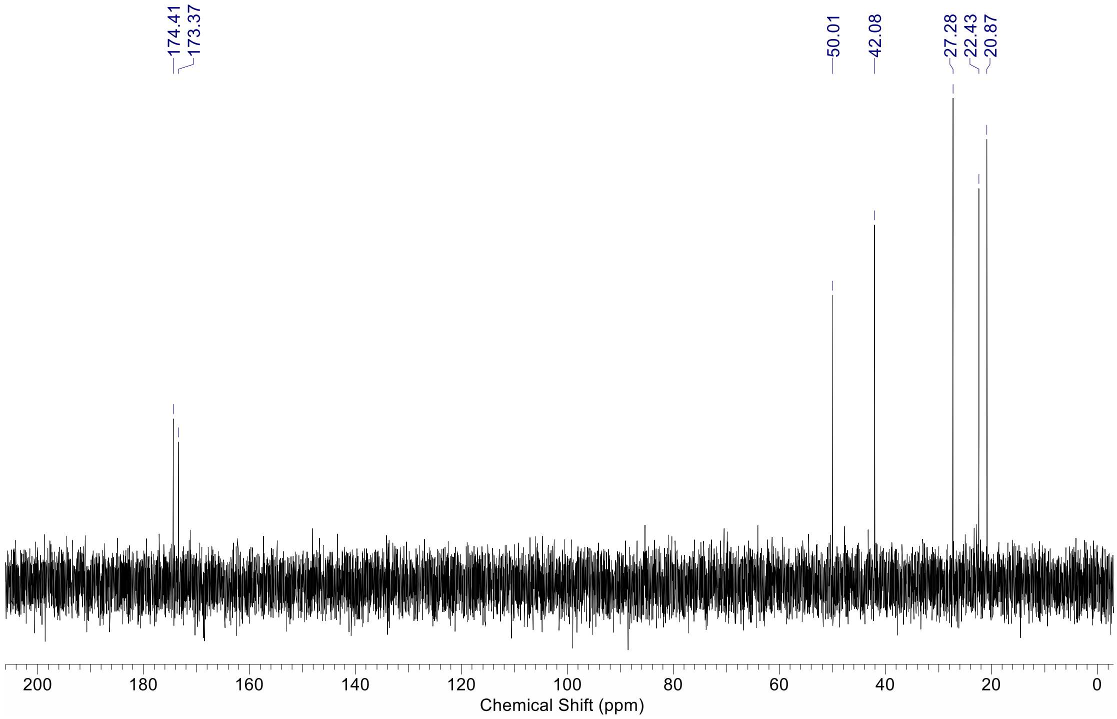

*Supplementary Figure 141. ^{13}C NMR (176 MHz, D_2O, 0 – 200 ppm) spectrum of **2**.*

γ-lactam 3[11]

To L-2,4-Diaminobutyric acid dihydrochloride (58.2 mg, 0.30 mmol) was added Ac_2O/pyridine (5.00 mL, 10:1). The reaction mixture was stirred at room temperature for 18 h. Volatiles were removed *in vacuo.* Purification by column chromatography (CH_2Cl_2 to CH_2Cl_2/MeOH; 9:1) afforded **3** (7.04 mg, 0.05 mmol, 17%) as an off-white powder. **^{1}H NMR** (400 MHz, D_2O) δ_H 4.52 (t, *J* = 9.8 Hz, 1H, (C2)–H), 3.35-3.45 (m, 2H, (C4)–H_2), 2.46-2.54 (m, 1H, (C3)–H), 1.99-2.12 (m, 1H, (C3)–H'), 2.03 (s, 3H, $COCH_3$). **^{13}C NMR** (100 MHz, D_2O) δ_C 177.9 (C1), 174.8 (**C**OCH_3), 51.4 (C2), 39.8 (C4), 27.8 (C3), 22.4 (CO**C**H_3). **HRMS-ESI** $[M+H]^+$ calc. for $C_6H_{11}N_2O_2^+$ 143.0815; obs. 143.0816. **IR** (solid, cm^{-1}): 3271, 2982, 1719, 1695, 1632, 1543.

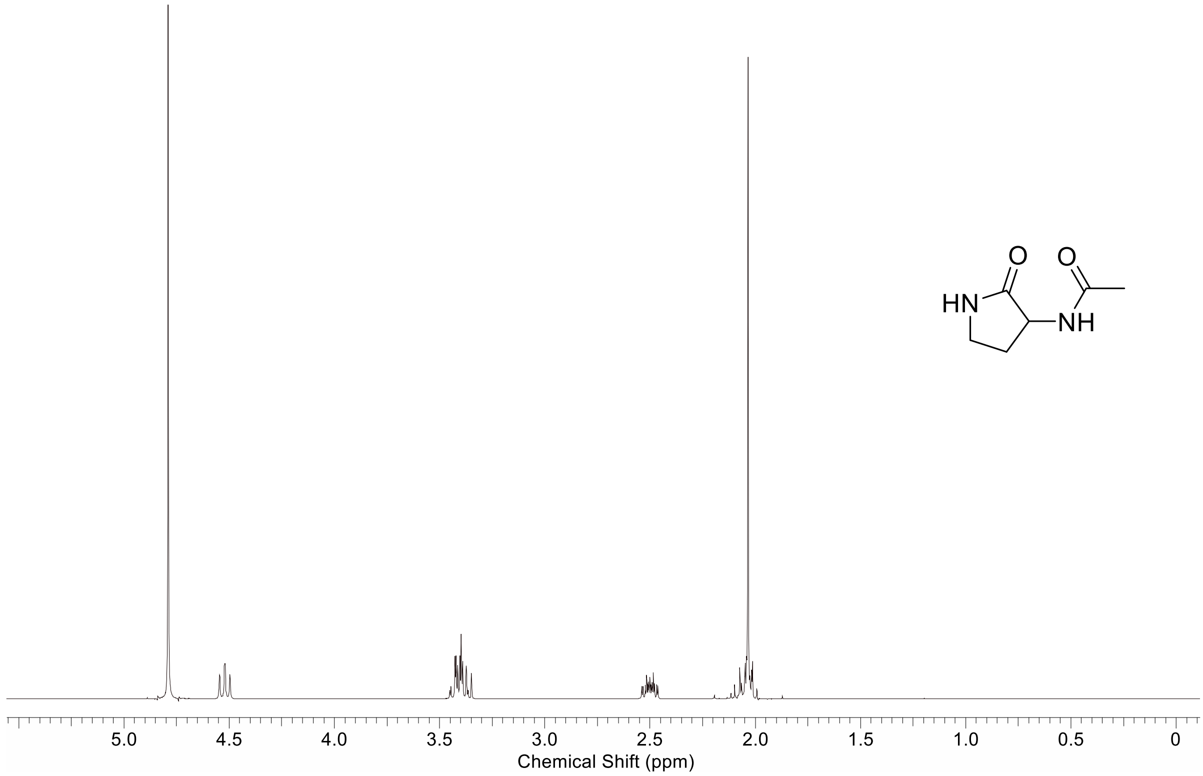

*Supplementary Figure 142. ^{1}H NMR (400 MHz, D_2O, 0.0 – 5.5 ppm) spectrum of **3**.*

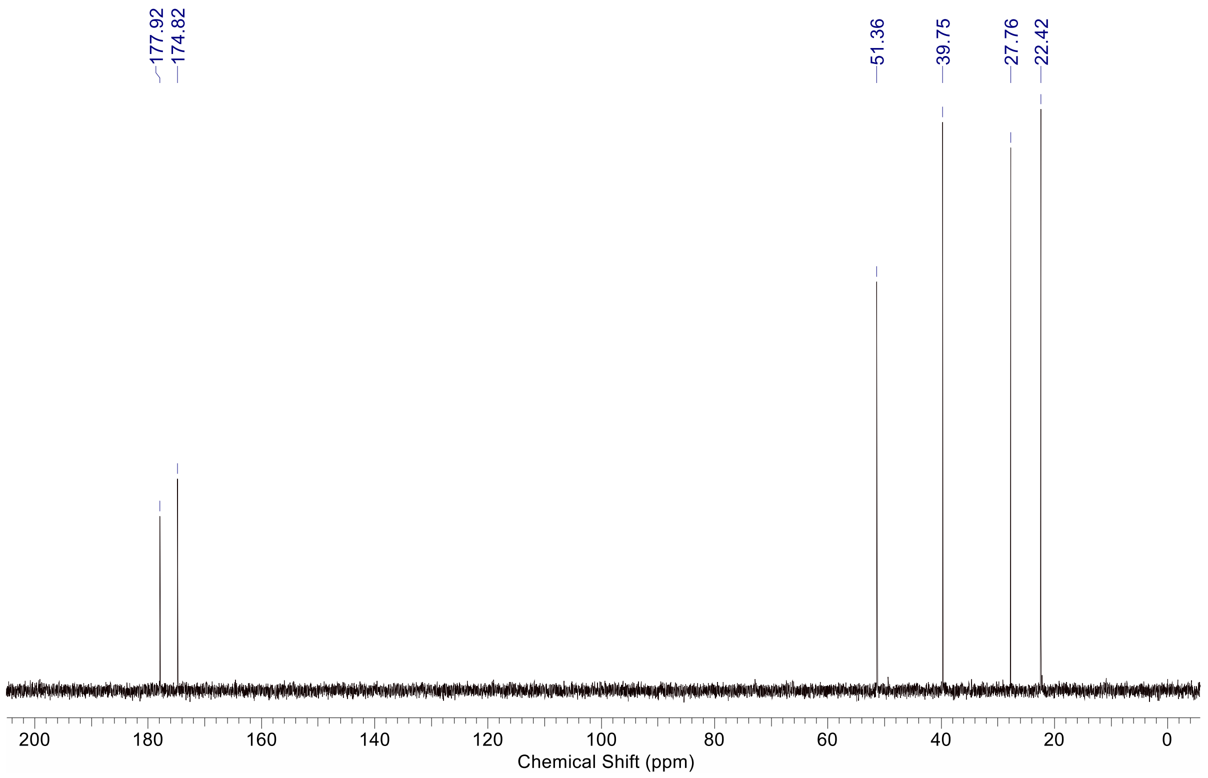

*Supplementary Figure 143. ^{13}C NMR (100 MHz, D_2O, 0 – 200 ppm) spectrum of **3**.*

β–lactam 4[12]

DL-Dpr-OH·HCl (188 mg, 0.82 mmol) was dissolved in 2 M NaOH (0.41 mL, 0.82 mmol). The sample was lyophilised. To the neutralised DL-Dpr-OH (0.82 mmol) was added MeCN/H_2O (95:5, 17.7 mL), followed by PPh_3 (258 mg, 0.98 mmol) and $(PyS)_2$ (216 mg, 0.98 mmol). The reaction mixture was heated at 60 °C for 5.5 h. Volatiles were removed *in vacuo*. Purification by column chromatography (EtOAc to EtOAc/MeOH; 8:2) afforded 3-amino-2-oxoazetidine[12] (5.5 mg, 0.06 mmol, 8%). To 3-amino-2-oxoazetidine (4.20 mg, 0.05 mmol) in H_2O (0.40 mL) at pH 9 was added Ac_2O (8.60 µL, 0.09 mmol) portion wise whilst the solution was continually readjusted to pH 9 with 4 M NaOH. The solvent was removed *in vacuo*. Purification of the crude residue by column chromatography (EtOAc to EtOAc/MeOH; 9:1) afforded **4** as a white solid (1.40 mg, 0.01 mmol, 20%). **^{1}H NMR** (700 MHz, CD_3OD) δ_H 4.93 (dd, *J* = 5.4, 2.5 Hz, 1H, (C2)–H), 3.56 (app t, *J* = 5.4 Hz, 1H, (C3)–H), 3.27 (dd, *J* = 5.5, 2.5 Hz, 1H, (C3)–H'), 1.98 (s, 3H, $COCH_3$). **^{13}C NMR** (176 MHz, D_2O) δ_C 173.6 (**C**OCH_3), 171.1 (C1), 58.5 (C2), 44.7 (C3), 22.5 (CO**C**H_3). **HRMS-ESI** $[M+H]^+$ calc. for $C_5H_9N_2O_2^+$ 129.0659; obs. 129.0662.

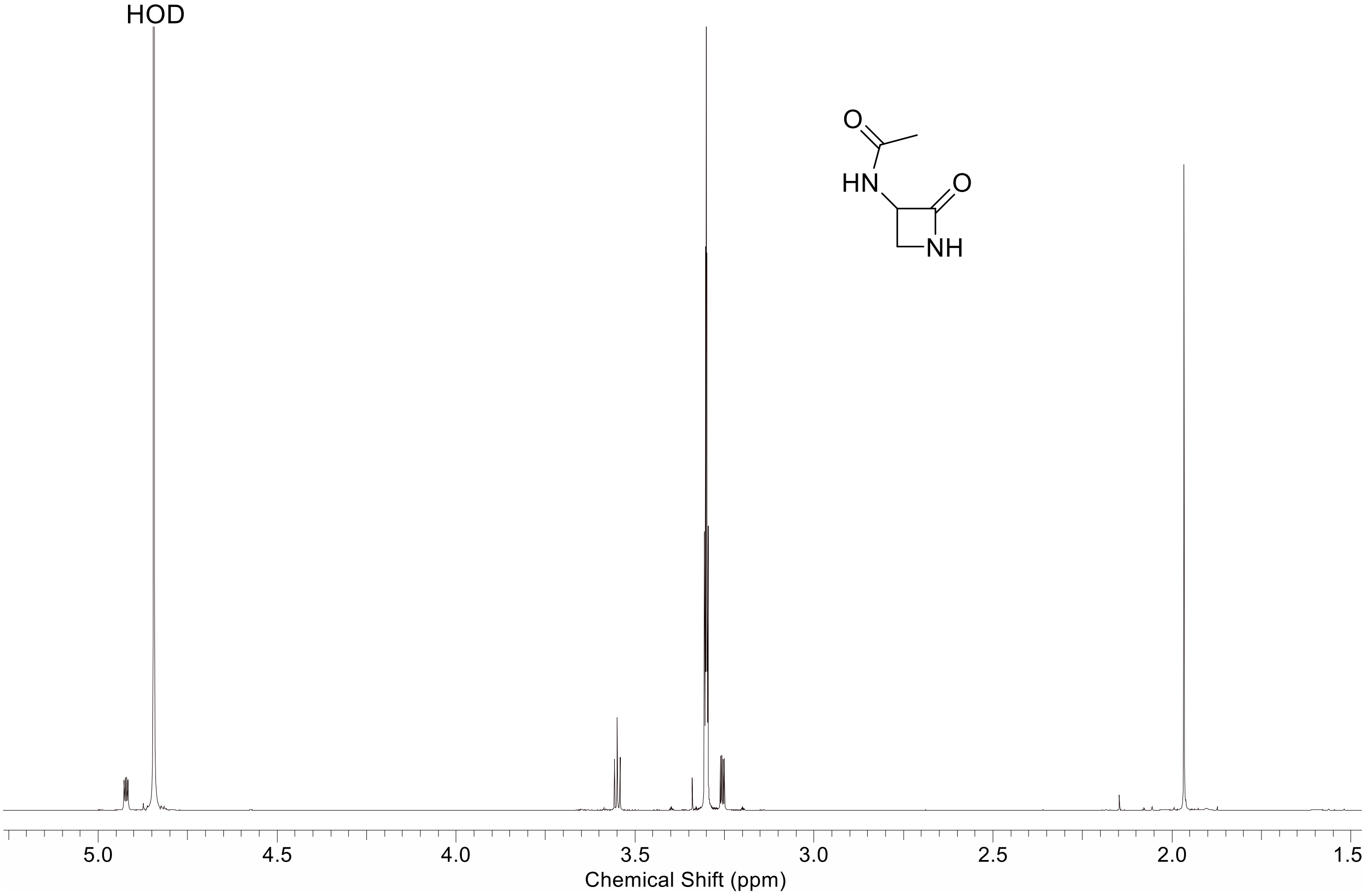

*Supplementary Figure 144. ^{1}H NMR (700 MHz, CD_3OD, 1.5 – 5.2 ppm) spectrum of **4**.*

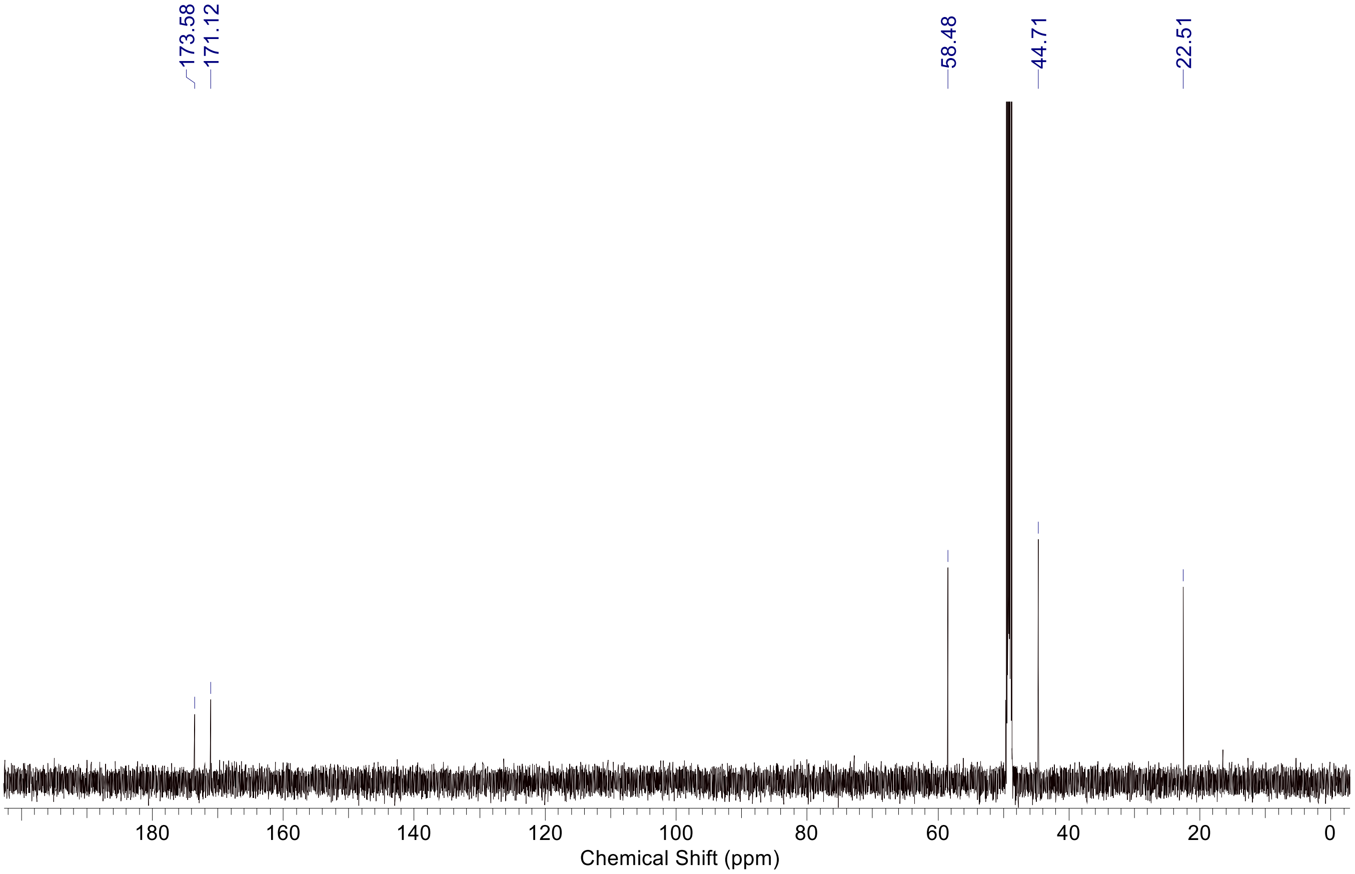

Supplementary Figure 145. *^{13}C NMR (176 MHz, CD_3OD, 0 – 200 ppm) spectrum of* ***4****.*

Ac-Dpr-OH·HCl [8]

To **Ac-Dpr(Boc)-OH** (13.2 mg, 0.05 mmol) in EtOAc/dioxane (0.4 mL, 1:1) at 0 °C was added 4 M HCl in dioxane (0.2 mL, 0.80 mmol). The reaction mixture was stirred at 0 °C for 5 min and then at room temperature for 16 h. The suspension was centrifuged, the precipitate washed with Et_2O (3 × 0.2 mL) and dried *in vacuo* to afford **Ac-Dpr-OH**·HCl (9.30 mg, 0.05 mmol, 95%). **^{1}H NMR** (400 MHz, D_2O) δ_H 4.70 (ABX, J = 5.3, 8.3 Hz, 1H, (C2)–H), 3.53 (ABX, J = 5.3, 13.4 Hz, 1H, (C3)–H), 3.32 (ABX, J = 8.3, 13.4 Hz, 1H, (C3)–H'), 2.08 (s, 3H, $COCH_3$). **^{13}C NMR** (100 MHz, D_2O) δ_C 175.4 (**C**OCH_3), 172.4 (C1), 50.9 (C2), 40.5 (C3), 22.3 (CO**C**H_3). **HRMS-ESI** $[M+H]^+$ calc. for $C_5H_{11}N_2O_3^+$ 147.0764; obs. 147.0764. **IR** (solid, cm^{-1}): 2858, 1729 (br), 1641, 1545, 1374,1211.

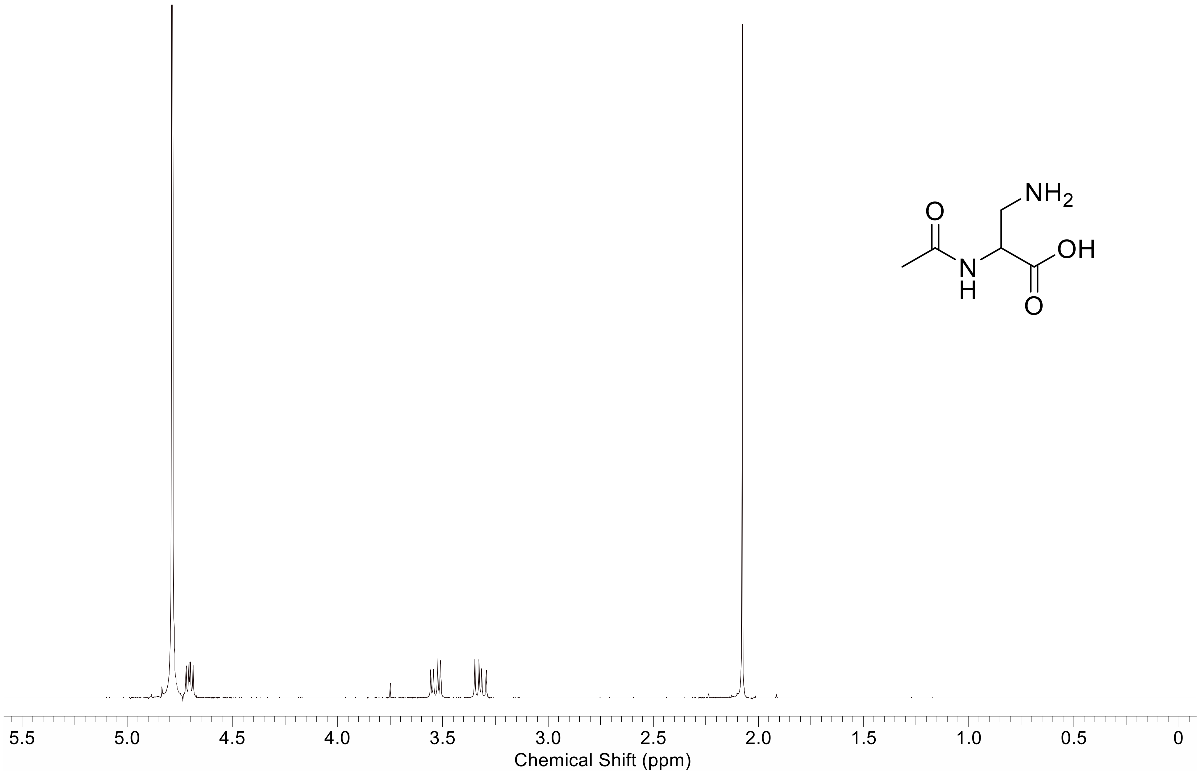

*Supplementary Figure 146. ^{1}H NMR (400 MHz, D_2O, 0.0 – 5.5 ppm) spectrum of **Ac-Dpr-OH**.*

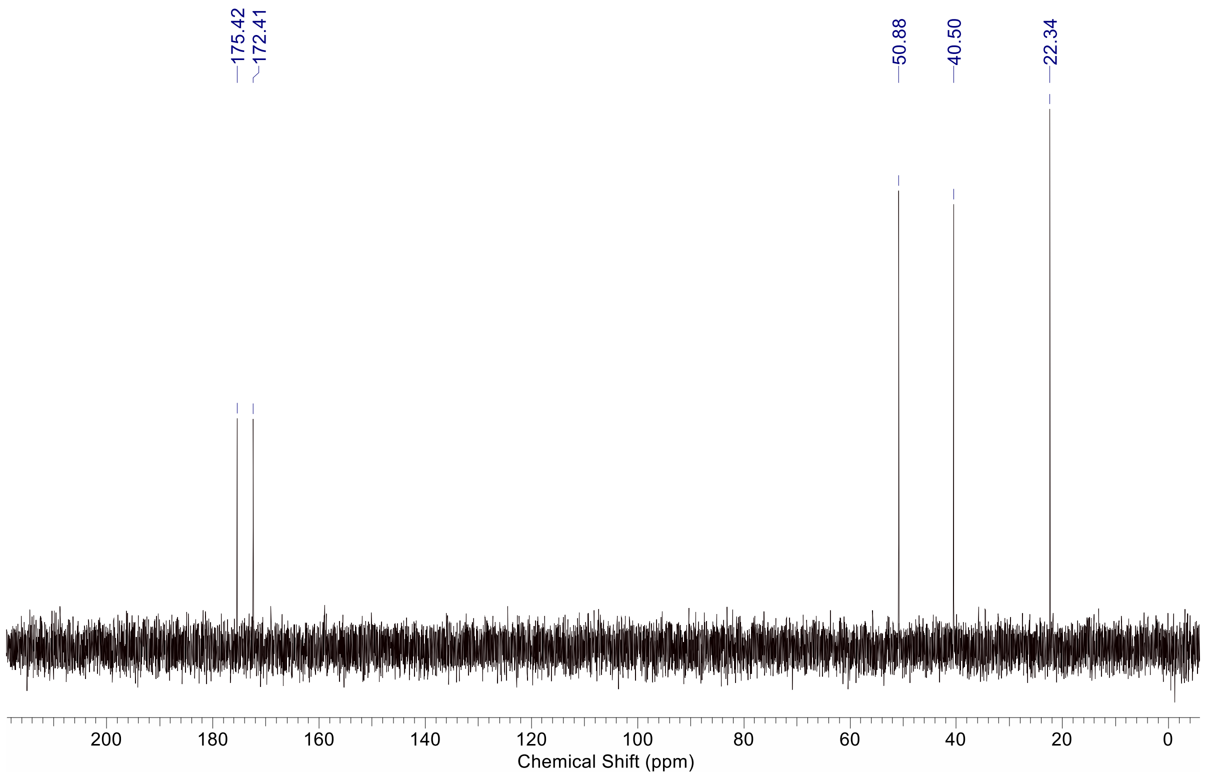

*Supplementary Figure 147. ^{13}C NMR (100 MHz, D_2O, 0 – 200 ppm) spectrum of **Ac-Dpr-OH**.*

N-Fluoren-9-ylmethoxycarbonylaminoacetaldehyde[13]

To 3-amino-1,2-propanediol (1.19 g, 11.4 mmol) in dioxane/water (1:1, 20 mL) at 0 °C was added *N*-(9-fluorenylmethoxycarbonyloxy)succinimide (2.8 g, 8.3 mmol). The reaction mixture was stirred at room temperature for 16 h, diluted with water (20 mL) and extracted with EtOAc (3 × 15 mL). The combined organic layers were washed with water (3 × 15 mL), brine (15 mL), dried over anhydrous Na_2SO_4, filtered, and concentrated *in vacuo* to afford the crude *N*-Fmoc diol (3.97 g) as a white powder which was used immediately without further purification. To the above crude mixture in MeCN/water (1:1, 30 mL) at 30 °C was added $NaIO_4$ (2.13 g, 9.90 mmol). After 3 h, volatiles were removed *in vacuo* and the crude residue was diluted with water (30 mL), extracted with CH_2Cl_2 (3 × 20 mL), dried over anhydrous Na_2SO_4, filtered and concentrated *in vacuo* to afford the title compound (1.95 g, 6.93 mmol, 61% over 2 steps) as an off-white powder. **^{1}H NMR** (500 MHz, $CDCl_3$) δ_H 9.67 (s, 1H, (C1)–H), 7.78 (d, *J* = 7.6 Hz, 2H, $(C_6H_4)_2$), 7.61 (d, *J* = 7.4 Hz, 2H, $(C_6H_4)_2$), 7.42 (t, *J* = 7.4 Hz, 2H, $(C_6H_4)_2$), 7.33 (t, *J* = 7.4 Hz, 2H, $(C_6H_4)_2$), 5.45 (br s, 1H, NH), 4.44 (d, *J* = 6.9 Hz, 2H, $OC\mathbf{H}_2CH(C_6H_4)_2$), 4.25 (t, *J* = 6.9 Hz, 1H, $OCH_2C\mathbf{H}(C_6H_4)_2$), 4.17 (d, *J* = 5.0 Hz, 2H, (C2)–H). **^{13}C NMR** (126 MHz, $CDCl_3$) δ_C 196.4 (C1), 156.3 ($\mathbf{C}OOCH_2CH(C_6H_4)_2$), 143.9 (2C, $(C_6H_4)_2$), 141.5 (2C, $(C_6H_4)_2$), 127.9 (2C, $(C_6H_4)_2$), 127.2 (2C, $(C_6H_4)_2$), 125.2 (2C, $(C_6H_4)_2$), 120.2 (2C, $(C_6H_4)_2$), 67.4 ($O\mathbf{C}H_2CH(C_6H_4)_2$), 51.8 (C2), 47.3 ($OCH_2\mathbf{C}H(C_6H_4)_2$). **HRMS-ESI** $[M+H]^+$ calc. for $C_{17}H_{16}NO_3^+$ 282.1125; obs. 282.1119. **IR** (solid, cm^{-1}): 3339, 1710, 1686, 1534.

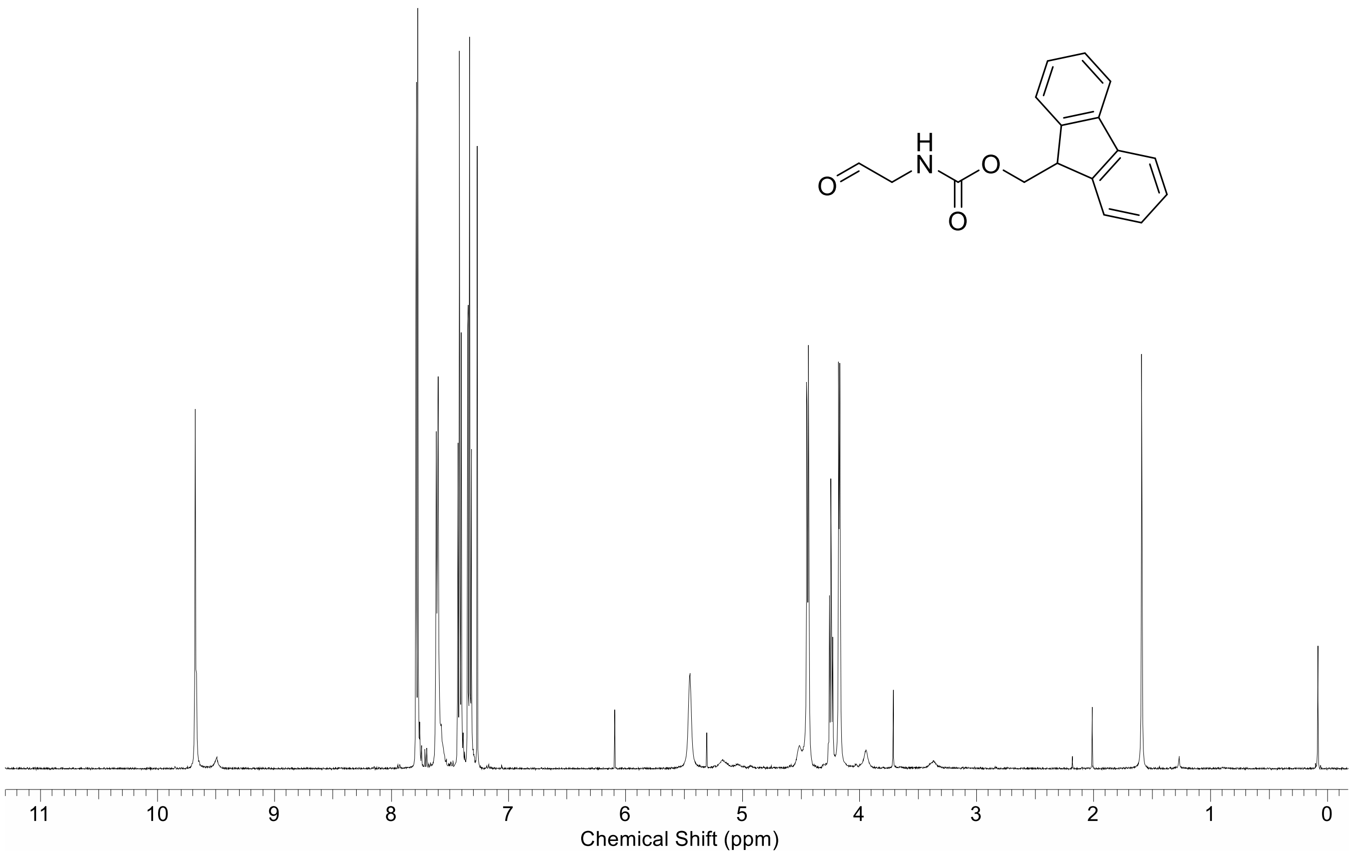

Supplementary Figure 148. ^{1}H NMR (500 MHz, $CDCl_3$, 0.0 – 11.0 ppm) spectrum of N-fluoren-9-ylmethoxycarbonylaminoacetaldehyde.

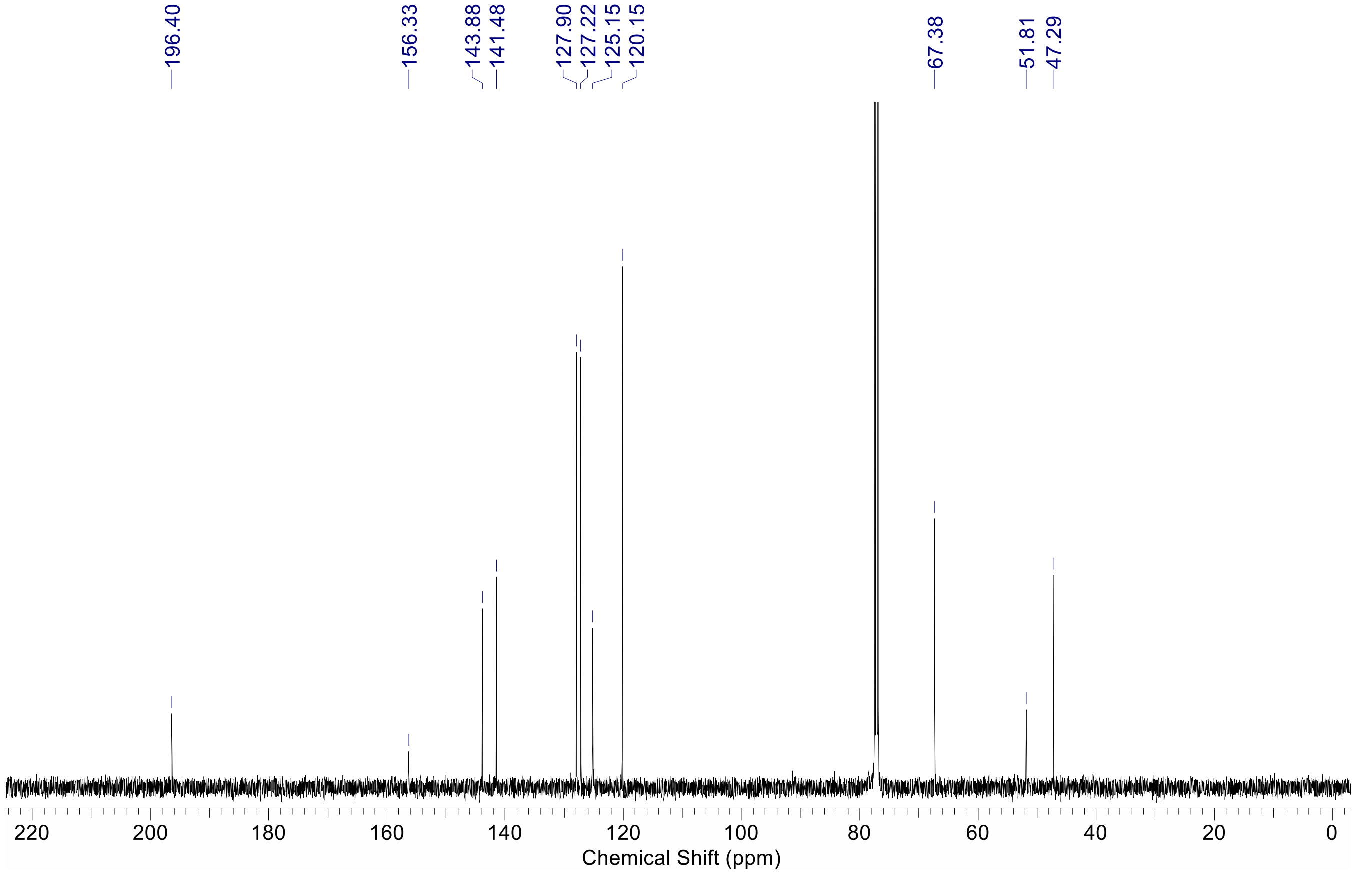

Supplementary Figure 149. ^{13}C NMR (126 MHz, $CDCl_3$, 0 – 220 ppm) spectrum of N-fluoren-9-ylmethoxycarbonylaminoacetaldehyde.

Ac-Dpr(Fmoc)-CN

To *N*-fluoren-9-ylmethoxycarbonylaminoacetaldehyde (680 mg, 2.42 mmol) in dioxane (5 mL) was added NaCN (142 mg, 2.9 mmol) and 30-33% NH_4OH (3.15 mL) pre-mixed in H_2O (10 mL) at pH 10. Dioxane (5 mL) was added, and the reaction stirred at room temperature for 16 h. The reaction mixture was diluted with H_2O (10 mL) and extracted with EtOAc (3 × 25 mL). The organic layers were combined and washed with H_2O (25 mL) followed by brine (25 mL), dried over anhydrous Na_2SO_4, filtered and concentrated *in vacuo* to afford crude **H-Dpr(Fmoc)-CN** (880 mg) as a yellow oil. The crude aminonitrile was dissolved in $CHCl_3$ (10 mL) and Ac_2O (0.34 mL, 3.63 mmol) was added dropwise. After 20 min, volatiles were removed *in vacuo*. Purification by column chromatography (gradient, 40:60 petroleum ether to EtOAc) afforded **Ac-Dpr(Fmoc)-CN** (374 mg, 1.07 mmol, 44% over 2 steps) as a white powder. **^{1}H NMR** (500 MHz, d_6-DMSO) δ_H 8.66 (d, *J* = 8.0 Hz, 1H, $(C_6H_4)_2$), 7.89 (d, *J* = 7.6 Hz, 2H, $(C_6H_4)_2$), 7.73 (t, *J* = 5.8 Hz, 1H, $(C_6H_4)_2$), 7.42 (t, *J* = 7.4 Hz, 2H, $(C_6H_4)_2$), 7.33 (t, *J* = 7.4 Hz, 2H, $(C_6H_4)_2$), 4.82 (dd, *J* = 14.5, 7.8 Hz, 1H, (C2)–H), 4.35 (d, *J* = 6.9 Hz, 2H, OC**H**$_2$CH$(C_6H_4)_2$), 4.24 (t, *J* = 6.7 Hz, 1H, OCH$_2$C**H**$(C_6H_4)_2$), 3.36-3.42 (m, 1H, (C3)–H), 3.24-3.29 (m, 1H, (C3)–H′), 1.87 (s, 3H, $COCH_3$). **^{13}C NMR** (126 MHz, d_6-DMSO) δ_C 169.3 (**C**OCH$_3$), 156.2 (**C**OOCH$_2$CH$(C_6H_4)_2$), 143.7 ($(C_6H_4)_2$), 143.7 ($(C_6H_4)_2$), 140.7 ($(C_6H_4)_2$), 127.6 ($(C_6H_4)_2$), 127.0 ($(C_6H_4)_2$), 125.0 ($(C_6H_4)_2$), 120.1 ($(C_6H_4)_2$), 118.2 (C1), 65.7 (O**C**H$_2$CH$(C_6H_4)_2$), 46.6 (OCH$_2$**C**H$(C_6H_4)_2$), 41.6 (C3), 40.2 (C2), 22.2 (CO**C**H$_3$). **HRMS-ESI** $[M+H]^+$ calc. for $C_{20}H_{20}N_3O_3^+$ 350.1499; obs. 350.1493. **IR** (solid, cm^{-1}): 3325, 1694, 1662, 1541, 1522. **R$_f$** = 0.5 in EtOAc.

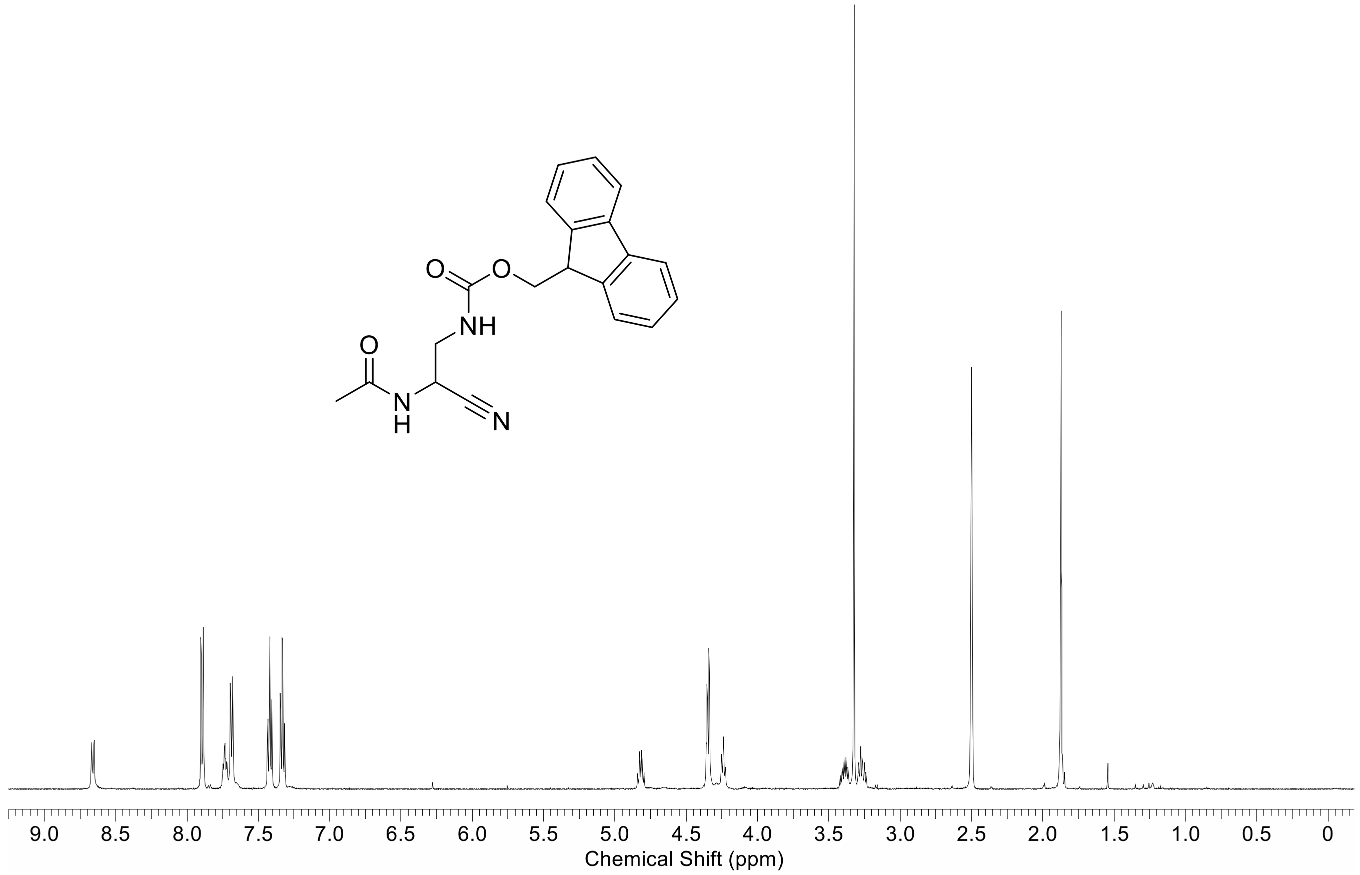

*Supplementary Figure 150. ^{1}H NMR (500 MHz, d_6-DMSO, 0.0 – 9.0 ppm) spectrum of **Ac-Dpr(Fmoc)-CN**.*

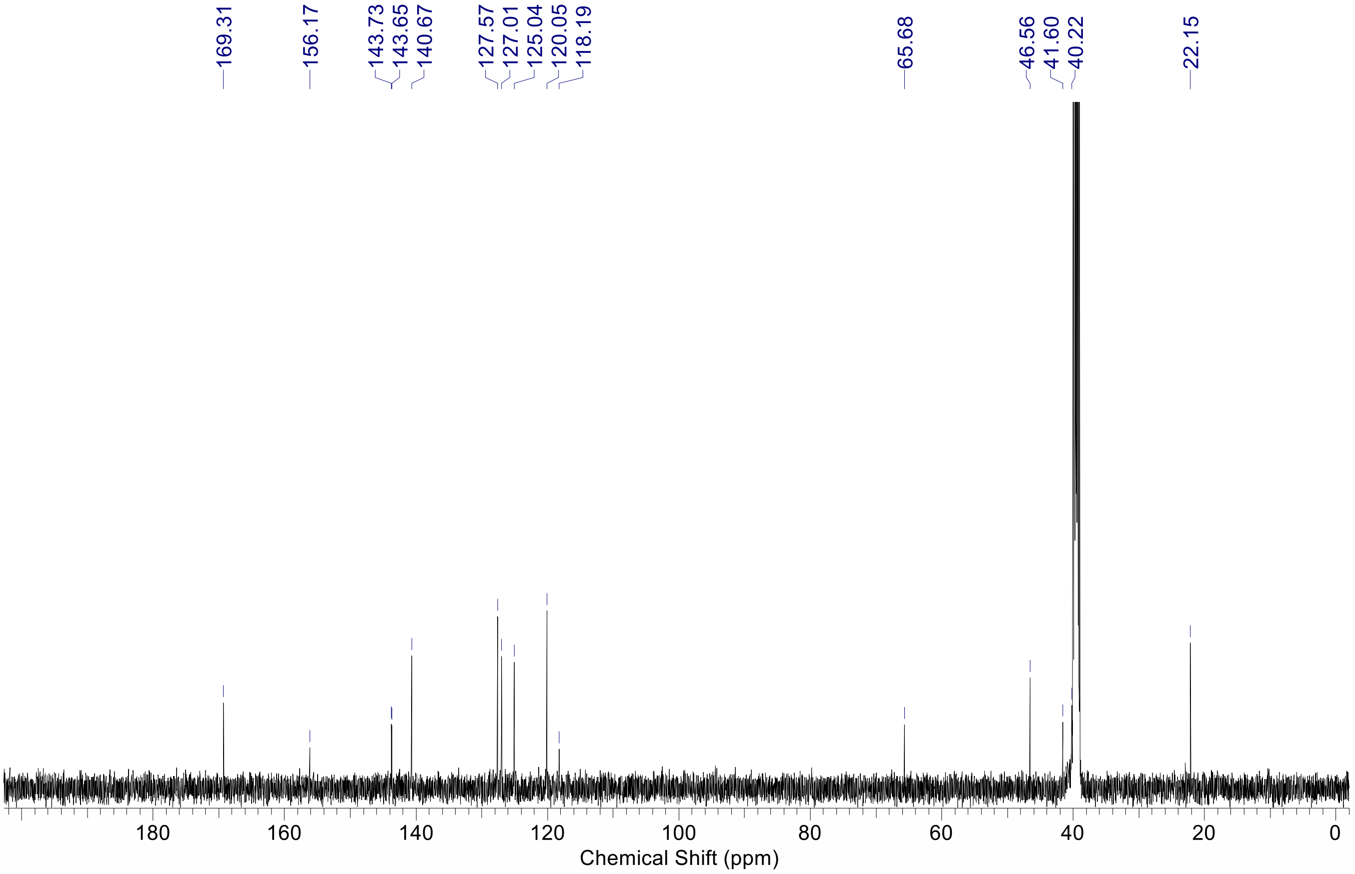

Supplementary Figure 151. ^{13}C NMR (126 MHz, d_6-DMSO, 0 – 200 ppm) spectrum of ***Ac-Dpr(Fmoc)-CN****.*

Ac-Dpr-CN

To **Ac-Dpr(Fmoc)-CN** (157 mg, 0.45 mmol) in CH_2Cl_2 (7.0 mL) was added morpholine (7.00 mL) dropwise and the reaction mixture stirred for 2 h. Volatiles were removed *in vacuo* and the crude residue purified by column chromatography (gradient, EtOAc to 4:1 EtOAc/MeOH; R_f = 0.17 in 4:1 EtOAc/MeOH) to afford **Ac-Dpr-CN** (44 mg, 0.35 mmol, 77%) as a colourless oil. **^{1}H NMR** (700 MHz, 9:1 H_2O/D_2O, noesygppr1d, partial assignment) δ_H 3.11 (1H, ABX, *J* = 13.5, 6.2 Hz), 3.07 (1H, ABX, *J* = 13.5, 7.0 Hz), 2.08 (3H, s). **^{13}C NMR** (176 MHz, 9:1 H_2O/D_2O) δ_H 174.9 (**C**OCH$_3$), 118.7 (C1), 44.0 (C2), 42.8 (C3), 22.3 (CO**C**H$_3$). **HRMS-ESI** $[M+H]^+$ calc. for $C_5H_{10}N_3O^+$ 128.0818; obs. 128.0818. **IR** (oil, cm^{-1}): 3257 (br), 3041, 2933, 2245, 1659, 1537.

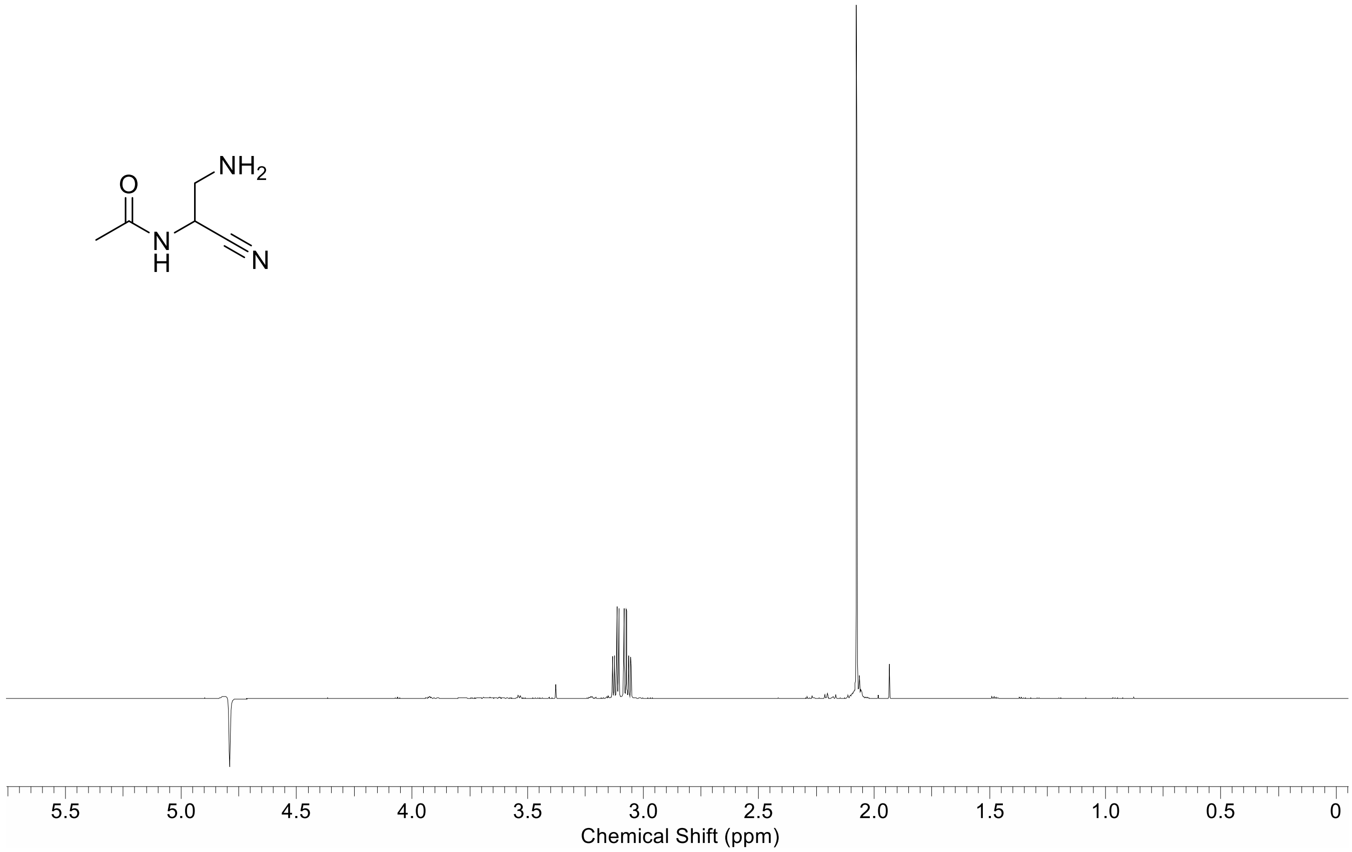

*Supplementary Figure 152. ^{1}H NMR (700 MHz, 9:1 H_2O/D2O, noesygppr1d, 0.0 – 5.5 ppm) spectrum of **Ac-Dpr-CN**. The (C2)–H of **Ac-Dpr-CN** is under water, visible by ^{1}H-^{13}C HMBC*

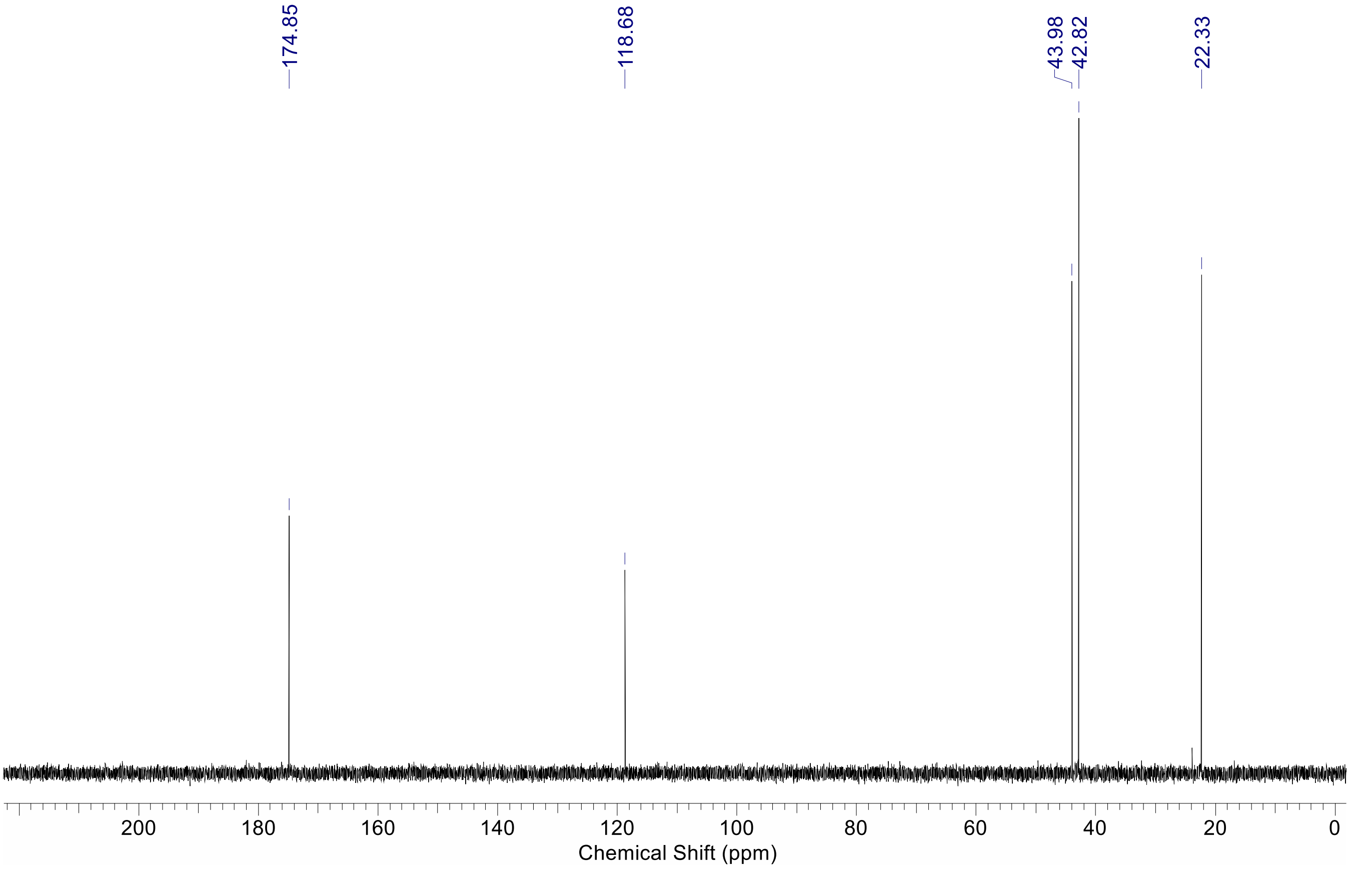

Supplementary Figure 153. ^{13}*C NMR (176 MHz, 9:1* H_2O/D_2O*, 0 – 220 ppm) spectrum of* ***Ac-Dpr-CN****.*

Ac-Lys(Boc)-SNH2

To **Fmoc-Lys(Boc)-SNH2**[14] (483 mg, 1.00 mmol) was added $CHCl_3$/DBU (96:4, 2.50 mL). The reaction mixture was stirred for 1 h at room temperature and then purified by column chromatography (EtOAc to EtOAc/MeOH; 8:2; R_f = 0.29 in 8:2 EtOAc/MeOH) to afford **H-Lys(Boc)-SNH2** (150 mg, 0.57 mmol, 57%) as a colourless oil. The oil was dissolved in $CHCl_3$ (2.5 mL) and Ac_2O (0.46 mL, 4.9 mmol) and NEt_3 (0.68 mL, 4.9 mmol) were added concurrently. The reaction mixture was stirred for 10 min at rt, immobilised onto silica and purified by column chromatography (gradient, 100% 40:60 petroleum ether to 100% EtOAc) to afford **Ac-Lys(Boc)-SNH2** (77 mg, 0.25 mmol, 45%) as a white powder. **^{1}H NMR** (700 MHz, CD_3OD) δ_H 4.57 (dd, *J* = 8.6, 5.4 Hz, 1H, (C2)–H), 3.03 (t, *J* = 7.0 Hz, 2H, (C6)–H_2), 1.99 (s, 3H, $COCH_3$), 1.85-1.91 (m, 1H, (C3)–H), 1.68-1.74 (m, 1H, (C3)–H′), 1.34-1.52 (overlapping m, 4H, (C4)–H_2, (C5)–H_2), 1.43 (s, 9H, (CO)OC$(CH_3)_3$). **^{13}C NMR** (176 MHz, CD_3OD) δ_C 210.5 (C1), 173.2 (**C**OCH_3), 158.7 ((**C**O)OC$(CH_3)_3$), 80.0 ((CO)O**C**$(CH_3)_3$), 60.1 (C2), 41.3 (C6), 36.0 (C3), 30.8 (C5), 28.9 (3C, (CO)OC(**C**$H_3)_3$), 24.4 (C4), 22.8 (CO**C**H_3). **HRMS-ESI** $[M+H]^+$ calc. for $C_{13}H_{26}N_3O_3S^+$ 304.1689; obs. 304.1689. **IR** (solid, cm^{-1}): 3305 (br), 2930, 1655 (br), 1518. **R_f** = 0.30 in EtOAc.

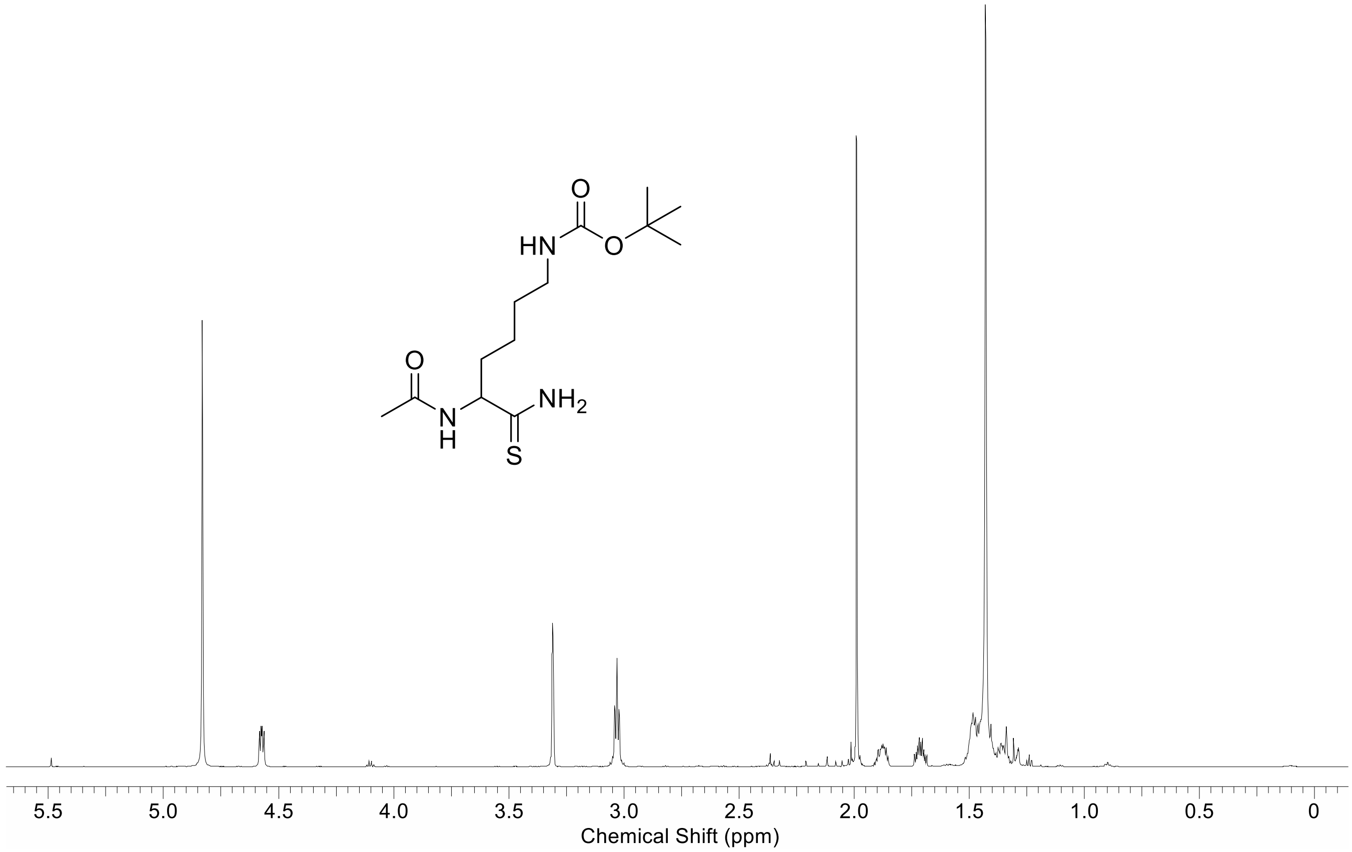

*Supplementary Figure 154. ^{1}H NMR (700 MHz, CD_3OD, 0.0 – 5.5 ppm) spectrum of **Ac-Lys(Boc)-SNH2.***

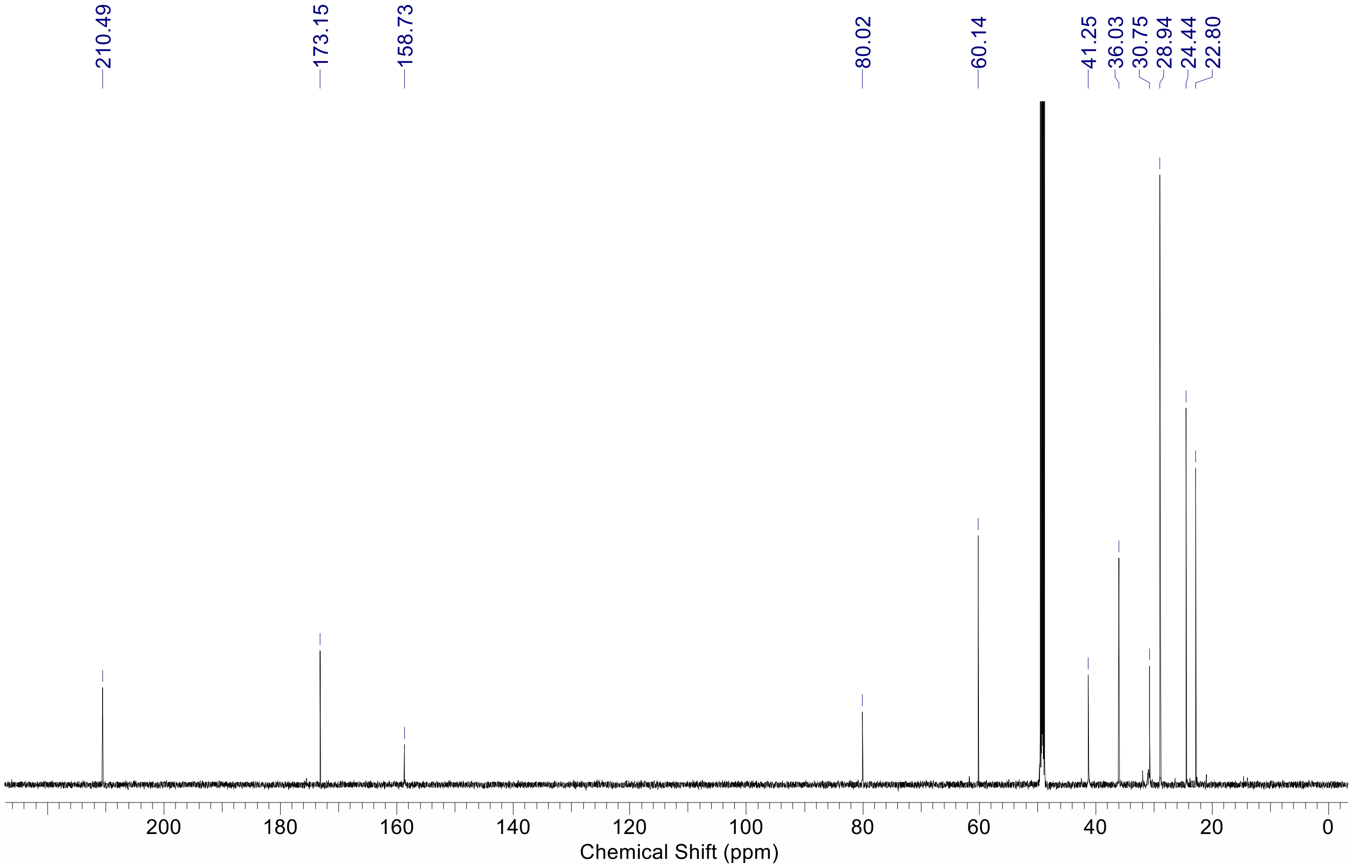

Supplementary Figure 155. ^{13}C NMR (176 MHz, CD_3OD, 0 – 220 ppm) spectrum of ***Ac-Lys(Boc)-SNH$_2$****.*

Ac-Lys-SNH$_2$·HCl

To **Ac-Lys(Boc)-SNH$_2$** (70 mg, 0.23 mmol) under an N_2 atmosphere was added 4 N HCl in dioxane (5.00 mL). The reaction mixture was stirred at room temperature for 5 mins, diluted with Et_2O (10 mL), centrifuged and the white pellet triturated with EtOAc (6 × 5 mL), dried *in vacuo* to afford **Ac-Lys-SNH$_2$**·HCl (33.6 mg, 0.14 mmol, 61%) as a white powder. **^{1}H NMR** (700 MHz, D_2O) δ_H 4.54 (dd, *J* = 9.0, 5.1 Hz, 1H, (C2)–H), 3.00 (app t, *J* = 6.7 Hz, 2H, (C6)–H), 2.05 (s, 3H, $COCH_3$), 1.92-1.97 (m, 1H, (C3)–H), 1.77-1.82 (m, 1H, (C3)–H′), 1.67-1.72 (m, 2H, (C5)–H_2), 1.41-1.53 (m, 2H, (C4)–H_2). **^{13}C NMR** (176 MHz, D_2O) δ_C 209.1 (C1), 174.9 (**C**OCH_3), 60.0 (C2), 39.8 (C6), 33.7 (C3), 26.8 (C5), 23.4 (C4), 22.4 (CO**C**H_3). **HRMS-ESI** $[M+H]^+$ calc. for $C_8H_{18}N_3OS^+$ 204.1165; obs. 204.1166.

A by-product of deprotection was observed, which we tentatively assigned as the oxazole **19** based on NMR data.[2] No further attempts at purification were made.

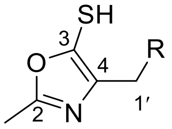

19 ▽

19 (▽) **^{1}H NMR** (700 MHz, D_2O, partial assignment) δ_H 2.76 (s, 3H, CH_3), 2.73-2.75 (m, 2H, (C1′)–H_2). **^{13}C NMR** (176 MHz, D_2O, partial assignment) δ_C 160.0 (C2), 142.2 (C3), 127.9 (C4), 25.2, 15.6 (CH_3).

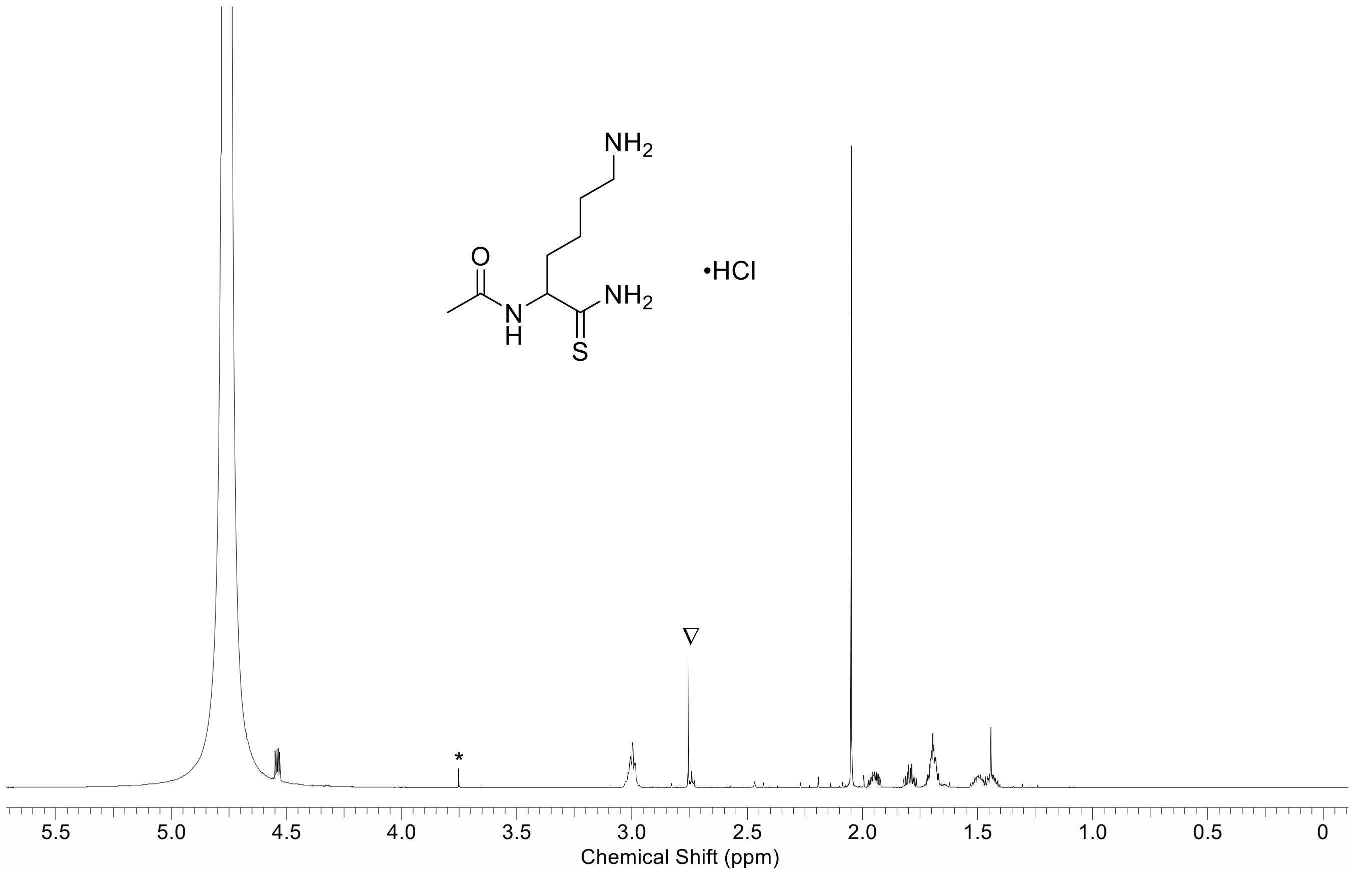

*Supplementary Figure 156. ^{1}H NMR (700 MHz, D_2O, 0.0 – 5.5 ppm) spectrum of **Ac-Lys-SNH$_2$**. ***** Dioxane.*

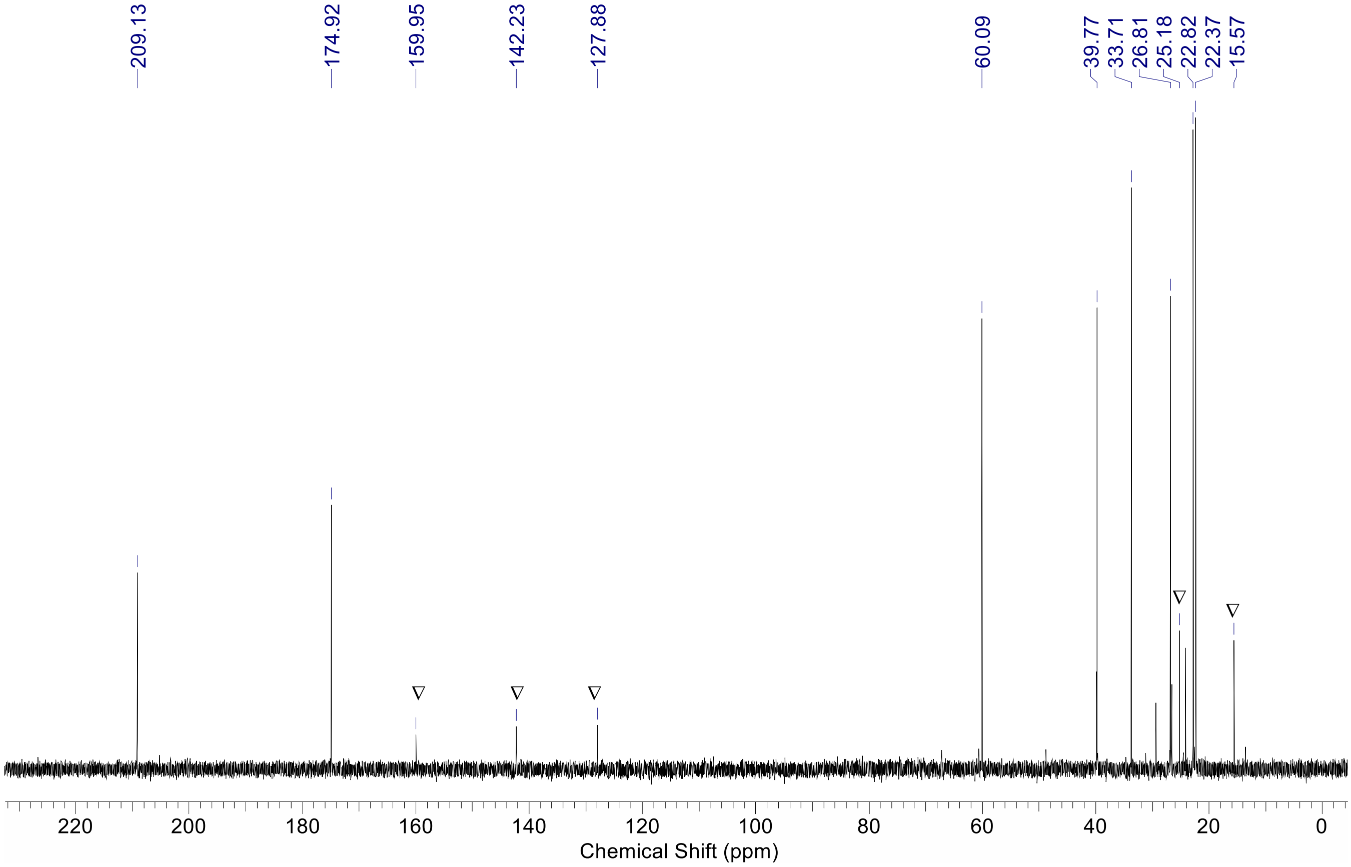

*Supplementary Figure 157. ^{13}C NMR (176 MHz, D_2O, 0 – 225 ppm) spectrum of **Ac-Lys-SNH₂**.*

Synthesis of Ac-Lys-CN

(5-Oxo-pentyl)-carbamic acid benzyl ester 20[15]

To 5-amino-*N*-benzyloxycarbonylpentanol[16] (1.00 g, 4.48 mmol) was added (2,2,6,6-tetramethylpiperidin-1-yl)oxyl (9.80 mg, 0.06 mmol) in CH_2Cl_2 (2.0 mL) and potassium bromide (53.3 mg, 0.45 mmol) in H_2O (2.0 mL) at 0 °C. 1 M aq. NaOCl pre-adjusted to pH 9.2 with $NaHCO_3$ was added dropwise. The reaction mixture was stirred for 1.5 h before $Na_2S_2O_3$ (177 mg, 1.12 mmol) and $NaHCO_3$ (94.1 mg, 1.12 mmol) in H_2O (3.0 mL) was added. The reaction mixture was diluted with CH_2Cl_2 (25 mL), the organic layer washed with H_2O (3 × 15 mL), dried over anhydrous Na_2SO_4, filtered and concentrated *in vacuo* to afford the crude product as a mixture of (5-oxo-pentyl)-carbamic acid benzyl ester and its cyclic isomer *N*-benzyloxycarbonyl-2-hydroxypiperidine (918 mg, 3.90 mmol, 87%) which was used without further purification.

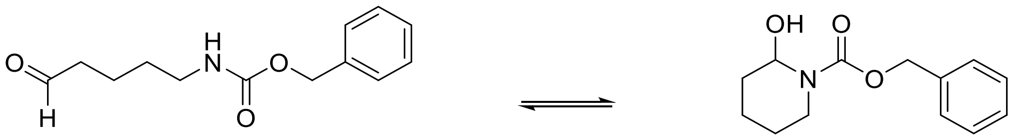

20 ● **21** ■

(5-Oxo-pentyl)-carbamic acid benzyl ester **20** was observed to slowly convert to *N*-benzyloxycarbonyl-2-hydroxypiperidine **21** if left in solution. This could be used to assign some $^{1}H/^{13}C$ signals alongside 2D NMR spectra.

20 (●): **^{1}H NMR** (500 MHz, $CDCl_3$, partial assignment) δ_H 9.77 (br s, 1H, (C1)–H), 5.10 (br s, 2H, CH_2OPh), 2.49 (t, *J* = 7.0 Hz, 2H, (C2)–H_2). **^{13}C NMR** (101 MHz, $CDCl_3$, partial assignment) δ_C 202.1 (C1), 156.6 (**C**OOBn), 136.7 (C_6H_5), 128.7 (C_6H_5), 128.2 (C_6H_5), 66.8 (O**C**H_2Ph), 43.5 (C2), 40.8 (C5), 19.2 (C3).

21 (■): **^{1}H NMR** (500 MHz, $CDCl_3$, partial assignment) δ_H 5.80 (br dd, 1H, (C1)–H), 5.16 (s, 2H, CH_2OPh), 3.65 (dd, *J* = 10.6, 5.2 Hz, 2H, (C5)–H_2). **^{13}C NMR** (101 MHz, $CDCl_3$, partial assignment) δ_C 156.6 (**C**OOBn), 136.6 (C_6H_5), 128.7 (C_6H_5), 128.1 (C_6H_5), 75.2 (C1), 67.4 (O**C**H_2Ph), 62.8 (C5), 39.5 (C2), 24.8 (C4), 17.8 (C3).

HRMS-ESI $[M-H]^-$ calc. for $C_{13}H_{16}NO_3^-$ 234.1125; obs. 234.1131.

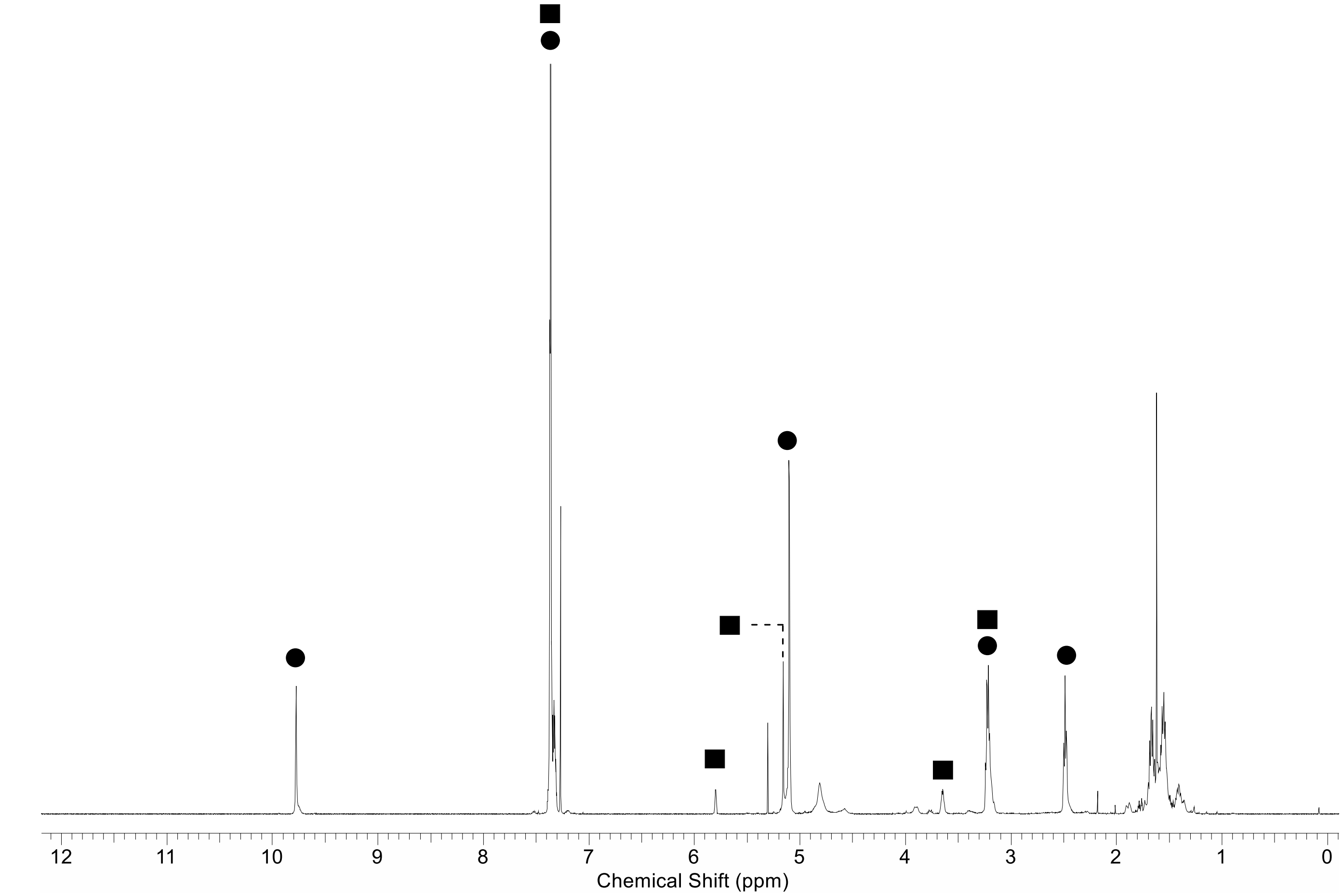

*Supplementary Figure 158. ^{1}H NMR (500 MHz, $CDCl_3$, 0.0 – 12.0 ppm) spectrum of **20** and **21**.*

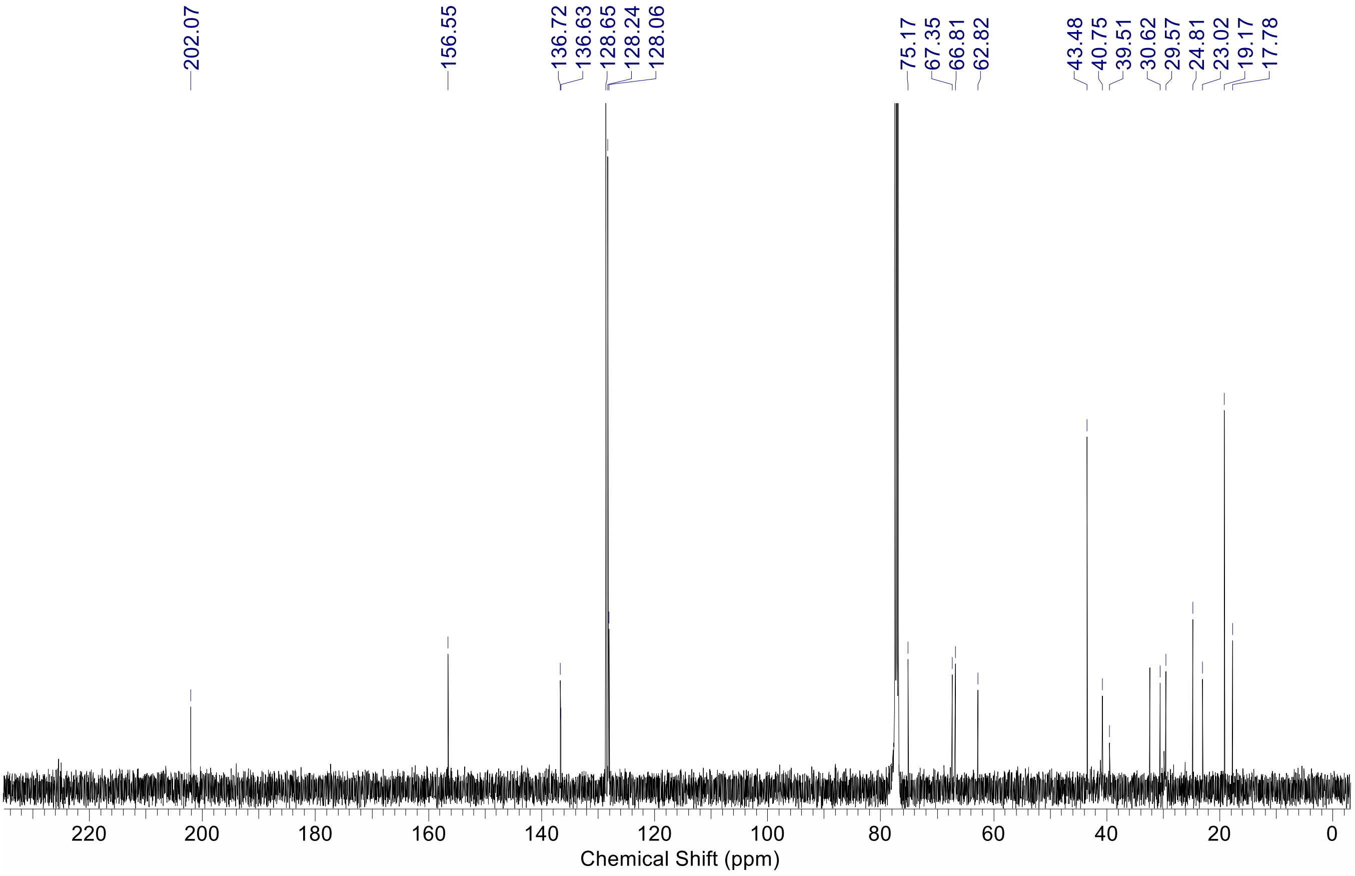

*Supplementary Figure 159. ^{13}C NMR (101 MHz, $CDCl_3$, 0 – 225 ppm) spectrum of **20** and **21**.*

Ac-Lys(CBz)-CN

To a mixture of **20** and **21** (120 mg, 0.51 mmol) in dioxane (3.0 mL) was added NaCN (50.0 mg, 1.02 mmol) and NH_4Cl (273 mg, 5.10 mmol) in H_2O (3.0 mL) pre-adjusted to pH 9. The reaction mixture was stirred for 5 d. NH_4Cl (281 mg, 5.25 mmol) was added, the reaction mixture readjusted to pH 9 with 4 M NaOH and heated at 60 °C for 16 h. The reaction mixture was diluted with H_2O (5 mL), extracted with EtOAc (3 × 5 mL) and the organic layers washed with H_2O (5 mL) followed by brine (5 mL), dried over anhydrous Na_2SO_4, filtered and concentrated *in vacuo* to afford the crude free base as a colourless oil (143 mg). To the crude product was added $CHCl_3$ (5.0 mL) and 4-(N,N-Dimethylamino)pyridine hydrochloride (7.90 mg, 0.05 mmol). Acetic anhydride (58.0 µL, 0.61 mmol) was added portionwise and the reaction mixture stirred for 45 min and volatiles were removed *in vacuo*. Purification by column chromatography (gradient, 100% 40:60 petroleum ether to 100% EtOAc) afforded the title compound as a colourless oil (45 mg, 0.17 mmol, 34 % over 2 steps). **^{1}H NMR** (600 MHz, CD_3OD) δ_H 7.27-7.37 (overlapping m, 5H, Ph), 5.06 (s, 2H, OCH_2Ph), 4.73 (t, *J* = 7.5 Hz, 1H, (C2)–H), 3.13 (t, *J* = 6.7 Hz, (C6)–H_2), 1.97 (s, 3H, $COCH_3$), 1.76-1.88 (m, 2H, (C3)–H_2), 1.52-1.56 (m, 2H, (C5)–H_2), 1.45-1.50 (m, 2H, (C4)–H_2). **^{13}C NMR** (151 MHz, CD_3OD) δ_C 173.0 (**C**OCH_3), 159.1 (COBn), 138.6 (C_6H_5), 129.6 (2C, C_6H_5), 129.1 (C_6H_5), 128.9 (2C, C_6H_5), 120.0 (C1), 67.5 (OCH_2Ph), 41.7 (C2), 41.4 (C6), 33.2 (C3), 30.2 (C5), 23.9 (C4), 22.3 (CO**C**H_3). **HRMS-ESI** $[M+H]^+$ calc. for $C_{16}H_{22}N_3O_3^+$ 304.1656; obs. 304.1652. **IR** (oil, cm^{-1}): 3307 (br), 2936, 1702, 1665, 1528. **R$_f$** = 0.35 in EtOAc.

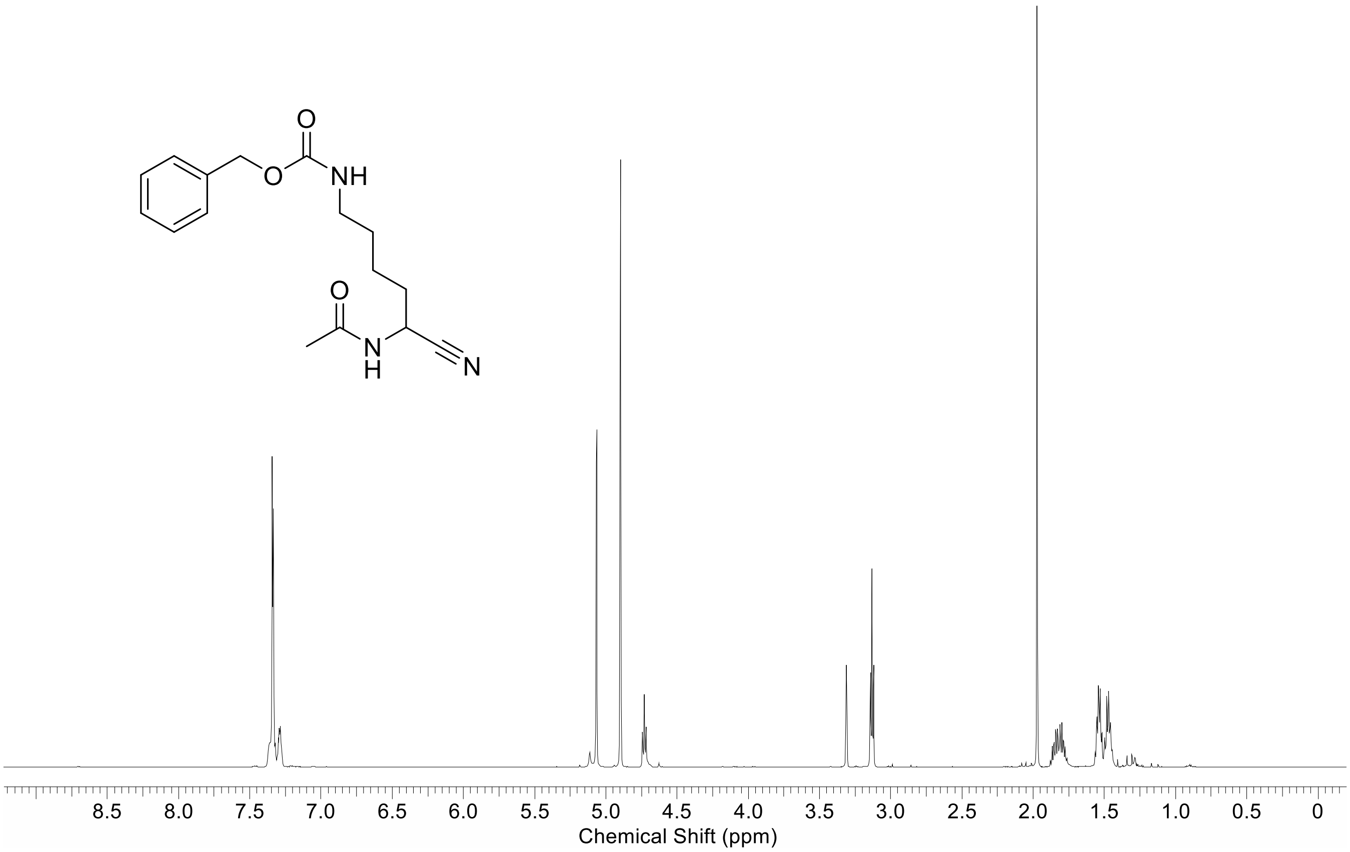

*Supplementary Figure 160. ^{1}H NMR (600 MHz, CD_3OD, 0.0 – 9.0 ppm) spectrum of **Ac-Lys(Cbz)-CN**.*

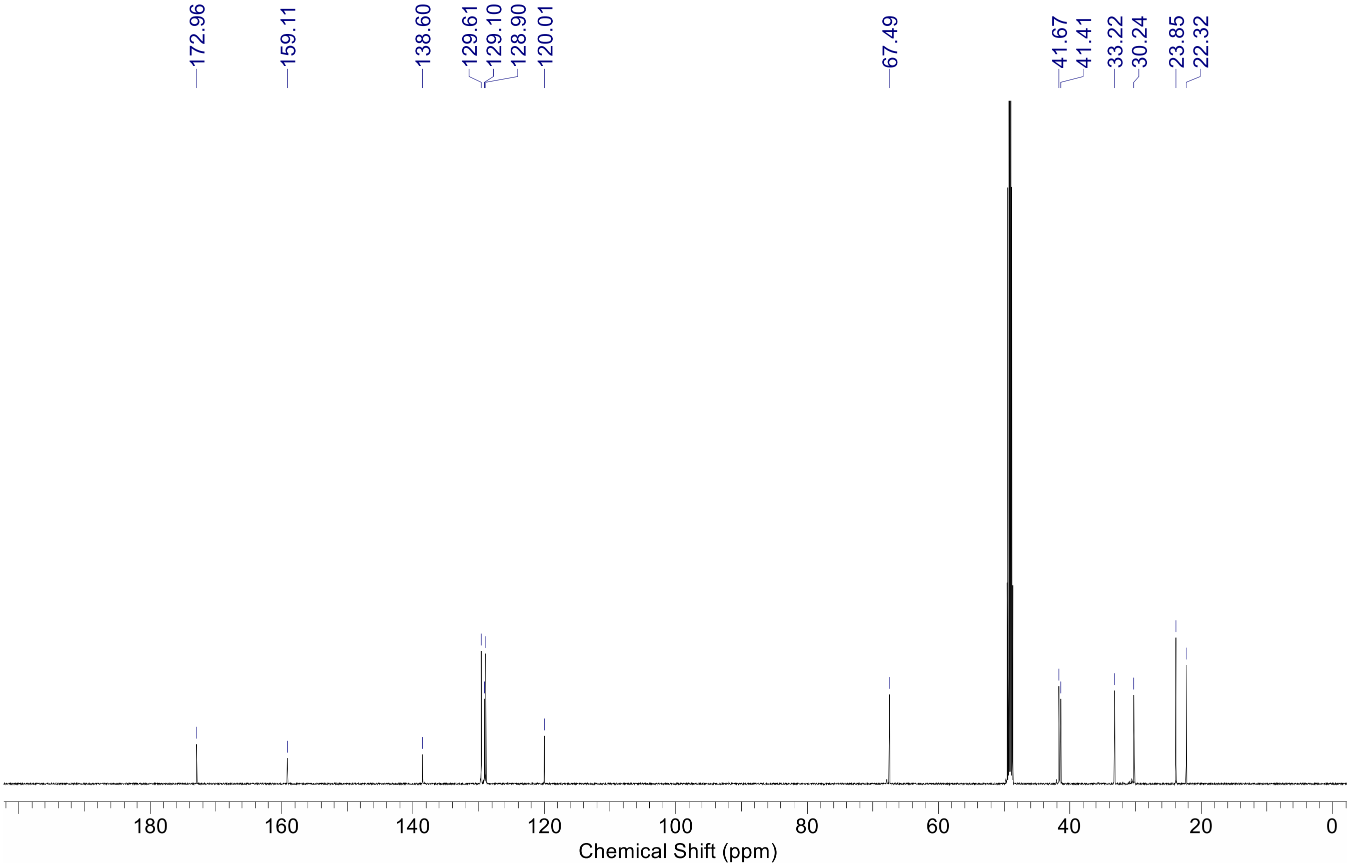

Supplementary Figure 161. ^{13}C NMR (151 MHz, CD_3OD, 0 – 200 ppm) spectrum of ***Ac-Lys(Cbz)-CN****.*

Ac-Lys-CN

To NaI (103 mg, 0.68 mmol) in MeCN (0.50 mL) under an Ar atmosphere was added trimethylsilyl chloride (87.0 µL, 0.68 mmol). The reaction mixture was stirred for 45 min before **Ac-Lys(Cbz)-CN** (42.0 mg, 0.14 mmol) was added portionwise and stirred for a further 4 h. MeCN (0.50 mL) was added, the reaction mixture filtered and then concentrated *in vacuo*. The crude residue was redissolved in CH_2Cl_2 (0.5 mL), extracted with H_2O (2 × 1.0 mL) and the combined aqueous layers washed with CH_2Cl_2 (8 × 0.5 mL) and lyophilised. Purification by column chromatography (EtOAc/NEt_3; 99:1 to EtOAc/MeOH/NEt_3; 79:20:1) afforded the product (5.1 mg, 0.03 mmol, 21%). **^{1}H NMR** (700 MHz, D_2O, partial assignment) δ_H 3.03 (app t, *J* = 7.6 Hz, (C6)–H_2), 2.05 (s, 3H, $COCH_3$), 1.90-2.01 (m, 2H, (C3)–H_2), 1.71-1.76 (m, 2H, (C5)–H_2), 1.53-1.58 (m, 2H, (C4)–H_2). **^{13}C NMR** (176 MHz, D_2O) δ_C 174.6 (**C**OCH_3), 119.8 (C1), 41.2 (C2), 39.8 (C6), 31.3 (C3), 26.6 (C5), 22.5 (C4/CO**C**H_3), 22.3 (C4/CO**C**H_3). **HRMS-ESI** $[M+H]^+$ calc. for $C_8H_{16}N_3O^+$ 170.1288; obs. 170.1282.

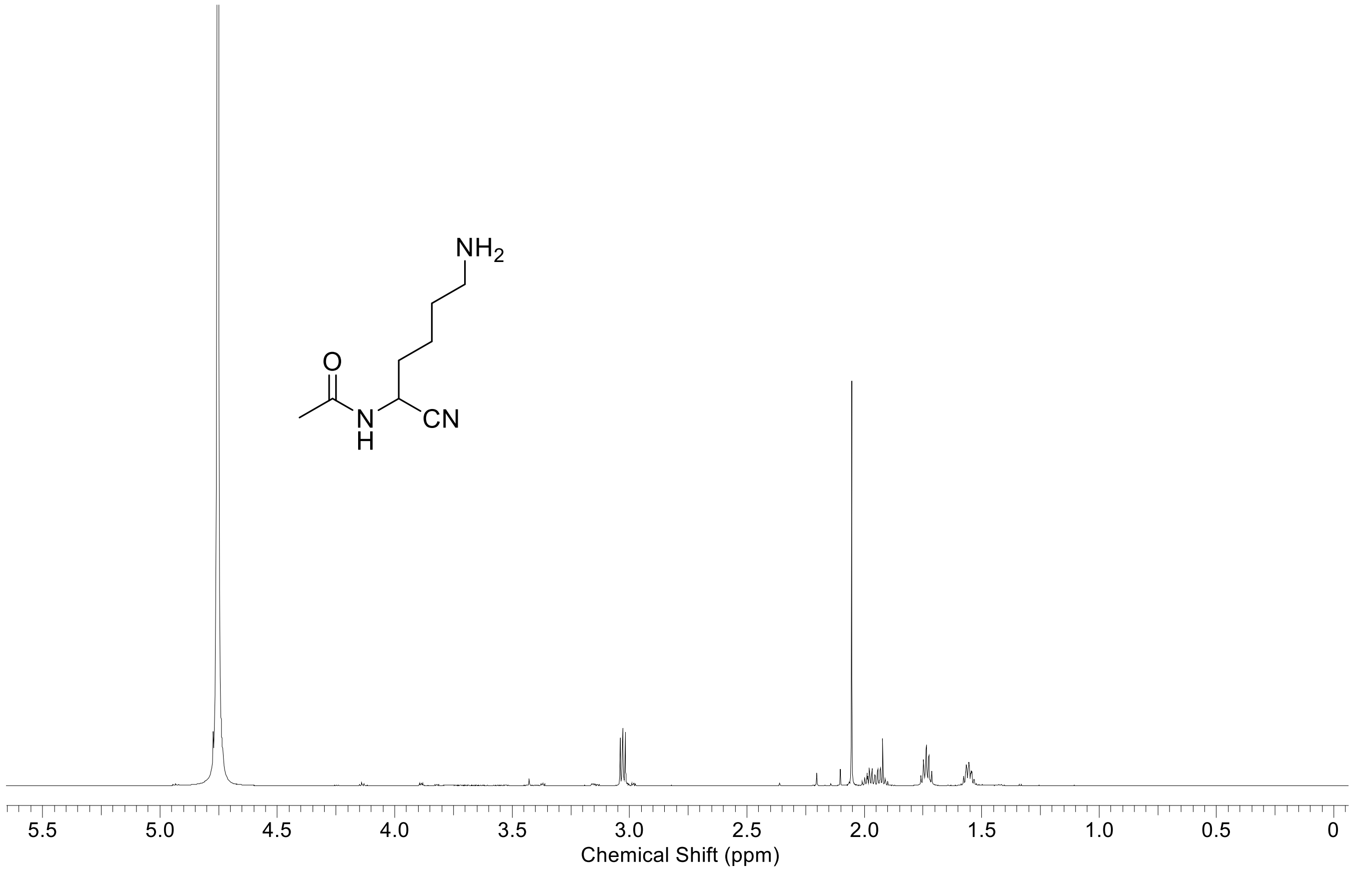

*Supplementary Figure 162. ^{1}H NMR (700 MHz, D_2O, 0.0 – 5.5 ppm) spectrum of **Ac-Lys-CN**. The (C2)–H of **Ac-Lys-CN** is under water.*

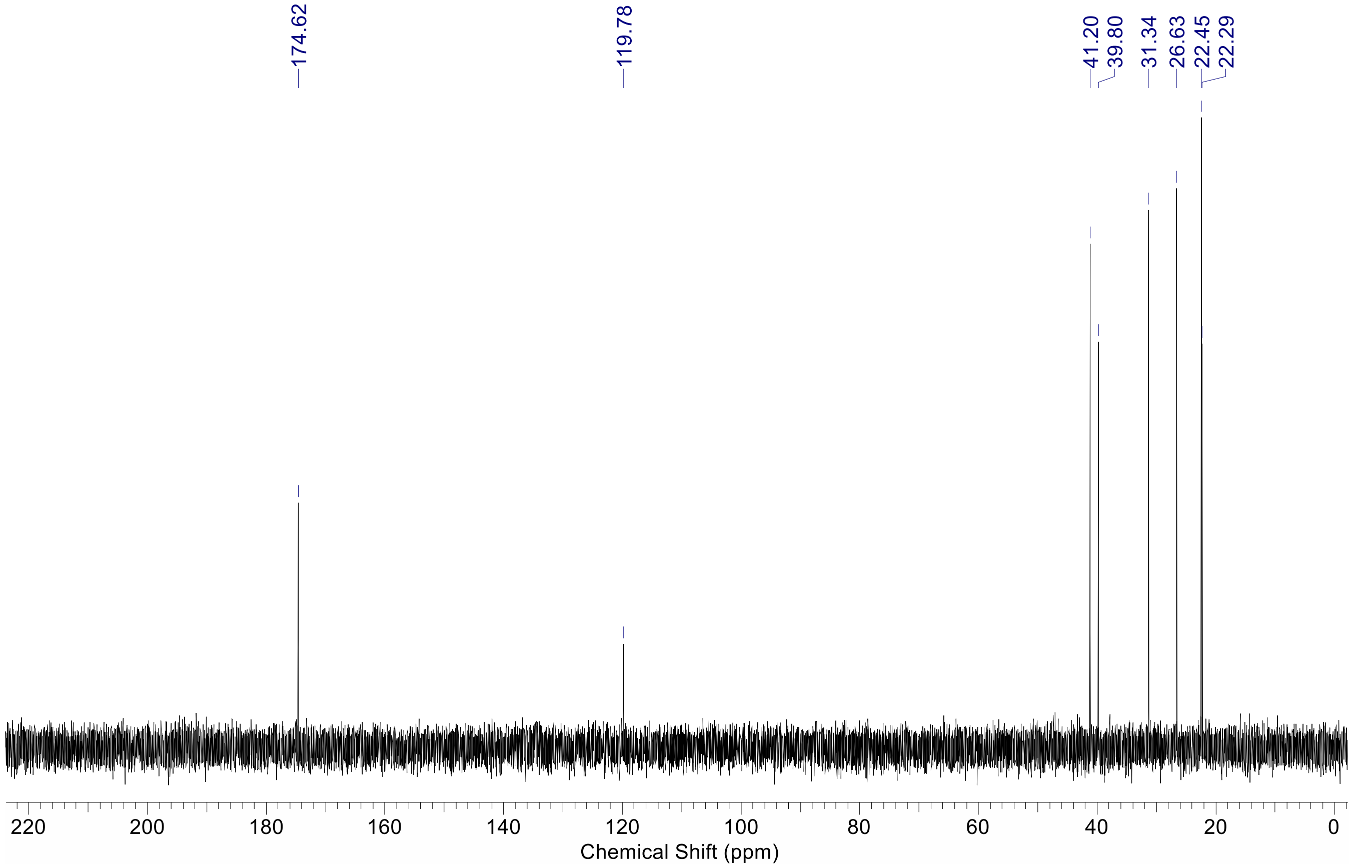

*Supplementary Figure 163. ^{13}C NMR (176 MHz, D_2O, 0 – 220 ppm) spectrum of **Ac-Lys-CN**.*

Synthesis of Dpr-CN

Dpr-OMe·2HCl[17]

To DL-2,3-diaminopropionic acid·HCl (272 mg, 1.93 mmol) in MeOH (12.0 mL) at 0 °C was added $SOCl_2$ (1.40 mL, 19.3 mmol) dropwise. The reaction mixture was warmed to rt, refluxed for 2 h and then stirred at room temperature overnight. The reaction mixture was concentrated to dryness and recrystallised from ^{i}PrOH/MeOH to afford the title compound (290 mg, 1.52 mmol, 79%) as an off-white solid. **^{1}H NMR** (D_2O, 600 MHz) δ_H 4.55 (ABX, *J* = 8.2, 5.3 Hz, 1H, (C2)–H), 3.93 (s, 3H, OCH_3), 3.64 (ABX, *J* = 13.8, 8.2 Hz, 1H, (C3)–H), 3.56 (ABX, *J* = 13.8, 5.3 Hz, (C3)–H′). **^{13}C NMR** (D_2O, 151 MHz) δ_H 168.1 (C1), 55.0 (OCH_3), 50.4 (C2), 38.7 (C3). **HRMS-ESI** $[M+H]^+$ calc. for $C_4H_{11}N_2O_2^+$ 119.0815; obs. 119.0816. **IR** (solid, cm^{-1}): 2869, 2803, 1745, 1601, 1578. Characterisation data were in accordance with literature values.[17]

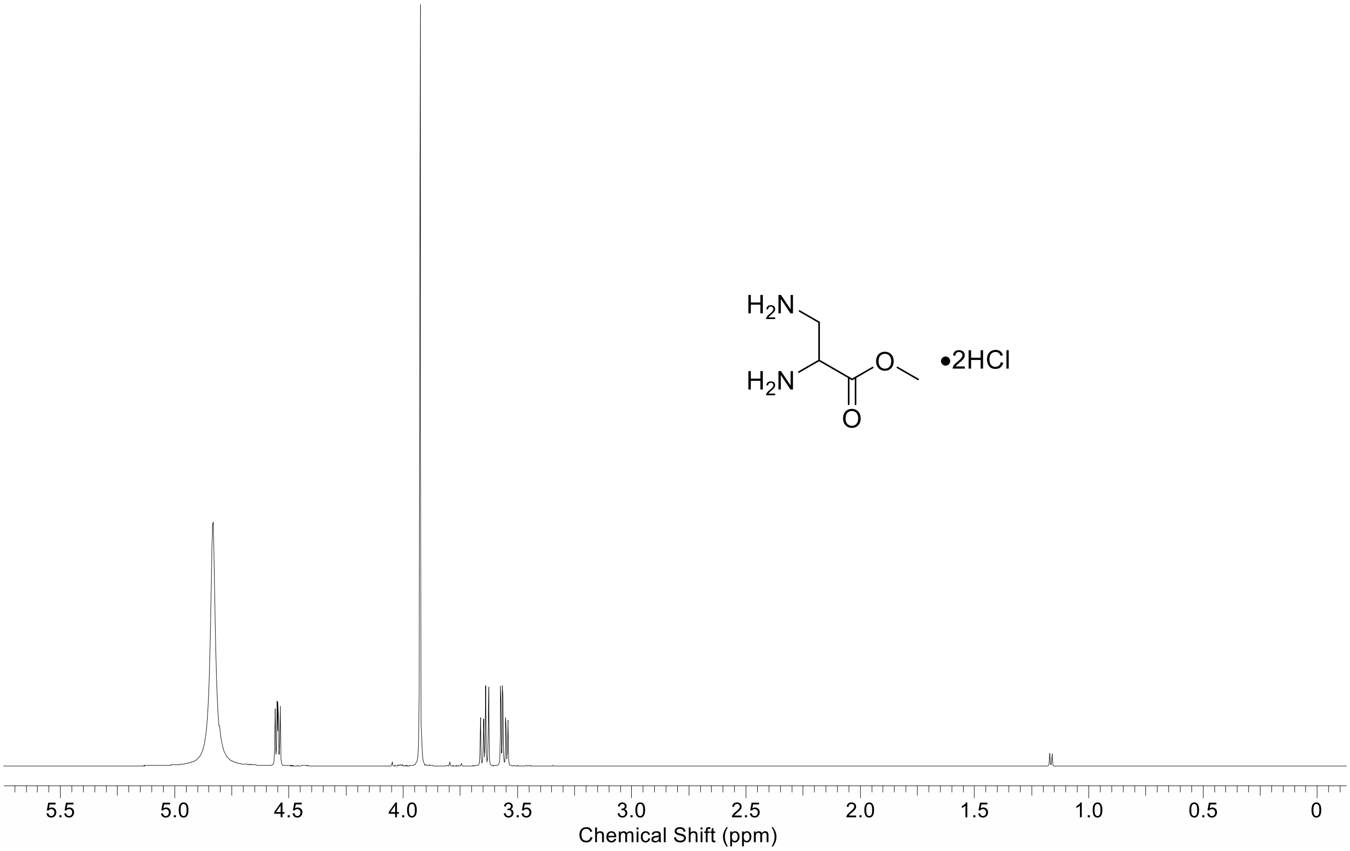

Supplementary Figure 164. ^{1}H NMR (600 MHz, D_2O, 0.0 – 5.5 ppm) spectrum of ***Dpr-OMe****·2HCl.*

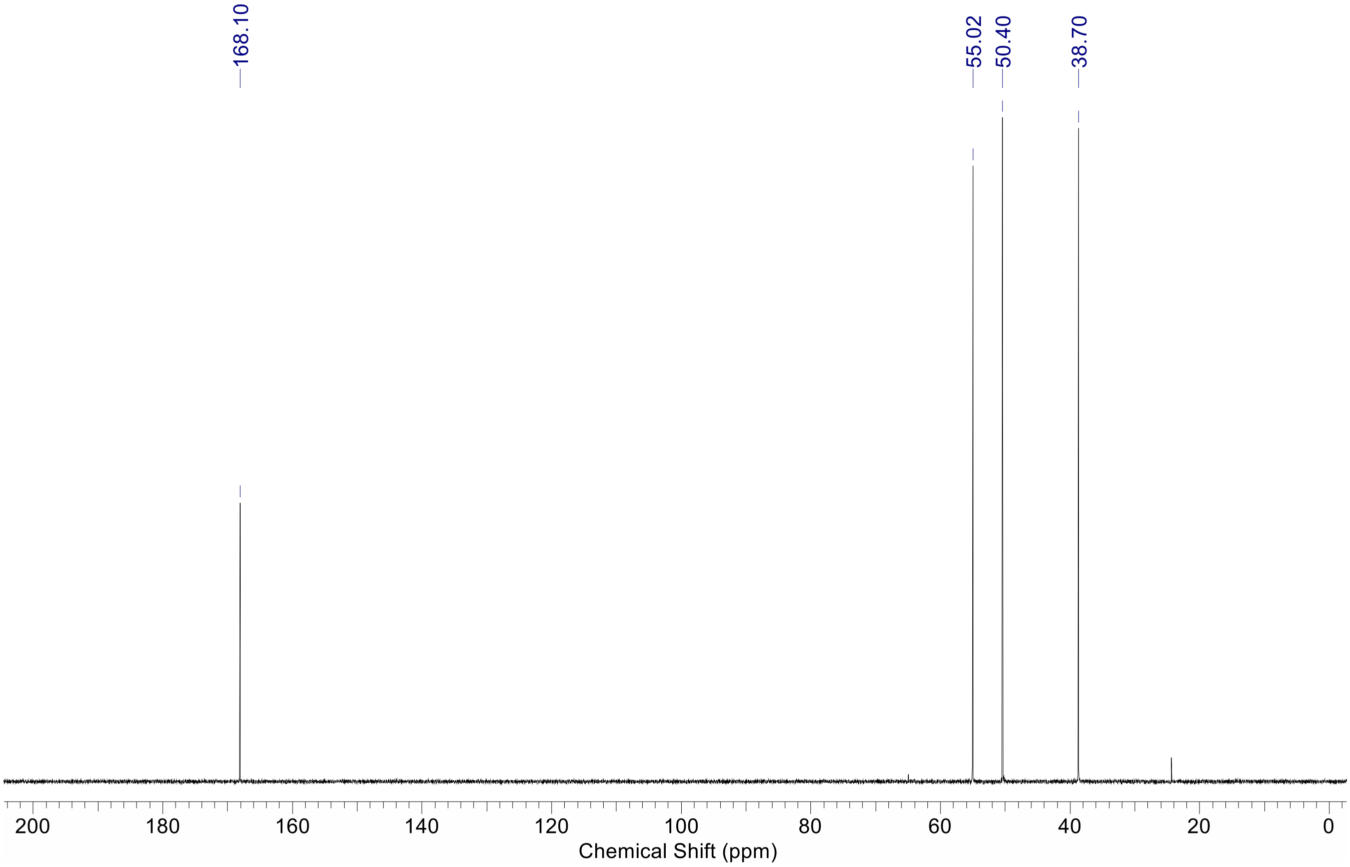

Supplementary Figure 165. ^{13}C NMR (151 MHz, D_2O, 0 – 200 ppm) spectrum of ***Dpr-OMe***·*2HCl.*

Boc-Dpr(Boc)-OMe[18]

To **Dpr-OMe**·2HCl (247 mg, 1.29 mmol) in MeOH (10 mL) was added Boc_2O (846 mg, 3.88 mmol) and $NaHCO_3$ (810 mg, 9.64 mmol). The reaction mixture was stirred at room temperature for 24 h, filtered through Celite®, purified by column chromatography (40:60 petroleum ether to 40:60 petroleum ether/EtOAc; 1:1) and triturated with hexanes to afford the title compound (210 mg, 0.66 mmol, 51%) as a white solid. **^{1}H NMR** (700 MHz, CD_3OD) δ_H 4.22 (br ABX, 1H, (C2)–H), 3.72 (s, 3H, OCH_3), 3.41 (ABX, J = 14.1, 5.1 Hz, 1H, (C3)–H), 3.36 (br ABX, 1H, (C3)–H′), 1.44 (s, 9H, $C(CH_3)_3$), 1.43 (s, 9H, $C(CH_3)_3$). **^{13}C NMR** (176 MHz, CD_3OD) δ_C 173.2 (C1), 158.7 (C3NH(**C**O)OC(CH_3)$_3$), 158.0 (C2NH(**C**O)OC(CH_3)$_3$), 80.9 ((CO)O**C**(CH_3)$_3$), 80.6 ((CO)O**C**(CH_3)$_3$), 55.7 (C2), 53.0 (OCH_3), 42.7 (C3), 28.8 (3C, (CO)OC(**C**H_3)$_3$), 28.8 (3C, (CO)OC(**C**H_3)$_3$). **HRMS-ESI** $[M+H]^+$ calc. for $C_{14}H_{27}N_2O_6^+$ 319.1864; obs. 318.1863. **IR** (solid, cm^{-1}): 3360, 3303, 1742, 1681, 1524. **R$_f$** = 0.50 in 1:1 40:60 petroleum ether/EtOAc. Characterisation data were in accordance with literature values.[18]

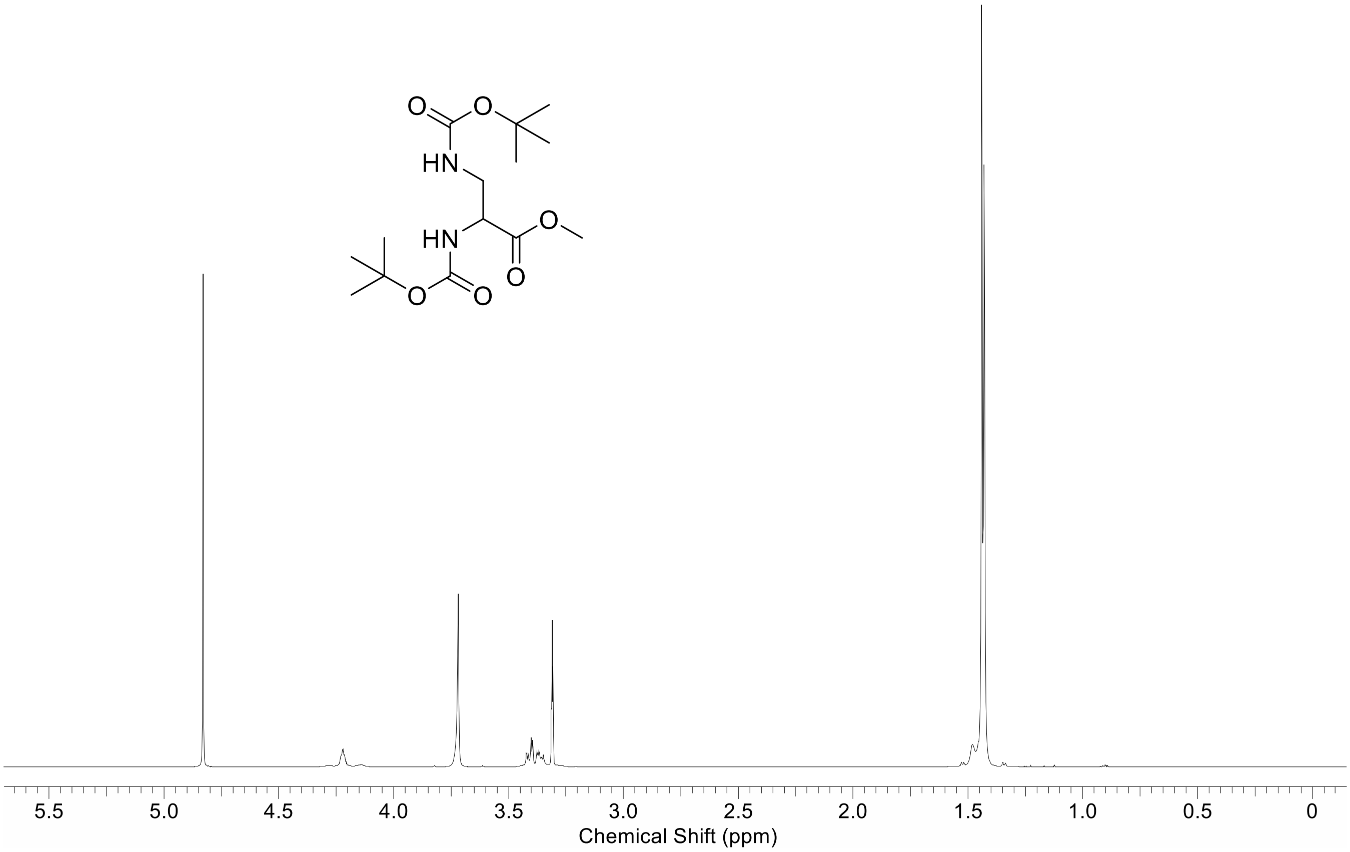

Supplementary Figure 166. ^{1}H NMR (700 MHz, CD_3OD, 0.0 – 5.5 ppm) spectrum of ***Boc-Dpr(Boc)-OMe****.*

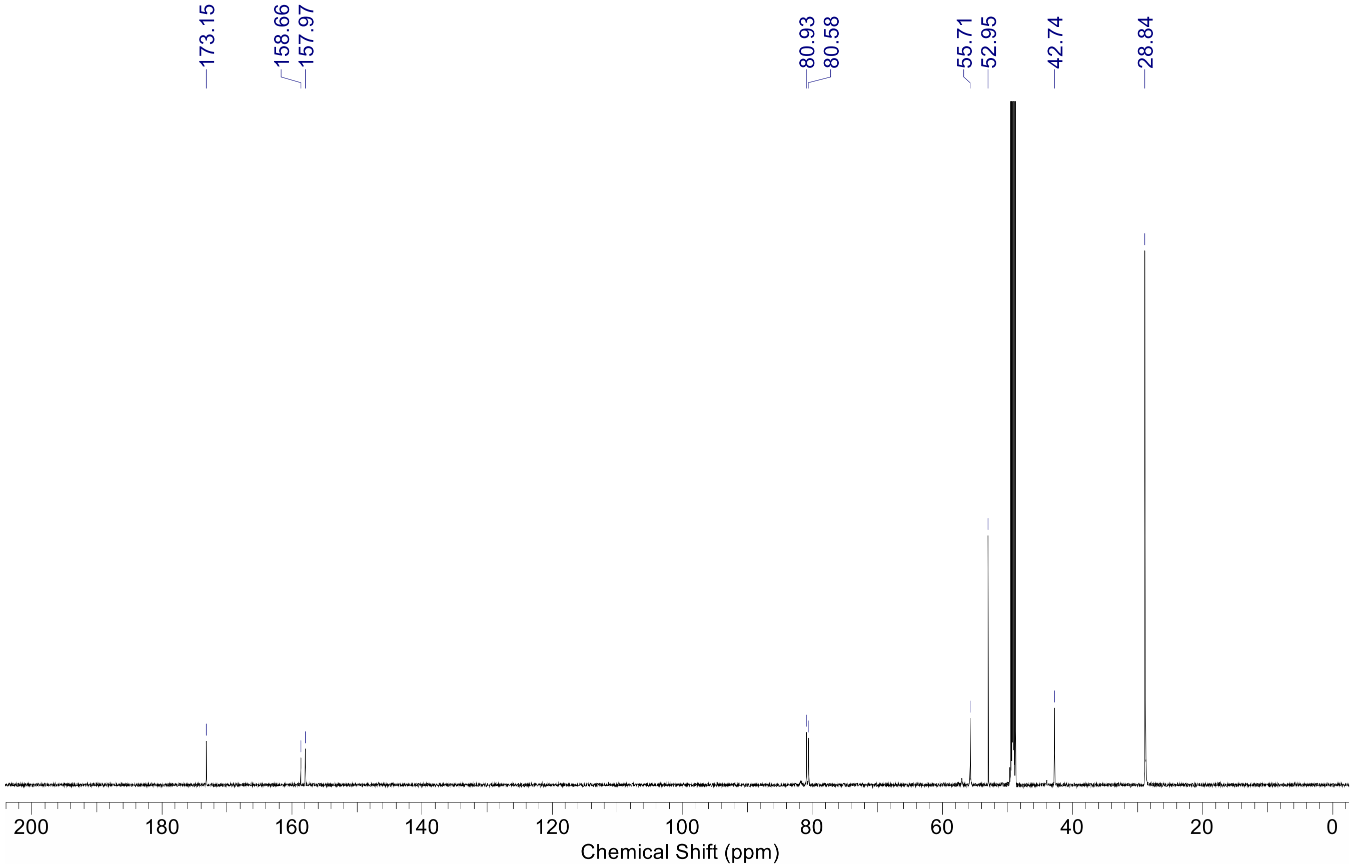

*Supplementary Figure 167. ^{13}C NMR (176 MHz, CD_3OD, 0 – 220 ppm) spectrum of **Boc-Dpr(Boc)-OMe**.*

Boc-Dpr(Boc)-NH$_2$[19]

Boc-Dpr(Boc)-OMe (185 mg, 0.58 mmol) was dissolved in 7 N NH_3 in MeOH (10.0 mL) in a sealed pressure tube. The reaction mixture was heated at 32 °C for 40 h and the volatiles then removed *in vacuo* to afford the title compound (180 mg, 175 mg, 99%) as an off-white powder. **^{1}H NMR** (400 MHz, CD_3OD) δ_H 4.16 (br ABX, 1H, (C2)–H), 3.41 (ABX, *J* = 14.1, 4.8 Hz, 1H, (C3)–H), 3.25 (br ABX, 1H, (C3)–H′), 1.45 (s, 9H, C(CH_3)$_3$), 1.44 (s, 9H, C(CH_3)$_3$). **^{13}C NMR** (151 MHz, $CDCl_3$) δ_C 173.2 (C1), 157.2 ((**C**O)OC(CH_3)$_3$), 156.2 ((**C**O)OC(CH_3)$_3$), 80.4 ((CO)O**C**(CH_3)$_3$), 80.2 ((CO)O**C**(CH_3)$_3$), 55.5 (C2), 42.4 (C3), 28.3 (6C, (CO)OC(**C**H_3)$_3$). **HRMS-ESI** [M+H]$^+$ calc. for $C_{13}H_{26}N_3O_5^+$ 304.1867; obs. 304.1862. **IR** (solid, cm^{-1}): 3378, 2977, 1727, 1666, 1519. Characterisation data were in accordance with literature values.[19]

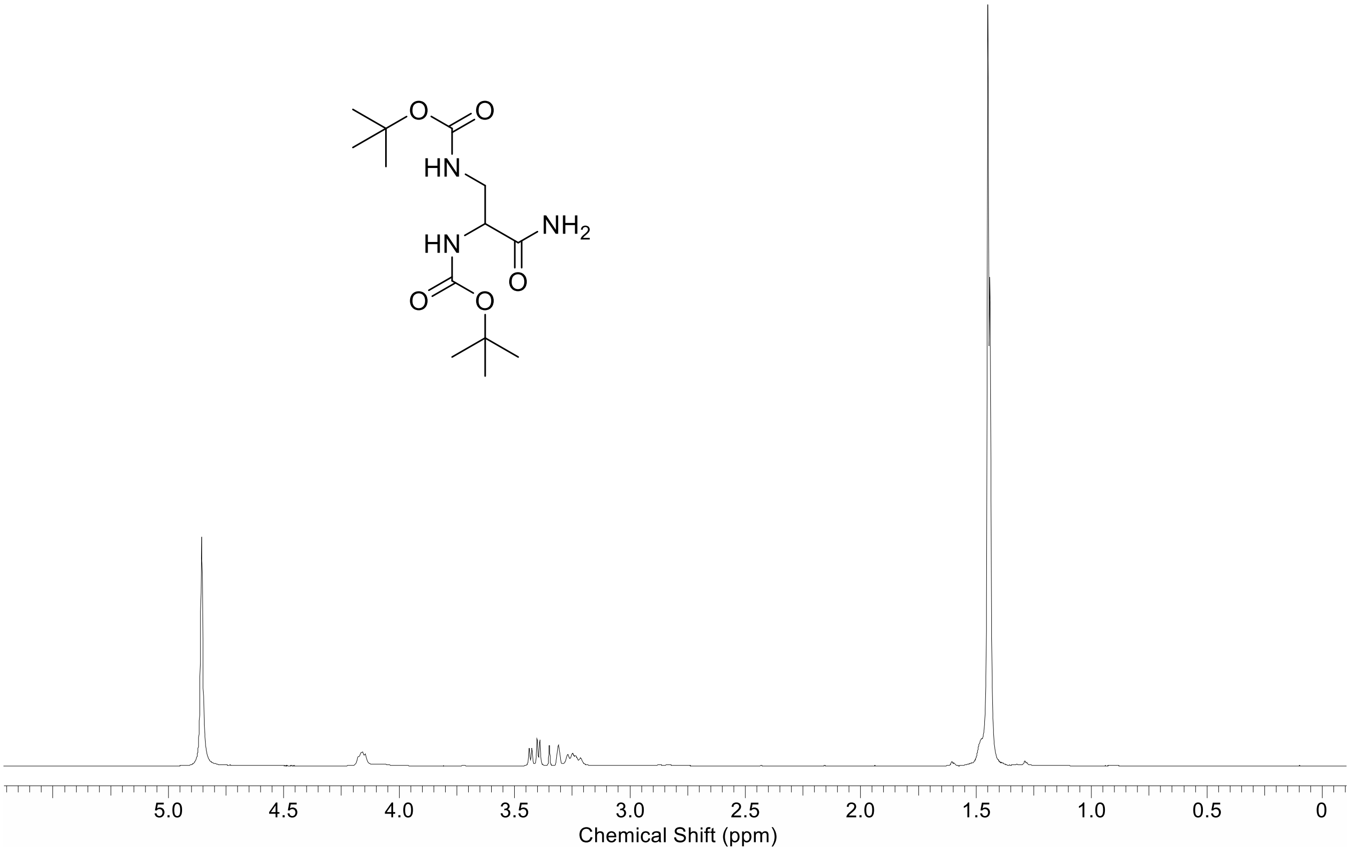

Supplementary Figure 168. ^{1}H NMR (400 MHz, CD_3OD, 0.0 – 5.5 ppm) spectrum of ***Boc-Dpr(Boc)-NH$_2$****.*

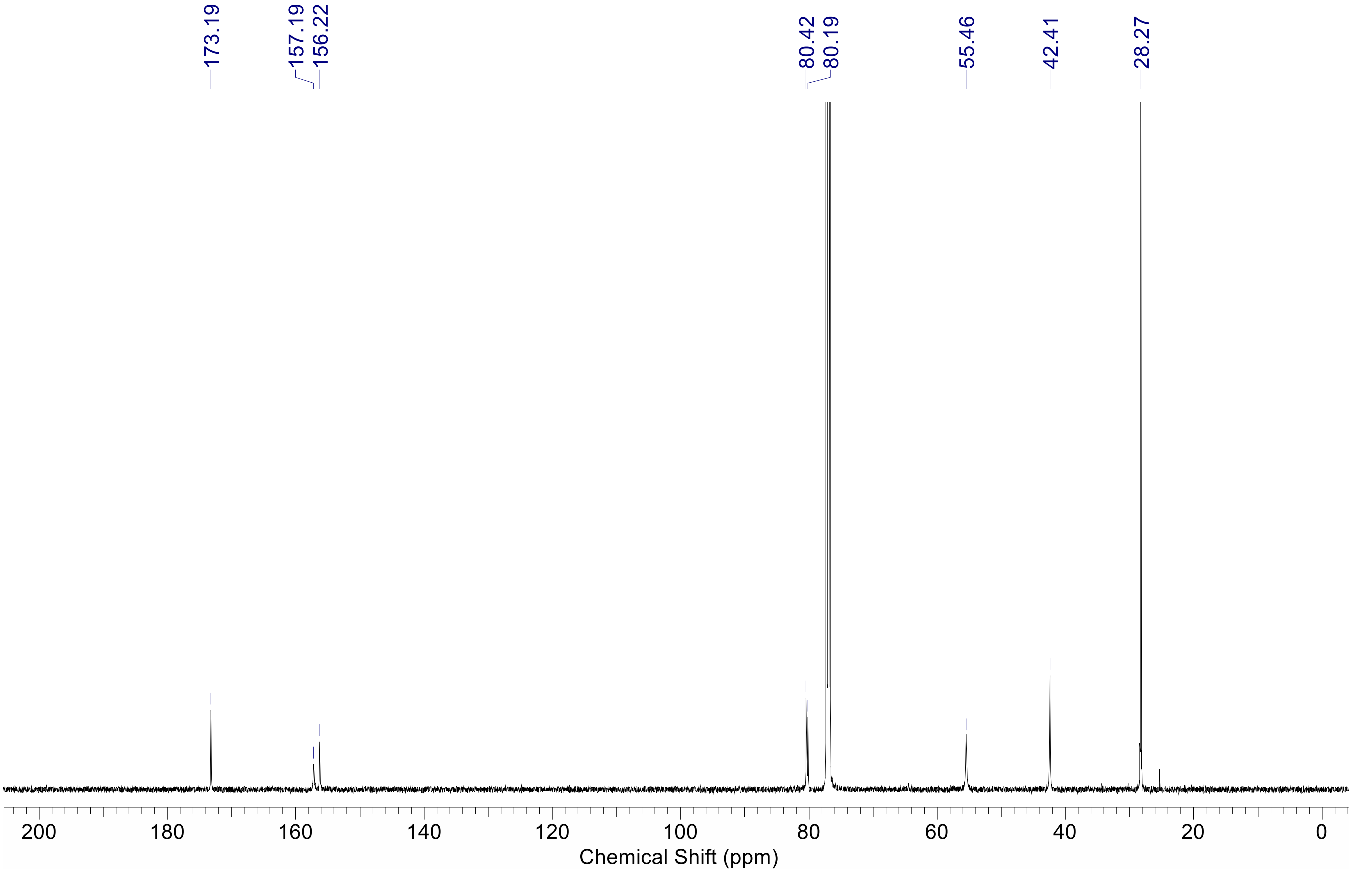

Supplementary Figure 169. ^{13}C *NMR (151 MHz,* $CDCl_3$*, 0 – 200 ppm) spectrum of* ***Boc-Dpr(Boc)-NH₂.***

Boc-Dpr(Boc)-CN

To **Boc-Dpr(Boc)-NH_2** (163 mg, 0.54 mmol) in MeCN/H_2O (2:1, 1.50 mL) was added dichloroacetonitrile (434 µL, 5.40 mmol) and $PdCl_2$ (1.00 mg, 5.64 µmol). The reaction mixture was stirred at 60 °C for 24 h, filtered through a pad of Celite® and volatiles removed *in vacuo*. The crude residue was dissolved in EtOAc (10 mL), washed with H_2O (3 × 5 mL) and the combined organic layers dried over anhydrous Na_2SO_4, filtered and then concentrated *in vacuo*. Purification by column chromatography (40:60 petroleum ether to 7:3 40:60 petroleum ether/EtOAc) followed by trituration with hexanes afforded the title compound (85 mg, 0.30 mmol, 55%) as a white powder. **^{1}H NMR** (600 MHz, CD_3OD) δ_H 4.63 (app t, *J* = 6.9 Hz, 1H, (C2)–H), 3.41 (ABX, *J* = 13.8, 7.1 Hz, 1H, (C3)–H), 3.32 (obs. ABX, *J* = 7.3 Hz, 1H, (C3)–H′), 1.46 ($C(CH_3)_3$), 1.45 ($C(CH_3)_3$). **^{13}C NMR** (151 MHz, CD_3OD) δ_C 158.5 (C3NH(**C**O)OC(CH_3)$_3$), 157.1 (C2NH(**C**O)OC(CH_3)$_3$), 119.2 (C1), 81.6 ((CO)O**C**(CH_3)$_3$), 80.9 ((CO)O**C**(CH_3)$_3$), 44.0 (C2), 43.2 (C3), 28.8 (3C, (CO)OC(**C**H_3)$_3$), 28.7 (3C, (CO)OC(**C**H_3)$_3$). **HRMS-ESI** $[M+H]^+$ calc. for $C_{13}H_{24}N_3O_4^+$ 286.1761; obs. 286.1757. **IR** (solid, cm^{-1}): 3371, 3315, 2977, 2471, 1685, 1517.

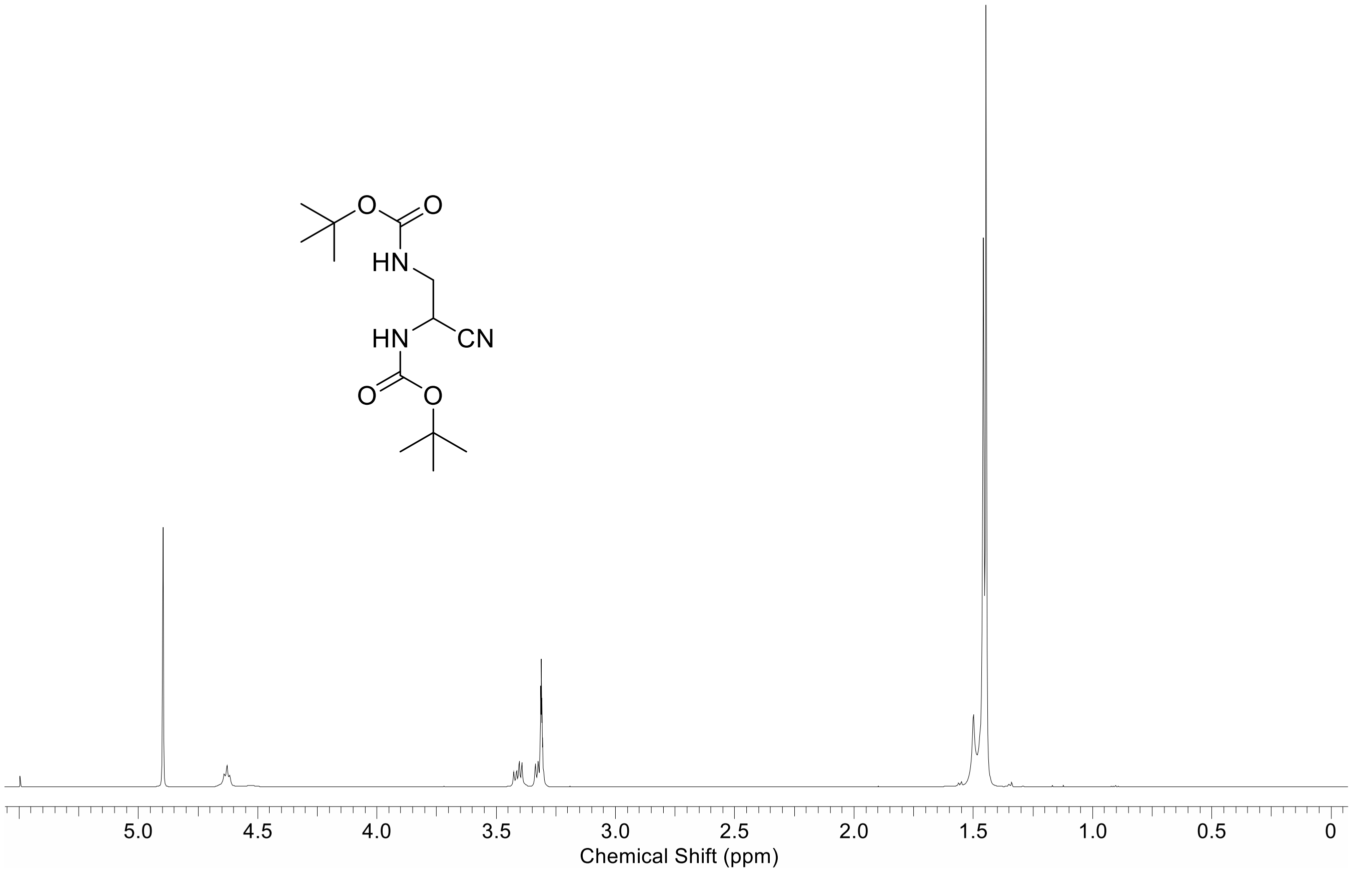

Supplementary Figure 170. ^{1}H NMR (600 MHz, CD_3OD, 0.0 – 5.5 ppm) spectrum of ***Boc-Dpr(Boc)-CN****.*

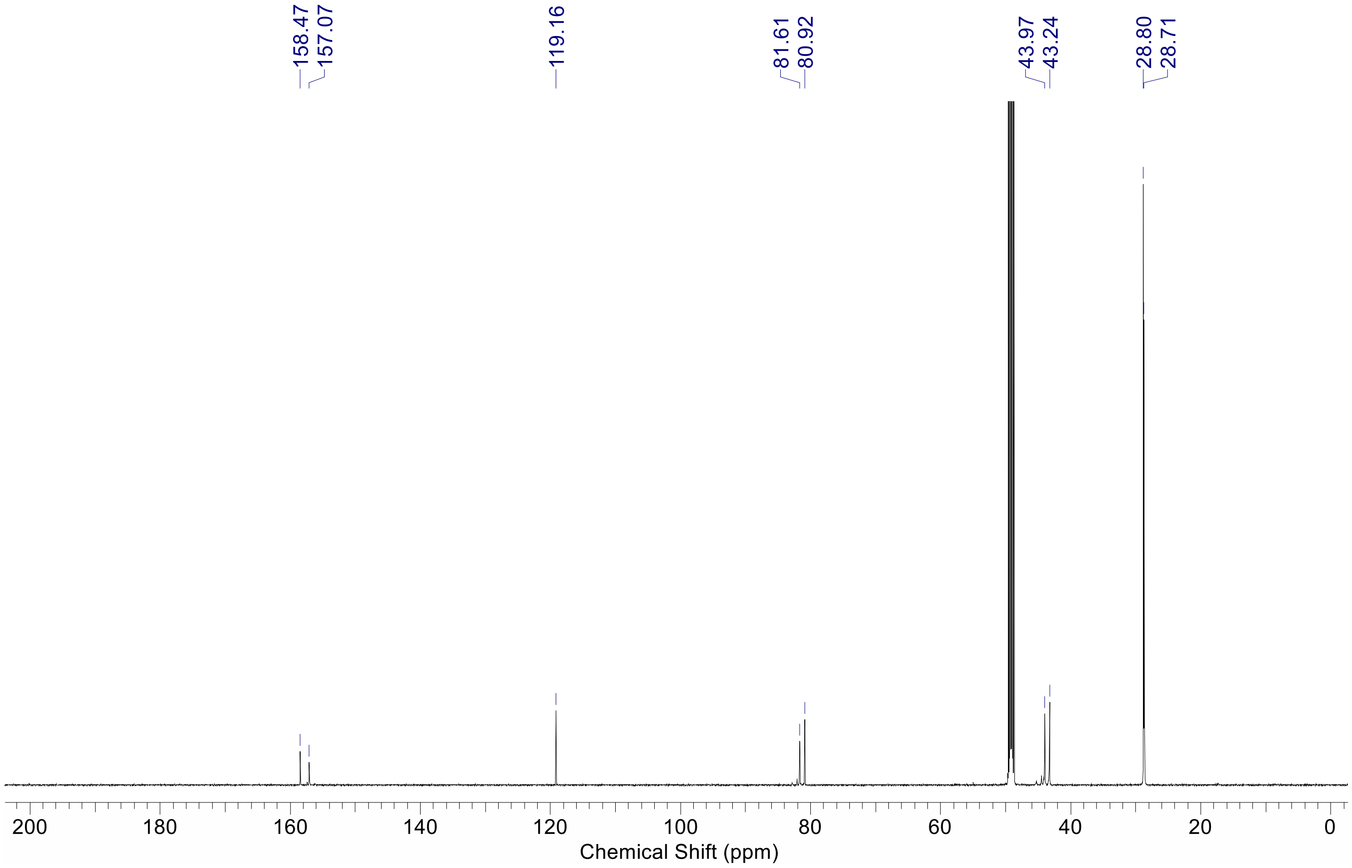

Supplementary Figure 171. ^{13}*C NMR (151 MHz,* CD_3OD*, 0 – 200 ppm) spectrum of* ***Boc-Dpr(Boc)-CN****.*

Dpr-CN·2HCl[20]

To **Boc-Dpr(Boc)-CN** (28.1 mg, 0.10 mmol) in AcOH (0.10 mL) was added 4 M HCl in dioxane (0.20 mL). The reaction mixture was stirred under an Ar atmosphere for 1 h. The white precipitate was isolated by centrifugation and washed with Et_2O (3 × 0.5 mL) to afford the title compound as a white powder (15.0 mg, 0.09 mmol, 97%). **^{1}H NMR** (600 MHz, D_2O) δ_H 4.23 (ABX, *J* = 8.4, 6.0 Hz, 1H, (C2)–H), 3.42 (ABX, *J* = 13.1, 6.0 Hz, 1H, (C3)–H), 3.31 (ABX, *J* = 13.1, 8.4 Hz, 1H, (C3)–H′). **^{13}C NMR** (151 MHz, D_2O) δ_C 119.7 (C1), 42.1 (C3), 41.1 (C2). **HRMS-ESI** $[M+H]^+$ calc. for $C_3H_8N_3^+$ 86.0713; obs. 86.0715.

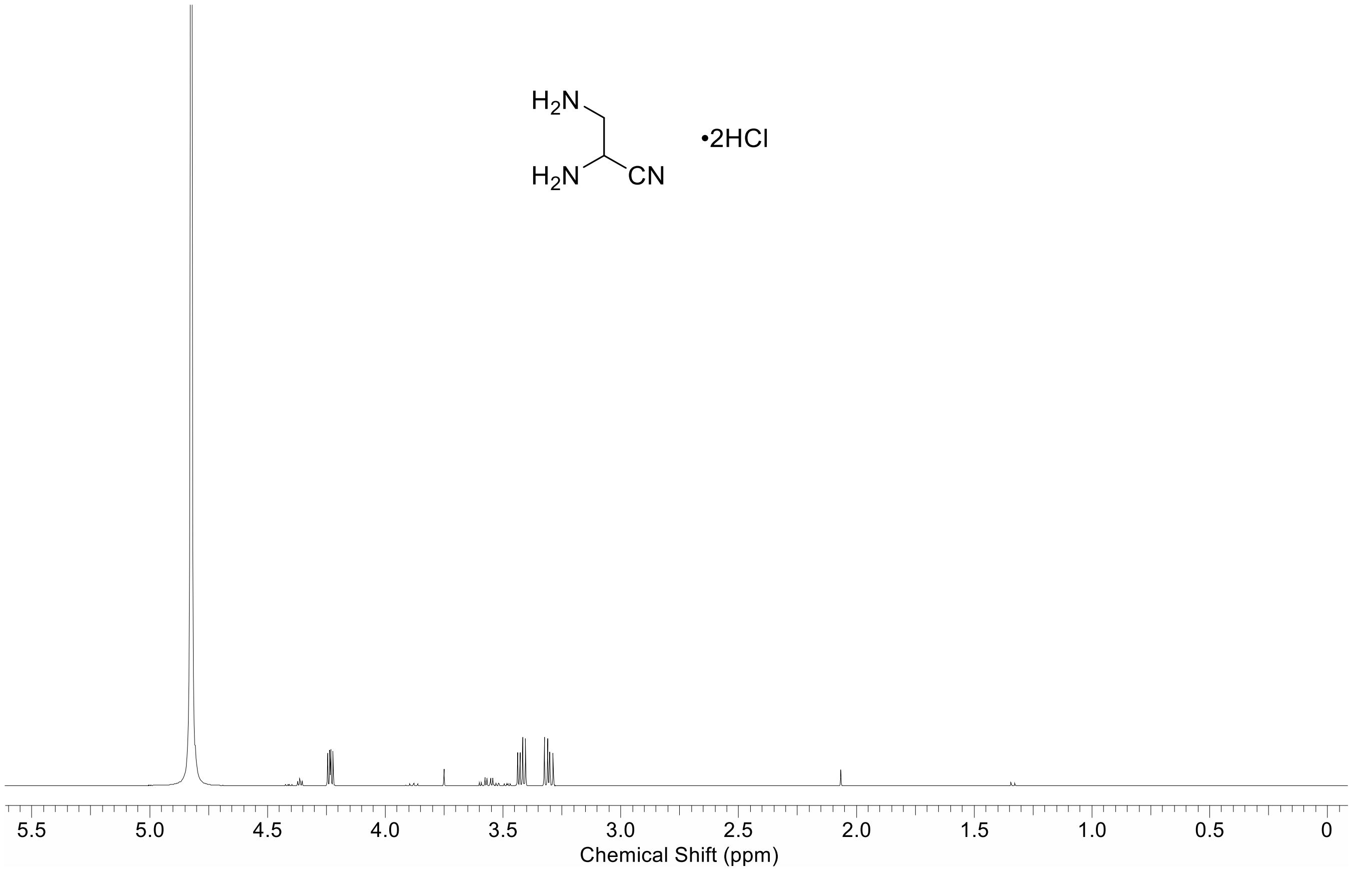

*Supplementary Figure 172. ^{1}H NMR (600 MHz, D_2O, 0.0 – 5.5 ppm) spectrum of **Dpr-CN**·2HCl. Small amounts of what we suspected to be 3,6-bis-aminomethyl-piperazine-2,5-dione[21] were observed as an impurity.*

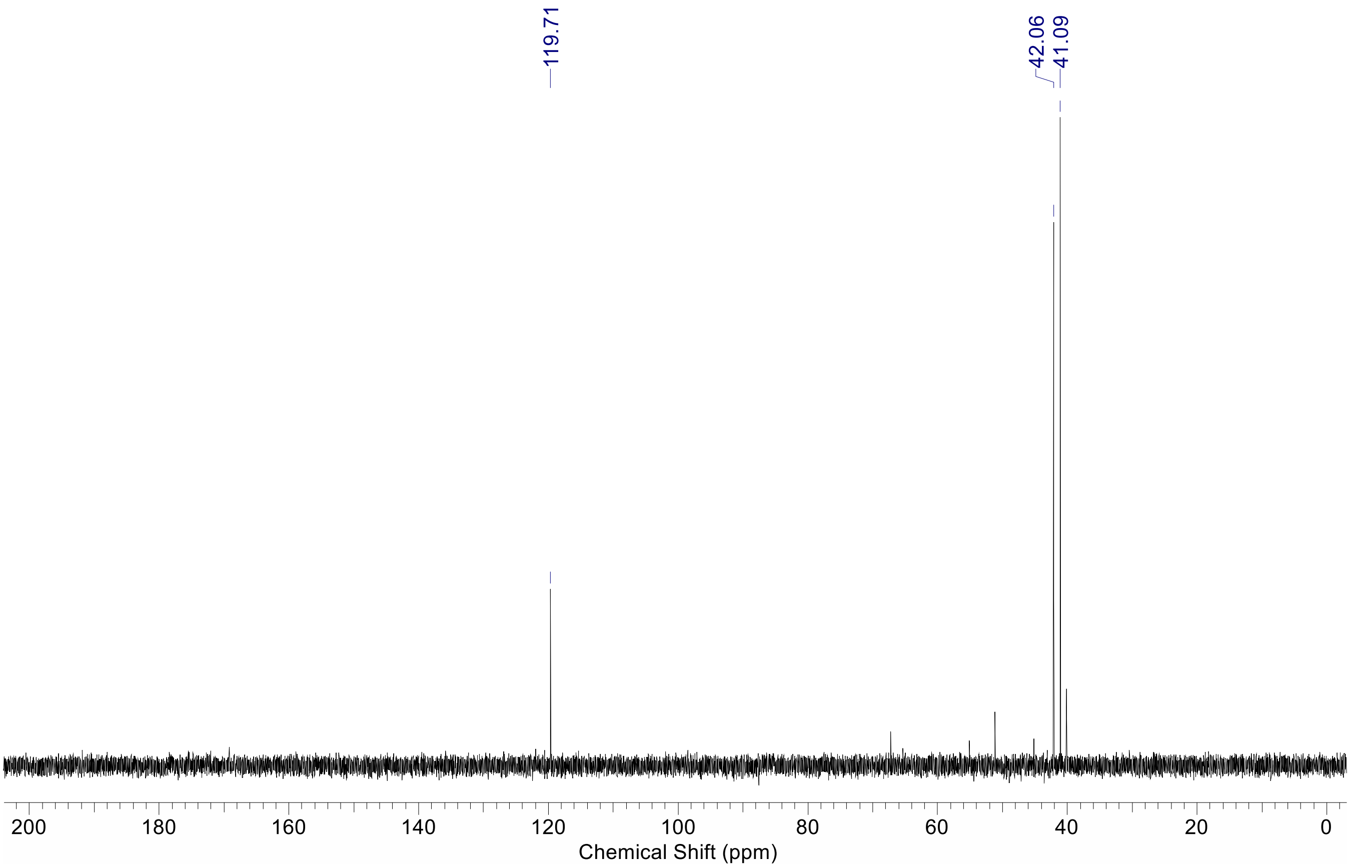

Supplementary Figure 173. ^{13}C NMR (151 MHz, D_2O, 0 – 200 ppm) spectrum of ***Dpr-CN****·2HCl.*

[1] Covington, A. K., Paabo, M., Robinson, R. A. & Bates, R. G. Use of the glass electrode in deuterium oxide and the relation between the standardized pD (pa_D) scale and the operational pH in heavy water. *Anal. Chem.* **1968**, *40*, 700–706.

[2] Canavelli, P., Islam, S. & Powner, M. W. Peptide Ligation by Chemoselective Aminonitrile Coupling in Water. Nature 2019, 571, 546–549.

[3] Stairs, S. et al. Divergent prebiotic synthesis of pyrimidine and 8-oxo-purine ribonucleotides. *Nat. Commun.* **2017**, *8*, 15270.

[4] Uchikawa, O., Fukatsu, K. & Aono, T. Aminothiazole derivatives. I. A convenient synthesis of monocyclic and condensed 5-aminothiazole derivatives. *J. Heterocycl. Chem.* **1994**, *31*, 877.

[5] Foden, C. et. al. Prebiotic Synthesis of Cysteine Peptides That Catalyze Peptide Ligation in Neutral Water. *Science.* **2020**, *370*, 865–869.

[6] Dushin, R. G. Heteroaryl sulfone-based conjugation handles, methods for their preparation, and their use in synthesizing antibody drug conjugates. **2018**, WO2018025168A1.

[7] Hirschmann, R. et al. Synthesis of the first tricyclic homodetic peptide. Use of coordinated orthogonal deprotection to achieve directed ring closure. *Tetrahedron.* **1998**, *54*, 7179–7202.

[8] Schneider, T. L., Walsh, C. T. & O'Connor, S. E. Utilization of Alternate Substrates by the First Three Modules of the Epothilone Synthetase Assembly Line. *J. Am. Chem. Soc.* **2002**, *124*, 11272–11273.

[9] Kildahl-Andersen, G. et al. Synthesis and biological evaluation of zinc chelating compounds as metallo-β-lactamase inhibitors. *Med. Chem. Commun.* **2019**, *10*, 528–537.

[10] Henkel, T. et al. Secondary metabolites by chemical screening, 18. Narbosines, new carbohydrate metabolites from *Streptomyces*. *Liebigs Ann. Chem.* **1991**, 575–580.

[11] Natchev, I. A. Organophosphorus Analogues and Derivatives of the Natural L-Amino Carboxylic Acids and Peptides. I. Enzymatic Synthesis of D-, DL-, and L-Phosphinothricin and Their Cyclic Analogues. *Bull. Chem. Soc. Jpn.* **1988**, *61*, 3699–3704.

[12] Kobayashi, S., Iimori, T., Izawa, T. & Ohno, M. Ph_3P-$(PyS)_2$-CH_3CN as an excellent condensing system for β-lactam formation from β-amino acids. *J. Am. Chem. Soc.* **1981**, *103*, 2046–2048.

[13] Ngamwiriyawong, P. & Vilaivan, T. Synthesis and Nucleic Acids Binding Properties of Diastereomeric Aminoethylprolyl Peptide Nucleic Acids (aepPNA). *Nucleosides Nucleotides Nucleic Acids.* **2011**, *30*, 97–112.

[14] Singh, Y. et al. Novel Cylindrical, Conical, and Macrocyclic Peptides from the Cyclooligomerization of Functionalized Thiazole Amino Acids. *J. Am. Chem. Soc.* **2001**, *123*, 333–334.

[15] Peixoto, S., Nguyen, T. M., Crich, D., Delpech, B. & and Marazano, C. One-Pot Formation of Piperidine- and Pyrrolidine-Substituted Pyridinium Salts via Addition of 5-Alkylaminopenta-2,4-dienals to N-Acyliminium Ions: Application to the Synthesis of (±)-Nicotine and Analogs. *Org. Lett.* **2010**, *12*, 4760–4763.

[16] Zaidan, R. K. & Evans, P. Strategies for the Asymmetric Construction of Pelletierine and its Use in the Synthesis of Sedridine, Myrtine, and Lasubine. *Eur. J. Org. Chem.* **2019**, 5354–5367.

[17] Park, Y. et al. Highly Enantioselective Synthesis of (S)-α-Alkyl-α,β-diaminopropionic Acids via Asymmetric Phase-Transfer Catalytic Alkylation of 2-Phenyl-2-imidazoline-4-carboxylic Acid tert-Butyl Esters. *Org. Lett.* **2009**, *11*, 3738–3741.
[18] Wu, W. et al. Structure−Activity Relationships in Toll-like Receptor-2 Agonistic Diacylthioglycerol Lipopeptides. *J. Med. Chem.* **2010**, *53*, 3198–3213.
[19] Morán-Ramallal, R., Liz, R. & Gotor, V. Bacterial Preparation of Enantiopure Unactivated Aziridine-2-carboxamides and Their Transformation into Enantiopure Nonnatural Amino Acids and vic-Diamines. *Org. Lett.* **2007**, *9*, 521–524.
[20] Cook, A. H. & Smith, E. 632. Studies in the azole series. Part XXIII. A new synthesis of 6-aminopurines. *J. Chem. Soc.* **1949**, 3001–3007.
[21] Naini, S. R. et al. Sulfate Encapsulation in Supramolecular Structures from L-Asparagine-Derived 2,5-Diketopiperazine Scaffolds: Anion Binding. *Eur. J. Org. Chem.* **2014**, *31*, 7015–7022.
